# Stereocontrolled Access to Quaternary Centers by Birch Reduction/Alkylation of Chiral Esters of Salicylic Acids

**DOI:** 10.1021/acs.joc.3c00306

**Published:** 2023-04-11

**Authors:** Ryan A. Kozlowski, Hanh T. Nguyen, Michael E. Lehman, Christopher D. Vanderwal

**Affiliations:** † Department of Chemistry, 1102 Natural Sciences II, University of California, Irvine, California 92697-2025, United States; ‡ Department of Pharmaceutical Sciences, 101 Theory #100, University of California, Irvine, California 92617, United States

## Abstract

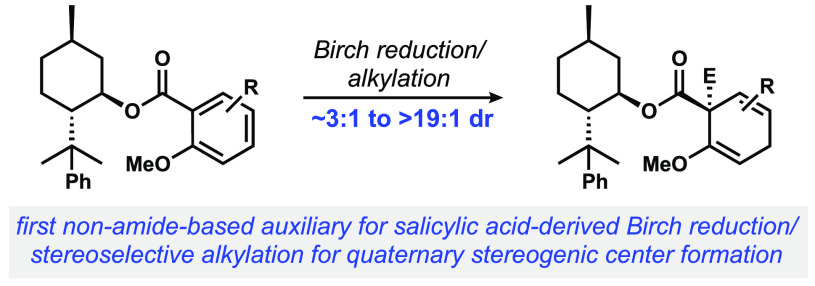

8-Phenylmenthol esters of salicylic acid derivatives undergo efficient Birch reduction and *in situ* diastereoselective alkylations to afford methoxycyclohexadienes bearing new quaternary stereogenic centers. The use of an ester-based auxiliary is a designed improvement over the use of prolinol-derived amides, which are expensive and often very difficult to cleave.

The venerable Birch reduction, initially disclosed in 1944, has served as a key transformation in the syntheses of many complex molecules.^1^ The ability to take feedstock or readily available aromatic compounds and obtain high-value-added, functional-group-rich products has proven central to the efficient synthesis of many targets.^2^ Although recent advances include ammonia-free^3^ or even metal-free^4^ Birch-type reduction conditions, the classic Birch reduction using a group 1 or 2 metal dissolved in ammonia remains the most widely used method.

The presence of an anion-stabilizing group on the substrate arene—usually a carboxylic acid derivative—drives the regiochemical control of the Birch reduction and generally results in the formation of a stabilized carbanion. The enolate so formed can be used productively for C–C bond formation, generating a new quaternary carbon.^5^ The stereoselective synthesis of quaternary carbons remains a significant challenge for synthetic chemistry,^6^ and the Birch reduction/alkylation reaction is a powerful tool for directly forming quaternary carbons from aromatic rings. This Birch reduction/alkylation process of carboxylic acid derivatives was rendered diastereoselective by Schultz through the use of a proline-derived chiral auxiliary attached as an amide.^7,8^ This gives rise to enantioenriched materials following removal of the chiral auxiliary. Schultz and others have synthesized several natural products using this method;^8b,9^ however, the auxiliary removal is often nontrivial, requiring harsh or substrate-tailored conditions, or multiple steps.^8b,9,10^ Herein we disclose the first example of a nonamide based chiral auxiliary for use in a diastereoselective Birch reduction/alkylation reaction to produce highly functionalized cyclohexadienes.

During the course of efforts toward the total synthesis of a natural product, we aimed to use Schultz’s auxiliary to set a critical quaternary stereogenic center starting from a substituted salicylic acid derivative (Figure 1). While we observed that the diastereoselective Birch reduction/alkylation reaction proceeded well, we found that the steric bulk of the auxiliary and proximal functionality prevented subsequent desired transformations. Additionally, cleanly removing the chiral auxiliary proved impossible, as a result of poor reactivity of the amide and/or undesired side reactivity. As a result of these difficulties, we aimed to develop a more easily removable chiral auxiliary for a diastereoselective Birch reduction/alkylations of benzenoid systems.

**Figure 1:**
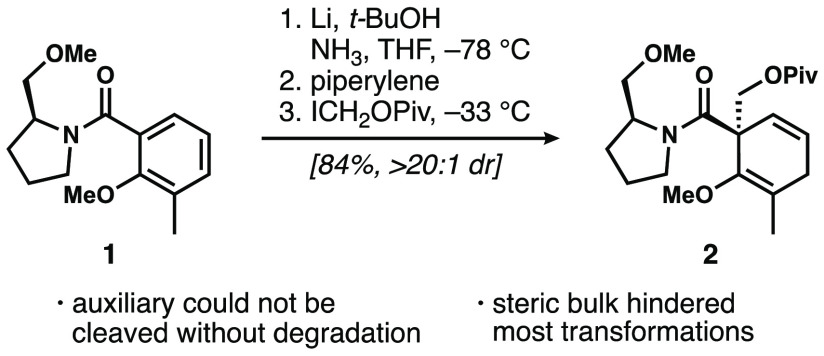
Successful Schultz-type Birch reduction/alkylation yields a product from which the auxiliary cannot be removed.

Our attention turned toward the chiral pool in the hope of discovering an ester-based chiral auxiliary, with the assumption that hydrolytic removal would be much easier than with the amides; furthermore, a reductive option is also available that is not trivial with amides, which would likely leave the prolinol auxiliary attached via an amine linkage. Initially, and unsurprisingly, borneyl, isomenthyl, and menthyl esters of 3,*O*-dimethyl salicylic acid (**3**, Figure 2) provided essentially no diastereoselectivity (∼1:1) using iodomethyl pivalate as the electrophile. We attribute this lack of diastereoselection to the lack of any obvious mechanism for conformational restriction, and thus transfer of chirality in the alkylation step. 8-Phenylmenthol esters (or esters of other chiral arene-bearing alcohols) have been found to deliver high diastereoselectivity in situations where they can engage in a π-stacking interaction with the substrate.^11^ The most relevant work is that by Donohoe and co-workers on the diastereoselective Birch reduction of pyrroles using “cumyl” and (−)-8-phenylmenthol esters.^12^ However, until recently, 8-arylmenthols were prohibitively expensive and nontrivial to synthesize. Importantly, recent work by Shenvi and co-workers has demonstrated that (−)-8-phenylmenthol and analogues bearing other aromatic substituents can easily be synthesized in two steps from pulegone,^13^ making this family of chiral auxiliaries more easily accessible than it has been previously.

**Figure 2:**
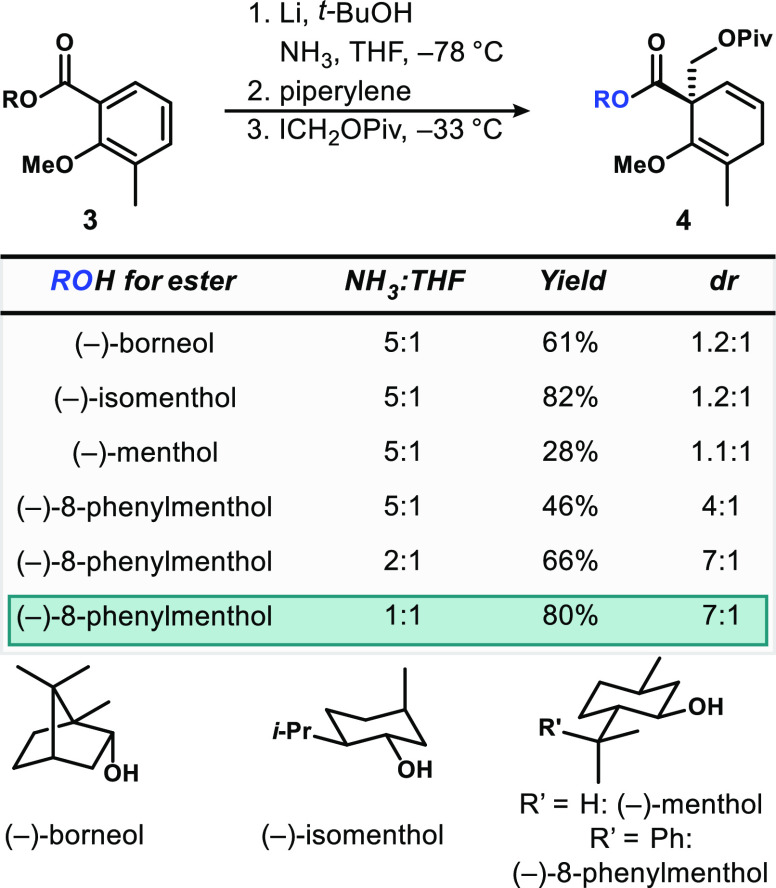
Identification of a competent ester-based auxiliary.

In a promising initial result, we found that the (−)-8-phenylmenthyl ester of 3,*O*-dimethyl salicylic acid was reduced and alkylated in 46% yield and with 4:1 dr, using iodomethylpivalate as the electrophile (Figure 2). We ascribe this increase in diastereoselectivity to a potential π-stacking interaction between the phenyl ring on the chiral auxiliary and the extended enolate resulting from the reduced aromatic ring. The moderate yield and diastereoselectivity was attributed to the poor solubility of **3** in ammonia, as observed by the reaction mixture becoming a viscous, difficult-to-stir, milky-white suspension at −78 °C. Increasing the amount of THF (5:1 NH_3_/THF → 2:1) improved solubility and increased the yield and d.r. to 66% and 7:1, respectively. Increasing the ratio further to 1:1 ammonia/THF increased the yield to 80% and kept the d.r. at a respectable 7:1. Given the ease of synthesis of various arylated menthol derivatives according to the protocol of Shenvi and co-workers,^13^ 1-naphthyl, 2-naphthyl, 4-fluorobenzenyl, and 3,5-bistrifluoromethylbenzenyl derivatives were investigated to see if extending the aromatic system or making the aromatic ring on the chiral auxiliary more electron-poor would increase the hypothesized π-stacking interaction with the electron-rich Birch reduction intermediate.^14^ Unfortunately and not unexpectedly, competitive reduction of the more electron-deficient aromatic rings of these auxiliaries prevented validation of this hypothesis. Given these results, we proceeded with (−)-8-phenylmenthol because we observed no competitive reduction of the phenyl group, good yields of the desired product, and useful levels of diastereoselectivity.

To explore the scope of this reaction, we looked to vary the substitution pattern on the aromatic ring and the identity of the electrophile (Figure 3). For otherwise unsubstituted *O-*methyl salicylic esters (products **5**–**9**), as well as 3-methyl- (products **10**–**14**) and 5-methyl-substituted substrates (products **15**–**19**), benzylic, methyl, and alkyl halides were competent electrophiles, in all cases resulting in ≥3:1 dr. Particularly interesting electrophiles that generate synthetically malleable products include iodomethyl pivalate (generating **8** and **13** with high selectivities) and bromoacetonitrile (**18** and **19**). 6-Methyl salicylate derivatives (products **20**–**23**) performed particularly well with ∼20:1 dr in all cases except for with methyl iodide. If one trend with electrophiles did emerge from these examples, it is that smaller electrophiles tend toward slightly diminished stereoselectivities. The efficiency of reaction held for the 4-methyl substrate, generating **24** with two new stereogenic centers; the configuration of the distal one at C4 is presumably not controlled by the distal auxiliary, resulting in a mixture of diastereomers.^15^ These results overall strongly suggest that a broad range of 8-phenylmenthyl esters of *O-*alkyl salicylates will undergo Birch reduction/alkylation with synthetically useful levels of selectivity. Furthermore, the reaction can be performed preparatively, with generation of **10** done on 2-g scale in 83% yield with the same 5:1 dr; further chromatography led to a 63% yield of diastereomerically enriched product (11:1 dr).

**Figure 3:**
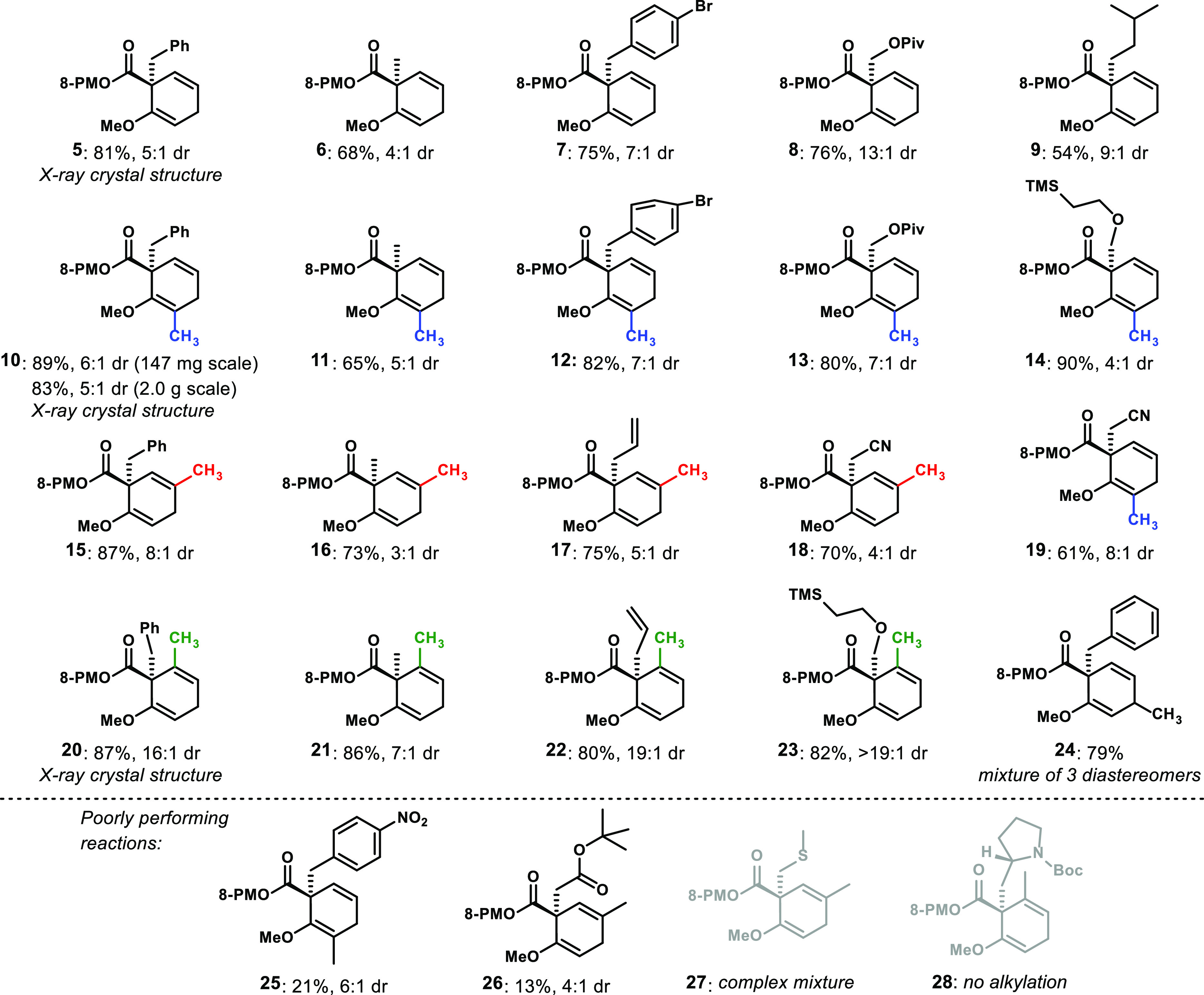
Products of Birch reduction/diastereoselective alkylation of 8-phenylmenthol esters of salicyclic acid derivatives (8-PM = 8-phenylmenthyl). The relative configurations of **5** and **20** were assigned via X-ray crystallography; others assigned by analogy.

There were some cases wherein this protocol proved poorly effective (products **25**–**28**). Nitrobenzyl electrophiles—designed to try to encourage crystallinity in the products—decomposed under the reaction conditions, leading to low yields. *tert*-Butyl haloacetates were poorly reactive, resulting in low conversion in the alkylation step. Attempted reaction of the intermediate enolate with chloromethyl methyl sulfide led to decomposition. Finally, and not surprisingly, β-branched electrophiles, such as the prolinol-derived reactant that might have formed **28**, were completely unreactive.

Looking to expand beyond salicylates, we examined 2-methyl and 2-fluoro substrates **29** and **30** (Figure 4). Although these compounds each underwent reduction/alkylation in reasonable yields, the diastereoselectivities were well below 2:1, strongly implicating chelation as a stereocontrol element in the more selective alkylations, as we had anticipated.^16^ Similarly, 3-substituted substrates **31** and **32** performed efficiently in the reaction, but again with essentially no stereocontrol. Perhaps not surprisingly, 3-chloro reactant **33** decomposed during the reduction step, presumably via Cl–C bond reduction.

**Figure 4:**
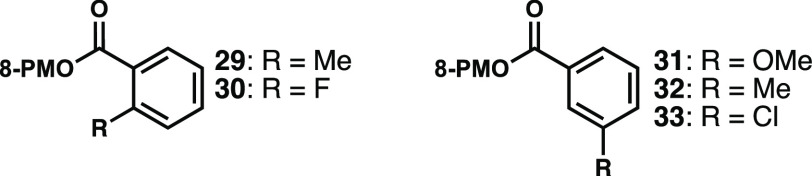
Substrates that resulted in poor diastereoselectivity or decomposed during reduction (8-PM: 8-phenylmenthyl).

We attribute the requirement of the 2-alkoxy group to its likely coordination to lithium during and after formation of the extended enolate following arene reduction. The resulting conformational control, along with likely π-stacking of the phenyl group with the cross-conjugated dienolate, leads to shielding of one enolate face, allowing for selective alkylation (Figure 5). We expect that the reactive conformation is approximated by structure **34a**, in which no obvious deleterious nonbond interactions exist, and both chelation and π-stacking can be operative; the products for which we have unambiguous structural assignment from X-ray crystallography^14^ are consistent with this model. Rotation about the enolate C–O bond can produce conformation **34b**, largely devoid of π-stacking and engendering significant nonbonded interactions between the carbinol H and R group at C6 on the arene; circumstantially, this idea is supported by the improved selectivity with R = CH_3_ compared with R = H (see examples **20**–**23** compared to their analogues without the C6 methyl, Figure 3). Conformation **34c**, while maintaining π-stacking, results in nonbonded interactions with axial C–H bonds on the phenylmenthol ring. Of course, without a C2-alkoxy group to coordinate the lithium, the substrate may form the enolate in either configuration, potentially leading to poor diastereoselectivity even if π-stacking is maintained.

**Figure 5:**
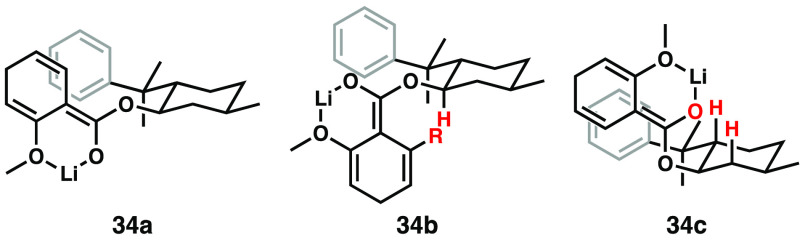
Conformational analysis of the reactive cross-conjugated dienolate.

One of the hallmarks of auxiliary-based diastereoselection is that separation can often lead to the isolation of stereochemically homogeneous products; unfortunately, in many of the cases described in Figure 3, significant upgrading of stereochemical purity via column chromatography was infeasible. Removal of the chiral auxiliary can be accomplished efficiently and with good recovery of (−)-8-phenylmenthol using lithium aluminum hydride (Figure 6). However, there remain some limitations of the ester-based auxiliary in that, like the Schultz amide system, simple hydrolysis is often ineffective, almost certainly owing to the substantial steric encumbrance to the approach of any nucleophile to π* of the ester carbonyl. Lewis acid activation and dealkylation conditions were similarly ineffective.^14^ However, the ability to achieve simple reductive removal is an advance relative to amides in many cases, wherein reductive formation of the amine incorporating the auxiliary is generally undesired.

**Figure 6:**
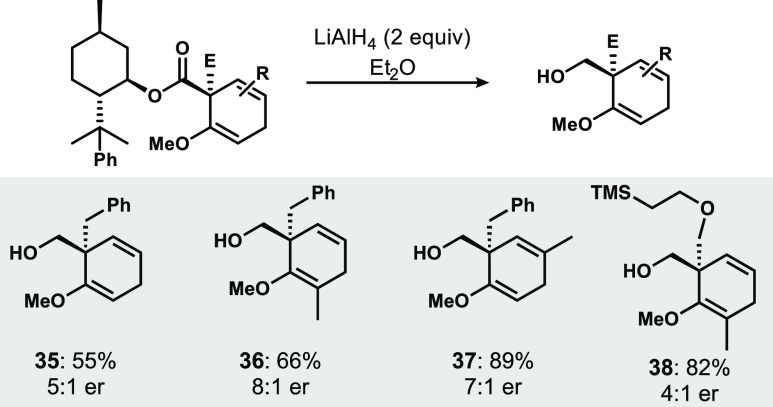
Representative examples of reductive auxiliary cleavage.

We have developed an auxiliary-controlled stereoselective Birch reduction/alkylation reaction, making use of esters of the readily available 8-phenylmenthol. The product 1,4-cyclohexadienes bearing quaternary stereogenic centers are in many cases formed in good yield and high diastereoselectivity. The ester linkage to the chiral auxiliary is easily reduced, rendering the overall process a marked improvement over previous amide-based chiral auxiliaries. Despite the requirement of 2-alkoxy substitution in almost all cases, substitution at the 3, 5, and 6 positions on the aromatic ring are tolerated, as are a variety of electrophiles. In short, this method provides chemists with a simple, scalable, and diastereoselective reaction to form all-carbon quaternary centers for use in pursuit of highly functionalized cyclohexane scaffolds.

## Data Availability

The data underlying this study are available in the published article and its Supporting Information.

## Supporting Information Available

Experimental procedures, characterization data, NMR spectra for all new compounds, and X-ray crystallographic data (PDF)
