# Supplementary material for: Stereocontrolled Access to Quaternary Centers by Birch Reduction/Alkylation of Chiral Esters of Salicylic Acids
Source: J Org Chem. 2023 Apr 11;88(9):6232–6. doi: 10.1021/acs.joc.3c00306 (PMC10167686; doi:10.1021/acs.joc.3c00306)
Supplement: Supplementary file 1 — jo3c00306_si_001.pdf [file jo3c00306_si_001.pdf]

Stereocontrolled Access to Quaternary Centers by Birch Reduction/Alkylation of Chiral Esters of Salicylic Acids

Ryan A. Kozlowski,[a§] Hanh T. Nguyen,[a§] Michael E. Lehman,[a] and Christopher D. Vanderwal[a,b,*]
[a] *Department of Chemistry, 1102 Natural Sciences II, University of California, Irvine, CA 92697, USA.*
[b] *Department of Pharmaceutical Sciences, 101 Theory #100, University of California, Irvine, CA 92617, USA.*
**cdv@uci.edu*

[§]These authors contributed equally.

Supporting Information

Table of Contents

1. Materials and Methods……………………………………………………………… SI-2
2. Experimental Procedures and Characterization Data…….………………………… SI-3
   IR and NMR spectra…………………………………………………………… SI-33
3. SFC Data ……………………………………………..…………………………… SI-178
4. X-Ray Crystallographic Data…………………………………………….………… SI-184
5. References……………………………………………..………………………….. SI-235

1. Materials and methods

All reactions were performed in oven-dried (120 °C) or flame-dried glassware under a dry argon atmosphere unless otherwise noted. Phenylsilane was purchased from Oakwood Chemicals and used without further purification. Reaction solvents including hexanes (C_6H_{14}, Fisher, ACS Grade), dichloromethane (CH_2Cl_2, Fisher, ACS Grade), diethyl ether (Et_2O, Fisher, BHT stabilized, ACS Grade), benzene (C_6H_6, Fisher), and tetrahydrofuran (THF, Fisher), were dried by percolation through a column packed with neutral alumina and a column packed with Q5 reactant, a supported copper catalyst for scavenging oxygen, under a positive pressure of argon. Acetone (Fisher) was dried over neutral alumina. Pyridine was purified by distillation from CaH_2. Grignard reagents were titrated using salicylaldehyde phenylhydrazone in THF.

Solvents used for work-up and flash column chromatography were hexanes (Fisher or EMD, ACS Grade) and ethyl acetate (EtOAc, Fisher, ACS Grade). Flash column chromatography was performed using EMD Millipore 60 Å (0.040–0.063 mm) mesh silica gel (SiO_2). Analytical thin-layer chromatography was performed on Merck silica gel 60 F254 TLC plates. Visualization was accomplished with UV light (254 or 210 nm), and/or p-anisaldehyde, vanillin, potassium permanganate ($KMnO_4$), phosphomolybdic acid (PMA), or ceric ammonium molybdate (CAM) stains, using heat as a developing agent. Chloroform-*d* ($CDCl_3$, D 99.8%, DLM-7) and benzene-*d6* (C_6D_6, D 99.5%, DLM-1-100) were purchased from Cambridge Isotope Laboratories. K_2CO_3 (anhydrous, 99%, Alfa Aesar), $NaHCO_3$ (ACS grade, Fisher), NaOH (ACS grade, Macron or Fisher), DMAP (ACS grade, Fisher) and DCC (ACS grade, Fisher) were purchased and used without further purification.

Proton and carbon magnetic resonance spectra (^1H NMR and ^{13}C NMR) were recorded at 298K on a Bruker CRYO500 (500 MHz, ^1H; 125 MHz, ^{13}C) or a Bruker AVANCE600 (600 MHz, ^1H; 151 MHz, ^{13}C) spectrometer with residual solvent resonance used as the internal standard for ^1H NMR: $CHCl_3$ at 7.26 ppm and C_6H_6 at 7.16 ppm, and deuterated solvent as the internal standard for ^{13}C NMR: $CDCl_3$ at 77.16 ppm and C_6D_6 at 128.1 ppm). ^1H NMR data are reported as follows: chemical shift, multiplicity (s = singlet, d = doublet, t = triplet, q = quartet, p = pentet, h = heptet, dd = doublet of doublets, dp = doublet of pentets, ddd = doublet of doublet of doublets, td = triplet of doublets, tdd = triplet of doublet of doublets, qd = quartet of doublets, m = multiplet, br s = broad singlet). Coupling constants (J) are reported in Hertz (Hz). The raw fid files were processed into the included NMR spectra using MestReNova 10.0 (Mestrelab Research S.L.). Mass spectrometry data were obtained from the University of California, Irvine Mass Spectrometry Facility. High resolution mass spectra (HRMS) were recorded on a Waters LCT Premier S2 spectrometer using ESI-TOF (electrospray ionization-time of flight) and data are reported in the form of (m/z). Diastereomeric ratios (d.r.) were determined by ^1H NMR integrations and measured prior to purification. Analytical samples for ^1H NMR characterization not indicative of substrate diastereoselectivity are noted where applicable. Enantiomeric ratios (e.r.) were determined by chiral SFC analysis using an Agilent Technologies HPLC (1200 series) system and Aurora A5 Fusion with CHIRALCEL® OD-H column or using a Mettler SFC supercritical CO_2 analytical

chromatography system with CHIRALPAK® AD-H column, OD-H column, and IC-3 column obtained from Daicel Chemical Industries, Ltd.

2. Experimental Procedures and Characterization Data

General procedure for methylation.

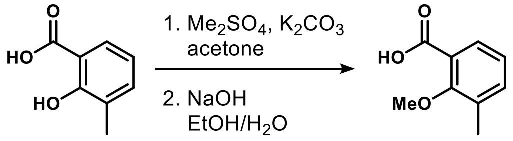

S1 **S2**

Carboxylic acid (S2). To a flame-dried 50 mL round bottom flask equipped with a magnetic stir bar was added K_2CO_3 (25.4 g, 184 mmol, 4.0 equiv.) and 3-methyl salicylic acid (7.0 g, 46 mmol, 1.0 equiv.). The flask was slowly evacuated and refilled with argon atmosphere three times and allowed to cool under positive pressure of argon. Acetone (20 mL) was added to form a thick slurry. Dimethyl sulfate (17.4 mL, 184 mmol, 4.0 equiv.) was added dropwise at room temperature. The reaction mixture was stirred until deemed complete by aliquot ^{1}H NMR (2 days). Once the reaction was complete, the suspension was filtered through Celite® using acetone as eluent, and the filtrate was concentrated *in vacuo*.[1] The resulting clear oil was dissolved in ethanol (50 mL) and added to a stirred solution of NaOH (9.2 g, 230 mmol, 10 equiv.) in water (50 mL) at room temperature. After the reaction was judged complete by TLC (30 min), the solution was concentrated *in vacuo* and cooled to 0 °C. The reaction mixture was then acidified with aqueous HCl (12 M, 20 mL) to pH = 1 followed by the addition of water (50 mL). The resulting mixture was extracted with EtOAc (3 x 100 mL). The combined organic extracts were dried over anhydrous $MgSO_4$, filtered through a pad of Celite®, and concentrated *in vacuo* to give carboxylic acid **S2** as a white solid (7.3 g, 95% yield). ^{1}H and ^{13}C NMR spectra were consistent with those previously reported.[1]

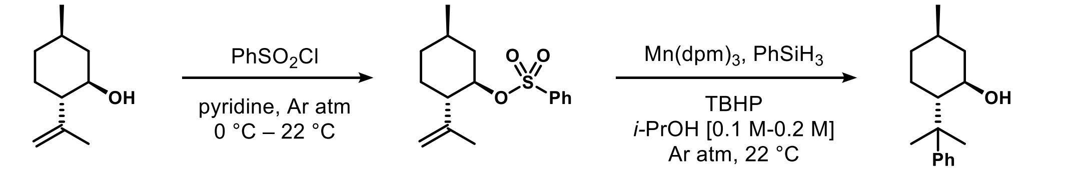

(–)-Isopulegol **S3** **(–)-8-phenylmenthol (S4)**

Sulfonate ester (S3). A flame-dried 100 mL round bottom flask equipped with a magnetic stir bar was charged with (–)-8-isopulegol (2.5 mL, 15.0 mmol, 1.0 equiv.) and anhydrous pyridine (19 mL) under an argon atmosphere. The solution was cooled to 0 °C in an ice bath, and benzenesulfonyl chloride (2.3 mL, 18.0 mmol, 1.2 equiv.) was slowly added to the reaction flask. The reaction mixture was warmed to room temperature and stirred until judged complete by TLC (2 hours). After addition of cold water, a white precipitate formed, which was filtered off, rinsed

with excess cold water, and dried under vacuum to obtain **S3** as an off-white solid (4.4 g, 99% yield). The solid was used without further purification. ^{1}H and ^{13}C NMR spectra were consistent with those previously reported.[2]

(–)-8-Phenylmenthol (S4). This procedure was adapted from literature precedent.[2] A flame-dried 1 L round bottom flask equipped with a magnetic stir bar was charged with sulfonate ester **S3** (15.0 g, 51 mmol, 1.0 equiv.) and $Mn(OAc)_3$•$2H_2O$ (13.7 g, 51 mmol, 1.0 equiv.). The flask was evacuated and refilled with argon three times. Following addition of degassed *i*-PrOH (500 mL), 2-hydroperoxy-2-methylpropane (5.5 M in nonane, 18.5 mL, 102 mmol, 2.0 equiv.) and 2,2,6,6-tetramethylheptane-3,5-dione (2.1 mL, 10 mmol, 0.2 equiv.) were added. After 20 min, $PhSiH_3$ (6.6 mL, 54 mmol, 1.1 equiv.) was added dropwise. After 2 days, the reaction was concentrated *in vacuo* to remove most of the *i*-PrOH. The resulting viscous solution was diluted with water (100 mL) and extracted with hexane (3 x 100 mL). The combined organic extracts were dried over Na_2SO_4, filtered through a pad of Celite®, and concentrated *in vacuo*. The resulting oil was purified by flash column chromatography (Et_2O, 3-5% hexane/EtOAc) to obtain (–)-8-phenylmenthol **S4** as a colorless oil (8.4 g, 71% yield). ^{1}H and ^{13}C NMR spectra were consistent with those previously reported.[2]

General procedure for esterification.

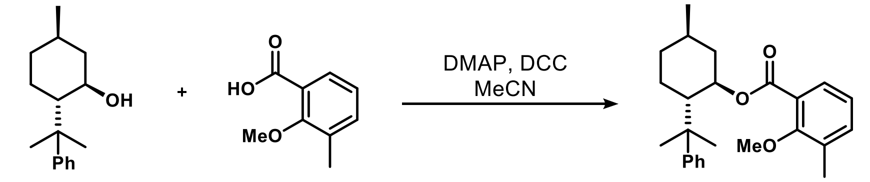

(–)-8-Phenylmenthol S2 S5

Ester (S5). A flame-dried 100 mL round bottom flask was charged with 2-methoxy-3-methylbenzoic acid (2.1 g, 12 mmol, 1.5 equiv.) and DMAP (0.2 g, 1.6 mmol, 0.2 equiv.), then evacuated and refilled with an argon atmosphere. A second flame-dried 50 mL round bottom flask was charged with DCC (2.5 g, 12 mmol, 1.5 equiv.), MeCN (12.4 mL), and (–)-8-phenylmenthol (1.9 g, 8.2 mmol, 1.0 equiv.). The suspension was transferred via syringe to the reaction flask and stirred at room temperature for 3 days or until the starting material was fully consumed by TLC (2 days). After completion, the reaction mixture was diluted with hexanes (30 mL), filtered through Celite®, and concentrated *in vacuo*. The thick yellow crude oil residue was purified by column chromatography (SiO_2, 2% EtOAc in hexanes) to afford **S5** as a colorless oil (2.9 g, 94% yield).
^{1}H NMR (500 MHz, $CDCl_3$) δ 7.27 (dt, *J* = 6.9, 3.5 Hz, 3H), 7.16 (t, *J* = 7.6 Hz, 2H), 7.09 (dd, *J* = 7.8, 1.8 Hz, 1H), 7.01 (t, *J* = 7.3 Hz, 1H), 6.90 (t, *J* = 7.6 Hz, 1H), 5.09 (td, *J* = 10.7, 4.4 Hz, 1H), 3.79 (s, 3H), 2.30 (s, 3H), 2.16 – 2.03 (m, 2H), 1.63 (td, *J* = 14.3, 3.3 Hz, 2H), 1.53 (dp, *J* = 10.0, 3.5 Hz, 1H), 1.35 (s, 3H), 1.27 (s, 3H), 1.19 – 1.04 (m, 2H), 0.91 – 0.86 (m, 4H).
^{13}C{^{1}H} NMR (126 MHz, $CDCl_3$) δ 165.2, 158.6, 151.4, 134.9, 132.5, 129.2, 128.1, 125.6, 125.2, 125.1, 123.3, 75.1, 61.5, 50.7, 42.0, 40.1, 34.8, 31.5, 27.1, 26.9, 26.8, 22.0, 16.1.

HRMS (ES+) m/z calc'd for $C_{25}H_{32}O_3Na$ $[M + Na]^+$: 403.2249; found 403.2237.
IR (film) ν 2953, 2922, 2868, 1716, 1594, 1294 cm^{-1}.

General procedure for Birch reduction/alkylation of ester.

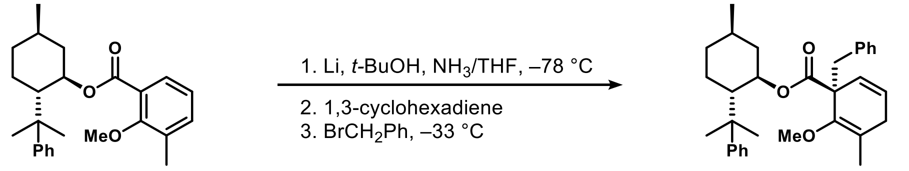

S5 → 10

Diene (10).
147 mg scale reaction: Liquid NH_3 (4 mL) was distilled into a flame-dried 25 mL three-neck round bottom flask at –78 °C equipped with a glass-encased stir bar and a dry ice condenser. From a separate flame-dried 10 mL pear-shaped flask, a solution of ester **S5** (147 mg, 0.4 mmol, 1.0 equiv.) and *t*-BuOH (1 M in THF, 0.4 mL, 0.4 mmol, 1.0 equiv.) in anhydrous THF (4 mL) was added to the reaction flask via syringe. Freshly cut lithium wire (8.5 mg, 1.2 mmol, 3.0 equiv.) was added in two pieces, which resulted in a sustained deep blue solution after 10 min. The reaction mixture was stirred for 30 min at –78 °C before adding 1,3-cyclohexadiene (0.1 mL, 1.0 mmol, 2.5 equiv.) dropwise which led to discharge of the color. After stirring for an additional 10 min at –78 °C, benzyl bromide (0.14 mL, 1.2 mmol, 3.0 equiv.) was added dropwise. The solution was allowed to warm up slowly to –33 °C in a MeOH/water/dry ice bath and was stirred for an additional 2 h, or until the reaction was complete by TLC. Once the reaction was complete, it was quenched with NH_4Cl (62 mg, 1.2 mmol, 3.0 equiv.) and warmed slowly to room temperature to allow liquid NH_3 to evaporate. Brine (10 mL) was added, and the mixture was extracted with hexanes (3 x 10 mL). The combined organic extracts were dried over anhydrous $MgSO_4$, filtered through a pad of Celite®, and concentrated *in vacuo* to afford a yellow oil. The crude mixture was purified by flash column chromatography (SiO_2, 2% EtOAc in hexanes) to afford **10** as a colorless oil (162 mg, 89% yield, 6:1 d.r.).
2.0 g scale reaction: Prepared according to the procedure shown above, using ester **S5** (2.0 g, 5.3 mmol, 1.0 equiv.) and benzyl bromide (1.9 mL, 15.9 mmol, 3.0 equiv.) to give **10** as a colorless oil (2.1 g, 83% yield, 5:1 d.r.). Crystals of suitable quality for X-ray diffraction were formed in CH_2Cl_2 after standing in the freezer at –18 °C for a week.
^{1}H NMR (600 MHz, $CDCl_3$) δ 7.36 – 7.29 (m, 4H), 7.19 (tt, *J* = 6.3, 2.1 Hz, 1H), 7.16 – 7.11 (m, 3H), 7.04 – 6.99 (m, 2H), 5.58 (dt, *J* = 9.9, 3.4 Hz, 1H), 5.27 (dt, *J* = 9.9, 2.0 Hz, 1H), 4.91 (td, *J* = 10.6, 4.3 Hz, 1H), 3.70 (s, 3H), 3.10 (d, *J* = 13.2 Hz, 1H), 2.51 (d, *J* = 13.2 Hz, 1H), 2.45 (dt, *J* = 22.1, 2.5 Hz, 1H), 2.08 (ddd, *J* = 12.2, 10.5, 3.5 Hz, 1H), 2.03 – 1.96 (m, 1H), 1.88 – 1.81 (m, 1H), 1.58 (dt, *J* = 12.9, 3.4 Hz, 1H), 1.53 (s, 3H), 1.50 – 1.45 (m, 2H), 1.36 (s, 3H), 1.23 (s, 3H), 1.10 – 0.96 (m, 2H), 0.88 (d, *J* = 6.5 Hz, 3H), 0.86 (qd, 1H).
^{13}C{^{1}H} NMR (151 MHz, $CDCl_3$) δ 173.4, 151.5, 147.4, 137.7, 131.0, 128.3, 127.1, 126.9, 126.0, 125.85, 125.76, 125.5, 117.5, 76.1, 61.3, 54.9, 50.1, 42.0, 40.3, 39.8, 34.7, 33.1, 31.5, 28.0, 27.3, 26.0, 22.0, 16.2.

HRMS (ES+) m/z calc'd for $C_{32}H_{40}O_3Na$ [M + Na]$^+$: 495.2875, found 495.2864.
IR (film) ν 2952, 2922, 2868, 1715, 1594, 1293, 699 cm^{-1}.

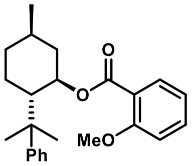

S6

Ester (S6). Prepared according to **general procedure for esterification**, using (–)-8-phenylmenthyl (1.0 g, 4.1 mmol, 1.0 equiv.), 2-methoxybenzoic acid (1.3 g, 8.2 mmol, 2.0 equiv.), DMAP (100 mg, 0.82 mmol, 0.2 equiv.), and DCC (1.3 g, 6.1 mmol, 1.5 equiv.) to give **S6** as a colorless oil (1.4 g, 93% yield).
^{1}H NMR (500 MHz, $CDCl_3$) δ 7.39 (ddd, *J* = 8.3, 7.3, 1.9 Hz, 1H), 7.30 – 7.26 (m, 2H), 7.19 – 7.13 (m, 3H), 7.04 – 6.99 (m, 1H), 6.90 (dd, *J* = 8.4, 1.0 Hz, 1H), 6.79 (td, *J* = 7.5, 1.0 Hz, 1H), 5.08 (td, *J* = 10.7, 4.4 Hz, 1H), 3.88 (s, 3H), 2.12 (ddd, *J* = 12.2, 10.5, 3.5 Hz, 1H), 2.08 – 2.01 (m, 1H), 1.62 (ddq, *J* = 13.5, 6.7, 3.3 Hz, 2H), 1.53 – 1.46 (m, 1H), 1.35 (s, 3H), 1.26 (s, 3H), 1.18 – 1.02 (m, 2H), 0.88 (d, *J* = 6.7 Hz, 4H).
^{13}C{^{1}H} NMR (151 MHz, $CDCl_3$) δ 164.8, 159.6, 151.7, 133.3, 131.9, 128.1, 125.6, 125.1, 120.2, 119.9, 111.8, 74.8, 56.0, 50.7, 42.0, 40.0, 34.8, 31.5, 27.1, 27.0, 26.6, 22.0.
HRMS (ES+) m/z calc'd for $C_{24}H_{30}O_3$ [M + Na]$^+$: 389.2093; found 389.2094.
IR (film) ν 2952, 2920, 1716, 1599, 1244 cm^{-1}.

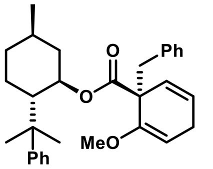

5

Diene (5). Prepared according to **general procedure for Birch reduction/alkylation of ester**, using ester **S6** (100 mg, 0.27 mmol, 1.0 equiv.) and benzyl bromide (0.10 mL, 0.82 mmol, 3.0 equiv.) to give **5** as a colorless oil (101 mg, 81% yield, 5:1 d.r.). Crystals of suitable quality for X-ray diffraction were formed in DCM (50 mg/mL) after vapor diffusion of pentane at room temperature.
^{1}H NMR (600 MHz, $CDCl_3$) δ 7.30 (d, *J* = 4.3 Hz, 4H), 7.20 – 7.14 (m, 4H), 7.05 – 7.01 (m, 2H), 5.78 – 5.72 (m, 1H), 5.40 (dt, *J* = 9.8, 2.1 Hz, 1H), 4.85 (td, *J* = 10.6, 4.2 Hz, 1H), 4.59 (t, *J* = 3.5 Hz, 1H), 3.48 (s, 3H), 3.38 (d, *J* = 13.3 Hz, 1H), 2.74 (d, *J* = 13.4 Hz, 1H), 2.64 – 2.56 (m, 1H), 2.17 (dq, *J* = 22.2, 3.0 Hz, 1H), 1.97 (d, *J* = 12.5 Hz, 1H), 1.94 – 1.87 (m, 1H), 1.49 (dd, *J* = 13.1, 2.9 Hz, 1H), 1.44 (d, *J* = 6.7 Hz, 1H), 1.32 (s, 3H), 1.28 – 1.26 (m, 1H), 1.27 (s, 3H), 1.02 – 0.86 (m, 2H), 0.85 (d, *J* = 6.4 Hz, 3H), 0.74 (qd, *J* = 12.8, 3.5 Hz, 1H).
^{13}C{^{1}H} NMR (151 MHz, $CDCl_3$) δ 172.9, 151.3, 150.9, 137.8, 130.8, 128.1, 127.4, 126.8, 126.5, 126.02, 125.98, 125.4, 94.6, 76.5, 53.64, 53.57, 50.4, 41.6, 40.4, 40.3, 34.7, 31.5, 29.9, 27.6, 26.2, 23.6, 21.9.

HRMS (ES+) m/z calc'd for $C_{31}H_{38}O_3$ $[M]^+$: 458.2821, found 458.2808.
IR (film) ν 2944, 2867, 1718, 1689, 1601, 1223, 697 cm^{-1}.

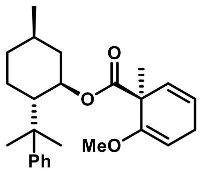

6

Diene (6). Prepared according to **general procedure for Birch reduction/alkylation of ester**, using ester **S6** (87 mg, 0.24 mmol, 1.0 equiv.) and methyl iodide (0.040 mL, 0.72 mmol, 3.0 equiv.) to give **6** as a colorless oil (62 mg, 68% yield, 4:1 d.r.).
^{1}H NMR (499 MHz, $CDCl_3$) δ 7.27 (d, J = 5.0 Hz, 4H), 7.18 – 7.10 (m, 1H), 5.78 (dtd, J = 9.8, 3.4, 1.1 Hz, 1H), 5.42 (dt, J = 9.8, 2.0 Hz, 1H), 4.77 (td, J = 10.6, 4.3 Hz, 1H), 4.70 (td, J = 3.6, 1.1 Hz, 1H), 3.47 (s, 3H), 2.93 – 2.75 (m, 2H), 1.96 – 1.75 (m, 2H), 1.49 – 1.40 (m, 1H), 1.34 (s, 3H), 1.29 (s, 3H), 1.23 (s, 3H), 1.22 – 1.15 (m, 1H), 0.97 – 0.83 (m, 2H), 0.81 (d, J = 6.5 Hz, 4H), 0.70 (tdd, J = 12.9, 11.4, 3.4 Hz, 1H).
^{13}C{^{1}H} NMR (151 MHz, $CDCl_3$) δ 173.4, 154.6, 150.8, 129.1, 128.1, 126.0, 125.4, 124.7, 91.8, 76.3, 54.0, 50.5, 48.0, 41.6, 40.5, 34.7, 31.4, 30.4, 27.6, 26.6, 23.5, 23.1, 21.9.
HRMS (ES+) m/z calc'd for $C_{25}H_{34}O_3Na$ $[M + Na]^+$: 405.2406, found 405.2395.
IR (film) ν 2952, 1726, 1686, 1600, 1229, 700 cm^{-1}.

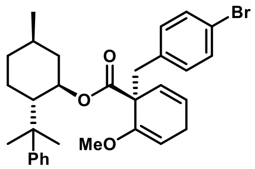

7

Diene (7). Prepared according to **general procedure for Birch reduction/alkylation of ester**, using ester **S6** (158 mg, 0.43 mmol, 1.0 equiv.) and 4-bromobenzyl bromide (324 mg, 1.3 mmol, 3.0 equiv.) to give **7** as a colorless oil (175 mg, 75% yield, 7:1 d.r.).
^{1}H NMR (500 MHz, $CDCl_3$) δ 7.33 – 7.26 (m, 6H), 7.21 – 7.12 (m, J = 4.4 Hz, 1H), 6.93 – 6.86 (m, 2H), 5.77 (dt, J = 9.7, 3.2 Hz, 1H), 5.35 (dt, J = 9.8, 2.0 Hz, 1H), 4.84 (td, J = 10.5, 4.2 Hz, 1H), 4.60 (t, J = 3.6 Hz, 1H), 3.49 (d, J = 4.8 Hz, 1H), 3.47 (s, 3H), 3.31 (d, J = 13.4 Hz, 1H), 2.67 – 2.57 (m, 2H), 2.22 (dq, J = 22.3, 3.0 Hz, 1H), 2.01 – 1.86 (m, 2H), 1.50 (dt, J = 12.8, 3.0 Hz, 1H), 1.47 – 1.36 (m, 1H), 1.31 (s, 3H), 1.26 (s, 3H), 1.01 – 0.89 (m, 2H), 0.85 (d, J = 6.4 Hz, 3H), 0.80 – 0.69 (m, 1H).
^{13}C{^{1}H} NMR (126 MHz, $CDCl_3$) δ 172.6, 151.0, 150.8, 136.9, 132.4, 130.4, 128.1, 127.1, 126.2, 126.0, 125.4, 120.0, 94.8, 76.6, 53.4, 50.4, 41.6, 40.4, 39.6, 34.6, 31.5, 29.7, 27.5, 26.2, 23.8, 21.9.
HRMS (ES+) m/z calc'd for $C_{31}H_{37}BrO_3Na$ $[M + Na]^+$: 559.1824, found 559.1844.
IR (film) ν 2952, 2924, 1724, 1690, 1600, 1228, 701 cm^{-1}.

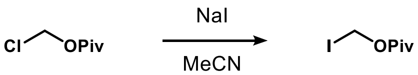

S7

Iodomethyl pivalate (S7). A flame-dried 250 mL round bottom flask equipped with a stir bar was charged with anhydrous MeCN (40 mL), chloromethyl pivalate (9.6 mL, 66.4 mmol, 1.0 equiv.), and NaI (11.9 g, 79.7 mmol, 1.2 equiv.). After stirring for 5 h, the solution was filtered through a pad of Celite® using EtOAc as eluent and concentrated *in vacuo*. The resulting mixture was dissolved in CH_2Cl_2 (500 mL), washed with a saturated aqueous solution of $Na_2S_2O_3$ (100 mL), and water (100 mL). The organic phase was separated, dried over anhydrous $MgSO_4$, filtered, and concentrated *in vacuo* to afford **S7** as a yellow-orange oil (15.4 g, 96% yield). This material was used without further purification. ^{1}H and ^{13}C NMR spectra were consistent with those previously reported.[3]

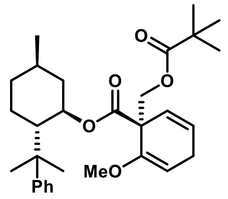

8

Diene (8). Prepared according to **general procedure for Birch reduction/alkylation of ester**, using ester **S6** (92.9 mg, 0.3 mmol, 1.0 equiv.) and iodomethyl pivalate (184 mg, 0.8 mmol, 3.0 equiv.) to give **8** as a colorless oil (92.5 mg, 76% yield, 13:1 d.r.).

^{1}H NMR (500 MHz, $CDCl_3$) δ 7.28 (d, *J* = 6.5 Hz, 4H), 7.15 (tt, *J* = 5.6, 2.2 Hz, 1H), 5.95 (dt, *J* = 9.7, 3.5 Hz, 1H), 5.43 (dt, *J* = 9.8, 2.1 Hz, 1H), 4.86 (t, 1H), 4.82 (dt, *J* = 10.6, 5.3 Hz, 1H), 4.42 (d, *J* = 10.7 Hz, 1H), 4.23 (d, *J* = 10.7 Hz, 1H), 3.48 (s, 3H), 2.85 (qq, *J* = 22.5, 3.2 Hz, 2H), 1.95 – 1.90 (m, 1H), 1.90 – 1.84 (m, 1H), 1.52 – 1.45 (m, 1H), 1.44 – 1.35 (m, 1H), 1.30 (s, 3H), 1.24 (s, 3H), 1.13 (s, 9H), 1.01 – 0.92 (m, 1H), 0.89 (d, *J* = 7.0 Hz, 1H), 0.86 (d, *J* = 6.7 Hz, 1H), 0.83 (d, *J* = 6.5 Hz, 3H), 0.73 (qd, *J* = 12.8, 3.4 Hz, 1H).

^{13}C{^{1}H} NMR (126 MHz, $CDCl_3$) δ 178.1, 170.6, 150.62, 150.59, 128.2, 128.1, 127.6, 126.0, 126.0, 125.5, 124.7, 94.4, 76.6, 64.9, 54.2, 50.4, 41.6, 40.4, 34.6, 31.4, 30.0, 27.5, 27.3, 26.6, 23.6, 21.9.

HRMS (ES+) m/z calc'd for $C_{30}H_{42}O_5H$ $[M + H]^+$: 483.3110; found 483.3108.

IR (film) ν 2958, 1726, 1690, 1265, 1241, 734 cm^{-1}.

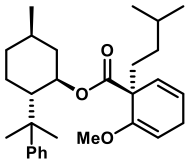

9

Diene (9). Prepared according to **general procedure for Birch reduction/alkylation of ester**, using ester **S6** (100 mg, 0.3 mmol, 1.0 equiv.) and 1-iodo-3-methylbutane (162 mg, 0.8 mmol, 3.0 equiv.) to give **9** as a colorless oil (64.7 mg, 54% yield, 9:1 d.r.).

^{1}H NMR (500 MHz, $CDCl_3$) δ 7.28 (d, *J* = 4.8 Hz, 4H), 7.18 – 7.11 (m, 1H), 5.91 – 5.84 (m, 1H), 5.29 (dd, *J* = 9.9, 2.2 Hz, 1H), 4.84 – 4.73 (m, 2H), 3.47 (s, 3H), 2.93 – 2.75 (m, 2H), 2.05 (td, *J* = 12.9, 4.7 Hz, 1H), 1.91 (d, *J* = 12.5 Hz, 1H), 1.88 – 1.81 (m, 1H), 1.52 – 1.43 (m, 2H), 1.42 – 1.35 (m, 1H), 1.30 (s, 3H), 1.24 (s, 3H), 1.20 (dd, *J* = 13.7, 3.4 Hz, 1H), 1.02 (tt, *J* = 12.2, 5.3 Hz, 1H), 0.98 – 0.87 (m, 4H), 0.87 (d, *J* = 2.2 Hz, 3H), 0.86 (d, *J* = 2.2 Hz, 3H), 0.82 (d, *J* = 6.4 Hz, 3H), 0.72 (qd, 1H).

^{13}C{^{1}H} NMR (126 MHz, $CDCl_3$) δ 173.3, 152.4, 150.8, 128.08, 128.05, 127.7, 126.3, 126.0, 125.4, 93.7, 76.1, 54.0, 52.2, 50.5, 41.6, 40.5, 34.7, 33.4, 32.0, 31.5, 30.5, 28.4, 27.7, 26.7, 23.1, 22.9, 22.8, 21.9.

HRMS (ES+) m/z calc'd for $C_{29}H_{42}O_3Na$ [M + Na]$^+$: 461.3032, found 461.3011.

IR (film) ν 2954, 2924, 2869, 1723, 1685, 1211, 736 cm^{-1}.

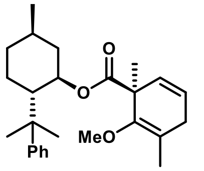

11

Diene (11). Prepared according to **general procedure for Birch reduction/alkylation of ester**, using ester **S5** (738 mg, 1.9 mmol, 1.0 equiv.) and iodomethane (0.40 mL, 5.8 mmol, 3.0 equiv.) to give **11** as a colorless oil (498 mg, 65% yield, 5:1 d.r.).

^{1}H NMR (600 MHz, CDCl3) δ 7.31 – 7.26 (m, 4H), 7.19 – 7.12 (m, 1H), 5.72 (dt, J = 9.8, 3.4 Hz, 1H), 5.39 (dt, J = 9.8, 2.0 Hz, 1H), 4.77 (td, J = 10.6, 4.2 Hz, 1H), 3.65 (s, 3H), 2.77 (t, 1H), 2.69 (d, 1H), 2.02 – 1.85 (m, 2H), 1.69 (s, 3H), 1.50 (dp, J = 12.8, 2.7 Hz, 1H), 1.36 (d, J = 15.1 Hz, 2H), 1.32 (s, 3H), 1.30 (s, 3H), 1.23 (s, 3H), 1.00 – 0.92 (m, 1H), 0.92 – 0.86 (m, 1H), 0.84 (d, J = 6.5 Hz, 3H), 0.75 (qd, 1H).

^{13}C{^{1}H} NMR (151 MHz, $CDCl_3$) δ 173.6, 151.0, 149.9, 129.8, 128.1, 125.9, 125.4, 124.4, 115.9, 76.2, 61.4, 50.5, 49.7, 41.6, 40.4, 34.7, 33.4, 31.4, 29.8, 27.5, 24.1, 23.0, 21.9, 16.2.

HRMS (ES+) m/z calc'd for $C_{26}H_{36}O_3$ [M]$^+$: 396.2664, found 396.2669.

IR (film) ν 2925, 2870, 1721, 1600, 1225, 699 cm^{-1}.

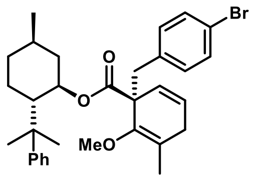

12

Diene (12). Prepared according to **general procedure for Birch reduction/alkylation of ester**, using ester **S5** (130 mg, 0.3 mmol, 1.0 equiv.) and 1-bromo-4-(bromomethyl)benzene (256 mg, 1.0 mmol, 3.0 equiv.) to give **12** as a colorless oil (155 mg, 82% yield, 7:1 d.r.).

^{1}H NMR (600 MHz, $CDCl_3$) δ 7.30 – 7.26 (m, 6H), 7.14 (hept, *J* = 4.2 Hz, 1H), 6.94 (dt, 2H), 5.60 (dt, *J* = 9.8, 3.4 Hz, 1H), 5.32 (dt, *J* = 9.9, 1.9 Hz, 1H), 4.84 (td, *J* = 10.7, 4.2 Hz, 1H), 3.79 (s, 3H), 3.07 (d, *J* = 13.3 Hz, 1H), 2.84 (d, *J* = 13.3 Hz, 1H), 2.52 – 2.43 (m, 1H), 2.10 – 2.03 (m, 1H), 1.98 – 1.90 (m, 2H), 1.58 (s, 3H), 1.53 (dq, *J* = 12.9, 3.1 Hz, 1H), 1.49 – 1.42 (m, 1H), 1.35 (s, 3H), 1.32 (dq, *J* = 13.8, 3.4 Hz, 1H), 1.25 (s, 3H), 1.05 – 0.91 (m, 2H), 0.86 (d, *J* = 6.5 Hz, 3H), 0.77 (qd, *J* = 13.0, 3.5 Hz, 1H).

^{13}C{^{1}H} NMR (151 MHz, $CDCl_3$) δ 173.3, 150.7, 147.0, 136.7, 132.7, 130.2, 128.2, 127.1, 126.8, 125.9, 125.5, 120.0, 116.9, 76.8, 61.5, 55.2, 50.7, 41.8, 40.5, 39.4, 34.7, 33.4, 31.5, 30.1, 27.6, 24.2, 21.9, 16.3.

HRMS (ES+) m/z calc'd for $C_{32}H_{39}BrO_3Na$ $[M + Na]^+$: 573.1980, found 573.1993.

IR (film) ν 2923, 1716, 1599, 1226, 699 cm^{-1}.

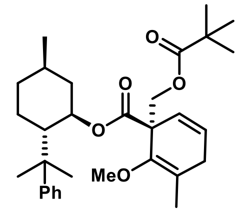

13

Ester (13). Prepared according to **general procedure for Birch reduction/alkylation of ester**, using ester **S5** (110 mg, 0.29 mmol, 1.0 equiv.) and iodomethyl pivolate (477 mg, 1.97 mmol, 3.0 equiv.) to give **13** as a colorless oil (259 mg, 80% yield, 7:1 d.r.).

^{1}H NMR (500 MHz, $CDCl_3$) δ 7.24 (d, *J* = 6.4 Hz, 4H), 7.11 (tt, *J* = 5.5, 2.2 Hz, 1H), 5.91 (dt, *J* = 9.9, 3.6 Hz, 1H), 5.39 (dt, *J* = 9.9, 2.1 Hz, 1H), 4.84 – 4.73 (m, 2H), 4.38 (d, *J* = 10.7 Hz, 1H), 4.18 (d, *J* = 10.7 Hz, 1H), 3.44 (s, 3H), 2.90 – 2.72 (m, 2H), 1.92 – 1.79 (m, 2H), 1.44 (dt, *J* = 12.8, 3.3 Hz, 1H), 1.36 (td, *J* = 13.1, 6.7 Hz, 1H), 1.26 (s, 3H), 1.20 (s, 3H), 1.09 (s, 9H), 0.93 – 0.82 (m, 3H), 0.79 (d, *J* = 6.6 Hz, 3H), 0.68 (qd, *J* = 12.8, 3.4 Hz, 1H).

^{13}C{^{1}H} NMR (126 MHz, $CDCl_3$) δ 178.1, 170.6, 150.62, 150.59, 128.2, 127.6, 126.0, 125.5, 124.7, 94.5, 76.6. 64.9, 54.2, 52.4, 50.4, 41.6, 40.4, 38.9, 34.6, 31.4, 30.0, 27.5, 27.3, 26.6, 23.6, 21.9.

HRMS (ES+) m/z calc'd for $C_{31}H_{44}O_5Na$ $[M + Na]^+$: 519.3087, found 519.3079.

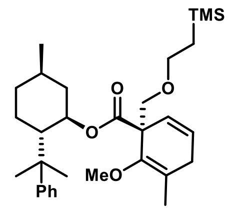

14

Diene (14). Prepared according to **general procedure for Birch reduction/alkylation of ester**, using ester **S5** (110 mg, 0.29 mmol, 1.0 equiv.) and (2-(chloromethoxy)ethyl)trimethylsilane (0.15 mL, 0.87 mmol, 3.0 equiv.) to give **14** as a colorless oil (134 mg, 90% yield, 4:1 d.r.).

^{1}H NMR (500 MHz, $CDCl_3$) δ 7.28 (t, *J* = 4.4 Hz, 4H), 7.15 (hept, *J* = 4.2 Hz, 1H), 5.85 (dt, *J* = 9.8, 3.4 Hz, 1H), 5.49 (ddt, *J* = 9.9, 8.0, 2.0 Hz, 1H), 4.76 (td, *J* = 10.6, 4.2 Hz, 1H), 3.72 (d, *J* = 9.1 Hz, 1H), 3.64 (s, 3H), 3.56 – 3.50 (m, 2H), 2.76 – 2.69 (m, 2H), 1.96 (dtd, *J* = 12.2, 3.8, 2.1 Hz, 1H), 1.90 (ddd, *J* = 12.1, 10.5, 3.4 Hz, 1H), 1.70 (s, 3H), 1.51 (ddp, *J* = 14.5, 6.4, 3.0 Hz, 1H), 1.45 – 1.37 (m, 1H), 1.30 (s, 3H), 1.23 (s, 3H), 1.00 – 0.85 (m, 6H), 0.84 (d, *J* = 6.6 Hz, 3H), 0.75 (qd, *J* = 12.9, 3.5 Hz, 1H), -0.01 (s, 9H).

^{13}C{^{1}H} NMR (151 MHz, $CDCl_3$) δ 171.6, 150.9, 146.7, 128.1, 127.1, 126.3, 125.9, 125.4, 118.3, 76.9, 76.3, 71.7, 68.6, 61.0, 54.7, 50.5, 41.6, 40.4, 34.7, 33.5, 31.4, 29.6, 27.5, 24.2, 21.9, 18.1, 16.4, -1.2.

HRMS (ES+) m/z calc'd for $C_{31}H_{48}O_4SiNa$ $[M + Na]^+$: 535.3220, found 535.3217.

IR (film) ν 2951, 2919, 2869, 1721, 1699, 1600, 1222, 699 cm^{-1}.

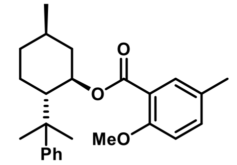

S8

Ester (S8). Prepared according to **general procedure for esterification**, using (–)-8-phenylmenthyl (700 mg, 3.0 mmol, 1.0 equiv.), 2-methoxy-5-methylbenzoic acid (750 mg, 4.5 mmol, 1.5 equiv.), DMAP (74 mg, 0.60 mmol, 0.2 equiv.), and DCC (932 mg, 4.5 mmol, 1.5 equiv.) to give **S8** as a colorless oil (1.1 g, 96% yield).

^{1}H NMR (500 MHz, $CDCl_3$) δ 7.31 – 7.26 (m, 2H), 7.21 – 7.12 (m, 3H), 7.03 – 6.96 (m, 2H), 6.80 (d, *J* = 8.5 Hz, 1H), 5.10 (td, *J* = 10.7, 4.4 Hz, 1H), 3.85 (s, 3H), 2.22 (s, 3H), 2.13 (ddd, *J* = 12.2, 10.5, 3.3 Hz, 1H), 2.02 (dd, *J* = 11.1, 5.4 Hz, 1H), 1.69 – 1.61 (m, 2H), 1.52 (ttd, *J* = 12.0, 6.3, 3.1 Hz, 1H), 1.36 (s, 3H), 1.26 (s, 3H), 1.19 – 1.03 (m, 2H), 0.93 – 0.85 (m, 4H).

^{13}C{^{1}H} NMR (151 MHz, $CDCl_3$) δ 164.9, 157.6, 151.7, 133.9, 132.0, 129.0, 127.9, 125.6, 125.0, 119.7, 111.9, 74.6, 56.2, 50.7, 42.1, 40.0, 34.8, 31.5, 27.2, 27.0, 26.3, 22.0, 20.4.

HRMS (ES+) m/z calc'd for $C_{25}H_{32}O_3$ $[M + Na]^+$: 403.2249, found 403.2235.

IR (film) ν 2952, 2922, 2869, 1718, 1580, 1249 cm^{-1}.

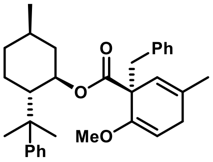

15

Diene (15). Prepared according to **general procedure for Birch reduction/alkylation of ester**, using ester **S8** (82 mg, 0.22 mmol, 1.0 equiv.) and benzyl bromide (0.080 mL, 0.67 mmol, 3.1 equiv.) to give **15** as a colorless oil (86 mg, 87% yield, 8:1 d.r.).

^{1}H NMR (600 MHz, $CDCl_3$) δ 7.34 – 7.28 (m, 4H), 7.19 – 7.13 (m, 4H), 7.02 – 6.98 (m, 2H), 5.11 (p, *J* = 1.4 Hz, 1H), 4.85 (td, *J* = 10.6, 4.2 Hz, 1H), 4.54 (t, *J* = 3.6 Hz, 1H), 3.48 (s, 3H), 3.31 (dd, *J* = 13.4, 4.6 Hz, 1H), 2.70 (d, *J* = 13.2 Hz, 1H), 2.45 (dd, *J* = 21.6, 3.9 Hz, 1H), 1.99 (dd, *J* = 17.5, 4.4 Hz, 2H), 1.93 (ddd, *J* = 13.2, 10.2, 3.2 Hz, 1H), 1.66 (s, 3H), 1.55 – 1.48 (m, 1H), 1.33 (s, 3H), 1.27 (s, 4H), 1.03 – 0.88 (m, 3H), 0.87 (d, *J* = 6.4 Hz, 3H), 0.76 (qd, *J* = 12.7, 3.5 Hz, 1H).

^{13}C{^{1}H} NMR (151 MHz, $CDCl_3$) δ 173.3, 151.4, 151.0, 138.0, 130.7, 128.1, 127.2, 126.6, 126.0, 125.8, 125.4, 121.4, 94.5, 76.4, 54.3, 53.8, 50.4, 41.6, 40.6, 40.4, 34.7, 31.5, 30.9, 29.8, 27.6, 23.7, 22.5, 21.9.

HRMS (ES+) m/z calc'd for $C_{32}H_{40}O_3Na$ $[M + Na]^+$: 495.2875, found 495.2862.

IR (film) ν 2952, 2870, 1721, 1697, 1601, 1225, 698 cm^{-1}.

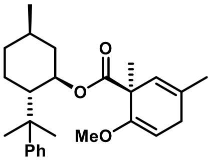

16

Diene (16). Prepared according to **general procedure for Birch reduction/alkylation of ester**, using ester **S8** (74 mg, 0.19 mmol, 1.0 equiv.) and methyl iodide (0.040 mL, 0.58 mmol, 3.0 equiv.) to give **16** as a colorless oil (56 mg, 73% yield, 3:1 d.r.).

^{1}H NMR (500 MHz, $CDCl_3$) δ 7.33 – 7.27 (m, 4H), 7.16 (h, *J* = 4.3 Hz, 1H), 5.14 (q, *J* = 1.6 Hz, 1H), 4.78 (td, *J* = 10.6, 4.3 Hz, 1H), 4.70 (t, *J* = 3.5 Hz, 1H), 3.49 (s, 3H), 2.84 – 2.66 (m, 2H), 1.96 – 1.82 (m, 2H), 1.74 (s, 3H), 1.47 (dt, *J* = 12.8, 3.1 Hz, 1H), 1.33 (s, 3H), 1.31 (s, 3H), 1.25 (s, 3H), 1.23 – 1.19 (m, 1H), 0.96 – 0.85 (m, 3H), 0.83 (d, *J* = 6.6 Hz, 3H), 0.78 – 0.64 (m, 1H).

^{13}C{^{1}H} NMR (126 MHz, $CDCl_3$) δ 173.7, 154.9, 150.8, 132.4, 128.1, 126.0, 125.4, 123.9, 91.8, 76.1, 54.2, 50.5, 48.8, 41.6, 40.5, 34.7, 31.5, 31.4, 30.4, 27.6, 23.5, 23.1, 22.6, 21.9.

HRMS (ES+) m/z calc'd for $C_{26}H_{36}O_3Na$ $[M + Na]^+$: 419.2562, found 419.2556.

IR (film) ν 2952, 1725, 1695, 1665, 1227, 701 cm^{-1}.

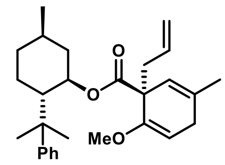

17

Diene (17). Prepared according to **general procedure for Birch reduction/alkylation of ester**, using ester **S8** (100 mg, 0.26 mmol, 1.0 equiv.) and allyl bromide (0.070 mL, 0.79 mmol, 3.0 equiv.) to give **17** as a colorless oil (83 mg, 75% yield, 5:1 d.r.).

^{1}H NMR (500 MHz, C_6D_6) δ 7.26 – 7.18 (m, 4H), 7.08 (tt, *J* = 6.7, 1.8 Hz, 1H), 5.88 (ddt, *J* = 17.3, 10.2, 7.2 Hz, 1H), 5.23 (dd, *J* = 1.6, 1.5 Hz, 1H), 5.16 (dq, *J* = 17.1, 1.6 Hz, 1H), 5.07 (dt, 1H), 4.98 (td, *J* = 10.5, 4.3 Hz, 1H), 4.63 (t, *J* = 3.5 Hz, 1H), 3.28 (s, 3H), 3.19 (dd, *J* = 14.1, 7.5 Hz, 1H), 2.89 (dd, *J* = 14.0, 6.9 Hz, 1H), 2.60 (qd, 2H), 2.19 (dq, *J* = 12.1, 3.2 Hz, 1H), 1.80 (ddd, *J* = 12.1, 10.5, 3.3 Hz, 1H), 1.58 (s, 3H), 1.41 (s, 3H), 1.31 (s, 3H), 1.22 (ddt, *J* = 12.8, 6.5, 3.4 Hz, 2H), 1.10 (dddt, *J* = 12.5, 9.5, 6.5, 3.1 Hz, 1H), 0.98 (td, *J* = 12.2, 10.5 Hz, 1H), 0.81 – 0.71 (m, 1H), 0.70 (d, *J* = 6.4 Hz, 3H), 0.60 – 0.48 (m, 1H).

^{13}C{^{1}H} NMR (126 MHz, $CDCl_3$) δ 172.3, 153.9, 150.6, 135.2, 134.6, 126.3, 125.7, 122.4, 117.1, 93.5, 76.0, 54.1, 53.2, 51.2, 42.0, 40.8, 39.8, 34.8, 31.8, 31.4, 31.3, 27.9, 23.2, 22.4, 22.0.

HRMS (ES+) m/z calc'd for $C_{28}H_{38}O_3$ $[M]^+$:422.2821, found 422.2814.

IR (film) ν 2952, 2923, 2871, 1725, 1697, 1600, 1226, 701 cm^{-1}.

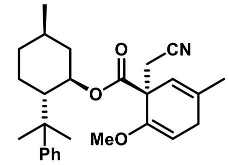

18

Diene (18). Liquid NH_3 (4 mL) was distilled into a flame-dried 25 mL three-neck round bottom flask at –78 °C equipped with a glass-encased stir bar and condenser. In a separate flame-dried 10 mL pear-shaped flask, a solution of ester **S8** (89 mg, 0.2 mmol, 1.0 equiv.) in anhydrous THF (2.5 mL) was prepared. Lithium metal (4.9 mg, 0.7 mmol, 3.0 equiv.) was added to the stirred solution in two pieces, which resulted in a deep blue solution after 10 min. After 30 min, the solution was allowed to warm up slowly to 0 °C for 30 min. The reaction was concentrated under an argon stream; then, it was cooled back down to –78 °C, followed by the addition of bromoacetonitrile (0.1 mL, 1.2 mmol, 5.0 equiv.) dropwise. The reaction mixture was stirred for an additional 2 h at –78 °C. Once the reaction was complete, the reaction mixture was quenched with NH_4Cl (37.5 mg, 0.7 mmol, 3.0 equiv.). Brine (10 mL) was added, and the mixture was extracted with hexanes (3 x 10 mL). The combined extracts were dried over anhydrous $MgSO_4$, filtered, and concentrated under reduced pressure to afford a yellow oil, which was purified by flash column chromatography (SiO_2, 2% EtOAc in hexanes) to give diene **18** as a colorless oil (69.0 mg, 70% yield, >95% pure, 4:1 d.r.).

^{1}H NMR (500 MHz, C_6D_6) δ 7.21 – 7.18 (m, 4H), 7.12 – 7.04 (m, 1H), 5.09 (s, 1H), 4.89 (td, *J* = 10.6, 4.2 Hz, 1H), 4.59 (t, 1H), 3.12 (s, 3H), 2.94 (d, *J* = 16.7 Hz, 1H), 2.70 – 2.47 (m, 2H), 2.35

(d, *J* = 16.7 Hz, 1H), 2.03 (d, *J* = 12.2 Hz, 1H), 1.76 (td, *J* = 11.9, 3.5 Hz, 1H), 1.55 (s, 3H), 1.27 (s, 3H), 1.16 (s, 3H), 1.12 (td, *J* = 7.0, 1.7 Hz, 1H), 1.09 – 1.01 (m, 1H), 0.91 (q, 1H), 0.71 (qd, *J* = 14.0, 4.5 Hz, 1H), 0.68 (d, *J* = 6.4 Hz, 3H), 0.56 – 0.47 (m, 1H), 0.48 – 0.40 (m, 1H).
^{13}C{^{1}H} NMR (126 MHz, $CDCl_3$) δ 170.8, 150.9, 150.2, 137.3, 128.2, 125.8, 125.5, 118.8, 117.8, 95.2, 77.3, 54.5, 50.2, 41.3, 40.1, 34.5, 31.4, 31.3, 31.3, 28.3, 27.2, 25.1, 25.0, 22.7, 21.8.
HRMS (ES+) m/z calc'd for $C_{27}H_{35}NO_3Na$ $[M + Na]^+$: 444.2515, found 444.2502.
IR (film) ν 2953, 2918, 1724, 1698, 1600, 1244, 701 cm^{-1}.

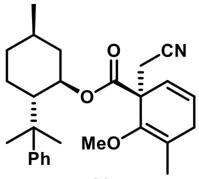

19

Diene (19). Prepared according to **general procedure for Birch reduction/alkylation of ester**, using ester **S5** (100 mg, 0.26 mmol, 1.0 equiv.) and 2-bromoacetonitrile (0.60 mL, 0.79 mmol, 3.0 equiv.) to give **19** as a colorless oil (68 mg, 61% yield, 8:1 d.r.).
^{1}H NMR (500 MHz, $CDCl_3$) δ 7.35 – 7.27 (m, 4H), 7.19 (tt, *J* = 5.6, 2.2 Hz, 1H), 5.98 (dt, *J* = 9.8, 3.4 Hz, 1H), 5.30 (dt, *J* = 9.8, 2.0 Hz, 1H), 4.76 (td, *J* = 10.7, 4.3 Hz, 1H), 3.66 (s, 3H), 2.81 (dt, *J* = 11.6, 2.6 Hz, 2H), 2.69 (d, *J* = 16.8 Hz, 1H), 2.05 (ddd, *J* = 12.2, 10.5, 3.4 Hz, 1H), 1.96 – 1.87 (m, 2H), 1.74 (s, 3H), 1.65 – 1.55 (m, 2H), 1.52 – 1.40 (m, 1H), 1.28 (s, 3H), 1.18 (s, 3H), 1.09 – 0.94 (m, 2H), 0.90 – 0.77 (m, 4H).
^{13}C{^{1}H} NMR (151 MHz, $CDCl_3$) δ 170.6, 151.6, 145.9, 128.9, 128.4, 125.6, 125.6, 124.7, 119.0, 117.9, 66.0, 61.7, 51.6, 50.0, 41.4, 40.0, 34.6, 33.5, 31.4, 27.2, 27.0, 26.4, 24.2, 21.9, 16.4.
HRMS (ES+) m/z calc'd for $C_{27}H_{35}O_3Na$ $[M + Na]^+$: 444.2515, found 444.2518.

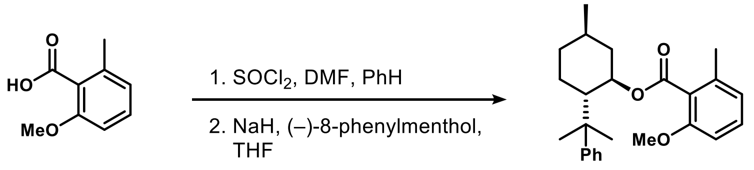

S9 **S10**

Ester (S10). This ester could not be made via the general procedure described above using DCC. A flame-dried 50 mL round bottom flask equipped with a stir bar was charged with 2-methoxy-6-methylbenzoic acid **S9** (698 mg, 4.2 mmol, 1.2 equiv.) and benzene (20 mL) under argon atmosphere. $SOCl_2$ (0.38 mL, 5.2 mmol, 1.5 equiv.) and DMF (0.27 mL, 3.4 mmol, 1.0 equiv.) were added. The reaction was heated to 70 °C for 5 h and judged complete by ^{1}H NMR of aliquots. After cooling to room temperature, the reaction mixture was diluted with benzene (10 mL) and concentrated *in vacuo*, repeating two additional times. The resulting yellow oil was dissolved in anhydrous THF (7 mL) and added dropwise to a stirred solution of (–)-8-phenylmenthol (800 mg, 3.4 mmol, 1.0 equiv.) and NaH (60 wt%, 180 mg, 4.5 mmol, 1.3 equiv.) in THF (7 mL) at 0 °C. After stirring overnight, the reaction mixture was concentrated *in vacuo*, diluted with CH_2Cl_2 (20 mL), washed with saturated NH_4Cl (20 mL) and brine (20 mL). The combined organic extracts

were dried over Na_2SO_4, filtered, and concentrated *in vacuo*. The resulting yellow oil was purified by column chromatography (SiO_2, 5% EtOAc in hexanes) to afford the product **S10** as a colorless oil (1.2 g, 90% yield).
^{1}H NMR (499 MHz, $CDCl_3$) δ 7.27 (d, J = 8.2 Hz, 2H), 7.21 (t, J = 7.7 Hz, 3H), 7.08 (t, J = 7.2 Hz, 1H), 6.76 (dd, J = 17.2, 8.0 Hz, 2H), 5.05 (td, J = 10.6, 4.2 Hz, 1H), 3.79 (s, 3H), 2.30 (s, 3H), 1.97 – 1.88 (m, 1H), 1.54 – 1.49 (m, 2H), 1.37 (s, 3H), 1.36 (s, 3H), 1.28 – 1.23 (m, 1H), 1.12 (q, J = 11.6 Hz, 1H), 1.00 – 0.92 (m, 1H), 0.90 (d, J = 6.3 Hz, 4H), 0.77 (qd, J = 13.1, 3.4 Hz, 1H).
^{13}C{^{1}H} NMR (151 MHz, $CDCl_3$) δ 168.1, 156.2, 150.9, 136.1, 130.0, 127.9, 125.9, 125.3, 124.7, 122.5, 108.5, 76.7, 55.6, 50.9, 41.7, 40.6, 34.7, 31.6, 30.1, 27.7, 23.0, 22.0, 19.3.
HRMS (ES+) m/z calc'd for $C_{25}H_{32}O_3H$ $[M + H]^+$: 381.2430, found 381.2411.
IR (film) ν 2953, 1716, 1584, 1471, 1263 cm^{-1}.

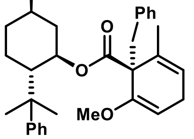

20

Diene (20). Prepared according to **general procedure for Birch reduction/alkylation of ester**, using ester **S10** (100 mg, 0.26 mmol, 1.0 equiv.) and benzyl bromide (0.10 mL, 0.79 mmol, 3.0 equiv.) to give **20** as a colorless oil (109 mg, 87% yield, 16:1 d.r.). Crystals of suitable quality for X-ray diffraction were formed in DCM (50 mg/mL) after vapor diffusion of pentane at room temperature.
^{1}H NMR (600 MHz, $CDCl_3$) δ 7.31 – 7.26 (m, 4H), 7.19 – 7.13 (m, 4H), 7.04 – 6.98 (m, 2H), 5.53 (d, J = 1.3 Hz, 1H), 4.83 (td, J = 10.6, 4.0 Hz, 1H), 4.57 (t, J = 3.6 Hz, 1H), 3.47 (s, 3H), 3.42 (d, J = 13.7 Hz, 1H), 3.04 (d, J = 13.7 Hz, 1H), 2.49 (dtd, J = 22.0, 4.2, 1.9 Hz, 1H), 2.18 – 2.11 (m, 1H), 1.98 (dq, J = 21.9, 2.6 Hz, 1H), 1.85 (ddd, J = 12.2, 10.5, 3.4 Hz, 1H), 1.81 (q, J = 1.7 Hz, 3H), 1.50 – 1.41 (m, 2H), 1.31 (s, 3H), 1.26 (s, 3H), 1.11 (dq, J = 13.6, 3.3 Hz, 1H), 0.99 (q, J = 11.6 Hz, 1H), 0.85 (d, J = 6.3 Hz, 3H), 0.82 (dd, J = 13.5, 10.5 Hz, 1H), 0.71 (qd, J = 13.0, 3.3 Hz, 1H).
^{13}C{^{1}H} NMR (151 MHz, $CDCl_3$) δ 172.8, 150.8, 150.7, 137.9, 130.3, 129.7, 128.1, 127.3, 126.1, 126.0, 125.4, 124.2, 94.5, 57.0, 53.7, 50.6, 41.2, 40.5, 37.1, 34.7, 31.5, 31.1, 28.0, 25.9, 22.0, 21.8, 19.3.
HRMS (ES+) m/z calc'd for $C_{32}H_{40}O_3Na$ $[M + Na]^+$: 495.2875, found 495.2864.
IR (film) ν 2920, 2872, 2853, 1727, 1697, 1602, 1200, 702 cm^{-1}.

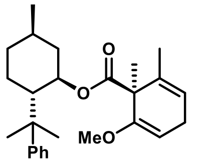

21

Diene (21). Prepared according to **general procedure for Birch reduction/alkylation**, using ester **S10** (100 mg, 0.26 mmol, 1.0 equiv.) and iodomethane (0.050 mL, 0.79 mmol, 3.0 equiv.) to give **21** as a colorless oil (90 mg, 86% yield, 7:1 d.r.).

^{1}H NMR (500 MHz, C_6D_6) δ 7.25 – 7.17 (m, 4H), 7.07 (td, *J* = 6.8, 1.7 Hz, 1H), 5.45 (s, 1H), 4.98 (td, *J* = 10.6, 4.1 Hz, 1H), 4.47 (t, *J* = 3.6 Hz, 1H), 3.18 (s, 2H), 2.72 (qtd, *J* = 22.0, 3.6, 1.9 Hz, 2H), 2.31 – 2.22 (m, 1H), 1.84 – 1.71 (m, 7H), 1.40 (s, 3H), 1.30 (s, 3H), 1.26 – 1.07 (m, 3H), 1.03 – 0.93 (m, 1H), 0.93 – 0.82 (m, 1H), 0.70 (d, *J* = 6.5 Hz, 3H), 0.68 – 0.63 (m, 1H), 0.51 (dtd, *J* = 24.6, 12.8, 3.3 Hz, 1H).

^{13}C{^{1}H} NMR (151 MHz, C_6D_6) δ 172.3, 154.9, 150.7, 133.9, 128.4, 126.4, 125.7, 121.0, 91.4, 76.7, 53.8, 51.6, 50.8, 41.6, 40.7, 34.7, 31.5, 31.4, 28.1, 26.6, 22.2, 22.1, 22.0, 19.2.

HRMS (ES+) m/z calc'd for $C_{26}H_{36}O_3Na$ $[M + Na]^+$: 419.2562, found 419.2579.

IR (film) ν 2950, 2869, 1727, 1697, 1600, 1227, 700 cm^{-1}.

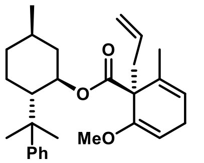

22

Diene (22). Prepared according to **general procedure for Birch reduction/alkylation**, using ester **S10** (99 mg, 0.26 mmol, 1.0 equiv.) and allyl bromide (0.070 mL, 0.78 mmol, 3.0 equiv.) to give **22** as a colorless oil (88 mg, 80% yield, 19:1 d.r.).

^{1}H NMR (500 MHz, $CDCl_3$) δ 7.31 – 7.26 (m, 4H), 7.16 (tt, *J* = 7.0, 2.0 Hz, 1H), 5.66 (s, 1H), 5.55 (ddt, *J* = 17.3, 10.2, 7.2 Hz, 1H), 5.03 (ddt, *J* = 17.1, 2.6, 1.5 Hz, 1H), 4.96 (dt, 1H), 4.81 (t, *J* = 3.7 Hz, 1H), 4.77 (td, *J* = 10.7, 4.2 Hz, 1H), 3.46 (s, 3H), 2.88 (ddt, *J* = 14.5, 6.8, 1.4 Hz, 1H), 2.85 – 2.71 (m, 2H), 2.55 (dd, *J* = 14.5, 7.5 Hz, 1H), 2.04 (dq, 1H), 1.82 (ddd, *J* = 12.1, 10.5, 3.4 Hz, 1H), 1.64 (d, *J* = 1.8 Hz, 3H), 1.47 – 1.39 (m, 2H), 1.30 (s, 3H), 1.24 (s, 3H), 1.11 (dq, *J* = 13.6, 3.3 Hz, 1H), 1.00 – 0.90 (m, 1H), 0.92 – 0.86 (m, 1H), 0.83 (d, *J* = 6.3 Hz, 3H), 0.83 – 0.76 (m, 1H), 0.69 (qd, *J* = 13.0, 3.1 Hz, 1H).

^{13}C{^{1}H} NMR (151 MHz, $CDCl_3$) δ 172.6, 151.3, 150.7, 134.2, 130.5, 128.1, 126.1, 125.4, 123.0, 116.7, 93.7, 76.9, 66.0, 55.3, 54.0, 50.6, 41.1, 40.5, 35.7, 34.7, 31.4, 31.1, 27.9, 26.3, 22.0, 21.9, 19.0, 15.4.

HRMS (ES+) m/z calc'd for $C_{28}H_{38}O_3Na$ $[M + Na]^+$: 445.2719, found 445.2736.

IR (film) ν 2951, 1726, 1697, 1600, 1225, 700 cm^{-1}.

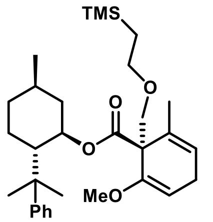

23

Diene (23). Prepared according to **general procedure for Birch reduction/alkylation**, using ester **S10** (100 mg, 0.26 mmol, 1.0 equiv.) and (2-(chloromethoxy)ethyl)trimethylsilane (0.14 mL, 0.79 mmol, 3.0 equiv.) to give **23** as a colorless oil (111 mg, 82% yield, >20:1 d.r.).

^{1}H NMR (600 MHz, CDCl3) δ 7.30 – 7.26 (m, 4H), 7.15 (ddt, J = 7.2, 6.3, 1.6 Hz, 1H), 5.72 (s, 1H), 4.85 (t, J = 3.6 Hz, 1H), 4.75 (td, J = 10.6, 4.2 Hz, 1H), 3.94 (d, J = 9.9 Hz, 1H), 3.76 (d, J = 9.9 Hz, 1H), 3.53 (ddt, J = 11.2, 7.1, 2.4 Hz, 2H), 3.48 (s, 3H), 2.82 (dh, J = 5.4, 1.8 Hz, 2H), 1.99 (dq, J = 12.2, 3.6 Hz, 1H), 1.81 (ddd, J = 12.1, 10.5, 3.4 Hz, 1H), 1.70 (d, J = 1.6 Hz, 3H), 1.42 (ddq, J = 17.8, 10.0, 3.4 Hz, 2H), 1.29 (s, 3H), 1.23 (s, 3H), 1.13 (dq, J = 13.6, 3.4 Hz, 1H), 0.98 – 0.91 (m, 1H), 0.91 – 0.85 (m, 3H), 0.82 (d, J = 6.4 Hz, 3H), 0.81 – 0.77 (m, 1H), 0.69 (qd, 1H), -0.03 (s, 9H).

^{13}C{^{1}H} NMR (151 MHz, $CDCl_3$) δ 171.5, 150.64, 150.60, 129.9, 128.1, 126.1, 125.4, 123.7, 94.4, 76.6, 68.9, 68.4, 56.1, 54.0, 50.5, 41.1, 40.5, 34.6, 31.4, 31.0, 27.8, 26.4, 22.2, 21.9, 19.1, 17.9, -1.3.

HRMS (ES+) m/z calc'd for $C_{31}H_{48}O_4SiNa$ $[M + Na]^+$: 535.3220, found 535.3217.

IR (film) ν 2950, 1721, 1699, 1600, 1230, 700 cm^{-1}.

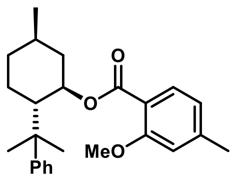

S11

Diene (S11). Prepared according to **general procedure for esterification**, using (–)-8-phenylmenthol (744 mg, 3.2 mmol, 1.0 equiv.), 2-methoxy-4-methylbenzoic acid (692 mg, 4.2 mmol, 1.3 equiv.), DMAP (78 mg, 0.6 mmol, 0.2 equiv.), and DCC (991 mg, 4.8 mmol, 1.5 equiv.) to give **S11** as a colorless oil (816 mg, 67% yield).

^{1}H NMR (500 MHz, $CDCl_3$) δ 7.31 – 7.25 (m, 2H), 7.18 (t, *J* = 7.8 Hz, 2H), 7.11 – 7.01 (m, 2H), 6.71 (s, 1H), 6.60 (d, 1H), 5.07 (td, *J* = 10.7, 4.3 Hz, 1H), 3.88 (s, 3H), 2.35 (s, 3H), 2.11 (ddd, *J* = 12.0, 10.5, 3.4 Hz, 1H), 2.04 (dtd, *J* = 12.2, 4.0, 2.2 Hz, 1H), 1.66 – 1.45 (m, 3H), 1.35 (s, 3H), 1.25 (s, 3H), 1.17 – 1.00 (m, 2H), 0.92 – 0.80 (m, 4H).

^{13}C{^{1}H} NMR (126 MHz, $CDCl_3$) δ 164.68, 159.90, 151.72, 144.39, 132.15, 128.10, 125.60, 125.08, 120.70, 117.06, 112.61, 77.41, 76.91, 74.51, 55.97, 50.69, 42.07, 40.10, 34.81, 31.53, 27.12, 26.94, 26.70, 22.07, 21.96.

HRMS (ES+) m/z calc'd for $C_{25}H_{32}O_3H$ $[M + H]^+$: 381.2430, found 381.2425.

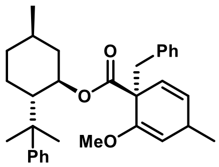

24

Diene (24). Prepared according to **general procedure for Birch reduction/alkylation**, using ester **S11** (110 mg, 0.29 mmol, 1.0 equiv.) and benzyl bromide (0.14 mL, 1.6 mmol, 3.0 equiv.) to give **24** as a colorless oil (108 mg, 79% yield, 1.8:1.1:1).
HRMS (ES+) m/z calc'd for $C_{32}H_{40}O_3Na$ $[M + Na]^+$: 495.2875, found 495.2862.

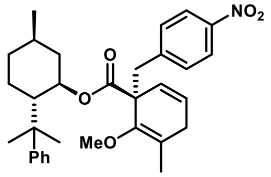

25

Diene (25). Prepared according to **general procedure for Birch reduction/alkylation**, using ester **S5** (144 mg, 0.4 mmol, 1.0 equiv.) and 1-(bromomethyl)-4-nitrobenzene (245 mg, 1.1 mmol, 3.0 equiv.) to give **25** as a colorless oil (41.7 mg, 21% yield, 6:1 d.r.).
^{1}H NMR (500 MHz, $CDCl_3$) δ 8.02 – 7.95 (m, 2H), 7.38 – 7.30 (m, 4H), 7.19 (tt, *J* = 6.7, 1.7 Hz, 1H), 7.16 – 7.10 (m, 2H), 5.60 (dt, *J* = 9.9, 3.4 Hz, 1H), 5.19 (dt, *J* = 9.9, 1.9 Hz, 1H), 4.92 (td, *J* = 10.7, 4.3 Hz, 1H), 3.69 (s, 3H), 3.16 (d, *J* = 13.0 Hz, 1H), 2.48 (dt, *J* = 22.3, 2.8 Hz, 1H), 2.31 (d, *J* = 12.9 Hz, 1H), 2.22 – 2.09 (m, 1H), 2.00 (d, *J* = 12.1 Hz, 1H), 1.85 – 1.77 (m, 1H), 1.67 – 1.60 (m, 1H), 1.55 – 1.52 (m, 1H), 1.51 (s, 3H), 1.36 (s, 3H), 1.21 (s, 3H), 1.13 – 1.01 (m, 2H), 0.89 (dd, *J* = 13.4, 6.6 Hz, 5H).
^{13}C{^{1}H} NMR (126 MHz, $CDCl_3$) δ 172.8, 151.8, 146.6, 146.5, 146.1, 131.7, 128.3, 126.8, 126.1, 125.7, 125.4, 122.2, 118.0, 76.4, 61.3, 54.8, 49.8, 41.9, 40.1, 39.3, 34.7, 33.0, 31.5, 27.3, 27.1, 26.7, 22.0, 16.0.
HRMS (ES+) m/z calc'd for $C_{32}H_{39}NO_5Na$ $[M + Na]^+$: 540.2726, found 540.2732.
IR (film) ν 2924, 2869, 1716, 1604, 1227, 699 cm^{-1}.

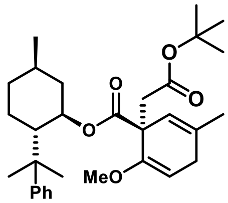

26

Diene (26). Prepared according to **general procedure for Birch reduction/alkylation of ester**, using ester **S8** (100 mg, 0.26 mmol, 1.0 equiv.) and tert-butyl 2-bromoacetate (0.12 mL, 0.79 mmol, 3.0 equiv.) to give **26** as a colorless oil (17 mg, 13% yield, 4:1 d.r.).

^{1}H NMR (500 MHz, $CDCl_3$) δ 7.31 – 7.27 (m, 4H), 7.18 – 7.13 (m, 1H), 5.30 (q, *J* = 1.6 Hz, 1H), 4.79 (t, *J* = 3.5 Hz, 1H), 4.75 (td, *J* = 10.6, 4.3 Hz, 1H), 3.50 (s, 3H), 2.82 (d, *J* = 13.9 Hz, 1H), 2.79 – 2.64 (m, 2H), 2.59 (d, *J* = 13.9 Hz, 1H), 1.96 – 1.88 (m, 1H), 1.84 (ddd, *J* = 12.2, 10.5, 3.5 Hz, 1H), 1.76 (s, 3H), 1.44 (s, 3H), 1.38 (s, 9H), 1.29 (d, *J* = 8.3 Hz, 3H), 1.23 (s, 3H), 0.97 – 0.84 (m, 2H), 0.83 (d, *J* = 6.5 Hz, 3H), 0.71 (qd, *J* = 12.7, 3.4 Hz, 1H).

^{13}C{^{1}H} NMR (151 MHz, $CDCl_3$) δ 172.0, 170.4, 151.8, 150.7, 133.8, 128.1, 126.0, 125.5, 121.2, 93.7, 79.8, 76.7, 54.2, 50.5, 41.5, 41.4, 40.4, 34.7, 31.4, 31.3, 30.2, 28.2, 28.1, 27.6, 23.2, 22.7, 21.9.

IR (film) ν 2954, 2925, 1724, 1207, 700 cm^{-1}.

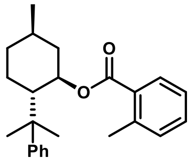

29

Ester (29). Prepared according to **general procedure for esterification**, using (–)-8-phenylmenthyl (538 mg, 2.3 mmol, 1.0 equiv.), 2-methylbenzoic acid (631 mg, 4.6 mmol, 2.0 equiv.), DMAP (57 mg, 0.46 mmol, 0.2 equiv.), and DCC (717 mg, 3.5 mmol, 1.5 equiv.) to give **29** as a colorless oil (778.2 mg, 96% yield).

^{1}H NMR (600 MHz, $CDCl_3$) δ 7.31 (ddd, *J* = 7.3, 5.8, 1.7 Hz, 2H), 7.26 – 7.23 (m, 2H), 7.18 – 7.10 (m, 3H), 7.08 (td, *J* = 7.6, 1.3 Hz, 1H), 6.98 (tt, *J* = 7.3, 1.2 Hz, 1H), 5.06 (td, *J* = 10.7, 4.4 Hz, 1H), 2.53 (s, 3H), 2.15 (ddd, *J* = 12.3, 10.5, 3.5 Hz, 1H), 2.03 (dtd, *J* = 12.3, 3.9, 2.1 Hz, 1H), 1.71 – 1.63 (m, 2H), 1.60 – 1.49 (m, 1H), 1.34 (s, 3H), 1.26 (s, 3H), 1.20 – 1.06 (m, 2H), 0.96 – 0.85 (m, 4H).

^{13}C{^{1}H} NMR (151 MHz, $CDCl_3$) δ 166.7, 151.5, 140.3, 131.7, 131.5, 130.8, 129.9, 128.0, 125.51, 125.46, 125.1, 74.8, 50.8, 42.1, 40.0, 34.8, 31.6, 27.4, 27.0, 26.3, 22.03, 21.97.

HRMS (ES+) m/z calc'd for $C_{24}H_{30}O_2$ $[M]^+$: 350.2246, found 350.2247.

IR (film) ν 2954, 2926, 1710, 1602, 1256 cm^{-1}.

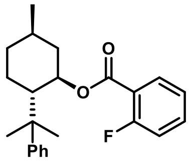

30

Ester (30). Prepared according to **general procedure for esterification**, using (–)-8-phenylmenthyl (200 mg, 0.9 mmol, 1.0 equiv.), 2-fluorobenzoic acid (241 mg, 1.7 mmol, 2.0 equiv.), DMAP (21 mg, 0.17 mmol, 0.2 equiv.), and DCC (266 mg, 1.3 mmol, 1.5 equiv.) to give **30** as a colorless oil (252 mg, 83% yield).

^{1}HNMR (600 MHz, $CDCl_3$) δ 7.42 (dddd, *J* = 8.3, 7.3, 4.8, 1.9 Hz, 1H), 7.33 (td, *J* = 7.7, 1.9 Hz, 1H), 7.27 (t, *J* = 1.4 Hz, 2H), 7.15 – 7.10 (m, 2H), 7.05 – 6.99 (m, 2H), 6.94 (tt, *J* = 7.3, 1.2 Hz, 1H), 5.12 (td, *J* = 10.7, 4.5 Hz, 1H), 2.17 (ddd, *J* = 12.3, 10.5, 3.6 Hz, 1H), 2.03 (dddd, *J* = 12.2, 4.4, 3.5, 2.2 Hz, 1H), 1.73 (dq, *J* = 13.5, 3.5 Hz, 1H), 1.68 (dtd, *J* = 13.2, 3.5, 2.3 Hz, 1H), 1.59 – 1.49 (m, 1H), 1.35 (s, 3H), 1.26 (s, 3H), 1.21 – 1.08 (m, 2H), 0.97 – 0.91 (m, 1H), 0.90 (d, *J* = 6.6 Hz, 3H).

^{13}C{^{1}H} NMR (151 MHz, $CDCl_3$) δ 151.5, 134.1, 134.0, 132.2, 128.0, 125.4, 125.0, 123.6, 123.5, 119.1, 116.8, 116.6, 75.1, 50.6, 41.9, 39.9, 34.7, 31.5, 27.8, 26.8, 25.6, 21.9, 14.2.

HRMS (ES+) m/z calc'd for $C_{23}H_{27}FO_2$ $[M]^+$: 354.1995, found 354.1987.

IR (film) ν 2954, 2922, 1720, 1702, 1612, 1297 cm^{-1}.

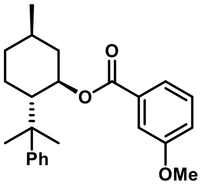

31

Ester (31). Prepared according to **general procedure for esterification**, using (–)-8-phenylmenthyl (480 mg, 2.1 mmol, 1.0 equiv.), 3-methoxybenzoic acid (629 mg, 4.1 mmol, 2.0 equiv.), DMAP (51 mg, 0.41 mmol, 0.2 equiv.), and DCC (639 mg, 3.1 mmol, 1.5 equiv.) to give **31** as a colorless oil (626.9 mg, 83% yield).

^{1}H NMR (500 MHz, $CDCl_3$) δ 7.25 (d, *J* = 5.2 Hz, 3H), 7.22 – 7.13 (m, 4H), 7.04 – 6.98 (m, 2H), 5.09 (td, *J* = 10.7, 4.4 Hz, 1H), 3.82 (s, 3H), 2.16 (ddd, *J* = 12.1, 10.5, 3.3 Hz, 1H), 2.04 – 1.96 (m, 1H), 1.71 – 1.62 (m, 2H), 1.54 (s, 1H), 1.35 (s, 3H), 1.25 (s, 3H), 1.19 – 1.04 (m, 2H), 0.96 – 0.90 (m, 1H), 0.88 (d, *J* = 6.6 Hz, 3H).

^{13}C{^{1}H} NMR (151 MHz, $CDCl_3$) δ 165.7, 159.4, 151.5, 132.0, 129.1, 128.1, 125.5, 125.2, 122.2, 119.2, 114.1, 75.3, 55.5, 50.8, 42.0, 40.0, 34.8, 31.5, 27.2, 27.0, 26.4, 21.9.

HRMS (ES+) m/z calc'd for $C_{24}H_{30}O_3Na$ $[M + Na]^+$: 389.2093, found 389.2094.

IR (film) ν 2956, 1705, 1587, 1275, 730 cm^{-1}.

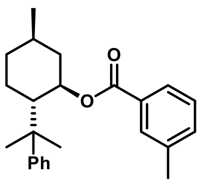

32

Ester (32). Prepared according to **general procedure for esterification**, using (–)-8-phenylmenthyl (500 mg, 2.2 mmol, 1.0 equiv.), 3-methylbenzoic acid (586 mg, 4.3 mmol, 2.0 equiv.), DMAP (53 mg, 0.43 mmol, 0.2 equiv.), and DCC (666 mg, 3.2 mmol, 1.5 equiv.) to give **32** as a white solid (651 mg, 86% yield).

^{1}H NMR (600 MHz, $CDCl_3$) δ 7.42 (dd, *J* = 7.8, 1.7 Hz, 1H), 7.37 – 7.34 (m, 1H), 7.27 (td, *J* = 4.6, 1.6 Hz, 3H), 7.18 (t, *J* = 7.6 Hz, 1H), 7.17 – 7.13 (m, 2H), 7.02 (ddt, *J* = 8.4, 7.1, 1.2 Hz, 1H), 5.08 (td, *J* = 10.7, 4.4 Hz, 1H), 2.33 (s, 3H), 2.19 (ddd, *J* = 12.3, 10.5, 3.5 Hz, 1H), 1.99 (dtd, *J* = 12.3, 4.0, 2.2 Hz, 1H), 1.72 (dq, *J* = 13.6, 3.5 Hz, 1H), 1.70 – 1.64 (m, 1H), 1.60 – 1.50 (m, 1H), 1.34 (s, 3H), 1.24 (s, 3H), 1.16 (qd, *J* = 13.1, 3.4 Hz, 1H), 1.07 (td, *J* = 12.2, 10.8 Hz, 1H), 0.97 – 0.90 (m, 1H), 0.88 (d, *J* = 6.6 Hz, 3H).

^{13}C{^{1}H} NMR (151 MHz, $CDCl_3$) δ 166.0, 151.8, 137.7, 133.4, 130.6, 130.2, 128.1, 128.0, 126.9, 125.5, 125.1, 75.1, 50.7, 41.9, 39.9, 34.8, 31.5, 27.9, 26.9, 25.7, 22.0, 21.4.

HRMS (ES+) m/z calc'd for $C_{24}H_{30}O_2$ $[M]^+$: 350.2246, found 350.2253.

IR (film) ν 2955, 2923, 1699, 1590, 1277 cm^{-1}.

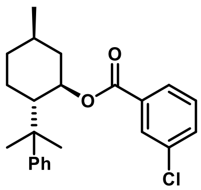

33

Ester (33). Prepared according to **general procedure for esterification**, using (–)-8-phenylmenthyl (494 mg, 2.1 mmol, 1.0 equiv.), 3-chlorobenzoic acid (666 mg, 4.3 mmol, 2.0 equiv.), DMAP (52 mg, 0.43 mmol, 0.2 equiv.), and DCC (658 mg, 3.2 mmol, 1.5 equiv.) to give **33** as a white solid (471.0 mg, 60% yield).

^{1}H NMR (600 MHz, $CDCl_3$) δ 7.44 (dt, *J* = 7.8, 1.4 Hz, 1H), 7.41 (ddd, *J* = 8.0, 2.2, 1.1 Hz, 1H), 7.33 (t, *J* = 1.9 Hz, 1H), 7.24 (dd, 2H), 7.21 (t, *J* = 7.9 Hz, 1H), 7.11 (t, 2H), 6.98 (tt, 1H), 5.08 (td, *J* = 10.7, 4.4 Hz, 1H), 2.22 (ddd, *J* = 12.3, 10.5, 3.6 Hz, 1H), 1.95 (dtd, *J* = 12.2, 3.9, 2.2 Hz, 1H), 1.85 (dq, *J* = 13.6, 3.5 Hz, 1H), 1.72 (dq, 1H), 1.59 – 1.52 (m, 1H), 1.32 (s, 3H), 1.21 (s, 3H), 1.18 (dd, *J* = 13.1, 3.5 Hz, 1H), 1.07 (td, *J* = 12.2, 10.8 Hz, 1H), 1.00 – 0.93 (m, 1H), 0.90 (d, *J* = 6.6 Hz, 3H).

^{13}C{^{1}H} NMR (151 MHz, $CDCl_3$) δ 164.6, 151.8, 134.0, 132.6, 132.2, 129.6, 129.2, 128.1, 127.8, 125.3, 125.2, 75.4, 50.6, 41.9, 39.7, 34.8, 31.5, 29.1, 26.7, 24.1, 21.9.

HRMS (ES+) m/z calc'd for $C_{23}H_{27}ClO_2$ $[M]^+$: 370.1700, found 370.1702.

IR (film) ν 2955, 2923, 1714, 1574, 1293, 1256 cm^{-1}.

General procedure reductive auxiliary removal.

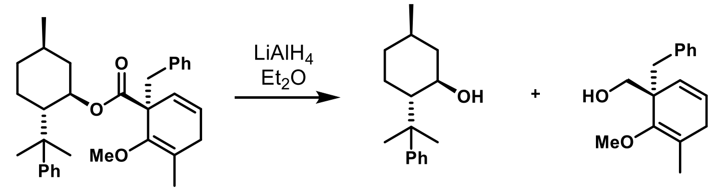

10 (–)-8-Phenylmenthol 36

Alcohol (36). To a flame-dried 2-dram vial equipped with a magnetic stir bar was added diene **10** (135 mg, 0.3 mmol, 1.0 equiv.) in Et_2O (3 mL) and the resulting solution was cooled to 0 °C. $LiAlH_4$ (24 mg, 0.6 mmol, 2.2 equiv.) was added. The reaction mixture was warmed to room temperature and stirred for 1 h. The reaction mixture was diluted with Et_2O (10 mL) and cooled to 0 °C. A saturated solution of Rochelle's salt (10 mL) was added dropwise and the mixture was stirred until distinct layers formed. The aqueous solution was extracted with Et_2O (3 x 10 mL). The combined organic extracts were dried over Na_2SO_4, filtered, and concentrated in *vacuo*. The crude residue was purified with flash chromatography (SiO_2, 2% EtOAc in hexanes) to afford (–)-8-phenylmenthol (47 mg, 71% yield) and alcohol **36** as a colorless oil (46 mg, 66% yield, 8.1:1 e.r.).

^{1}H NMR (500 MHz, $CDCl_3$) δ 7.22 – 7.12 (m, 3H), 7.12 – 7.08 (m, 2H), 5.75 (dt, *J* = 10.0, 3.4 Hz, 1H), 5.30 (dt, *J* = 9.9, 2.0 Hz, 1H), 3.84 – 3.78 (m, 1H), 3.76 (s, 3H), 3.41 (dd, *J* = 10.4, 2.2 Hz, 1H), 2.89 (d, *J* = 12.9 Hz, 1H), 2.55 (d, *J* = 12.9 Hz, 1H), 2.50 (ddd, *J* = 3.8, 1.9, 1.0 Hz, 1H), 2.17 (dddd, *J* = 22.1, 3.2, 2.3, 1.1 Hz, 1H), 1.79 – 1.70 (m, 1H), 1.64 (s, 3H).

^{13}C{^{1}H} NMR (126 MHz, $CDCl_3$) δ 149.4, 137.6, 130.6, 129.6, 127.8, 127.4, 125.9, 118.8, 68.0, 61.2, 49.3, 40.7, 33.4, 16.2.

IR (film) ν 3433, 3027, 2920, 2850, 1657, 722 cm^{-1}.

$[\alpha]_D^{22}$ +93.4 (c = 0.102, $CHCl_3$).

Chiral SFC: CHIRALCEL OD-H, 2% *i*-PrOH, 2.5 mL/min, 220 nm, 44 °C, nozzle pressure = 200 bar CO_2, t_{R1} (major) = 4.5 min, t_{R2} (minor) = 5.1 min.

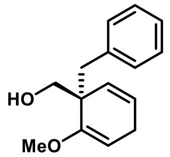

35

Alcohol (35). Prepared according to **general procedure for reductive auxiliary removal**, using ester **5** (55 mg, 0.12 mmol, 1.0 equiv.) and $LiAlH_4$ (9.1 mg, 0.24 mmol, 2 equiv.) to give (–)-8-phenylmenthol (15 mg, 53% yield) and **35** as a colorless oil (15 mg, 55% yield, 5.2:1 e.r.).

^{1}H NMR (500 MHz, C_6D_6) δ 7.16 – 7.10 (m, 4H), 7.10 – 7.03 (m, 1H), 5.58 (dtd, *J* = 10.0, 3.4, 1.2 Hz, 1H), 5.18 (dt, *J* = 10.0, 2.1 Hz, 1H), 4.35 (t, *J* = 3.7 Hz, 1H), 3.88 (dd, *J* = 10.3, 8.4 Hz, 1H), 3.48 (dd, *J* = 10.3, 4.1 Hz, 1H), 3.12 (d, *J* = 13.0 Hz, 1H), 3.09 (s, 3H), 2.48 (d, *J* = 13.0 Hz, 1H), 2.37 (dtd, *J* = 22.1, 3.7, 1.9 Hz, 1H), 2.18 (dq, *J* = 22.1, 3.0 Hz, 1H), 1.67 – 1.61 (m, 1H).

$^{13}C\{^1H\}$ **NMR** (151 MHz, C_6D_6) δ 154.6, 138.6, 130.7, 129.5, 128.6, 128.5, 127.8, 127.2, 126.2, 94.6, 69.2, 53.4, 48.0, 40.9, 26.7.
HRMS (ES+) m/z calc'd for $C_{15}H_{18}O_2H$ $[M + H]^+$: 231.1385, found 231.1380.
IR (film) ν 3391 (broad), 3027, 2923, 2853, 1603, 699 cm^{-1}.
$[\alpha]_D^{20}$ –2.34 (c = 1.08, $CHCl_3$).
Chiral SFC: CHIRALCEL OD-H, 2% *i*-PrOH, 2.5 mL/min, 220 nm, 44 °C, nozzle pressure = 200 bar CO_2, t_{R1} (major) = 5.2 min, t_{R2} (minor) = 6.4 min.

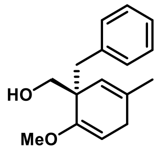

37

Alcohol (37). Prepared according to **general procedure for reductive auxiliary removal**, using ester **15** (64 mg, 0.12 mmol, 1.0 equiv.) and $LiAlH_4$ (9.2 mg, 0.24 mmol, 2.0 equiv.) to give (–)-8-phenylmenthol (25 mg, 89% yield) and **37** as a colorless oil (25 mg, 82% yield, 7.1:1 e.r.).
^{1}**H NMR** (500 MHz, C_6D_6) δ 7.14 (d, J = 7.4 Hz, 2H), 7.10 (d, 2H), 7.08 – 7.04 (m, 1H), 4.91 (d, J = 1.5 Hz, 1H), 4.36 (t, J = 3.6 Hz, 1H), 3.90 (dd, J = 10.2, 8.3 Hz, 1H), 3.51 (dd, J = 10.3, 3.2 Hz, 1H), 3.12 (s, 3H), 3.10 (s, 1H), 2.49 (d, J = 12.9 Hz, 1H), 2.28 (dd, J = 21.5, 3.8 Hz, 1H), 2.07 (d, J = 21.5 Hz, 1H), 1.71 (dd, J = 8.7, 4.1 Hz, 1H), 1.48 (s, 3H).
$^{13}C\{^1H\}$ **NMR** (151 MHz, C_6D_6) δ 154.9, 138.8, 134.7, 130.7, 127.7, 126.1, 124.5, 94.5, 69.4, 53.6, 48.6, 41.2, 31.4, 22.5.
HRMS (ES+) m/z calc'd for $C_{16}H_{20}O_2Na$ $[M + Na]^+$: 267.1361, found 267.1358.
IR (film) ν 3425 (broad), 3028, 2933, 2874, 1603, 1208, 699 cm^{-1}.
$[\alpha]_D^{22}$ +44.98 (c = 1.20, $CHCl_3$).
Chiral SFC: CHIRALCEL OD-H, 2% *i*-PrOH, 2.5 mL/min, 220 nm, 44 °C, nozzle pressure = 200 bar CO_2, t_{R1} (major) = 4.0 min, t_{R2} (minor) = 4.9 min.

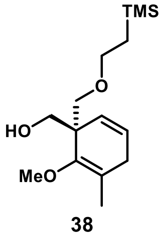

38

Alcohol (38). Prepared according to **general procedure for reductive auxiliary removal**, using ester **14** (44 mg, 0.086 mmol, 1.0 equiv.) and $LiAlH_4$ (9.8 mg, 0.26 mmol, 3.0 equiv.) to give (–)-8-phenylmenthol (19 mg, 95% yield) and **38** as a colorless oil (22 mg, 88% yield, 4.4:1 e.r.).
^{1}**H NMR** (500 MHz, C_6D_6) δ 5.68 (s, 2H), 3.81 (dd, *J* = 10.4, 8.6 Hz, 1H), 3.73 (dd, *J* = 10.5, 3.4 Hz, 1H), 3.58 (d, *J* = 8.7 Hz, 1H), 3.49 (s, 3H), 3.45 (dd, *J* = 8.4, 2.5 Hz, 1H), 3.40 (td, *J* = 7.4, 2.4 Hz, 2H), 2.48 (q, *J* = 22.3 Hz, 2H), 1.90 (dt, *J* = 9.1, 4.1 Hz, 1H), 1.57 (s, 3H), 0.84 (td, *J* = 7.5, 2.5 Hz, 2H), -0.01 (s, 9H).

$^{13}C\{^1H\}$ **NMR** (151 MHz, C_6D_6) δ 150.5, 130.0, 126.1, 117.8, 74.4, 68.8, 67.4, 60.6, 49.0, 33.7, 18.3, 16.2, -1.2.
HRMS (ES+) m/z calc'd for $C_{15}H_{28}O_3SiNa$ $[M + Na]^+$: 307.1705, found 307.1705.
IR (film) ν 3460 (broad), 2950, 2852, 1696, 1246, 835 cm^{-1}.
$[\alpha]_D^{22}$ +29.12 (c = 1.03, $CHCl_3$).
Chiral SFC: CHIRALPAK OD-H, 1% *i*-PrOH/CO_2, 3.5 mL/min, 220 nm, t_{R1} (major) = 5.2 min, t_{R2} (minor) = 5.7 min.

Substrates with low yields and/or low diastereoselectivity

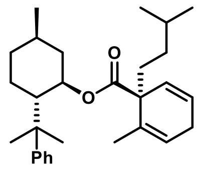

S12

Diene (S12). Prepared according to **general procedure for Birch reduction/alkylation of ester**, using ester **29** (85.0 mg, 0.2 mmol, 1.0 equiv.) and 1-iodo-3-methylbutane (140 mg, 0.7 mmol, 3.0 equiv.) to give **S12** as a colorless oil (62.9 mg, 61% yield, 1:1 d.r.).
^{1}**H NMR** (500 MHz, $CDCl_3$) δ 7.31 – 7.26 (m, 4H), 7.18 – 7.12 (m, 1H), 5.90 – 5.83 (m, 1H), 5.64 (s, 1H), 5.33 (dd, *J* = 15.3, 9.9 Hz, 1H), 4.77 (qd, *J* = 10.2, 4.2 Hz, 1H), 2.76 – 2.58 (m, 2H), 1.98 – 1.86 (m, 2H), 1.86 – 1.75 (m, 1H), 1.66 (dd, *J* = 3.4, 1.8 Hz, 3H), 1.49 (p, *J* = 6.8 Hz, 2H), 1.41 (td, *J* = 13.0, 4.2 Hz, 2H), 1.31 (d, *J* = 4.9 Hz, 3H), 1.23 (d, *J* = 6.7 Hz, 3H), 1.09 – 0.99 (m, 1H), 0.99 – 0.91 (m, 2H), 0.91 – 0.86 (m, 2H), 0.88 (s, 3H), 0.87 (s, 3H), 0.83 (dd, *J* = 6.4, 4.2 Hz, 3H), 0.79 – 0.66 (m, 1H).
$^{13}C\{^1H\}$ **NMR** (126 MHz, $CDCl_3$) δ 173.0, 150.8, 131.2, 128.8, 126.6, 126.2, 125.7, 125.5, 124.2, 76.1, 52.3, 50.7, 42.1, 40.6, 34.8, 33.7, 33.2, 31.5, 31.0, 28.8, 27.9, 27.4, 23.6, 23.1, 22.8, 22.0, 20.2.
HRMS (ES+) m/z calc'd for $C_{29}H_{42}O_2Na$ $[M + Na]^+$: 445.3083, found 445.3091.
IR (film) ν 2953, 2924, 2869, 1716, 1688, 1600, 1215, 737 cm^{-1}.

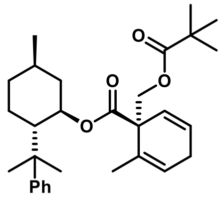

S13

Diene (S13). Prepared according to **general procedure for Birch reduction/alkylation of ester**, using ester **29** (130 mg, 0.3 mmol, 1.0 equiv.) and iodomethyl pivalate (270 mg, 1.1 mmol, 3.0 equiv.) to give **S13** as a colorless oil (118 mg, 68% yield, 1:1 d.r.).
^{1}**H NMR** (500 MHz, $CDCl_3$) δ 7.32 – 7.26 (m, 4H), 7.19 – 7.11 (m, 1H), 5.94 (dtd, *J* = 8.8, 4.0, 2.2 Hz, 1H), 5.72 (s, 1H), 5.50 (ddd, *J* = 10.1, 8.1, 2.0 Hz, 1H), 4.83 (tt, *J* = 9.6, 5.0 Hz, 1H), 4.32 (d, *J* = 11.0 Hz, 1H), 4.16 (d, *J* = 11.0 Hz, 1H), 2.77 – 2.58 (m, 2H), 2.00 – 1.83 (m, 2H), 1.72 (s,

3H), 1.55 – 1.47 (m, 1H), 1.31 (s, 3H), 1.23 (s, 3H), 1.14 (s, 9H), 1.03 – 0.86 (m, 4H), 0.84 (d, *J* = 3.2 Hz, 3H), 0.74 (pd, *J* = 13.2, 3.4 Hz, 1H).
^{13}C{^{1}H} NMR (126 MHz, $CDCl_3$) δ 178.2, 171.1, 150.7, 128.2, 126.7, 125.8, 125.5, 125.3, 124.9, 124.0, 76.7, 65.5, 52.2, 50.3, 41.8, 40.3, 34.6, 31.4, 30.6, 29.8, 27.4, 27.2, 27.0, 24.3, 21.9, 19.9.
HRMS (ES+) m/z calc'd for $C_{30}H_{42}O_4Na$ $[M + Na]^+$: 489.2981, found 489.2987.
IR (film) ν 2958, 1719, 1226, 1151, 729 cm^{-1}.

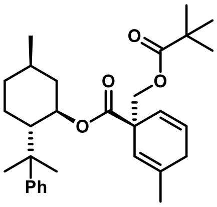

S14

Diene (S14). Prepared according to **general procedure for Birch reduction/alkylation of ester**, using ester **32** (92.9 mg, 0.3 mmol, 1.0 equiv.) and iodomethyl pivalate (192 mg, 0.8 mmol, 3.0 equiv.) to give **S14** as a colorless oil (110 mg, 89% yield, 1:1 d.r.).
^{1}H NMR (500 MHz, $CDCl_3$) δ 7.32 – 7.26 (m, 4H), 7.18 – 7.12 (m, 1H), 5.91 (dt, *J* = 16.9, 3.4 Hz, 1H), 5.67 (dq, *J* = 22.2, 2.1 Hz, 1H), 5.38 (dp, *J* = 5.3, 1.7 Hz, 1H), 4.83 (q, *J* = 4.4 Hz, 1H), 4.13 – 4.01 (m, 2H), 2.63 – 2.49 (m, 2H), 1.99 – 1.88 (m, 2H), 1.74 (s, 3H), 1.51 (dp, *J* = 9.7, 3.5 Hz, 1H), 1.47 – 1.37 (m, 1H), 1.32 (s, 3H), 1.24 (s, 3H), 1.17 (d, *J* = 2.2 Hz, 9H), 1.01 – 0.85 (m, 3H), 0.83 (d, *J* = 6.7 Hz, 3H), 0.75 (qd, 1H).
^{13}C{^{1}H} NMR (126 MHz, $CDCl_3$) δ 178.0, 172.0, 150.6, 134.7, 128.1, 127.2, 125.5, 124.2, 118.5, 76.2, 69.2, 50.3, 49.8, 41.9, 40.4, 39.0, 34.6, 31.4, 31.2, 30.1, 27.4, 27.3, 27.2, 24.2, 23.3, 21.8.
HRMS (ES+) m/z calc'd for $C_{30}H_{42}O_4H$ $[M + H]^+$: 467.3161, found 467.3156.
IR (film) ν 2956, 2871, 1727, 1281, 1217, 700 cm^{-1}.

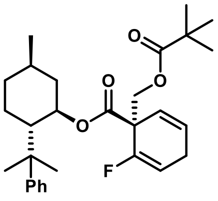

S15

Diene (S15). Prepared according to **general procedure for Birch reduction/alkylation of ester**, using ester **30** (85.2 mg, 0.2 mmol, 1.0 equiv.) and iodomethyl pivalate (175 mg, 0.7 mmol, 3.0 equiv.) to give **S15** as a colorless oil (75.9 mg, 67% yield, 2:1 d.r.).
^{1}H NMR (500 MHz, $CDCl_3$) δ 7.31 – 7.26 (m, 4H), 7.16 (s, 1H), 5.97 – 5.86 (m, 1H), 5.64 (dq, *J* = 26.5, 2.1 Hz, 1H), 5.56 – 5.44 (m, 1H), 4.86 (qd, *J* = 10.8, 4.4 Hz, 1H), 4.21 (dd, 2H), 2.89 – 2.58 (m, 2H), 2.01 – 1.85 (m, 2H), 1.51 (tt, *J* = 9.9, 4.4 Hz, 1H), 1.42 (dt, *J* = 9.4, 4.1 Hz, 1H), 1.30 (s, 3H), 1.24 (d, *J* = 1.6 Hz, 3H), 1.18 (s, 9H), 1.05 – 0.86 (m, 3H), 0.84 (d, *J* = 5.3 Hz, 3H), 0.75 (ddd, *J* = 19.8, 6.9, 3.3 Hz, 1H).

$^{13}C\{^1H\}$ NMR (151 MHz, $CDCl_3$) δ 178.1, 171.7, 150.7, 128.2, 127.4, 127.1, 125.8, 125.5, 124.2, 124.1, 103.4, 76.3, 69.0, 63.8, 50.4, 50.2, 48.6, 41.9, 41.4, 40.4, 34.5, 31.4, 29.8, 27.3, 26.4, 24.6, 21.8.
HRMS (ES+) m/z calc'd for $C_{29}H_{39}FO_4Na$ $[M + Na]^+$: 493.2730, found 493.2731.
IR (film) ν 2958, 1726, 1245, 729, 700 cm^{-1}.

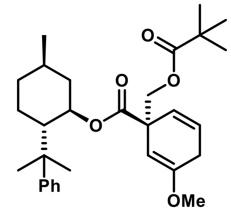

S16

Diene (S16). Prepared according to **general procedure for Birch reduction/alkylation of ester**, using ester **32** (101 mg, 0.3 mmol, 1.0 equiv.) and iodomethyl pivalate (199 mg, 0.8 mmol, 3.0 equiv.) to give **S16** as a colorless oil (105 mg, 79% yield, 1:1 d.r.).
^1H NMR (500 MHz, C_6D_6) δ 7.25 – 7.17 (m, 4H), 7.11 – 7.04 (m, 1H), 5.86 (tq, *J* = 9.9, 2.1 Hz, 1H), 5.59 (dq, *J* = 9.8, 3.3 Hz, 1H), 5.03 (qd, *J* = 10.8, 4.4 Hz, 1H), 4.78 (d, 1H), 4.49 – 4.37 (m, 2H), 3.27 (s, 3H), 2.70 – 2.53 (m, 2H), 2.09 (dddd, *J* = 13.9, 7.8, 6.2, 3.9 Hz, 1H), 1.86 (dddd, *J* = 12.2, 10.6, 4.5, 3.4 Hz, 1H), 1.39 (s, 3H), 1.30 – 1.26 (m, 1H), 1.25 (s, 3H), 1.21 (s, 9H), 1.16 – 1.05 (m, 1H), 0.96 (tdd, *J* = 12.2, 10.6, 4.3 Hz, 1H), 0.82 – 0.71 (m, 2H), 0.71 (d, *J* = 4.4 Hz, 3H), 0.61 – 0.49 (m, 1H).
$^{13}C\{^1H\}$ NMR (126 MHz, $CDCl_3$) δ 177.3, 172.0, 156.0, 150.6, 128.5, 126.1, 125.8, 125.7, 125.3, 92.4, 76.1, 70.0, 53.8, 51.2, 50.6, 42.1, 40.6, 39.0, 34.6, 31.4, 30.8, 29.3, 27.7, 27.4, 24.3, 21.9.
HRMS (ES+) m/z calc'd for $C_{30}H_{42}O_5Na$ $[M + Na]^+$: 505.2930; found 505.2915.
IR (film) ν 2956, 2919, 1728, 1651, 1600, 1216, 700 cm^{-1}.

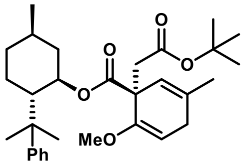

S17

Diene (S17). Prepared according to **general procedure for Birch reduction/alkylation of ester**, using ester **S8** (100 mg, 0.26 mmol, 1.0 equiv.) and iodomethyl pivalate (0.12 mL, 0.79 mmol, 3.0 equiv.) to give **S17** as a colorless oil (17 mg, 13% yield, 4:1 d.r.).
^1H NMR (500 MHz, $CDCl_3$) δ 7.31 – 7.27 (m, 4H), 7.18 – 7.13 (m, 1H), 5.30 (q, *J* = 1.6 Hz, 1H), 4.79 (t, *J* = 3.5 Hz, 1H), 4.75 (td, *J* = 10.6, 4.3 Hz, 1H), 3.50 (s, 3H), 2.82 (d, *J* = 13.9 Hz, 1H), 2.79 – 2.64 (m, 2H), 2.59 (d, *J* = 13.9 Hz, 1H), 1.96 – 1.88 (m, 1H), 1.84 (ddd, *J* = 12.2, 10.5, 3.5 Hz, 1H), 1.76 (s, 3H), 1.44 (s, 3H), 1.38 (s, 9H), 1.29 (d, *J* = 8.3 Hz, 3H), 1.23 (s, 3H), 0.97 – 0.84 (m, 2H), 0.83 (d, *J* = 6.5 Hz, 3H), 0.71 (qd, *J* = 12.7, 3.4 Hz, 1H).
$^{13}C\{^1H\}$ NMR (151 MHz, $CDCl_3$) δ 172.0, 170.4, 151.8, 150.7, 133.8, 128.1, 126.0, 125.5, 121.2, 93.7, 54.2, 50.5, 41.5, 41.4, 40.4, 34.7, 31.4, 31.3, 30.2, 28.2, 28.1, 27.6, 23.2, 22.7, 21.9.

HRMS (ES+) m/z calc'd for $C_{31}H_{44}O_5Na$ $[M + Na]^+$: 519.3087, found 519.3071.
IR (film) ν 2953, 2922, 1718, 1580, 1249, 700 cm^{-1}.

Auxiliary removal with MeMgBr

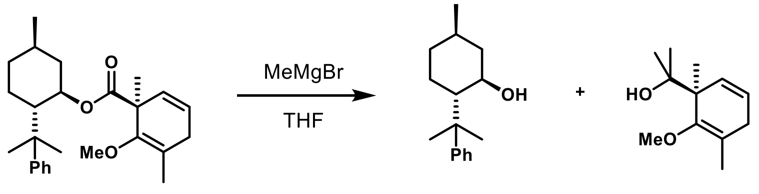

11 (–)-8-Phenylmenthol S18

Alcohol (S18). A flame-dried 2-dram vial equipped with a stir bar was charged diene **11** (60.0 mg, 0.2 mmol, 1.0 equiv.) in THF (1.5 mL), followed by the addition of MeMgBr (3 M in ether, 0.25 mL, 0.8 mmol, 5.0 equiv.) dropwise at 0 °C. The reaction was allowed to slowly warm up to room temperature and stirred for 2 h. Saturated aqueous NH_4Cl (0.5 mL) was added. The reaction mixture was extracted with Et_2O (3 x 5 mL), dried over anhydrous $MgSO_4$, filtered, and concentrated *in vacuo*. The product was purified by flash column chromatography (SiO_2, 3% EtOAc in hexanes) to afford 42 mg of material consisting of (–)-8-phenylmenthol (26 mg, 75% yield) and alcohol **S18** (16 mg, 52% yield, 4.3:1 e.r.) as a colorless oil. *Notes: (–)-8-phenylmenthol and alcohol S9 are inseparable by flash column chromatography. A small sample was purified by HPLC with 40% MeCN/H_2O for analytical purposes.*
^{1}H NMR (600 MHz, $CDCl_3$) δ 5.61 (ddd, *J* = 10.1, 3.7, 2.9 Hz, 1H), 5.45 (ddd, *J* = 10.0, 2.4, 1.5 Hz, 1H), 3.74 (s, 1H), 3.70 (s, 3H), 2.73 – 2.61 (m, 2H), 1.71 (t, *J* = 0.9 Hz, 3H), 1.30 (s, 3H), 1.14 (s, 3H), 1.11 (s, 3H).
^{13}C{^{1}H} NMR (151 MHz, $CDCl_3$) δ 153.7, 133.2, 123.2, 76.5, 61.9, 47.5, 33.6, 29.8, 27.5, 23.6, 20.6, 15.8.
HRMS (ES+) m/z calc'd for $C_{16}H_{20}O_2Na$ $[M + Na]^+$: 219.1361, found 219.1361.
IR (film) ν 3414 (broad), 2955, 2923, 2853, 1260, 799 cm^{-1}.
Chiral SFC: CHIRALPAK AD-H, 3% *i*-PrOH/CO_2, 3.5 mL/min, 220 nm, t_{R1} (minor) = 1.6 min, t_{R2} (major) = 1.8 min.

Auxiliary removal screening conditions

Table S1.

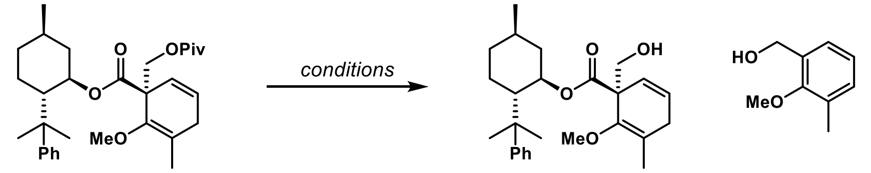

13 S19 S20

Conditions	Results	Conditions	Results
nBu$_4$NOH, dioxane, 50 °C	rearomatization	DBU, LiBr, MeOH	rearomatization
La/In/Ti/Zn/Yb/Sc/Sn triflates and other Lewis acid salts, MeOH, reflux	no conversion	Te, NaBH$_4$, *t*-BuOH, DMF, 80 °C	no conversion
		Et$_2$AlCl, MeONHMe·HCl, CH$_2$Cl$_2$	no conversion
KO*t*-Bu, DMSO, 80 °C	65% of benzyl alcohol **S20**; 94% of phenylmenthol	AlMe$_3$, EtSH, 1.5:1 toluene:CH$_2$Cl$_2$	70% of **S19**

Table S2.

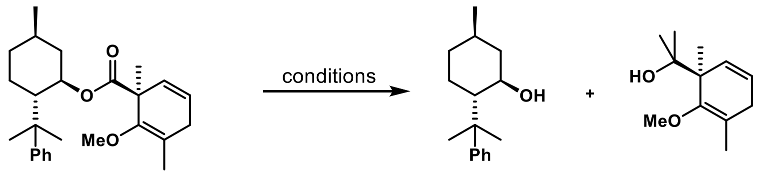

11 S4 S18

Conditions	Results	Conditions	Results
KOH, EtOH at 100 °C, 3d	no conversion	MeMgBr, THF, r.t.- 50 °C, 2 h	75% of phenylmenthol, 52% of alcohol **S18**
Zn, MeOH at 110 °C, 3d	no conversion		

Table S3.

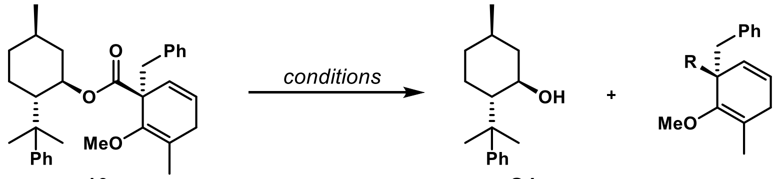

10 S4

Conditions	Results	Conditions	Results
nBu$_4$NOH, MeOH/dioxane at 95 °C, 2 d	no conversion	HCl, dioxane at 60 °C	rearomatization
KOH, MeOH/dioxane at 95 °C, 2 d	no conversion	LiOOH, THF/H$_2$O at 60 °C	rearomatization
KOH, dioxane/H2O at 195 °C in microwave	rearomatization	H$_2$SO$_4$, DCM at r.t.	unknown product
KOH, MeOH/H$_2$O at 95 °C	no conversion	HCl/HOAc at 50 °C	complex mixture
KOH, DMSO/H$_2$O at 95 °C	no conversion	H$_3$PO$_4$, HCl/HOAc at r.t.	no conversion
TFA, DCM at at 60 °C	no conversion	DIBAL-H, THF at –78 °C	no conversion

General procedure for Birch reduction/alkylation of carboxylic acid to produce racemic products for e.r. determination.

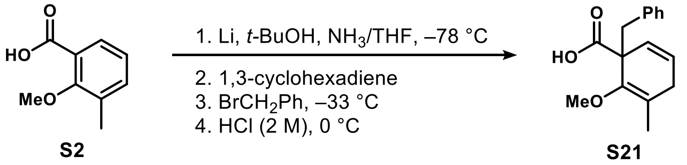

Carboxylic acid (S21). Liquid NH_3 (4 mL) was distilled into a flame-dried 25 mL three-neck round bottom flask at –78 °C equipped with a glass-encased stir bar and a dry ice condenser. From a separate flame-dried 10 mL pear-shaped flask, a solution of benzoic acid **S2** (255 mg, 1.5 mmol, 1.0 equiv.) and *t*-BuOH (1 M in THF, 1.5 mL, 1.5 mmol, 1.0 equiv.) in anhydrous THF (3 mL) was added to the reaction flask via syringe. Freshly cut lithium wire (27 mg, 3.8 mmol, 2.5 equiv.) was added in three pieces over 10 min, which resulted in a sustained deep blue solution after 10 min. The reaction mixture was stirred for 30 min at –78 °C before adding 1,3-cyclohexadiene (0.29 mL, 3.1 mmol, 2.0 equiv.) dropwise which led to discharge of the color. After stirring for an additional 10 min at –78 °C, benzyl bromide (0.55 mL, 4.6 mmol, 3.0 equiv.) was added dropwise. The cooling bath was removed, and the solvent was allowed to evaporate under a flow of argon. The reaction mixture was cooled to 0 °C followed by the addition of aqueous HCl (2 M) until the pH of the mixture fell below 3. The mixture was extracted with ether (3 × 15 mL). The combined organic extracts were washed with brine (30 mL), dried over anhydrous $MgSO_4$, and evaporated under reduced pressure to give the crude product as a light-yellow oil. This crude product was recrystallized in EtOAc and hexanes to afford **S21** as a white solid (245 mg, 62% yield).

^{1}H NMR (600 MHz, $CDCl_3$) δ 7.21 – 7.14 (m, 3H), 7.09 (dd, *J* = 7.6, 1.9 Hz, 2H), 5.69 (dt, *J* = 9.9, 3.4 Hz, 1H), 5.53 (dt, *J* = 9.8, 2.0 Hz, 1H), 3.76 (s, 3H), 3.31 (d, *J* = 13.4 Hz, 1H), 3.02 (d, *J* = 13.4 Hz, 1H), 2.56 – 2.49 (m, 1H), 1.96 (dt, *J* = 22.2, 3.0 Hz, 1H), 1.60 (s, 3H).

^{13}C{^{1}H} NMR (151 MHz, $CDCl_3$) δ 178.3, 146.8, 137.0, 131.0, 127.3, 127.0, 126.6, 126.2, 118.2, 61.2, 54.2, 40.5, 33.1, 16.2.

HRMS (ES+) m/z calc'd for $C_{16}H_{18}O_3Na$ $[M + Na]^+$: 281.1154, found 281.1145.

IR (film) ν 3029, 2938, 2632, 1693, 1604, 722 cm^{-1}.

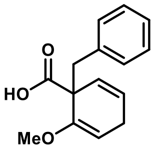

S22

d.r.)**Carboxylic acid (S22).** Prepared according to **general procedure for Birch reduction/alkylat-ion of carboxylic acid**, using 2-methoxybenzoic acid (244 mg, 1.6 mmol, 1.0 equiv.) and benzyl bromide (0.57 mL, 4.8 mmol, 3.0 equiv.) to give **S22** as a white solid (265 mg, 68% yield).

^{1}H NMR (600 MHz, $CDCl_3$) δ 10.38 (broad s, 1H), 7.18 (qd, *J* = 7.7, 3.8 Hz, 3H), 7.06 (dd, *J* = 7.6, 1.8 Hz, 2H), 5.82 (dt, *J* = 10.1, 3.4 Hz, 1H), 5.61 (dt, *J* = 10.0, 2.2 Hz, 1H), 4.74 – 4.69 (m, 1H), 3.60 (d, *J* = 1.9 Hz, 3H), 3.38 (d, *J* = 13.4 Hz, 1H), 2.99 (d, *J* = 13.4 Hz, 1H), 2.68 – 2.60 (m, 1H), 2.24 (dd, *J* = 22.4, 3.0 Hz, 1H).
$^{13}C\{^1H\}$ NMR (151 MHz, $CDCl_3$) δ 177.2, 151.1, 137.0, 130.7, 127.60, 127.55, 126.3, 126.1, 95.4, 54.3, 53.0, 40.7, 26.2.
HRMS (ES+) m/z calc'd for $C_{15}H_{16}O_3Na$ $[M + Na]^+$: 267.0997, found 267.0993.

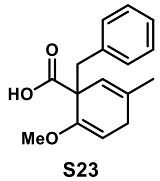

S23

Carboxylic acid (S23). Prepared according to **general procedure for Birch reduction/alkylation of carboxylic acid**, using 2-methoxy-5-methylbenzoic acid (312 mg, 1.9 mmol, 1.0 equiv.) and benzyl bromide (0.67 mL, 5.6 mmol, 3.0 equiv.) to give **S23** as a white solid (318 mg, 66% yield).
^{1}H NMR (500 MHz, CDC13) δ 7.20 – 7.14 (m, 3H), 7.02 (dd, J = 7.4, 2.2 Hz, 2H), 5.30 (d, J = 1.6 Hz, 1H), 4.65 (t, J = 3.6 Hz, 1H), 3.58 (s, 3H), 3.33 (d, J = 13.2 Hz, 1H), 2.96 (d, J = 13.2 Hz, 1H), 2.49 (dd, J = 21.8, 3.9 Hz, 1H), 2.05 (d, J = 22.4 Hz, 1H), 1.66 (s, 3H).
$^{13}C\{^1H\}$ NMR (151 MHz, $CDCl_3$) δ 178.2, 151.2, 137.3, 135.6, 130.7, 127.4, 126.2, 120.9, 95.2, 54.3, 53.9, 40.9, 30.9, 22.5.
HRMS (ES+) m/z calc'd for $C_{16}H_{18}O_3H$ [M + H]+: 259.1334, found 259.1340.
IR (film) ν 2926, 2636, 1693, 1603, 697 cm^{-1}.

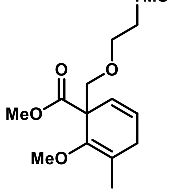

S24

Ester (S24). Prepared according to **general procedure for Birch reduction/alkylation of ester**, using methyl 2-methoxy-3-methylbenzoate (122 mg, 0.68 mmol, 1.0 equiv.) and (2-(chloromethoxy)ethyl)trimethylsilane (0.36 mL, 2.0 mmol, 3.0 equiv.) to give **S24** as a colorless oil (167 mg, 79% yield).
^{1}H NMR (500 MHz, CDC13) δ 5.87 (dt, J = 9.8, 3.4 Hz, 1H), 5.60 (dt, J = 9.9, 2.0 Hz, 1H), 3.74 (s, 2H), 3.69 (s, 3H), 3.61 (s, 3H), 3.56 (dd, J = 8.7, 7.4 Hz, 2H), 2.82 – 2.68 (m, 2H), 1.74 (s, 3H), 0.96 – 0.83 (m, 2H), -0.03 (s, 9H).
$^{13}C\{^1H\}$ NMR (126 MHz, $CDCl_3$) δ 173.0, 146.8, 127.0, 126.6, 118.4, 72.1, 68.8, 60.9, 54.2, 52.4, 33.4, 18.1, 16.4, -1.3.
HRMS (ES+) m/z calc'd for $C_{16}H_{28}O_4SiNa$ $[M + Na]^+$: 335.1655, found 335.1646.
IR (film) ν 2950, 2857, 1732, 1700, 1245 cm^{-1}.

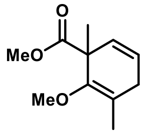

S25

Ester (S25). Prepared according to **general procedure for Birch reduction/alkylation of ester**, using methyl 2-methoxy-3-methylbenzoate (100 mg, 0.56 mmol, 1.0 equiv.) and iodomethane (0.10 mL, 1.7 mmol, 3.0 equiv.) to give **S25** as a yellow oil. The material was carried forward to the next step without further purification.
HRMS (ES+) m/z calc'd for $C_{16}H_{20}O_2Na$ $[M + Na]^+$: 219.0997, found 219.0988.

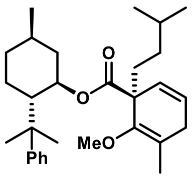

S26

Ester (S26). Prepared according to **general procedure for Birch reduction/alkylation of ester**, using ester **S5** (117 mg, 0.31 mmol, 1.0 equiv.) and 1-iodo-3-methylbutane (0.12 mL, 0.92 mmol, 3.0 equiv.) to give **S26** as a colorless oil (88 mg, 64% yield, 8:1 d.r.).
^{1}H NMR (600 MHz, $CDCl_3$) δ 7.31 – 7.25 (m, 4H), 7.15 (dtd, *J* = 8.4, 4.9, 3.4 Hz, 1H), 5.78 (dt, *J* = 9.9, 3.3 Hz, 1H), 5.26 (dt, *J* = 9.8, 2.0 Hz, 1H), 4.77 (tt, *J* = 10.4, 5.2 Hz, 1H), 3.65 (s, 3H), 2.79 – 2.63 (m, 2H), 2.00 – 1.86 (m, 3H), 1.70 (s, 3H), 1.55 – 1.37 (m, 4H), 1.32 (s, 3H), 1.31 – 1.23 (m, 2H), 1.23 (s, 3H), 1.05 – 0.91 (m, 3H), 0.90 – 0.85 (m, 6H), 0.84 (d, *J* = 6.4 Hz, 3H), 0.75 (qd, *J* = 13.0, 3.6 Hz, 1H).
^{13}C{^{1}H} NMR (151 MHz, $CDCl_3$) δ 173.6, 151.0, 147.8, 128.3, 128.1, 125.9, 125.6, 125.4, 117.2, 61.1, 53.8, 50.5, 41.7, 40.5, 34.7, 33.7, 33.6, 31.9, 31.5, 29.9, 28.6, 27.6, 23.9, 23.0, 22.9, 21.9, 16.4.
HRMS (ES+) m/z calc'd for $C_{30}H_{44}O_3Na$ $[M + Na]^+$: 475.3188, found 475.3196.

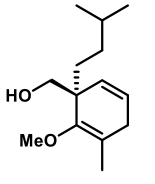

S27

Alcohol (S27). Prepared according to **general procedure for reductive auxiliary removal**, using ester **S25** (32 mg, 0.071 mmol, 1.0 equiv.) and $LiAlH_4$ (5.4 mg, 0.14 mmol, 2.0 equiv.) to give (–)-8-phenylmenthol (16 mg, 97% yield) and **S27** as a colorless oil (13 mg, 83% yield, 6:1 e.r.).
^{1}H NMR (500 MHz, C_6D_6) δ 5.88 (dt, *J* = 10.0, 3.3 Hz, 1H), 5.22 (dt, *J* = 9.9, 2.0 Hz, 1H), 3.68 (s, 3H), 3.63 (dd, *J* = 10.3, 8.1 Hz, 1H), 3.27 (dd, *J* = 10.4, 3.6 Hz, 1H), 2.71 (dq, *J* = 3.2, 0.9 Hz,

2H), 1.74 (s, 3H), 1.63 (dt, J = 15.2, 6.5 Hz, 2H), 1.46 (dp, J = 13.1, 6.5 Hz, 1H), 1.13 – 1.01 (m, 3H), 0.85 (dd, J = 6.6, 4.2 Hz, 6H).
^{13}C{^{1}H} NMR (151 MHz, C_6D_6) δ 149.8, 131.0, 127.2, 118.3, 69.0, 61.2, 48.3, 33.9, 33.7, 32.1, 28.7, 23.0, 22.8, 16.4.
HRMS (ES+) m/z calc'd for $C_{14}H_{24}O_2Na$ $[M + Na]^+$: 247.1674, found 247.1668.
IR (film) ν 3457 (broad), 2953, 2869, 1467, 1136 cm^{-1}.
$[\alpha]_D^{21}$ –10.89 (c = 1.13, $CHCl_3$).
Chiral SFC: CHIRALPAK IC-3, 5% *i*-PrOH/CO_2, 3.5 mL/min, 220 nm, t_{R1} (minor) = 2.5 min, t_{R2} (major) = 3.3 min.

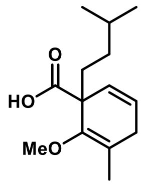

S28

Carboxylic acid (S28). Prepared according to **general procedure for Birch reduction/alkylation of carboxylic acid**, using 2-methoxy-3-methylbenzoic acid (200 mg, 1.2 mmol, 1.0 equiv.) and 1-iodo-3-methylbutane (0.48 mL, 3.6 mmol, 3.0 equiv.) to give **S28** as a colorless oil (69 mg, 24% yield).
^{1}H NMR (500 MHz, CDCl3) δ 5.84 (dt, J = 9.9, 3.3 Hz, 1H), 5.50 (dt, J = 9.9, 2.0 Hz, 1H), 3.70 (s, 3H), 2.83 – 2.67 (m, 2H), 1.97 (td, J = 12.9, 5.0 Hz, 1H), 1.76 (s, 3H), 1.74 – 1.67 (m, 1H), 1.51 (dh, J = 12.7, 6.4 Hz, 1H), 1.12 – 0.97 (m, 2H), 0.88 (d, J = 6.4 Hz, 6H).
^{13}C{^{1}H} NMR (126 MHz, $CDCl_3$) δ 177.7, 147.0, 127.9, 126.2, 118.4, 61.3, 52.8, 33.5, 33.4, 32.7, 28.5, 22.9, 22.8, 16.4.
HRMS (ES+) m/z calc'd for $C_{14}H_{22}O_3Na$ $[M + Na]^+$: 261.1467, found 261.1460.
IR (film) ν 3300-2500 (broad), 2954, 2869, 1693, 752 cm^{-1}.

IR and NMR Spectra

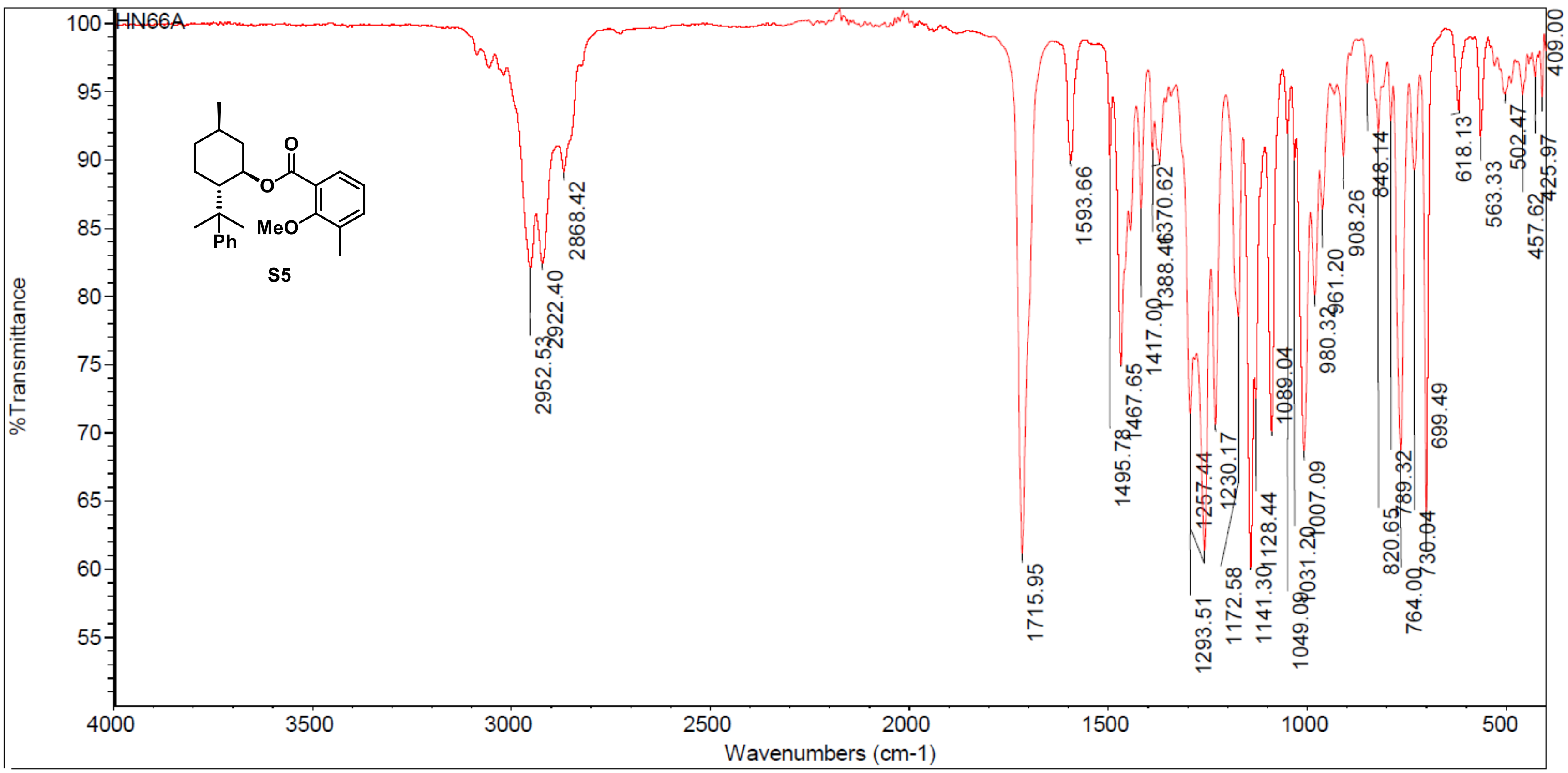
HN66A
MeO
O
O
Ph
S5
%Transmittance
Wavenumbers (cm-1)
2952.53
2922.40
2868.42
1715.95
1593.66
1495.78
1467.65
1417.00
1388.46
1370.62
1293.51
1257.44
1230.17
1172.58
1141.30
1128.44
1089.04
1049.09
1031.20
1007.09
980.32
961.20
908.26
848.14
820.65
789.32
764.00
730.04
699.49
618.13
563.33
502.47
457.62
425.97
409.00

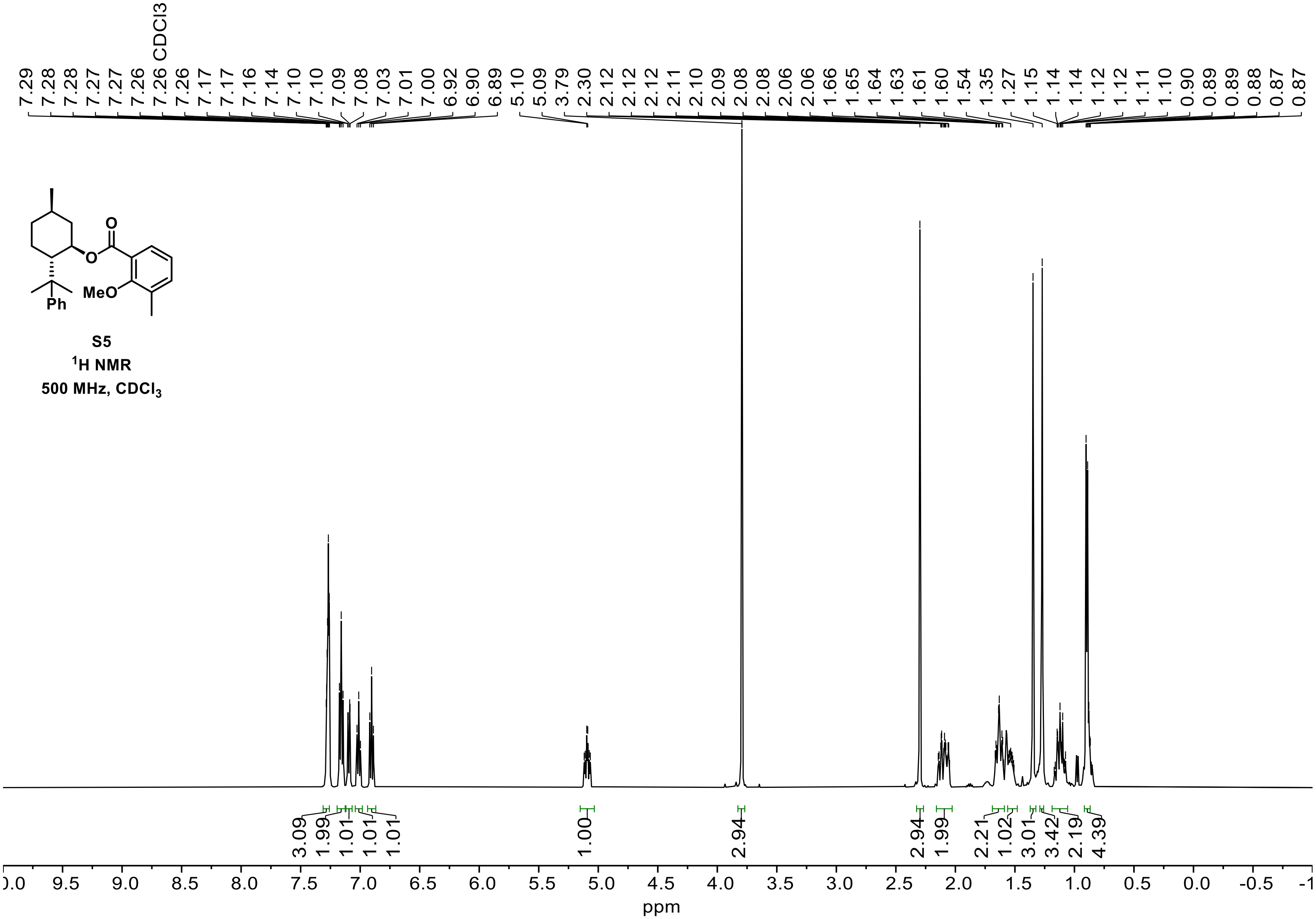

CDCl3
7.29 7.28 7.28 7.27 7.27 7.26 7.26 7.26 7.17 7.17 7.16 7.14 7.10 7.10 7.09 7.08 7.03 7.01 7.00 6.92 6.90 6.89 5.10 5.09 3.79 2.30 2.12 2.12 2.12 2.11 2.10 2.09 2.08 2.08 2.06 2.06 1.66 1.65 1.64 1.63 1.61 1.60 1.54 1.35 1.27 1.15 1.14 1.14 1.12 1.12 1.11 1.10 0.90 0.89 0.89 0.88 0.87 0.87
O
O
MeO
Ph
S5
1H NMR
500 MHz, CDCl3
3.09 1.99 1.01 1.01 1.01 1.00 2.94 2.94 1.99 2.21 1.02 3.01 3.42 2.19 4.39
0.0 9.5 9.0 8.5 8.0 7.5 7.0 6.5 6.0 5.5 5.0 4.5 4.0 3.5 3.0 2.5 2.0 1.5 1.0 0.5 0.0 -0.5 -1
ppm

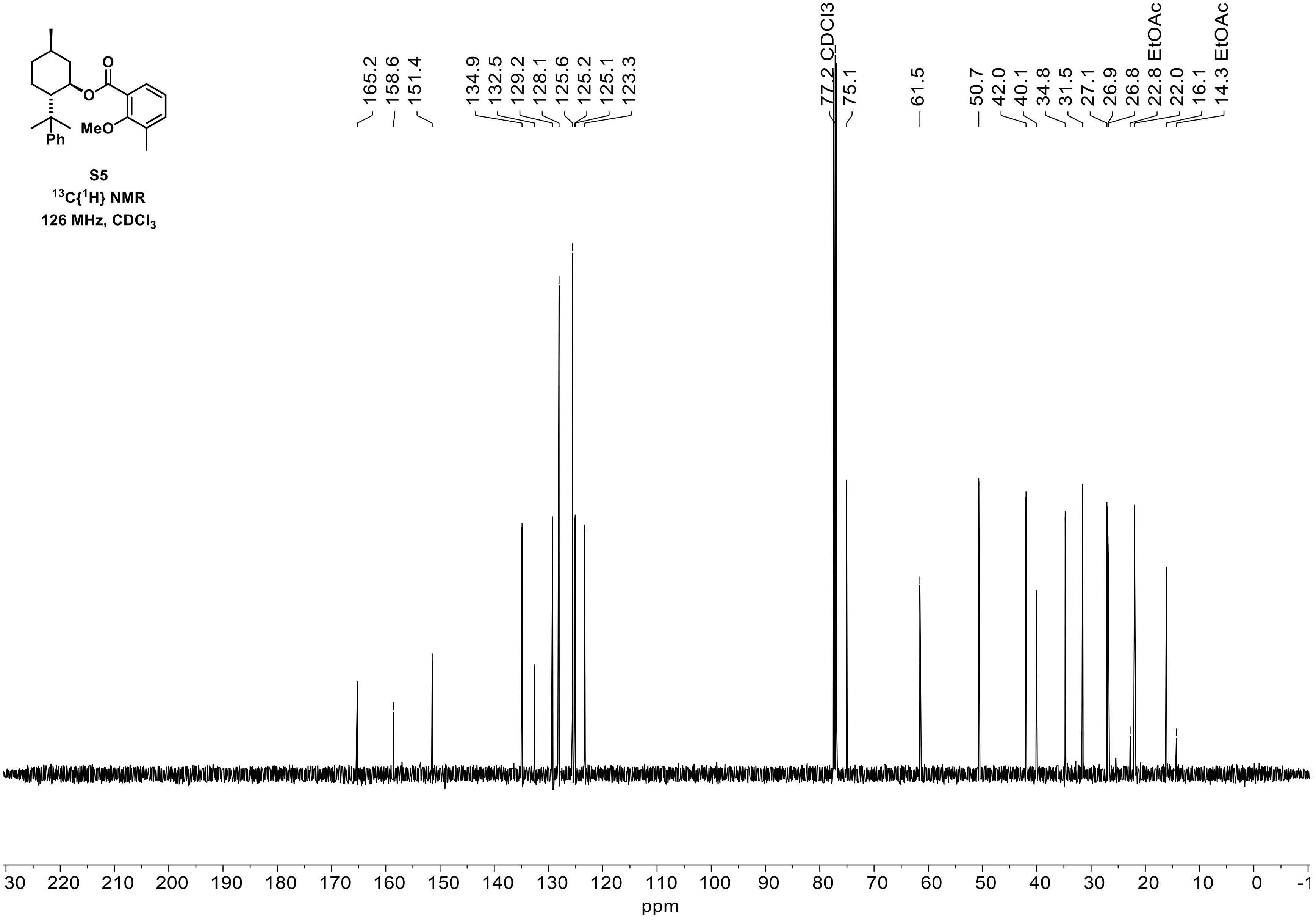
MeO
Ph
S5
$^{13}C\{^1H\}$ NMR
126 MHz, $CDCl_3$
165.2
158.6
151.4
134.9
132.5
129.2
128.1
125.6
125.2
125.1
123.3
77.2 CDCl3
75.1
61.5
50.7
42.0
40.1
34.8
31.5
27.1
26.9
26.8
22.8 EtOAc
22.0
16.1
14.3 EtOAc
ppm

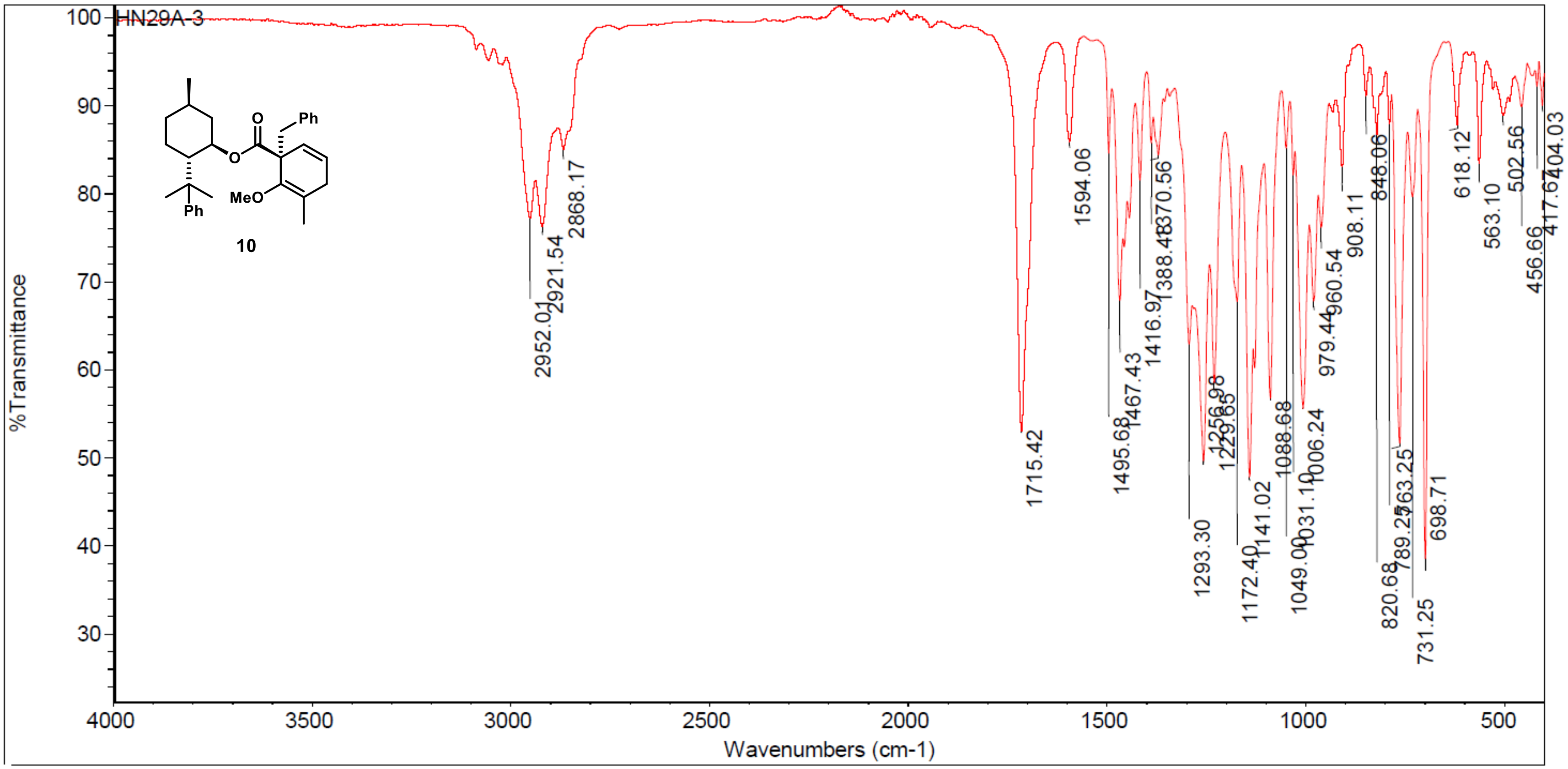
HN29A-3
10
%Transmittance
Wavenumbers (cm-1)
2952.01
2921.54
2868.17
1715.42
1594.06
1495.68
1467.43
1416.97
1388.48
1370.56
1293.30
1256.98
1229.65
1172.40
1141.02
1088.68
1049.00
1031.10
1006.24
979.44
960.54
908.11
848.06
820.68
789.25
763.25
731.25
698.71
618.12
563.10
502.56
456.66
417.64
404.03

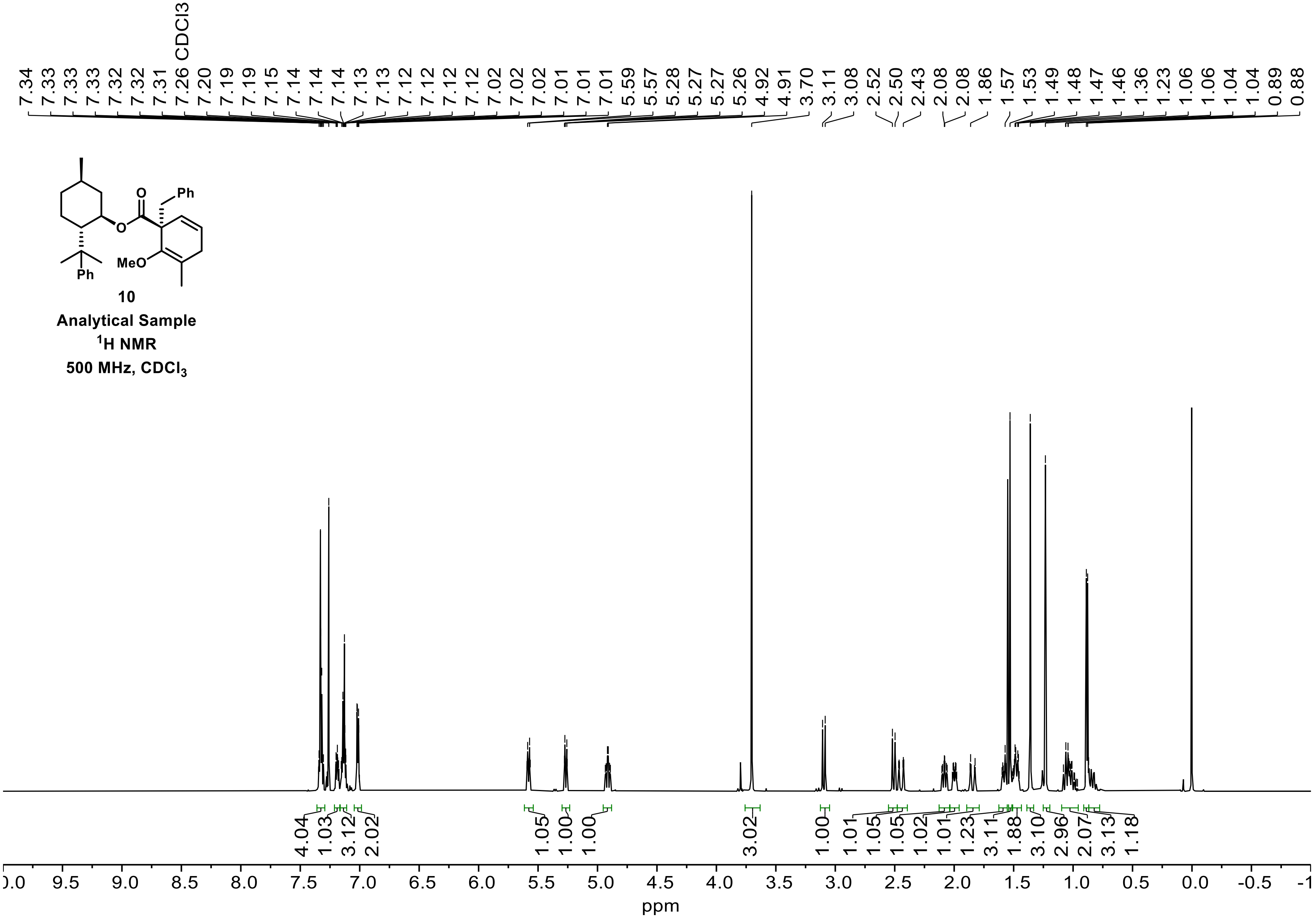
10
Analytical Sample
1H NMR
500 MHz, CDCl3
ppm

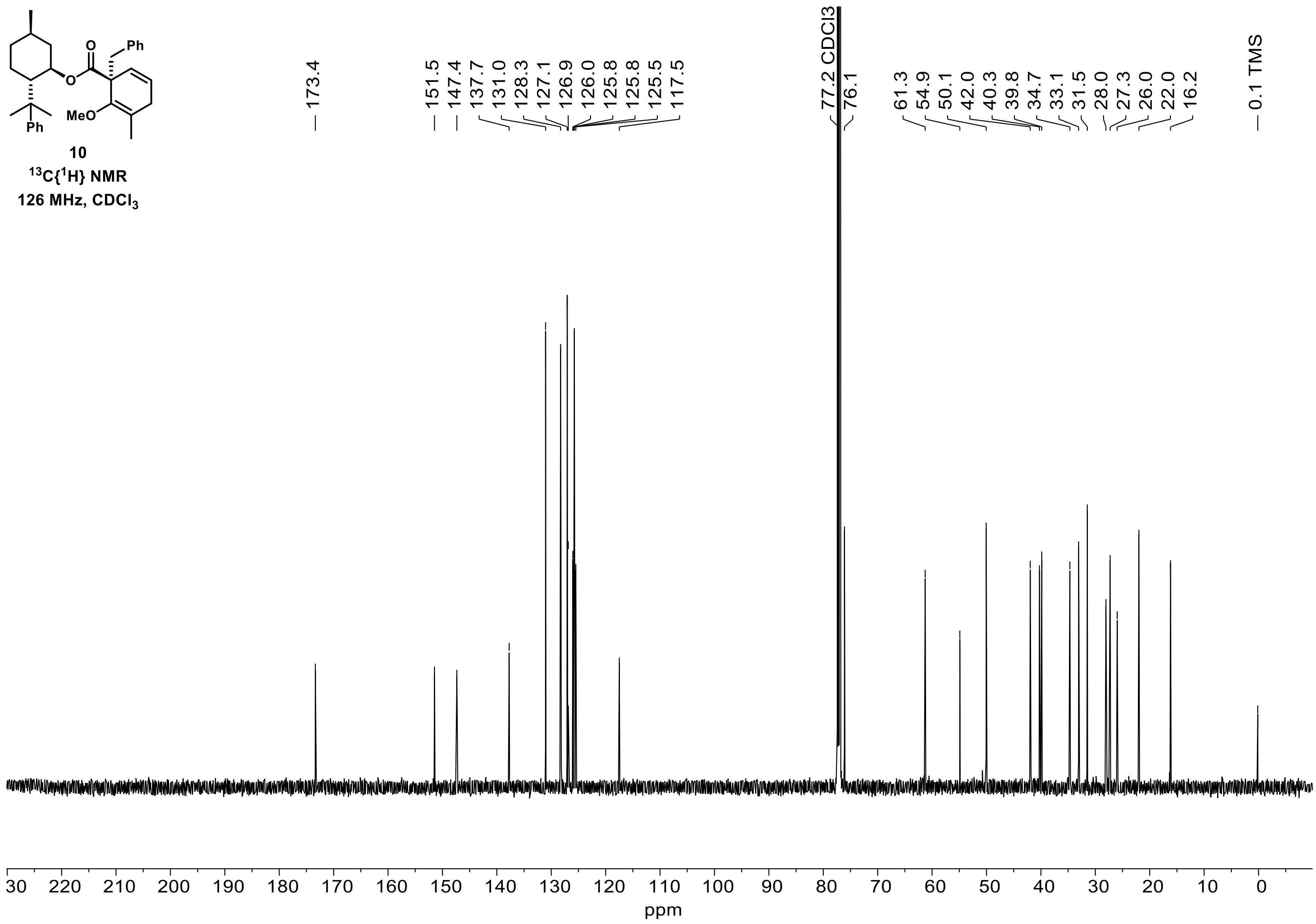

Ph
O
O
MeO
Ph
10
$^{13}C\{^1H\}$ NMR
126 MHz, $CDCl_3$
173.4
151.5
147.4
137.7
131.0
128.3
127.1
126.9
126.0
125.8
125.8
125.5
117.5
77.2 CDCl3
76.1
61.3
54.9
50.1
42.0
40.3
39.8
34.7
33.1
31.5
28.0
27.3
26.0
22.0
16.2
0.1 TMS
230 220 210 200 190 180 170 160 150 140 130 120 110 100 90 80 70 60 50 40 30 20 10 0
ppm

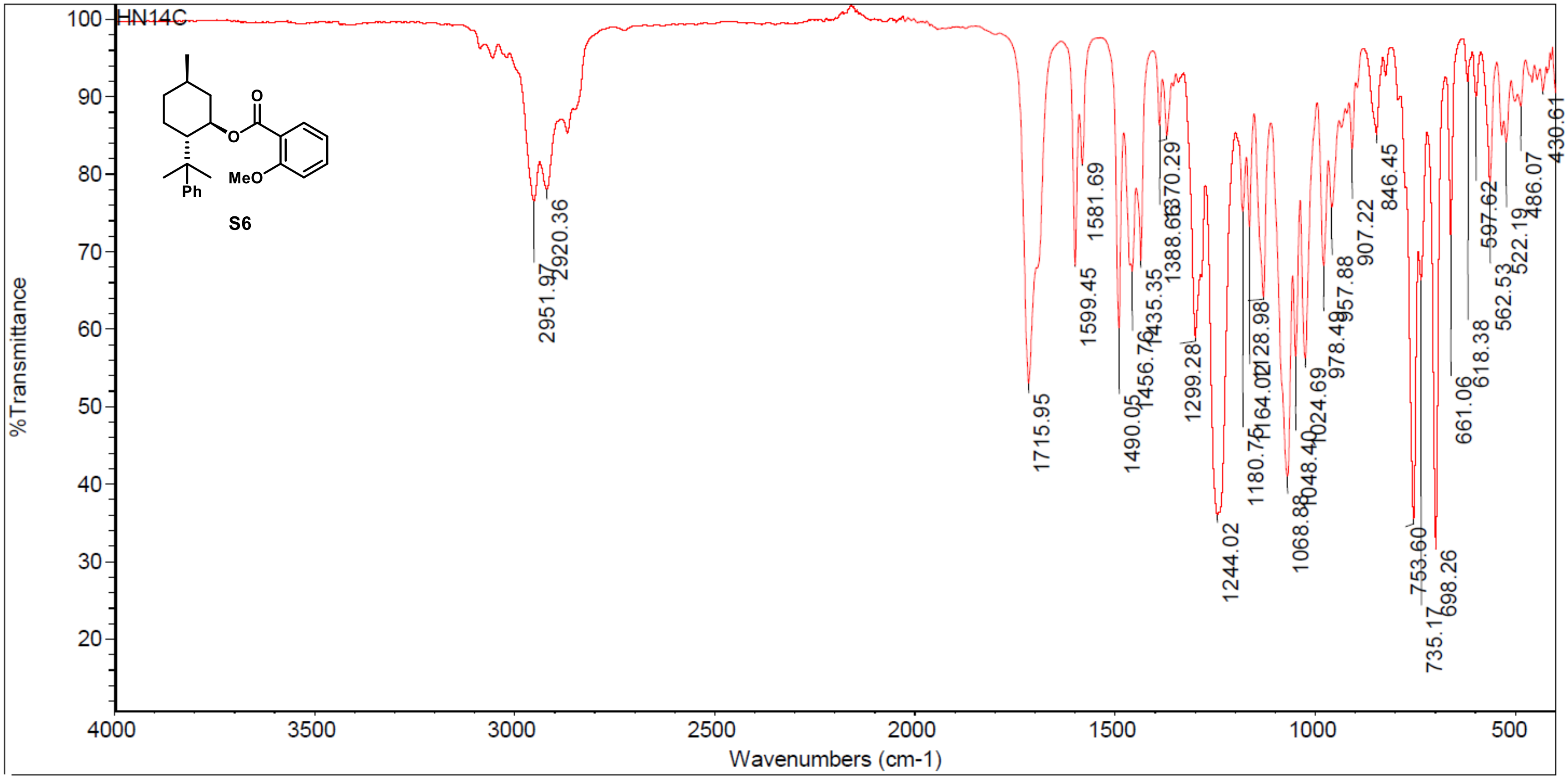
HN14C
%Transmittance
Wavenumbers (cm-1)
MeO
Ph
S6
2951.97
2920.36
1715.95
1599.45
1581.69
1490.05
1456.76
1435.35
1388.66
1370.29
1299.28
1244.02
1180.75
1164.02
1128.98
1068.88
1048.40
1024.69
978.49
957.88
907.22
846.45
753.60
735.17
698.26
661.06
618.38
597.62
562.53
522.19
486.07
430.61

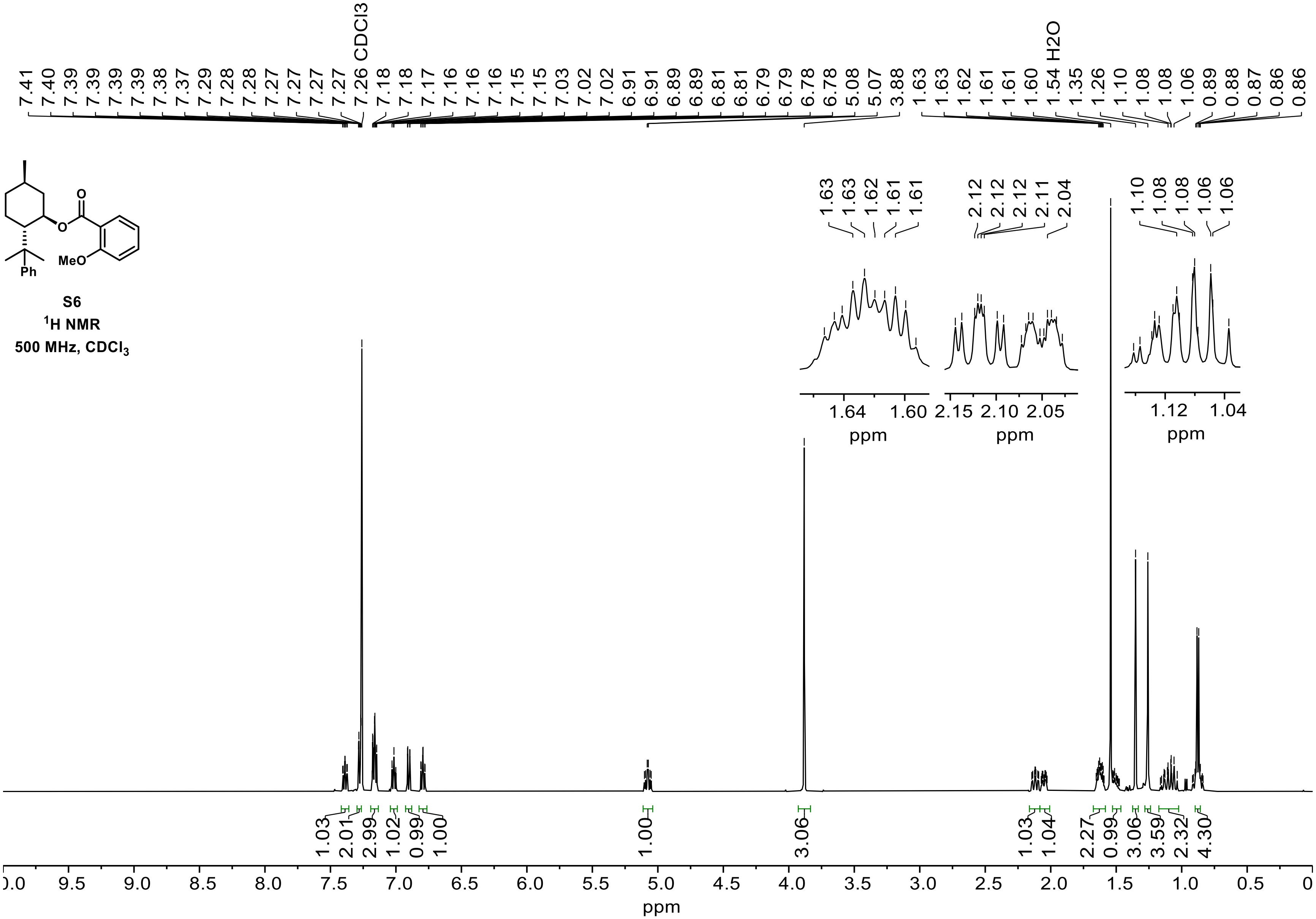

S6
^{1}H NMR
500 MHz, $CDCl_3$
MeO
Ph
CDCl3
H2O
ppm

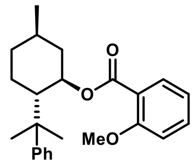

S6
$^{13}C\{^1H\}$ NMR
126 MHz, $CDCl_3$

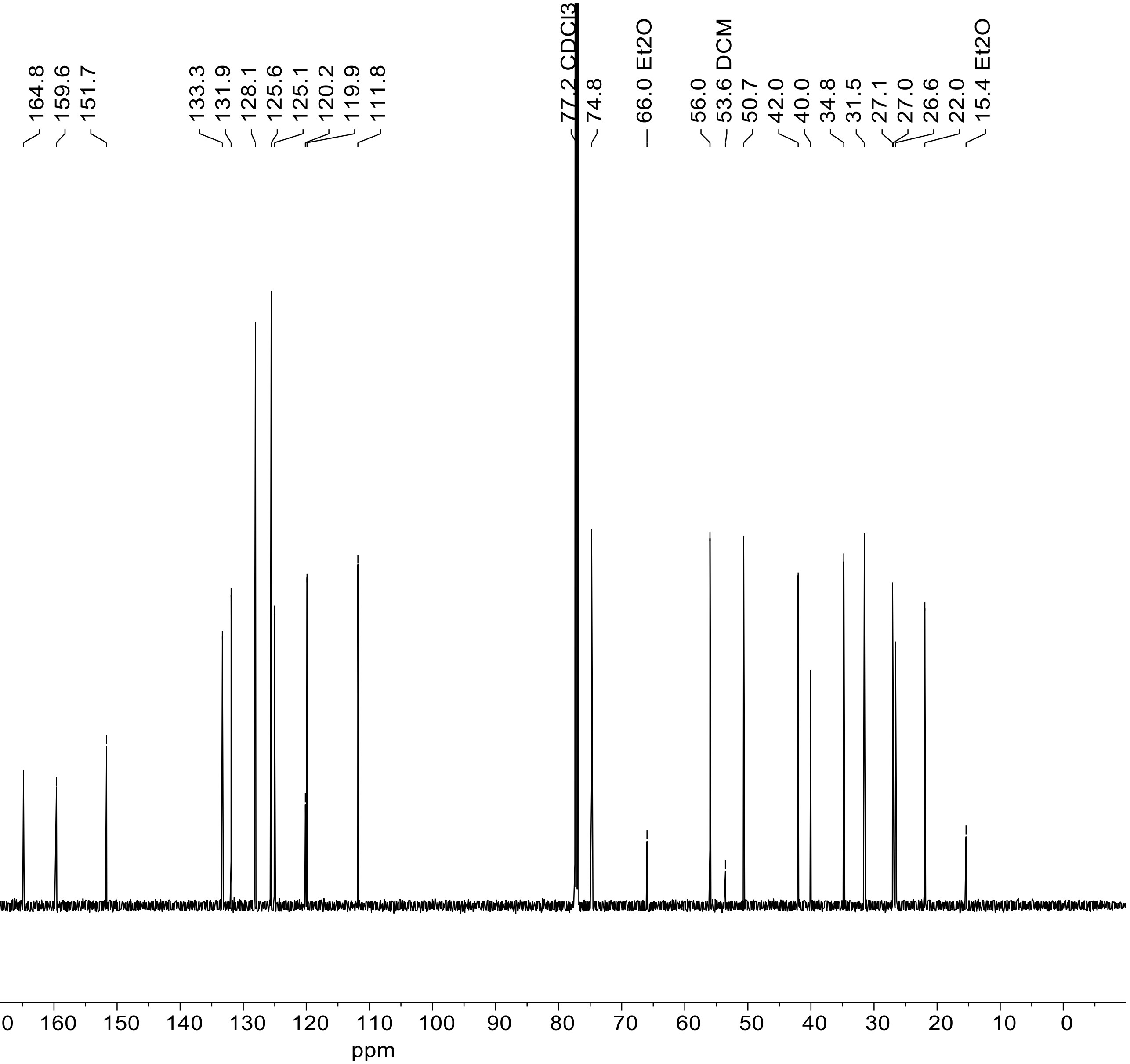

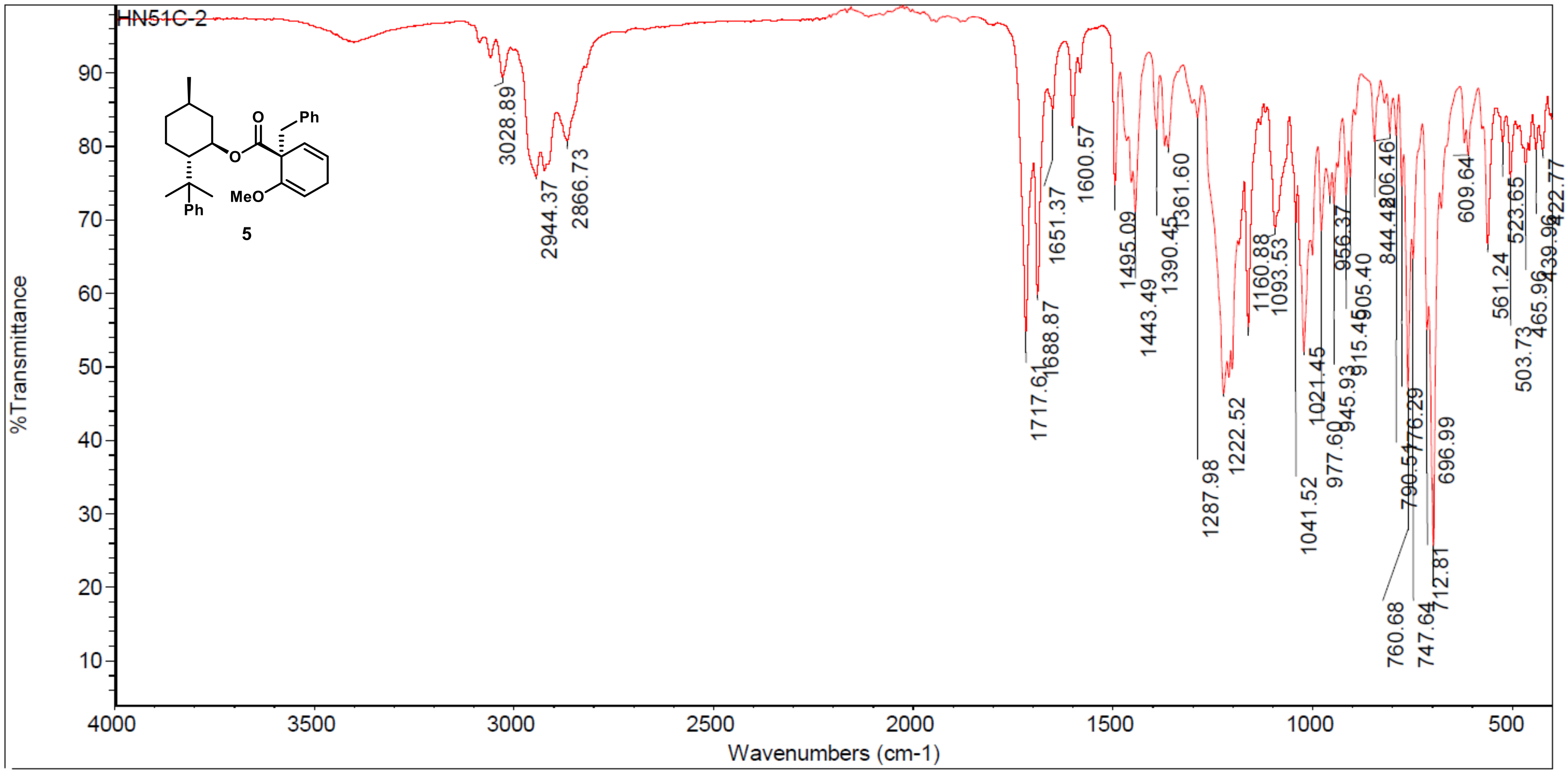
HN51C-2
%Transmittance
Wavenumbers (cm-1)
3028.89
2944.37
2866.73
1717.61
1688.87
1651.37
1600.57
1495.09
1443.49
1390.45
1361.60
1287.98
1222.52
1160.88
1093.53
1041.52
1021.45
977.60
956.37
945.93
915.45
905.40
844.48
806.46
790.54
776.29
760.68
747.64
712.81
696.99
609.64
561.24
523.65
503.73
465.96
439.96
422.77
Ph
O
O
MeO
5

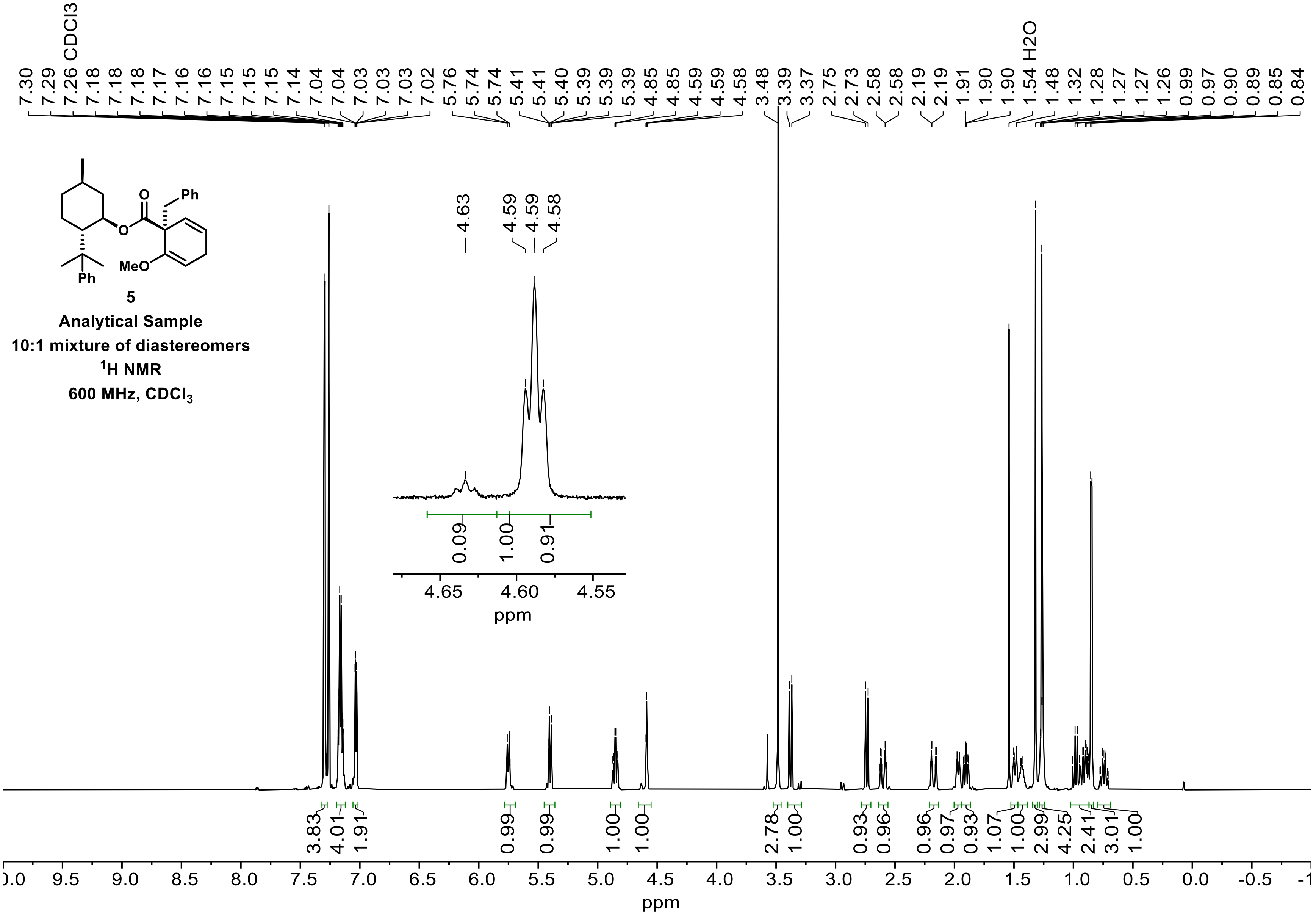

5

Analytical Sample

10:1 mixture of diastereomers

^{1}H NMR

600 MHz, $CDCl_3$

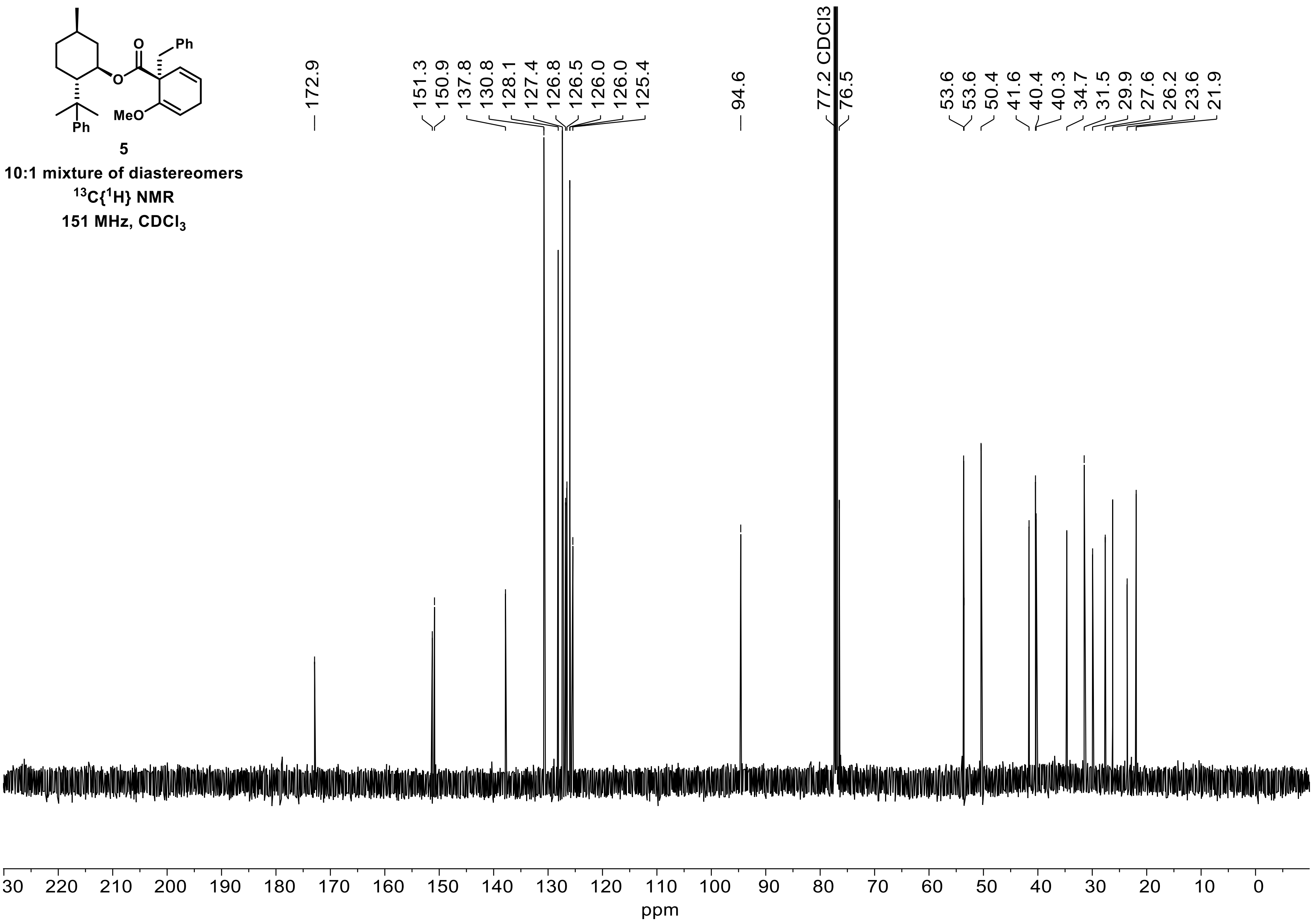
Ph
O
O
MeO
Ph
5
10:1 mixture of diastereomers
13C{1H} NMR
151 MHz, CDCl3
172.9
151.3
150.9
137.8
130.8
128.1
127.4
126.8
126.5
126.0
126.0
125.4
94.6
77.2 CDCl3
76.5
53.6
53.6
50.4
41.6
40.4
40.3
34.7
31.5
29.9
27.6
26.2
23.6
21.9
ppm

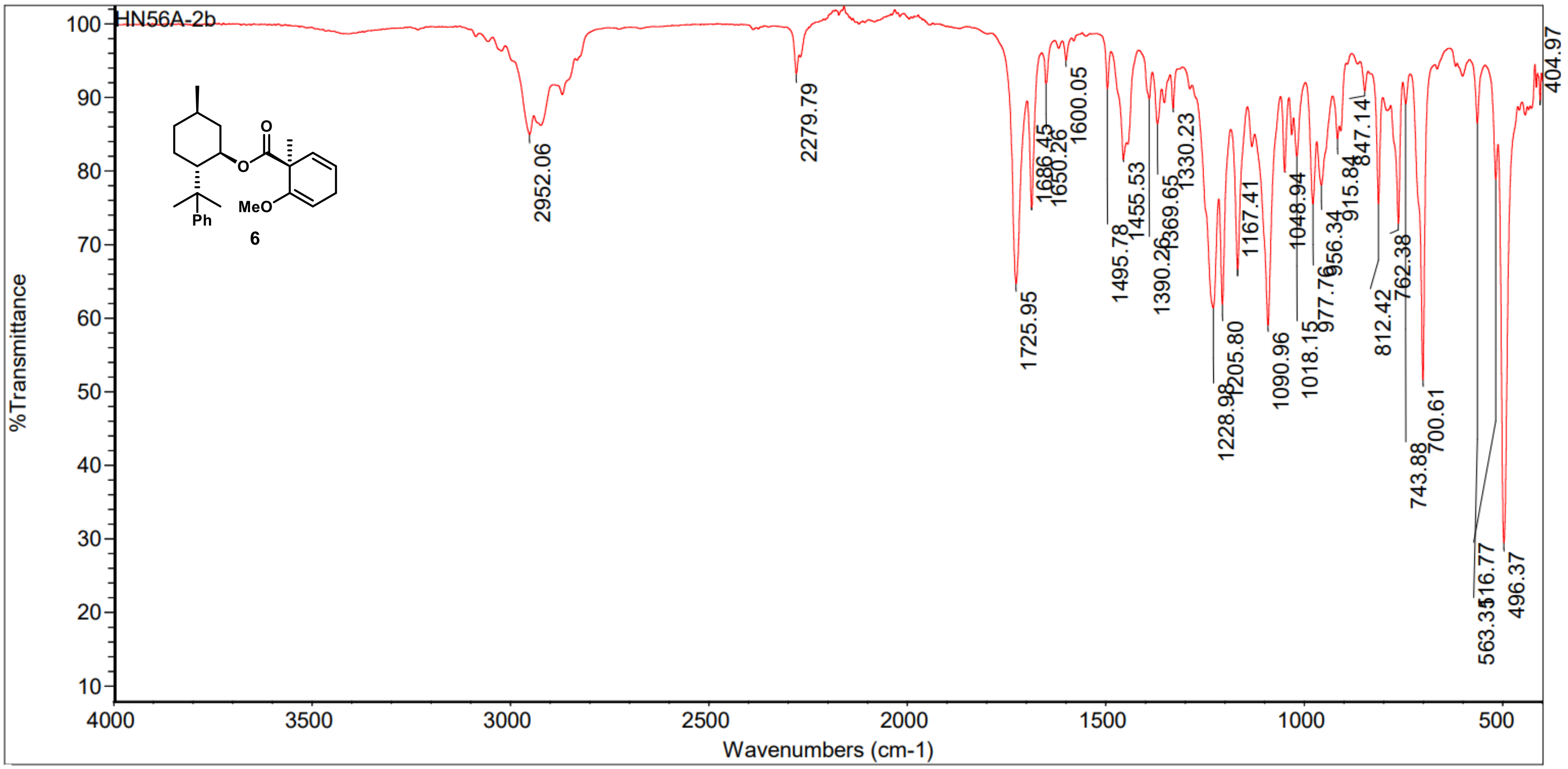
HN56A-2b
%Transmittance
Wavenumbers (cm-1)
MeO
Ph
O
6
2952.06
2279.79
1725.95
1686.45
1650.26
1600.05
1495.78
1455.53
1390.26
1369.65
1330.23
1228.98
1205.80
1167.41
1090.96
1048.94
1018.15
977.76
956.34
915.84
847.14
812.42
762.38
743.88
700.61
563.35
516.77
496.37
404.97

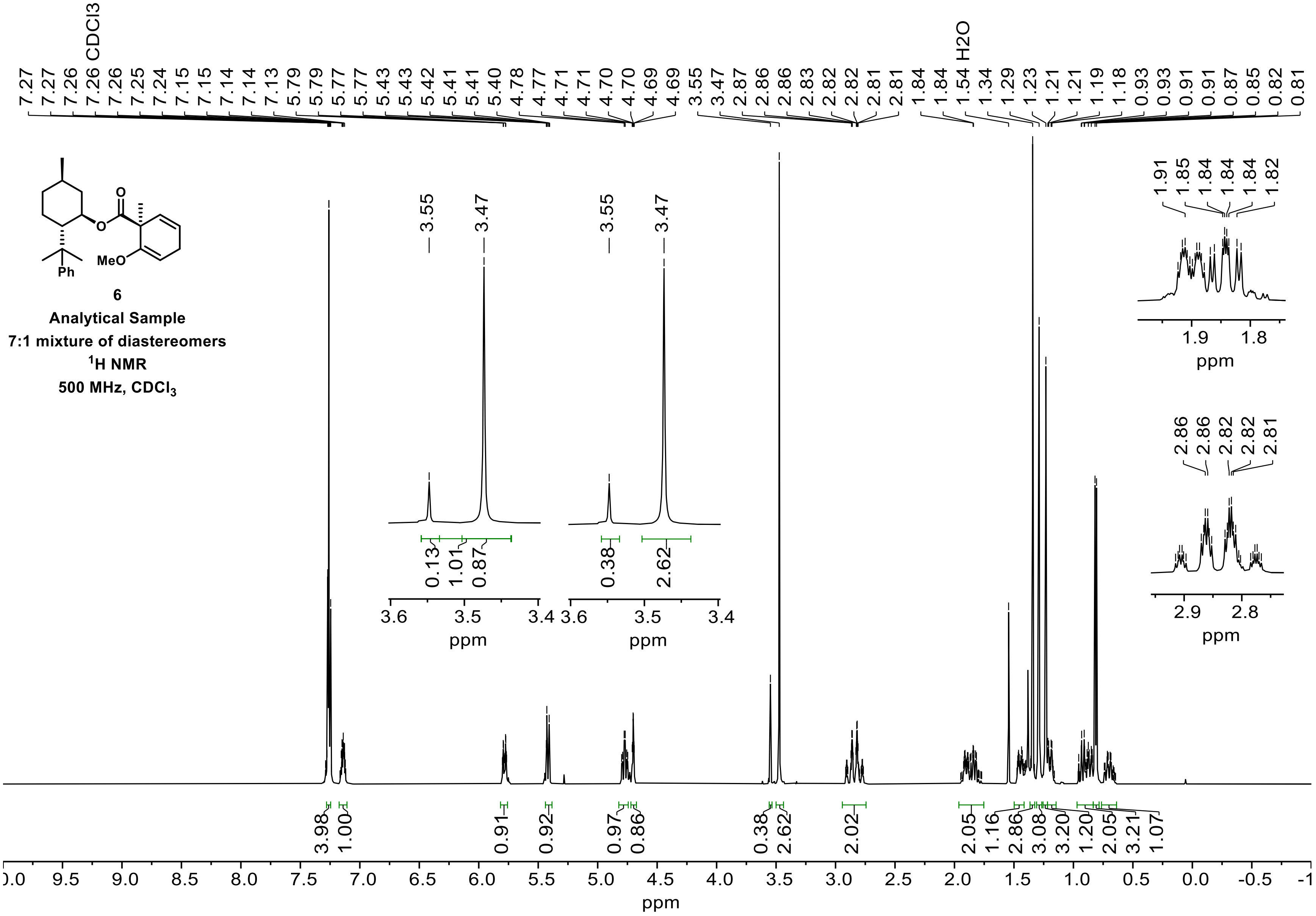

6
Analytical Sample
7:1 mixture of diastereomers
^{1}H NMR
500 MHz, $CDCl_3$

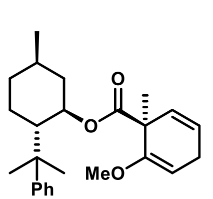

6

7:1 mixture of diastereomers

$^{13}C\{^{1}H\}$ NMR

151 MHz, $CDCl_3$

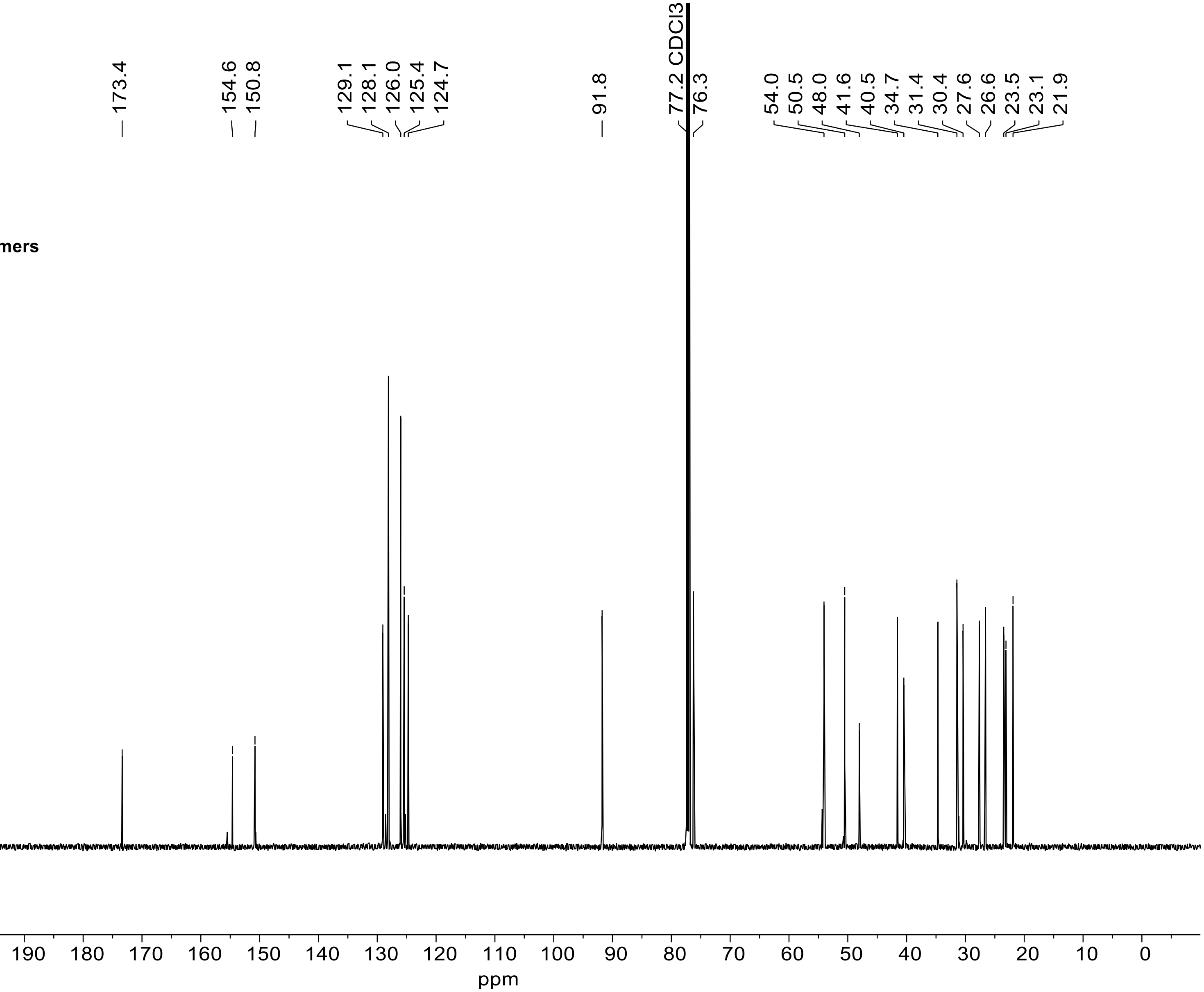

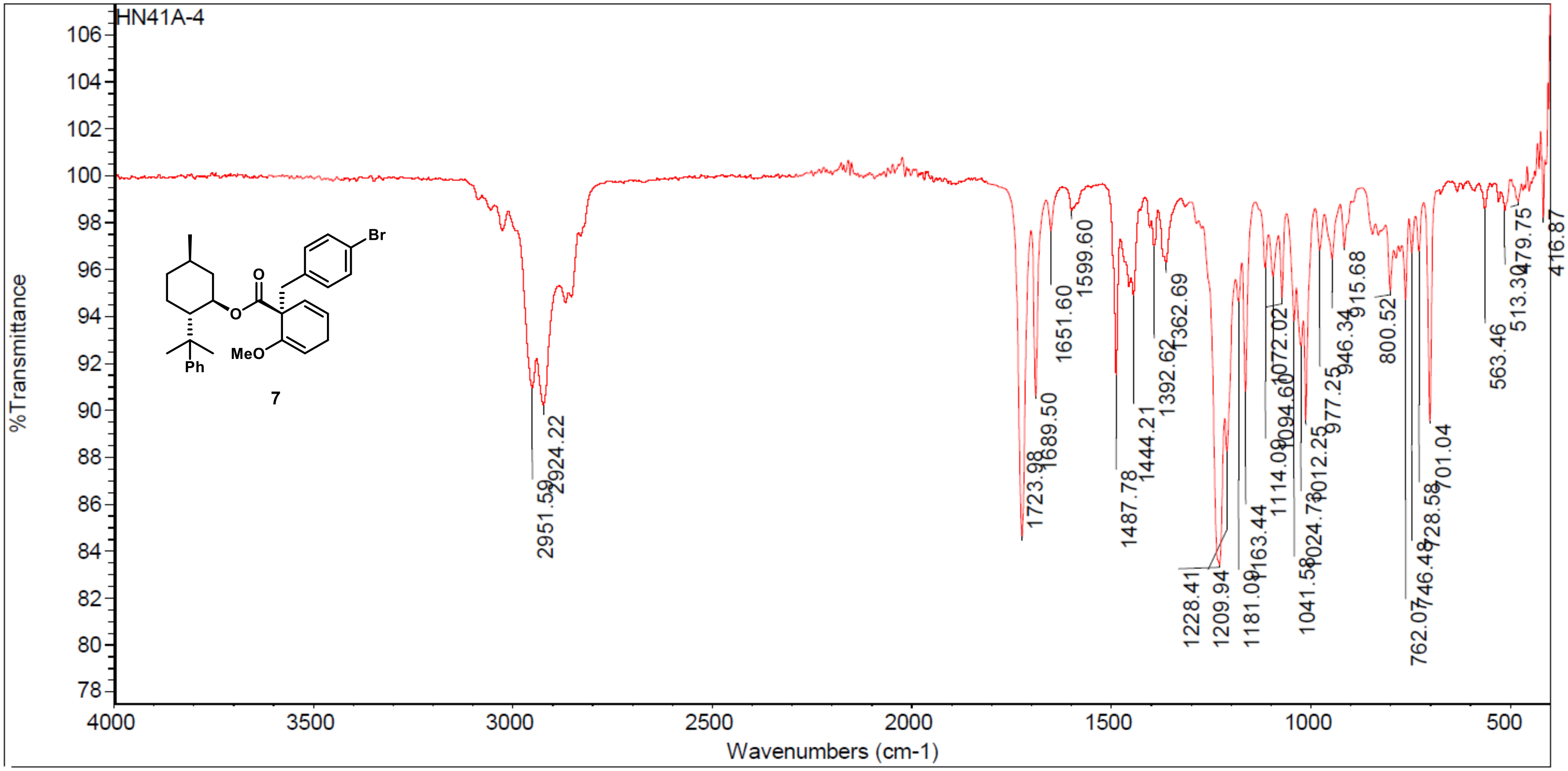
HN41A-4
%Transmittance
Wavenumbers (cm-1)
Br
O
O
MeO
Ph
7
2951.59
2924.22
1723.98
1689.50
1651.60
1599.60
1487.78
1444.21
1392.62
1362.69
1228.41
1209.94
1181.09
1163.44
1114.09
1094.60
1072.02
1041.58
1024.73
1012.25
977.25
946.34
915.68
800.52
762.07
746.48
728.58
701.04
563.46
513.30
479.75
416.87

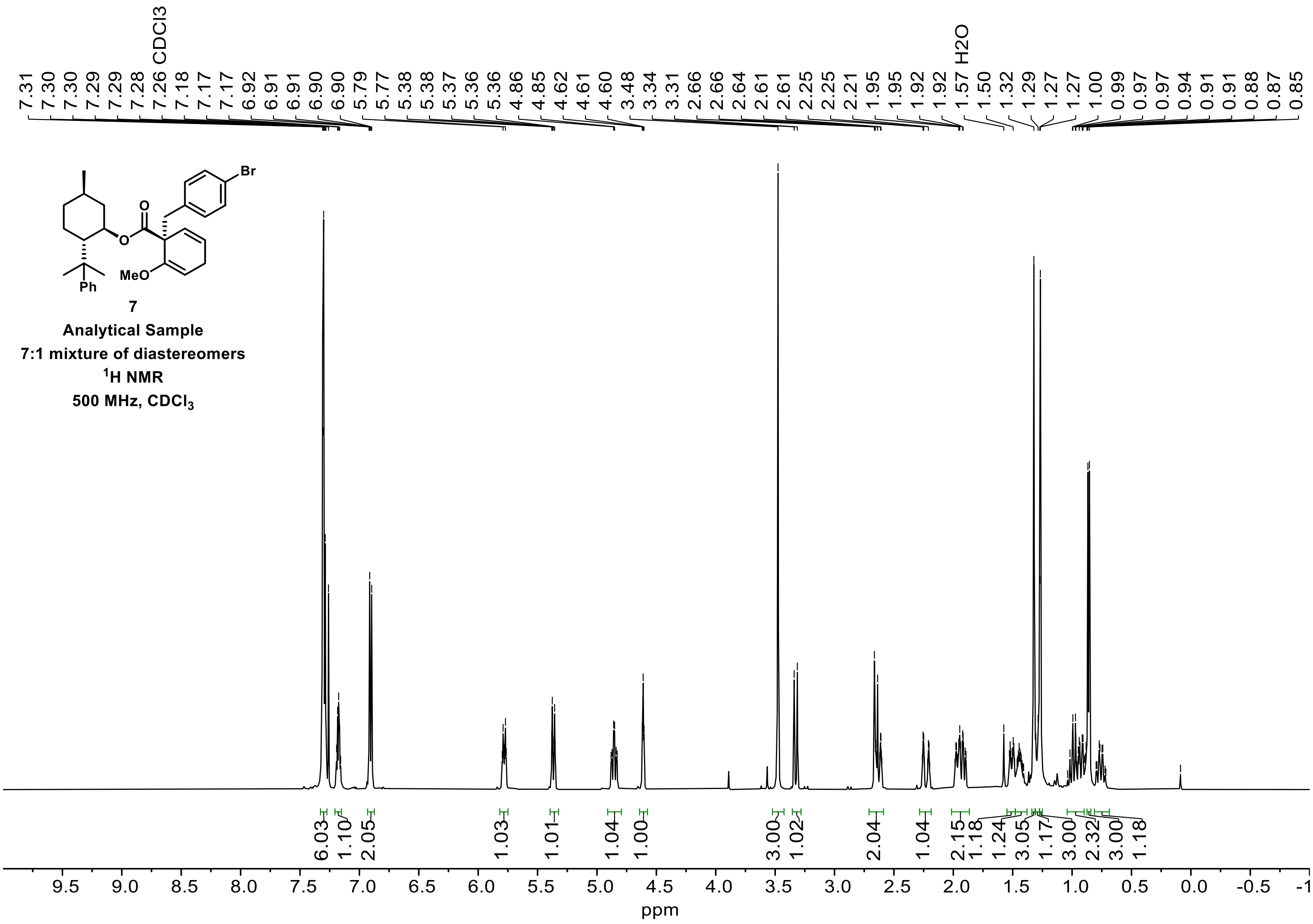

Br
O
O
MeO
Ph
7
Analytical Sample
7:1 mixture of diastereomers
1H NMR
500 MHz, CDCl3
CDCl3
H2O
ppm

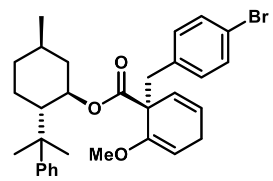

7

7:1 mixture of diastereomers

$^{13}C\{^1H\}$ NMR

126 MHz, $CDCl_3$

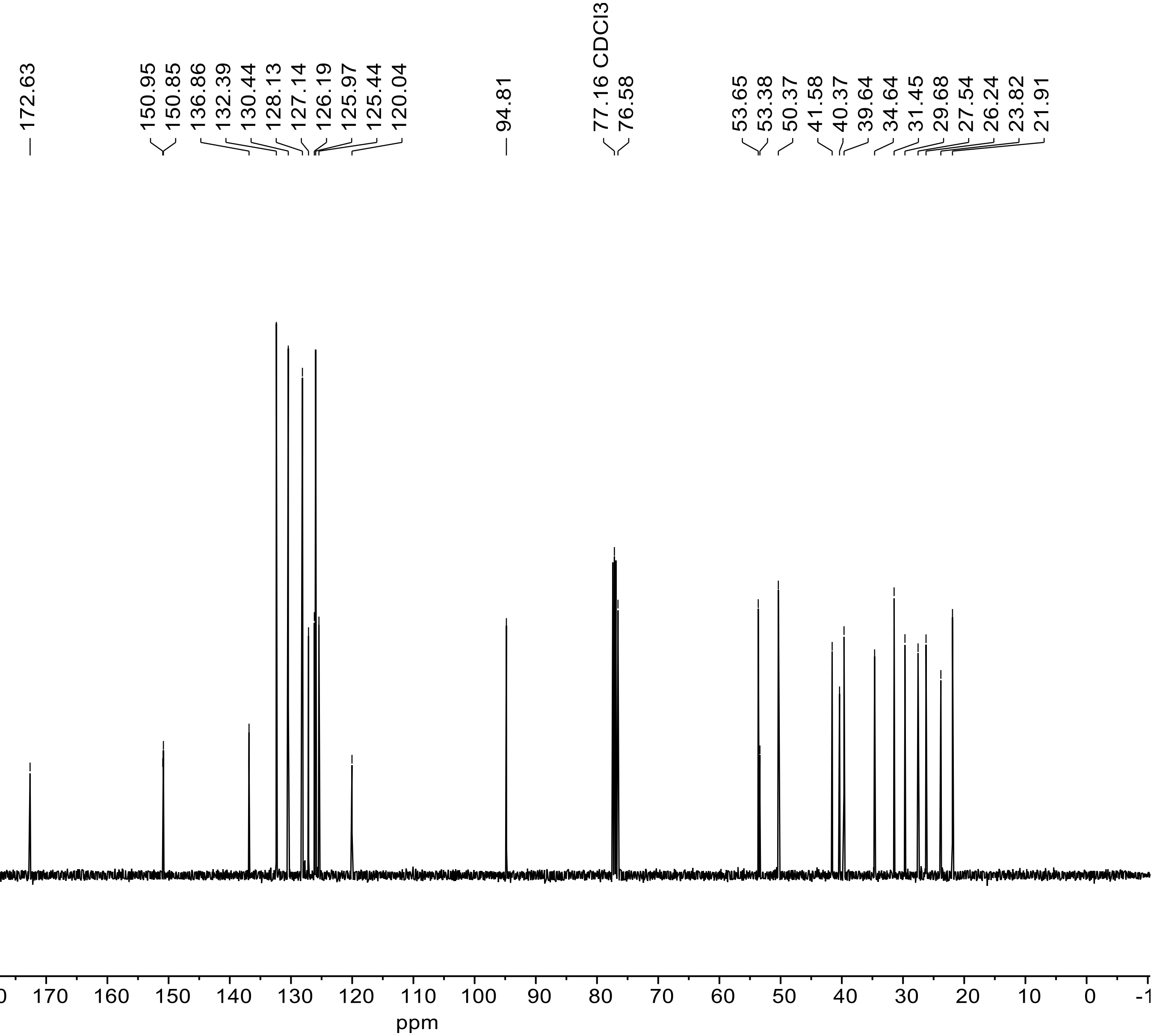

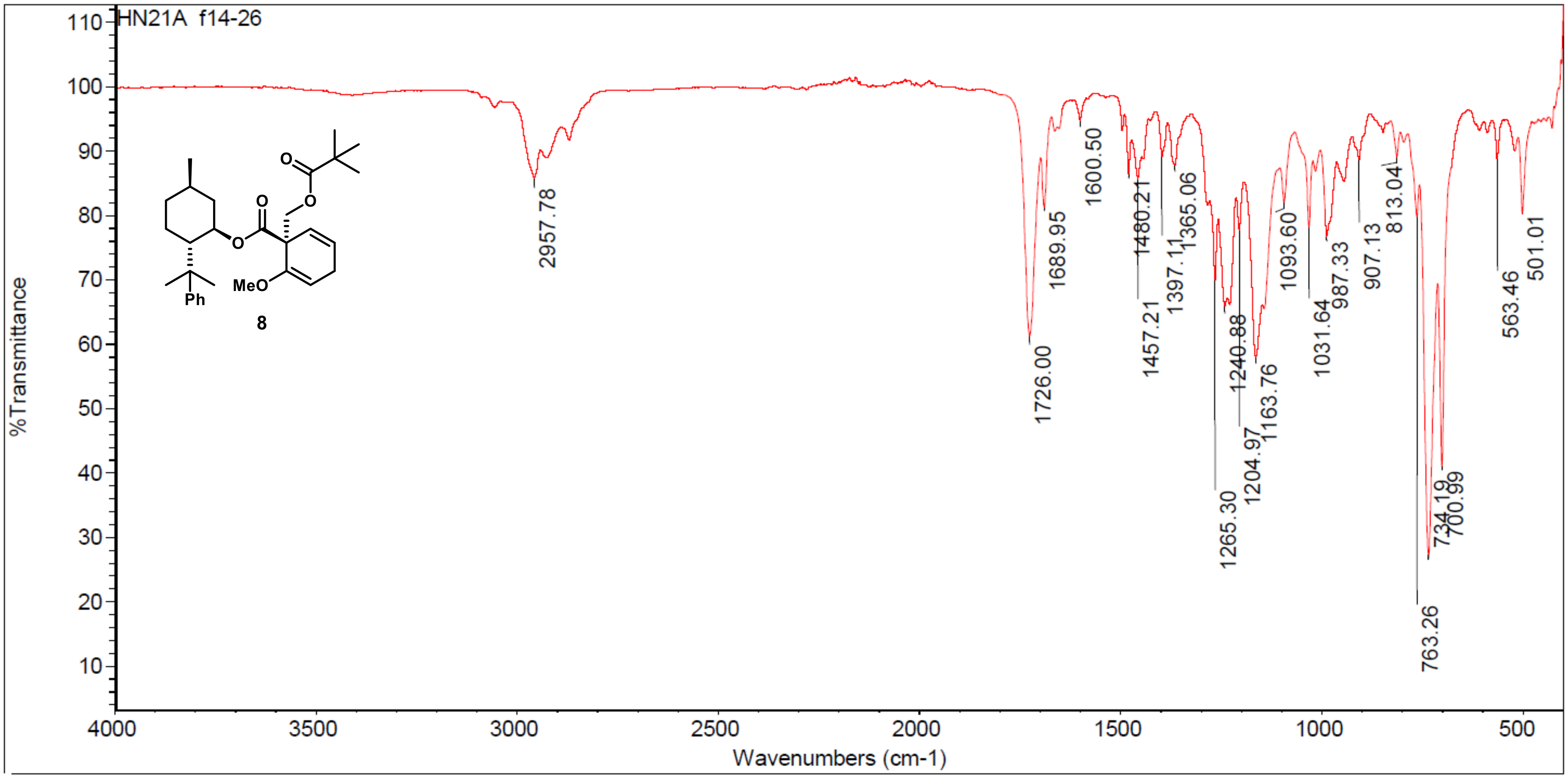
HN21A f14-26
%Transmittance
Wavenumbers (cm-1)
2957.78
1726.00
1689.95
1600.50
1480.21
1457.21
1397.11
1365.06
1265.30
1240.88
1204.97
1163.76
1093.60
1031.64
987.33
907.13
813.04
763.26
734.19
700.99
563.46
501.01
MeO
Ph
O
8

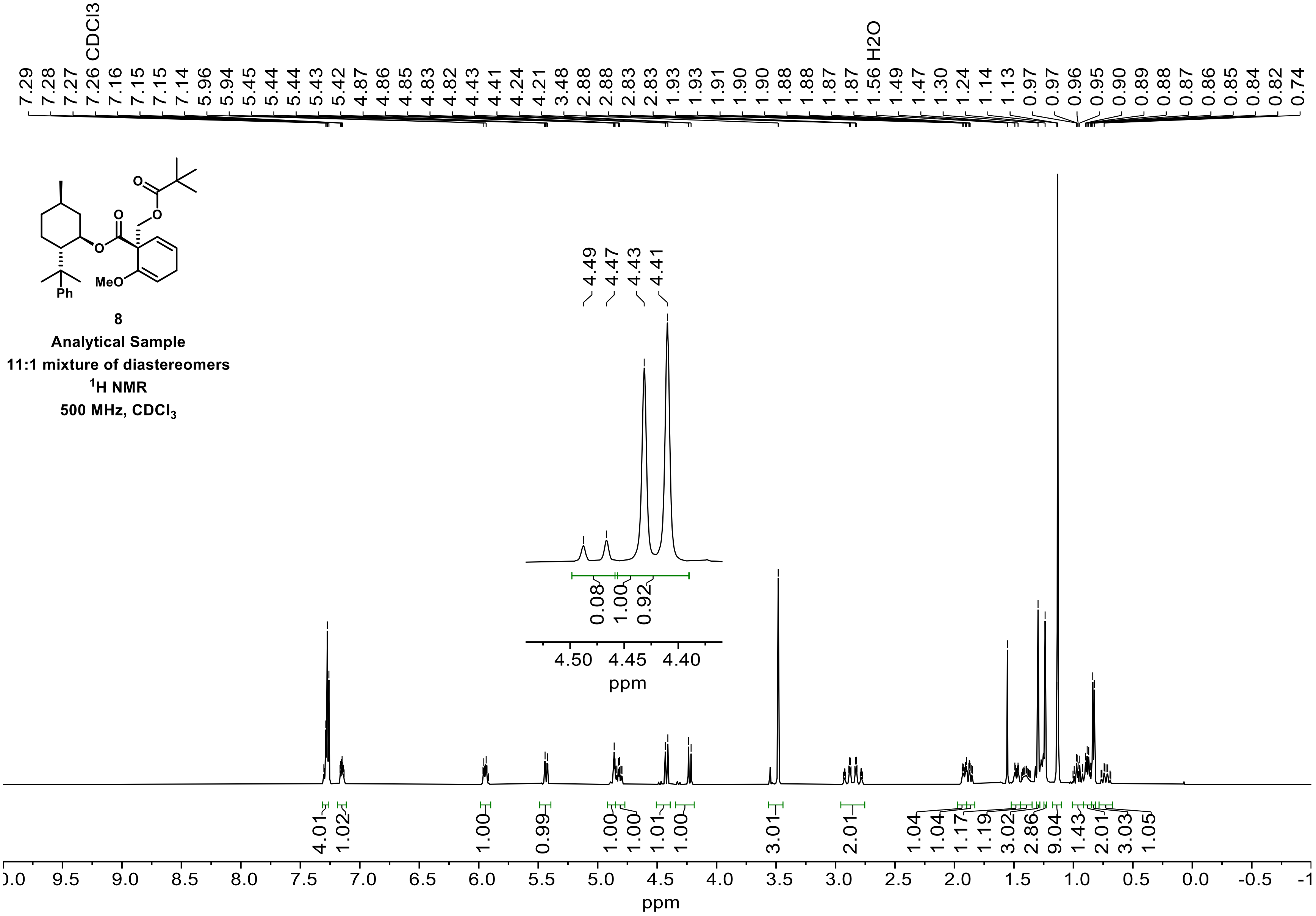
8
Analytical Sample
11:1 mixture of diastereomers
1H NMR
500 MHz, CDCl3
MeO
Ph
CDCl3
H2O
ppm

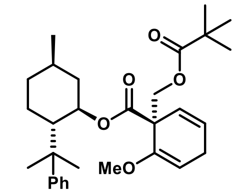

8

11:1 mixture of diastereomers

$^{13}C\{^{1}H\}$ NMR

126 MHz, $CDCl_3$

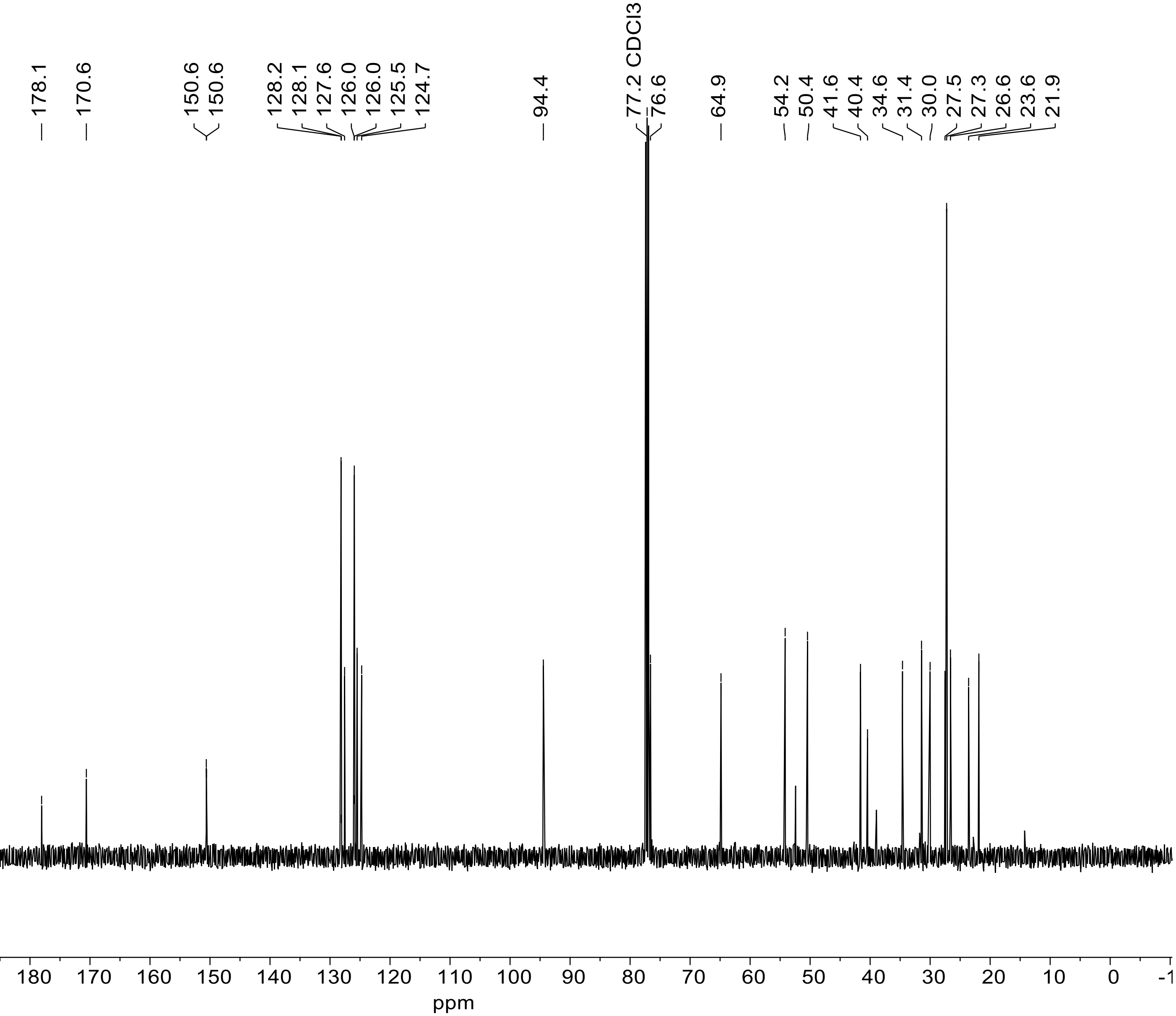

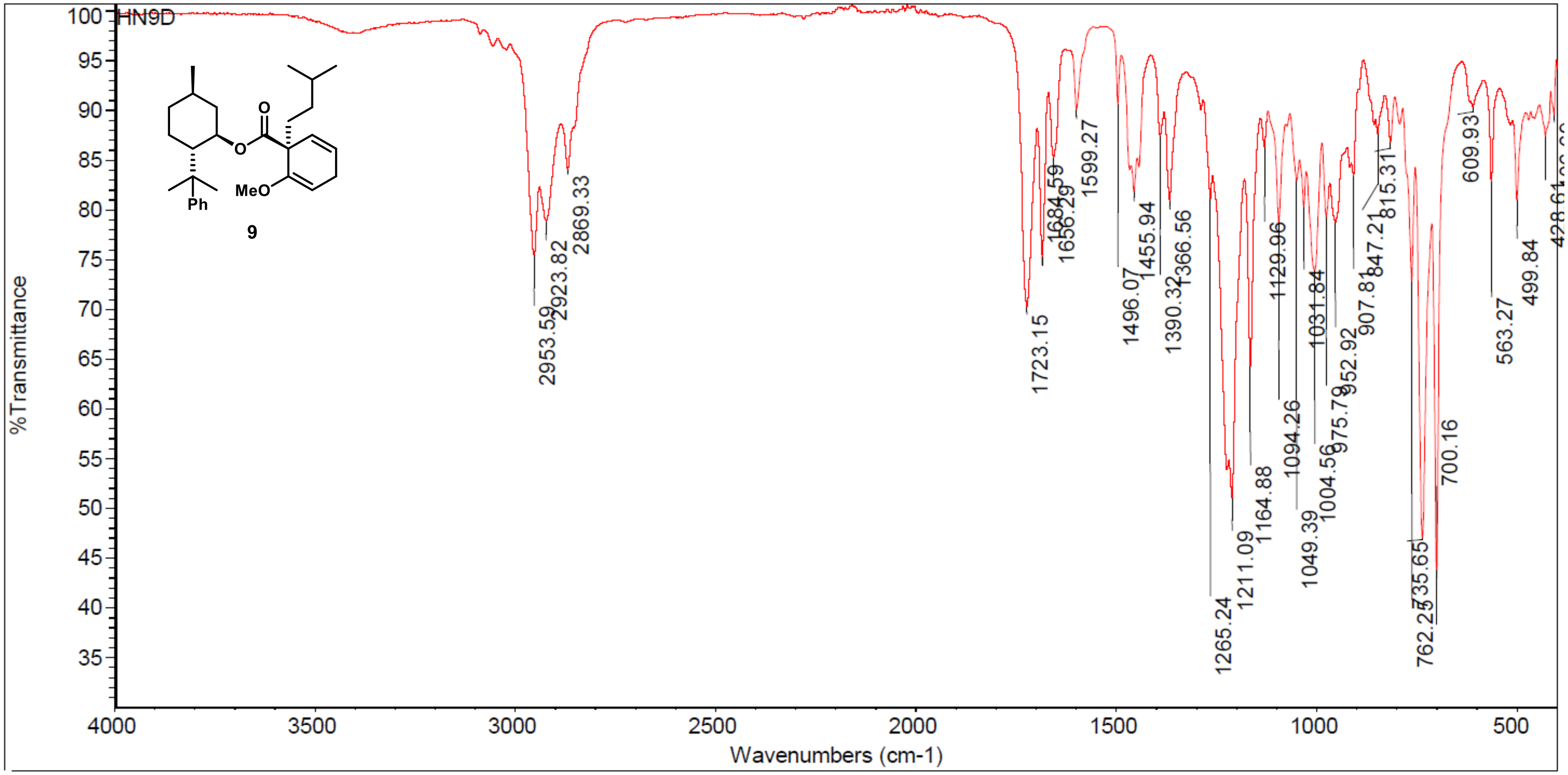
HN9D
%Transmittance
Wavenumbers (cm-1)
MeO
Ph
O
9
2953.59
2923.82
2869.33
1723.15
1684.59
1656.29
1599.27
1496.07
1455.94
1390.32
1366.56
1265.24
1211.09
1164.88
1129.96
1094.26
1049.39
1031.84
1004.56
975.79
952.92
907.81
847.21
815.31
762.25
735.65
700.16
609.93
563.27
499.84

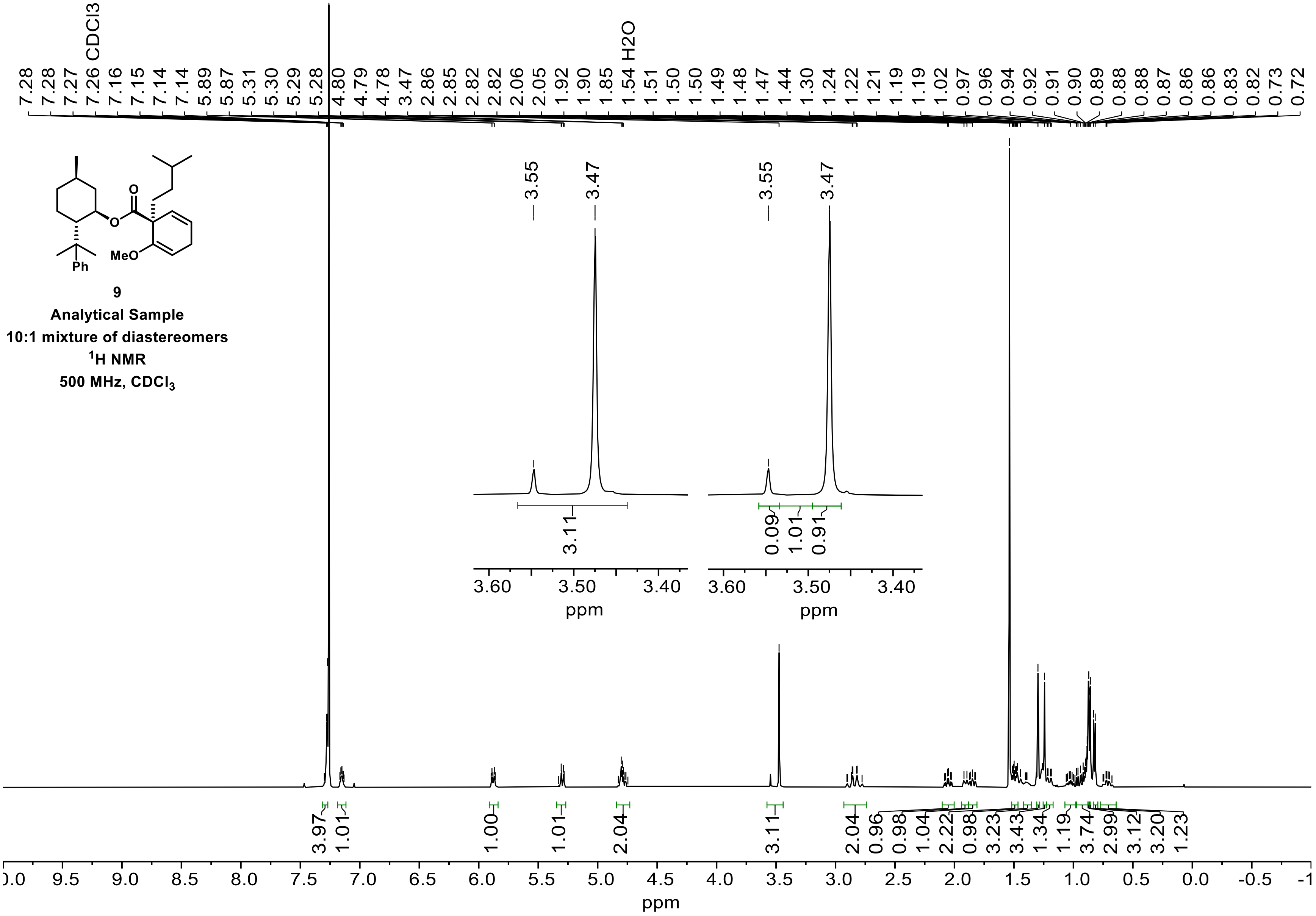
7.28 7.28 7.27 7.26 CDCl3 7.16 7.15 7.14 7.14 5.89 5.87 5.31 5.30 5.29 5.28 4.80 4.79 4.78 3.47 2.86 2.85 2.82 2.82 2.06 2.05 1.92 1.90 1.85 1.54 H2O 1.51 1.50 1.50 1.49 1.48 1.47 1.44 1.30 1.24 1.22 1.21 1.19 1.19 1.02 0.97 0.96 0.94 0.92 0.91 0.90 0.89 0.88 0.88 0.87 0.86 0.86 0.83 0.82 0.73 0.72
MeO
Ph
O
9
Analytical Sample
10:1 mixture of diastereomers
1H NMR
500 MHz, CDCl3
3.55
3.47
3.11
0.09
1.01
0.91
3.60 3.50 3.40
ppm
3.97 1.01 1.00 1.01 2.04 3.11 2.04 0.96 0.98 1.04 2.22 0.98 3.23 3.43 1.34 1.19 3.74 2.99 3.12 3.20 1.23
9.5 9.0 8.5 8.0 7.5 7.0 6.5 6.0 5.5 5.0 4.5 4.0 3.5 3.0 2.5 2.0 1.5 1.0 0.5 0.0 -0.5
ppm

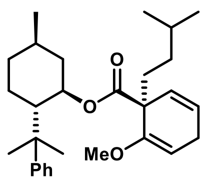

9
10:1 mixture of diastereomers
$^{13}C\{^{1}H\}$ NMR
126 MHz, $CDCl_3$

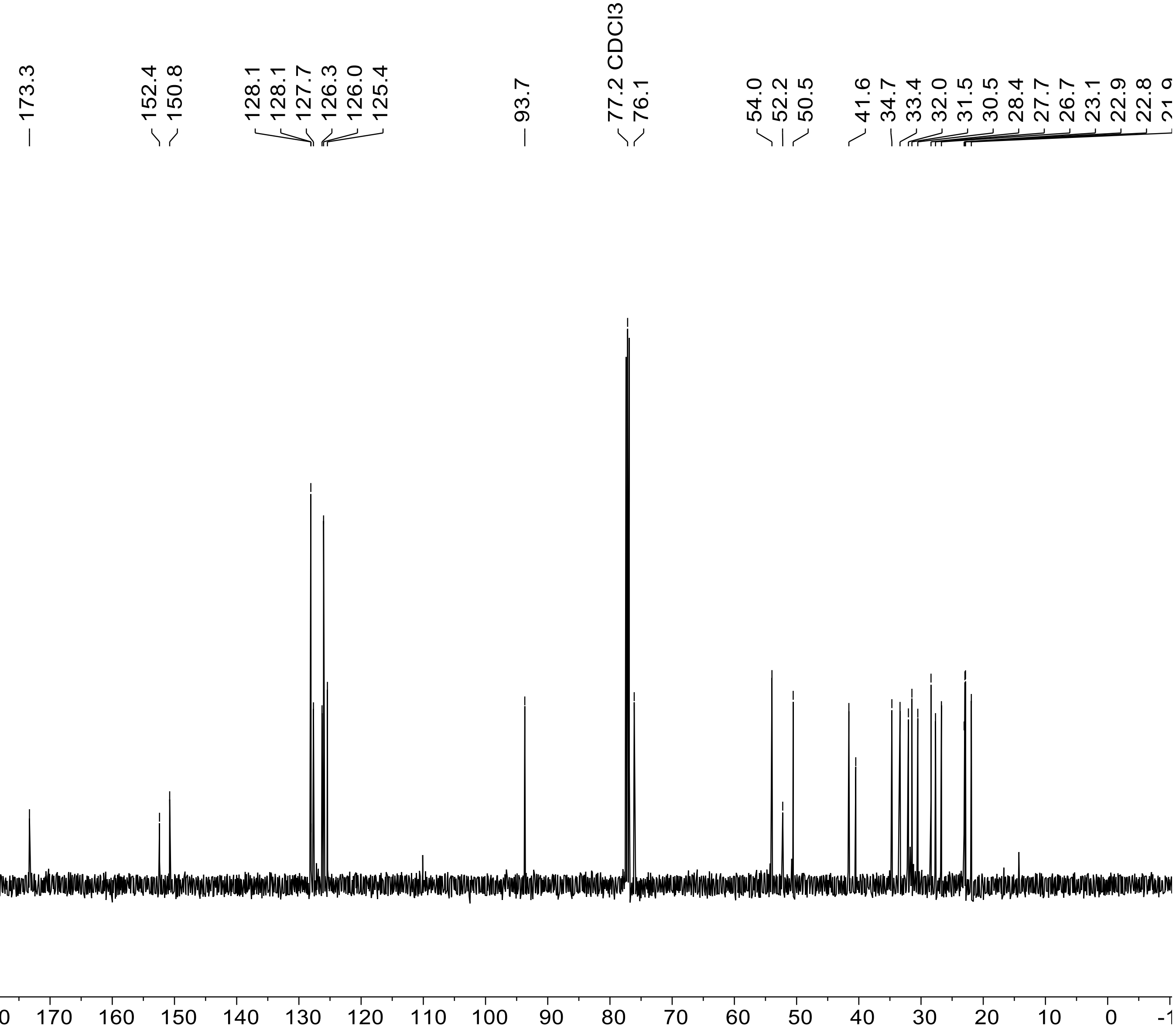

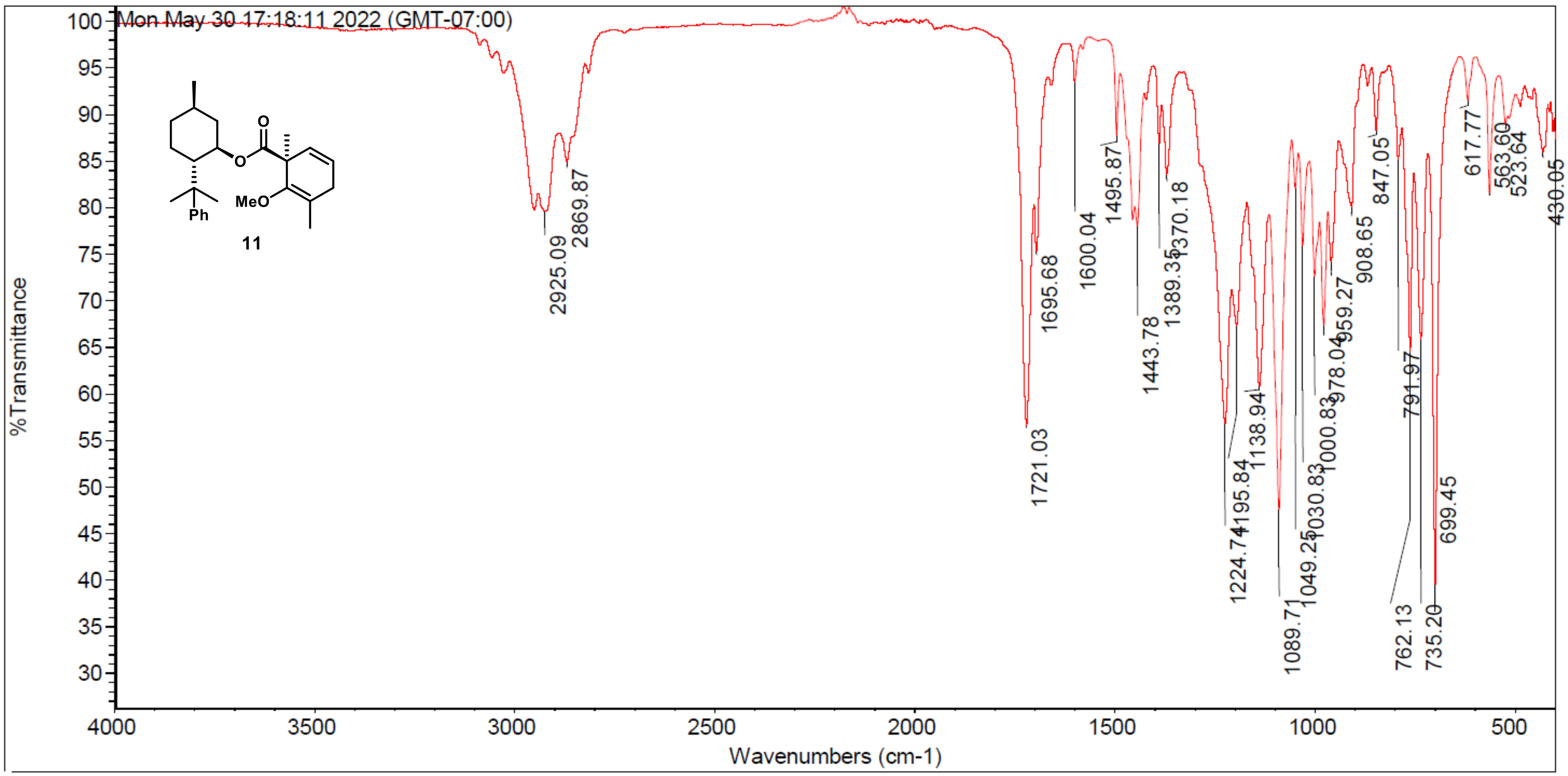

Mon May 30 17:18:11 2022 (GMT-07:00)
MeO
Ph
O
11
%Transmittance
Wavenumbers (cm-1)
2925.09
2869.87
1721.03
1695.68
1600.04
1495.87
1443.78
1389.35
1370.18
1224.74
1195.84
1138.94
1089.71
1049.25
1030.83
1000.83
978.04
959.27
908.65
847.05
791.97
762.13
735.20
699.45
617.77
563.60
523.64
430.05

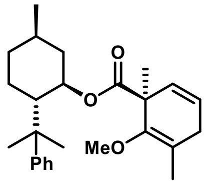

11
Analytical Sample
4:1 mixture of diastereomers
^{1}H NMR
600 MHz, $CDCl_3$

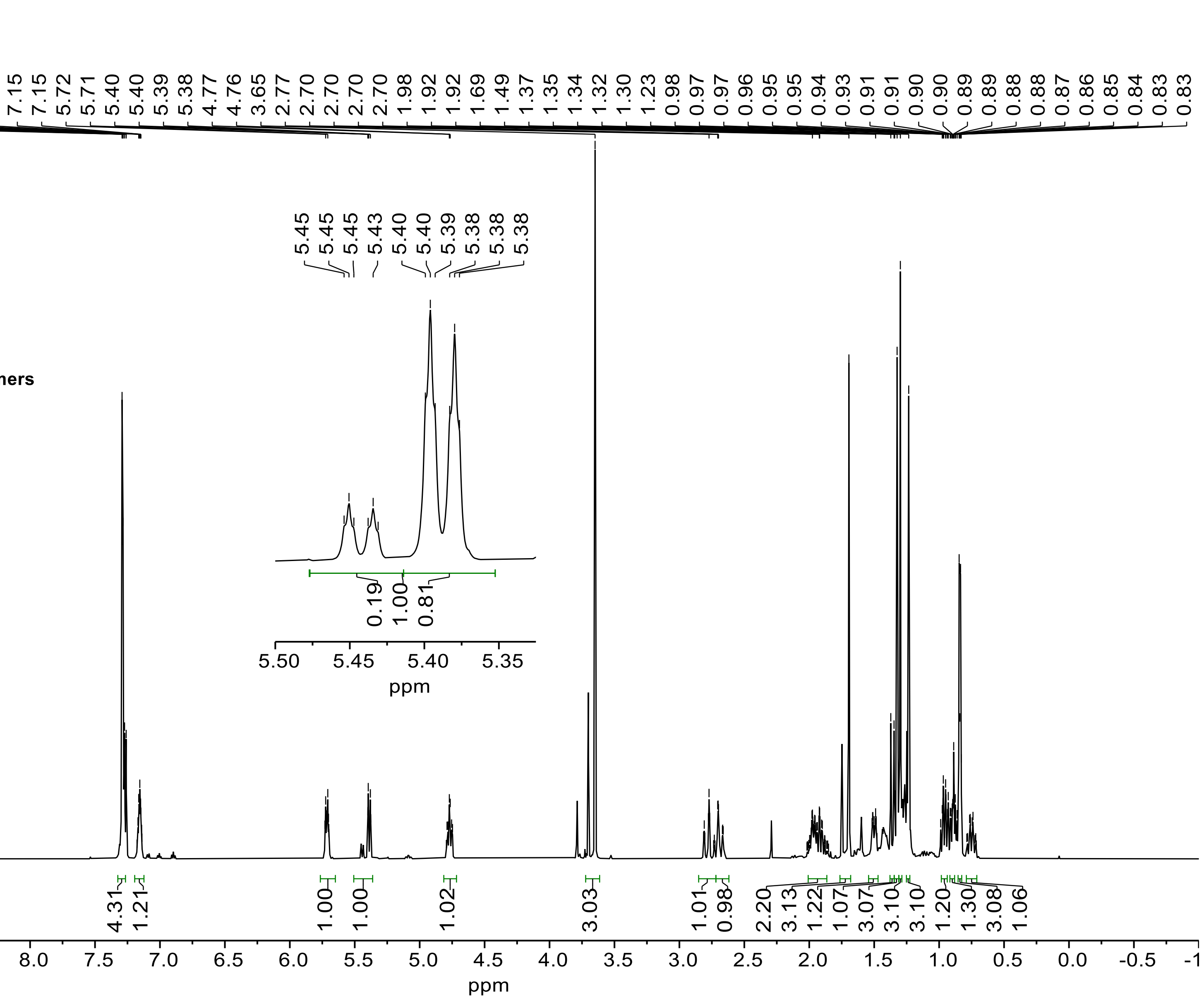

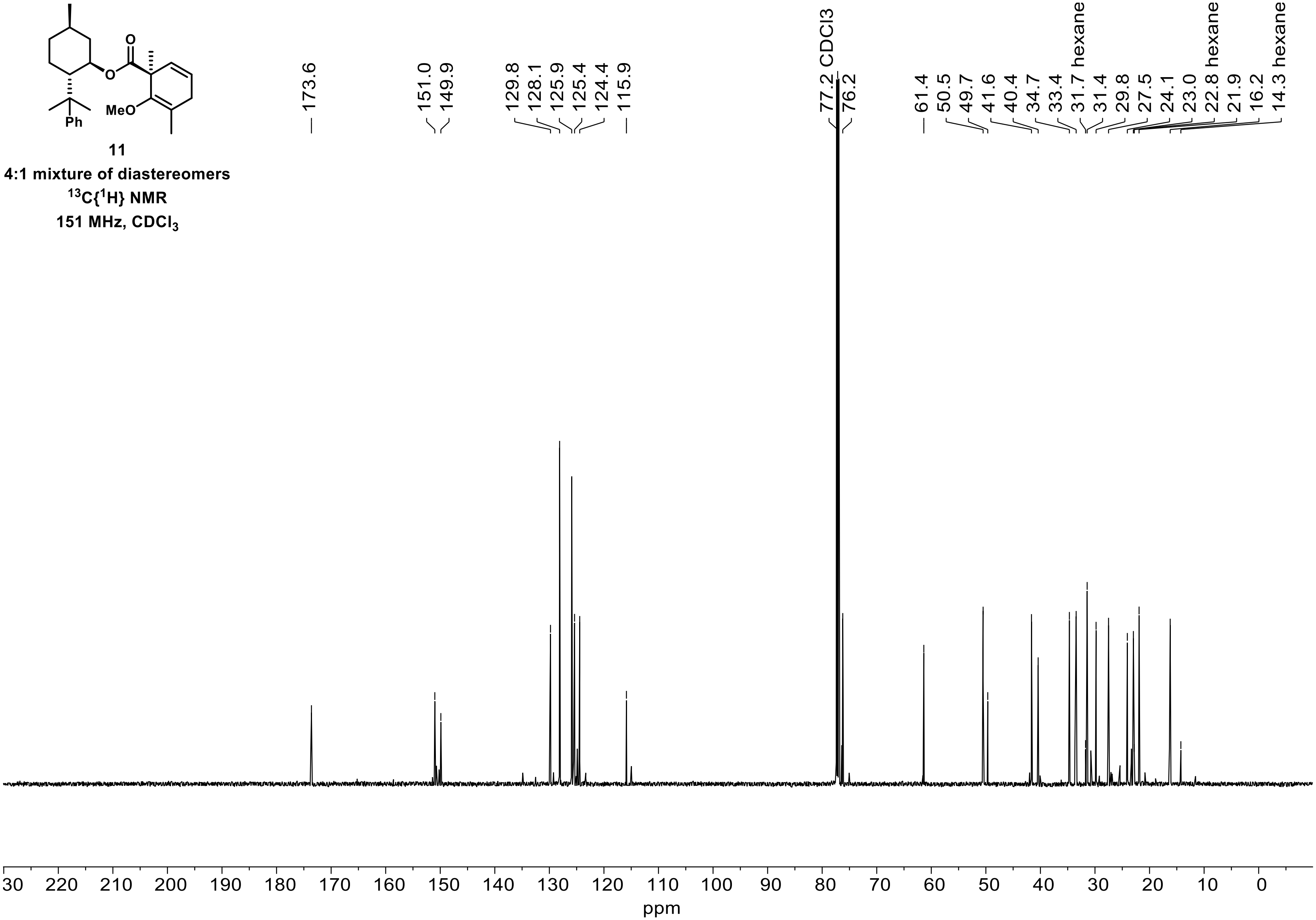
MeO
Ph
O
11
4:1 mixture of diastereomers
$^{13}C\{^{1}H\}$ NMR
151 MHz, $CDCl_3$
173.6
151.0
149.9
129.8
128.1
125.9
125.4
124.4
115.9
77.2 CDCl3
76.2
61.4
50.5
49.7
41.6
40.4
34.7
33.4
31.7 hexane
31.4
29.8
27.5
24.1
23.0
22.8 hexane
21.9
16.2
14.3 hexane
30 220 210 200 190 180 170 160 150 140 130 120 110 100 90 80 70 60 50 40 30 20 10 0
ppm

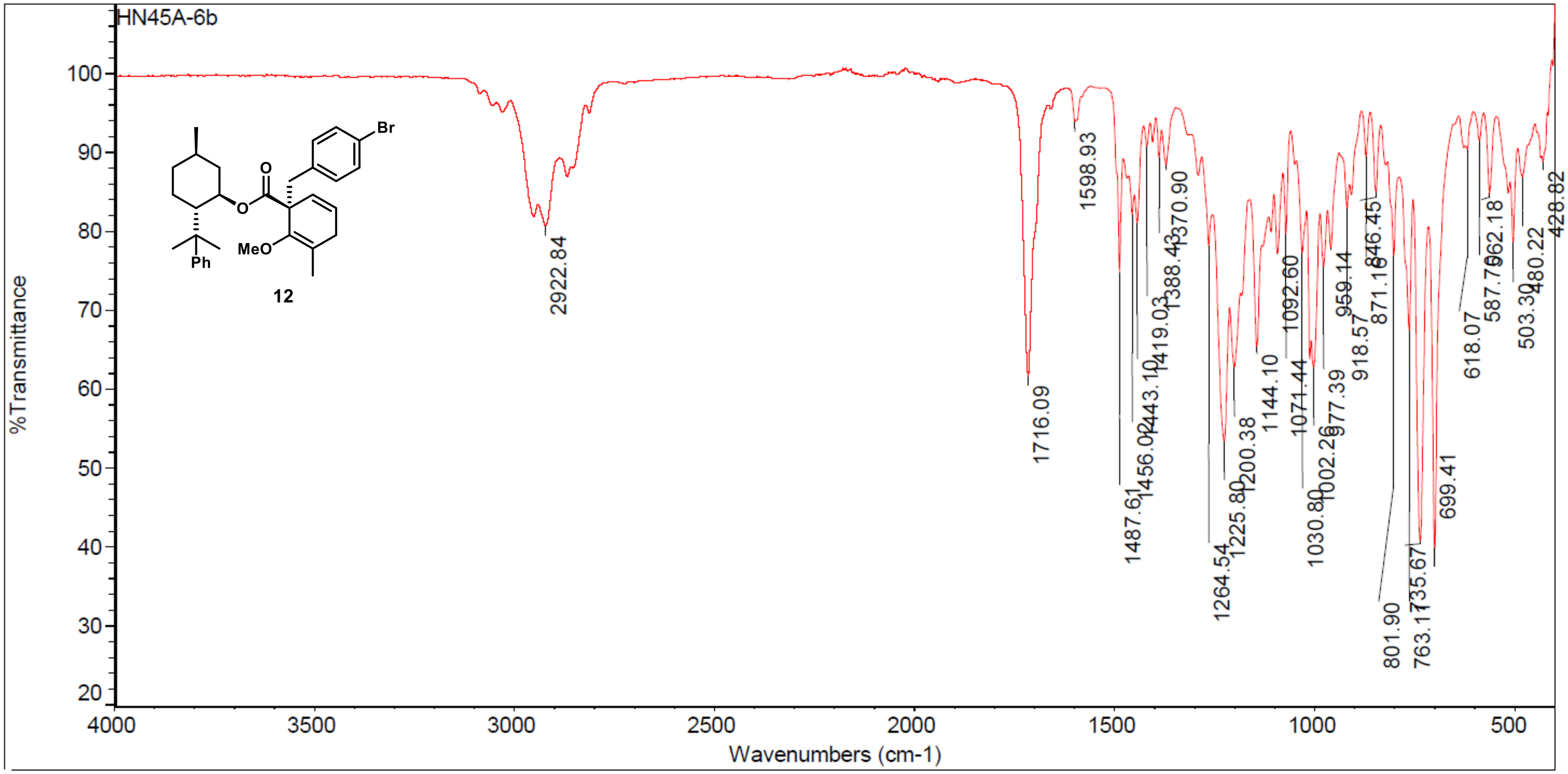
HN45A-6b
12
%Transmittance
Wavenumbers (cm-1)
2922.84
1716.09
1598.93
1487.61
1456.02
1443.10
1419.03
1388.43
1370.90
1264.54
1225.80
1200.38
1144.10
1092.60
1071.44
1030.80
1002.26
977.39
959.14
918.57
871.16
846.45
801.90
763.17
735.67
699.41
618.07
587.79
562.18
503.30
480.22
428.82

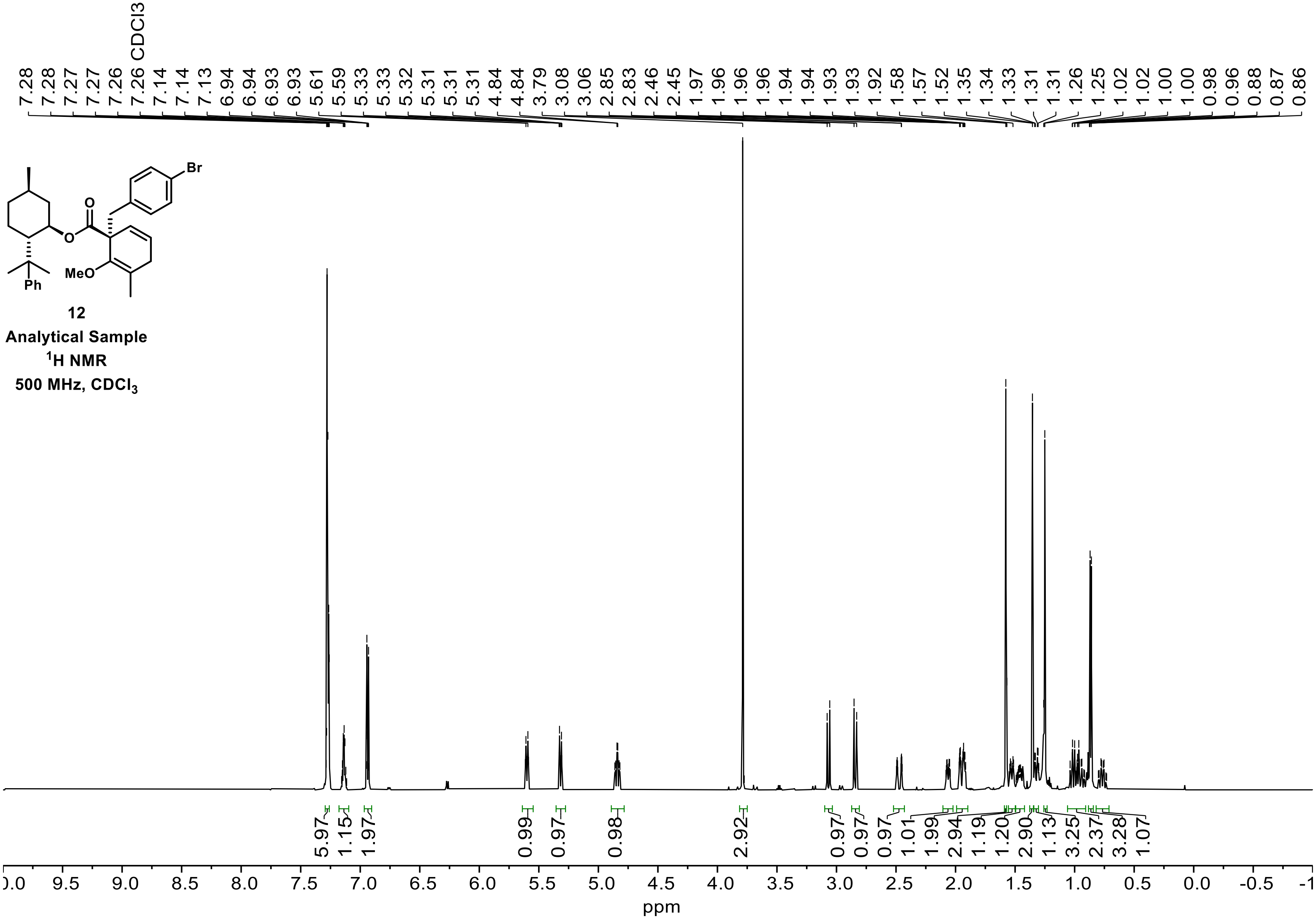

CDCl3
7.28
7.28
7.27
7.27
7.26
7.26
7.14
7.14
7.13
6.94
6.94
6.93
6.93
5.61
5.59
5.33
5.33
5.32
5.31
5.31
4.84
4.84
3.79
3.08
3.06
2.85
2.83
2.46
2.45
1.97
1.96
1.96
1.96
1.94
1.94
1.93
1.93
1.92
1.58
1.57
1.52
1.35
1.34
1.33
1.31
1.31
1.26
1.25
1.02
1.02
1.00
1.00
0.98
0.96
0.88
0.87
0.86
Br
O
O
MeO
Ph
12
Analytical Sample
^{1}H NMR
500 MHz, CDCl$_3$
5.97
1.15
1.97
0.99
0.97
0.98
2.92
0.97
0.97
0.97
1.01
1.99
2.94
1.19
1.20
2.90
1.13
3.25
2.37
3.28
1.07
ppm

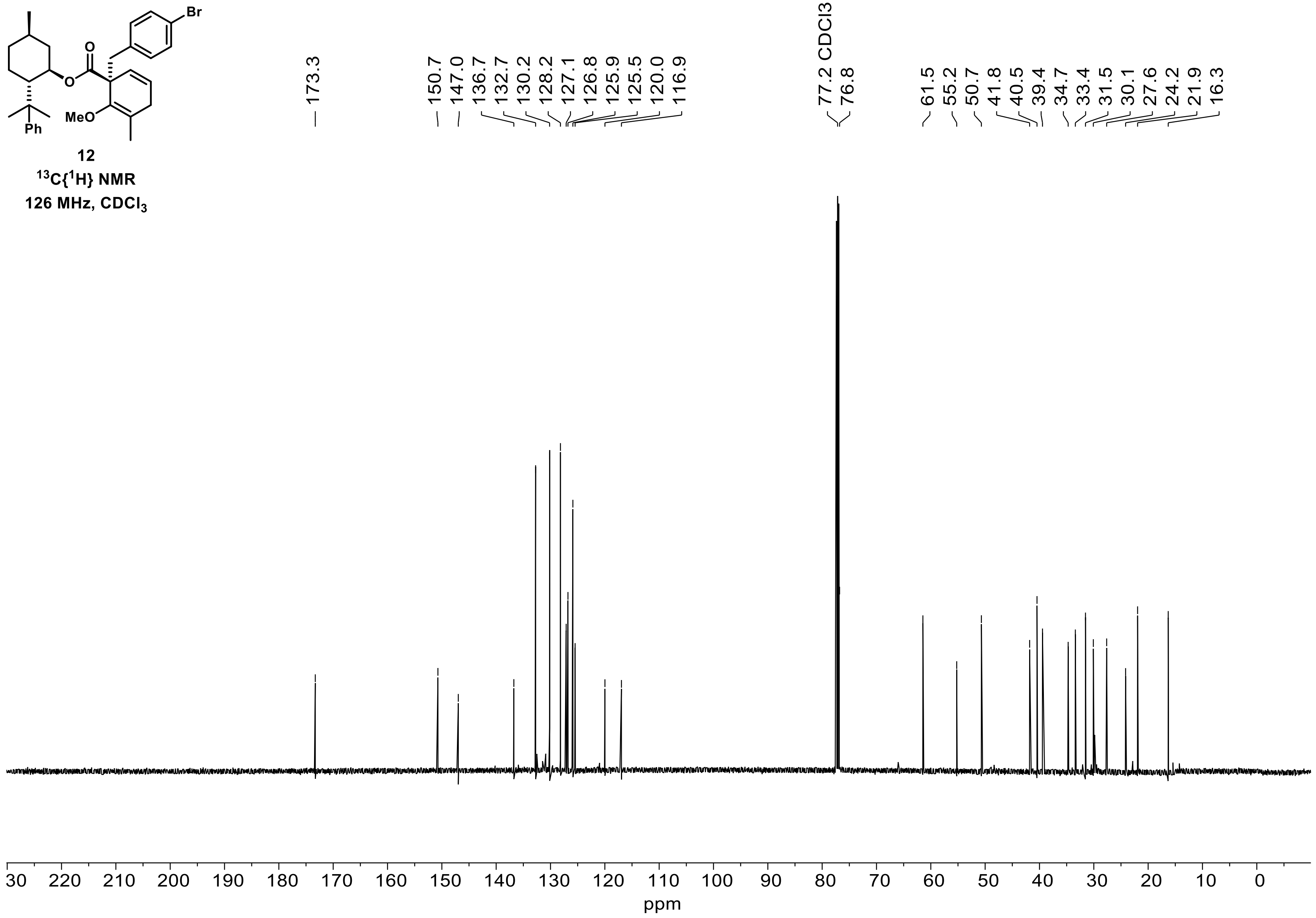
Br
O
O
MeO
Ph
12
$^{13}C\{^1H\}$ NMR
126 MHz, $CDCl_3$
173.3
150.7
147.0
136.7
132.7
130.2
128.2
127.1
126.8
125.9
125.5
120.0
116.9
77.2 CDCl3
76.8
61.5
55.2
50.7
41.8
40.5
39.4
34.7
33.4
31.5
30.1
27.6
24.2
21.9
16.3
30
220
210
200
190
180
170
160
150
140
130
120
110
100
90
80
70
60
50
40
30
20
10
0
ppm

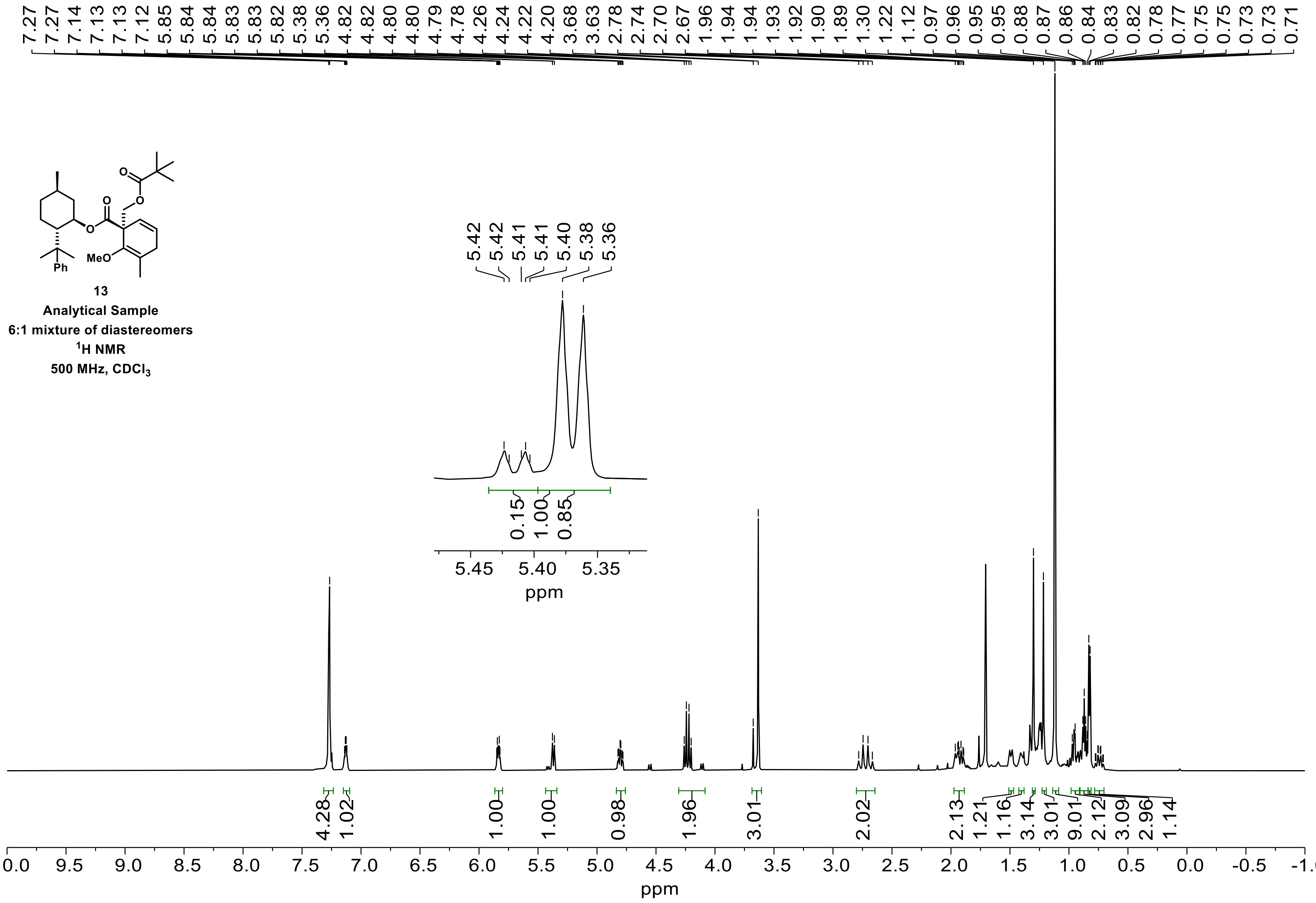

13
Analytical Sample
6:1 mixture of diastereomers
^{1}H NMR
500 MHz, $CDCl_3$

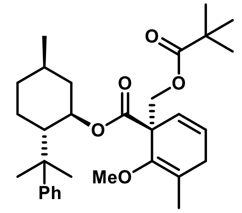

13
6:1 mixture of diastereomers
$^{13}C\{^{1}H\}$ NMR
151 MHz, $CDCl_3$

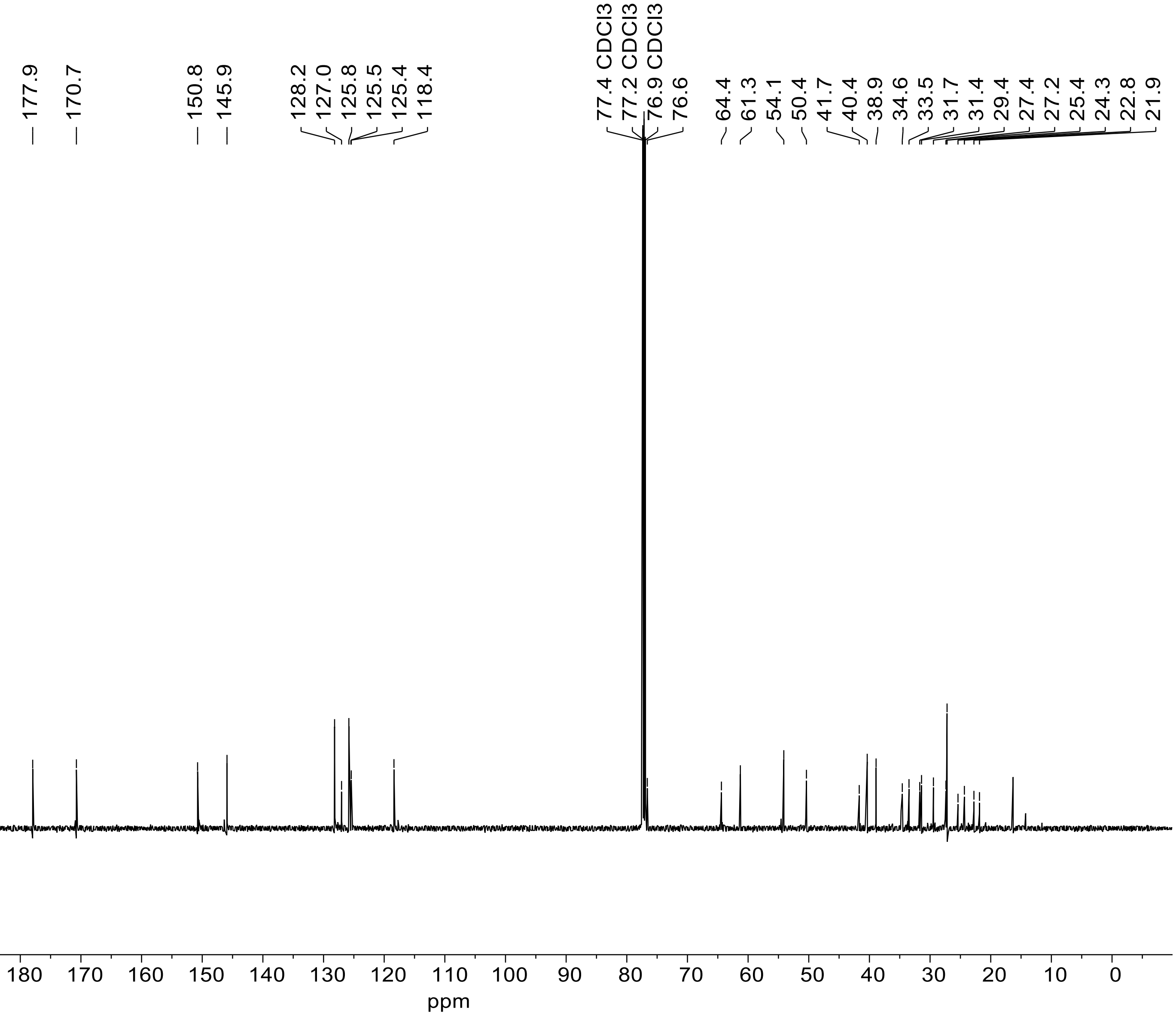

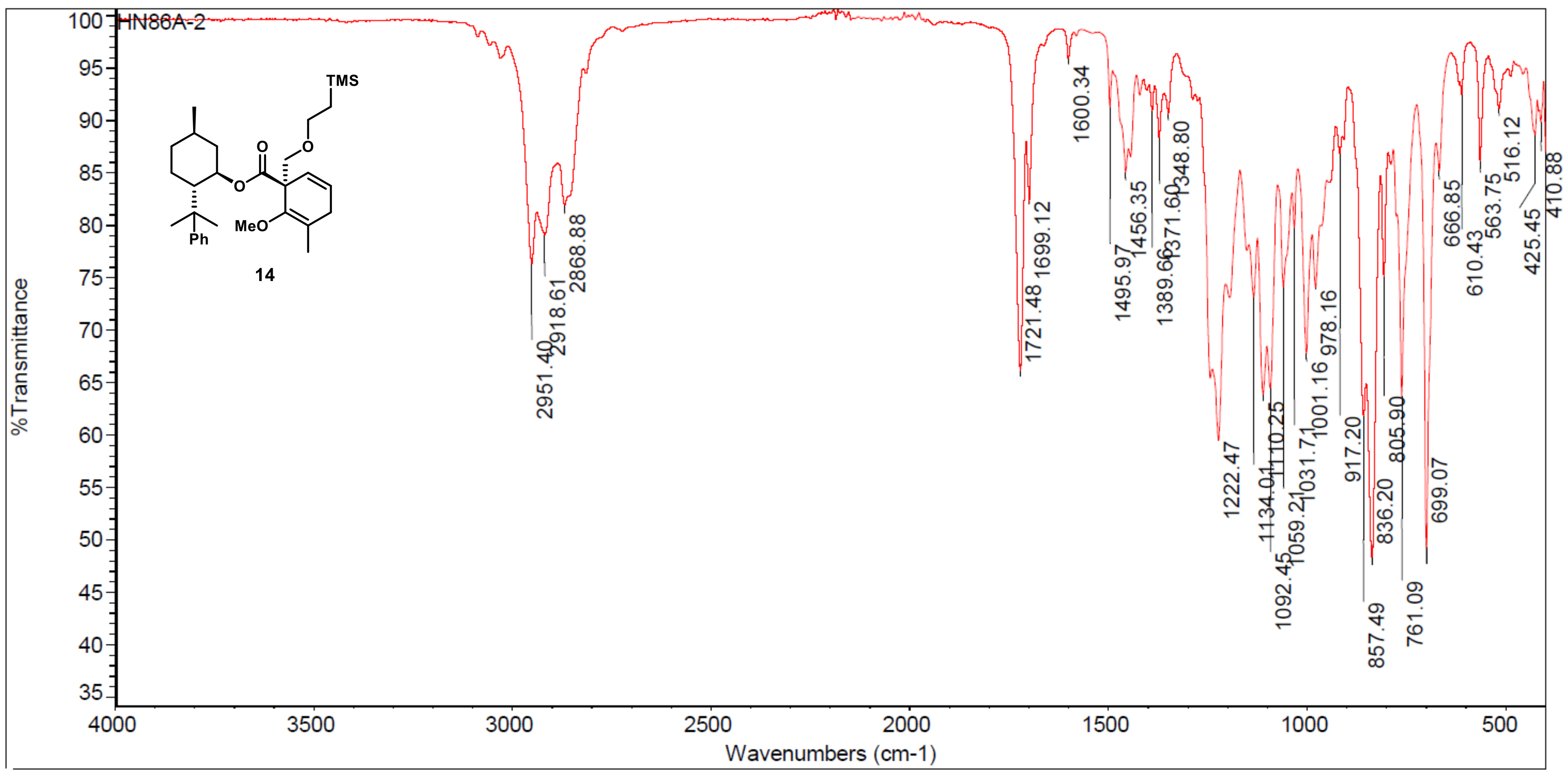
HN86A-2
TMS
O
O
O
MeO
Ph
14
%Transmittance
Wavenumbers (cm-1)
2951.40
2918.61
2868.88
1721.48
1699.12
1600.34
1495.97
1456.35
1389.66
1371.60
1348.80
1222.47
1134.01
1110.25
1092.45
1059.21
1031.71
1001.16
978.16
917.20
857.49
836.20
805.90
761.09
699.07
666.85
610.43
563.75
516.12
425.45
410.88

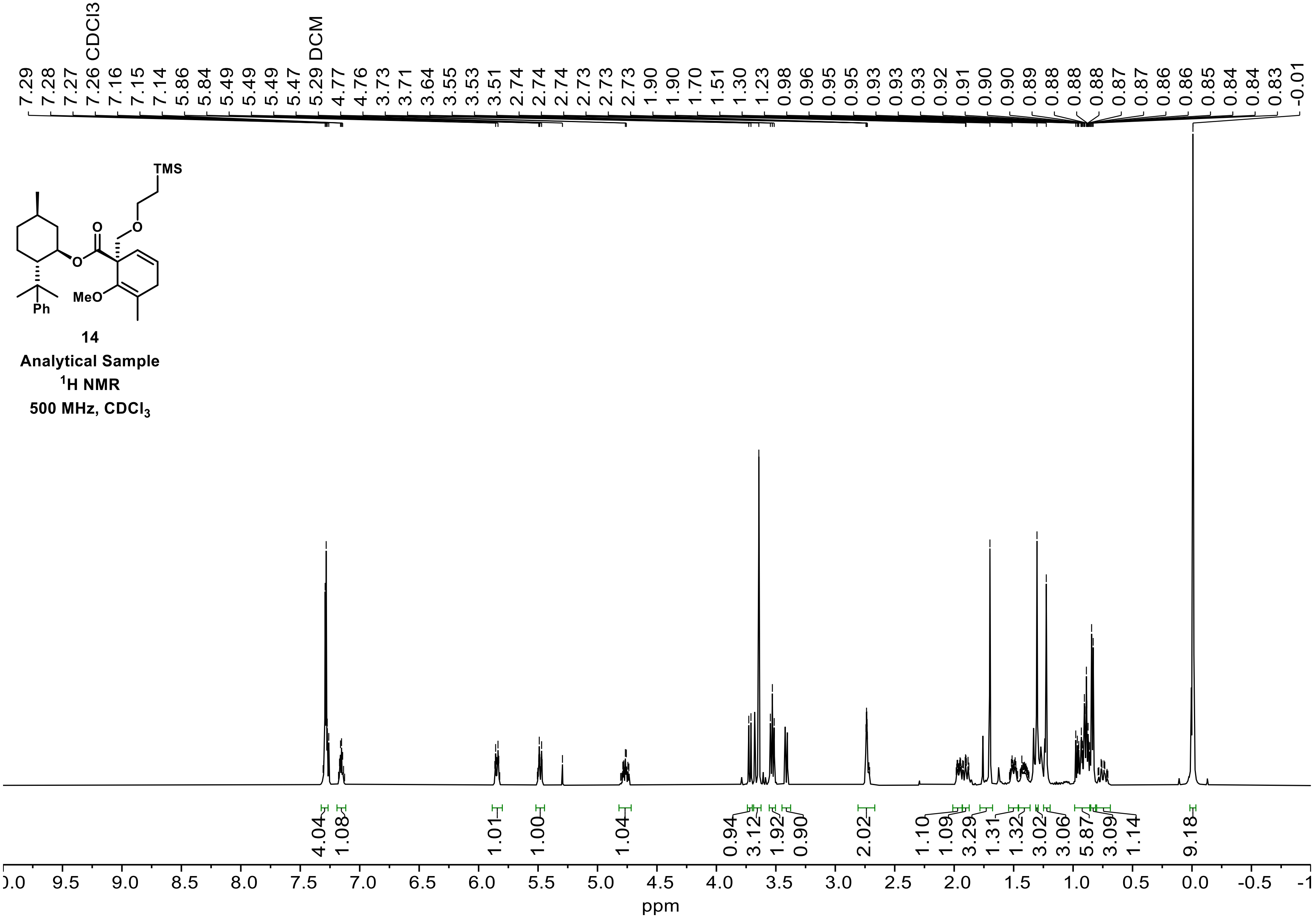
7.29
7.28
7.27
7.26 CDCl3
7.16
7.15
7.14
5.86
5.84
5.49
5.49
5.49
5.47
5.29 DCM
4.77
4.76
3.73
3.71
3.64
3.55
3.53
3.51
2.74
2.74
2.74
2.73
2.73
2.73
1.90
1.90
1.70
1.51
1.30
1.23
0.98
0.96
0.95
0.95
0.93
0.93
0.93
0.92
0.91
0.90
0.90
0.89
0.88
0.88
0.88
0.87
0.87
0.86
0.86
0.85
0.84
0.84
0.83
-0.01
TMS
O
O
O
MeO
Ph
14
Analytical Sample
^{1}H NMR
500 MHz, $CDCl_3$
4.04
1.08
1.01
1.00
1.04
0.94
3.12
1.92
0.90
2.02
1.10
1.09
3.29
1.31
1.32
3.02
3.06
5.87
3.09
1.14
9.18
10.0
9.5
9.0
8.5
8.0
7.5
7.0
6.5
6.0
5.5
5.0
4.5
4.0
3.5
3.0
2.5
2.0
1.5
1.0
0.5
0.0
-0.5
-1
ppm

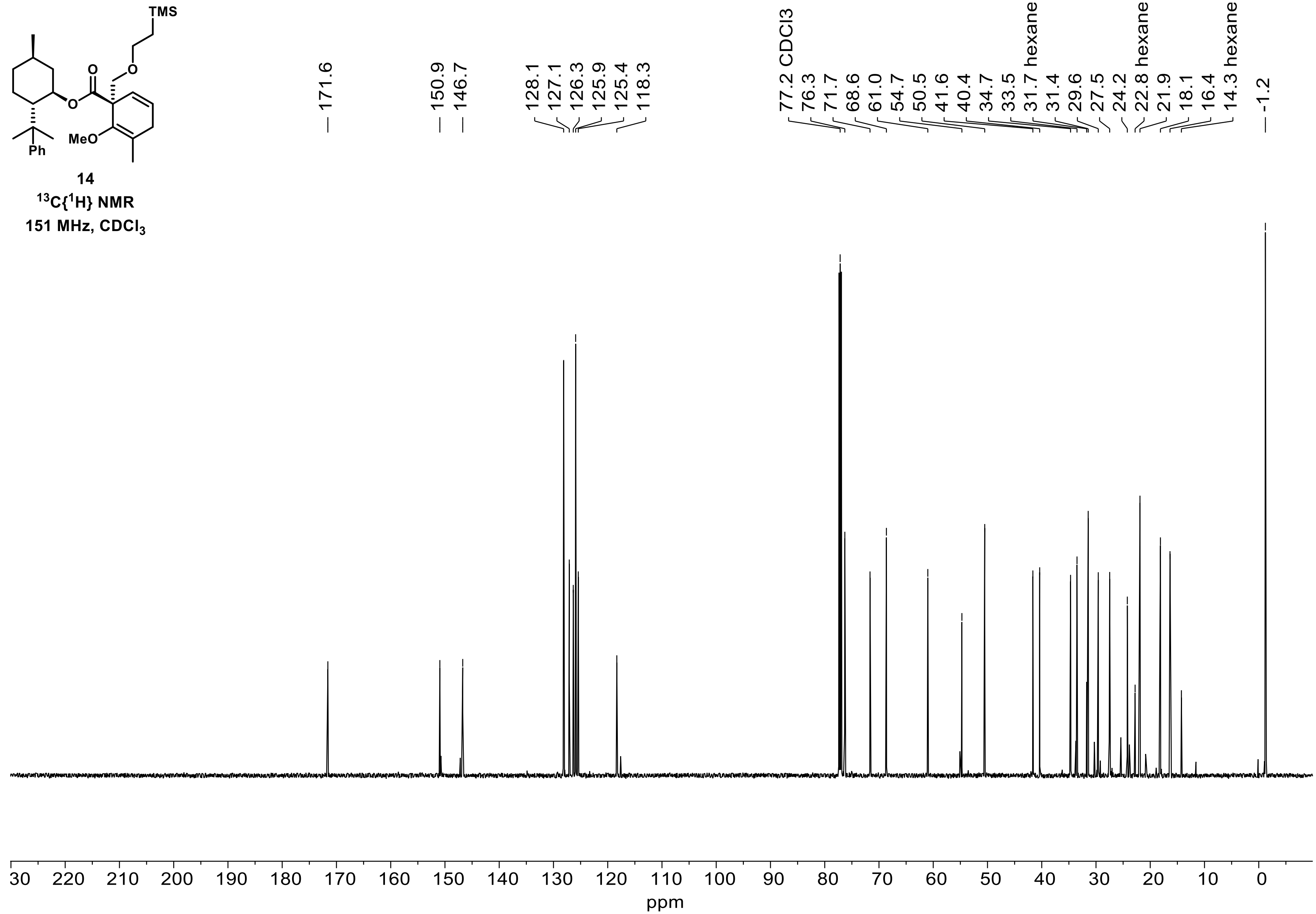
TMS
O
O
O
MeO
Ph
14
13C{1H} NMR
151 MHz, CDCl3
171.6
150.9
146.7
128.1
127.1
126.3
125.9
125.4
118.3
77.2 CDCl3
76.3
71.7
68.6
61.0
54.7
50.5
41.6
40.4
34.7
33.5
31.7 hexane
31.4
29.6
27.5
24.2
22.8 hexane
21.9
18.1
16.4
14.3 hexane
-1.2
30 220 210 200 190 180 170 160 150 140 130 120 110 100 90 80 70 60 50 40 30 20 10 0
ppm

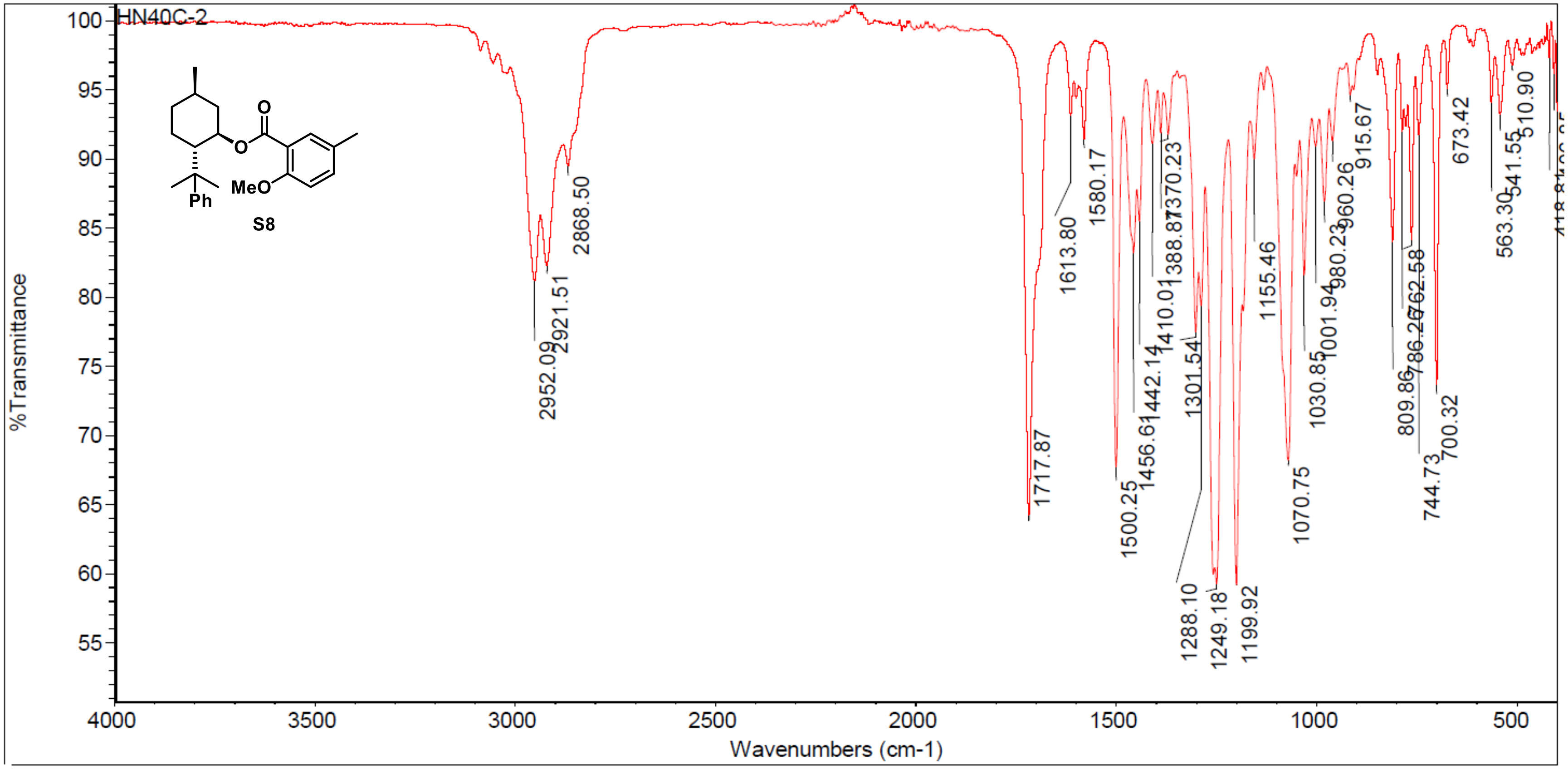
HN40C-2
%Transmittance
Wavenumbers (cm-1)
MeO
Ph
S8
2952.09
2921.51
2868.50
1717.87
1613.80
1580.17
1500.25
1456.61
1442.14
1410.01
1388.87
1370.23
1301.54
1288.10
1249.18
1199.92
1155.46
1070.75
1030.85
1001.94
980.23
960.26
915.67
809.86
786.26
762.58
744.73
700.32
673.42
563.30
541.55
510.90

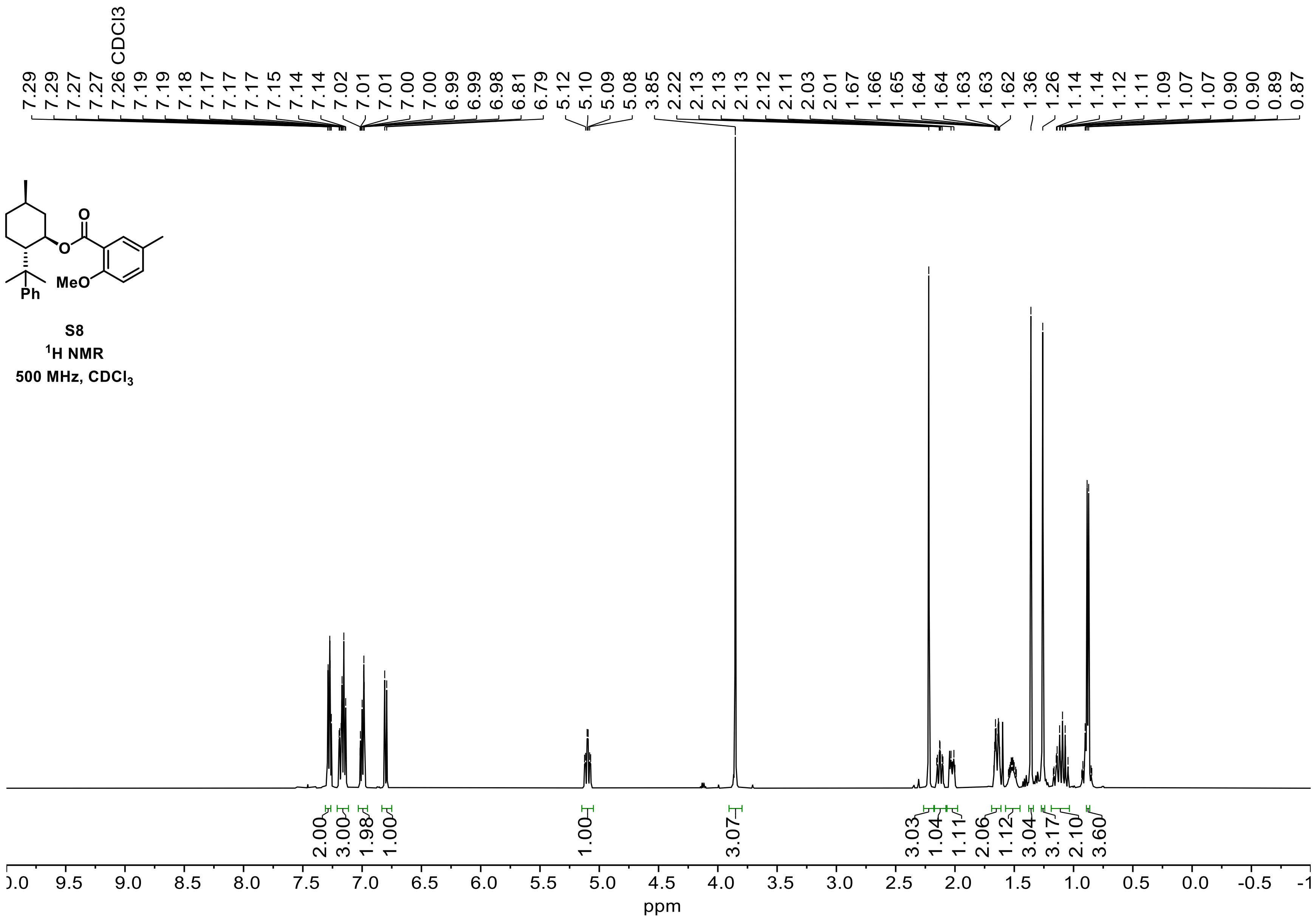
7.29 7.29 7.27 7.27 7.26 CDCl3 7.19 7.19 7.18 7.17 7.17 7.17 7.15 7.14 7.14 7.02 7.01 7.01 7.00 7.00 6.99 6.99 6.98 6.81 6.79 5.12 5.10 5.09 5.08 3.85 2.22 2.13 2.13 2.13 2.12 2.11 2.03 2.01 1.67 1.66 1.65 1.64 1.64 1.63 1.63 1.62 1.36 1.26 1.14 1.14 1.12 1.11 1.09 1.07 1.07 0.90 0.90 0.89 0.87
O
O
MeO
Ph
S8
^{1}H NMR
500 MHz, $CDCl_3$
2.00 3.00 1.98 1.00 1.00 3.07 3.03 1.04 1.11 2.06 1.12 3.04 3.17 2.10 3.60
ppm

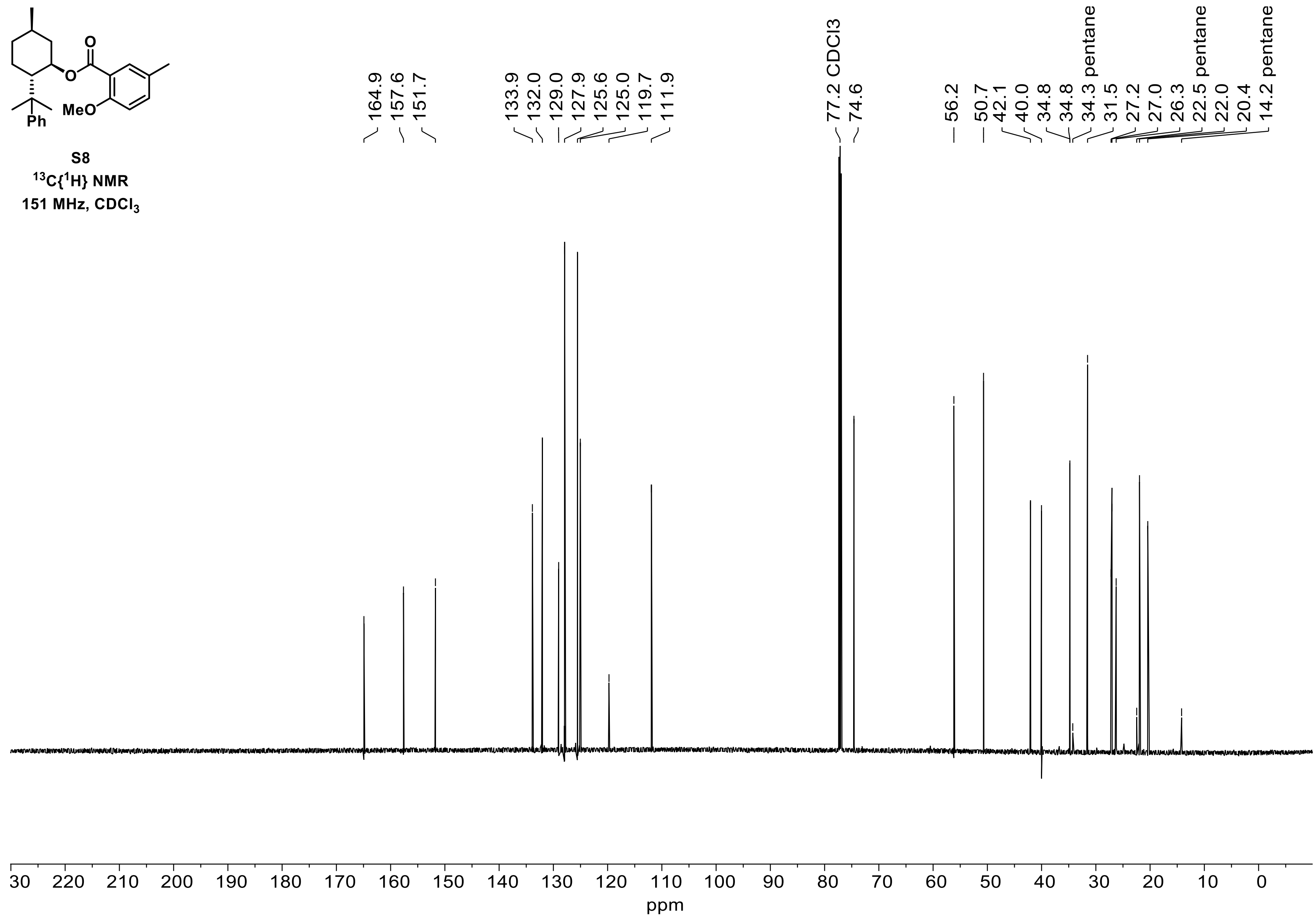

S8
$^{13}C\{^{1}H\}$ NMR
151 MHz, $CDCl_3$
164.9
157.6
151.7
133.9
132.0
129.0
127.9
125.6
125.0
119.7
111.9
77.2 CDCl3
74.6
56.2
50.7
42.1
40.0
34.8
34.8
34.3 pentane
31.5
27.2
27.0
26.3
22.5 pentane
22.0
20.4
14.2 pentane
ppm

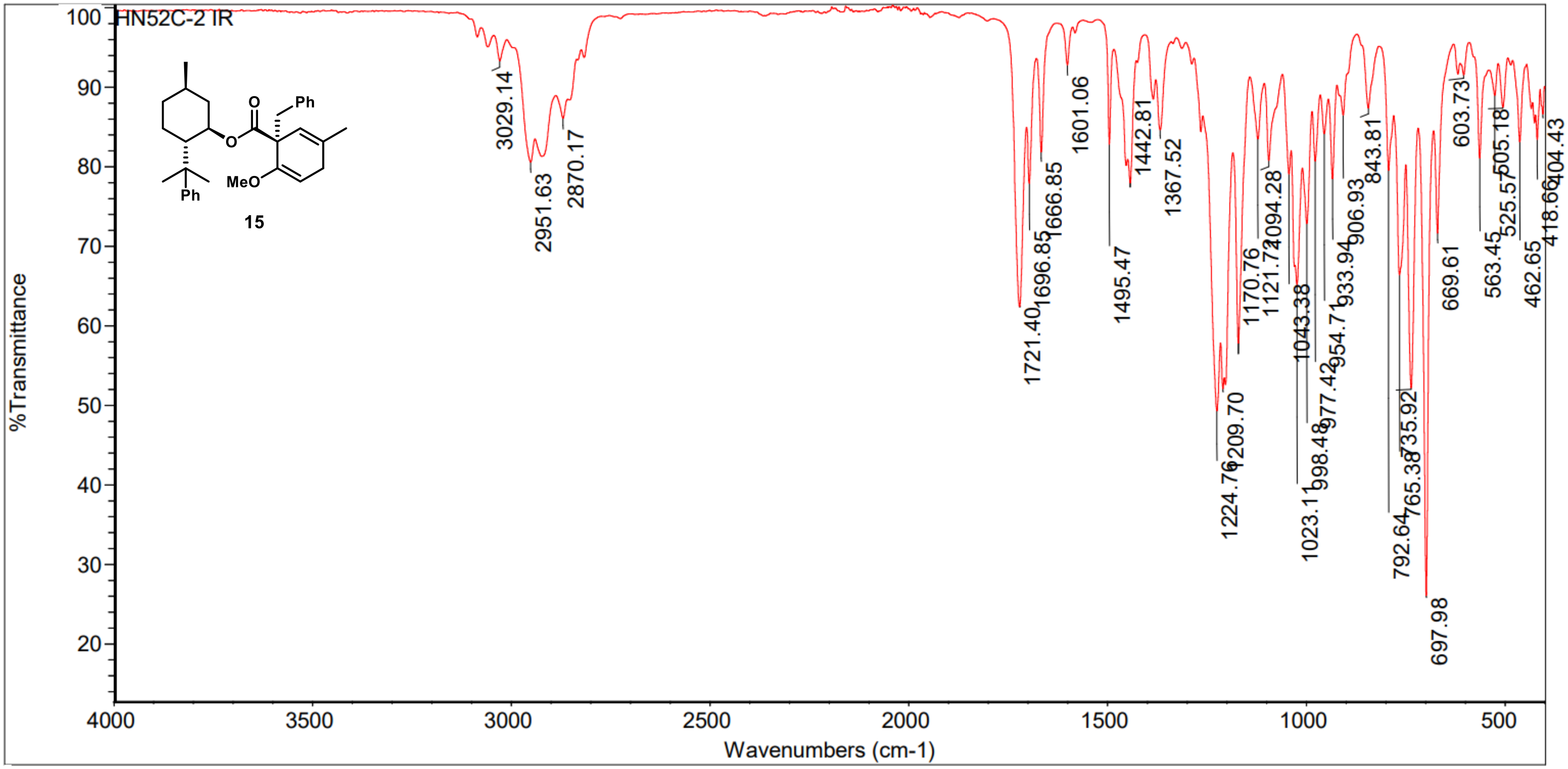
HN52C-2 IR
O
Ph
O
MeO
Ph
15
%Transmittance
Wavenumbers (cm-1)
100
90
80
70
60
50
40
30
20
4000
3500
3000
2500
2000
1500
1000
500
3029.14
2951.63
2870.17
1721.40
1696.85
1666.85
1601.06
1495.47
1442.81
1367.52
1224.76
1209.70
1170.76
1121.73
1094.28
1043.38
1023.11
998.48
977.42
954.71
933.94
906.93
843.81
792.64
765.38
735.92
697.98
669.61
603.73
563.45
525.57
505.18
462.65
418.66
404.43

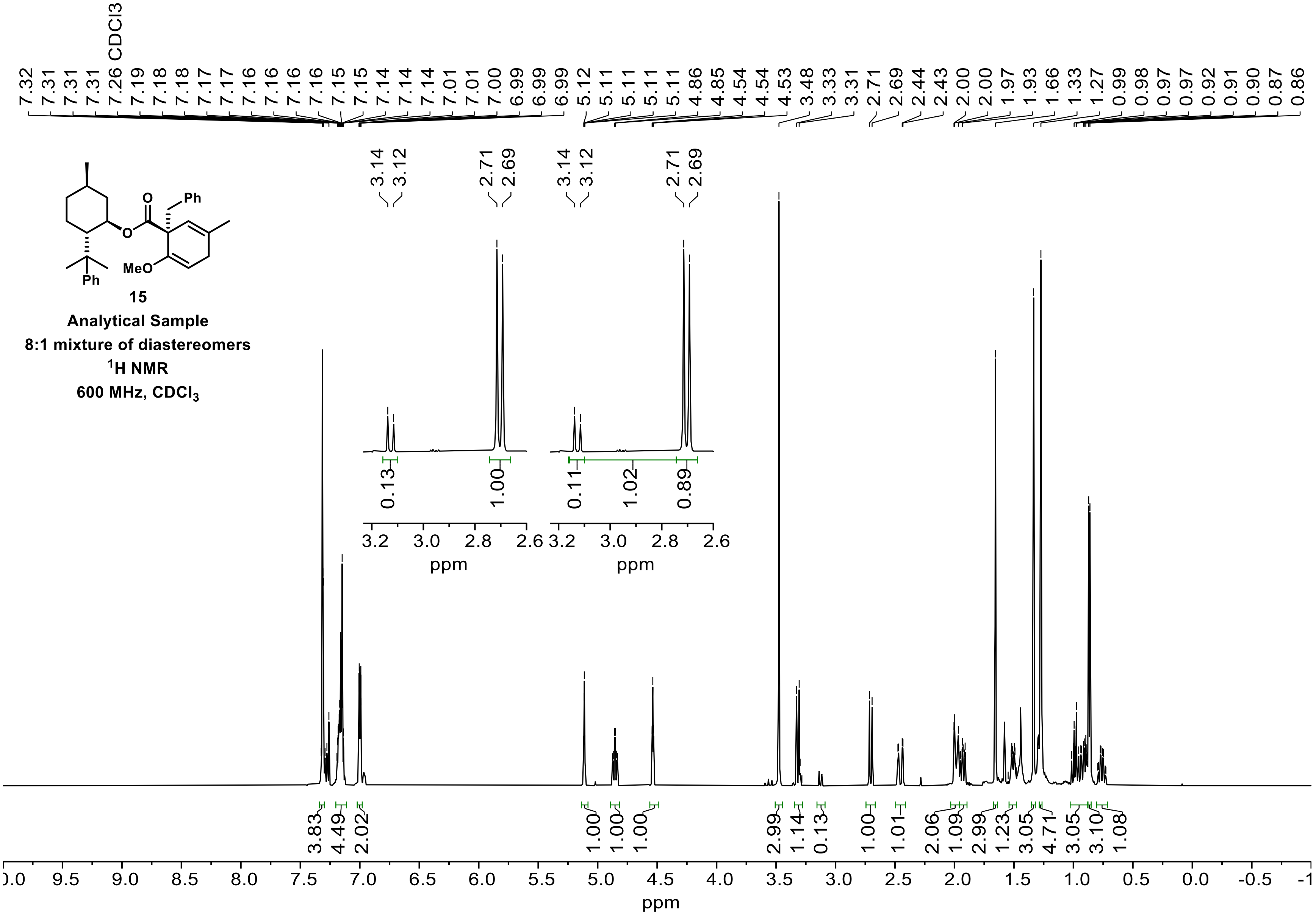

7.32 7.31 7.31 7.31 7.26 CDCl3 7.19 7.18 7.18 7.17 7.17 7.16 7.16 7.16 7.16 7.15 7.15 7.14 7.14 7.14 7.01 7.01 7.00 6.99 6.99 5.12 5.11 5.11 5.11 5.11 4.86 4.85 4.54 4.54 4.53 3.48 3.33 3.31 2.71 2.69 2.44 2.43 2.00 2.00 1.97 1.93 1.66 1.33 1.27 0.99 0.98 0.97 0.97 0.92 0.91 0.90 0.87 0.86
Ph
O
O
MeO
Ph
15
Analytical Sample
8:1 mixture of diastereomers
1H NMR
600 MHz, CDCl3
3.14 3.12 2.71 2.69
0.13 1.00
3.2 3.0 2.8 2.6
ppm
3.14 3.12 2.71 2.69
0.11 1.02 0.89
3.2 3.0 2.8 2.6
ppm
3.83 4.49 2.02 1.00 1.00 1.00 2.99 1.14 0.13 1.00 1.01 2.06 1.09 2.99 1.23 3.05 4.71 3.05 3.10 1.08
10.0 9.5 9.0 8.5 8.0 7.5 7.0 6.5 6.0 5.5 5.0 4.5 4.0 3.5 3.0 2.5 2.0 1.5 1.0 0.5 0.0 -0.5 -1
ppm

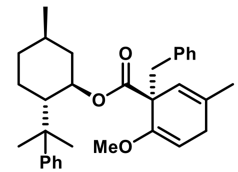

15

8:1 mixture of diastereomers

$^{13}C\{^{1}H\}$ NMR

151 MHz, $CDCl_3$

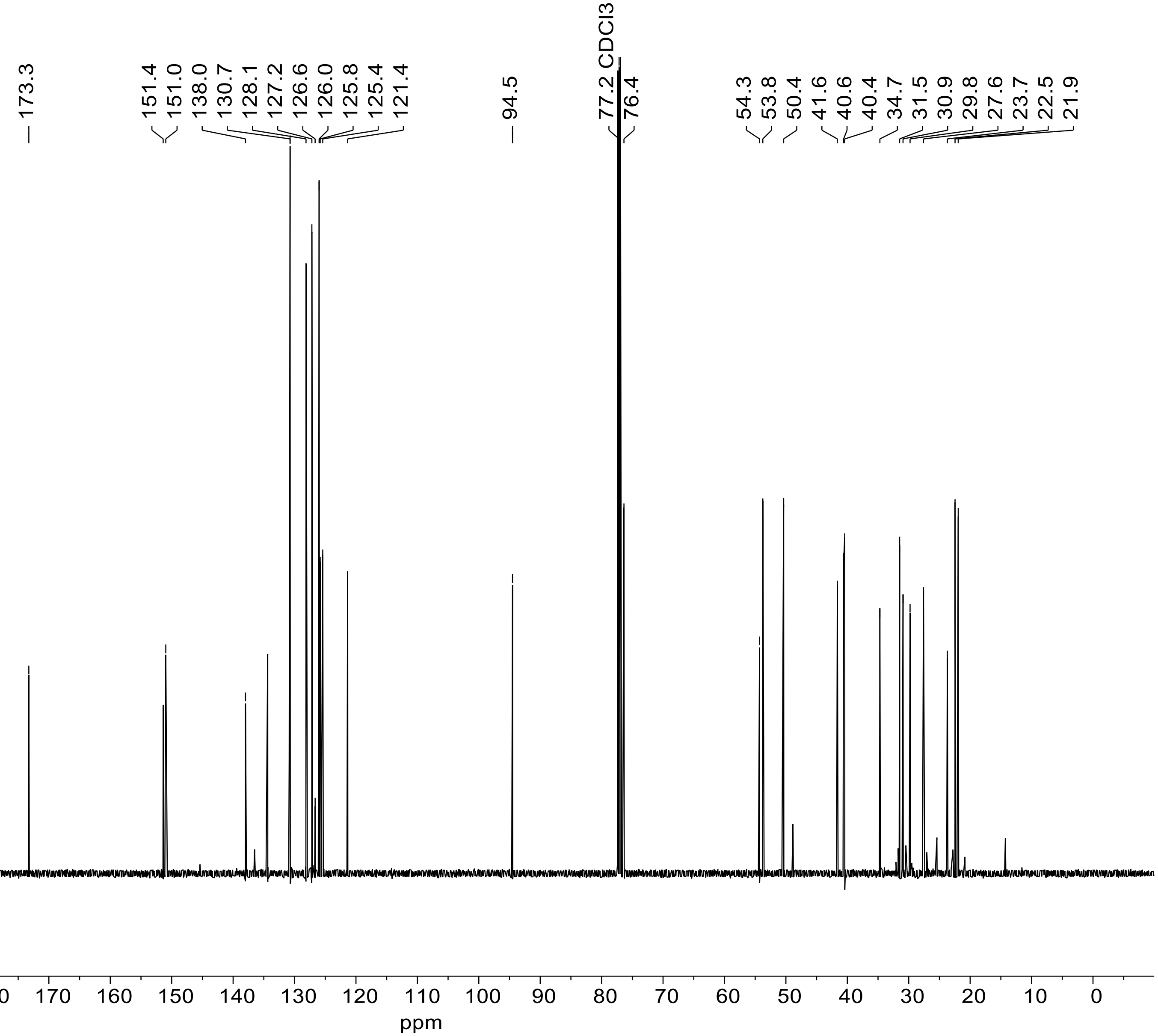

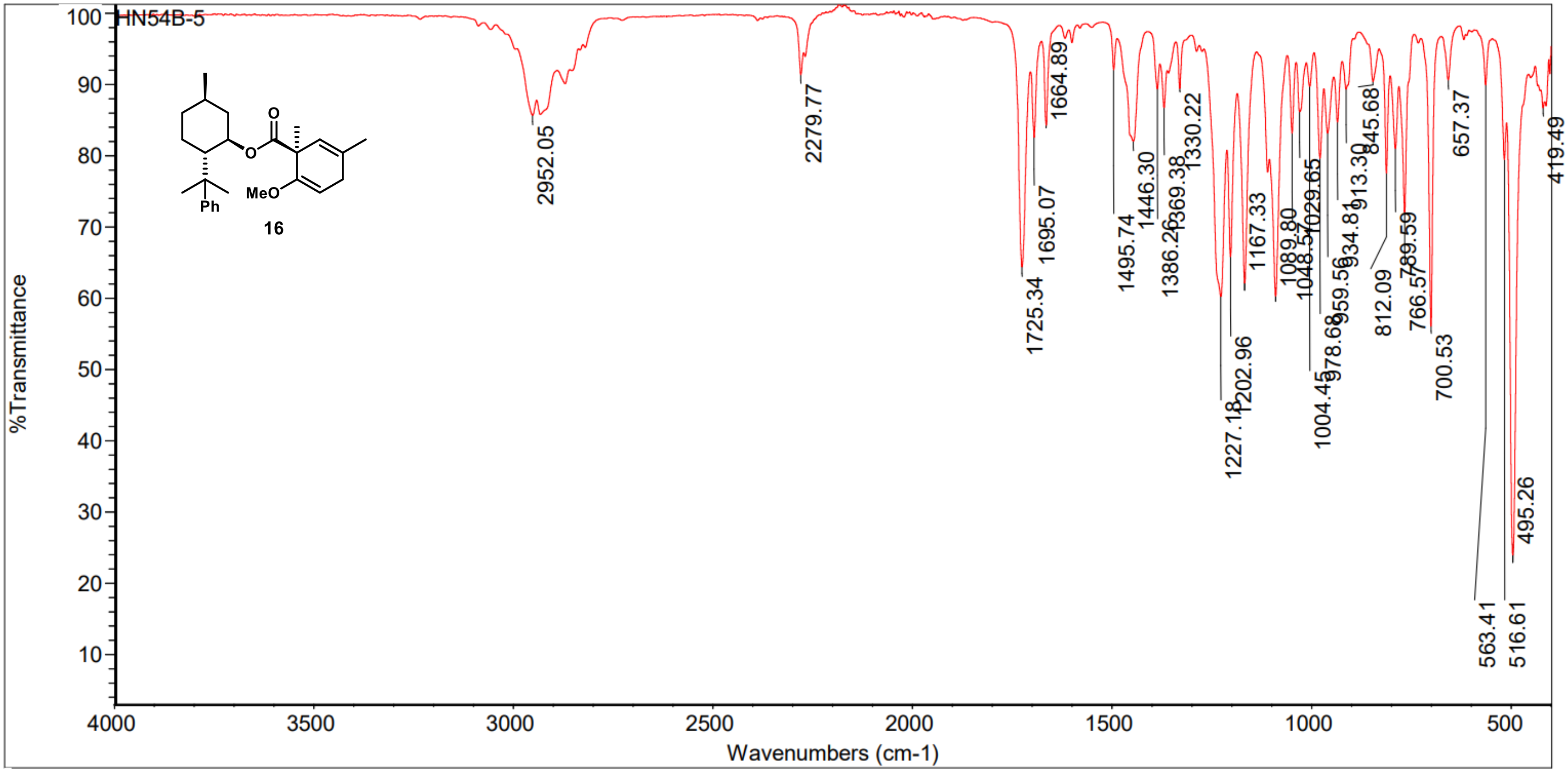
HN54B-5
O
O
MeO
Ph
16
%Transmittance
Wavenumbers (cm-1)
2952.05
2279.77
1725.34
1695.07
1664.89
1495.74
1446.30
1386.26
1369.38
1330.22
1227.18
1202.96
1167.33
1089.80
1048.57
1029.65
1004.45
978.68
959.56
934.81
913.30
845.68
812.09
789.59
766.57
700.53
657.37
563.41
516.61
495.26
419.49

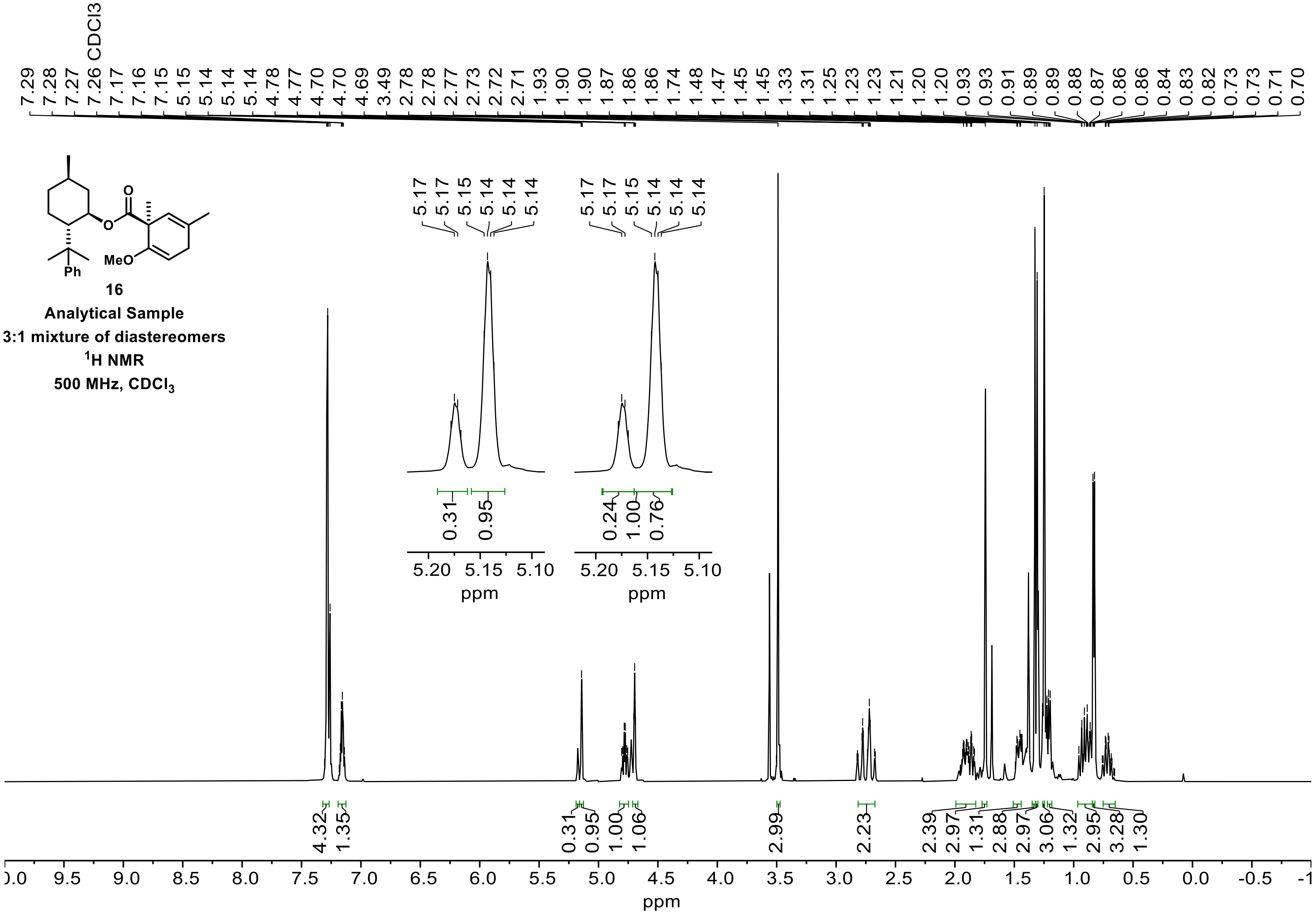
MeO
Ph
16
Analytical Sample
3:1 mixture of diastereomers
^{1}H NMR
500 MHz, CDCl$_3$
CDCl3
7.29 7.28 7.27 7.26 7.17 7.16 7.15 5.15 5.14 5.14 5.14 4.78 4.77 4.70 4.70 4.69 3.49 2.78 2.78 2.77 2.73 2.72 2.71 1.93 1.90 1.90 1.87 1.86 1.86 1.74 1.48 1.47 1.45 1.45 1.33 1.31 1.25 1.23 1.23 1.21 1.20 1.20 0.93 0.93 0.91 0.89 0.89 0.88 0.87 0.86 0.86 0.84 0.83 0.82 0.73 0.73 0.71 0.70
5.17 5.17 5.15 5.14 5.14 5.14
0.31 0.95
5.20 5.15 5.10
ppm
5.17 5.17 5.15 5.14 5.14 5.14
0.24 1.00 0.76
5.20 5.15 5.10
ppm
4.32 1.35 0.31 0.95 1.00 1.06 2.99 2.23 2.39 2.97 1.31 2.88 2.97 3.06 1.32 2.95 3.28 1.30
9.5 9.0 8.5 8.0 7.5 7.0 6.5 6.0 5.5 5.0 4.5 4.0 3.5 3.0 2.5 2.0 1.5 1.0 0.5 0.0 -0.5
ppm

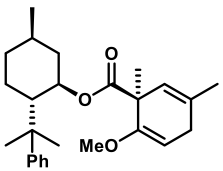

16
3:1 mixture of diastereomers
$^{13}C\{^{1}H\}$ NMR
126 MHz, $CDCl_3$

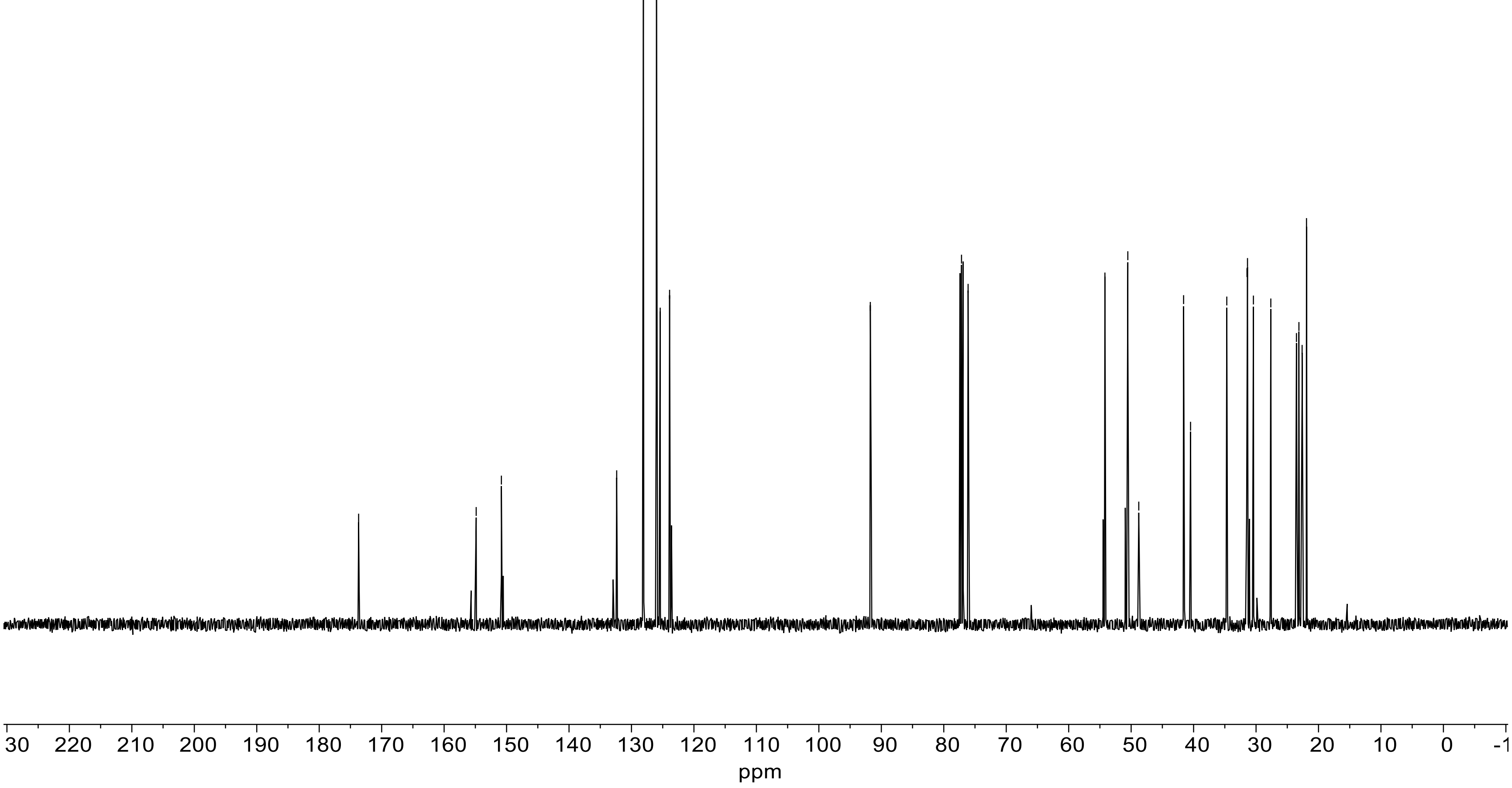

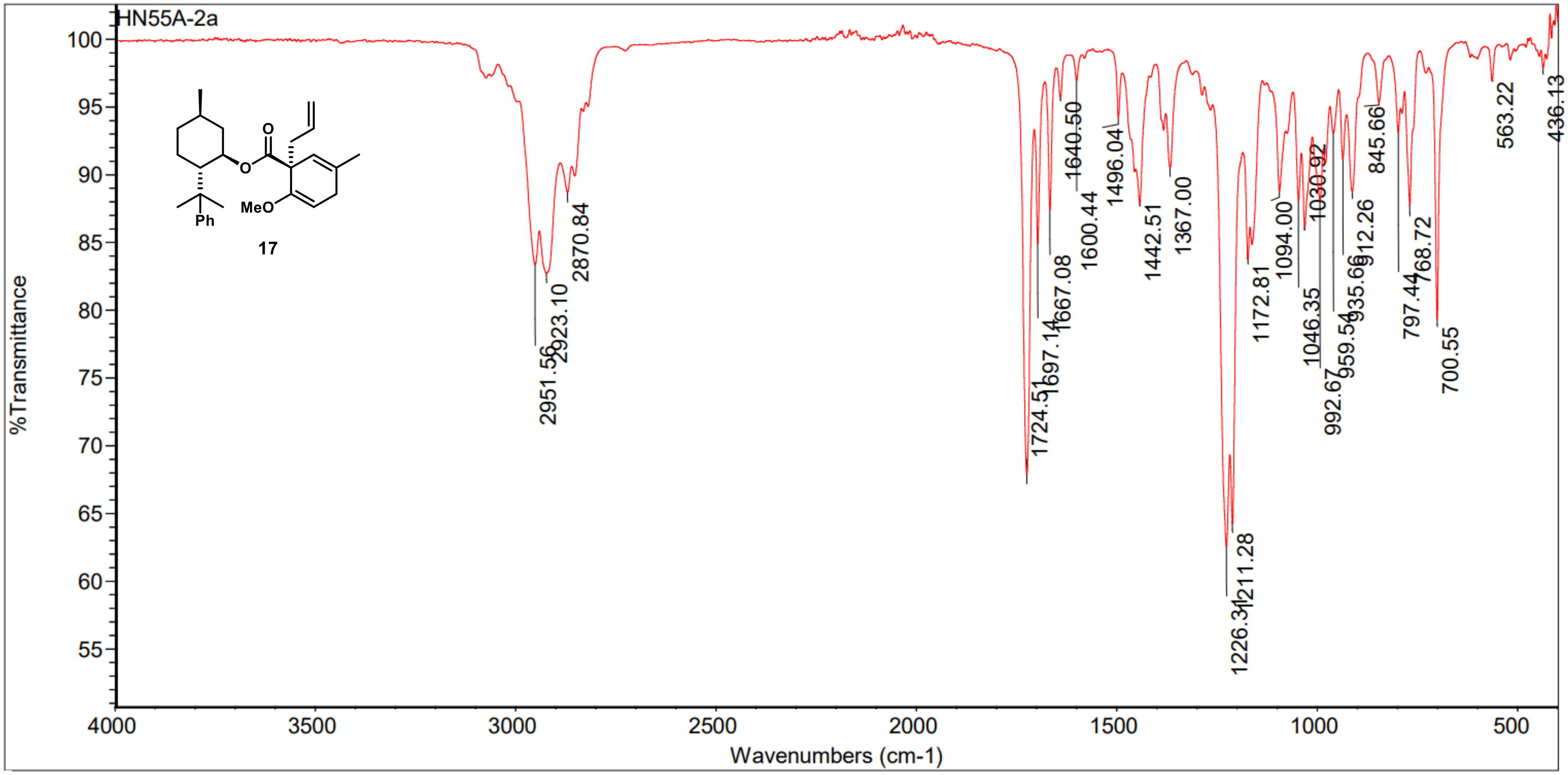
HN55A-2a
%Transmittance
Wavenumbers (cm-1)
MeO
Ph
O
17
2951.56
2923.10
2870.84
1724.51
1697.14
1667.08
1640.50
1600.44
1496.04
1442.51
1367.00
1226.31
1211.28
1172.81
1094.00
1046.35
1030.92
992.67
959.54
935.66
912.26
845.66
797.44
768.72
700.55
563.22
436.13

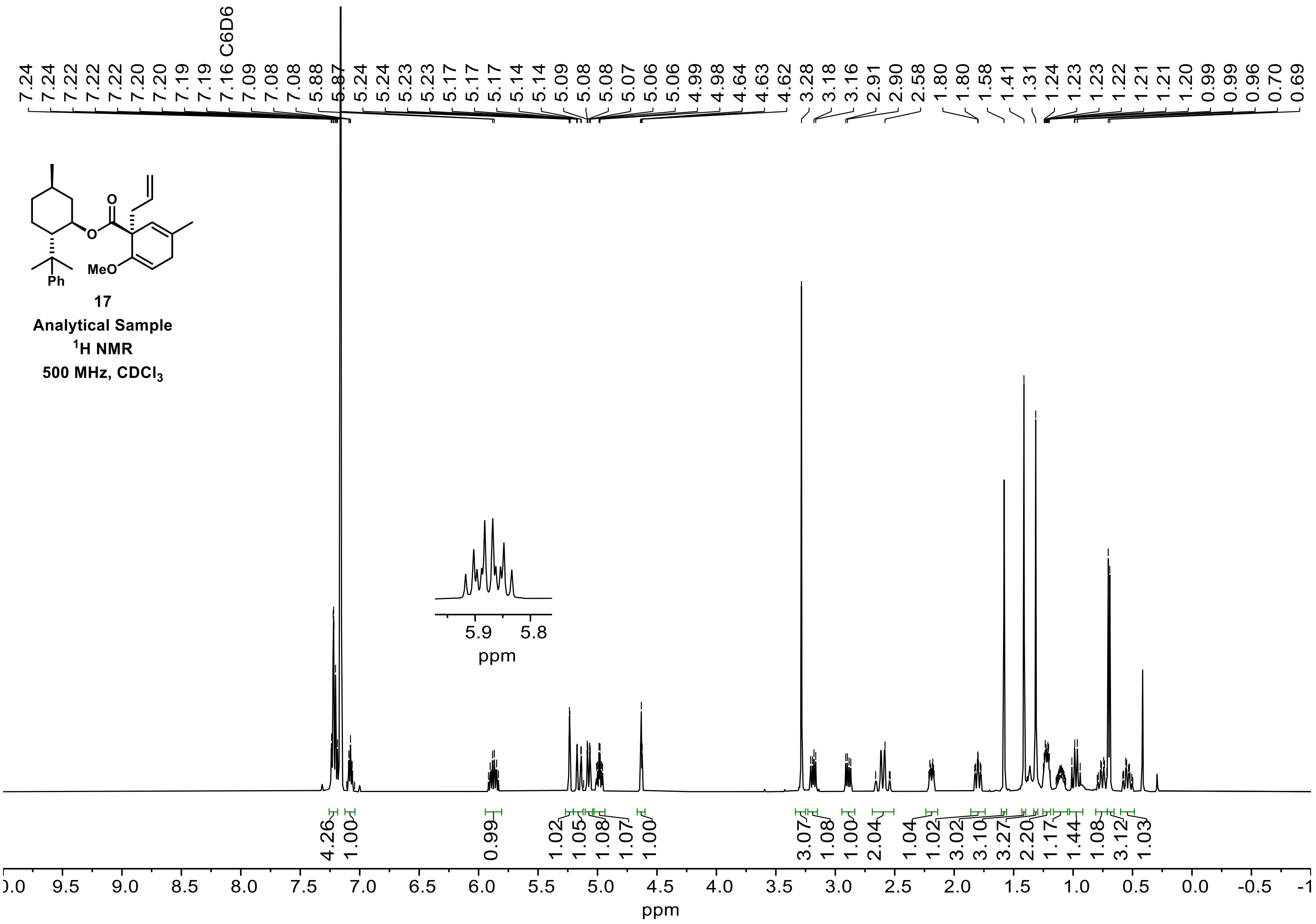

17
Analytical Sample
1H NMR
500 MHz, CDCl3
C6D6
ppm

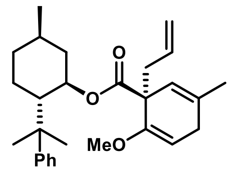

17

$^{13}C\{^{1}H\}$ NMR

126 MHz, $CDCl_3$

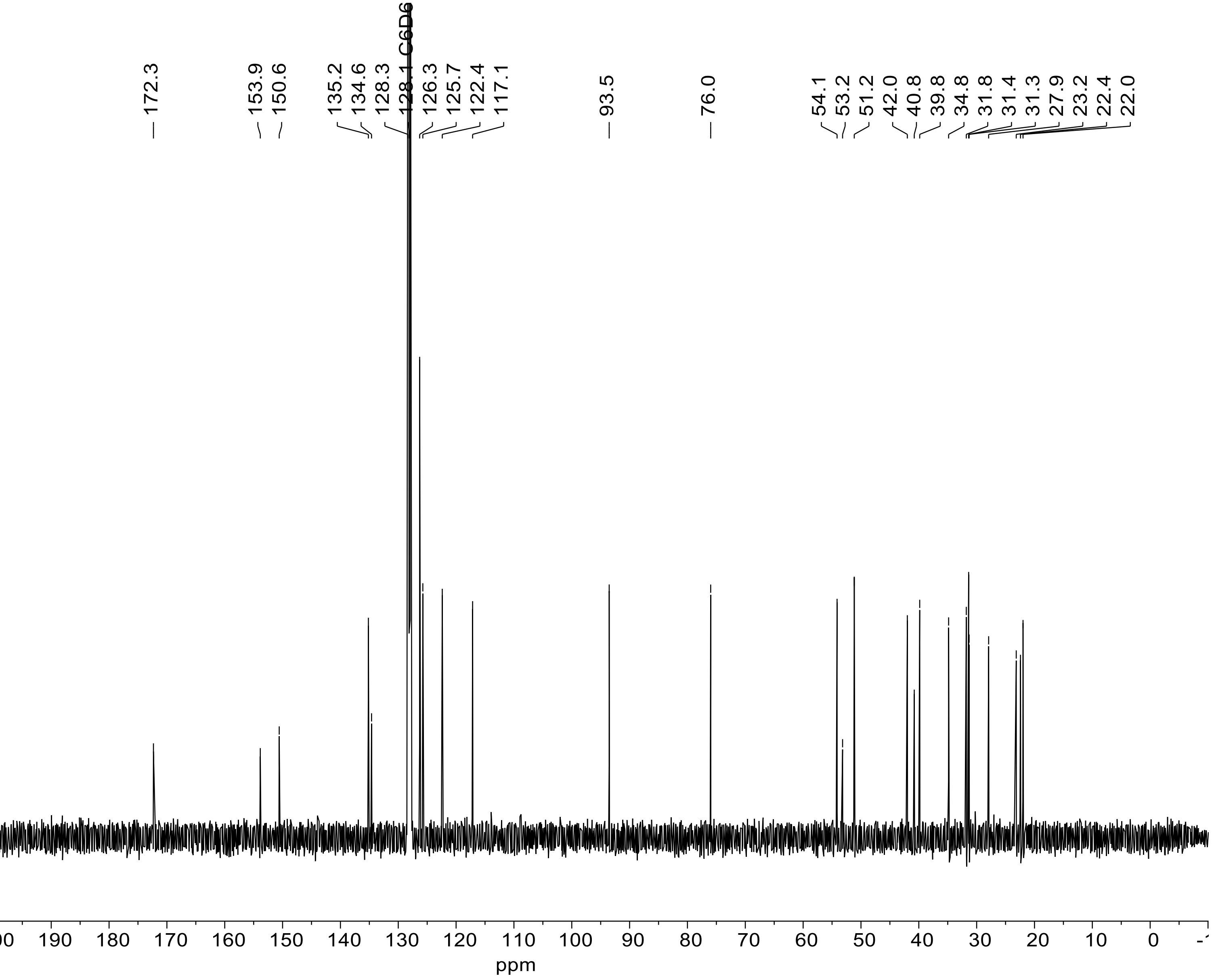

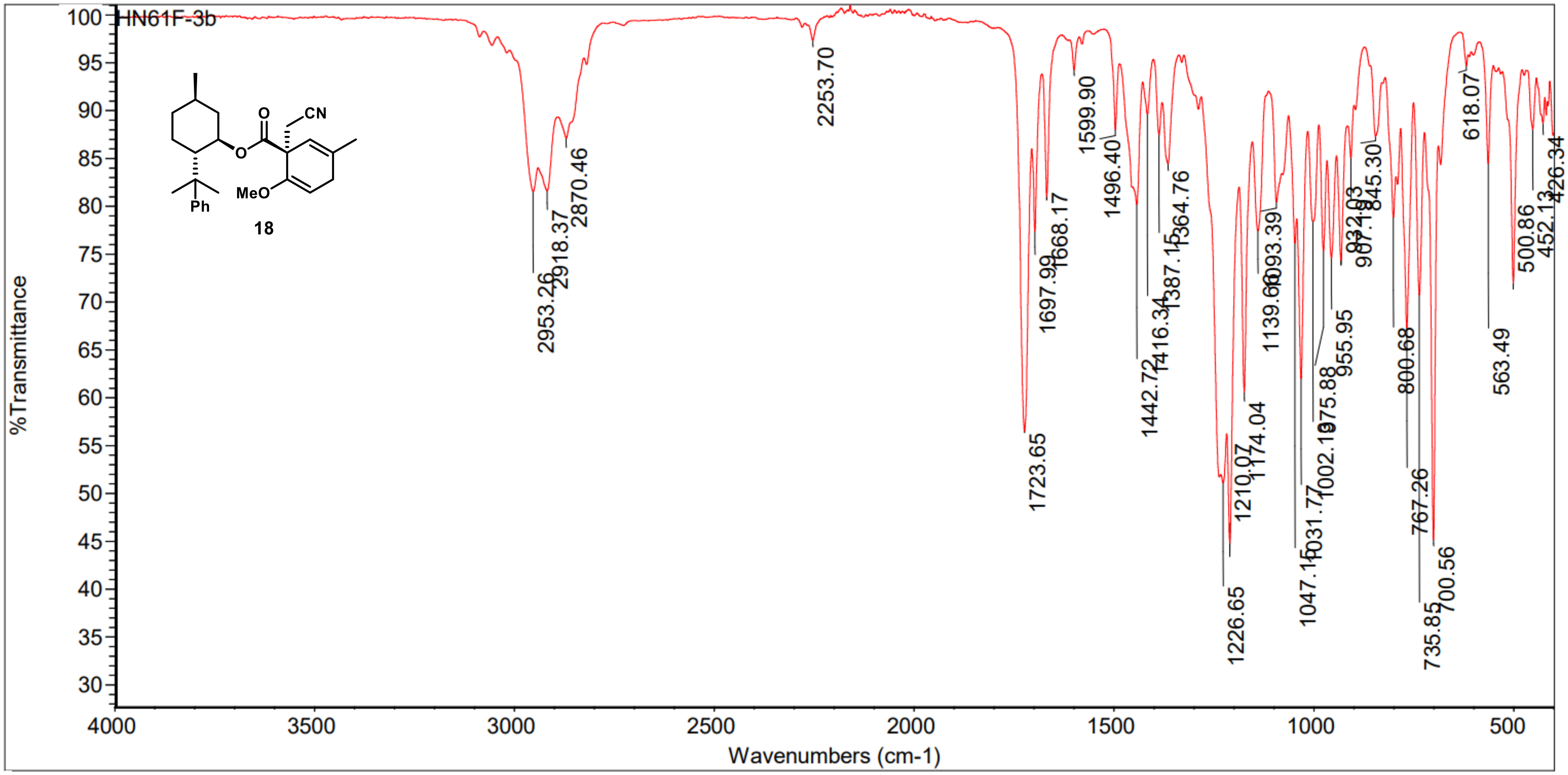

HN61F-3b
%Transmittance
Wavenumbers (cm-1)
O
CN
O
MeO
Ph
18
2953.26
2918.37
2870.46
2253.70
1723.65
1697.99
1668.17
1599.90
1496.40
1442.72
1416.34
1387.15
1364.76
1226.65
1210.07
1174.04
1139.60
1093.39
1047.15
1031.77
1002.19
975.88
955.95
800.68
767.26
735.85
700.56
845.30
618.07
563.49
500.86
452.13
426.34

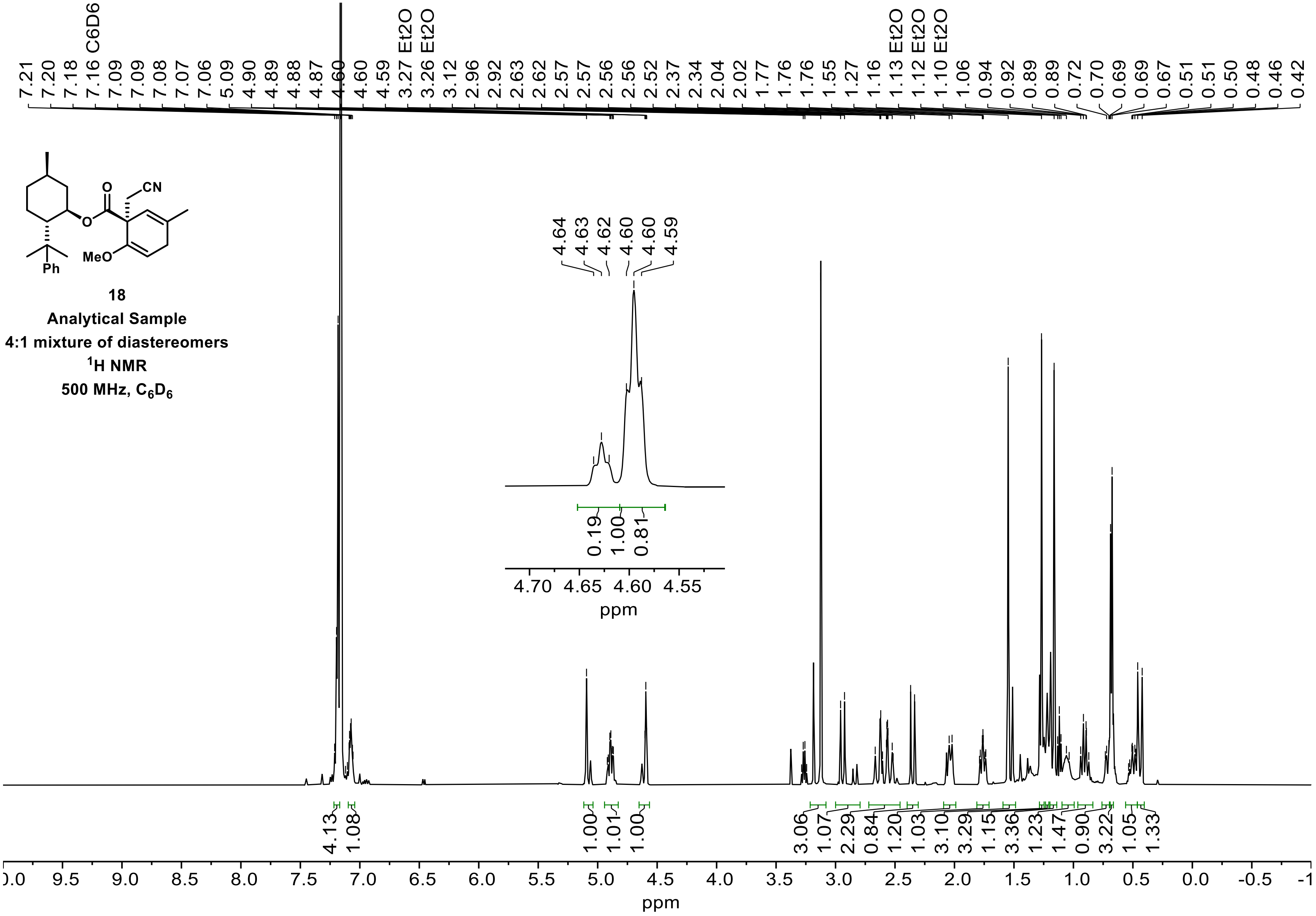

18
Analytical Sample
4:1 mixture of diastereomers
1H NMR
500 MHz, C6D6
ppm

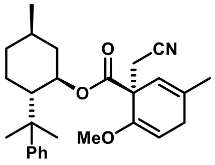

18
4:1 mixture of diastereomers
$^{13}C\{^1H\}$ NMR
126 MHz, $CDCl_3$

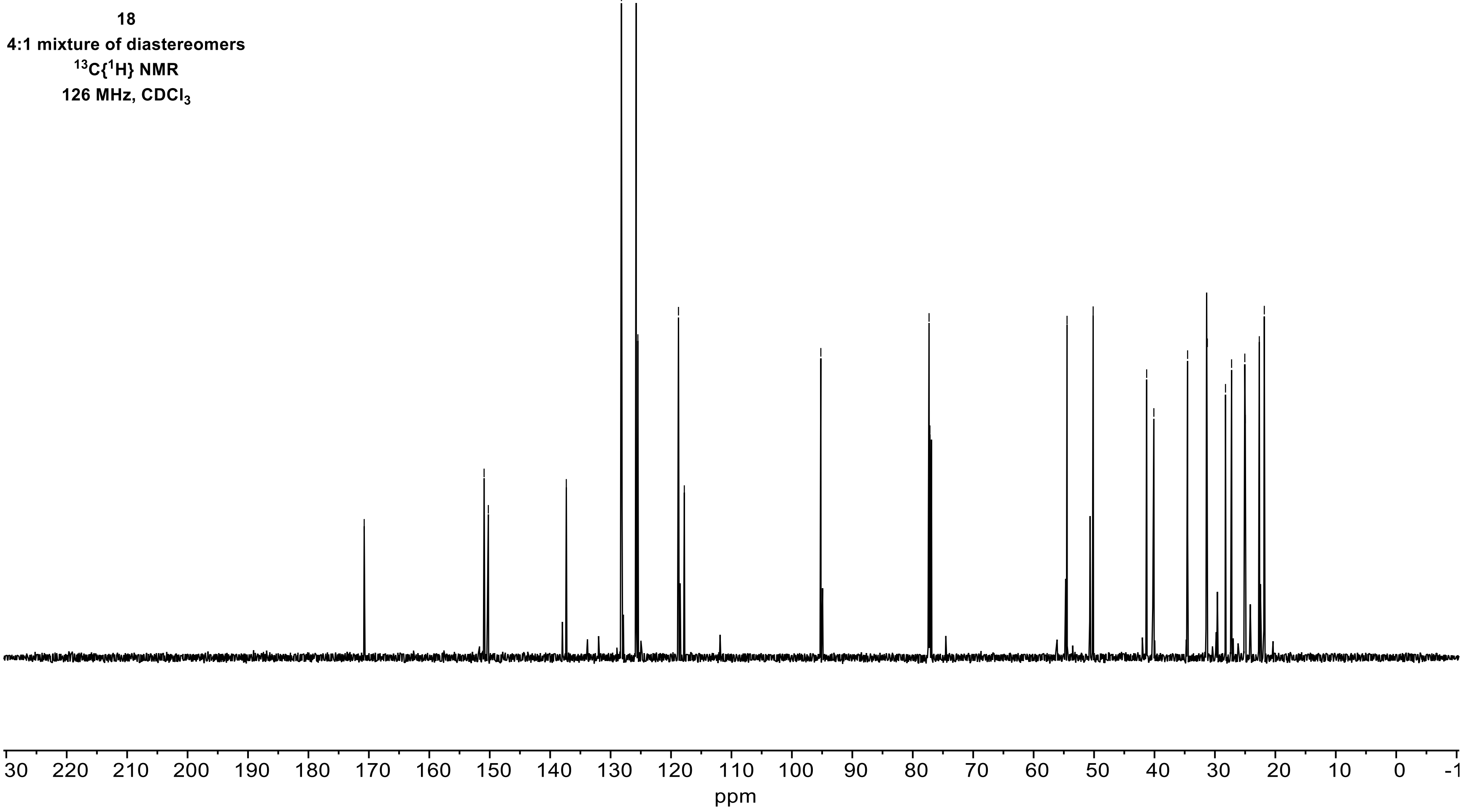

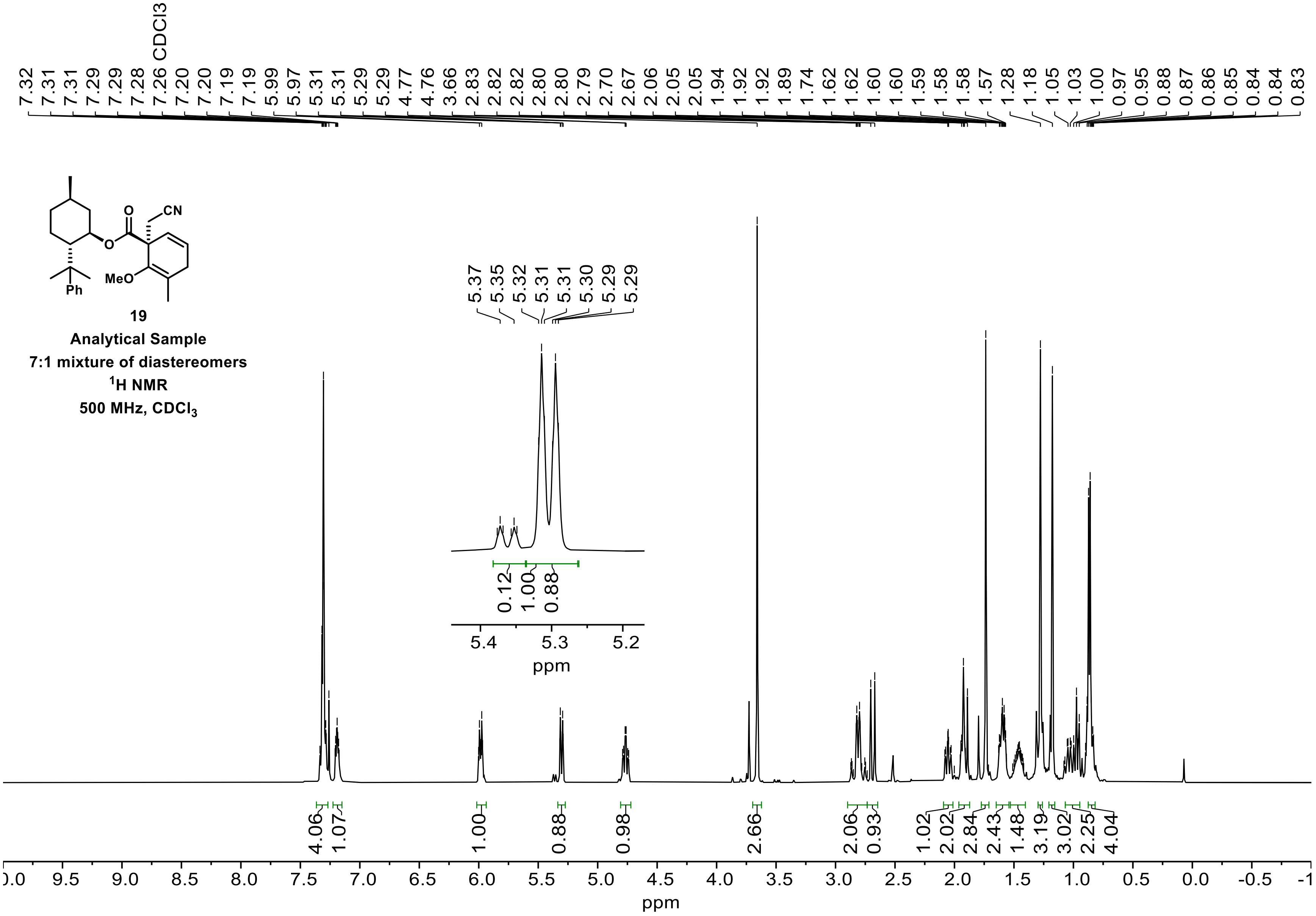
CDCl3
7.32 7.31 7.31 7.29 7.29 7.28 7.26 7.20 7.20 7.19 7.19 5.99 5.97 5.31 5.31 5.29 5.29 4.77 4.76 3.66 2.83 2.82 2.82 2.80 2.80 2.79 2.70 2.67 2.06 2.05 2.05 1.94 1.92 1.92 1.89 1.74 1.62 1.62 1.60 1.60 1.59 1.58 1.58 1.57 1.28 1.18 1.05 1.03 1.00 0.97 0.95 0.88 0.87 0.86 0.85 0.84 0.84 0.83
CN
O
MeO
Ph
19
Analytical Sample
7:1 mixture of diastereomers
1H NMR
500 MHz, CDCl3
5.37 5.35 5.32 5.31 5.31 5.30 5.29 5.29
0.12 1.00 0.88
5.4 5.3 5.2
ppm
4.06 1.07 1.00 0.88 0.98 2.66 2.06 0.93 1.02 2.02 2.84 2.43 1.48 3.19 3.02 2.25 4.04
10.0 9.5 9.0 8.5 8.0 7.5 7.0 6.5 6.0 5.5 5.0 4.5 4.0 3.5 3.0 2.5 2.0 1.5 1.0 0.5 0.0 -0.5 -1
ppm

HN01-91A-5a.4.fid

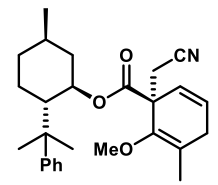

19

7:1 mixture of diastereomers

$^{13}C\{^1H\}$ NMR

151 MHz, $CDCl_3$

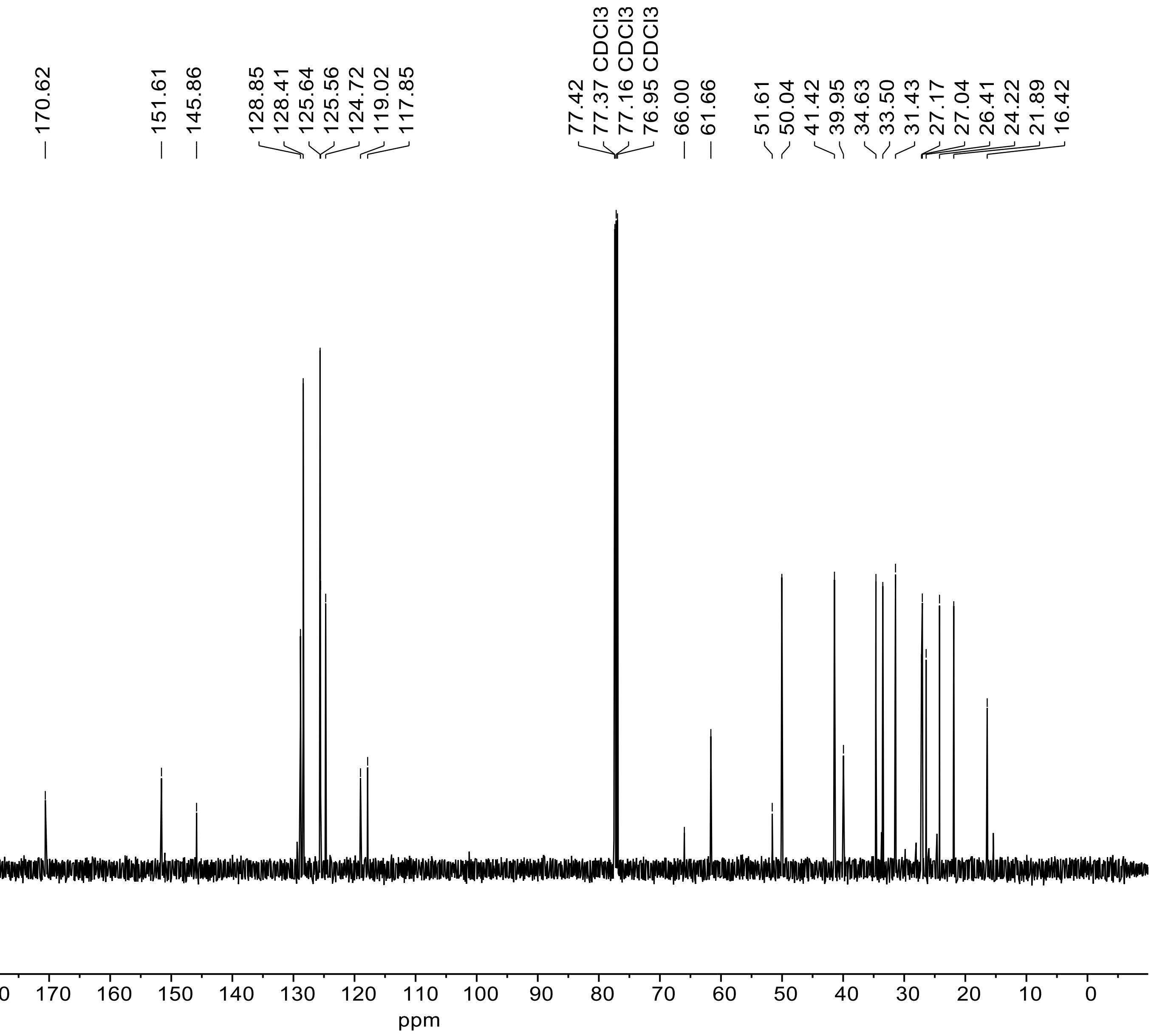

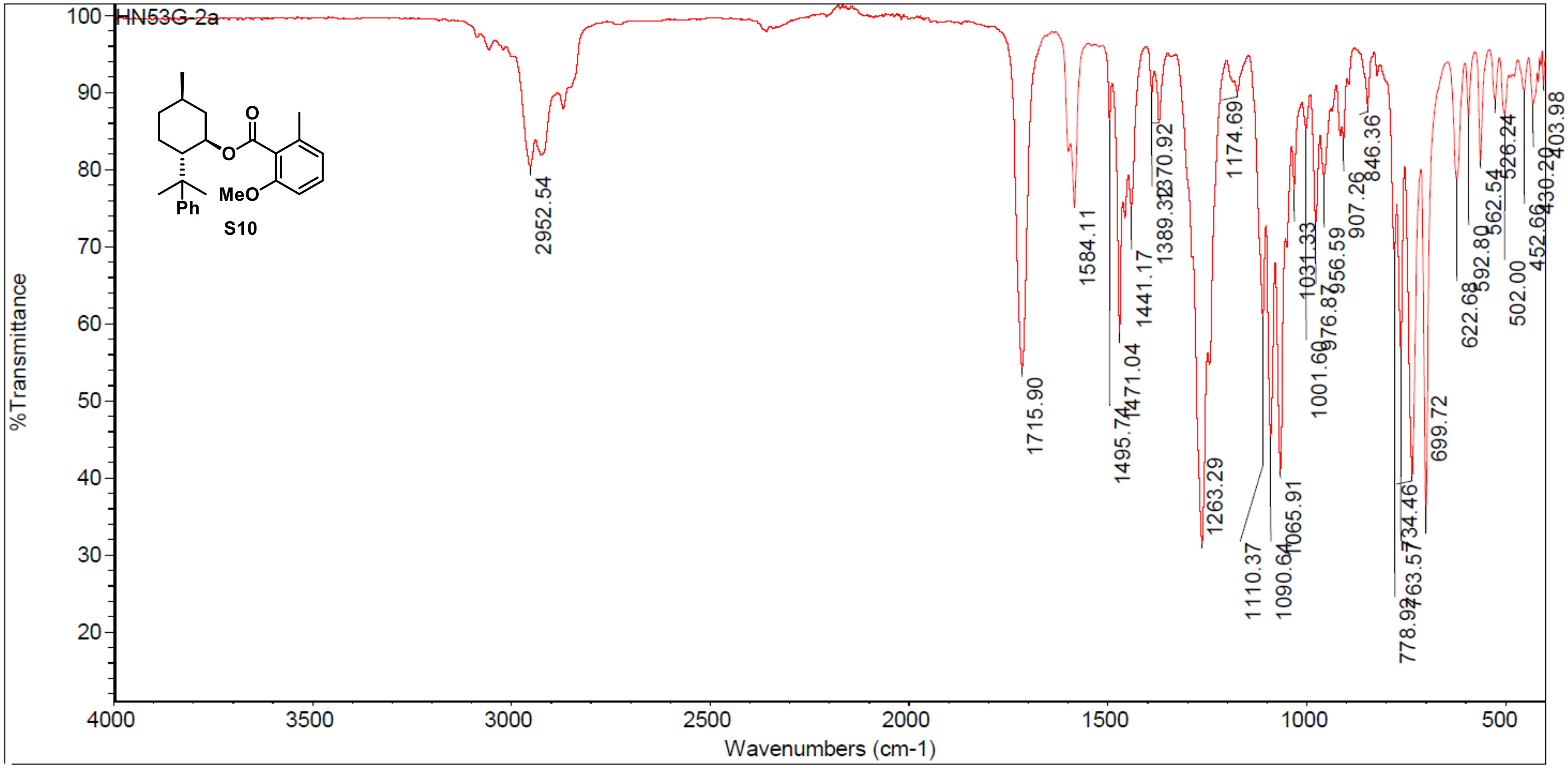
HN53G-2a
S10
MeO
Ph
%Transmittance
Wavenumbers (cm-1)
2952.54
1715.90
1584.11
1495.74
1471.04
1441.17
1389.31
1370.92
1263.29
1174.69
1110.37
1090.64
1065.91
1031.33
1001.60
976.87
956.59
907.26
846.36
778.92
763.57
734.46
699.72
622.68
592.80
562.54
526.24
502.00
452.66
430.29
403.98

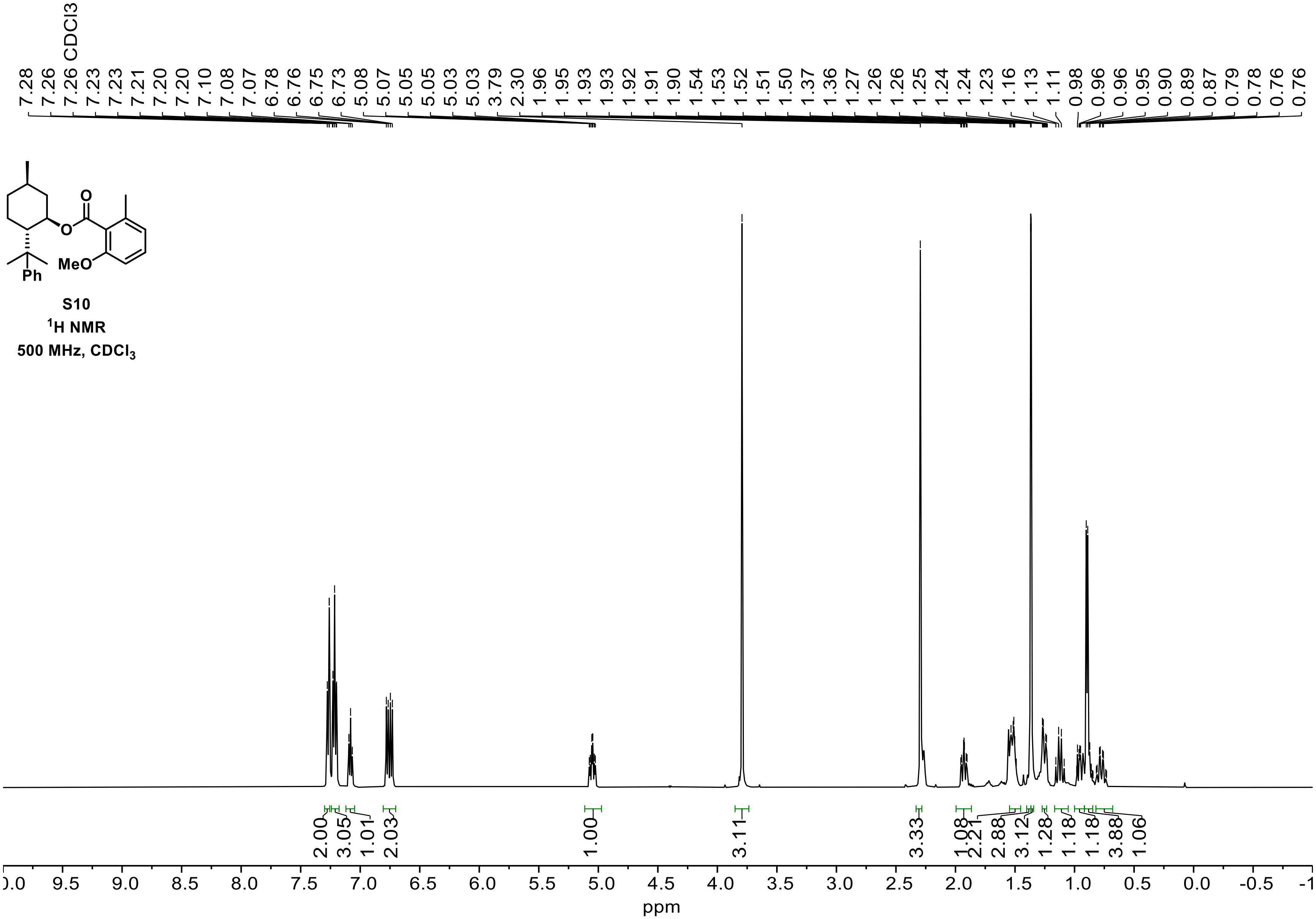
7.28 7.26 7.26 CDCl3 7.23 7.23 7.21 7.20 7.20 7.10 7.08 7.07 6.78 6.76 6.75 6.73 5.08 5.07 5.05 5.05 5.03 5.03 3.79 2.30 1.96 1.95 1.93 1.93 1.92 1.91 1.90 1.54 1.53 1.52 1.51 1.50 1.37 1.36 1.27 1.26 1.26 1.25 1.24 1.24 1.23 1.16 1.13 1.11 0.98 0.96 0.96 0.95 0.90 0.89 0.87 0.79 0.78 0.76 0.76
O
O
MeO
Ph
S10
^{1}H NMR
500 MHz, $CDCl_3$
2.00 3.05 1.01 2.03 1.00 3.11 3.33 1.08 2.21 2.88 3.12 1.28 1.18 1.18 3.88 1.06
10.0 9.5 9.0 8.5 8.0 7.5 7.0 6.5 6.0 5.5 5.0 4.5 4.0 3.5 3.0 2.5 2.0 1.5 1.0 0.5 0.0 -0.5 -1
ppm

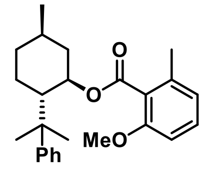

S10
$^{13}C\{^1H\}$ NMR
151 MHz, $CDCl_3$

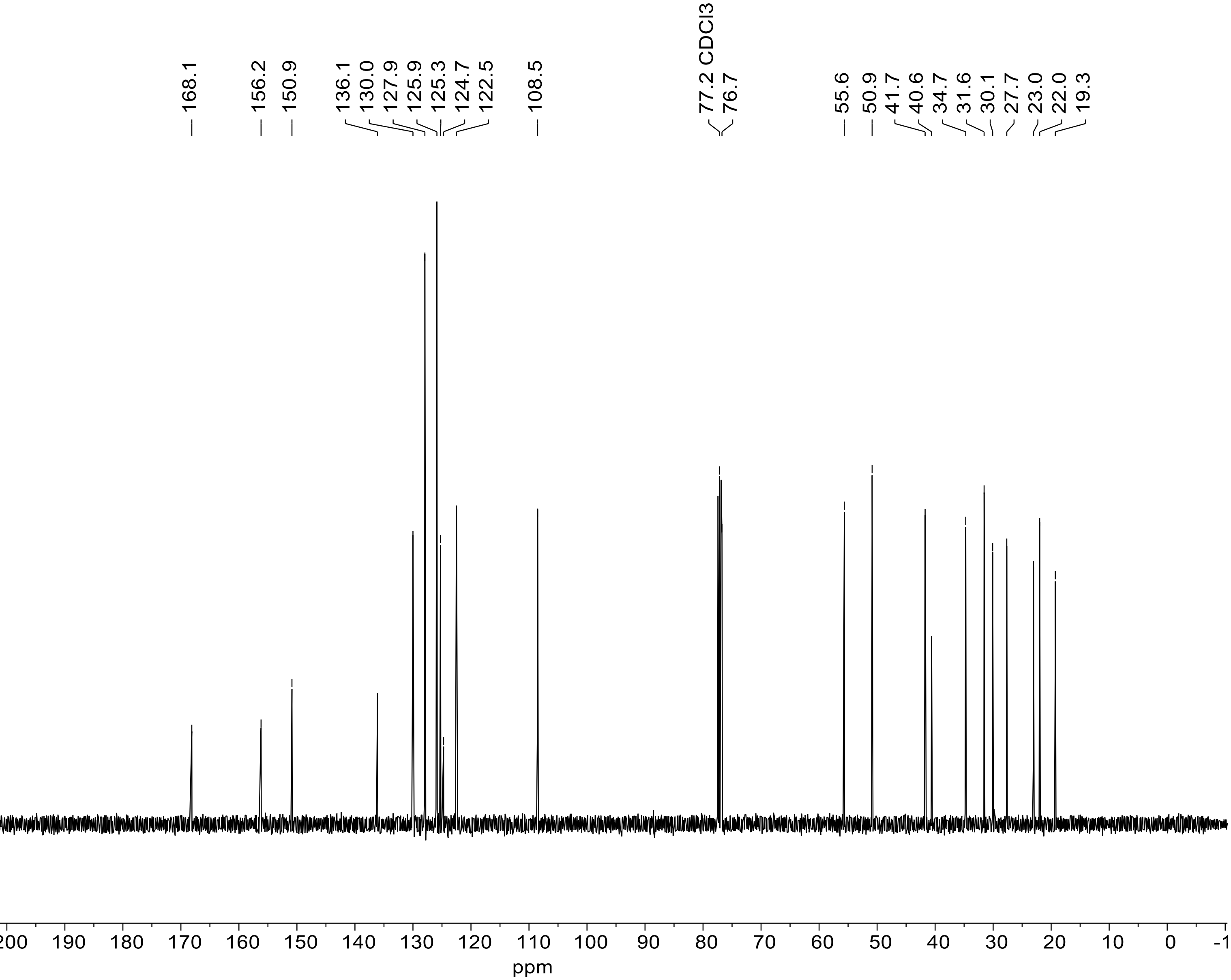

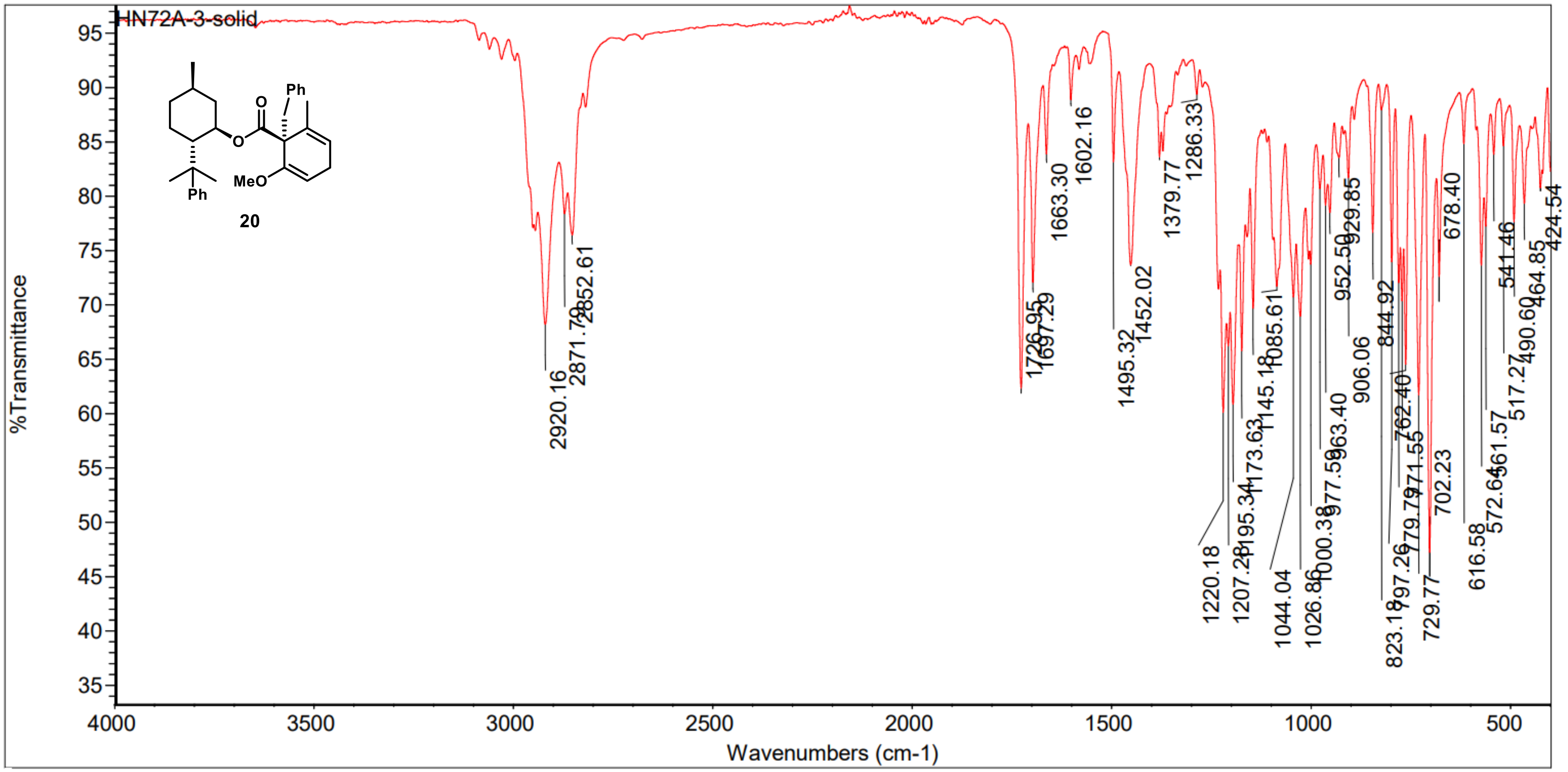
HN72A-3-solid
%Transmittance
Wavenumbers (cm-1)
20
2920.16
2871.79
2852.61
1726.95
1697.29
1663.30
1602.16
1495.32
1452.02
1379.77
1286.33
1220.18
1207.28
1195.34
1173.63
1145.18
1085.61
1044.04
1026.86
1000.38
977.59
963.40
952.50
929.85
906.06
844.92
823.19
797.26
779.79
771.55
762.40
729.77
702.23
678.40
616.58
572.64
561.57
541.46
517.27
490.60
464.85
424.54

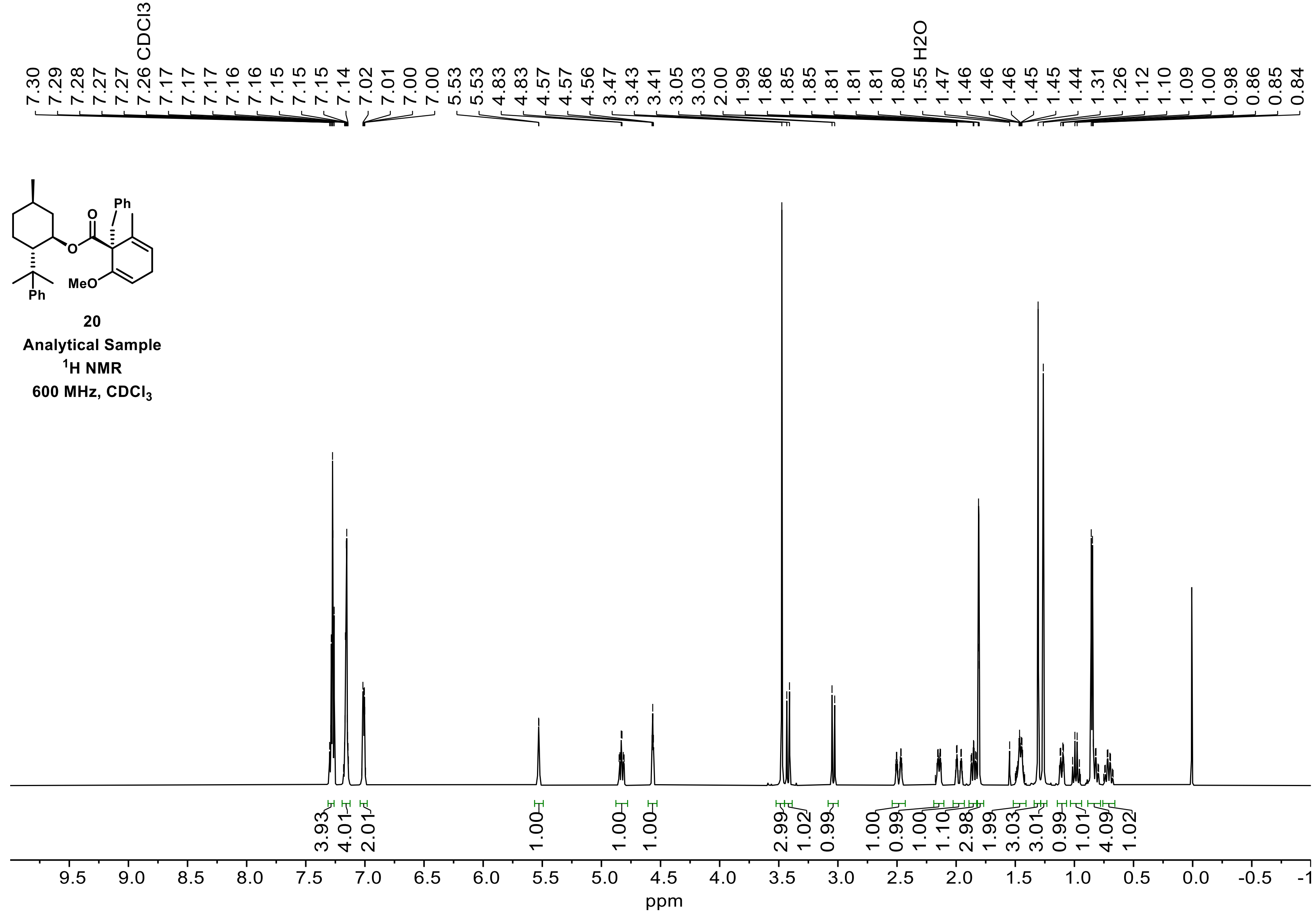
CDCl3
H2O
7.30 7.29 7.28 7.27 7.27 7.26 7.17 7.17 7.17 7.16 7.16 7.15 7.15 7.15 7.14 7.02 7.01 7.00 7.00 5.53 5.53 4.83 4.83 4.57 4.57 4.56 3.47 3.43 3.41 3.05 3.03 2.00 1.99 1.86 1.85 1.85 1.81 1.81 1.81 1.80 1.55 1.47 1.46 1.46 1.45 1.45 1.44 1.31 1.26 1.12 1.10 1.09 1.00 0.98 0.86 0.85 0.84
Ph
O
O
MeO
Ph
20
Analytical Sample
1H NMR
600 MHz, CDCl3
3.93 4.01 2.01 1.00 1.00 1.00 2.99 1.02 0.99 1.00 0.99 1.00 1.10 2.98 1.99 3.03 3.01 0.99 1.01 4.09 1.02
9.5 9.0 8.5 8.0 7.5 7.0 6.5 6.0 5.5 5.0 4.5 4.0 3.5 3.0 2.5 2.0 1.5 1.0 0.5 0.0 -0.5 -1
ppm

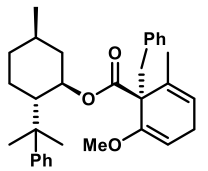

20

$^{13}C\{^1H\}$ NMR

151 MHz, $CDCl_3$

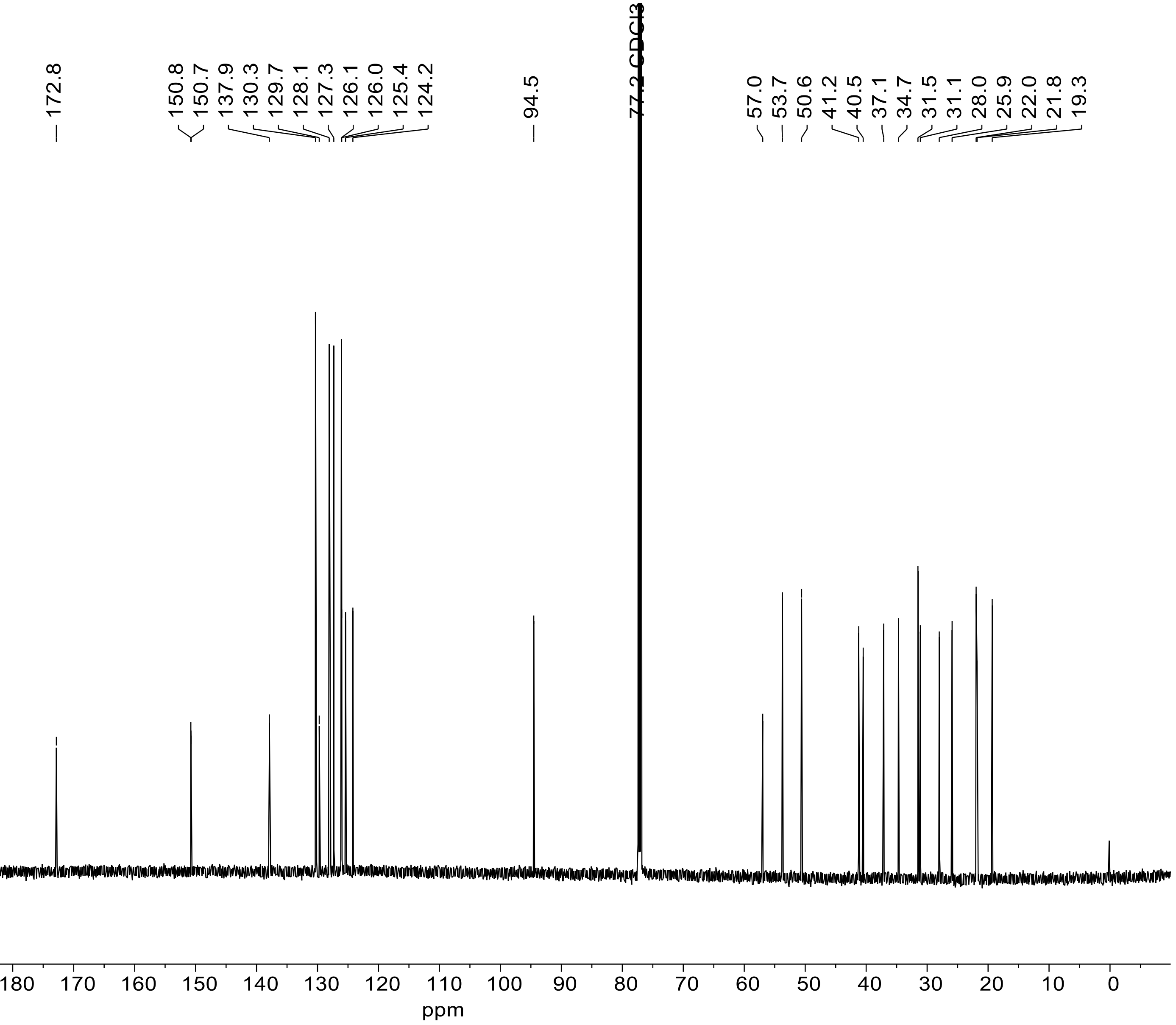

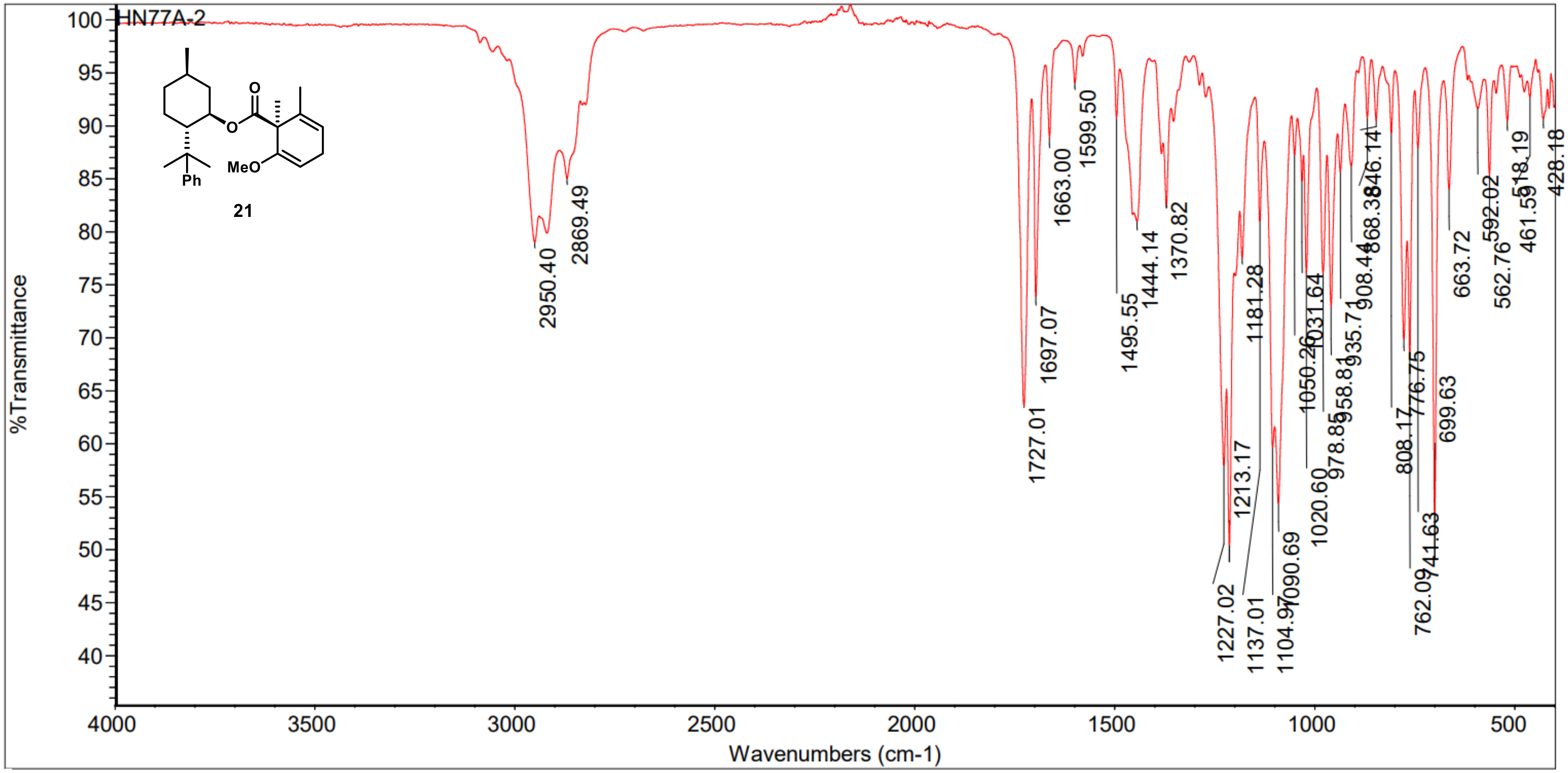
HN77A-2
21
MeO
Ph
%Transmittance
Wavenumbers (cm-1)
2950.40
2869.49
1727.01
1697.07
1663.00
1599.50
1495.55
1444.14
1370.82
1227.02
1213.17
1181.28
1137.01
1104.97
1090.69
1050.26
1031.64
1020.60
978.85
958.81
935.71
908.44
868.38
846.14
808.17
776.75
762.09
741.63
699.63
663.72
592.02
562.76
518.19
461.59
428.18

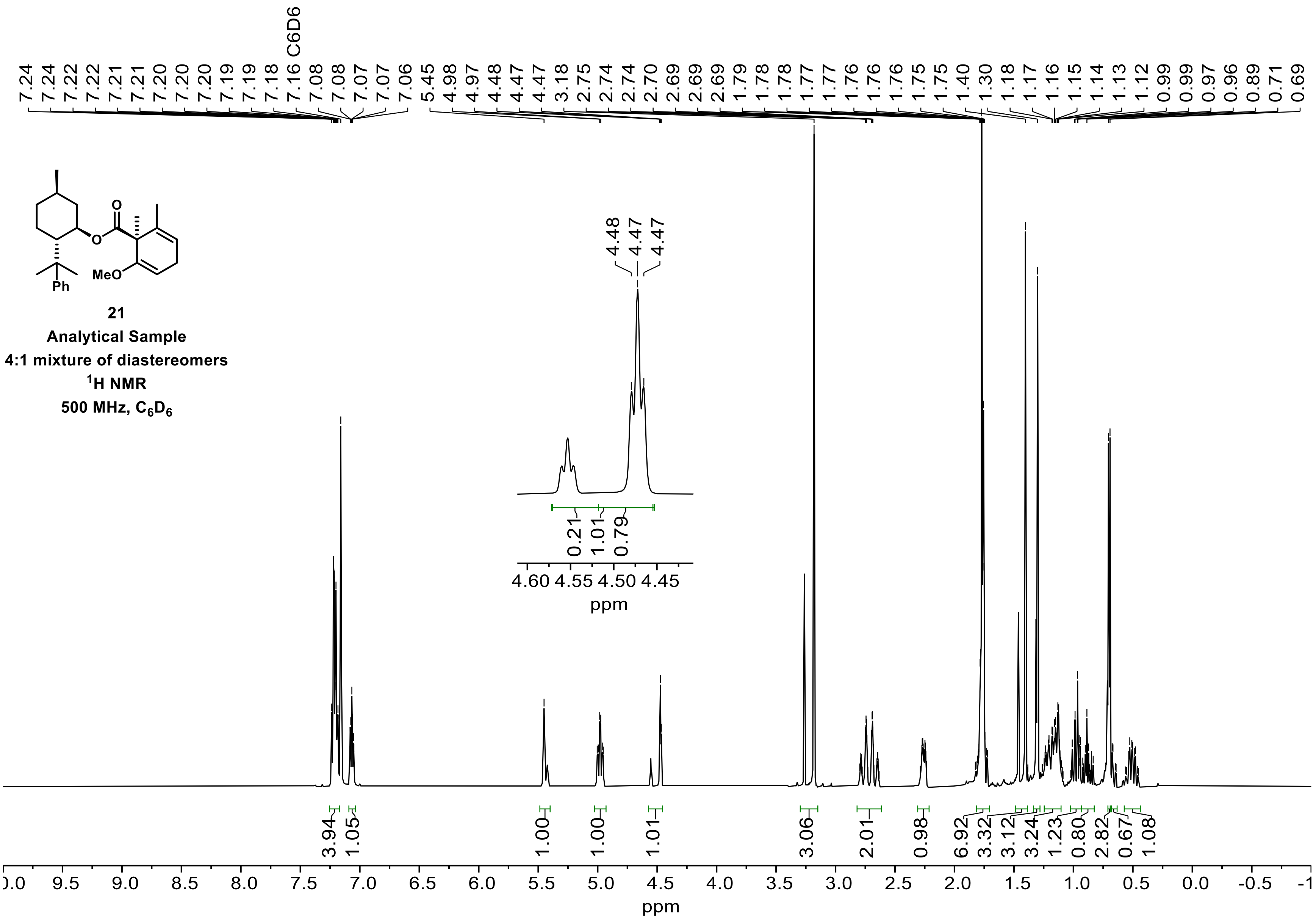

O
MeO
Ph
21
Analytical Sample
4:1 mixture of diastereomers
^{1}H NMR
500 MHz, C_6D_6
C6D6
7.24 7.24 7.22 7.22 7.21 7.21 7.20 7.20 7.20 7.19 7.19 7.18 7.16 7.08 7.08 7.07 7.07 7.06 5.45 4.98 4.97 4.48 4.47 4.47 3.18 2.75 2.74 2.74 2.70 2.69 2.69 2.69 1.79 1.78 1.78 1.77 1.77 1.77 1.76 1.76 1.76 1.75 1.75 1.40 1.30 1.18 1.17 1.16 1.15 1.14 1.13 1.12 0.99 0.99 0.97 0.96 0.89 0.71 0.69
4.48 4.47 4.47
0.21 1.01 0.79
4.60 4.55 4.50 4.45
ppm
3.94 1.05 1.00 1.00 1.01 3.06 2.01 0.98 6.92 3.32 3.12 3.24 1.23 0.80 2.82 0.67 1.08
9.5 9.0 8.5 8.0 7.5 7.0 6.5 6.0 5.5 5.0 4.5 4.0 3.5 3.0 2.5 2.0 1.5 1.0 0.5 0.0 -0.5
ppm

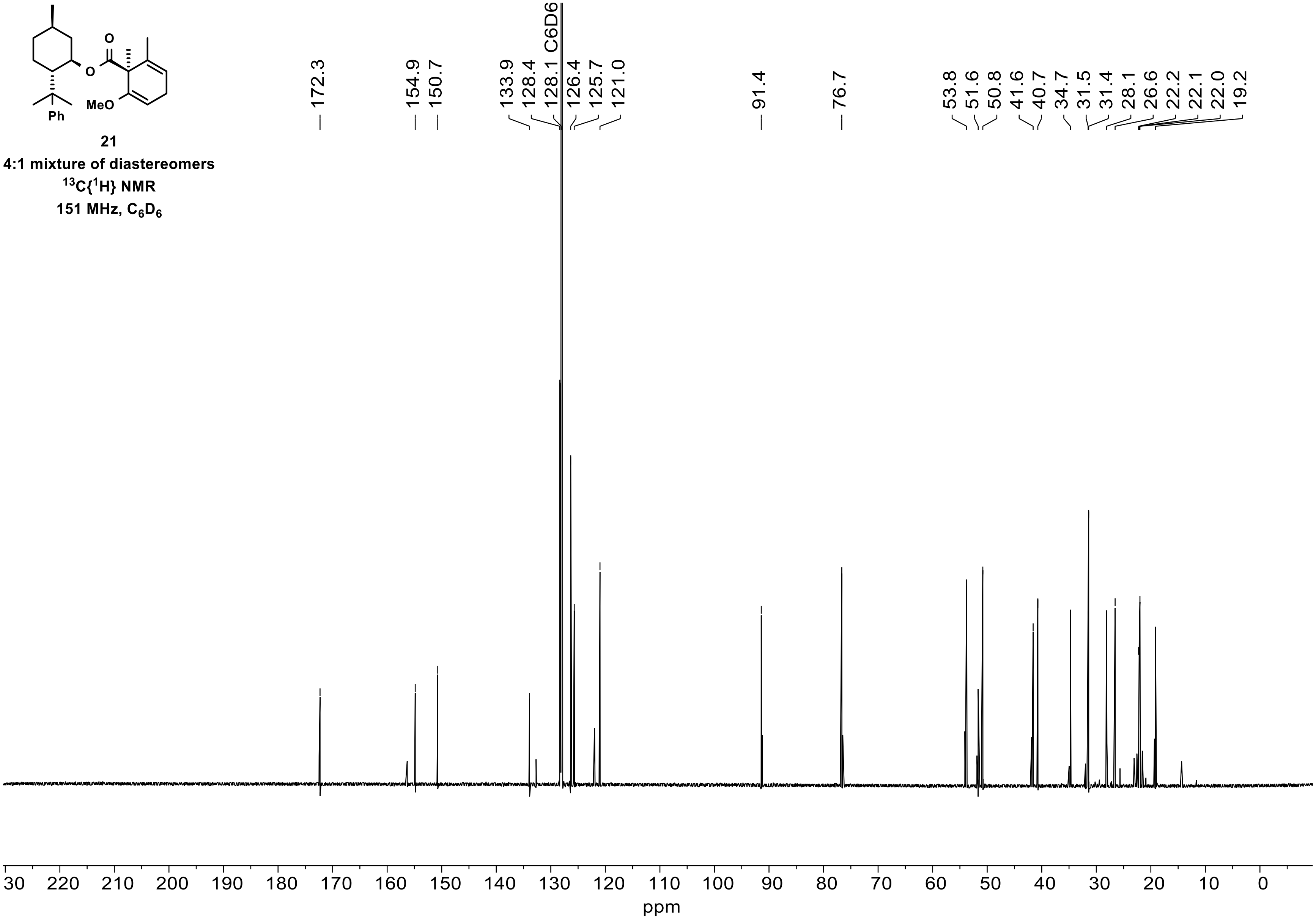

Ph
MeO
O
O
21
4:1 mixture of diastereomers
$^{13}C\{^1H\}$ NMR
151 MHz, C_6D_6
172.3
154.9
150.7
133.9
128.4
128.1 C6D6
126.4
125.7
121.0
91.4
76.7
53.8
51.6
50.8
41.6
40.7
34.7
31.5
31.4
28.1
26.6
22.2
22.1
22.0
19.2
30 220 210 200 190 180 170 160 150 140 130 120 110 100 90 80 70 60 50 40 30 20 10 0
ppm

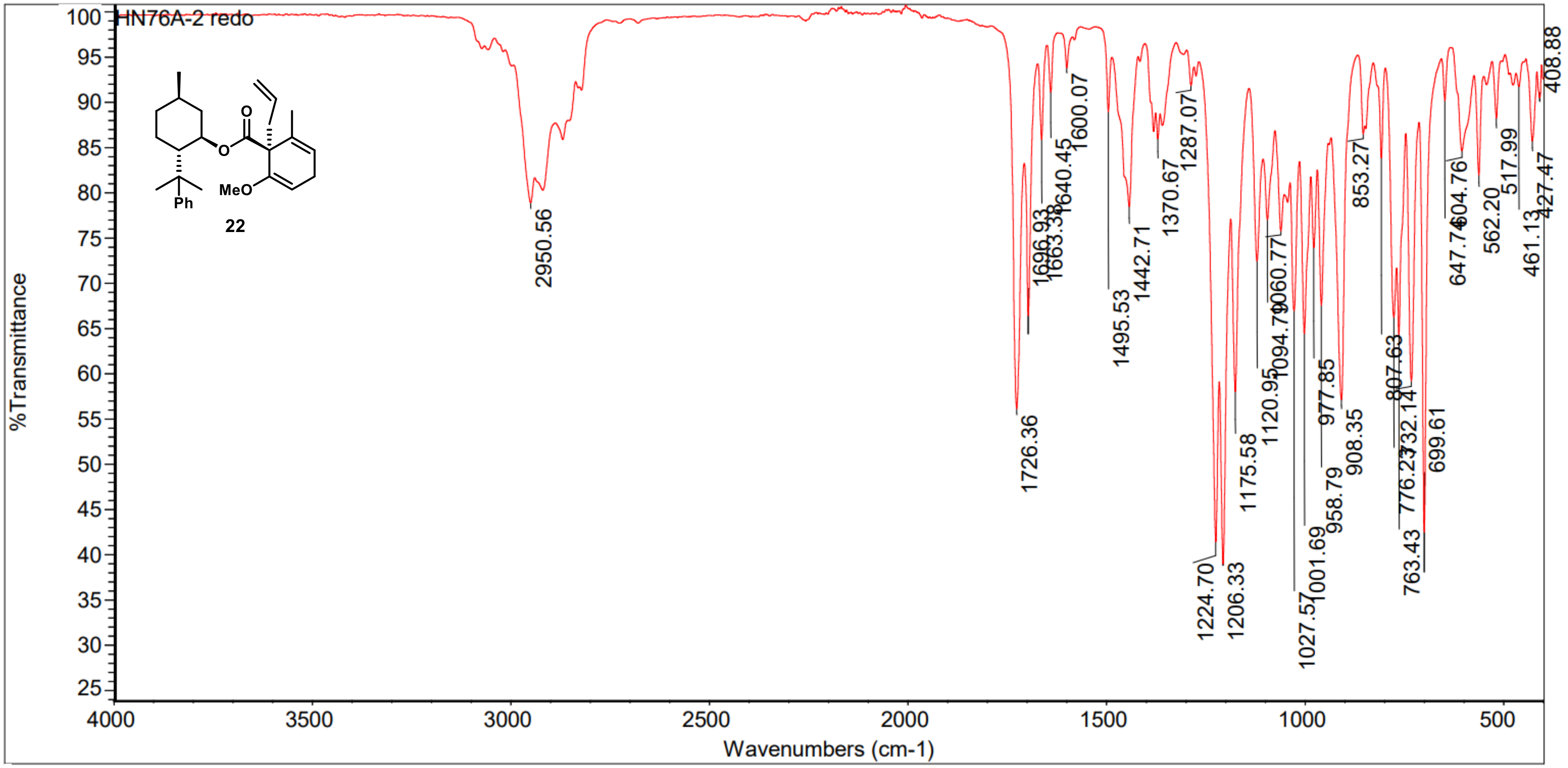
HN76A-2 redo
MeO
Ph
O
22
%Transmittance
Wavenumbers (cm-1)
2950.56
1726.36
1696.93
1663.38
1640.45
1600.07
1495.53
1442.71
1370.67
1287.07
1224.70
1206.33
1175.58
1120.95
1094.79
1060.77
1027.57
1001.69
977.85
958.79
908.35
853.27
807.63
776.23
763.43
732.14
699.61
647.74
604.76
562.20
517.99
461.13
427.47
408.88

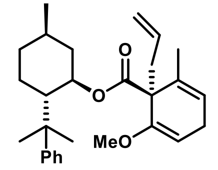

22
Analytical Sample
^{1}H NMR
500 MHz, $CDCl_3$

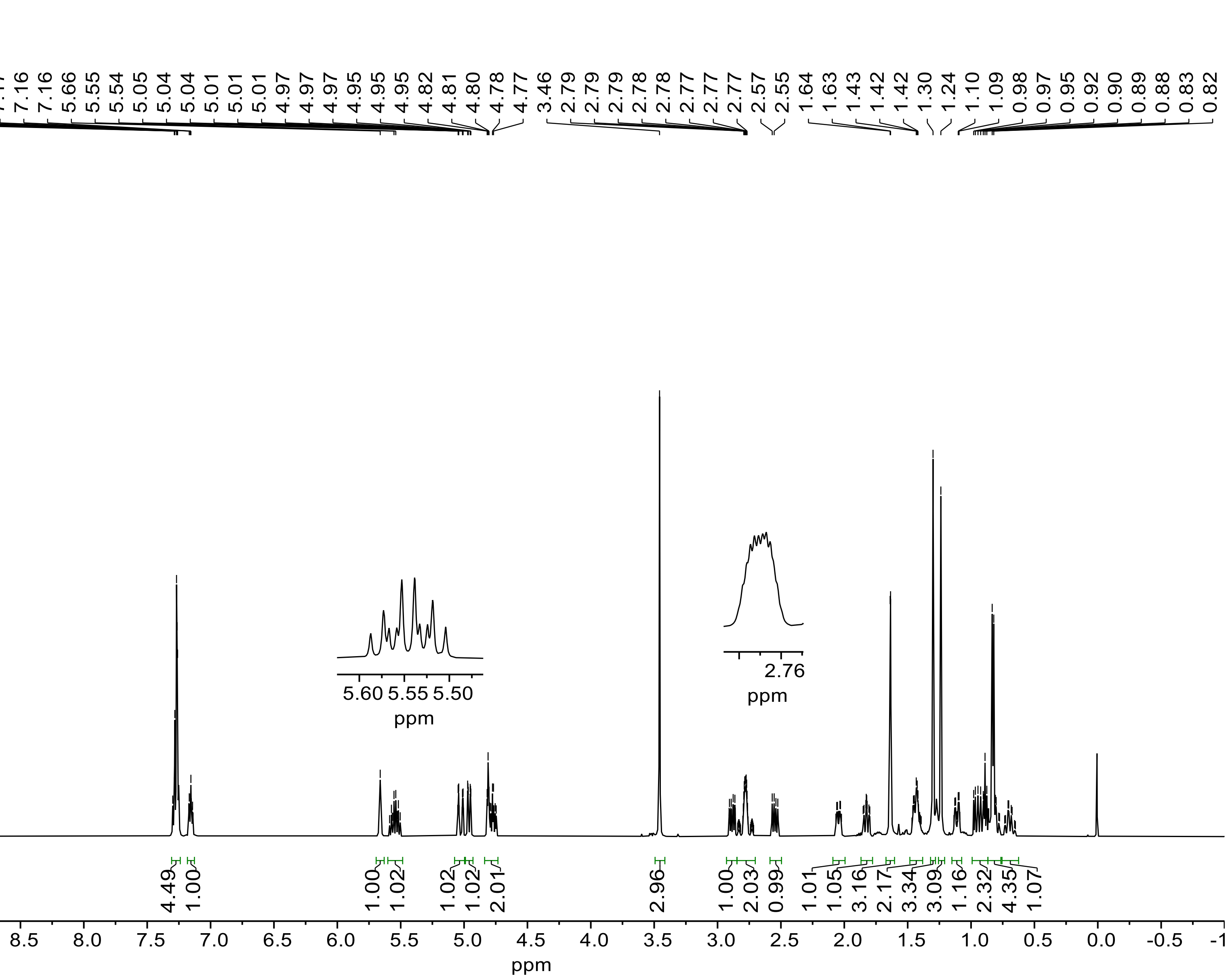

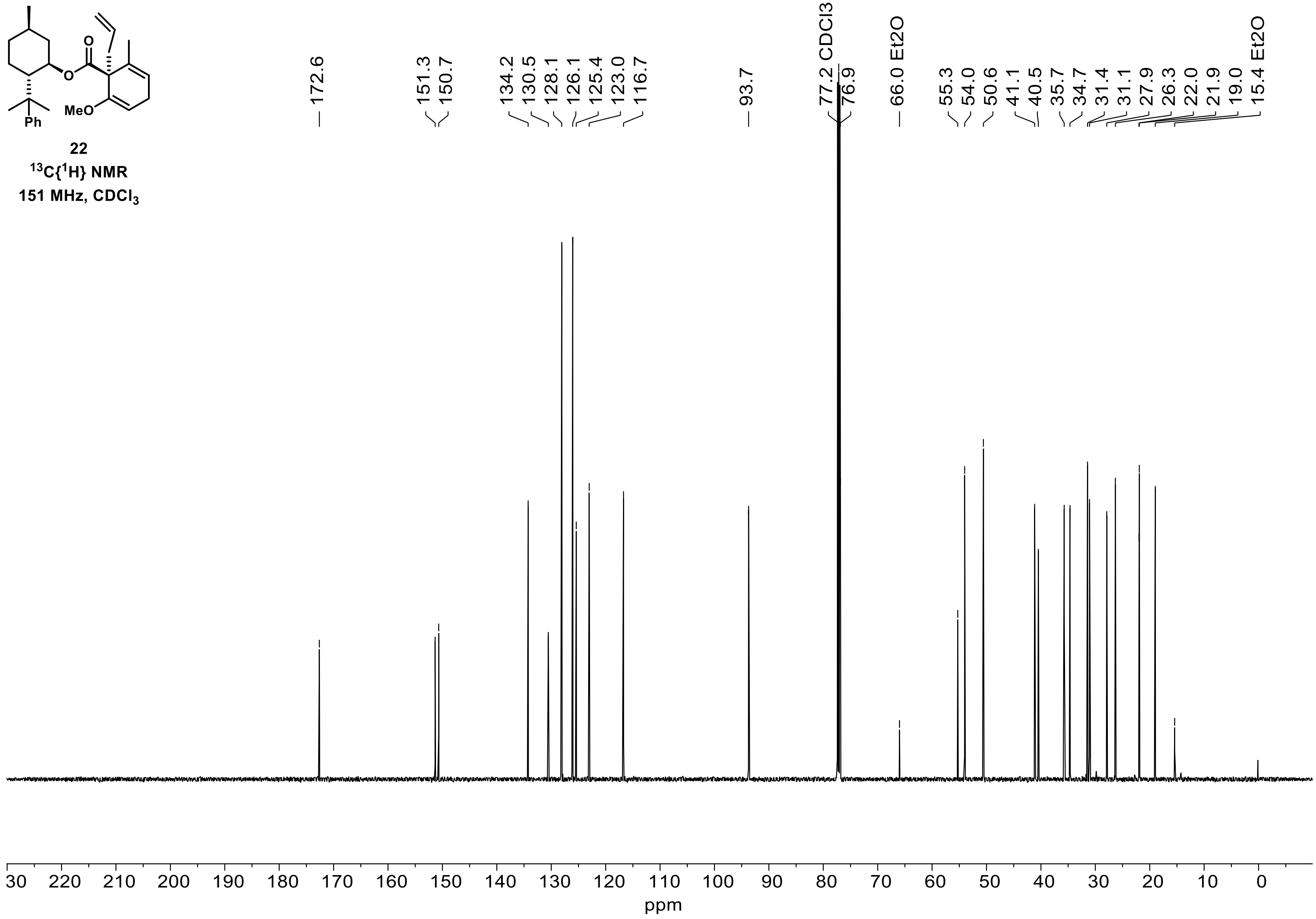
MeO
Ph
O
O
22
$^{13}C\{^{1}H\}$ NMR
151 MHz, $CDCl_3$
172.6
151.3
150.7
134.2
130.5
128.1
126.1
125.4
123.0
116.7
93.7
77.2 CDCl3
76.9
66.0 Et2O
55.3
54.0
50.6
41.1
40.5
35.7
34.7
31.4
31.1
27.9
26.3
22.0
21.9
19.0
15.4 Et2O
30 220 210 200 190 180 170 160 150 140 130 120 110 100 90 80 70 60 50 40 30 20 10 0
ppm

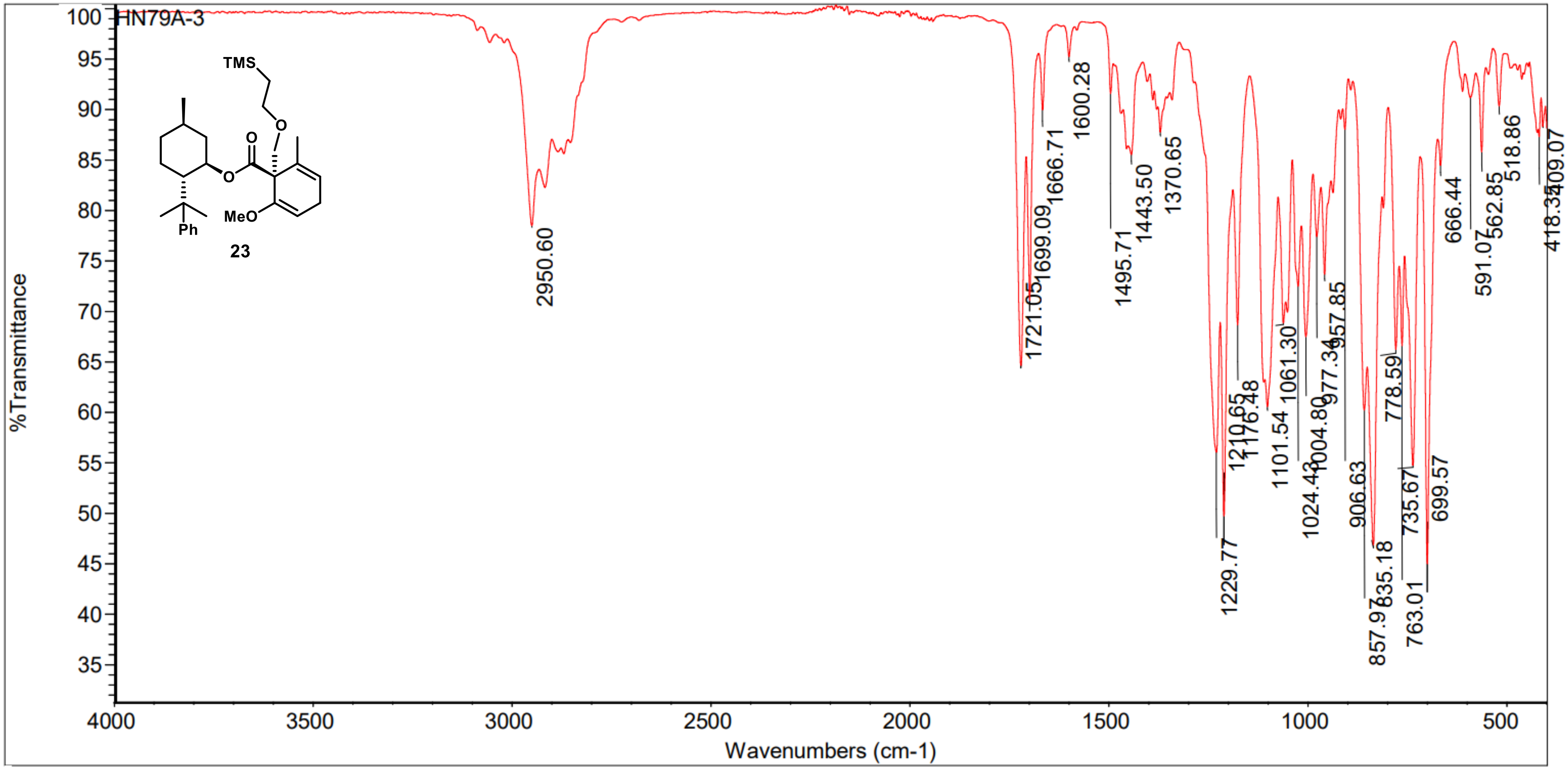
HN79A-3
TMS
O
MeO
Ph
23
%Transmittance
Wavenumbers (cm-1)
2950.60
1721.05
1699.09
1666.71
1600.28
1495.71
1443.50
1370.65
1229.77
1210.65
1176.48
1101.54
1061.30
1024.43
1004.80
977.34
957.85
906.63
857.97
835.18
778.59
763.01
735.67
699.57
666.44
591.07
562.85
518.86
418.34
409.07

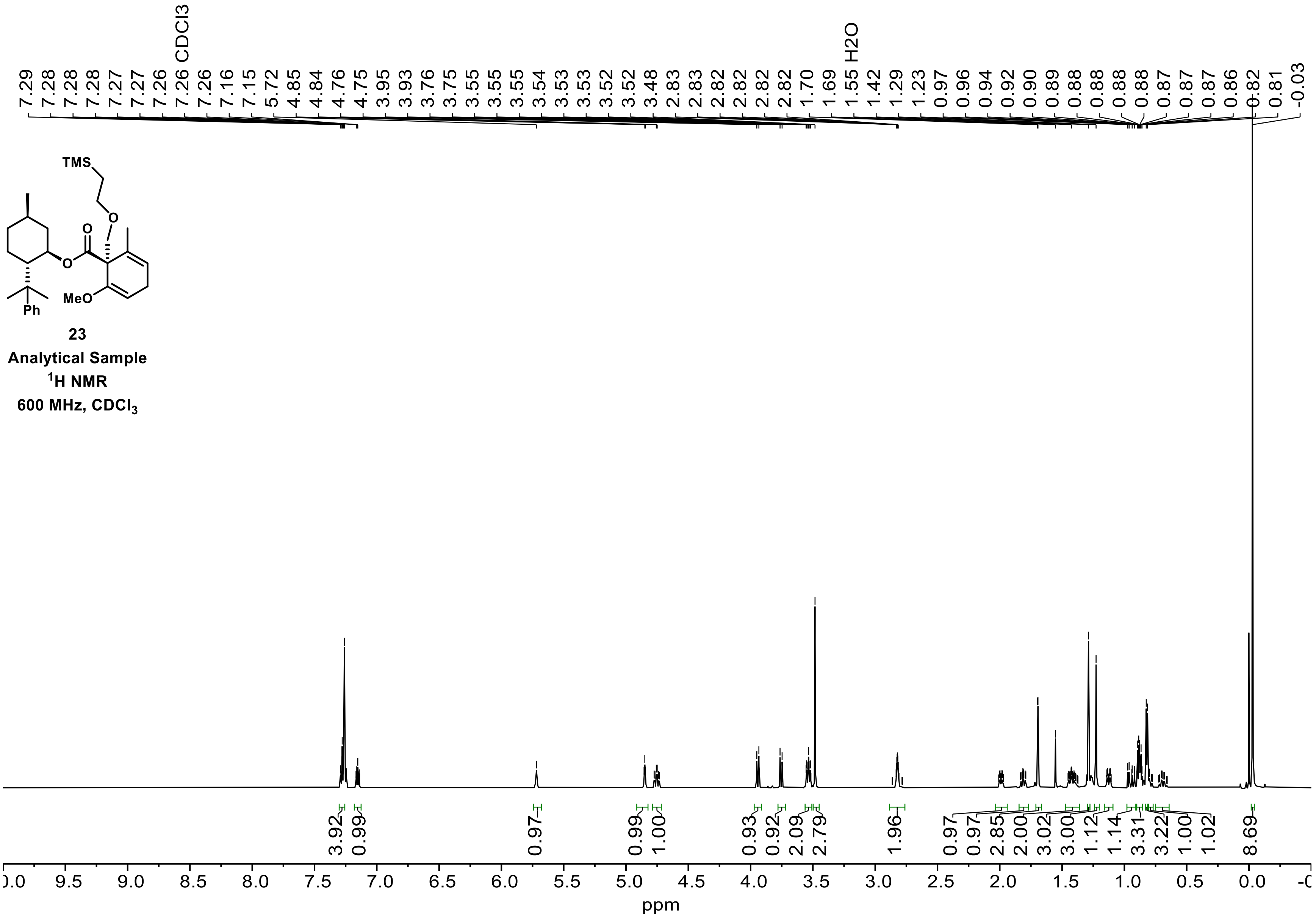
TMS
O
O
O
MeO
Ph
23
Analytical Sample
1H NMR
600 MHz, CDCl3
CDCl3
H2O
7.29 7.28 7.28 7.28 7.27 7.27 7.26 7.26 7.26 7.16 7.15 5.72 4.85 4.84 4.76 4.75 3.95 3.93 3.76 3.75 3.55 3.55 3.55 3.54 3.53 3.53 3.52 3.52 3.48 2.83 2.83 2.82 2.82 2.82 2.82 1.70 1.69 1.55 1.42 1.29 1.23 0.97 0.96 0.94 0.92 0.90 0.89 0.88 0.88 0.88 0.88 0.87 0.87 0.87 0.86 0.82 0.81 -0.03
3.92 0.99 0.97 0.99 1.00 0.93 0.92 2.09 2.79 1.96 0.97 0.97 2.85 2.00 3.02 3.00 1.12 1.14 3.31 3.22 1.00 1.02 8.69
ppm

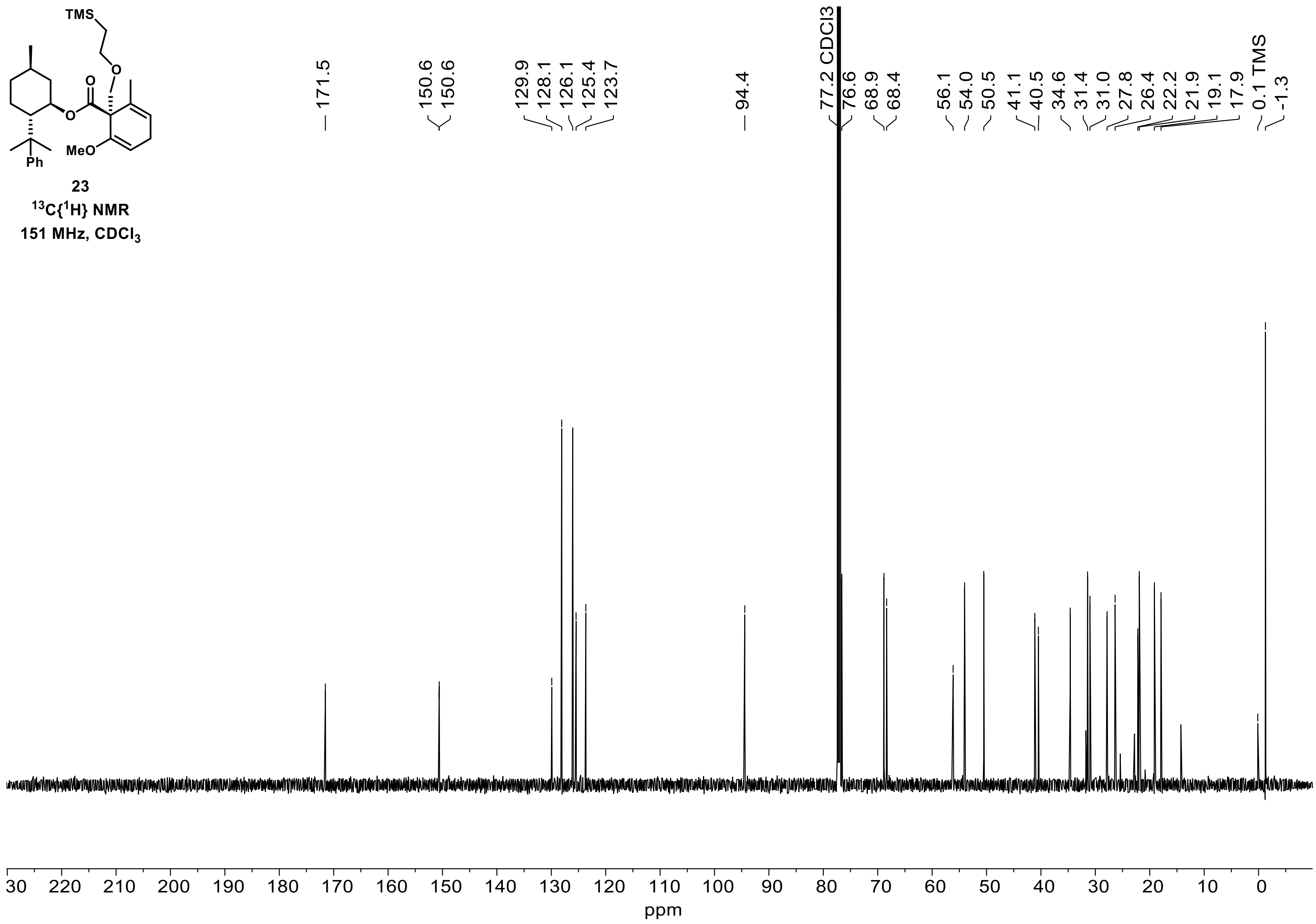
TMS
O
O
MeO
Ph
23
13C{1H} NMR
151 MHz, CDCl3
171.5
150.6
150.6
129.9
128.1
126.1
125.4
123.7
94.4
77.2 CDCl3
76.6
68.9
68.4
56.1
54.0
50.5
41.1
40.5
34.6
31.4
31.0
27.8
26.4
22.2
21.9
19.1
17.9
0.1 TMS
-1.3
ppm

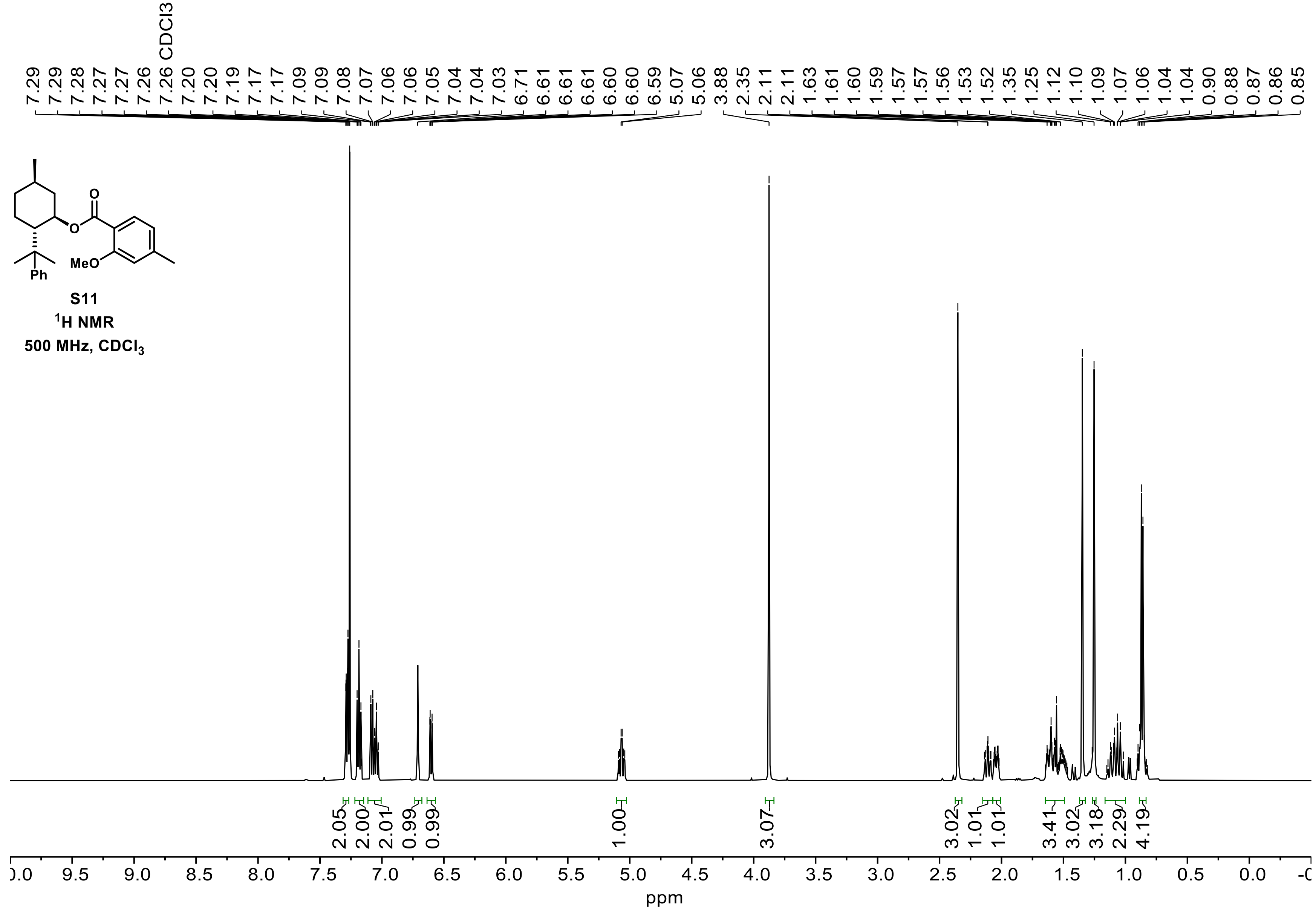
MeO
O
Ph
S11
^{1}H NMR
500 MHz, $CDCl_3$
7.29 7.29 7.28 7.27 7.27 7.26 7.26 CDCl3 7.20 7.20 7.19 7.17 7.17 7.09 7.09 7.08 7.07 7.06 7.06 7.05 7.04 7.04 7.03 6.71 6.61 6.61 6.61 6.60 6.60 6.59 5.07 5.06 3.88 2.35 2.11 2.11 1.63 1.61 1.60 1.59 1.57 1.57 1.56 1.53 1.52 1.35 1.25 1.12 1.10 1.09 1.07 1.06 1.04 1.04 0.90 0.88 0.87 0.86 0.85
2.05 2.00 2.01 0.99 0.99 1.00 3.07 3.02 1.01 1.01 3.41 3.02 3.18 2.29 4.19
9.5 9.0 8.5 8.0 7.5 7.0 6.5 6.0 5.5 5.0 4.5 4.0 3.5 3.0 2.5 2.0 1.5 1.0 0.5 0.0
ppm

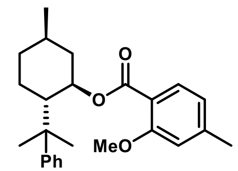

S11
$^{13}C\{1H\}$ NMR
126 MHz, $CDCl_3$

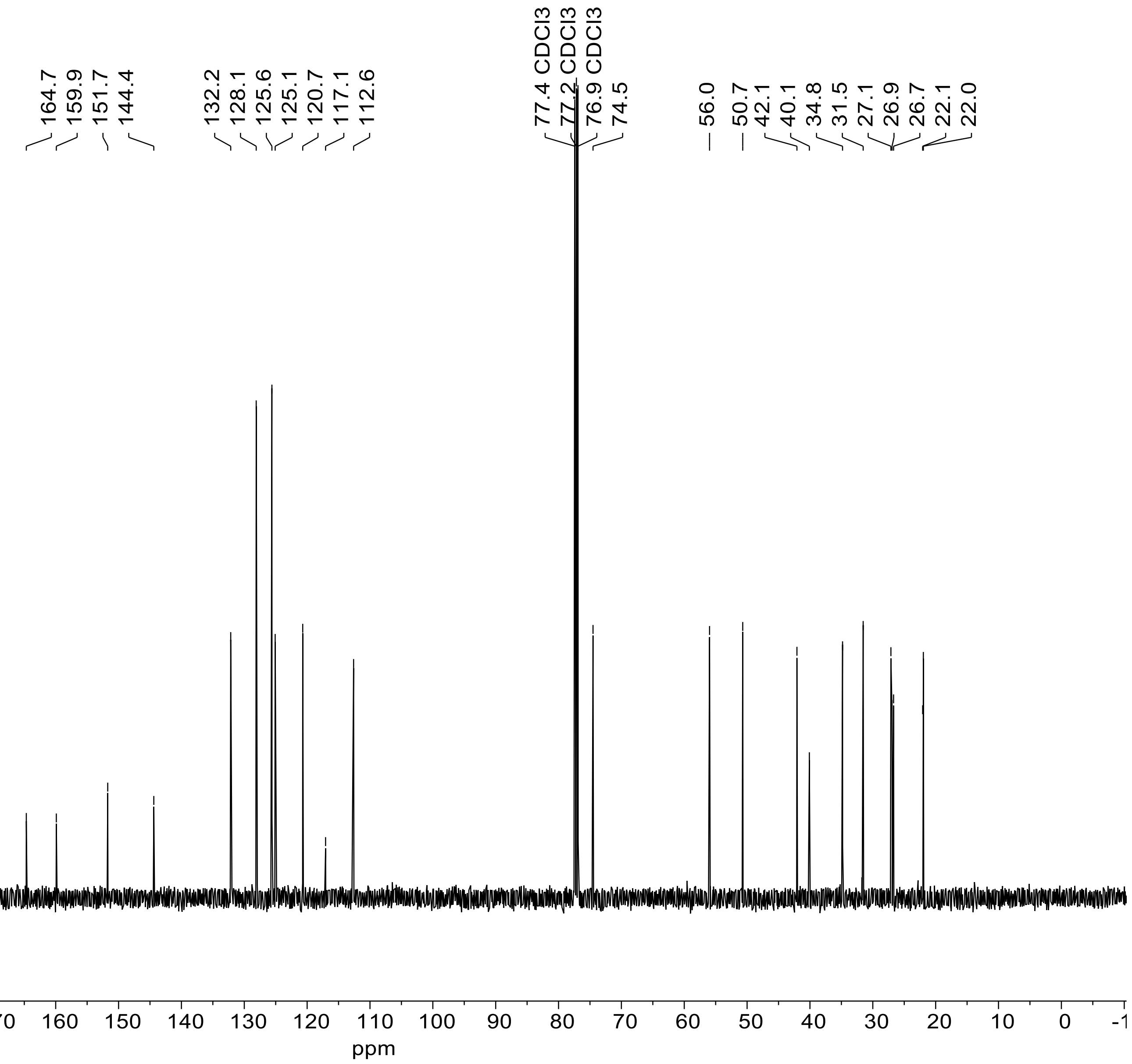

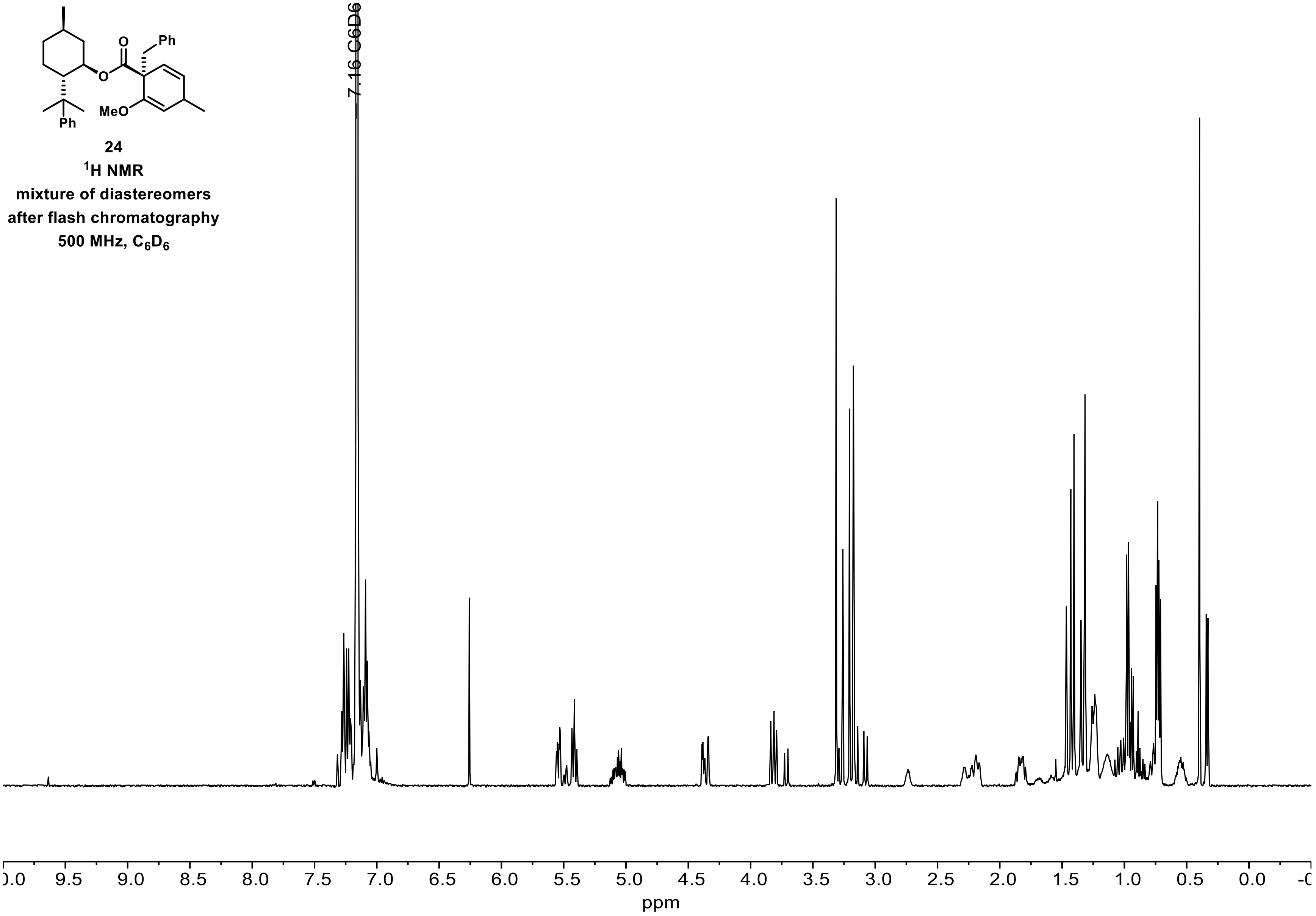
Ph
O
O
MeO
Ph
24
1H NMR
mixture of diastereomers
after flash chromatography
500 MHz, C6D6
7.16 C6D6
ppm
9.5
9.0
8.5
8.0
7.5
7.0
6.5
6.0
5.5
5.0
4.5
4.0
3.5
3.0
2.5
2.0
1.5
1.0
0.5
0.0

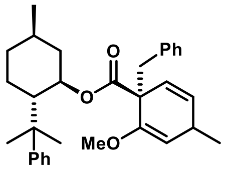

24

$^{13}C\{1H\}$ NMR

mixture of diastereomers

after flash chromatography

151 MHz, C_6D_6

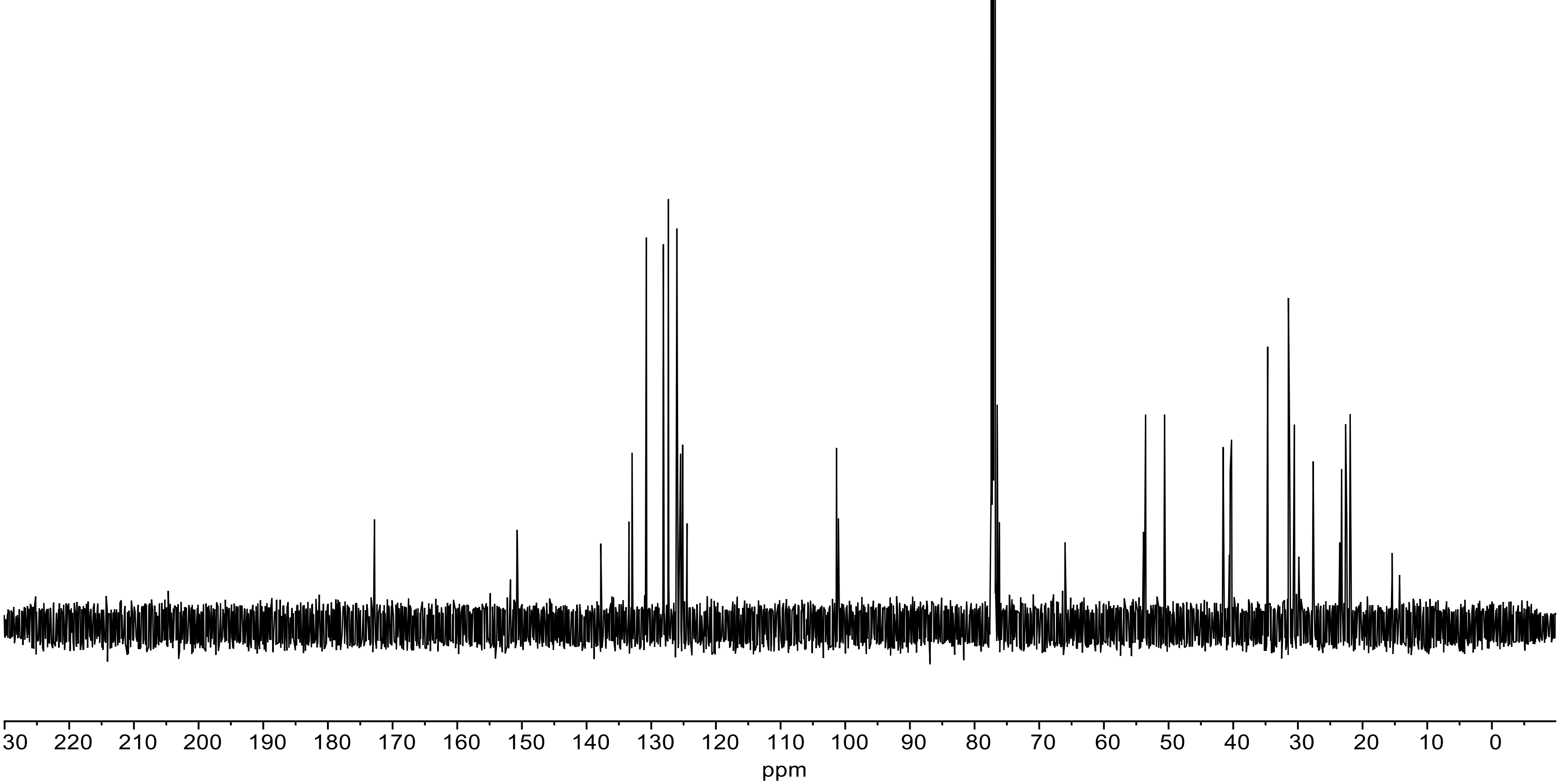

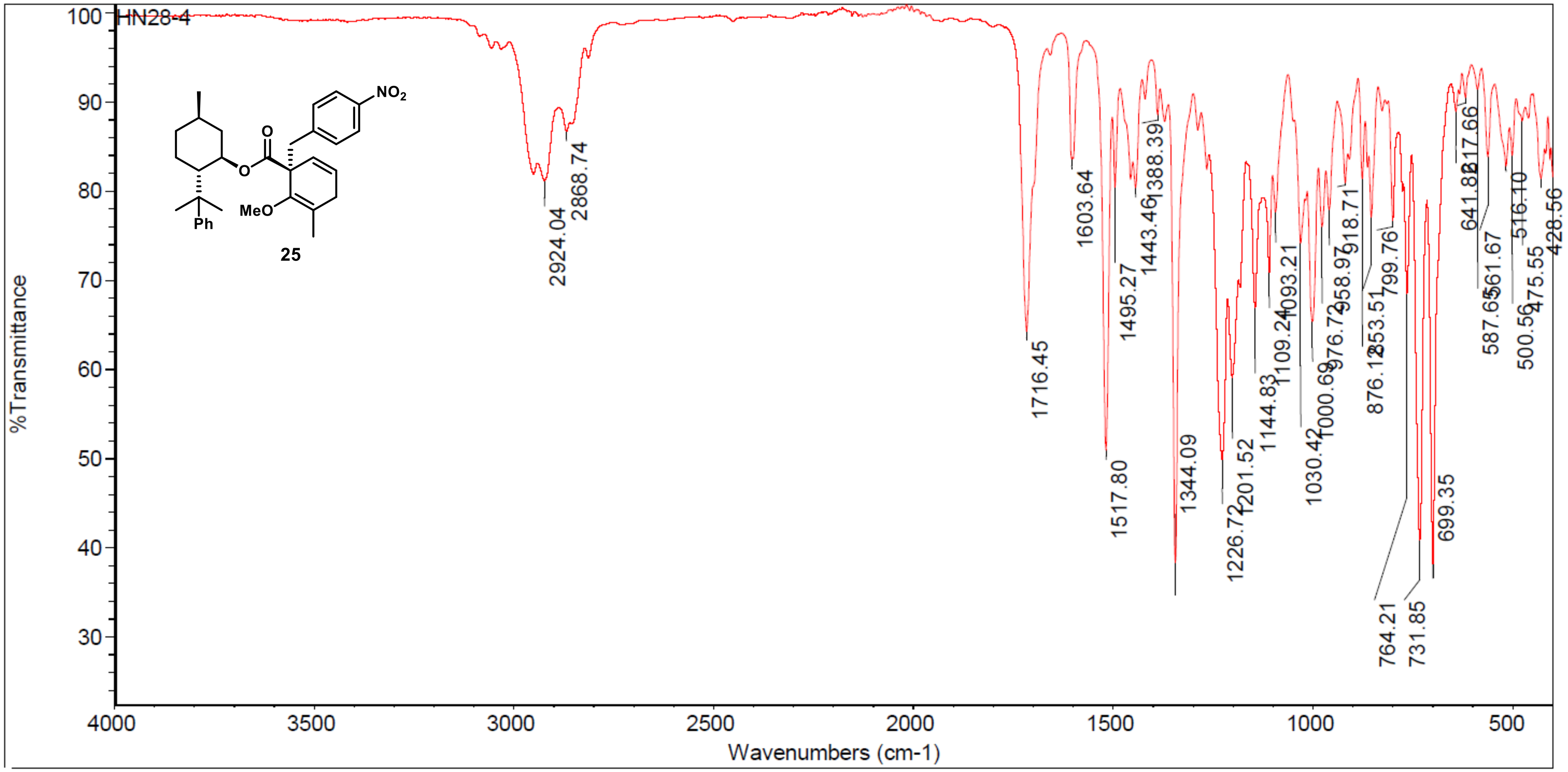

HN28-4
NO2
O
O
MeO
Ph
25
%Transmittance
Wavenumbers (cm-1)
100
90
80
70
60
50
40
30
4000
3500
3000
2500
2000
1500
1000
500
2924.04
2868.74
1716.45
1603.64
1517.80
1495.27
1443.46
1388.39
1344.09
1226.72
1201.52
1144.83
1109.24
1093.21
1030.42
1000.69
976.74
958.97
918.71
876.12
853.51
799.76
764.21
731.85
699.35
641.82
617.66
587.65
561.67
516.10
500.56
475.55
428.56

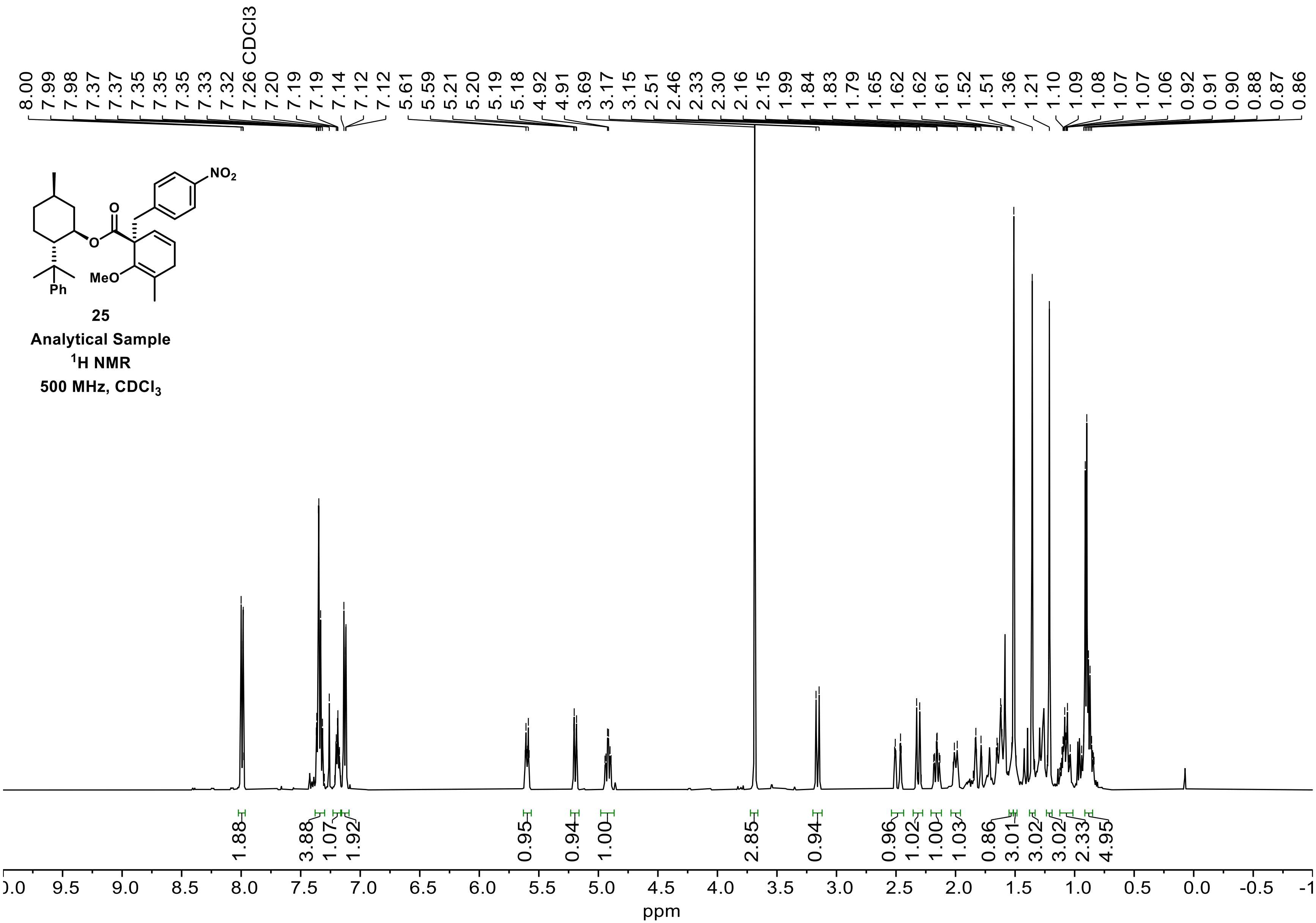

CDCl3
8.00 7.99 7.98 7.37 7.37 7.35 7.35 7.35 7.33 7.32 7.26 7.20 7.19 7.19 7.14 7.12 7.12 5.61 5.59 5.21 5.20 5.19 5.18 4.92 4.91 3.69 3.17 3.15 2.51 2.46 2.33 2.30 2.16 2.15 1.99 1.84 1.83 1.79 1.65 1.62 1.62 1.61 1.52 1.51 1.36 1.21 1.10 1.09 1.08 1.07 1.07 1.06 0.92 0.91 0.90 0.88 0.87 0.86
NO2
O
MeO
Ph
25
Analytical Sample
1H NMR
500 MHz, CDCl3
1.88 3.88 1.07 1.92 0.95 0.94 1.00 2.85 0.94 0.96 1.02 1.00 1.03 0.86 3.01 3.02 3.02 2.33 4.95
ppm

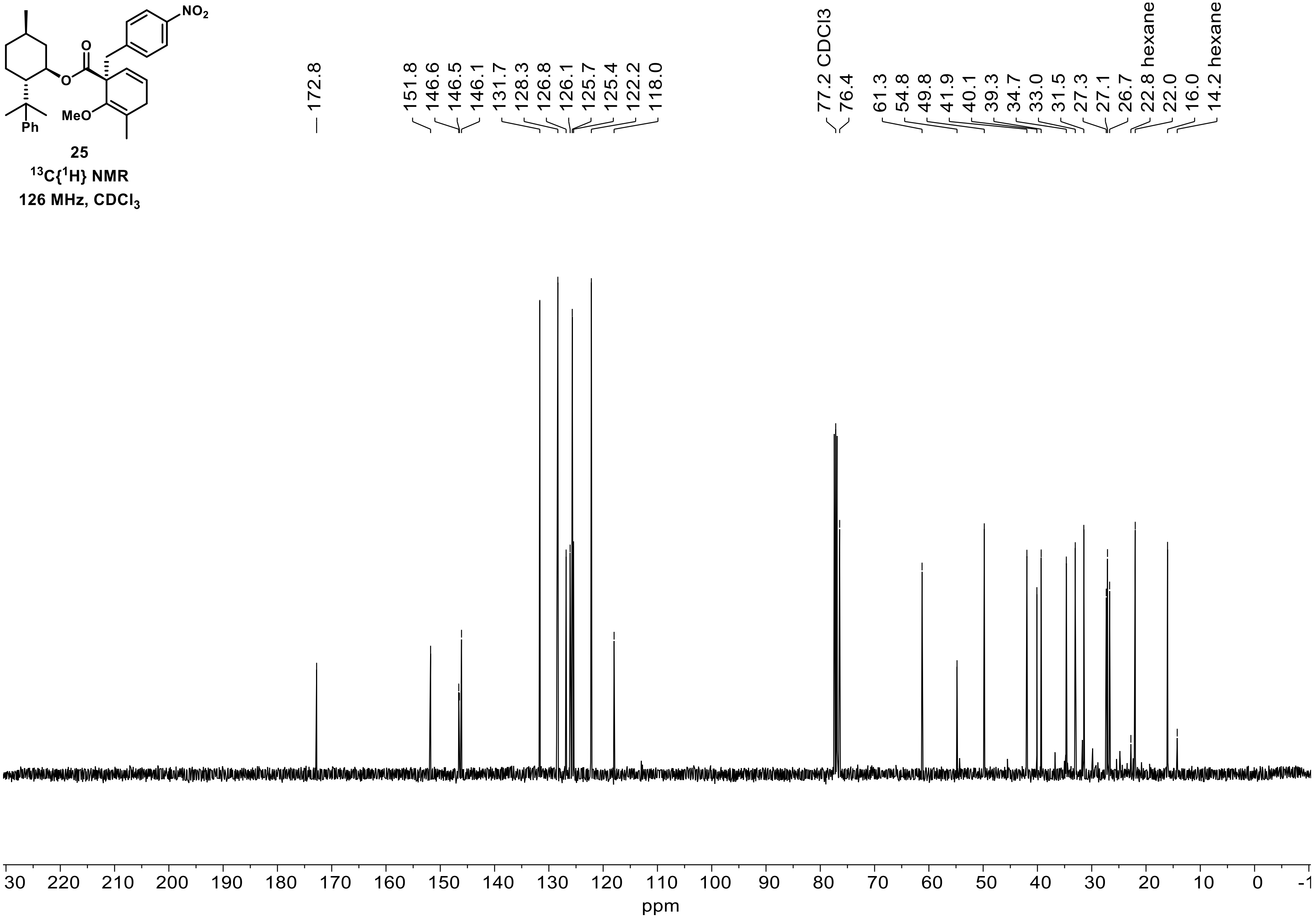
25
$^{13}C\{^1H\}$ NMR
126 MHz, $CDCl_3$
172.8
151.8
146.6
146.5
146.1
131.7
128.3
126.8
126.1
125.7
125.4
122.2
118.0
77.2 CDCl3
76.4
61.3
54.8
49.8
41.9
40.1
39.3
34.7
33.0
31.5
27.3
27.1
26.7
22.8 hexane
22.0
16.0
14.2 hexane
ppm

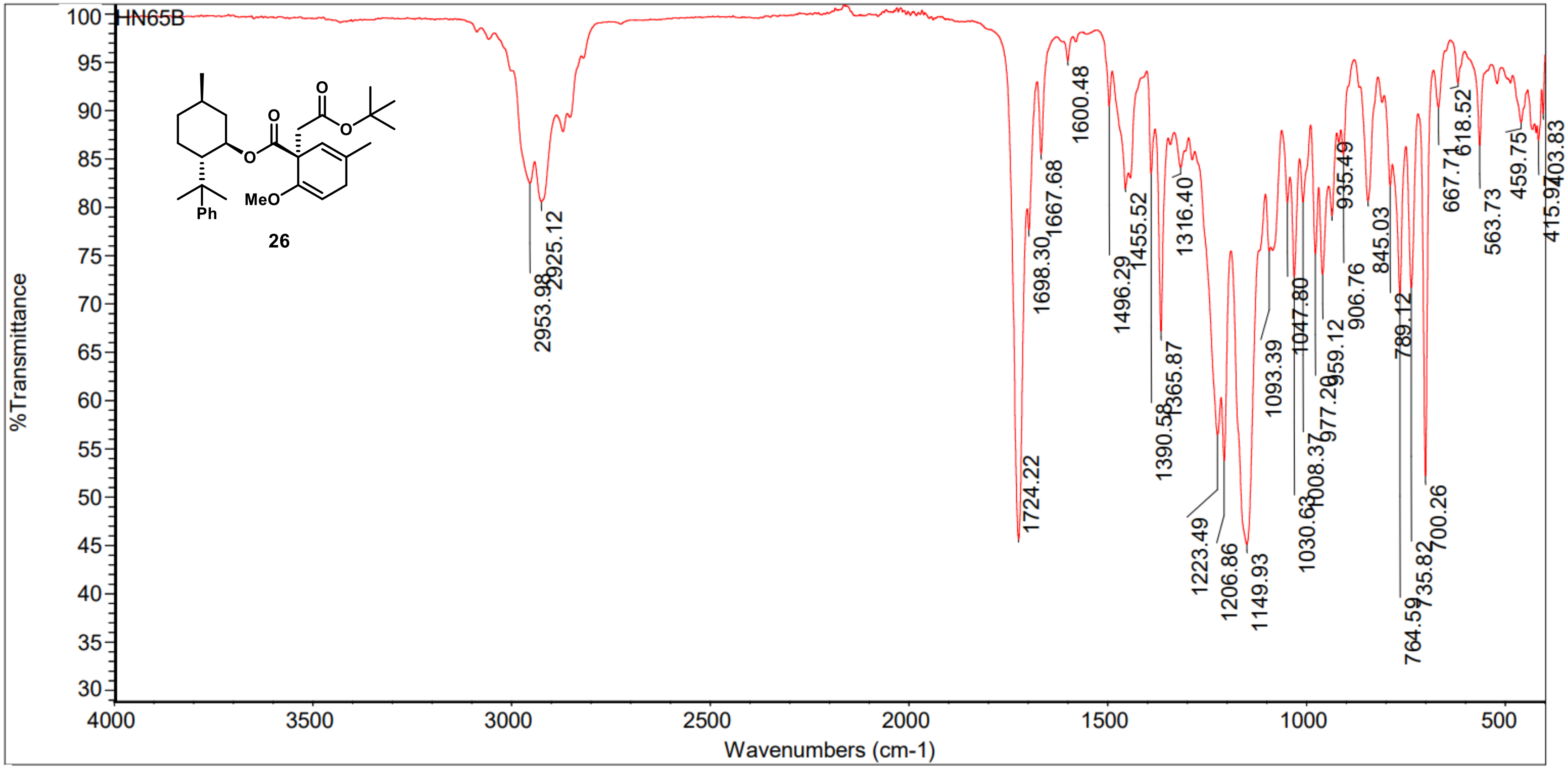
HN65B
%Transmittance
Wavenumbers (cm-1)
MeO
Ph
26
2953.98
2925.12
1724.22
1698.30
1667.68
1600.48
1496.29
1455.52
1390.58
1365.87
1316.40
1223.49
1206.86
1149.93
1093.39
1047.80
1030.63
1008.37
977.29
959.12
935.49
906.76
845.03
789.12
764.59
735.82
700.26
667.71
618.52
563.73
459.75
415.97
403.83

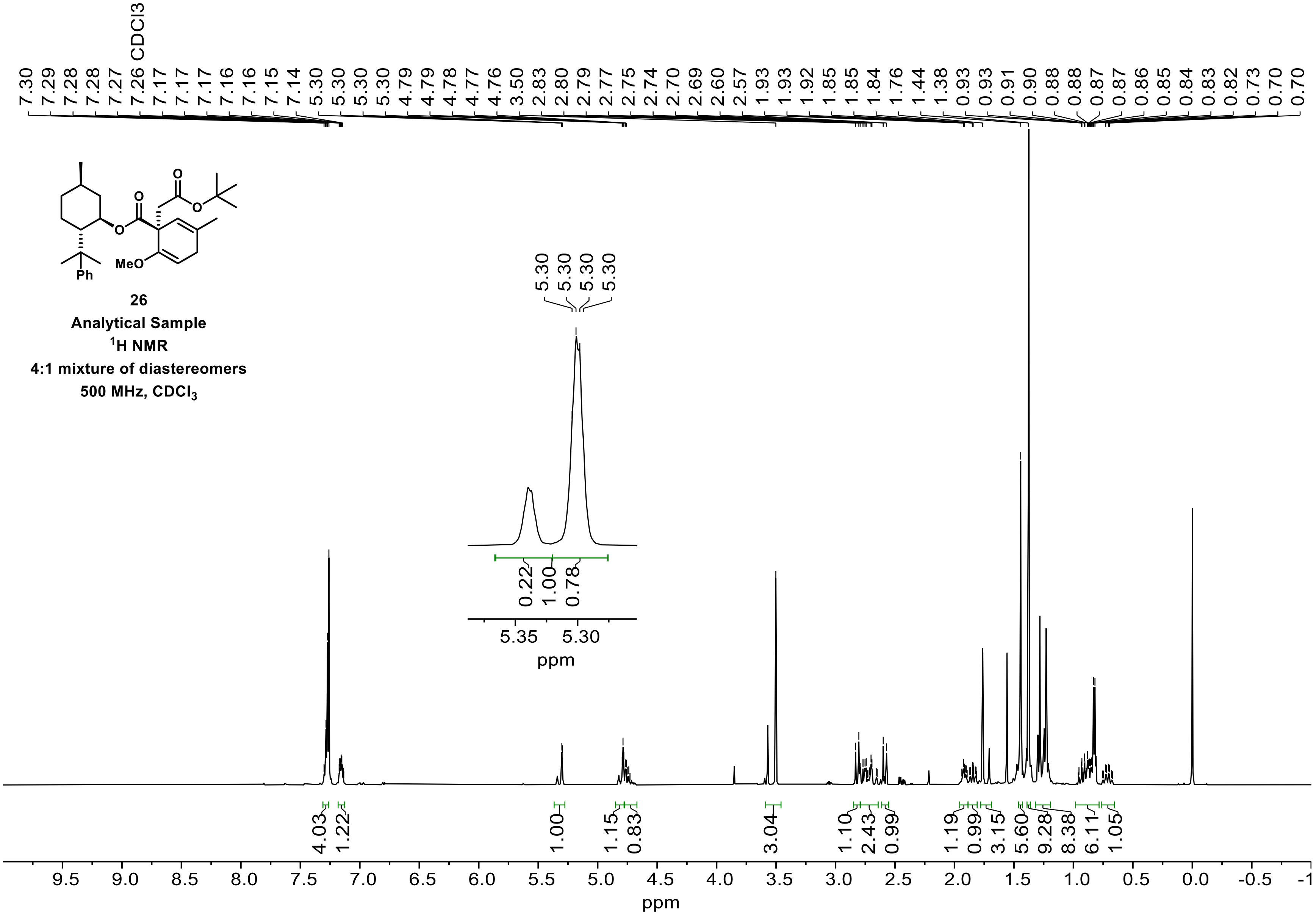

MeO
Ph
26
Analytical Sample
1H NMR
4:1 mixture of diastereomers
500 MHz, CDCl3
CDCl3
ppm

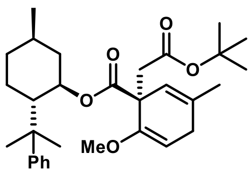

26

$^{13}C\{^{1}H\}$ NMR

4:1 mixture of diastereomers

151 MHz, $CDCl_3$

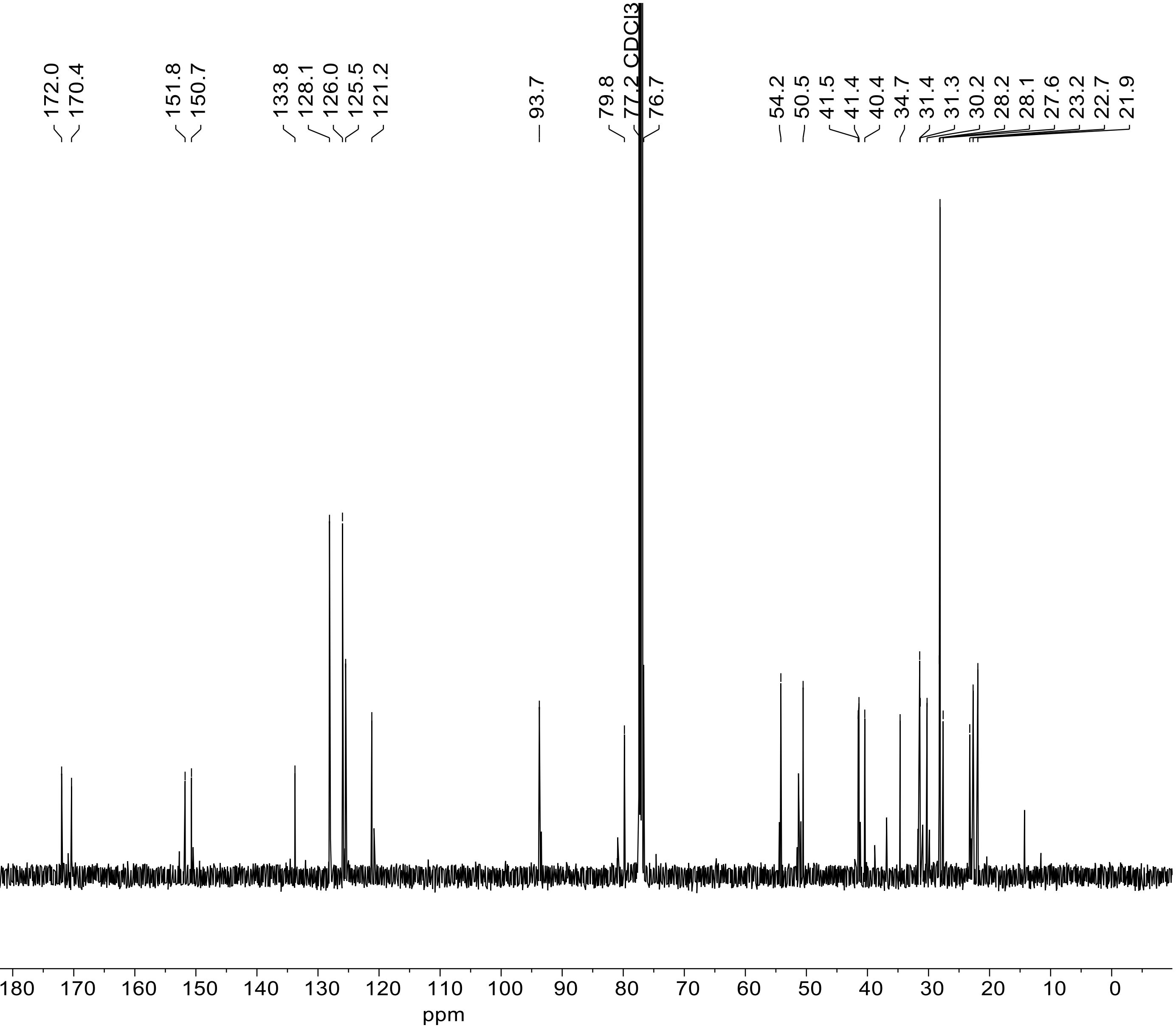

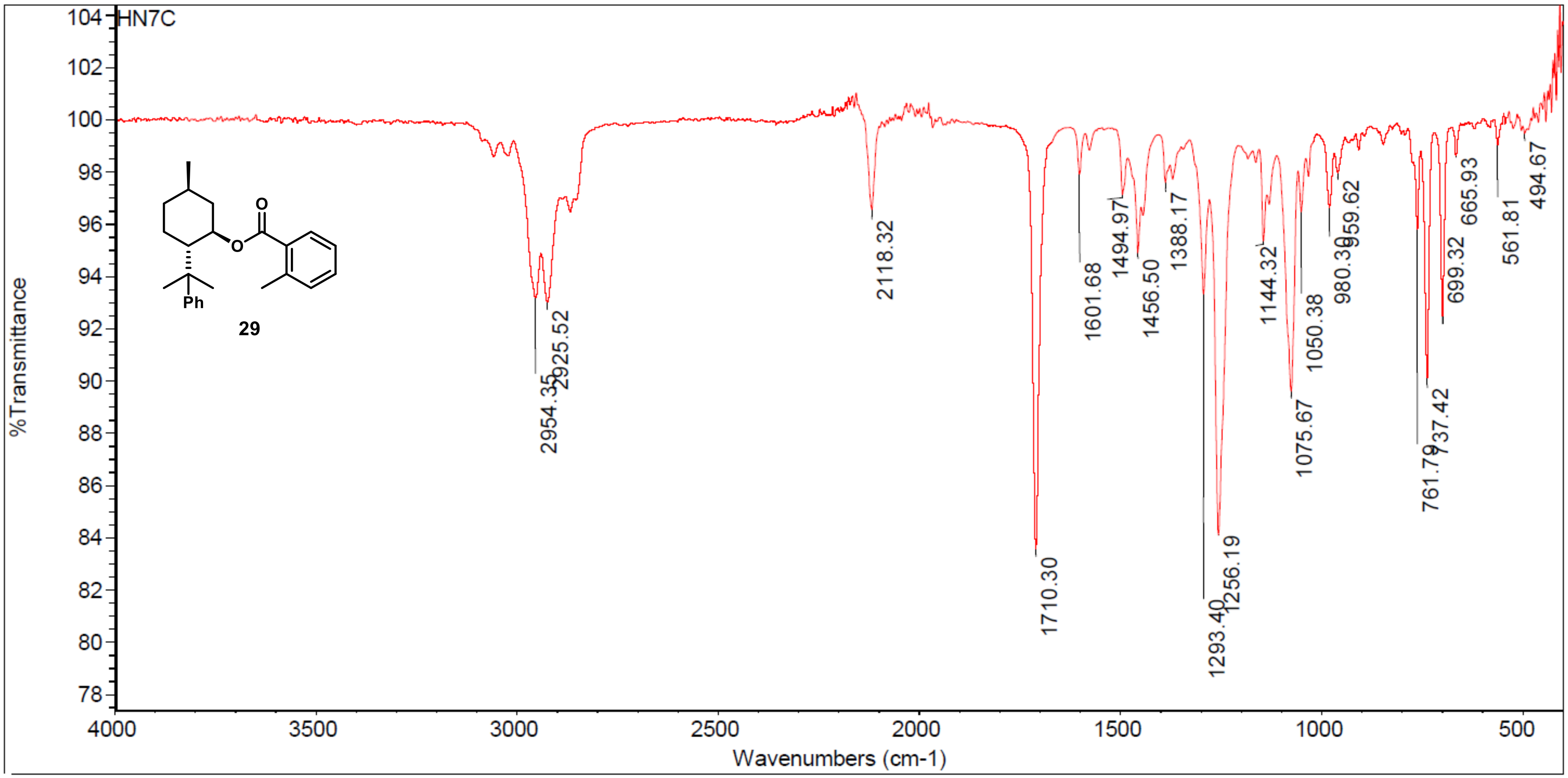
HN7C
%Transmittance
Wavenumbers (cm-1)
104
102
100
98
96
94
92
90
88
86
84
82
80
78
4000
3500
3000
2500
2000
1500
1000
500
2954.35
2925.52
2118.32
1710.30
1601.68
1494.97
1456.50
1388.17
1293.40
1256.19
1144.32
1075.67
1050.38
980.39
959.62
761.79
737.42
699.32
665.93
561.81
494.67
O
O
Ph
29

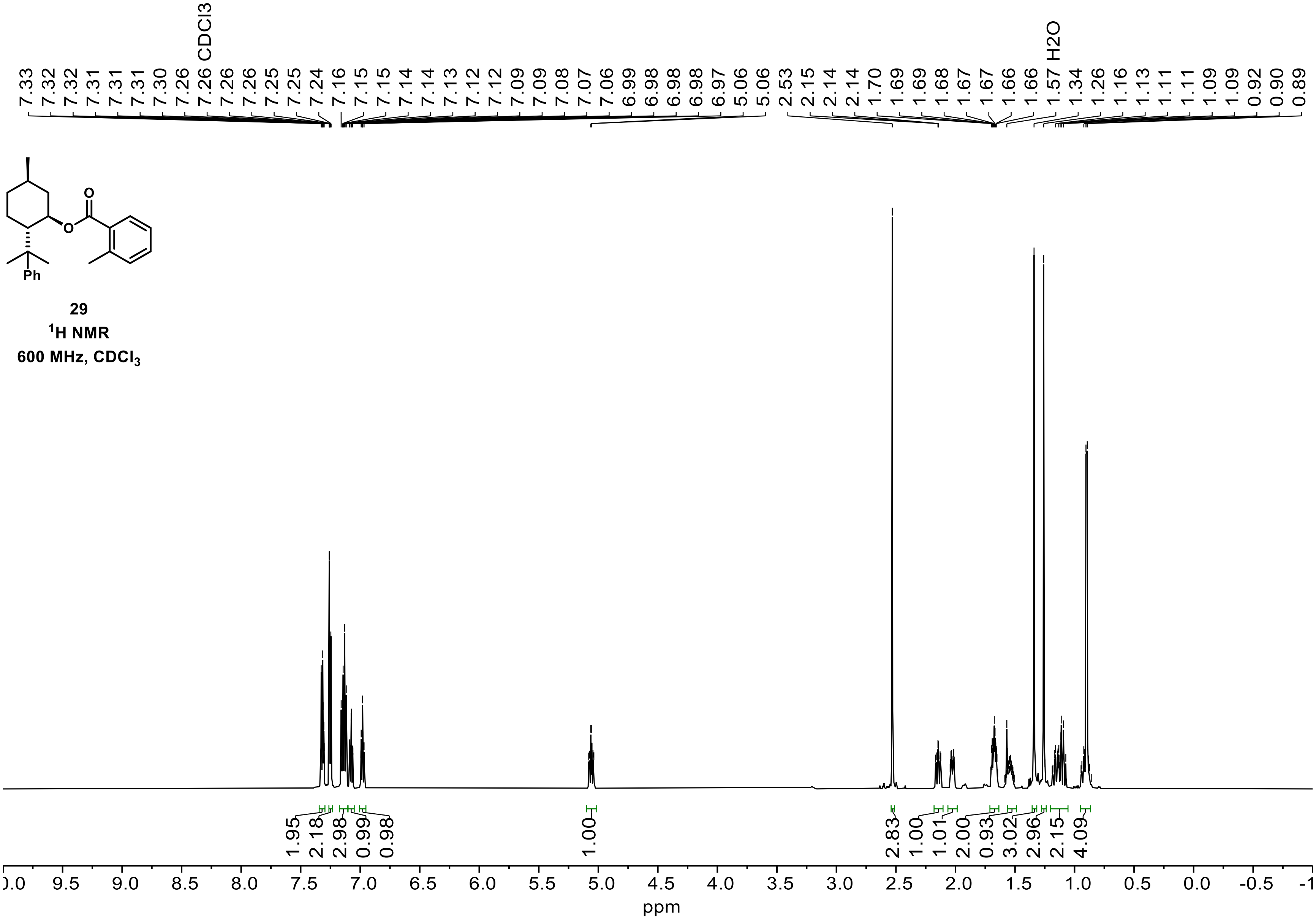

29
¹H NMR
600 MHz, CDCl₃
CDCl3
H2O
ppm

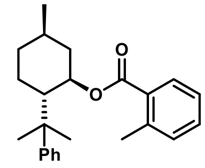

29

$^{13}C\{^{1}H\}$ NMR

151 MHz, $CDCl_3$

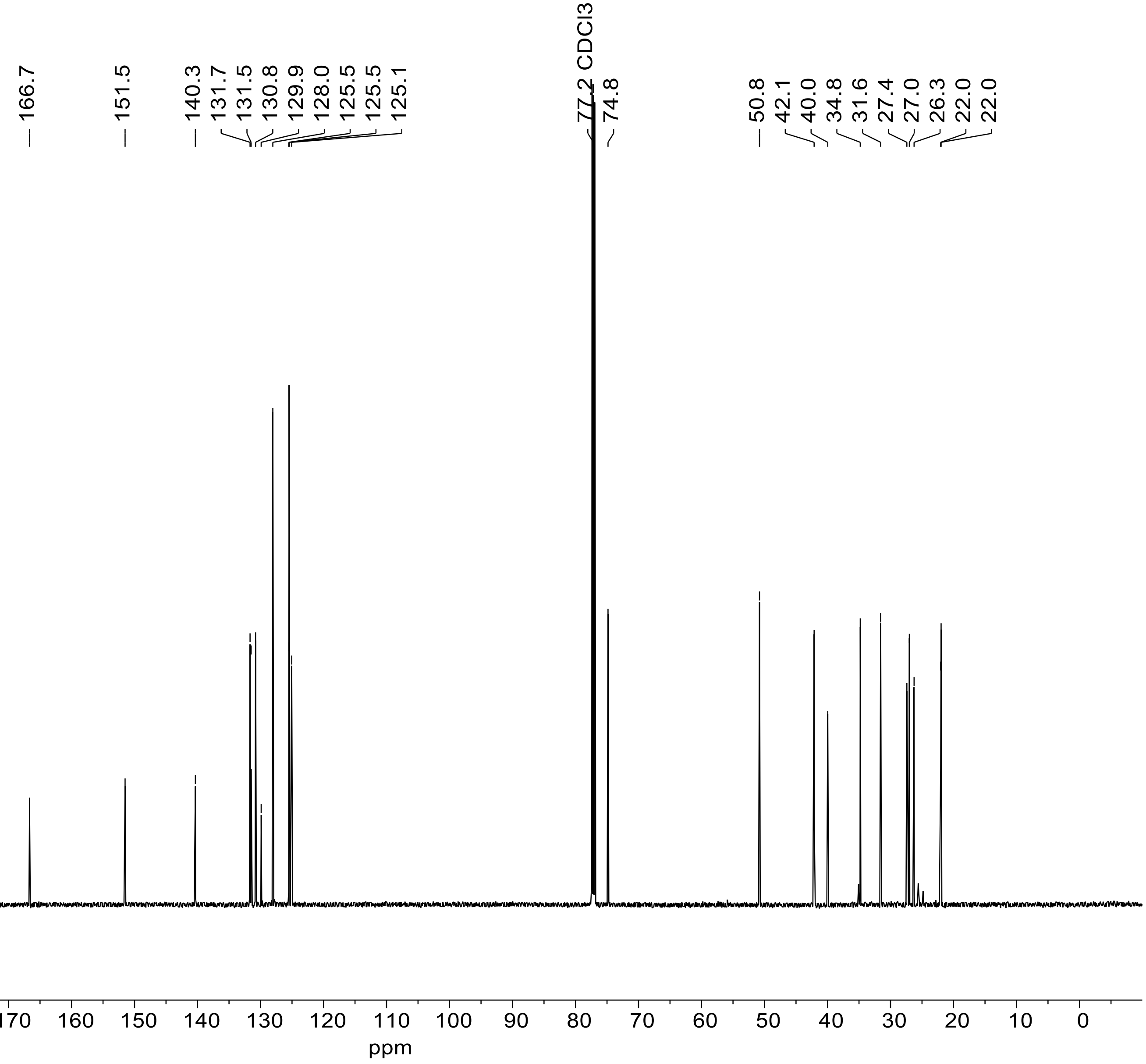

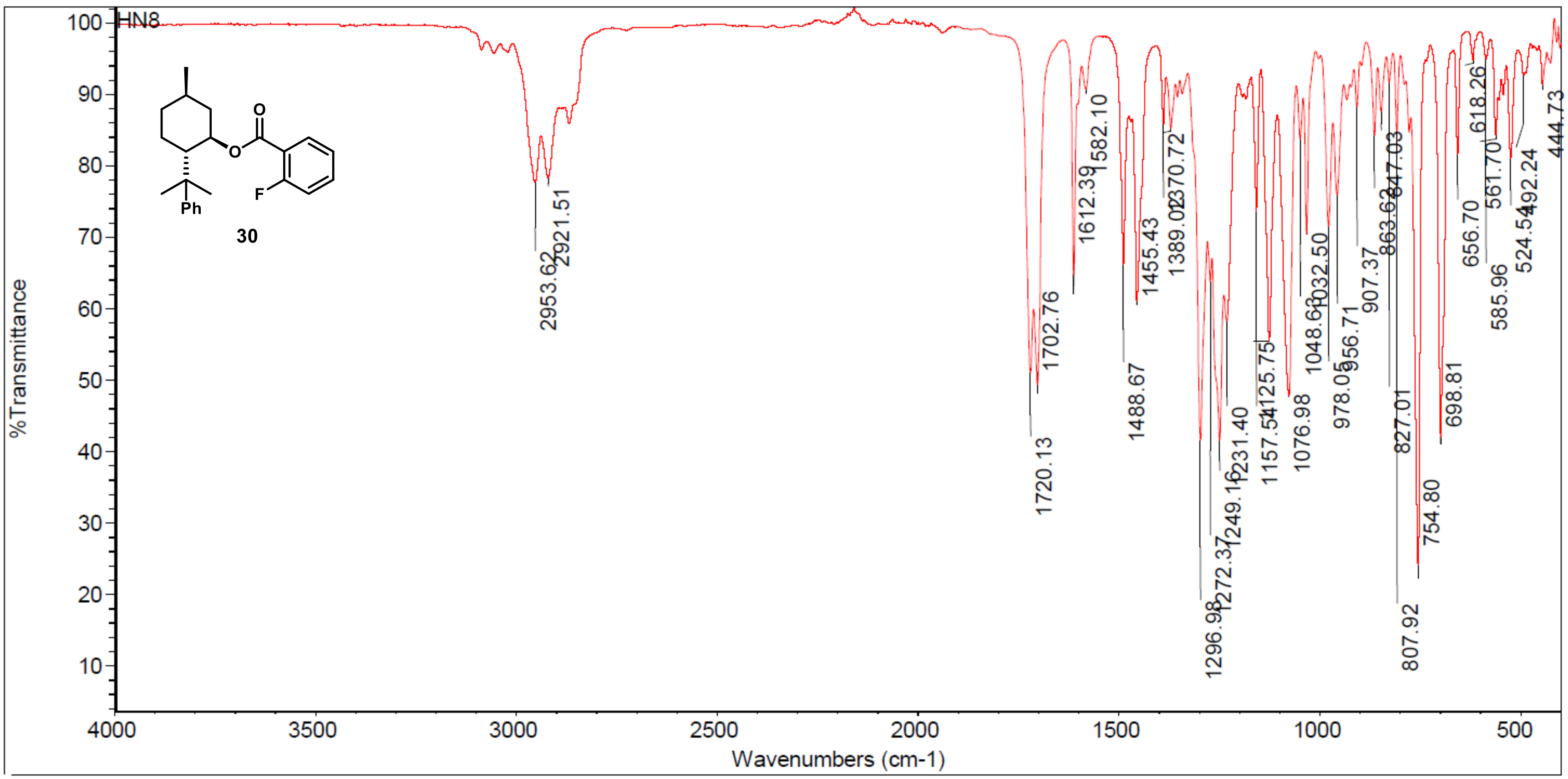
HN8
%Transmittance
Wavenumbers (cm-1)
30
2953.62
2921.51
1720.13
1702.76
1612.39
1582.10
1488.67
1455.43
1389.01
1370.72
1296.98
1272.37
1249.16
1231.40
1157.54
1125.75
1076.98
1048.63
1032.50
978.05
956.71
907.37
863.62
847.03
827.01
807.92
754.80
698.81
656.70
618.26
585.96
561.70
524.54
492.24
444.73

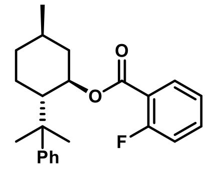

30
^{1}H NMR
600 MHz, $CDCl_3$

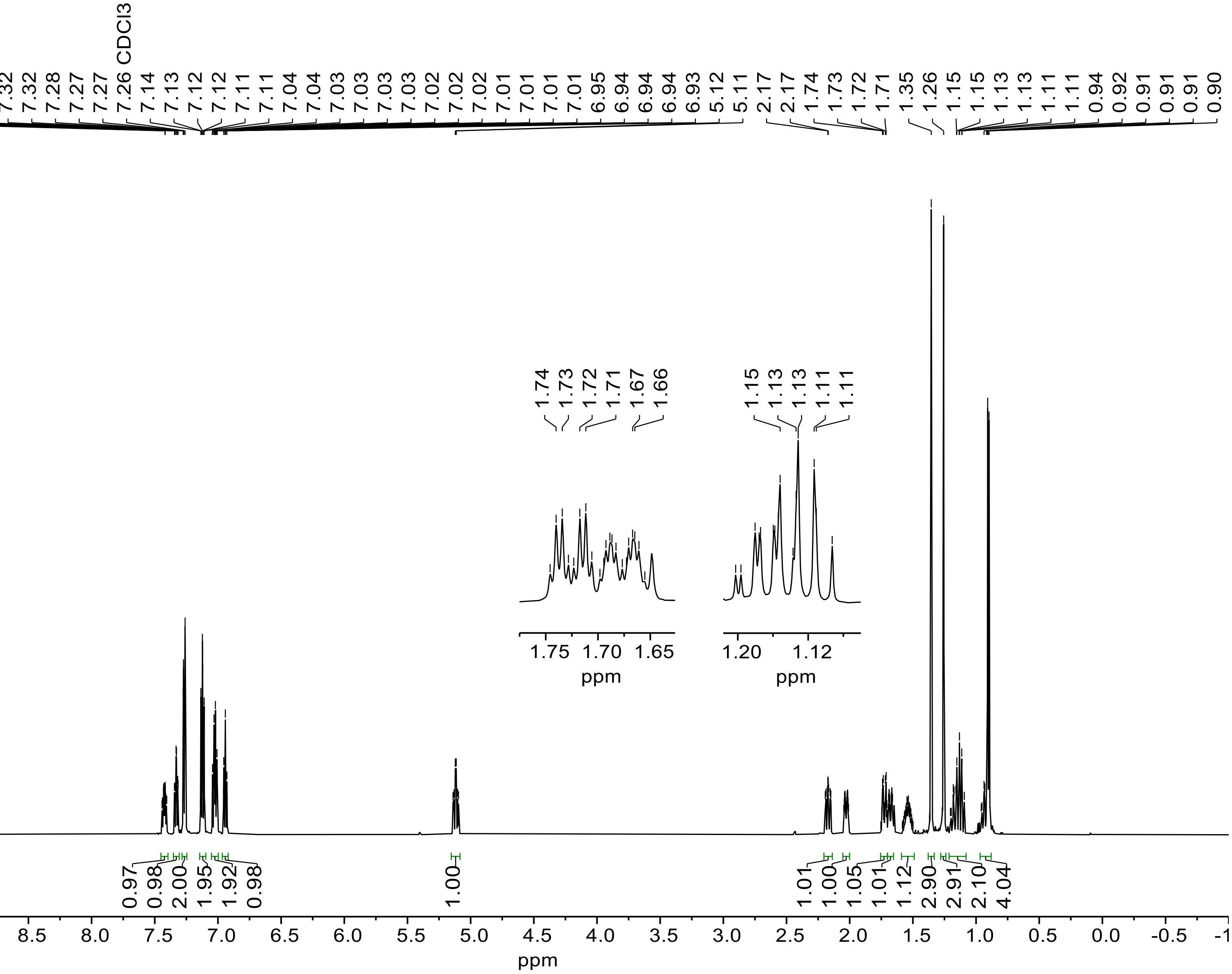

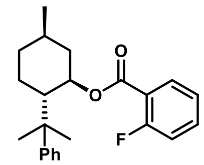

30

$^{13}C\{^{1}H\}$ NMR

151 MHz, $CDCl_3$

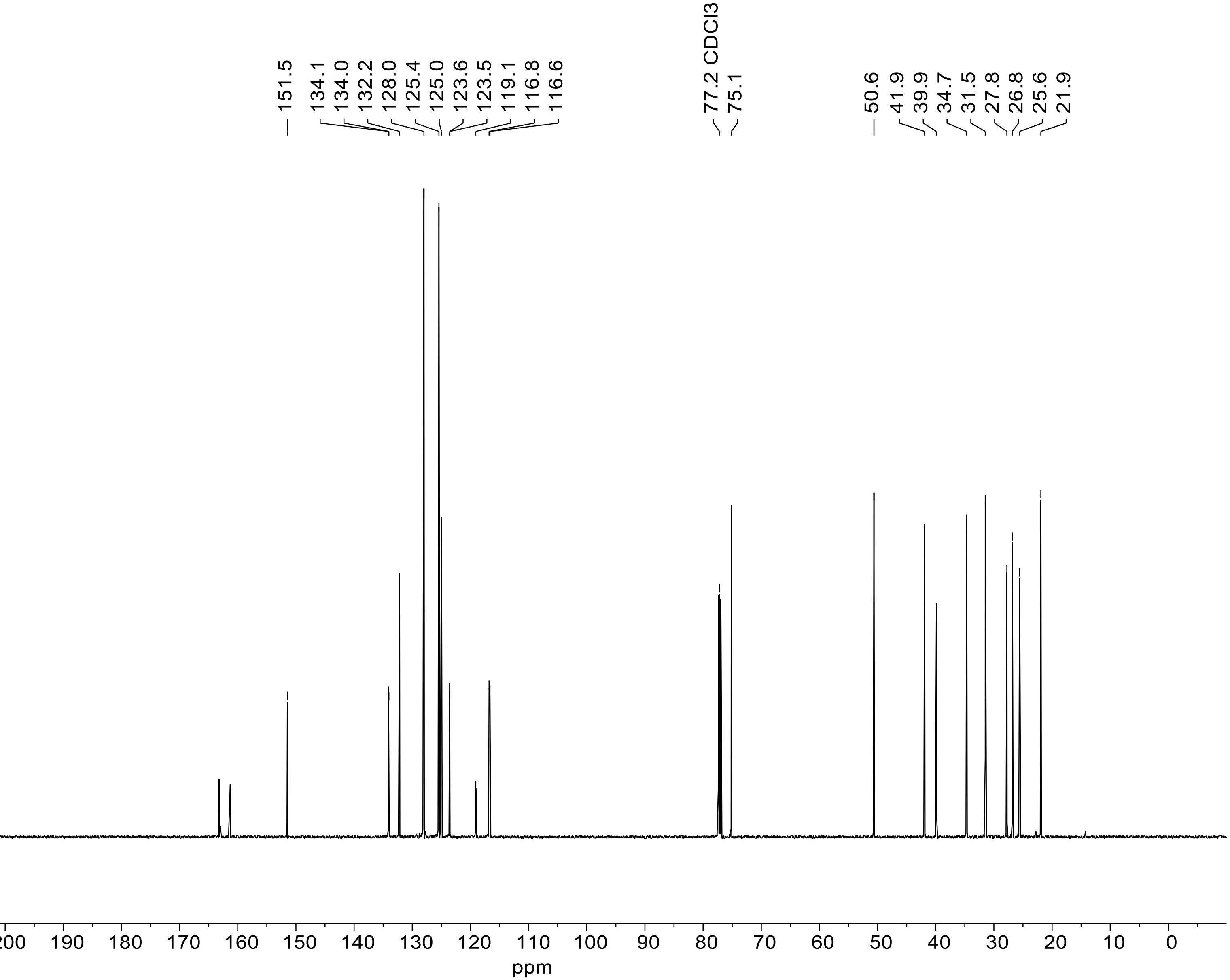

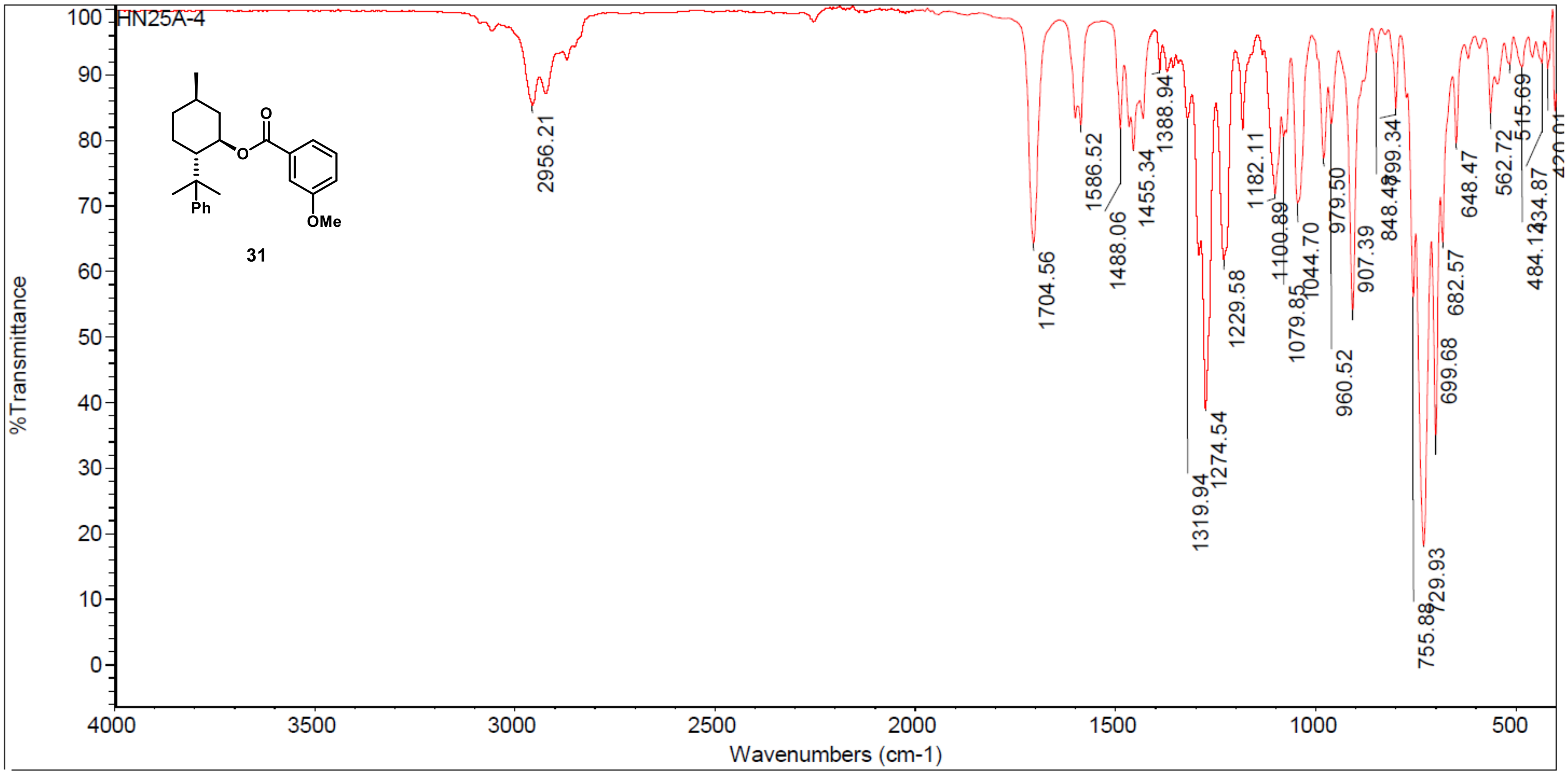
HN25A-4
%Transmittance
Wavenumbers (cm-1)
2956.21
1704.56
1586.52
1488.06
1455.34
1388.94
1319.94
1274.54
1229.58
1182.11
1100.89
1079.85
1044.70
979.50
960.52
907.39
848.46
799.34
755.88
729.93
699.68
682.57
648.47
562.72
515.69
484.12
434.87
O
O
Ph
OMe
31

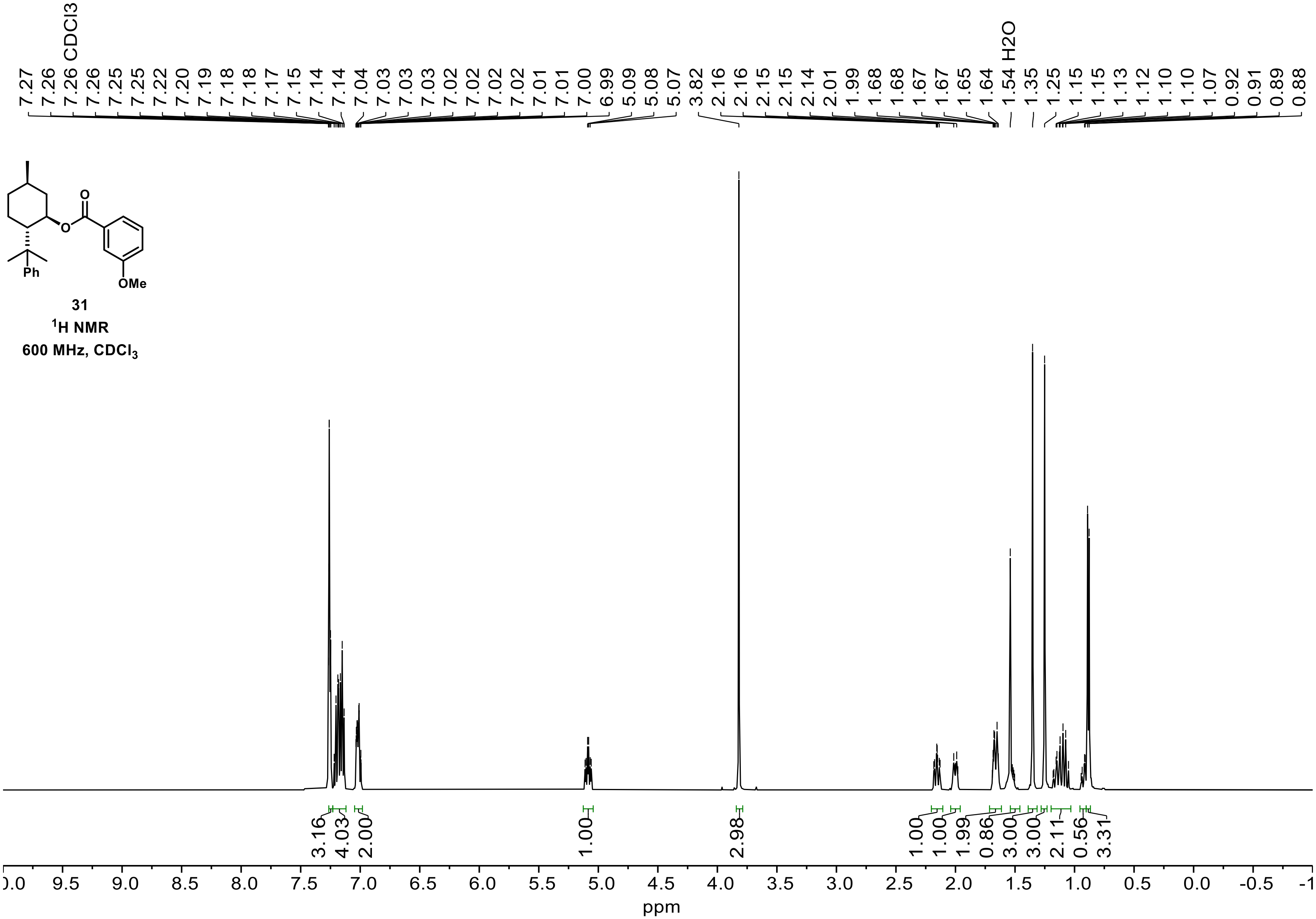

31

^{1}H NMR

600 MHz, $CDCl_3$

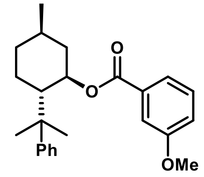

31

$^{13}C\{^1H\}$ NMR

151 MHz, $CDCl_3$

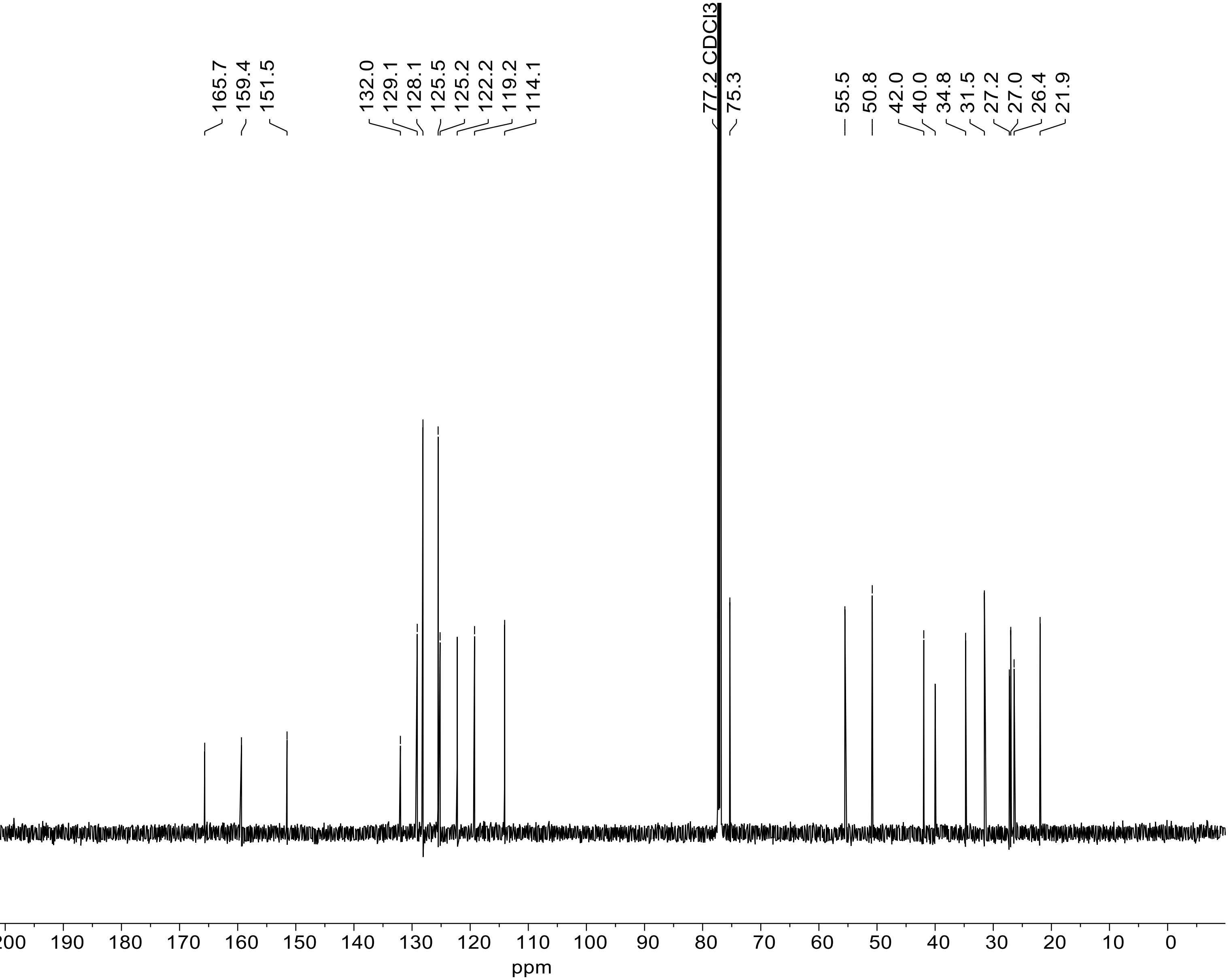

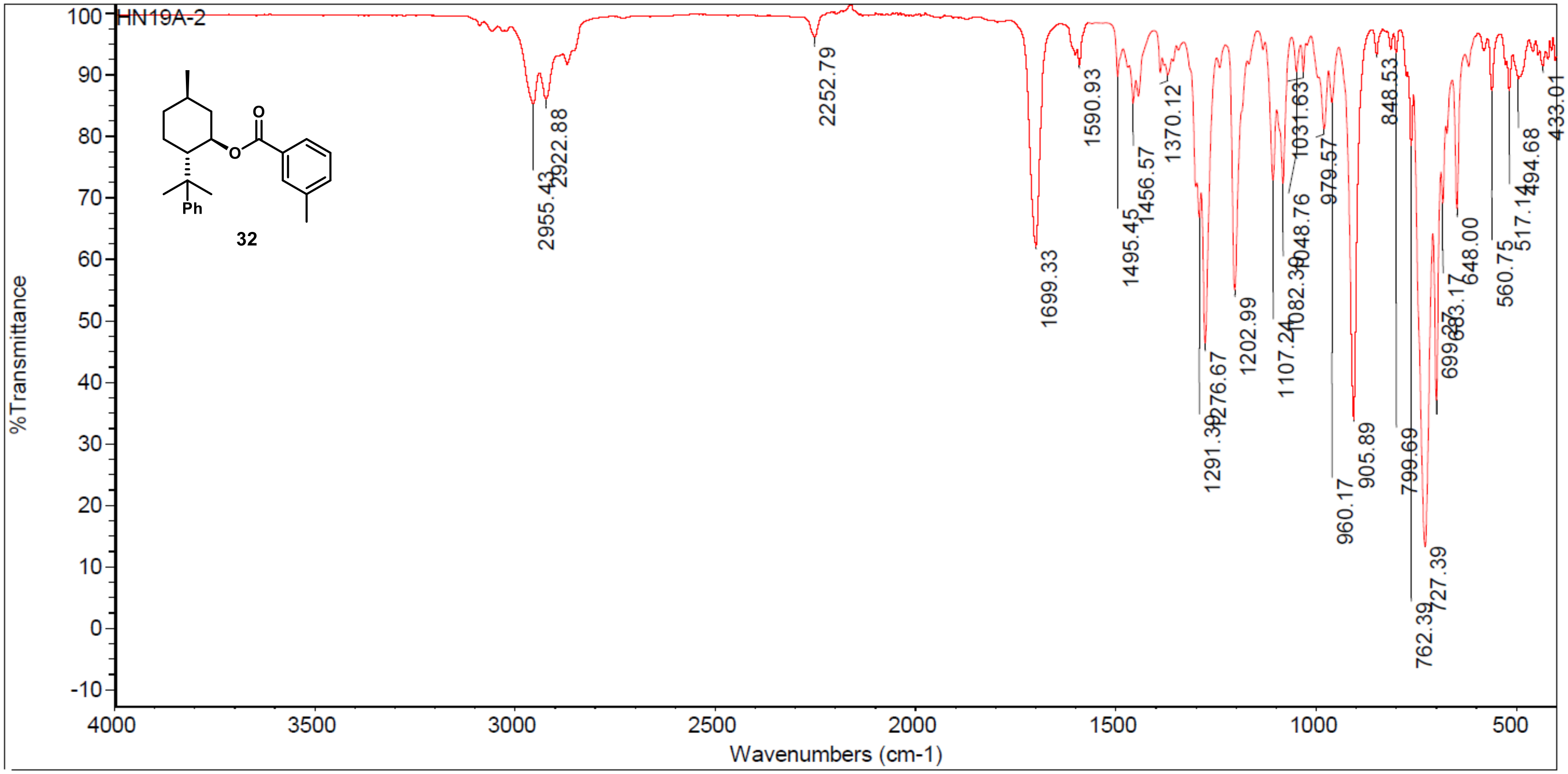
HN19A-2
%Transmittance
Wavenumbers (cm-1)
32
Ph
O
2955.43
2922.88
2252.79
1699.33
1590.93
1495.45
1456.57
1370.12
1291.39
1276.67
1202.99
1107.24
1082.39
1048.76
1031.63
979.57
960.17
905.89
848.53
799.69
762.39
727.39
699.37
683.17
648.00
560.75
517.14
494.68
433.01

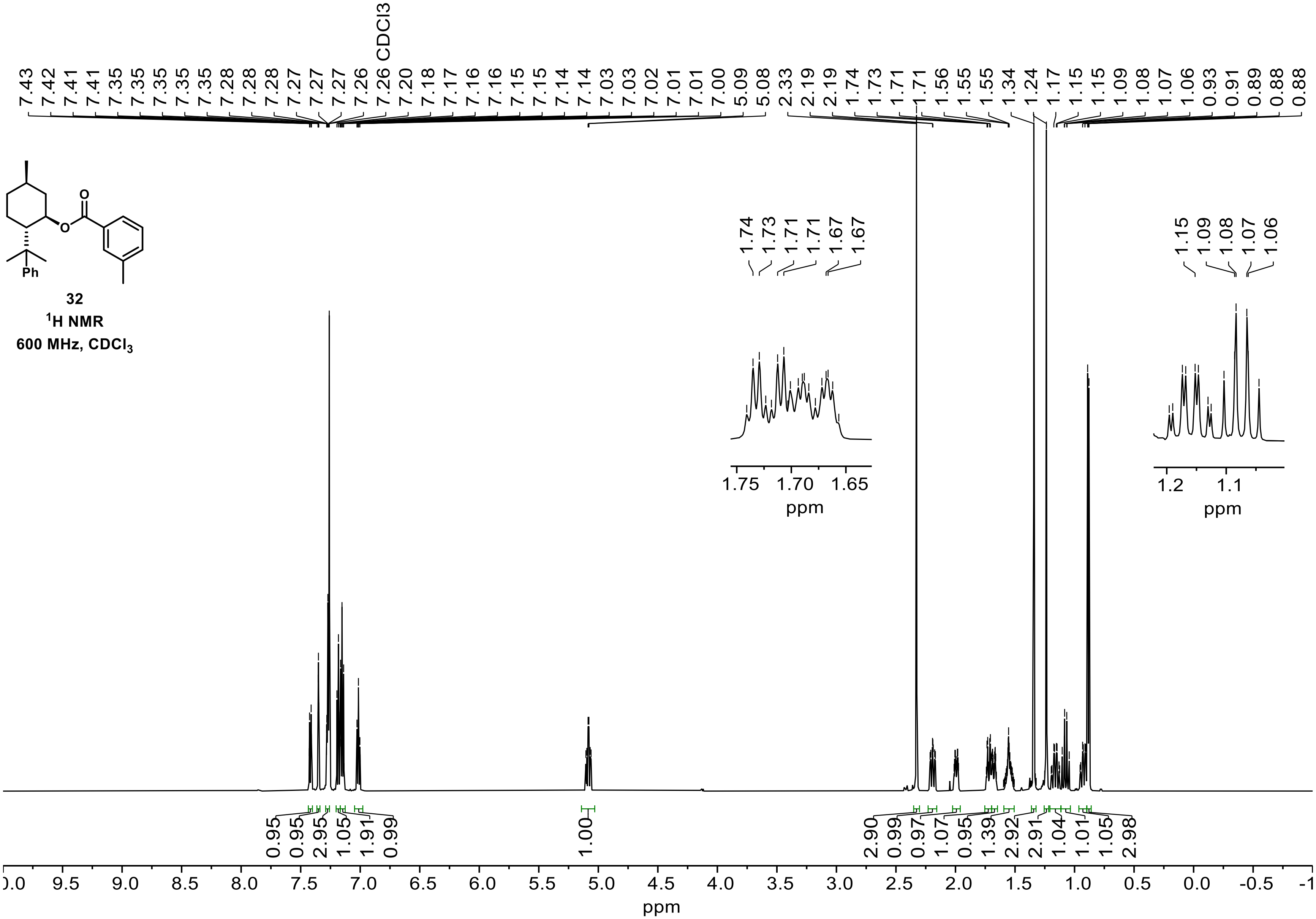
32
1H NMR
600 MHz, CDCl3
CDCl3
ppm

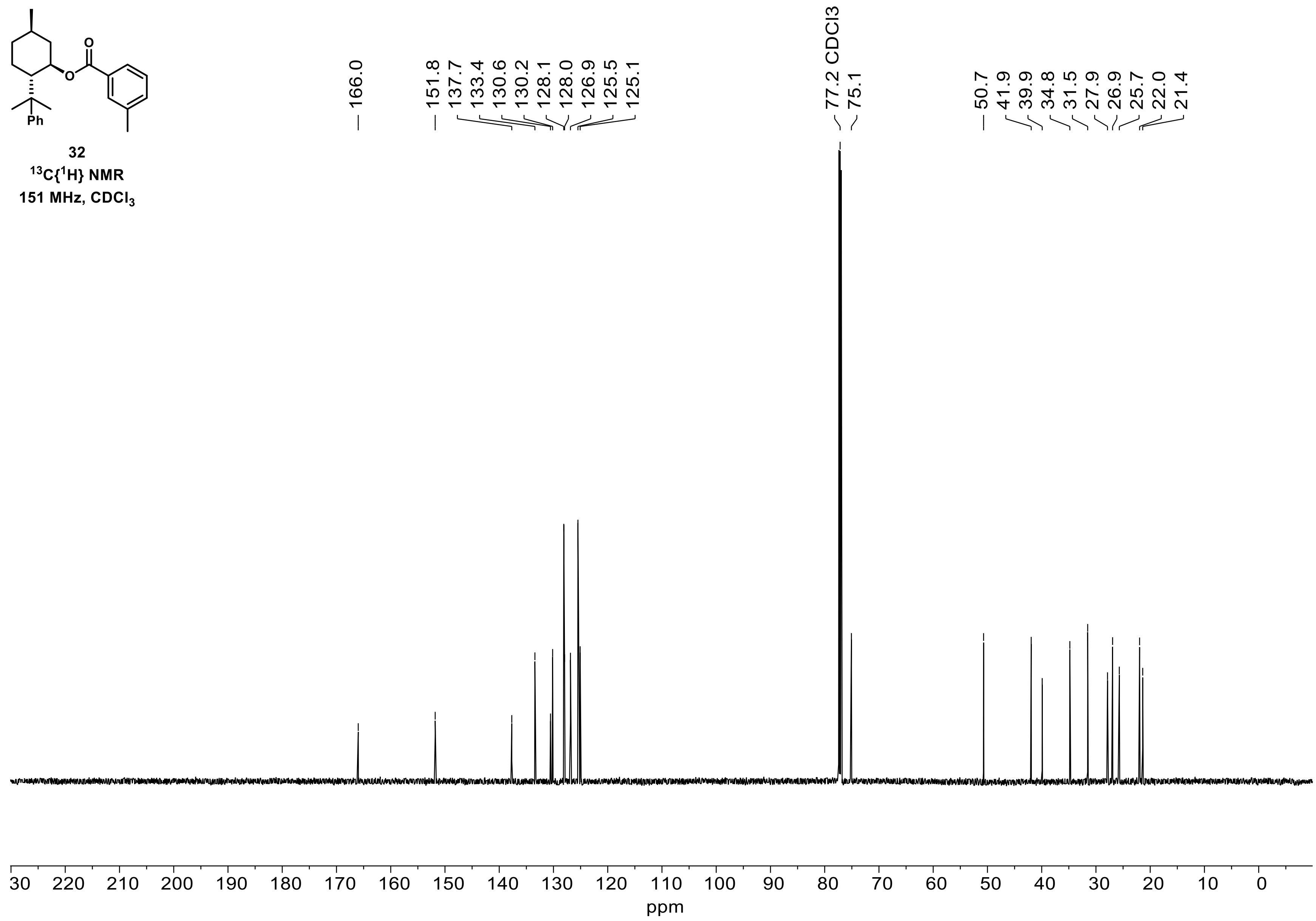

32
$^{13}C\{^{1}H\}$ NMR
151 MHz, $CDCl_3$

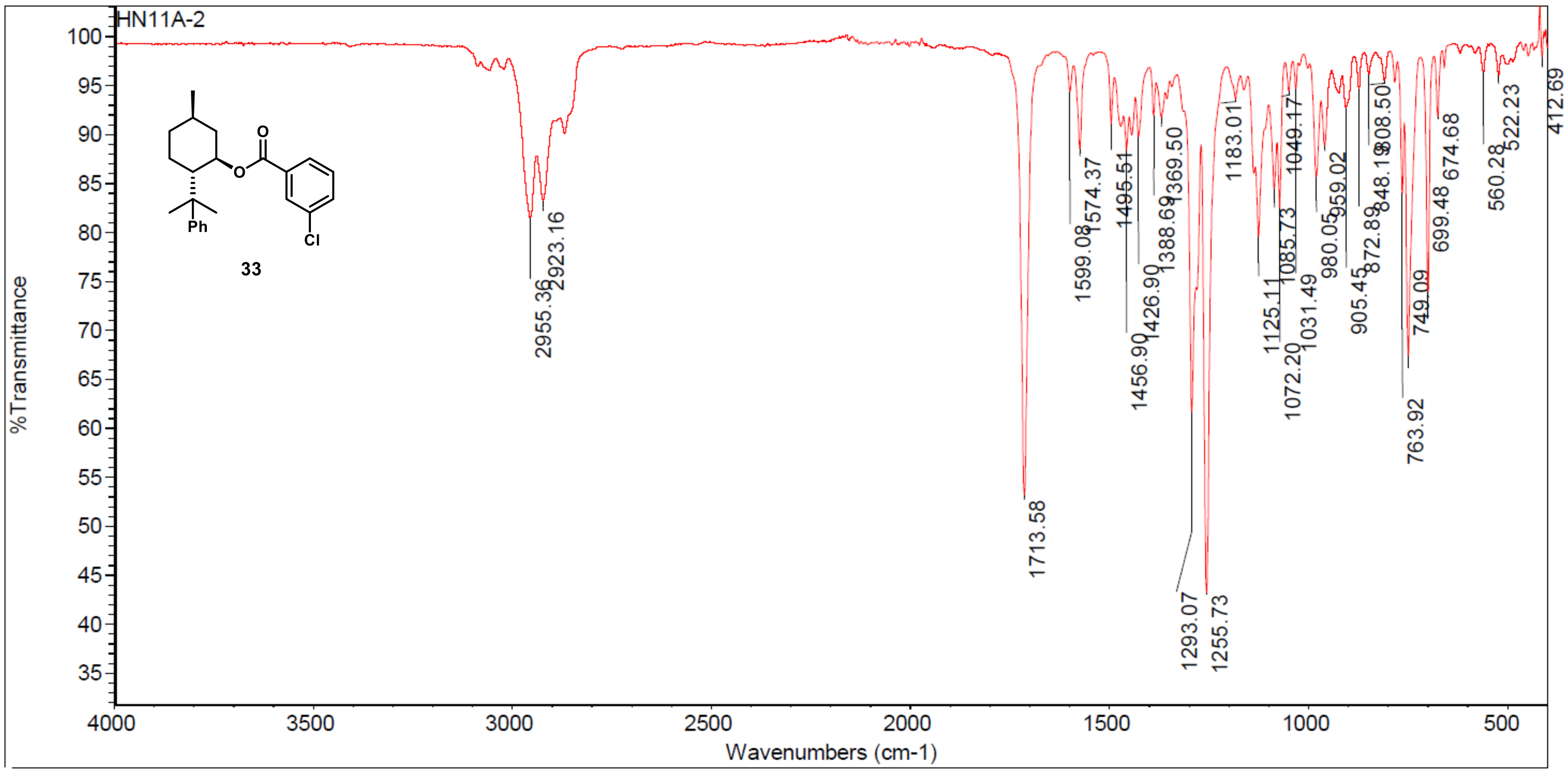

HN11A-2
%Transmittance
Wavenumbers (cm-1)
33
Ph
Cl
O
2955.36
2923.16
1713.58
1599.08
1574.37
1495.51
1456.90
1426.90
1388.69
1369.50
1293.07
1255.73
1183.01
1125.11
1085.73
1072.20
1049.17
1031.49
980.05
959.02
905.45
872.89
848.19
808.50
763.92
749.09
699.48
674.68
560.28
522.23
412.69

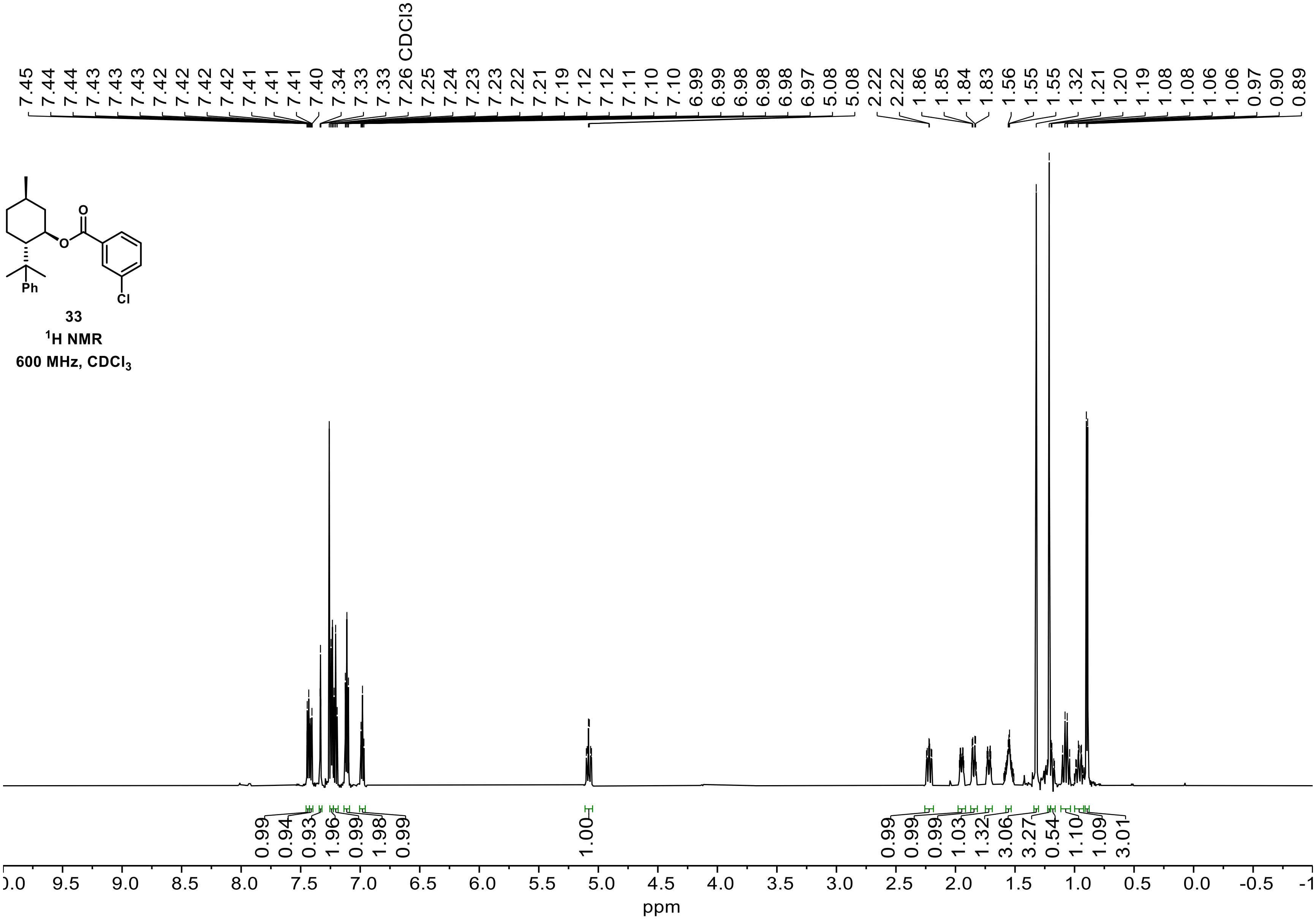

7.45 7.44 7.44 7.43 7.43 7.43 7.42 7.42 7.42 7.42 7.41 7.41 7.41 7.40 7.34 7.33 7.33 7.26 CDCl3 7.25 7.24 7.23 7.23 7.22 7.21 7.19 7.12 7.12 7.11 7.10 7.10 6.99 6.99 6.98 6.98 6.98 6.97 5.08 5.08 2.22 2.22 1.86 1.85 1.84 1.83 1.56 1.55 1.55 1.32 1.21 1.20 1.19 1.08 1.08 1.06 1.06 0.97 0.90 0.89
O
O
Ph
Cl
33
1H NMR
600 MHz, CDCl3
0.99 0.94 0.93 1.96 0.99 1.98 0.99
1.00
0.99 0.99 0.99 1.03 1.32 3.06 3.27 0.54 1.10 1.09 3.01
10.0 9.5 9.0 8.5 8.0 7.5 7.0 6.5 6.0 5.5 5.0 4.5 4.0 3.5 3.0 2.5 2.0 1.5 1.0 0.5 0.0 -0.5 -1
ppm

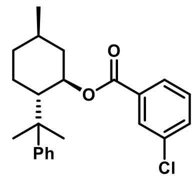

33

$^{13}C\{^{1}H\}$ NMR

151 MHz, $CDCl_3$

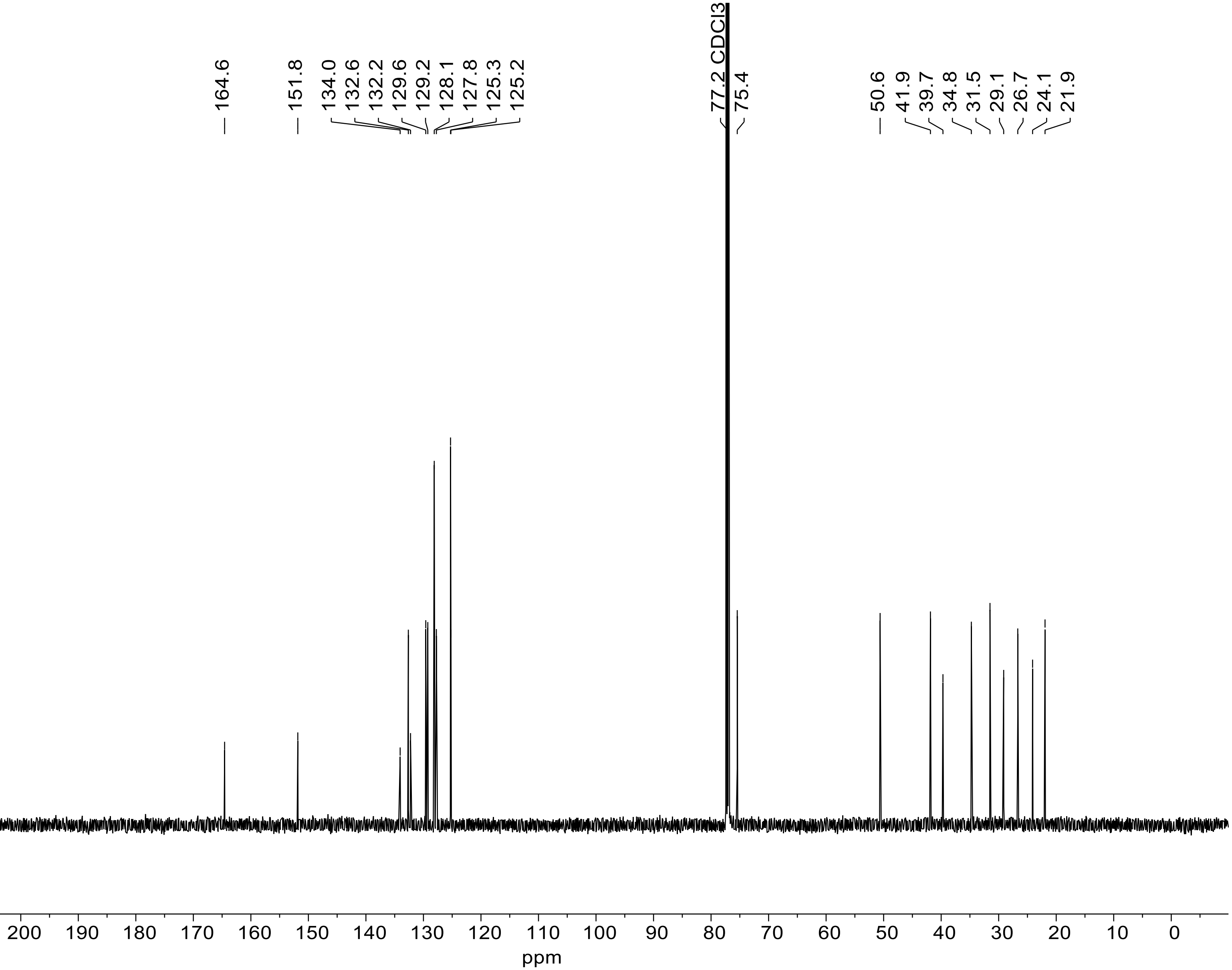

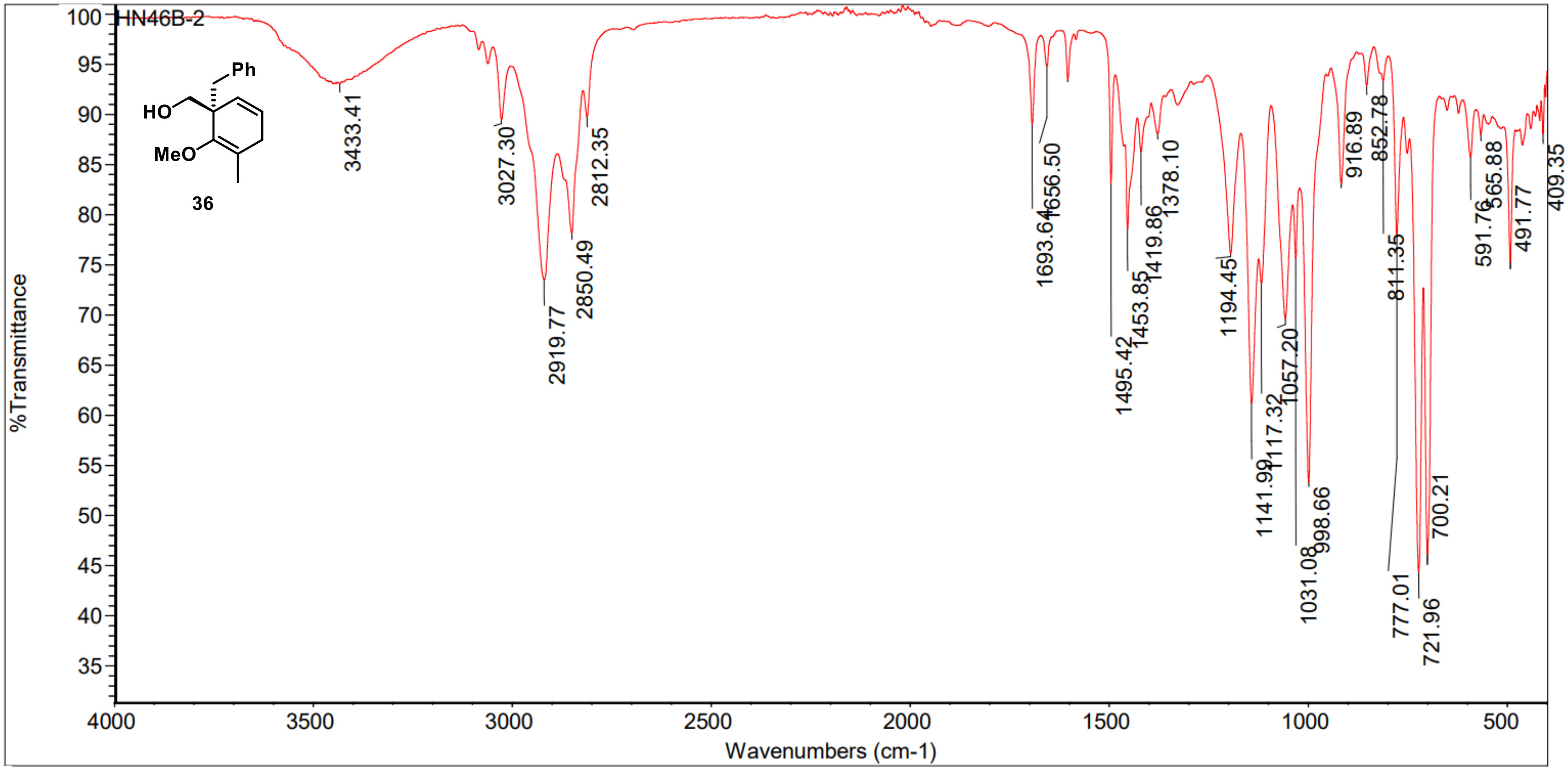
HN46B-2
HO
MeO
Ph
36
%Transmittance
Wavenumbers (cm-1)
3433.41
3027.30
2919.77
2850.49
2812.35
1693.64
1656.50
1495.42
1453.85
1419.86
1378.10
1194.45
1141.99
1117.32
1057.20
1031.08
998.66
916.89
852.78
811.35
777.01
721.96
700.21
591.76
565.88
491.77
409.35

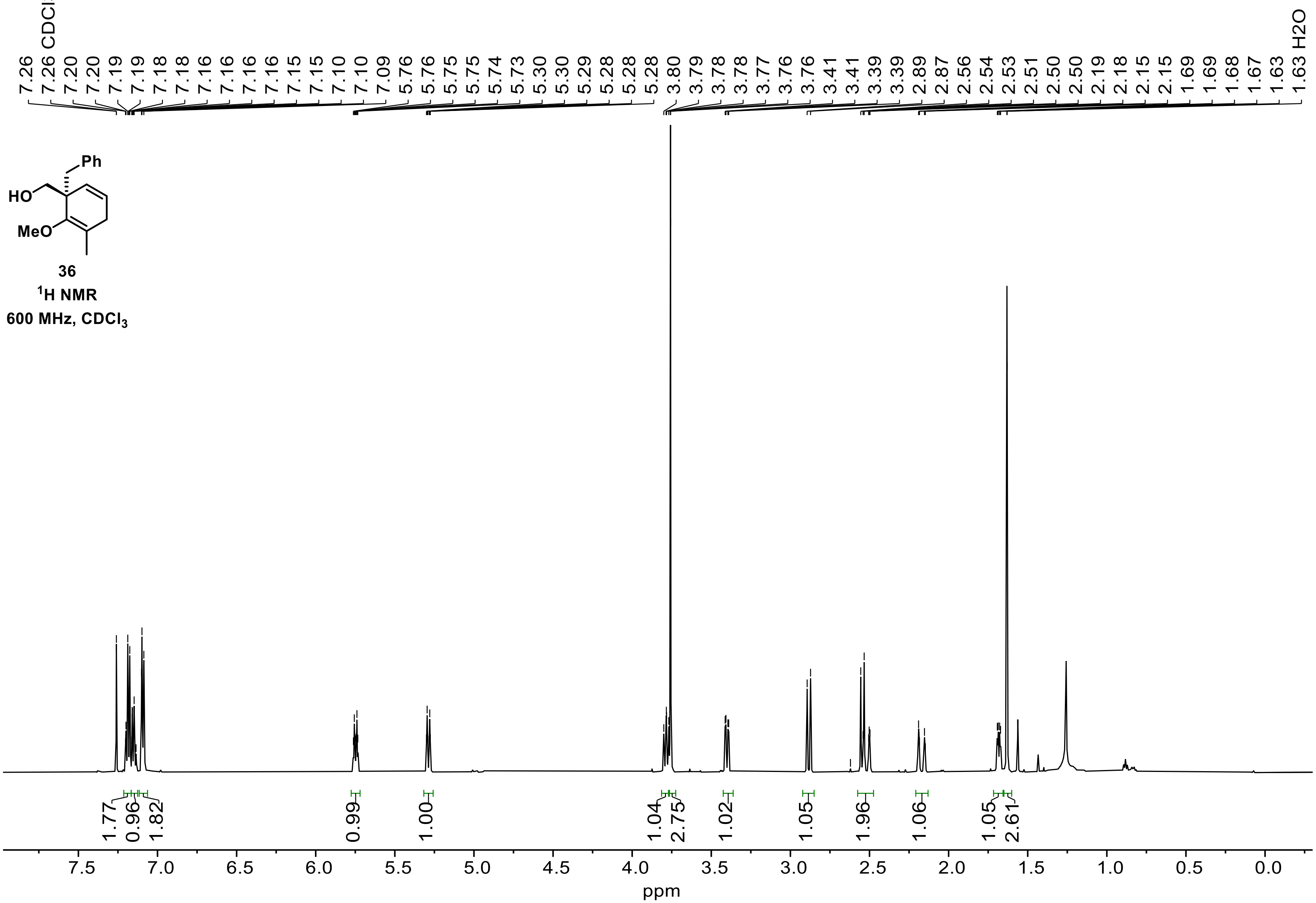

Ph
HO
MeO
36
1H NMR
600 MHz, CDCl3
7.26 CDCl3
H2O
ppm

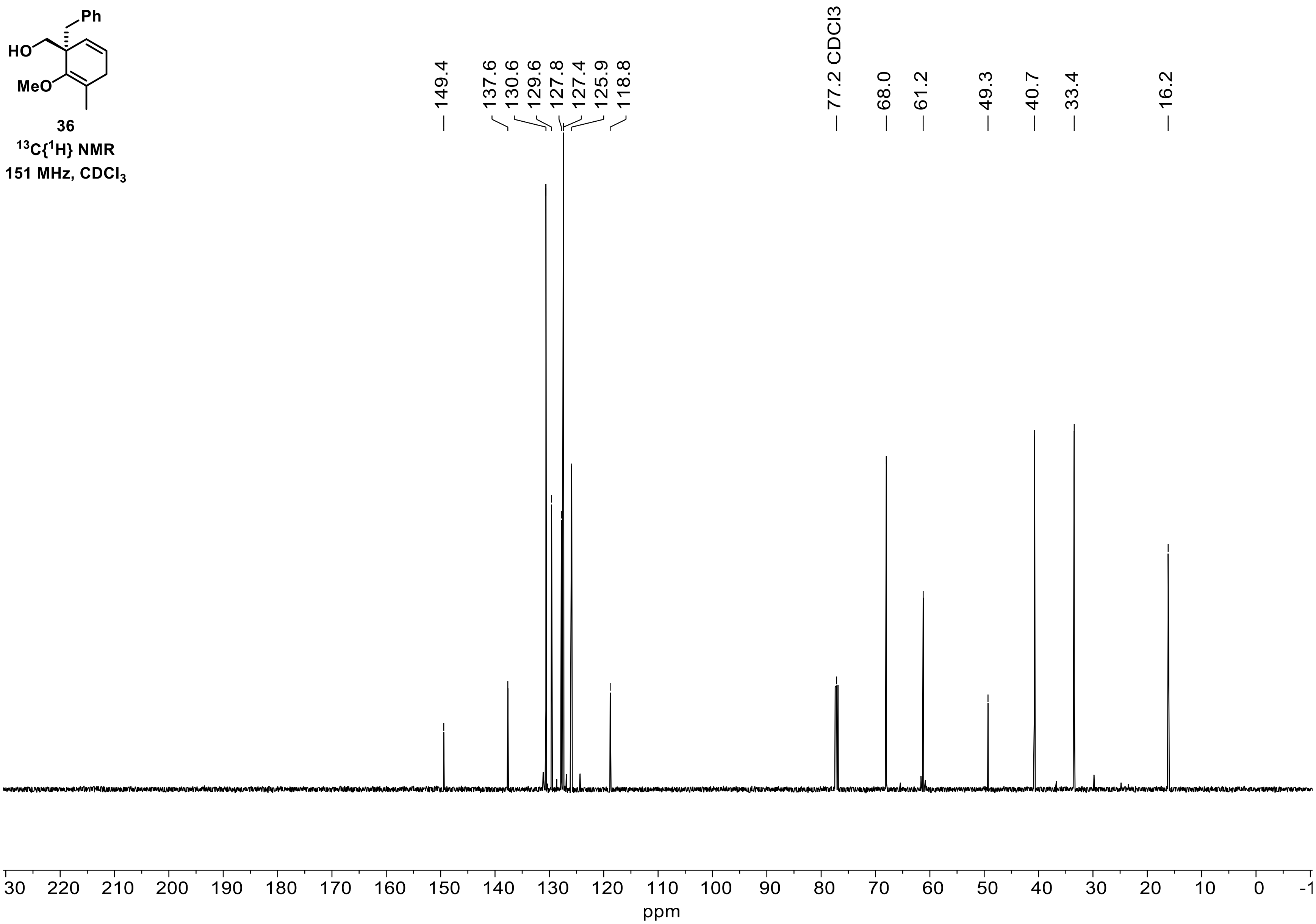
Ph
HO
MeO
36
13C{1H} NMR
151 MHz, CDCl3
149.4
137.6
130.6
129.6
127.8
127.4
125.9
118.8
77.2 CDCl3
68.0
61.2
49.3
40.7
33.4
16.2
ppm

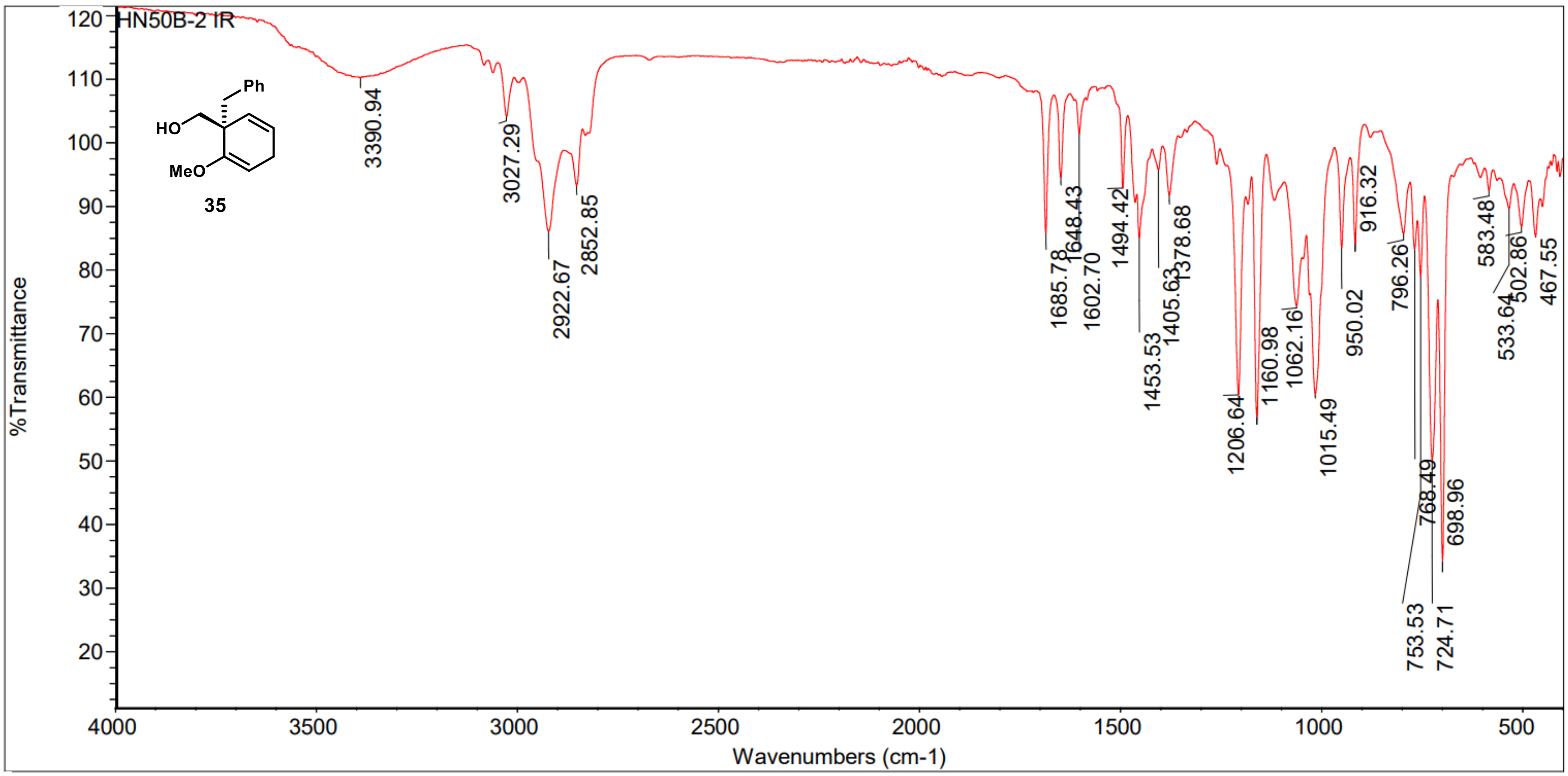
HN50B-2 IR
Ph
HO
MeO
35
%Transmittance
Wavenumbers (cm-1)
3390.94
3027.29
2922.67
2852.85
1685.78
1648.43
1602.70
1494.42
1453.53
1405.63
1378.68
1206.64
1160.98
1062.16
1015.49
950.02
916.32
796.26
768.49
753.53
724.71
698.96
583.48
533.64
502.86
467.55

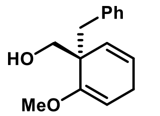

35

^{1}H NMR

600 MHz, $CDCl_3$

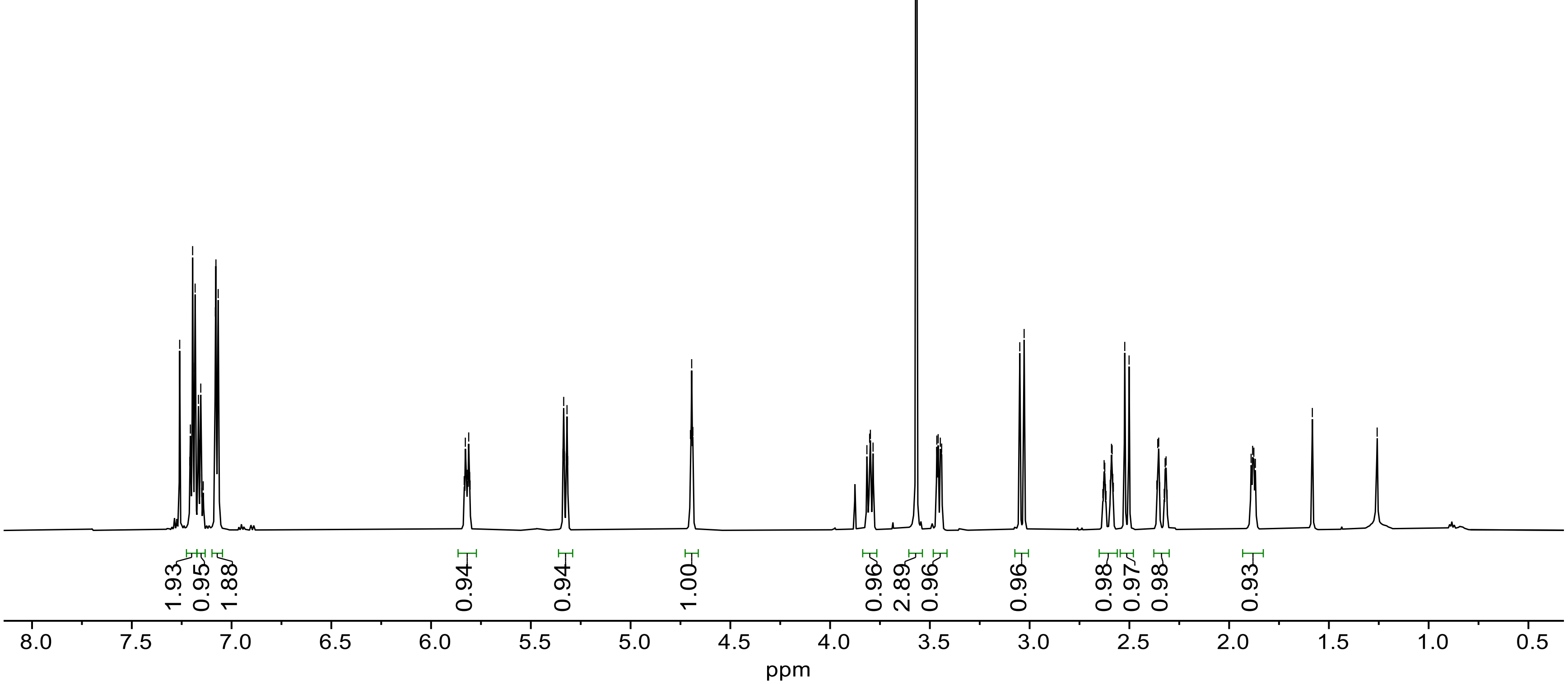

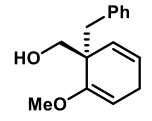

35

$^{13}C\{^{1}H\}$ NMR

151 MHz, $CDCl_3$

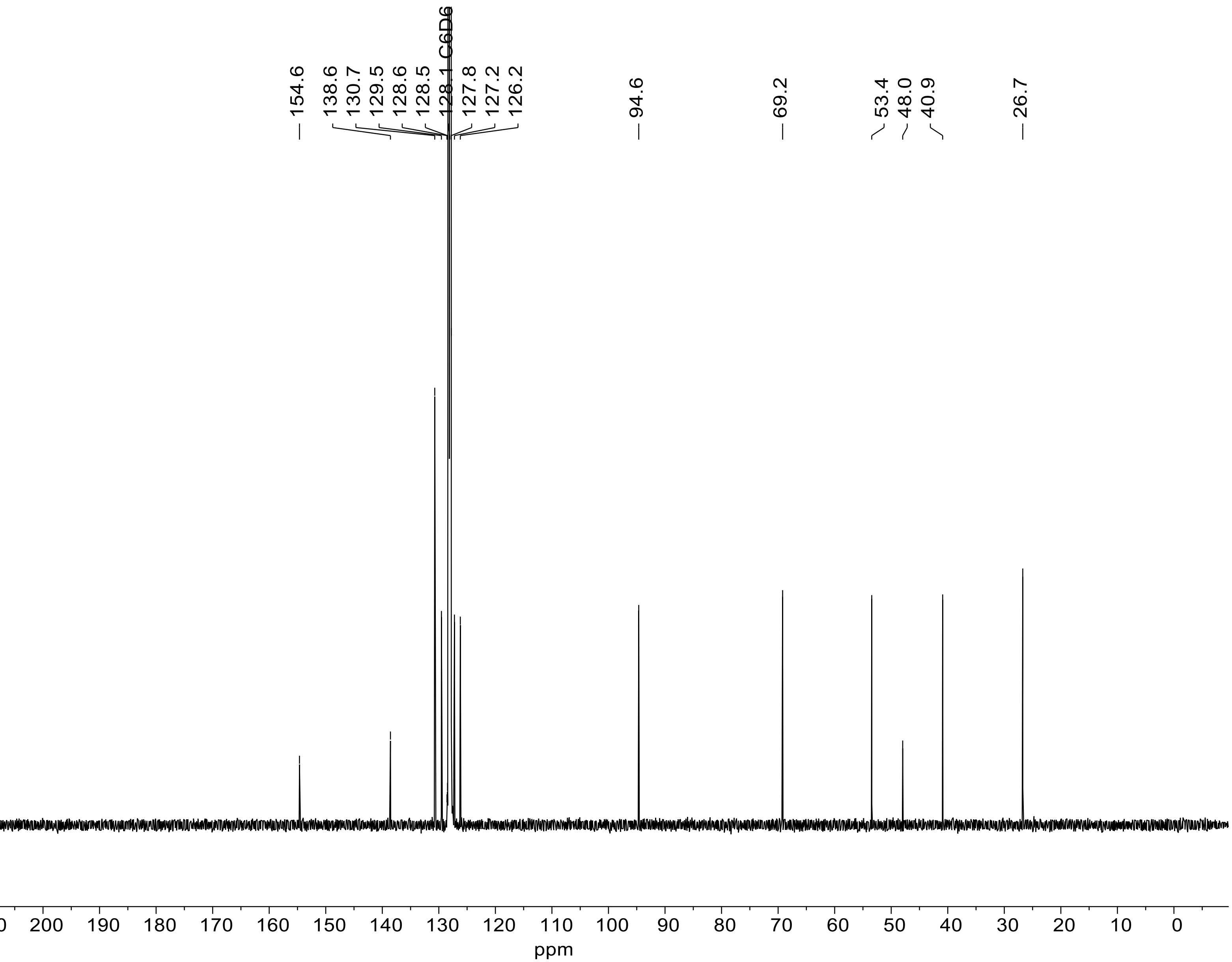

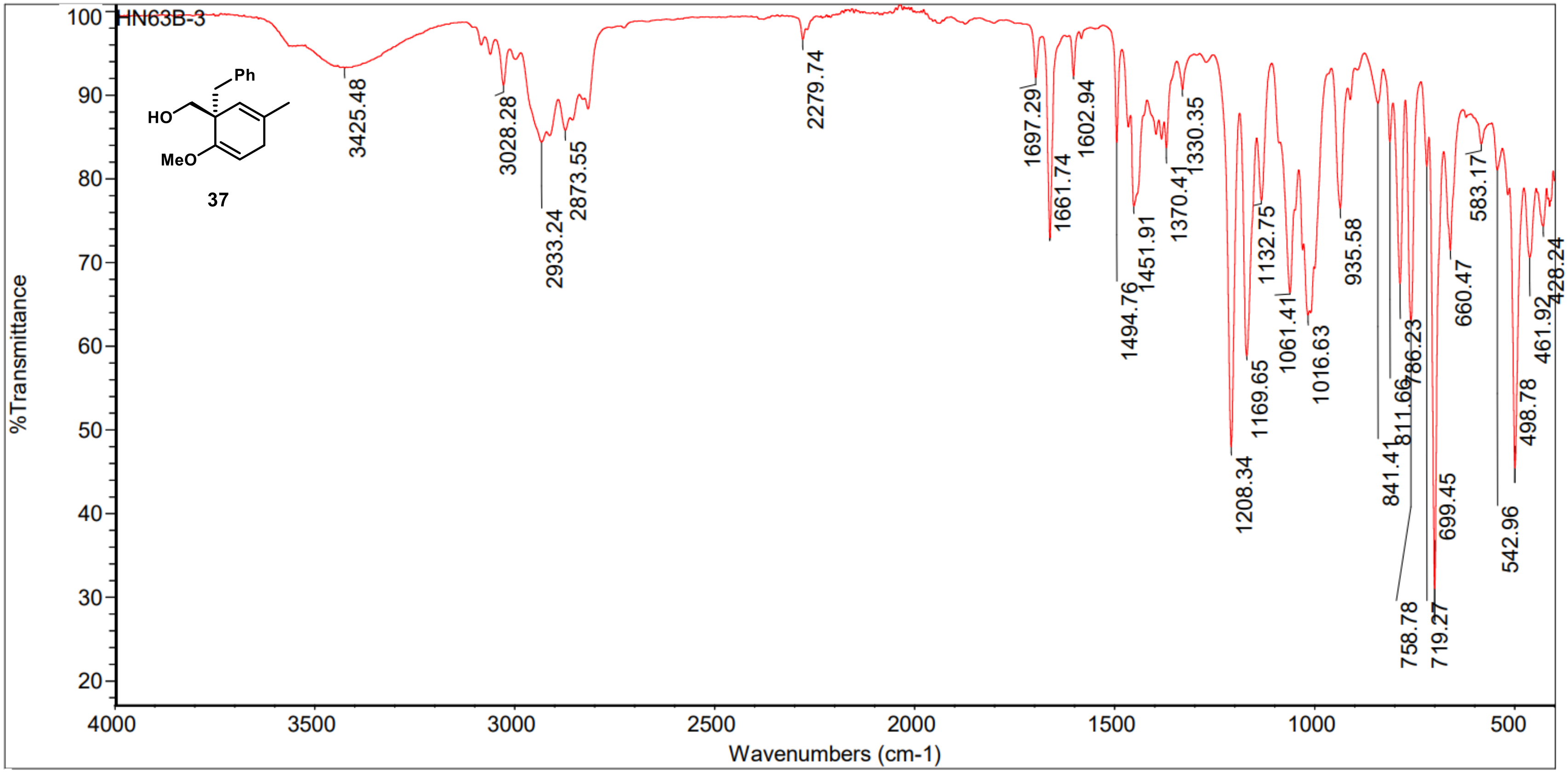

HN63B-3
HO
Ph
MeO
37
3425.48
3028.28
2933.24
2873.55
2279.74
1697.29
1661.74
1602.94
1494.76
1451.91
1370.41
1330.35
1208.34
1169.65
1132.75
1061.41
1016.63
935.58
841.41
811.66
786.23
758.78
719.27
699.45
660.47
583.17
542.96
498.78
461.92
428.24
%Transmittance
Wavenumbers (cm-1)

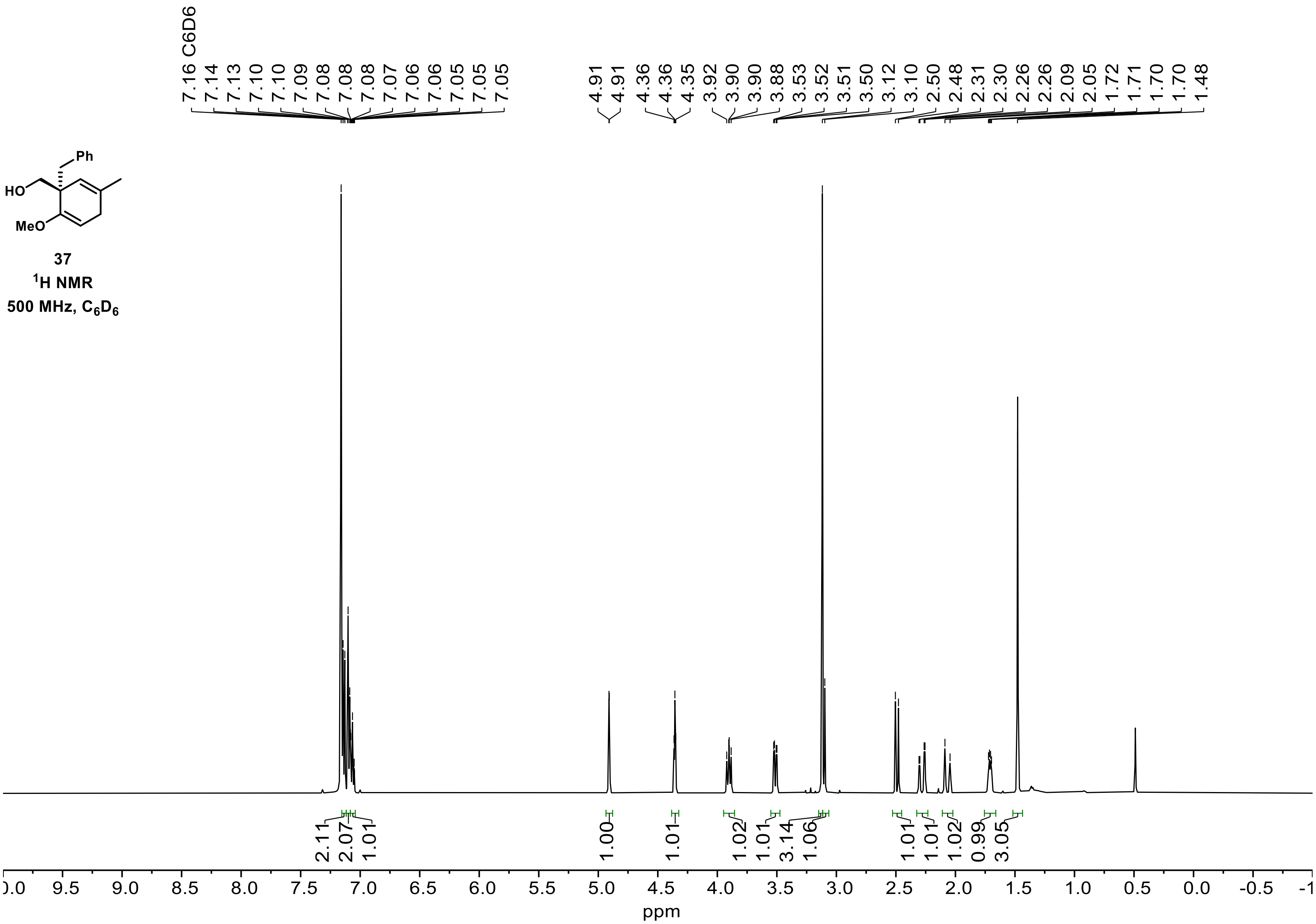
7.16 C6D6
7.14
7.13
7.10
7.10
7.09
7.08
7.08
7.08
7.07
7.06
7.06
7.05
7.05
7.05
4.91
4.91
4.36
4.36
4.35
3.92
3.90
3.90
3.88
3.53
3.52
3.51
3.50
3.12
3.10
2.50
2.48
2.31
2.30
2.26
2.26
2.09
2.05
1.72
1.71
1.70
1.70
1.48
Ph
HO
MeO
37
^{1}H NMR
500 MHz, C_6D_6
2.11
2.07
1.01
1.00
1.01
1.02
1.01
3.14
1.06
1.01
1.01
1.02
0.99
3.05
ppm

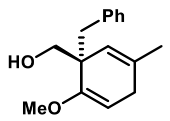

37

$^{13}C\{^1H\}$ NMR

151 MHz, C_6D_6

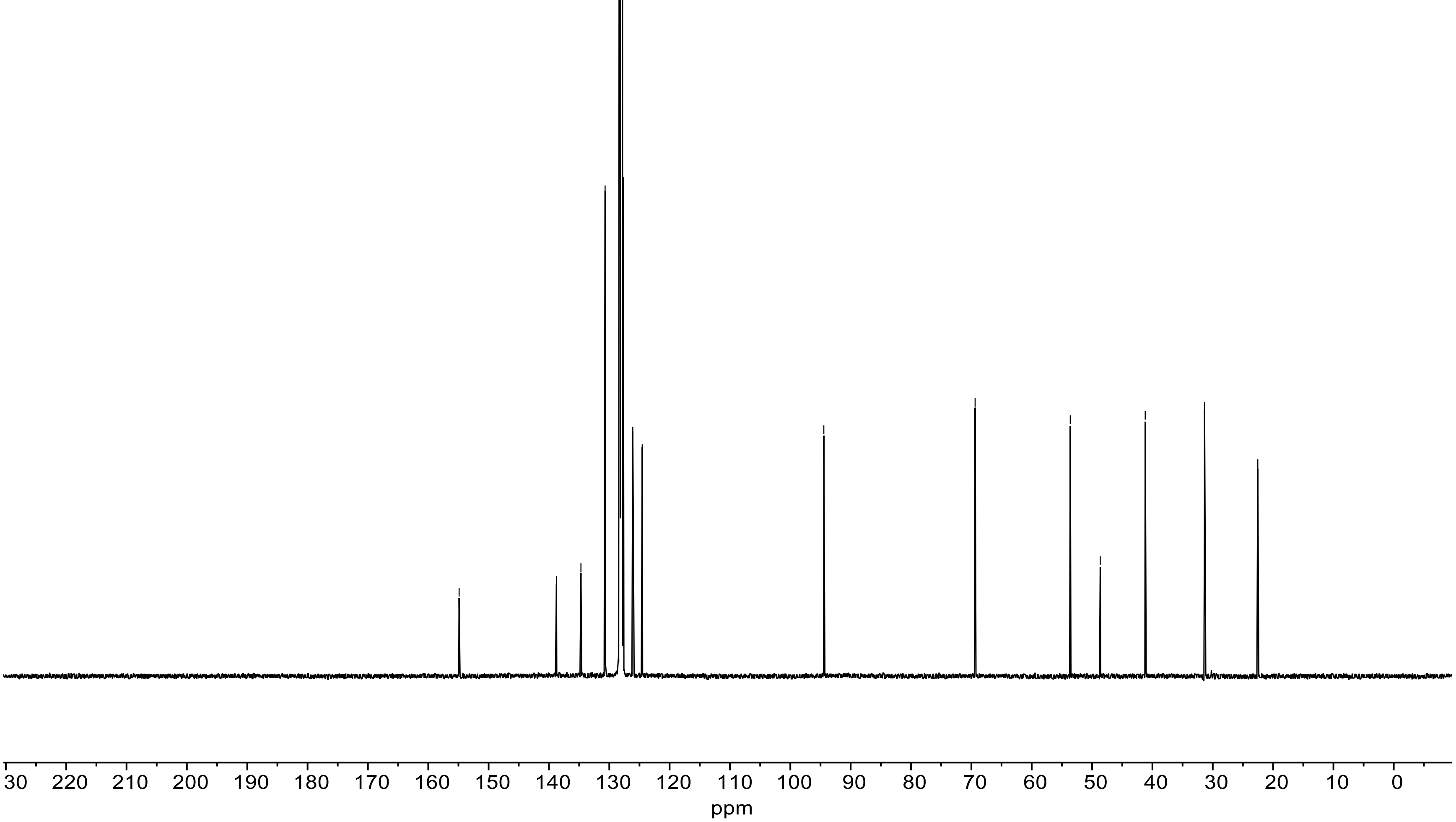

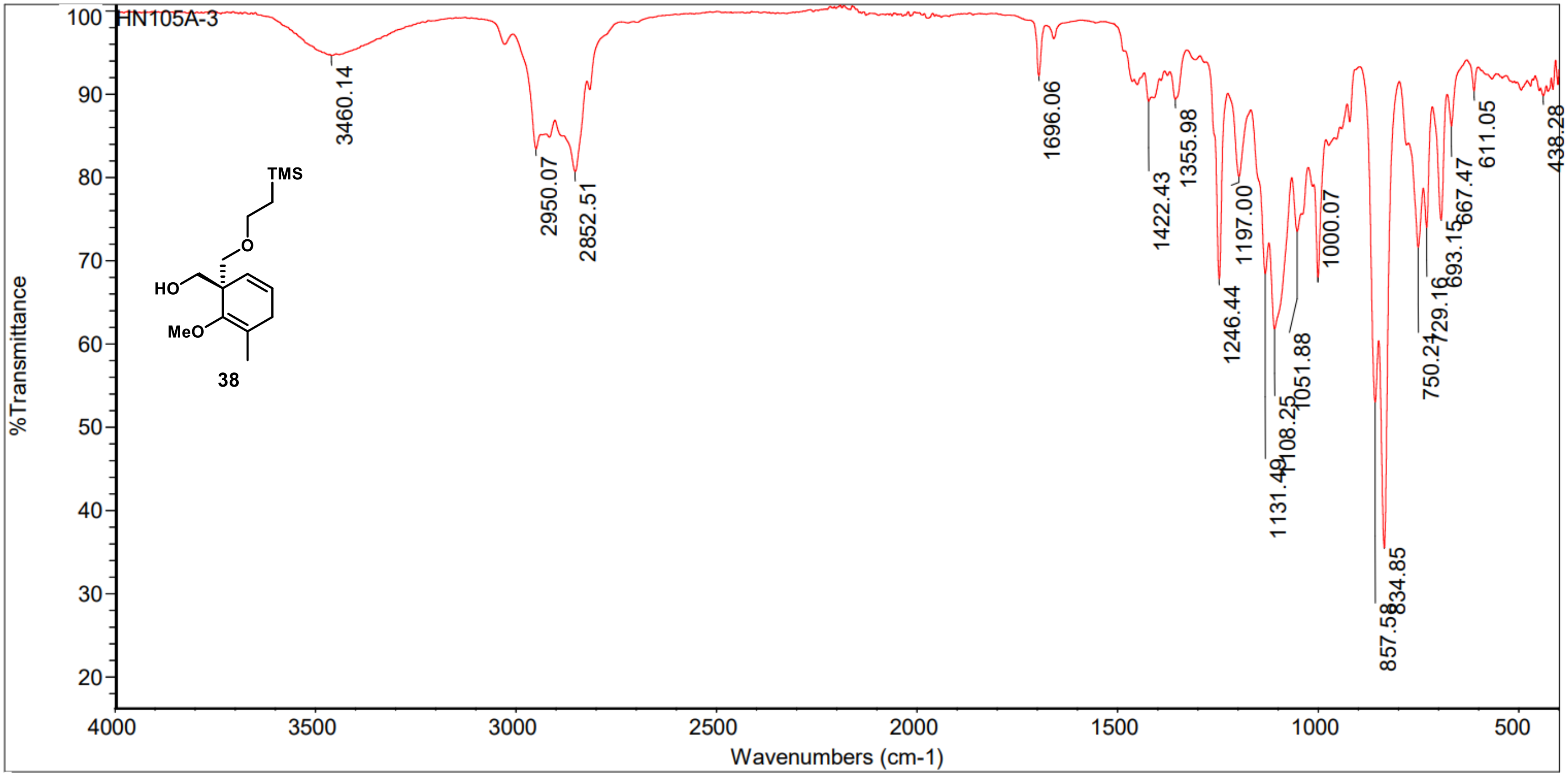

HN105A-3
%Transmittance
Wavenumbers (cm-1)
3460.14
2950.07
2852.51
1696.06
1422.43
1355.98
1246.44
1197.00
1131.49
1108.25
1051.88
1000.07
857.58
834.85
750.21
729.16
693.15
667.47
611.05
438.28
TMS
O
HO
MeO
38

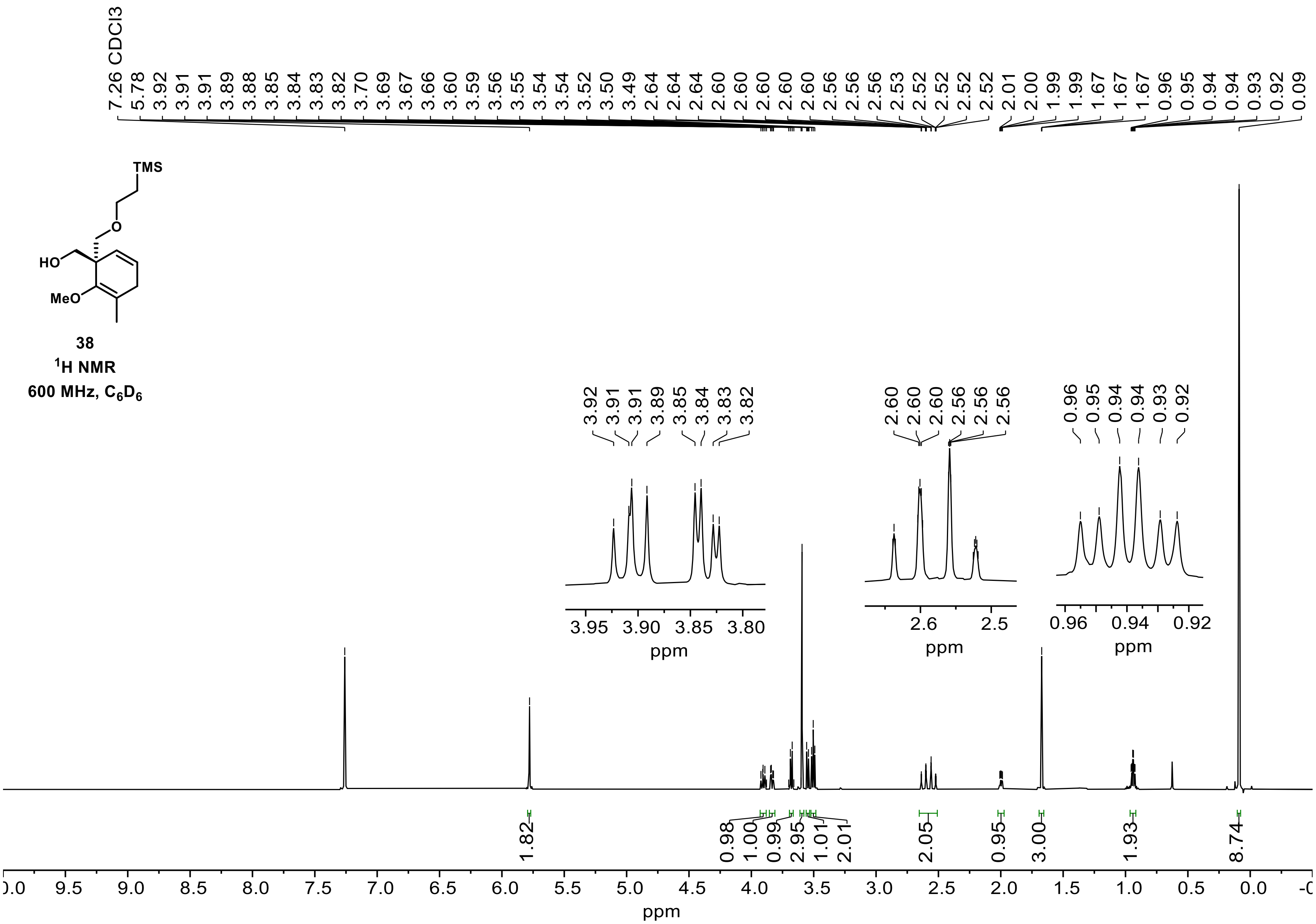
7.26 CDCl3
TMS
HO
O
MeO
38
¹H NMR
600 MHz, C6D6
ppm

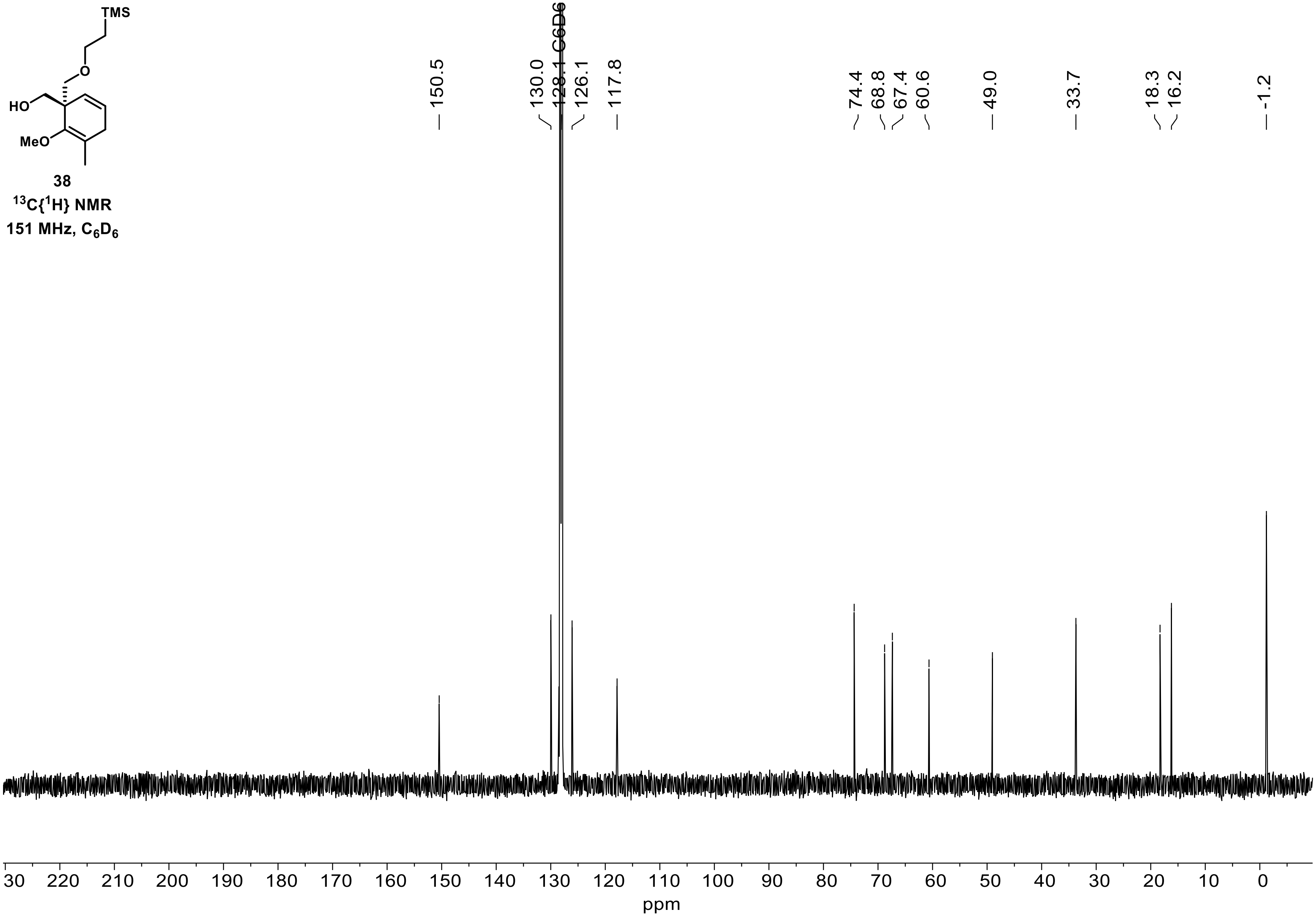
TMS
HO
MeO
O
38
$^{13}C\{^1H\}$ NMR
151 MHz, C_6D_6
150.5
130.0
128.1 C6D6
126.1
117.8
74.4
68.8
67.4
60.6
49.0
33.7
18.3
16.2
-1.2
ppm

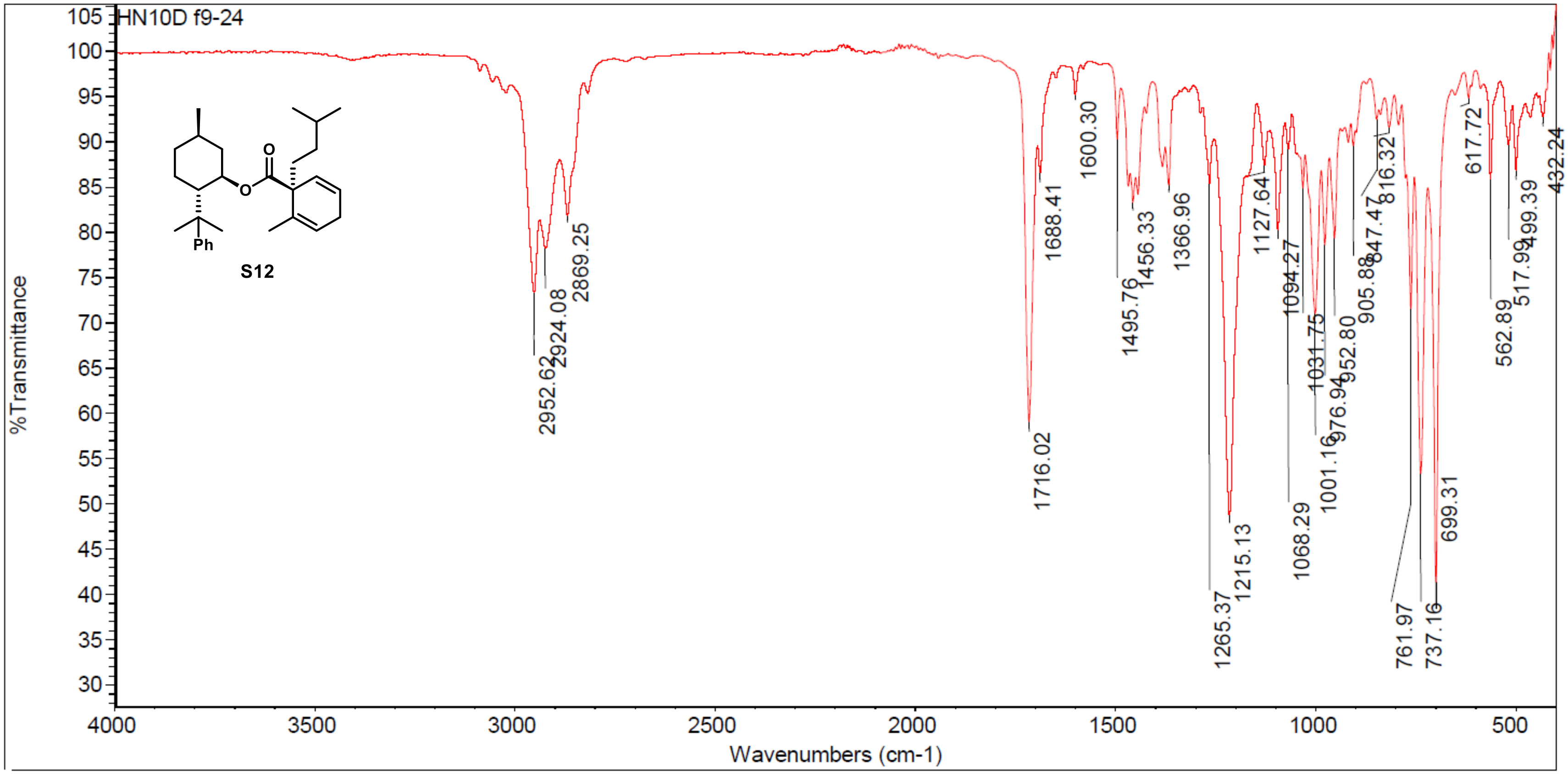
HN10D f9-24
%Transmittance
Wavenumbers (cm-1)
S12
Ph
O
2952.62
2924.08
2869.25
1716.02
1688.41
1600.30
1495.76
1456.33
1366.96
1265.37
1215.13
1127.64
1094.27
1068.29
1031.75
1001.16
976.94
952.80
905.88
847.47
816.32
761.97
737.16
699.31
617.72
562.89
517.99
499.39
432.24

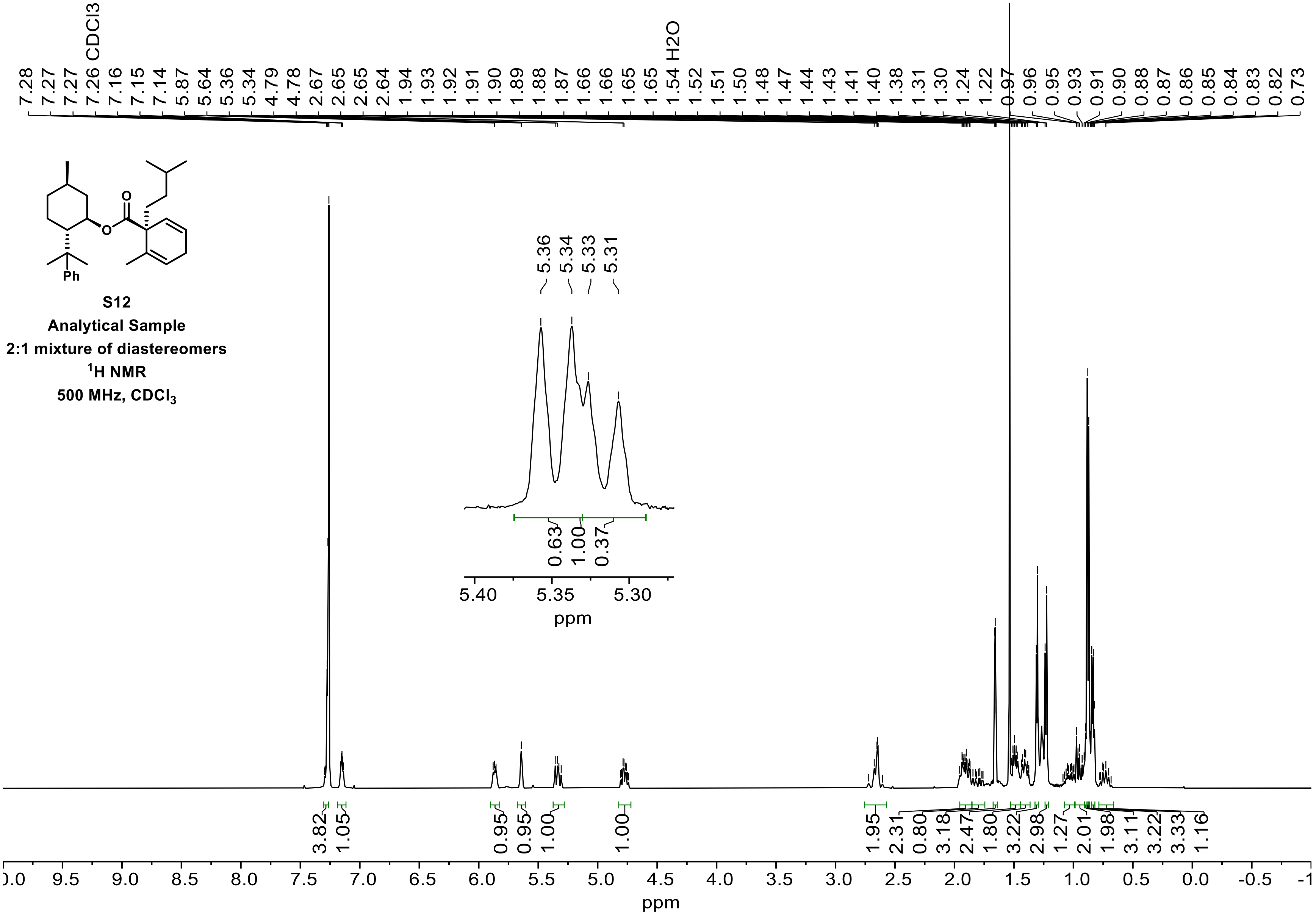
7.28 7.27 7.27 7.26 CDCl3 7.16 7.15 7.14 5.87 5.64 5.36 5.34 4.79 4.78 2.67 2.65 2.65 2.64 1.94 1.93 1.92 1.91 1.90 1.89 1.88 1.87 1.66 1.66 1.65 1.65 1.54 H2O 1.52 1.51 1.50 1.48 1.47 1.44 1.43 1.41 1.40 1.38 1.31 1.30 1.24 1.22 0.97 0.96 0.95 0.93 0.91 0.90 0.88 0.87 0.86 0.85 0.84 0.83 0.82 0.73
O
Ph
S12
Analytical Sample
2:1 mixture of diastereomers
^{1}H NMR
500 MHz, CDCl$_3$
5.36 5.34 5.33 5.31
0.63 1.00 0.37
5.40 5.35 5.30
ppm
3.82 1.05 0.95 0.95 1.00 1.00 1.95 2.31 0.80 3.18 2.47 1.80 3.22 2.98 1.27 2.01 1.98 3.11 3.22 3.33 1.16
ppm

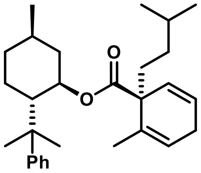

S12
2:1 mixture of diastereomers
$^{13}C\{^{1}H\}$ NMR
126 MHz, $CDCl_3$

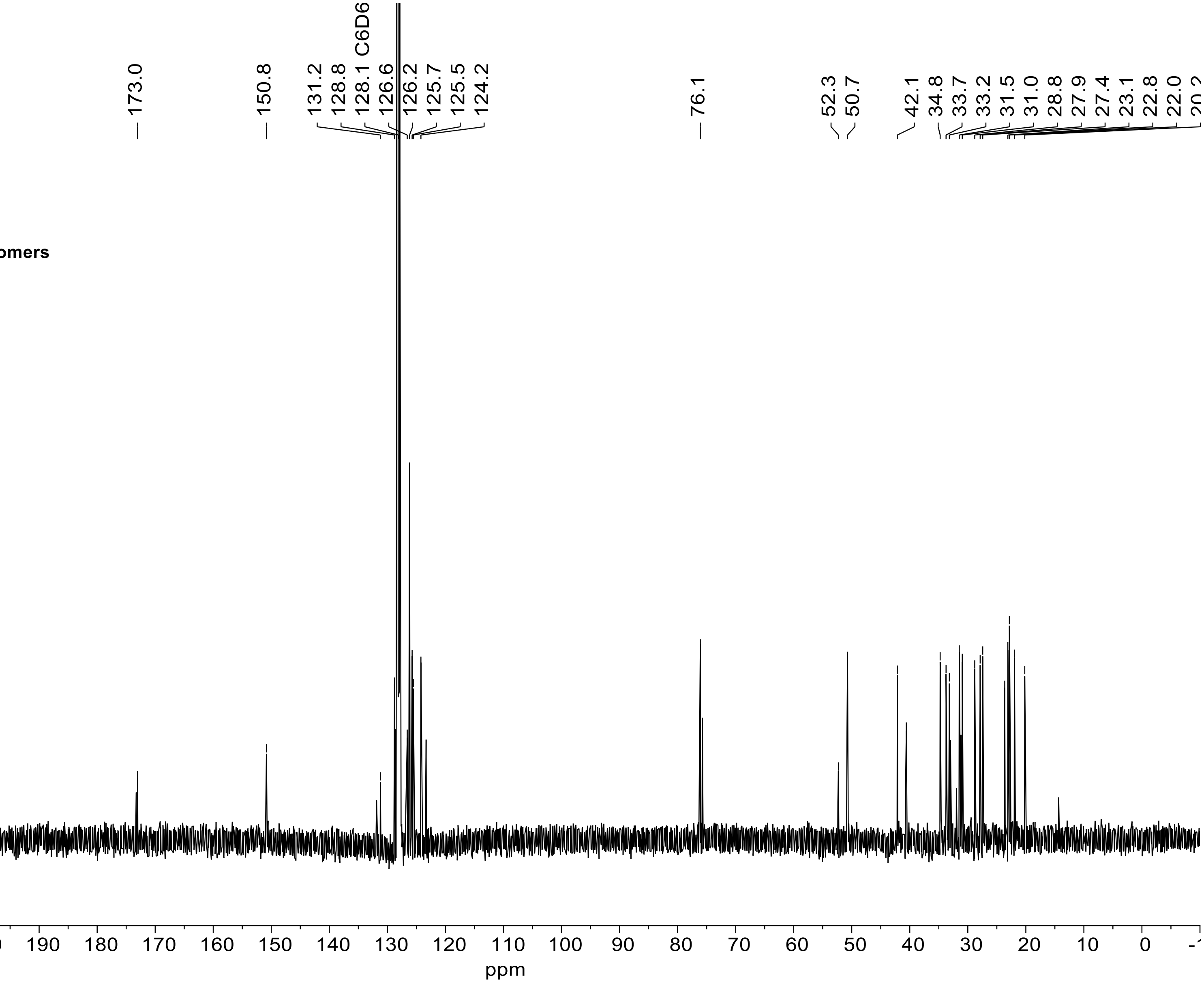

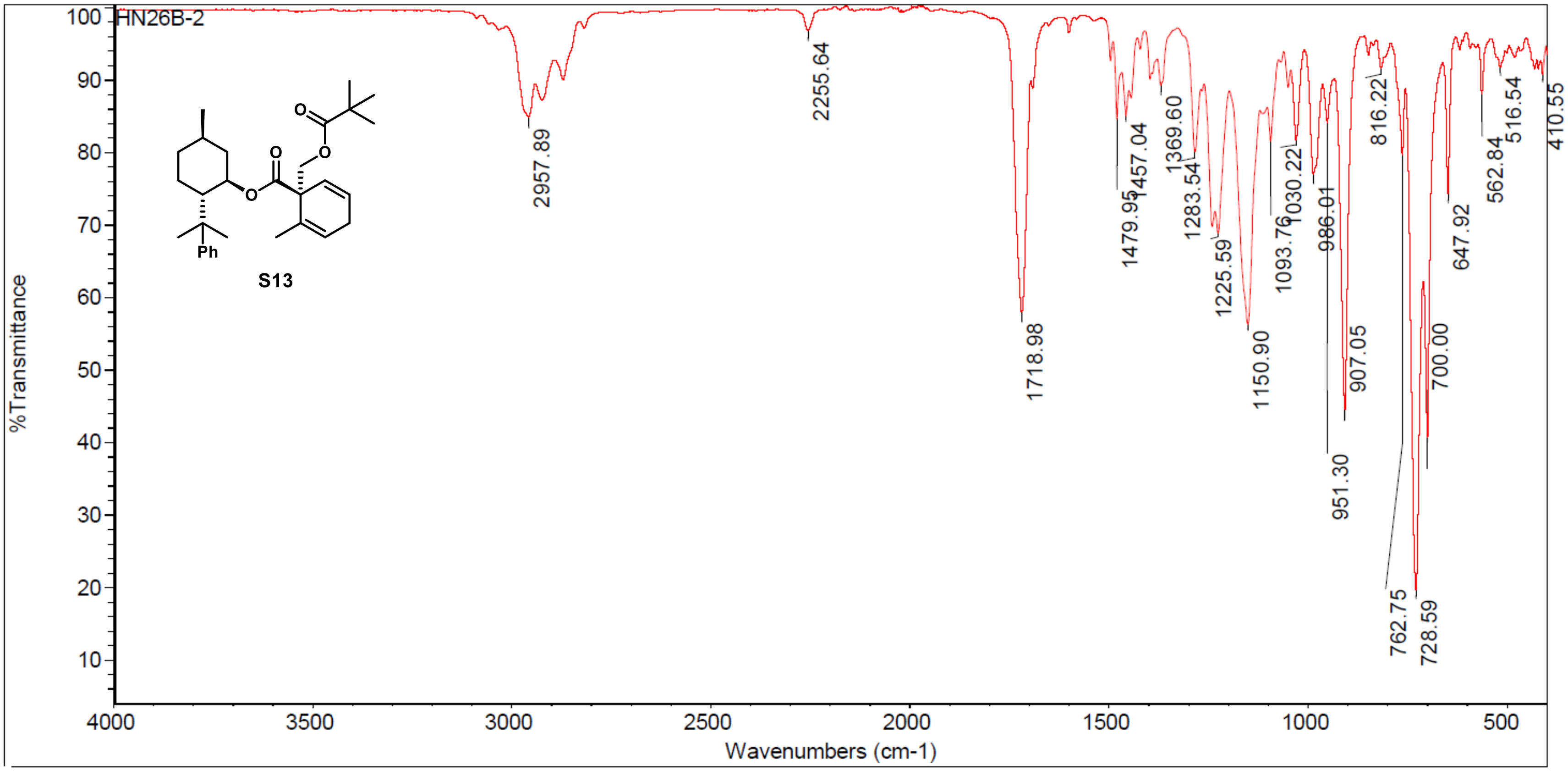
HN26B-2
S13
Ph
O
2957.89
2255.64
1718.98
1479.95
1457.04
1369.60
1283.54
1225.59
1150.90
1093.76
1030.22
986.01
951.30
907.05
816.22
762.75
728.59
700.00
647.92
562.84
516.54
410.55
%Transmittance
Wavenumbers (cm-1)
100
90
80
70
60
50
40
30
20
10
4000
3500
3000
2500
2000
1500
1000
500

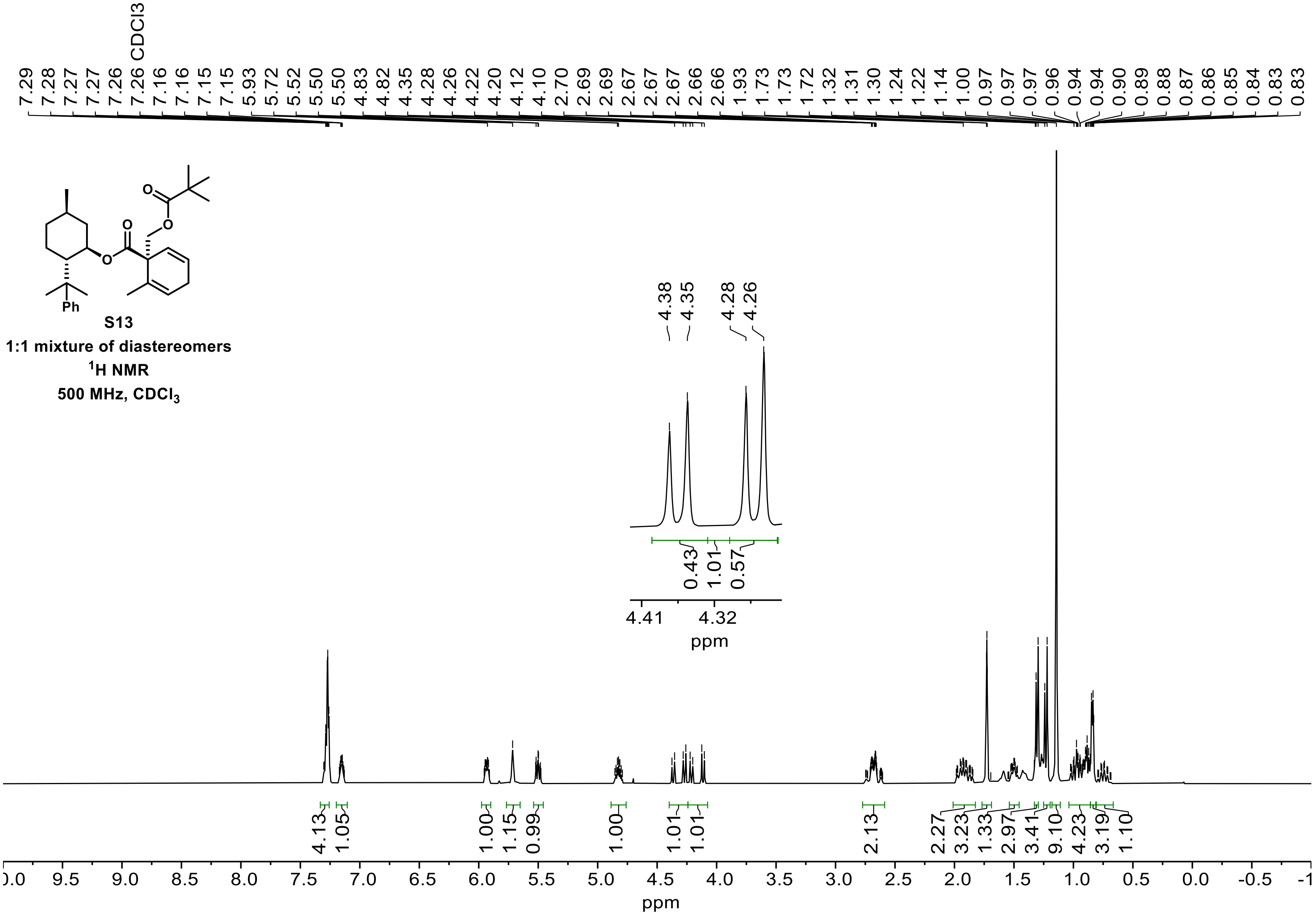
S13
1:1 mixture of diastereomers
^{1}H NMR
500 MHz, $CDCl_3$
ppm

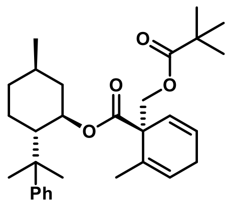

S13
1:1 mixture of diastereomers
$^{13}C\{^{1}H\}$ NMR
126 MHz, $CDCl_3$

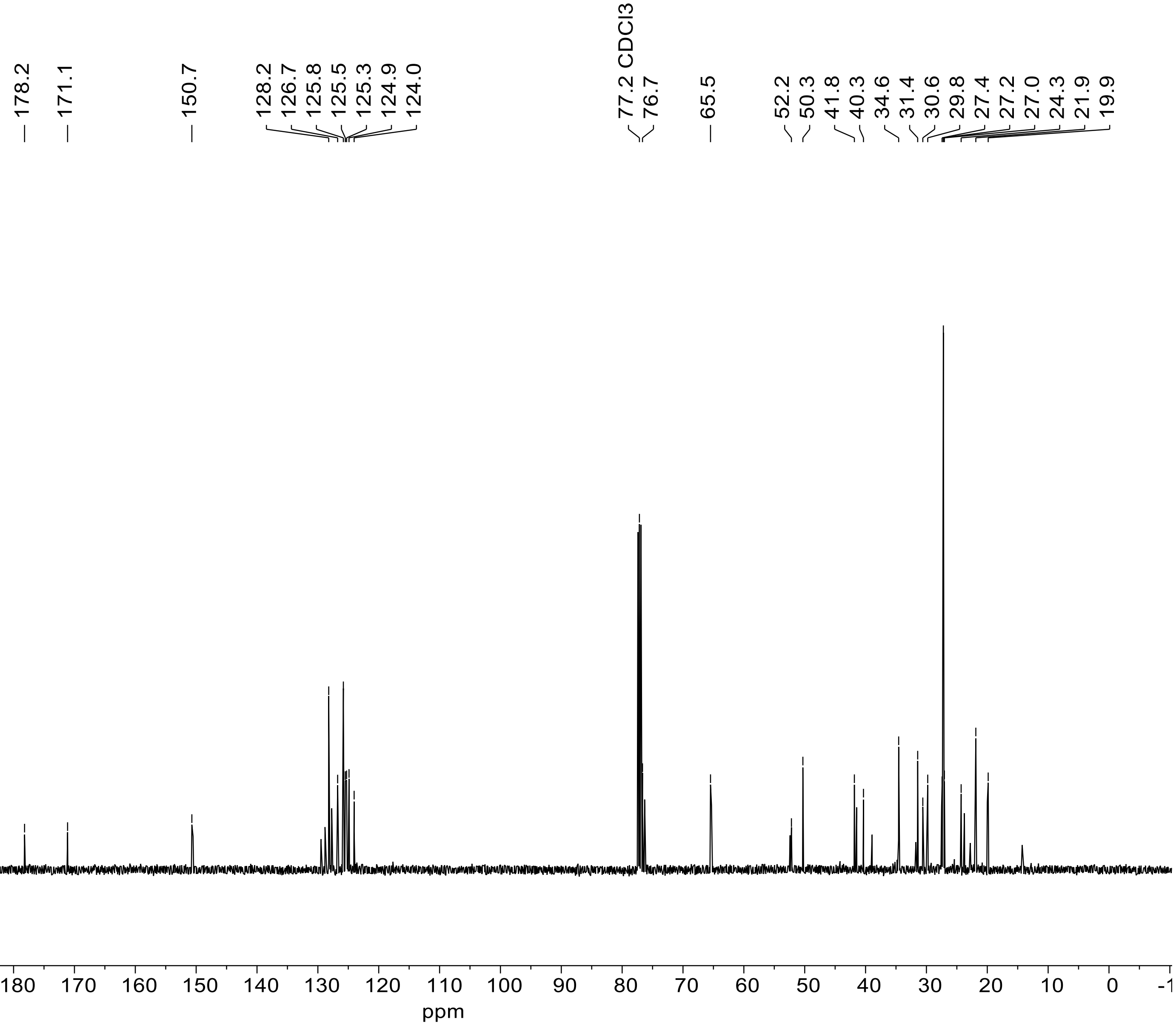

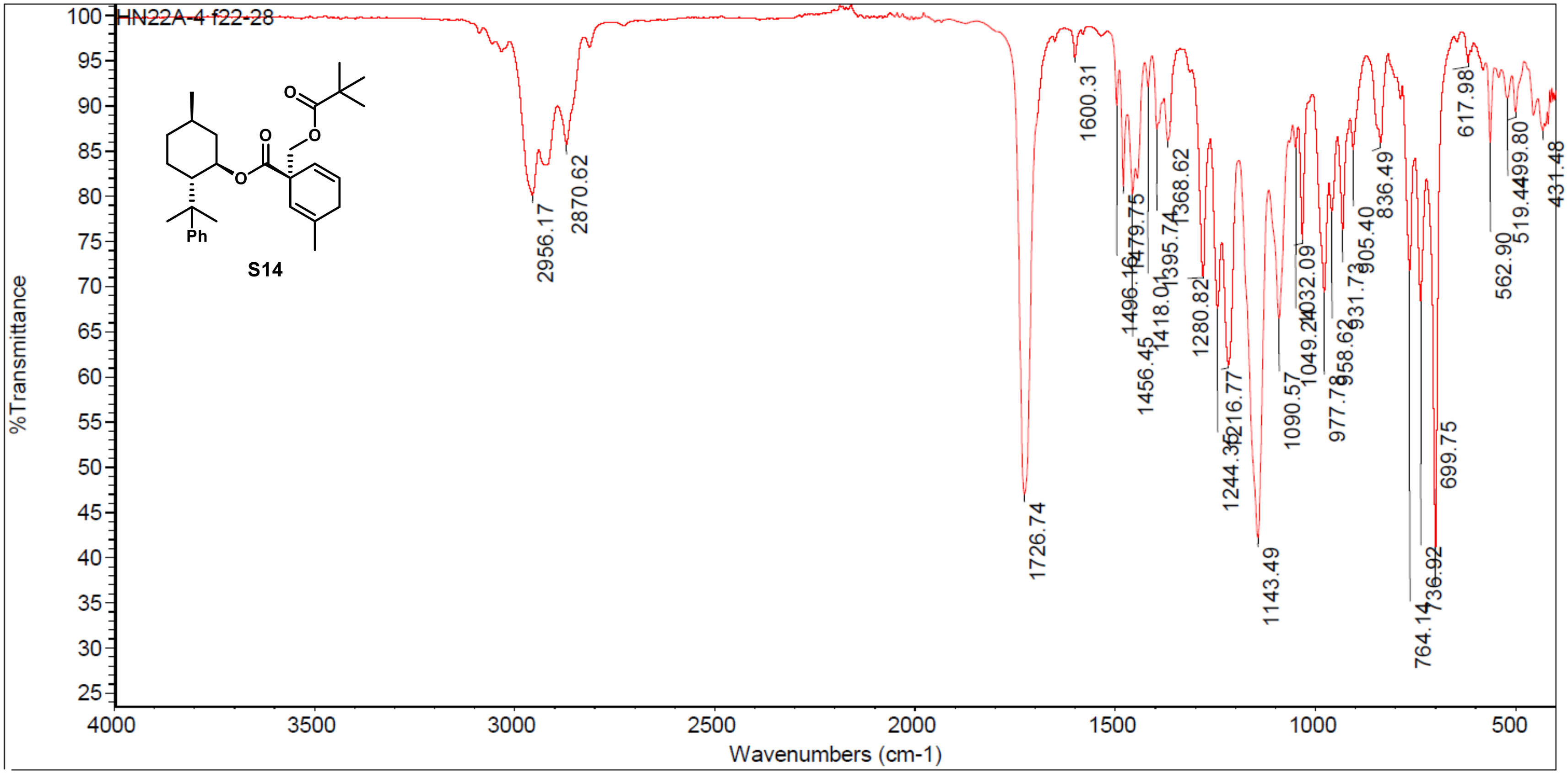
HN22A-4 f22-28
S14
Ph
O
%Transmittance
Wavenumbers (cm-1)
4000
3500
3000
2500
2000
1500
1000
500
100
95
90
85
80
75
70
65
60
55
50
45
40
35
30
25
2956.17
2870.62
1726.74
1600.31
1496.16
1479.75
1456.45
1418.01
1395.74
1368.62
1280.82
1244.35
1216.77
1143.49
1090.57
1049.24
1032.09
977.78
958.62
931.73
905.40
836.49
764.14
736.92
699.75
617.98
562.90
519.44
499.80
431.48

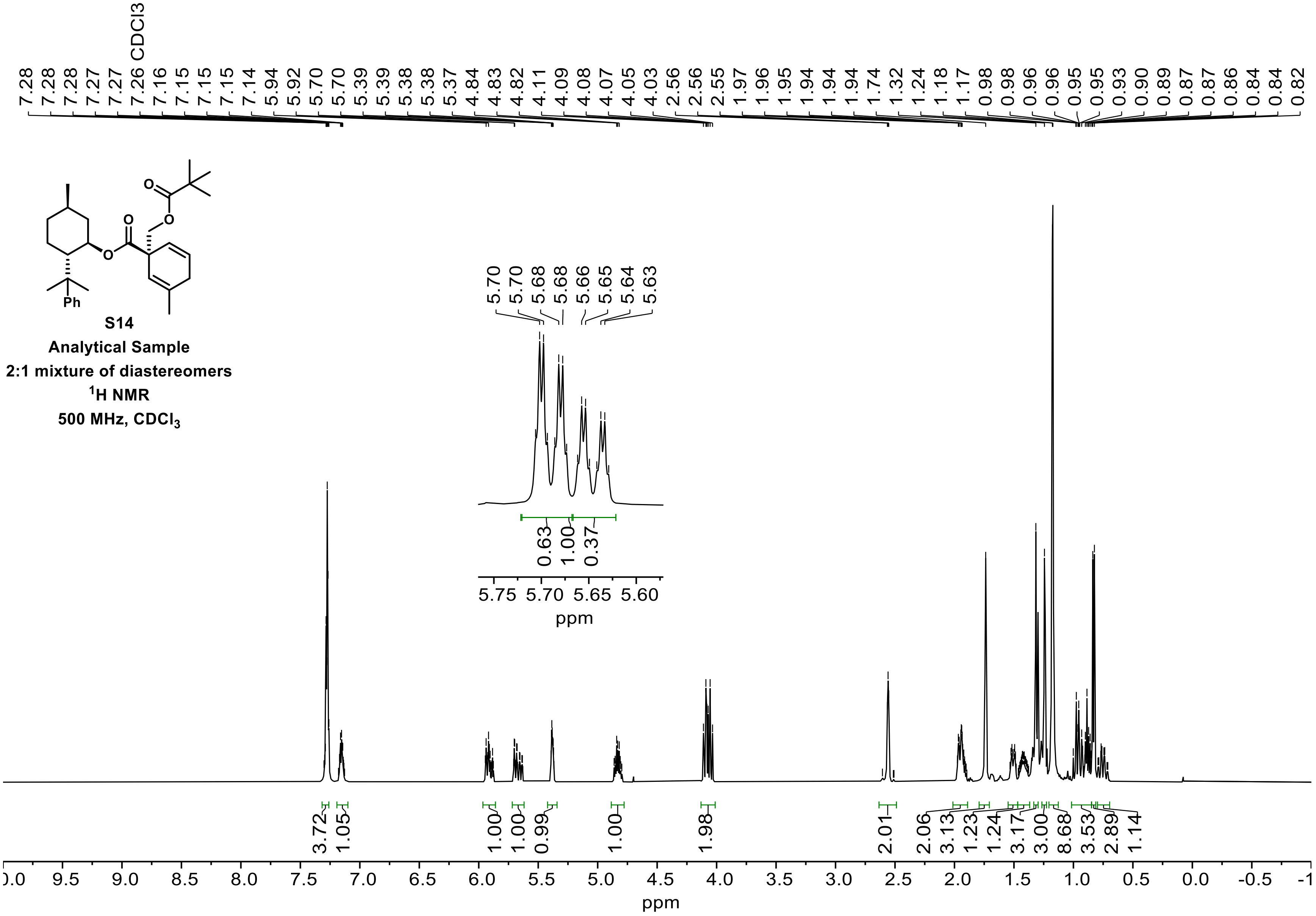
S14
Analytical Sample
2:1 mixture of diastereomers
^{1}H NMR
500 MHz, $CDCl_3$
ppm

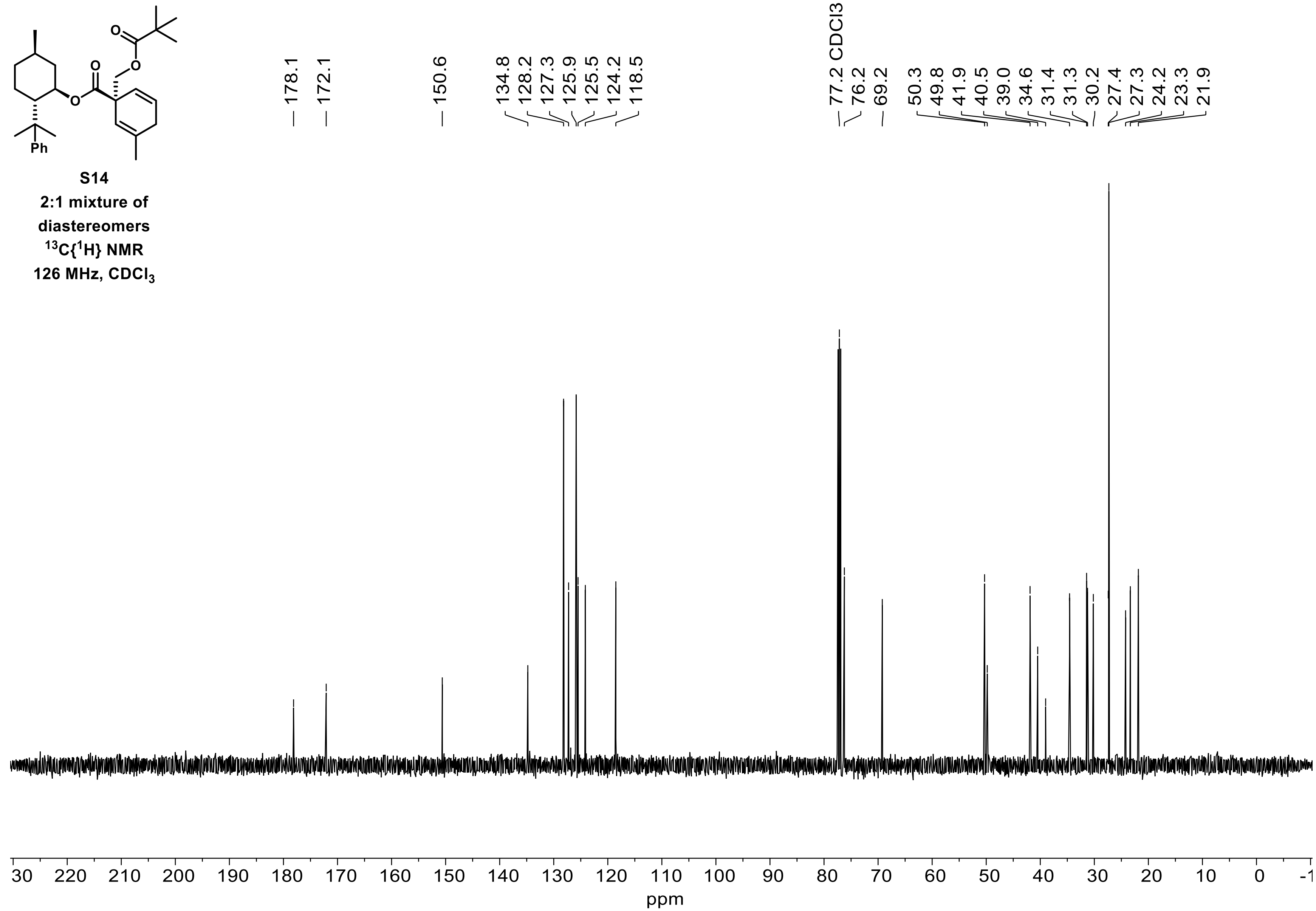

S14
2:1 mixture of diastereomers
$^{13}C\{^1H\}$ NMR
126 MHz, $CDCl_3$
178.1
172.1
150.6
134.8
128.2
127.3
125.9
125.5
124.2
118.5
77.2 CDCl3
76.2
69.2
50.3
49.8
41.9
40.5
39.0
34.6
31.4
31.3
30.2
27.4
27.3
24.2
23.3
21.9
ppm

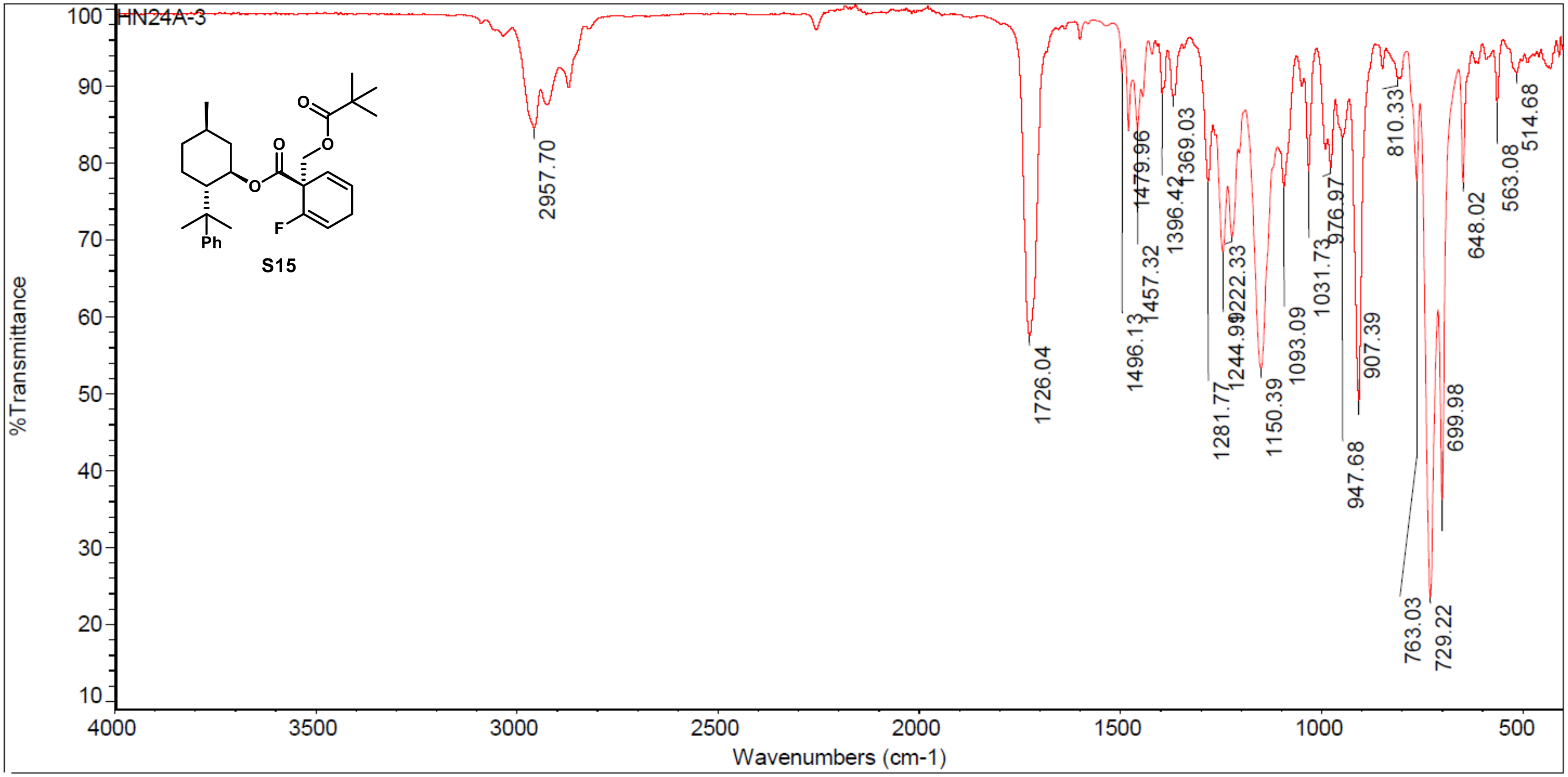

HN24A-3
%Transmittance
Wavenumbers (cm-1)
S15
2957.70
1726.04
1496.13
1479.96
1457.32
1396.42
1369.03
1281.77
1244.99
1222.33
1150.39
1093.09
1031.73
976.97
947.68
907.39
810.33
763.03
729.22
699.98
648.02
563.08
514.68

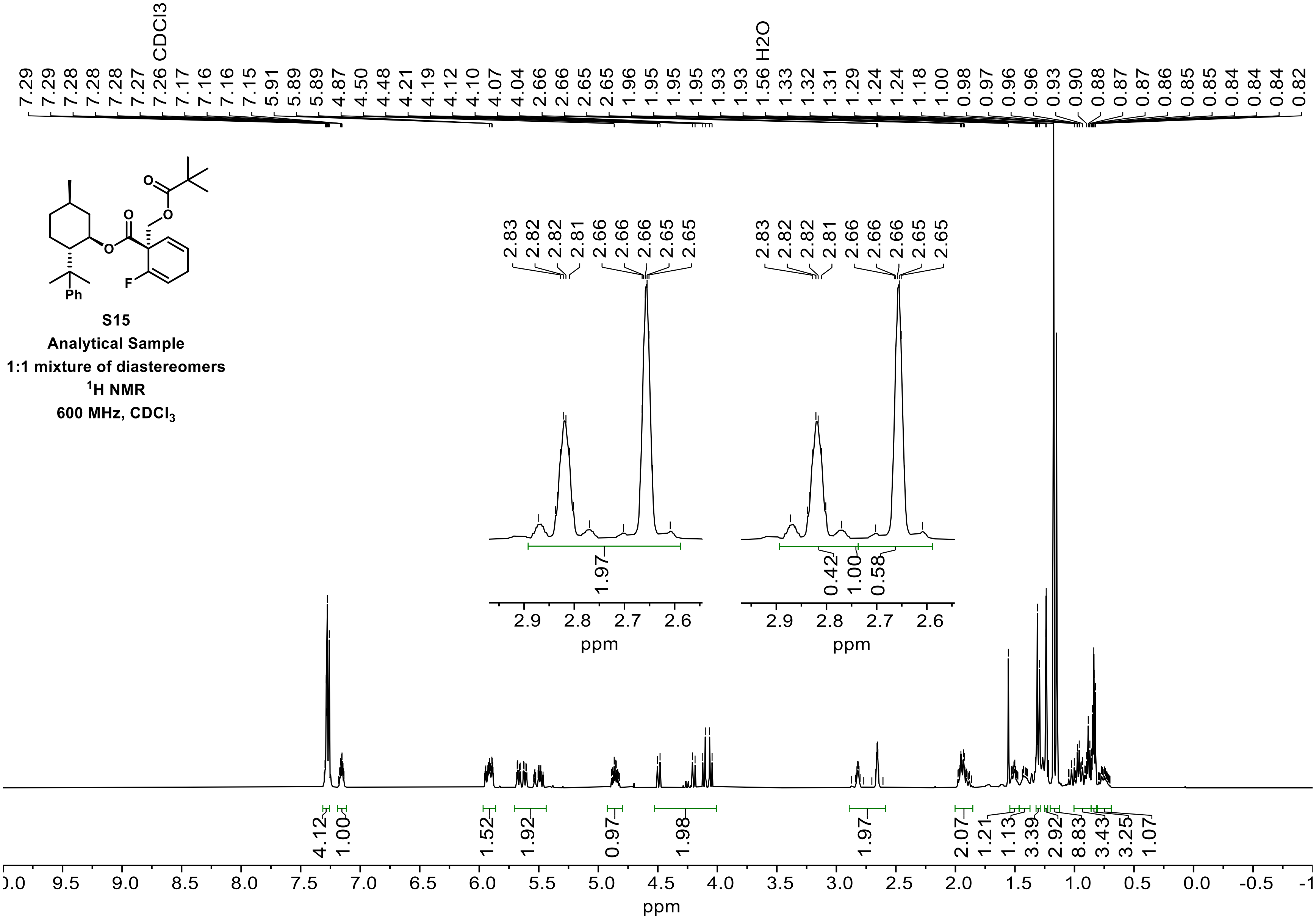

S15
Analytical Sample
1:1 mixture of diastereomers
^{1}H NMR
600 MHz, $CDCl_3$
ppm

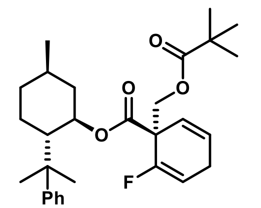

S15
1:1 mixture of diastereomers
$^{13}C\{^{1}H\}$ NMR
151 MHz, $CDCl_3$

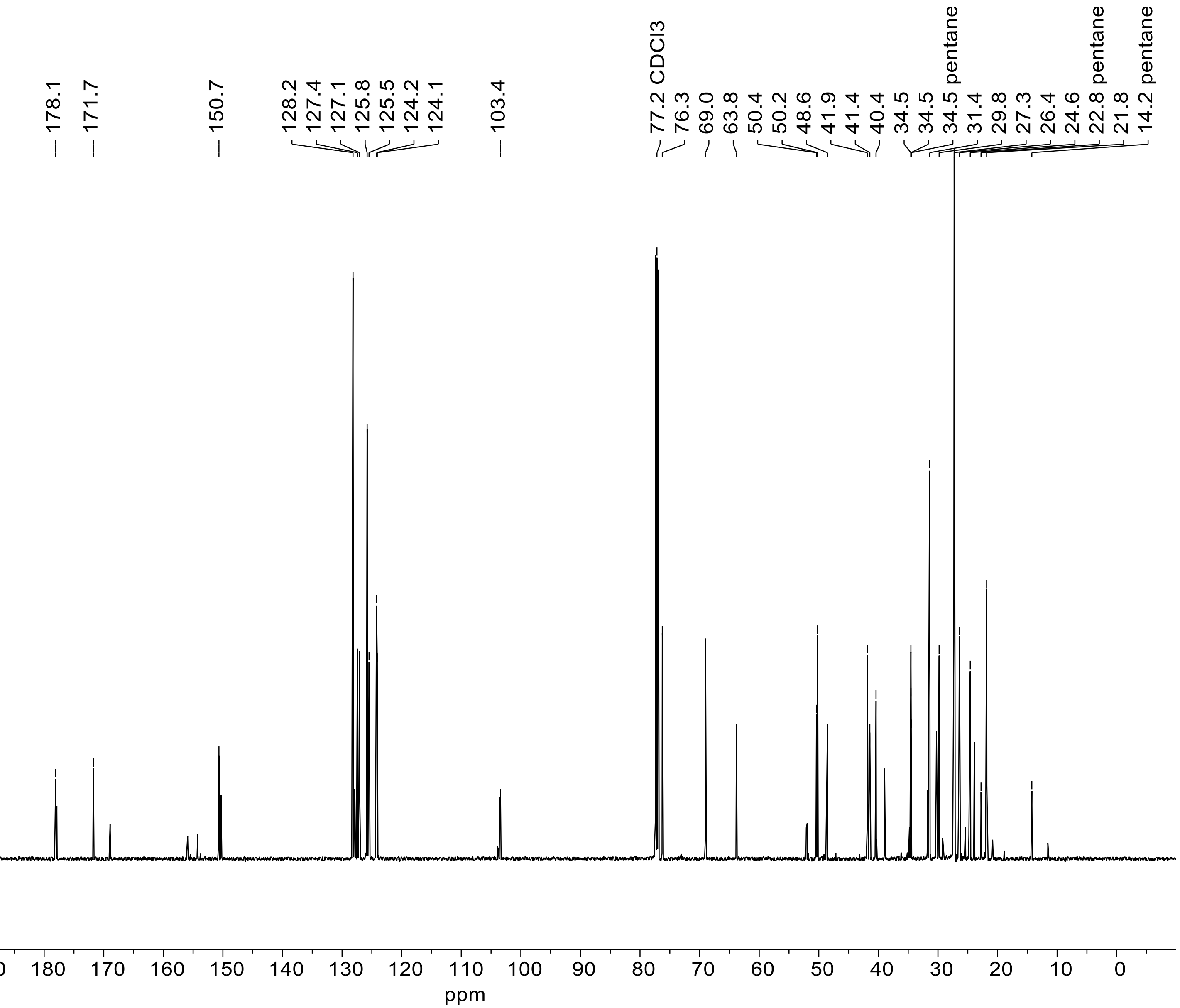

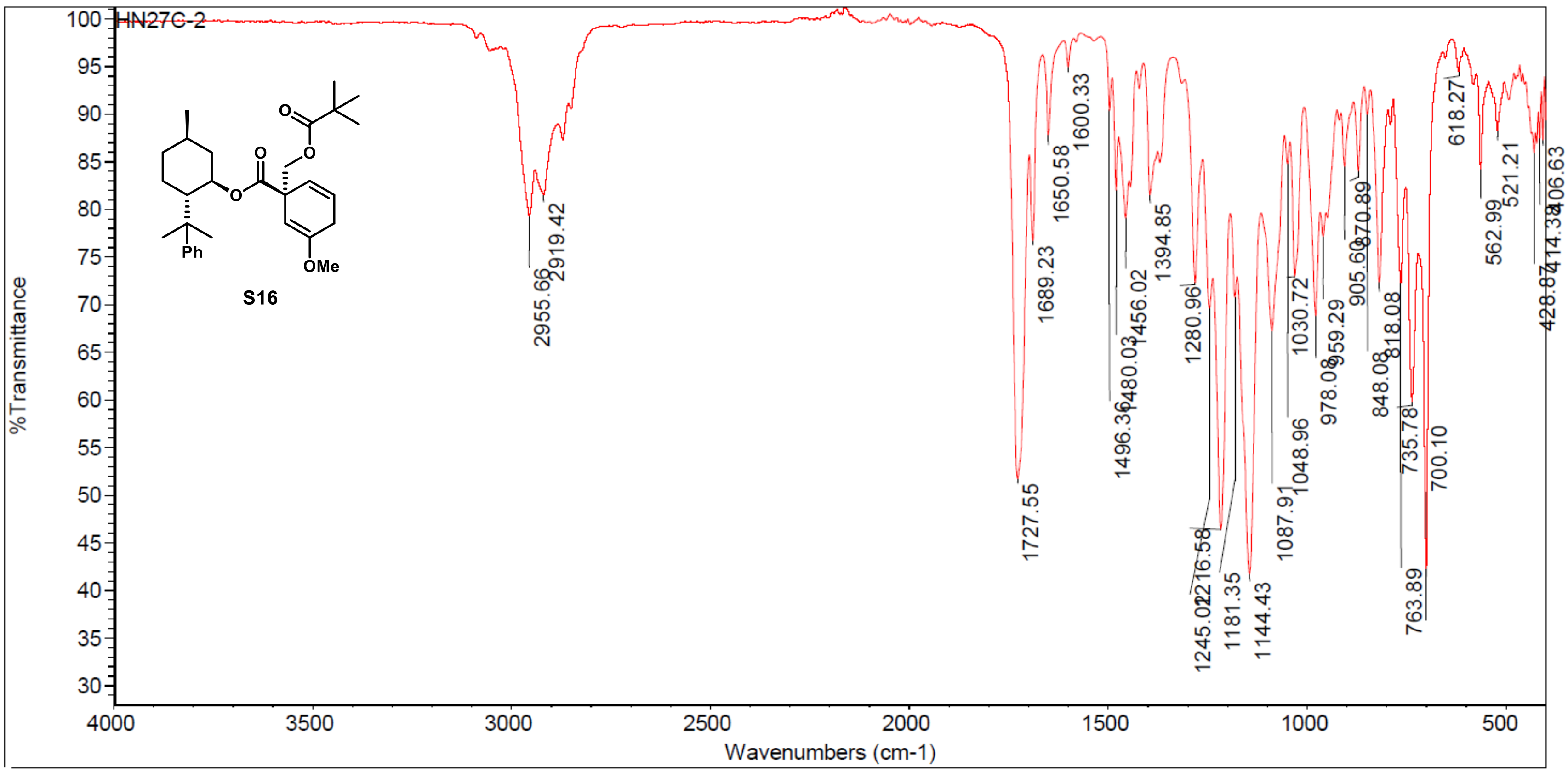
HN27C-2
S16
OMe
Ph
%Transmittance
Wavenumbers (cm-1)
2955.66
2919.42
1727.55
1689.23
1650.58
1600.33
1496.36
1480.03
1456.02
1394.85
1280.96
1245.02
1216.58
1181.35
1144.43
1087.91
1048.96
1030.72
978.08
959.29
905.60
870.89
848.08
818.08
763.89
735.78
700.10
618.27
562.99
521.21
428.87
414.38
406.63

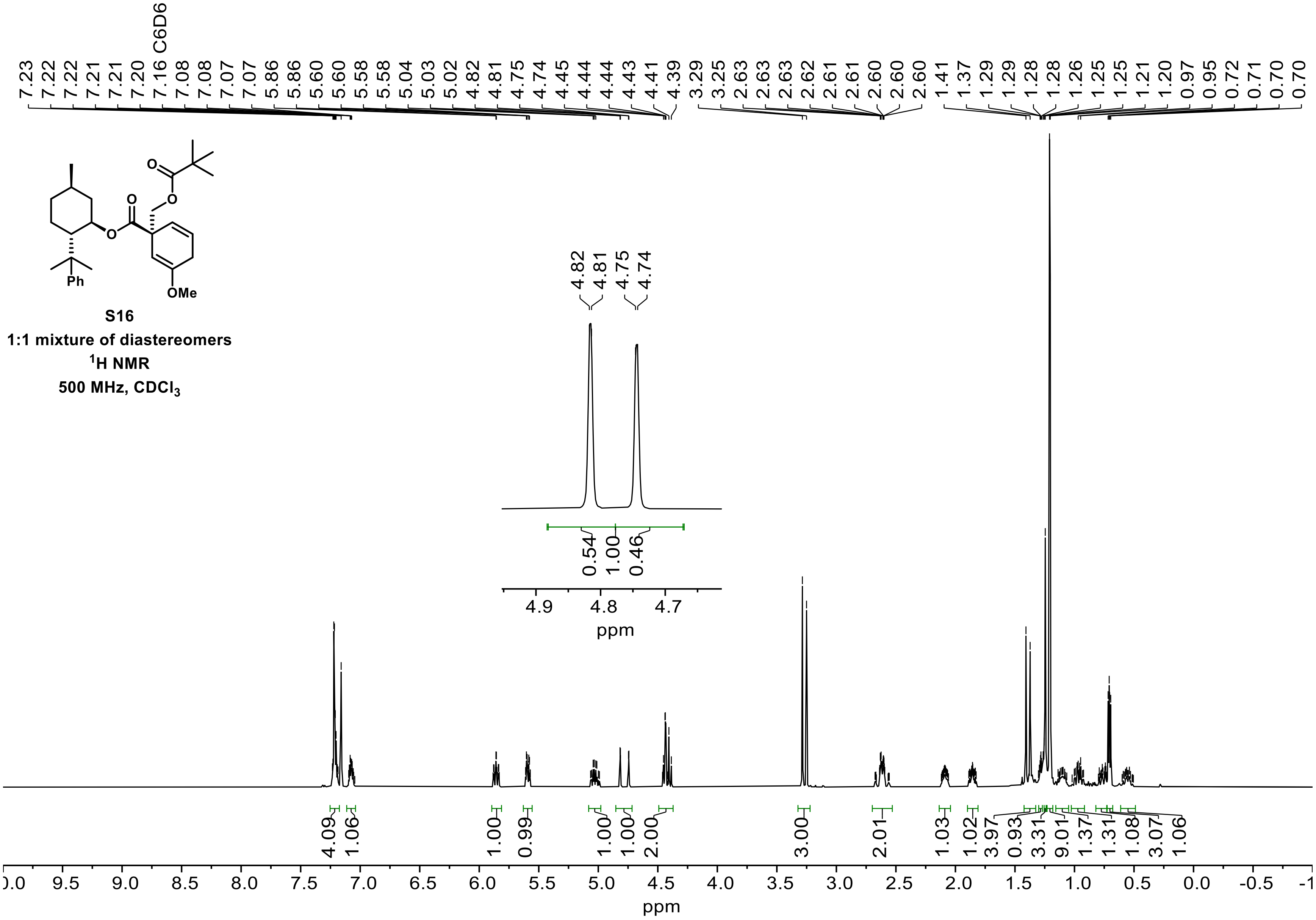
C6D6
7.23 7.22 7.22 7.21 7.21 7.20 7.16 7.08 7.08 7.08 7.07 7.07 5.86 5.86 5.60 5.60 5.58 5.58 5.04 5.03 5.02 4.82 4.81 4.75 4.74 4.45 4.44 4.44 4.43 4.41 4.39 3.29 3.25 2.63 2.63 2.63 2.62 2.61 2.61 2.60 2.60 2.60 1.41 1.37 1.29 1.29 1.28 1.28 1.26 1.25 1.25 1.21 1.20 0.97 0.95 0.72 0.71 0.70 0.70
Ph
OMe
S16
1:1 mixture of diastereomers
1H NMR
500 MHz, CDCl3
4.82 4.81 4.75 4.74
0.54 1.00 0.46
4.9 4.8 4.7
ppm
4.09 1.06 1.00 0.99 1.00 1.00 2.00 3.00 2.01 1.03 1.02 3.97 0.93 3.31 9.01 1.37 1.31 1.08 3.07 1.06
9.5 9.0 8.5 8.0 7.5 7.0 6.5 6.0 5.5 5.0 4.5 4.0 3.5 3.0 2.5 2.0 1.5 1.0 0.5 0.0 -0.5
ppm

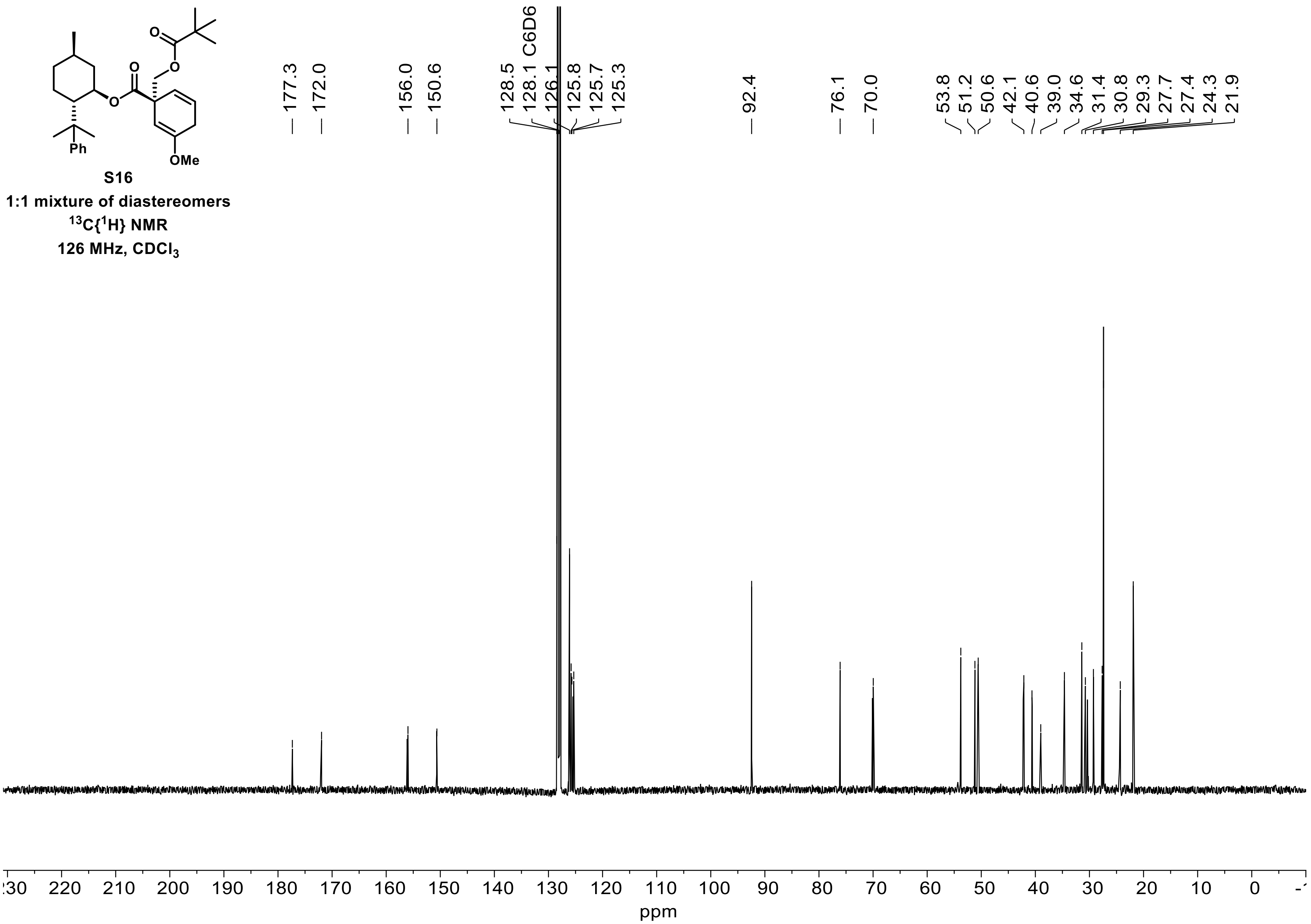
O
O
O
O
Ph
OMe
S16
1:1 mixture of diastereomers
$^{13}C\{^{1}H\}$ NMR
126 MHz, $CDCl_3$
177.3
172.0
156.0
150.6
128.5
128.1 C6D6
126.1
125.8
125.7
125.3
92.4
76.1
70.0
53.8
51.2
50.6
42.1
40.6
39.0
34.6
31.4
30.8
29.3
27.7
27.4
24.3
21.9
230 220 210 200 190 180 170 160 150 140 130 120 110 100 90 80 70 60 50 40 30 20 10 0
ppm

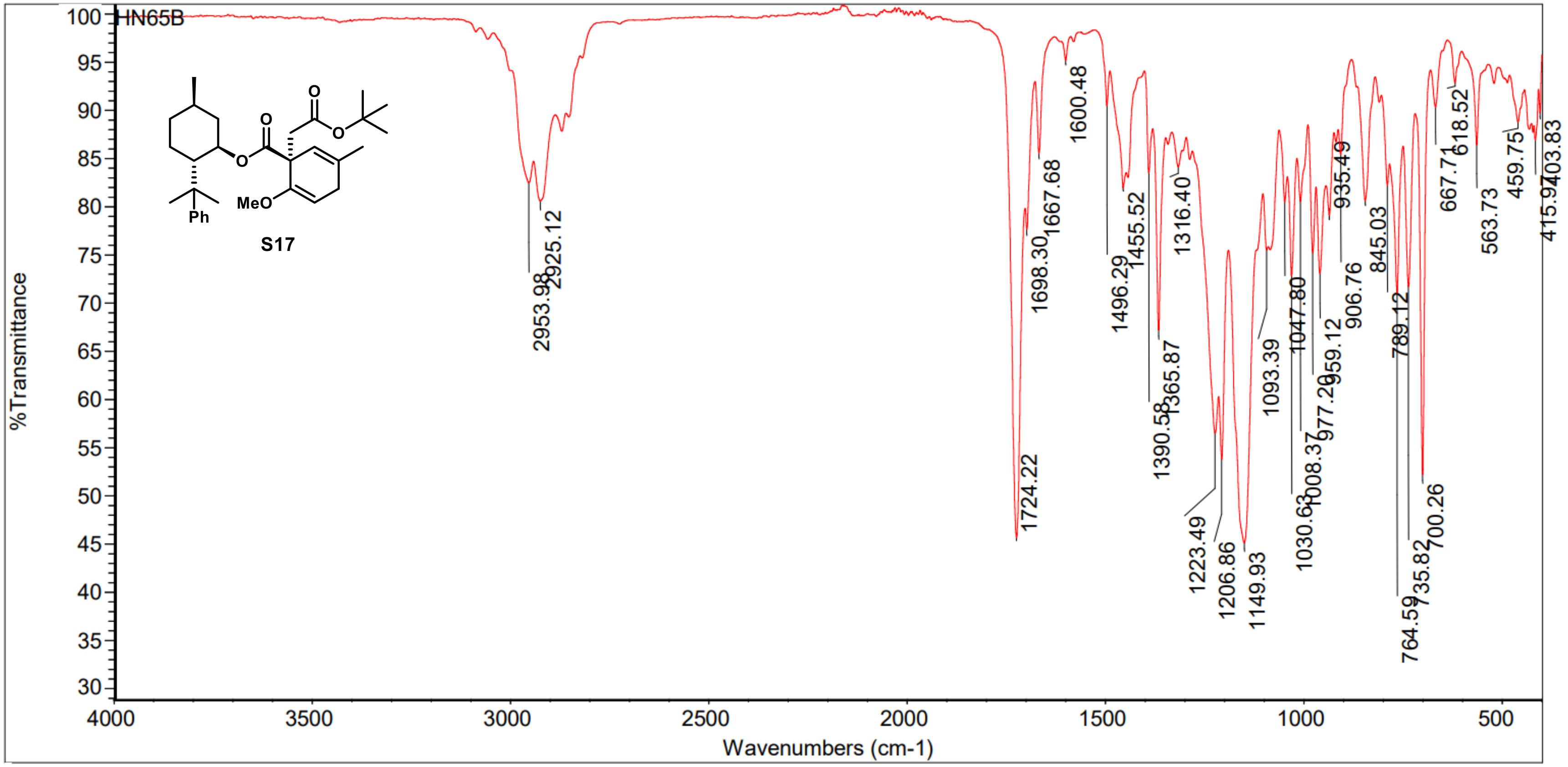
HN65B
S17
MeO
Ph
%Transmittance
Wavenumbers (cm-1)
2953.98
2925.12
1724.22
1698.30
1667.68
1600.48
1496.29
1455.52
1390.58
1365.87
1316.40
1223.49
1206.86
1149.93
1093.39
1047.80
1030.63
1008.37
977.29
959.12
935.49
906.76
845.03
789.12
764.59
735.82
700.26
667.71
618.52
563.73
459.75
415.97
403.83

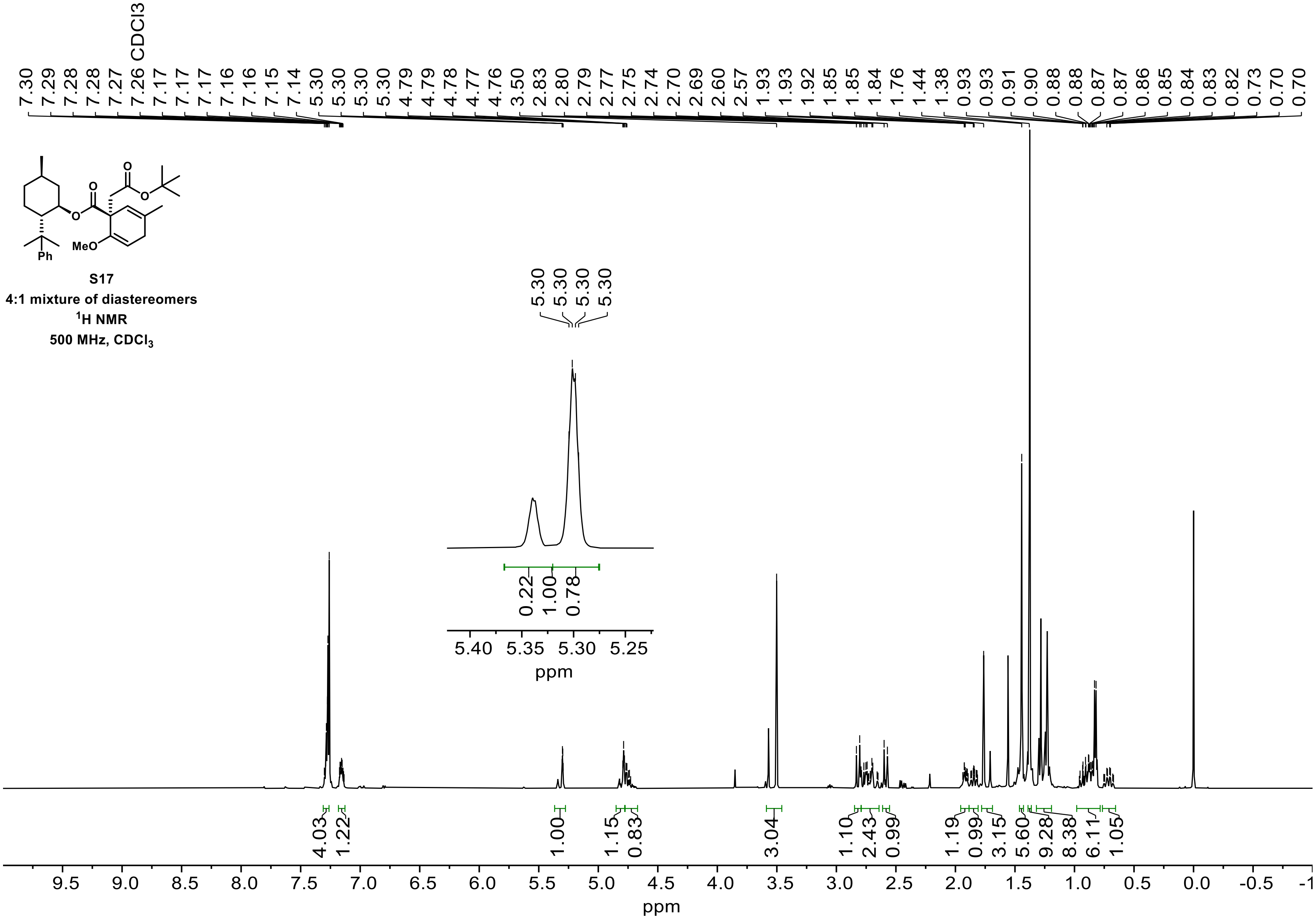

MeO
Ph
S17
4:1 mixture of diastereomers
^{1}H NMR
500 MHz, CDCl$_3$
CDCl3
7.30 7.29 7.28 7.28 7.27 7.26 7.17 7.17 7.17 7.16 7.16 7.15 7.14 5.30 5.30 5.30 5.30 4.79 4.79 4.78 4.77 4.76 3.50 2.83 2.80 2.79 2.77 2.75 2.74 2.70 2.69 2.60 2.57 1.93 1.93 1.92 1.85 1.85 1.84 1.76 1.44 1.38 0.93 0.93 0.91 0.90 0.88 0.88 0.87 0.87 0.86 0.85 0.84 0.83 0.82 0.73 0.70 0.70
5.30 5.30 5.30 5.30
0.22 1.00 0.78
5.40 5.35 5.30 5.25
ppm
4.03 1.22 1.00 1.15 0.83 3.04 1.10 2.43 0.99 1.19 0.99 3.15 5.60 9.28 8.38 6.11 1.05
9.5 9.0 8.5 8.0 7.5 7.0 6.5 6.0 5.5 5.0 4.5 4.0 3.5 3.0 2.5 2.0 1.5 1.0 0.5 0.0 -0.5 -1
ppm

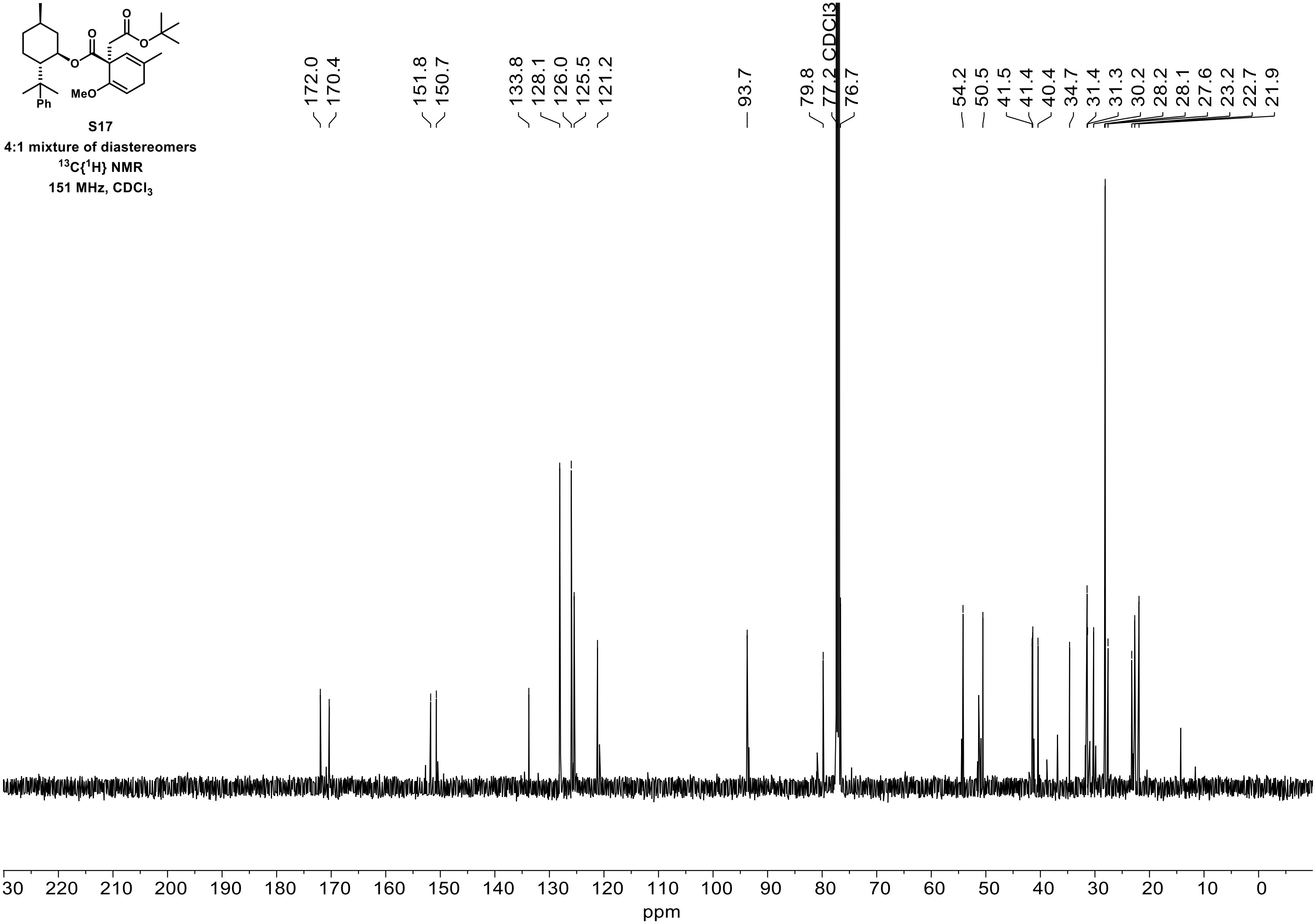
MeO
Ph
O
S17
4:1 mixture of diastereomers
$^{13}C\{^1H\}$ NMR
151 MHz, $CDCl_3$
172.0
170.4
151.8
150.7
133.8
128.1
126.0
125.5
121.2
93.7
79.8
77.2 CDCl3
76.7
54.2
50.5
41.5
41.4
40.4
34.7
31.4
31.3
30.2
28.2
28.1
27.6
23.2
22.7
21.9
30
220
210
200
190
180
170
160
150
140
130
120
110
100
90
80
70
60
50
40
30
20
10
0
ppm

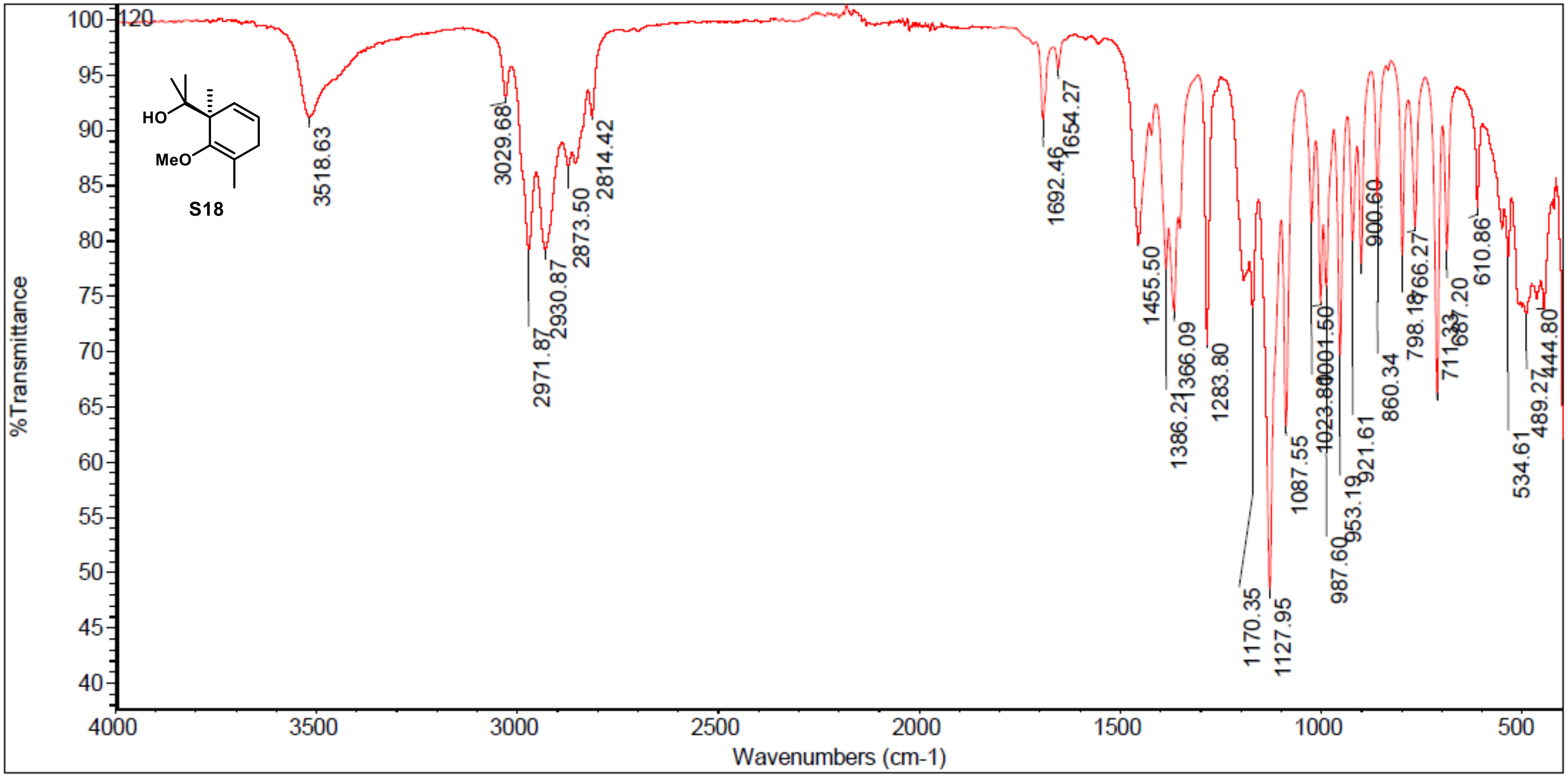

%Transmittance
Wavenumbers (cm-1)
HO
MeO
S18
3518.63
3029.68
2971.87
2930.87
2873.50
2814.42
1692.46
1654.27
1455.50
1386.21
1366.09
1283.80
1170.35
1127.95
1087.55
1023.80
1001.50
987.60
953.19
921.61
900.60
860.34
798.18
766.27
711.33
687.20
610.86
534.61
489.27
444.80

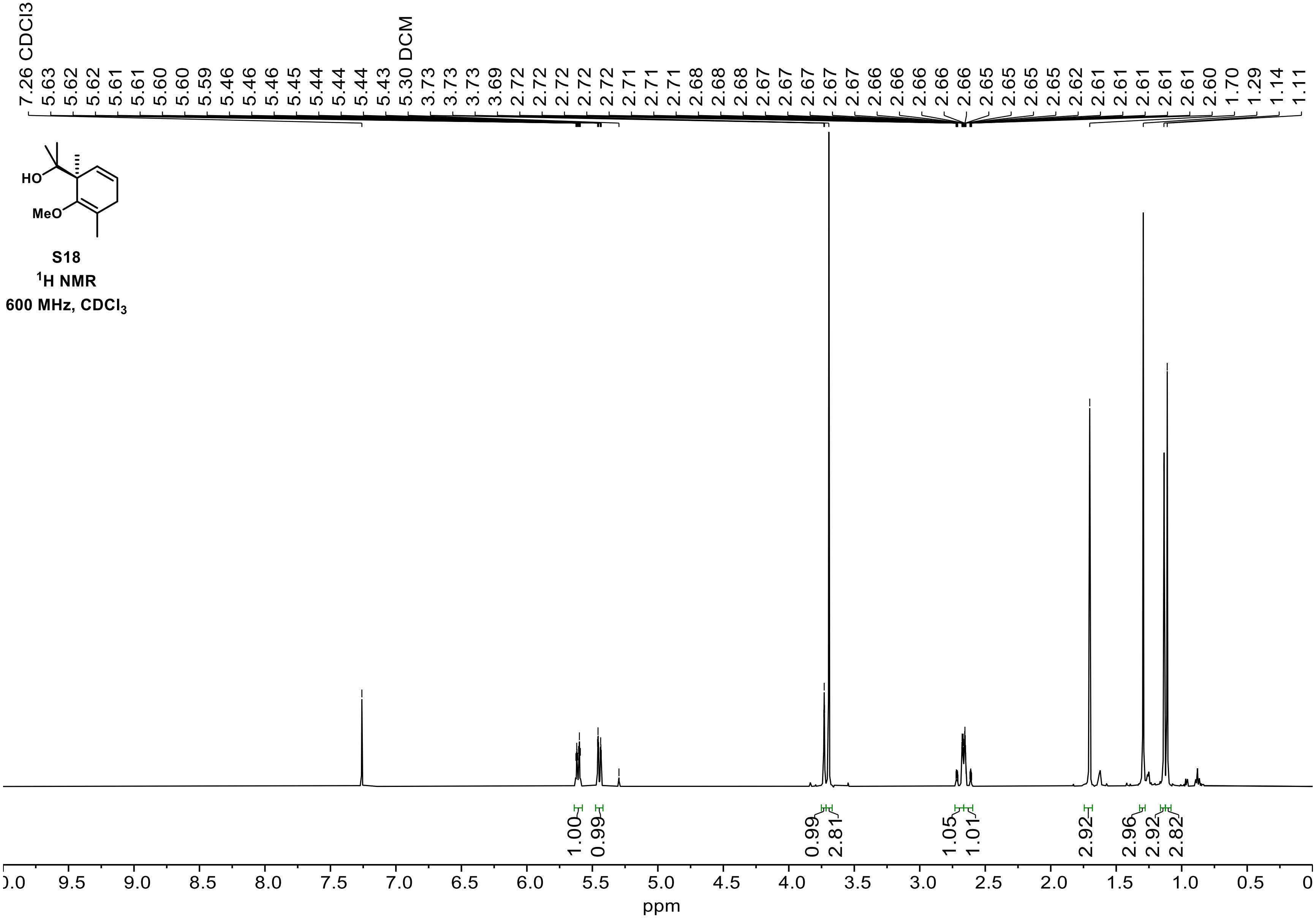
HO
MeO
S18
^{1}H NMR
600 MHz, $CDCl_3$
7.26 CDCl3
5.30 DCM
ppm

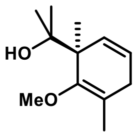

S18
$^{13}C\{^{1}H\}$ NMR
151 MHz, $CDCl_3$

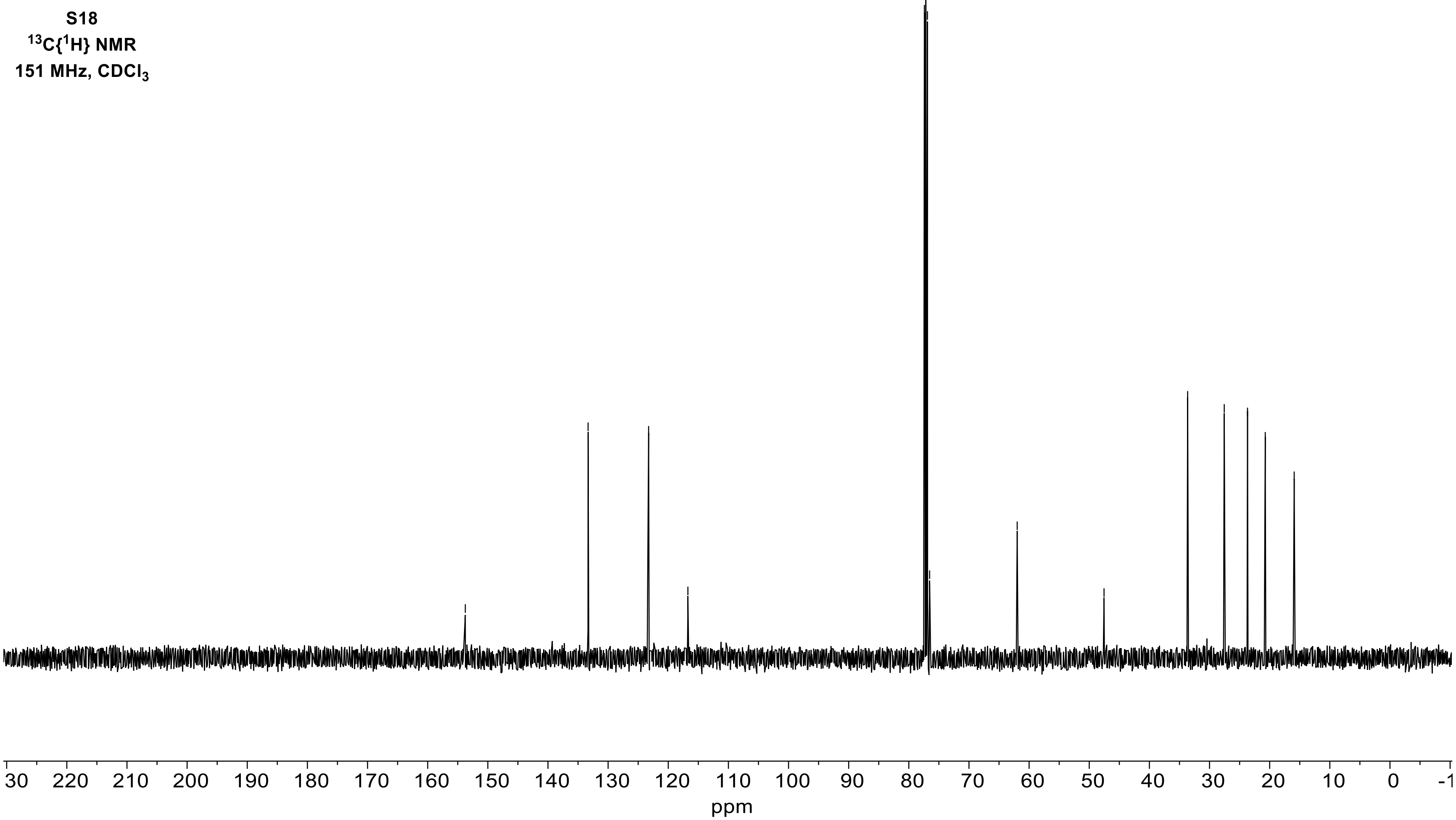

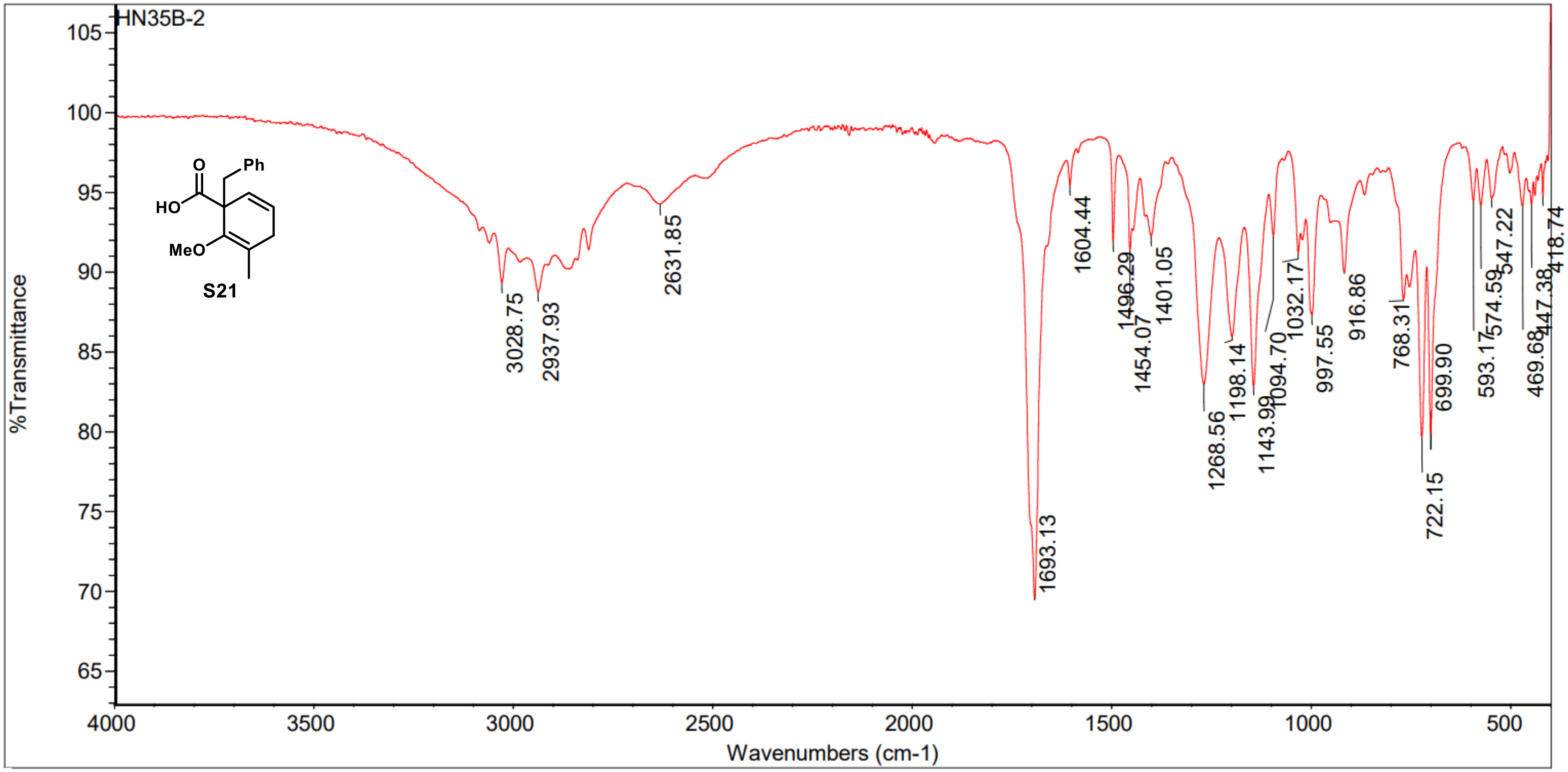
HN35B-2
%Transmittance
Wavenumbers (cm-1)
105
100
95
90
85
80
75
70
65
4000
3500
3000
2500
2000
1500
1000
500
3028.75
2937.93
2631.85
1693.13
1604.44
1496.29
1454.07
1401.05
1268.56
1198.14
1143.99
1094.70
1032.17
997.55
916.86
768.31
722.15
699.90
593.17
574.59
547.22
469.68
447.38
418.74
O
Ph
HO
MeO
S21

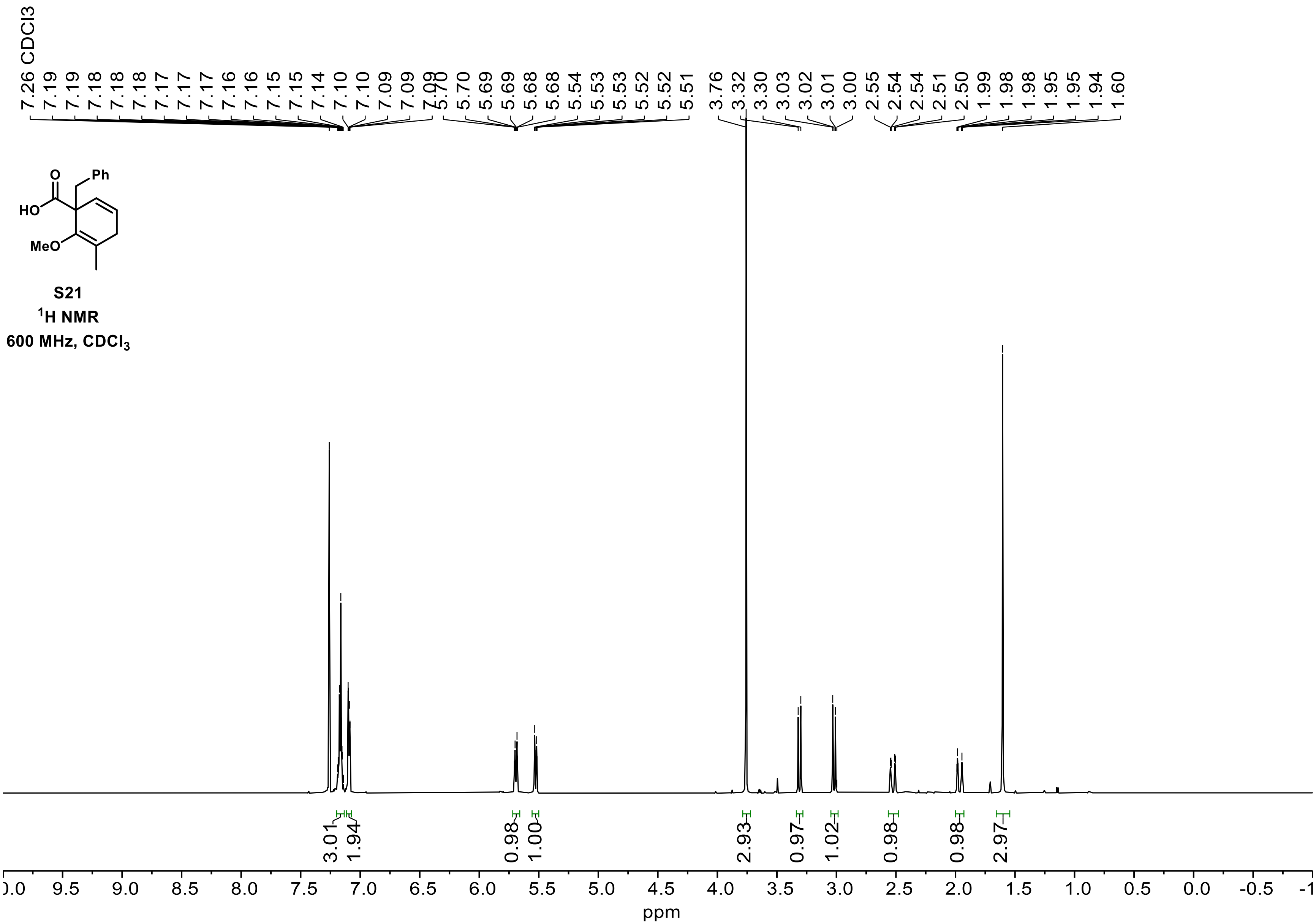

7.26 CDCl3
7.19
7.19
7.18
7.18
7.18
7.17
7.17
7.17
7.16
7.16
7.15
7.15
7.14
7.10
7.10
7.09
7.09
7.09
5.70
5.70
5.69
5.69
5.68
5.68
5.54
5.53
5.53
5.52
5.52
5.51
3.76
3.32
3.30
3.03
3.02
3.01
3.00
2.55
2.54
2.54
2.51
2.50
1.99
1.98
1.98
1.95
1.95
1.94
1.60
Ph
O
HO
MeO
S21
^{1}H NMR
600 MHz, CDCl$_3$
3.01
1.94
0.98
1.00
2.93
0.97
1.02
0.98
0.98
2.97
ppm

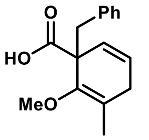

S21
$^{13}C\{^1H\}$ NMR
151 MHz, $CDCl_3$

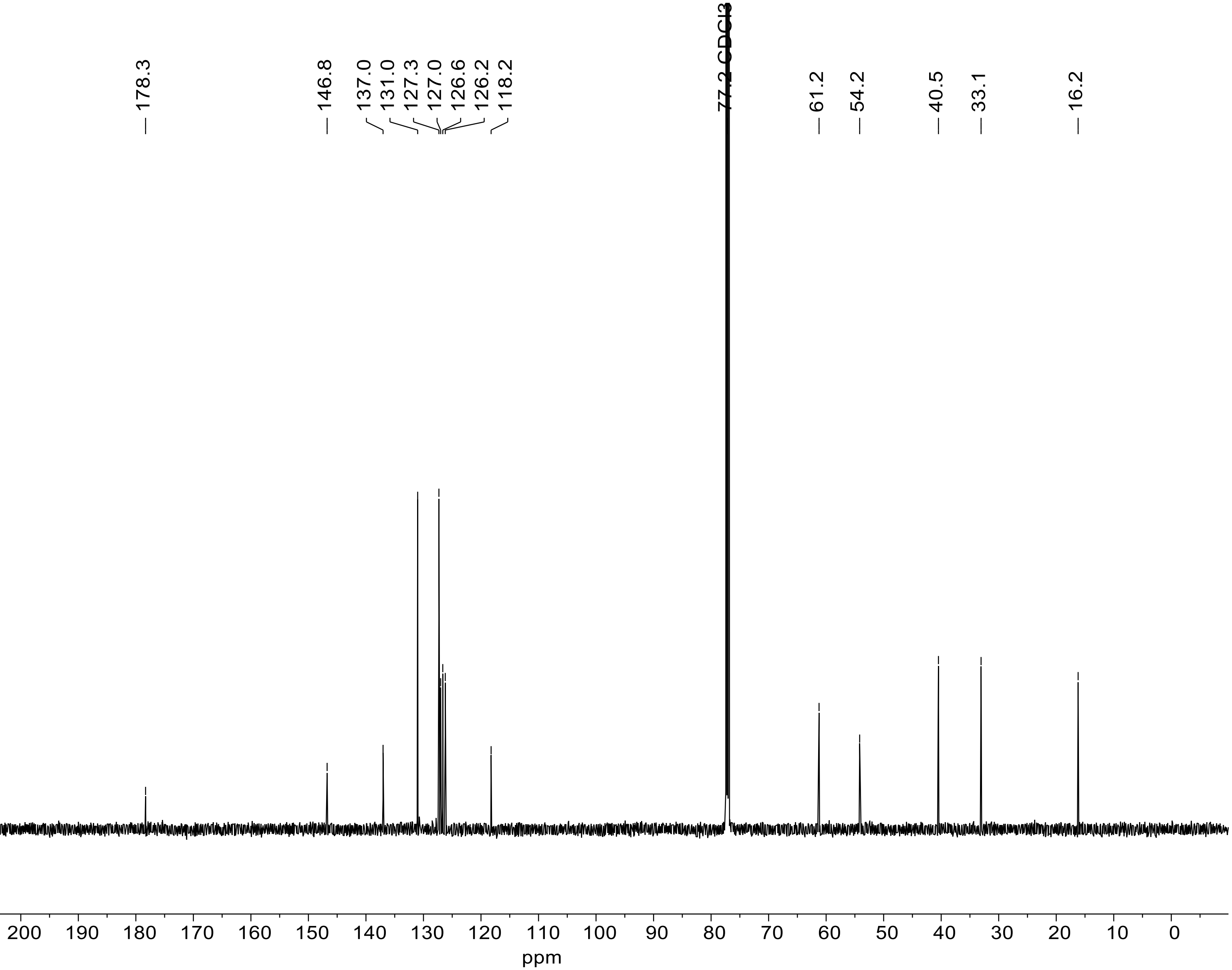

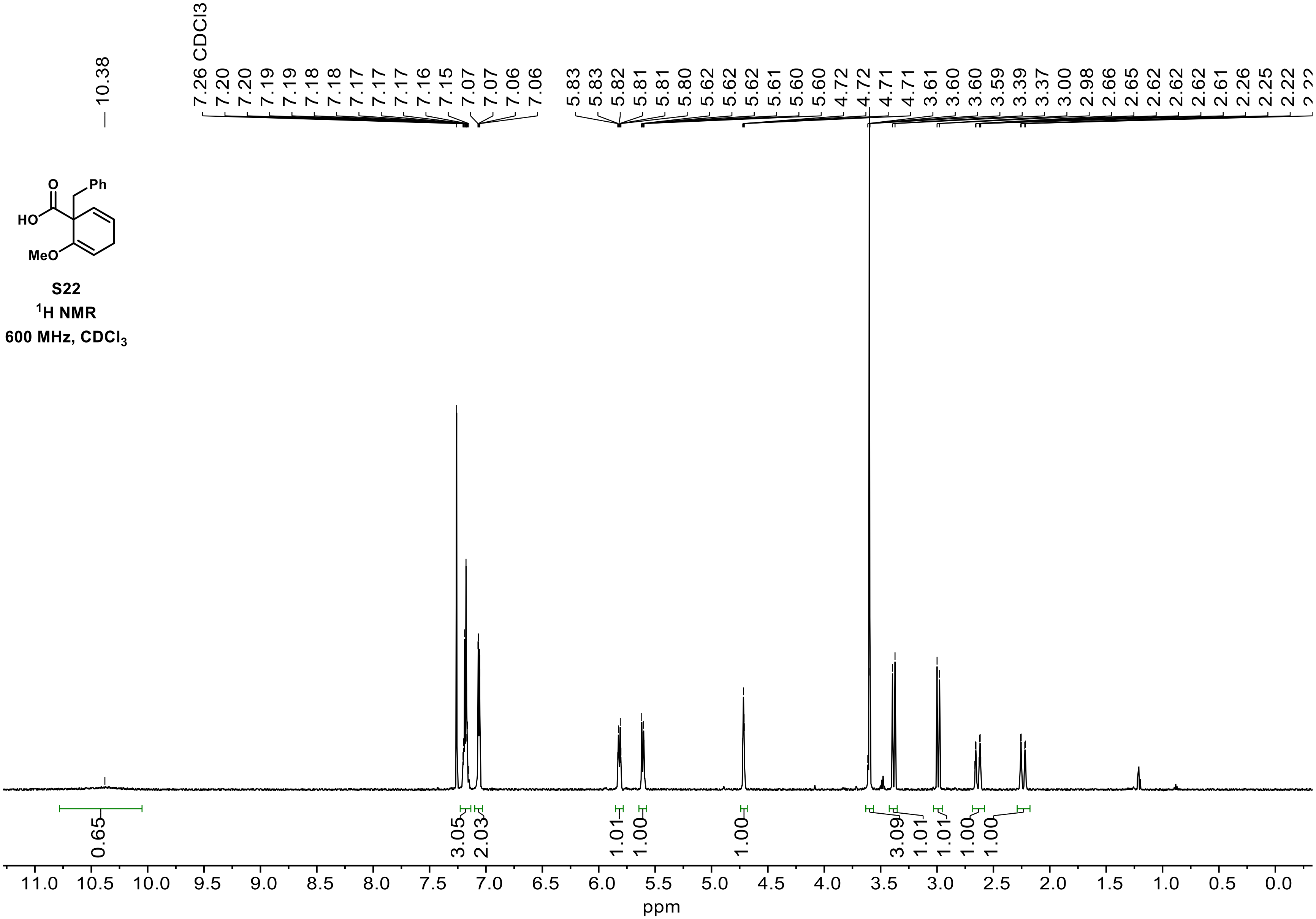

S22
^{1}H NMR
600 MHz, $CDCl_3$

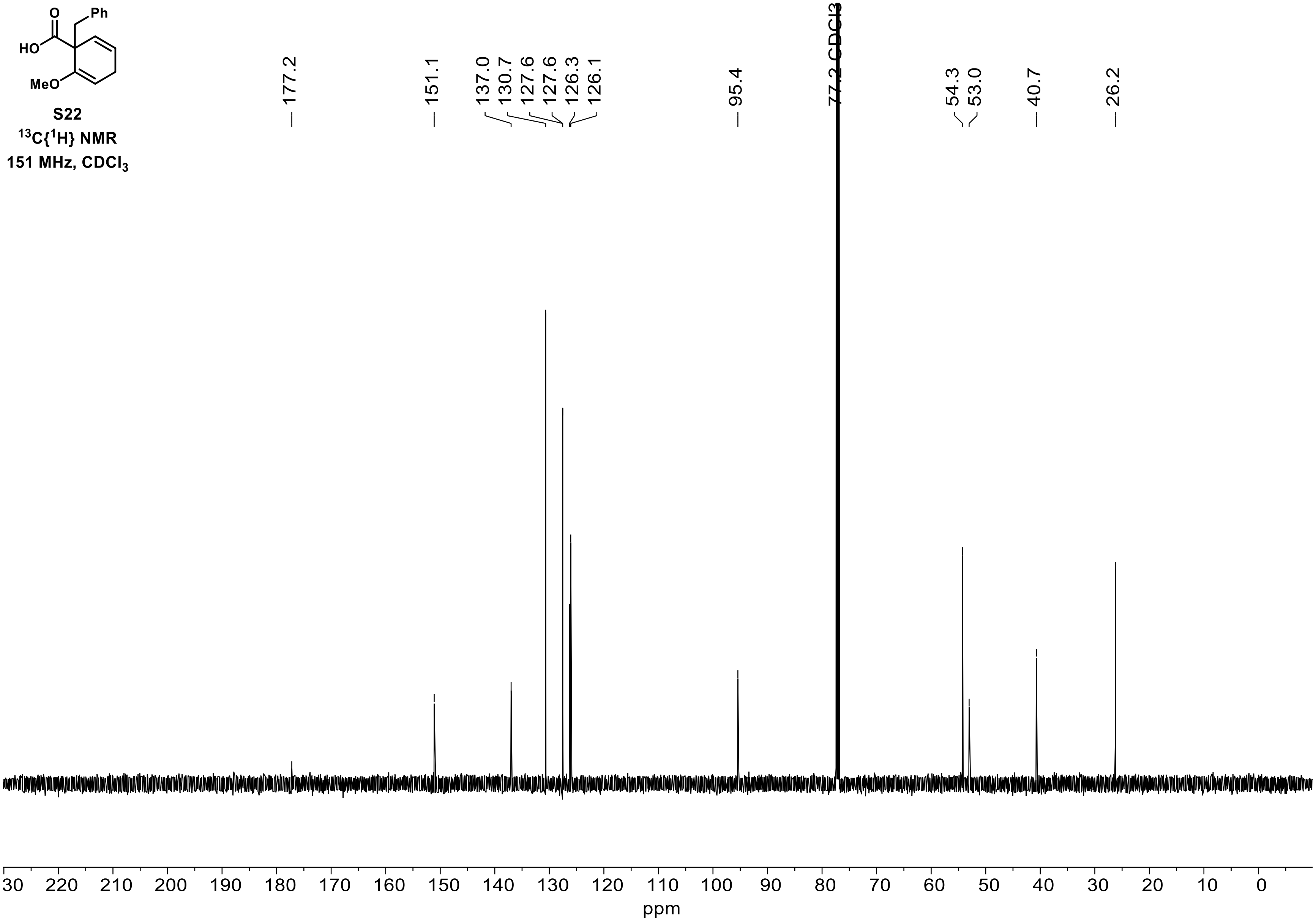

Ph
HO
O
MeO
S22
$^{13}C\{^1H\}$ NMR
151 MHz, $CDCl_3$
177.2
151.1
137.0
130.7
127.6
127.6
126.3
126.1
95.4
77.2 CDCl3
54.3
53.0
40.7
26.2
230 220 210 200 190 180 170 160 150 140 130 120 110 100 90 80 70 60 50 40 30 20 10 0
ppm

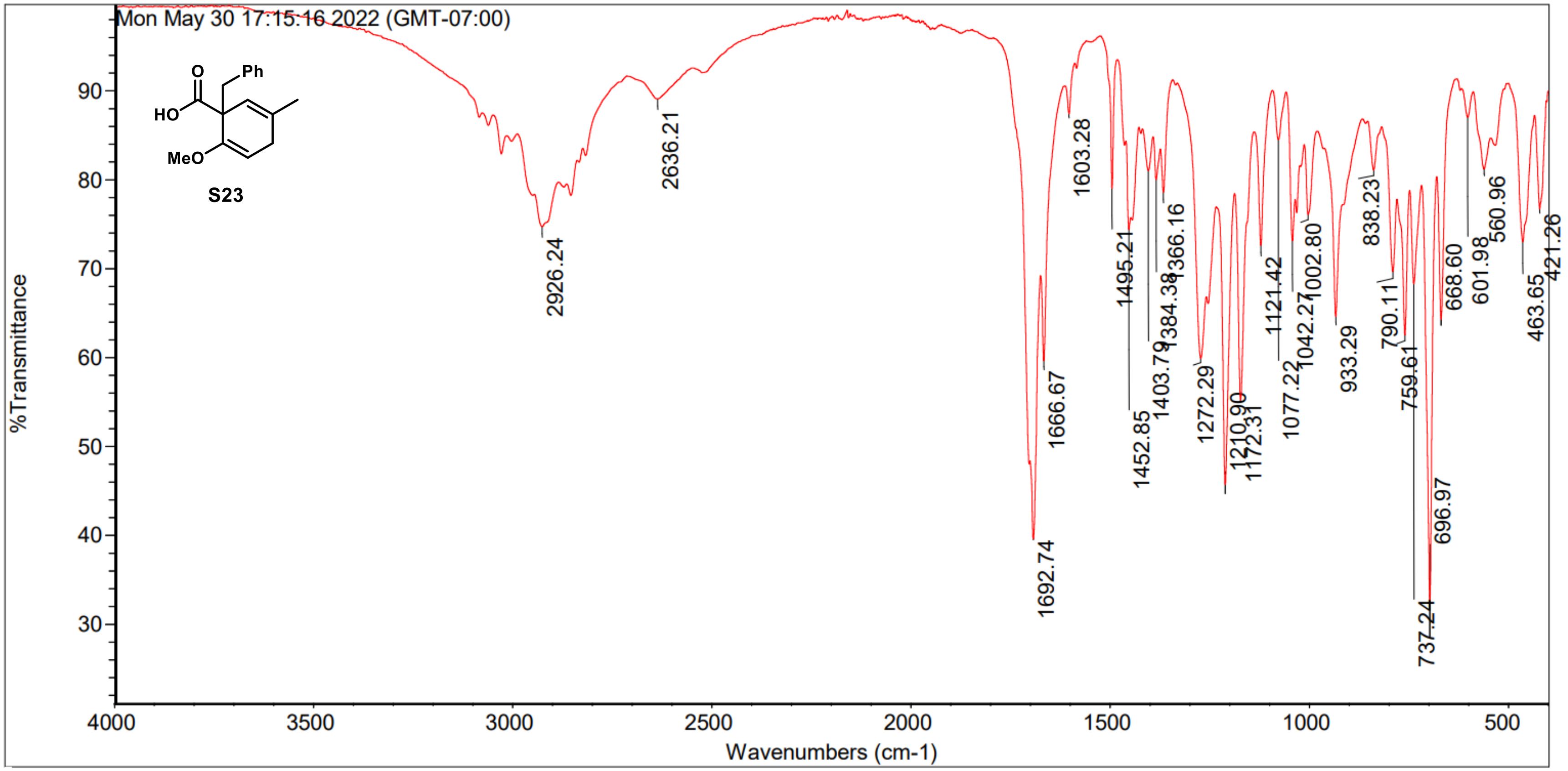
Mon May 30 17:15:16 2022 (GMT-07:00)
O
HO
Ph
MeO
S23
%Transmittance
Wavenumbers (cm-1)
2926.24
2636.21
1692.74
1666.67
1603.28
1495.21
1452.85
1403.79
1384.38
1366.16
1272.29
1210.90
1172.31
1121.42
1077.22
1042.27
1002.80
933.29
838.23
790.11
759.61
737.24
696.97
668.60
601.98
560.96
463.65
421.26

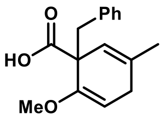

S23
^{1}H NMR
500 MHz, $CDCl_3$

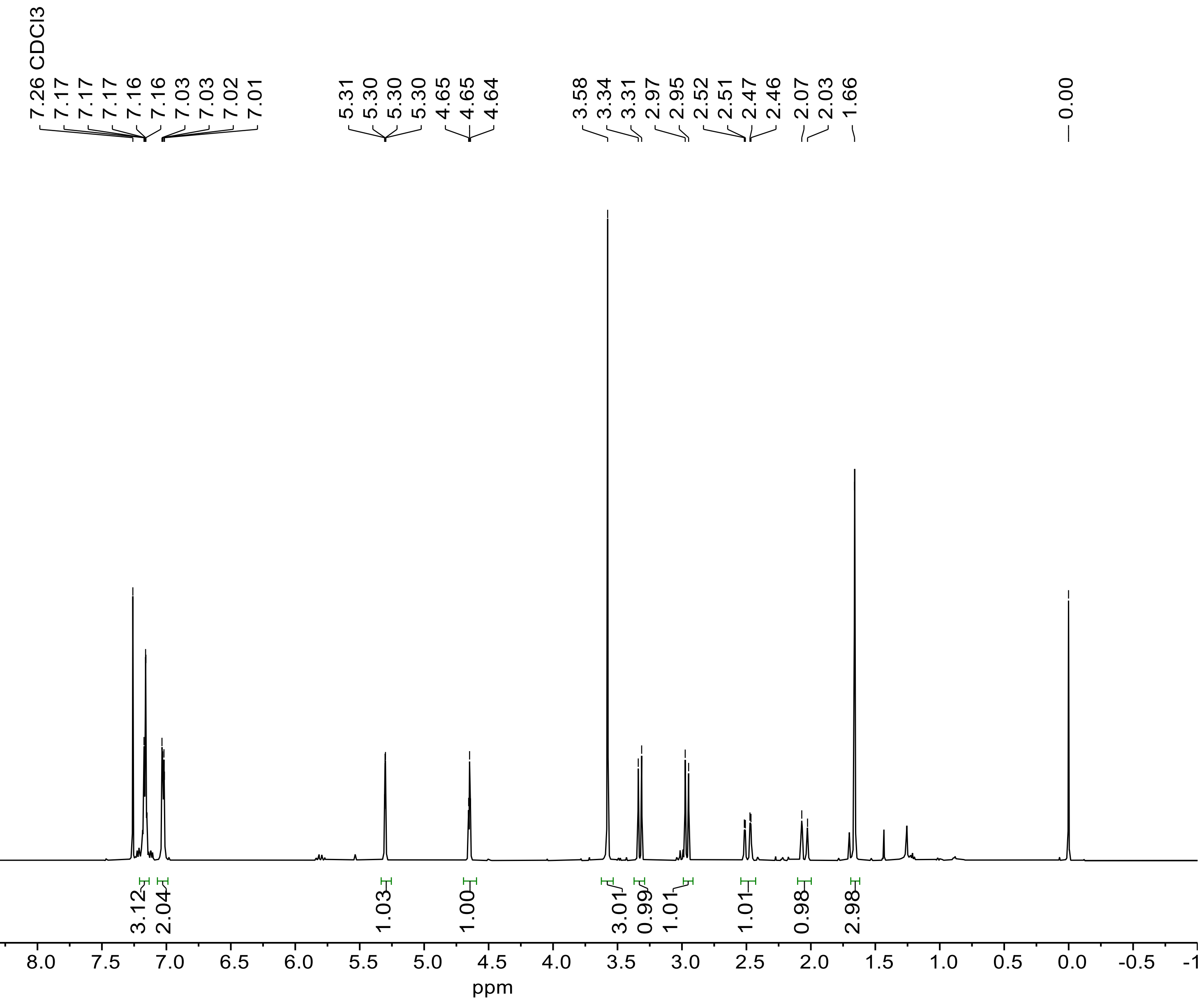

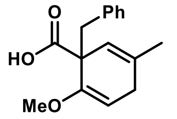

S23
$^{13}C\{^1H\}$ NMR
151 MHz, $CDCl_3$

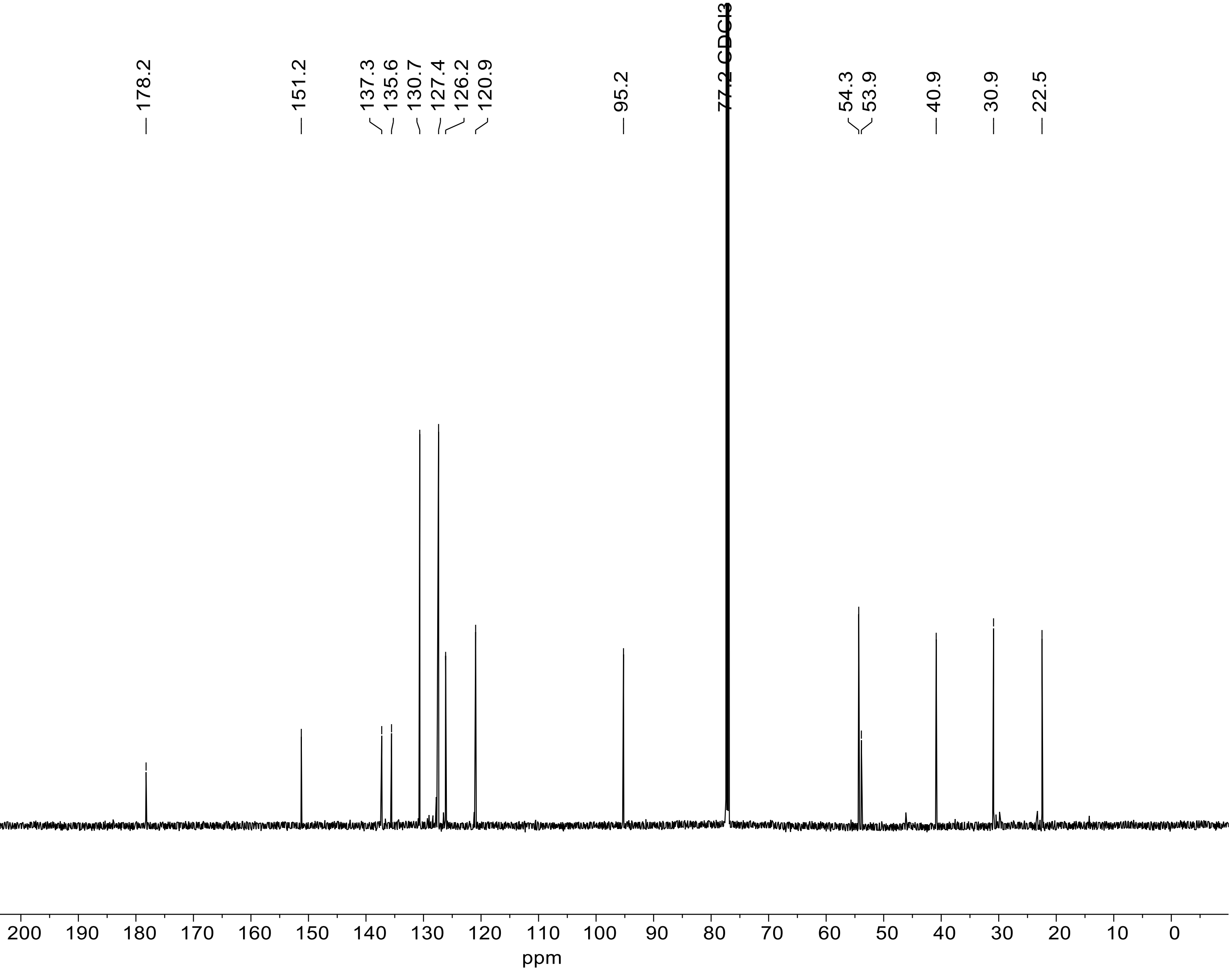

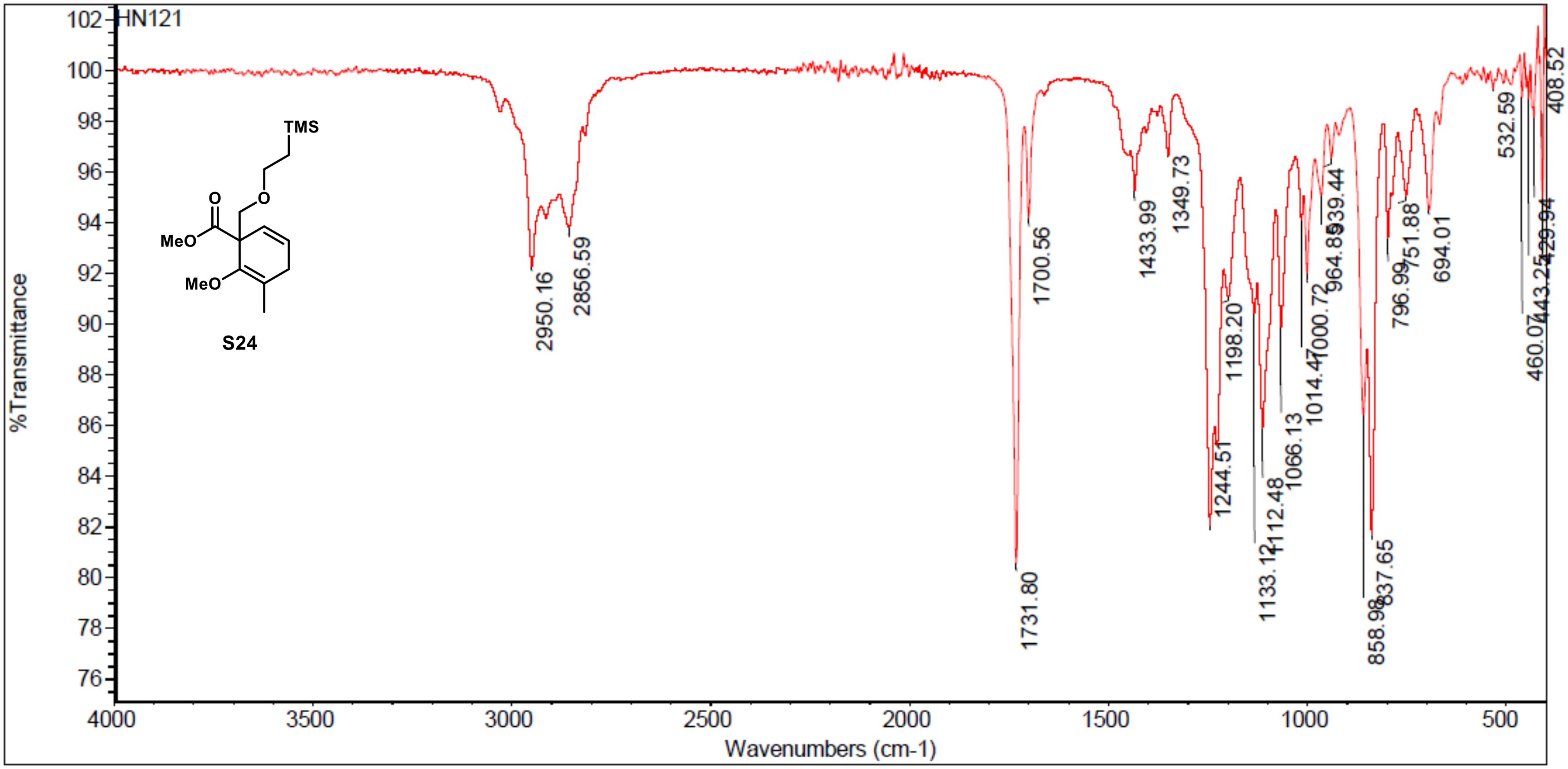

HN121
TMS
O
O
MeO
MeO
S24
%Transmittance
Wavenumbers (cm-1)
2950.16
2856.59
1731.80
1700.56
1433.99
1349.73
1244.51
1198.20
1133.12
1112.48
1066.13
1014.47
1000.72
964.85
939.44
858.98
837.65
796.99
751.88
694.01
532.59
460.07
443.25
429.94
408.52

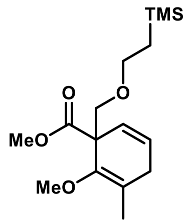

S24
^{1}H NMR
500 MHz, $CDCl_3$

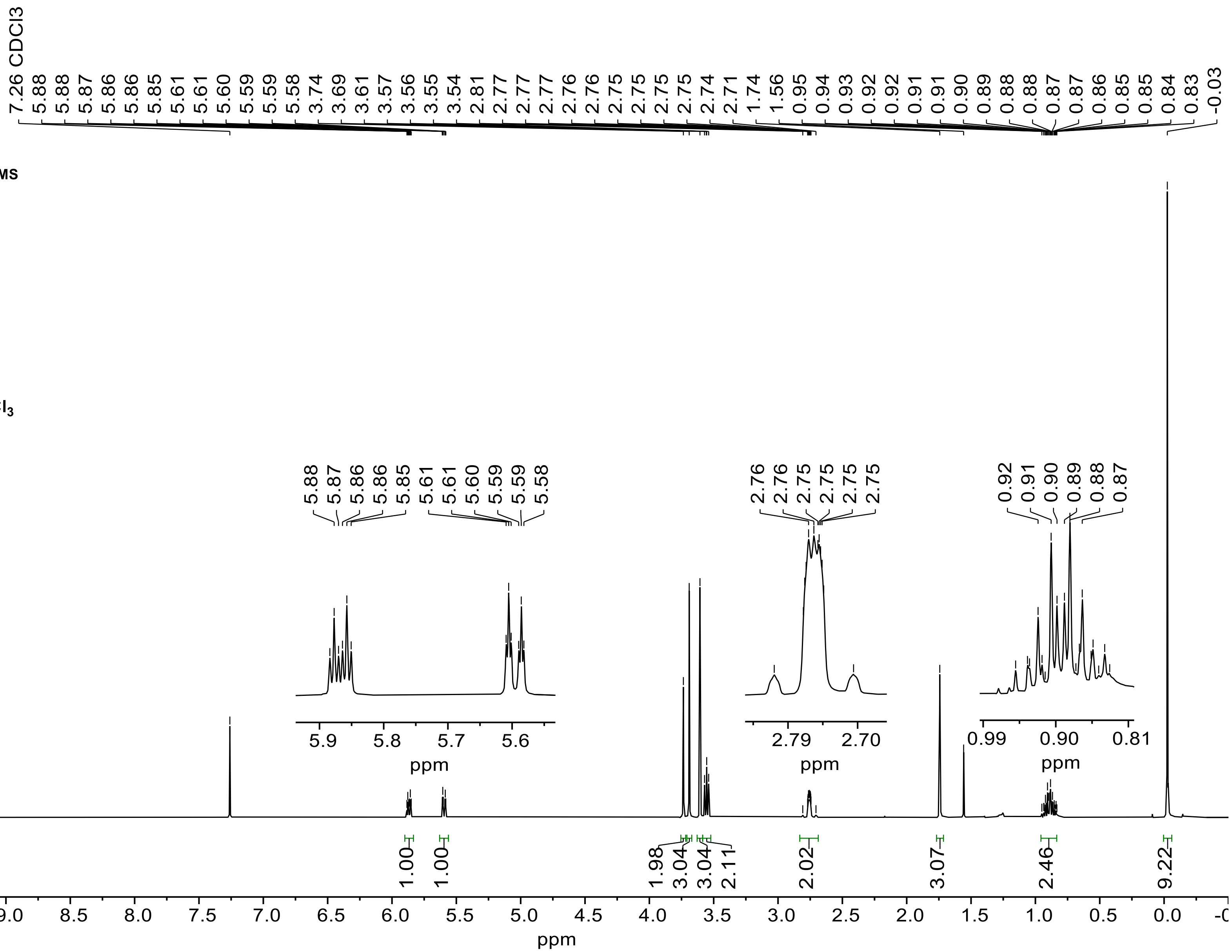

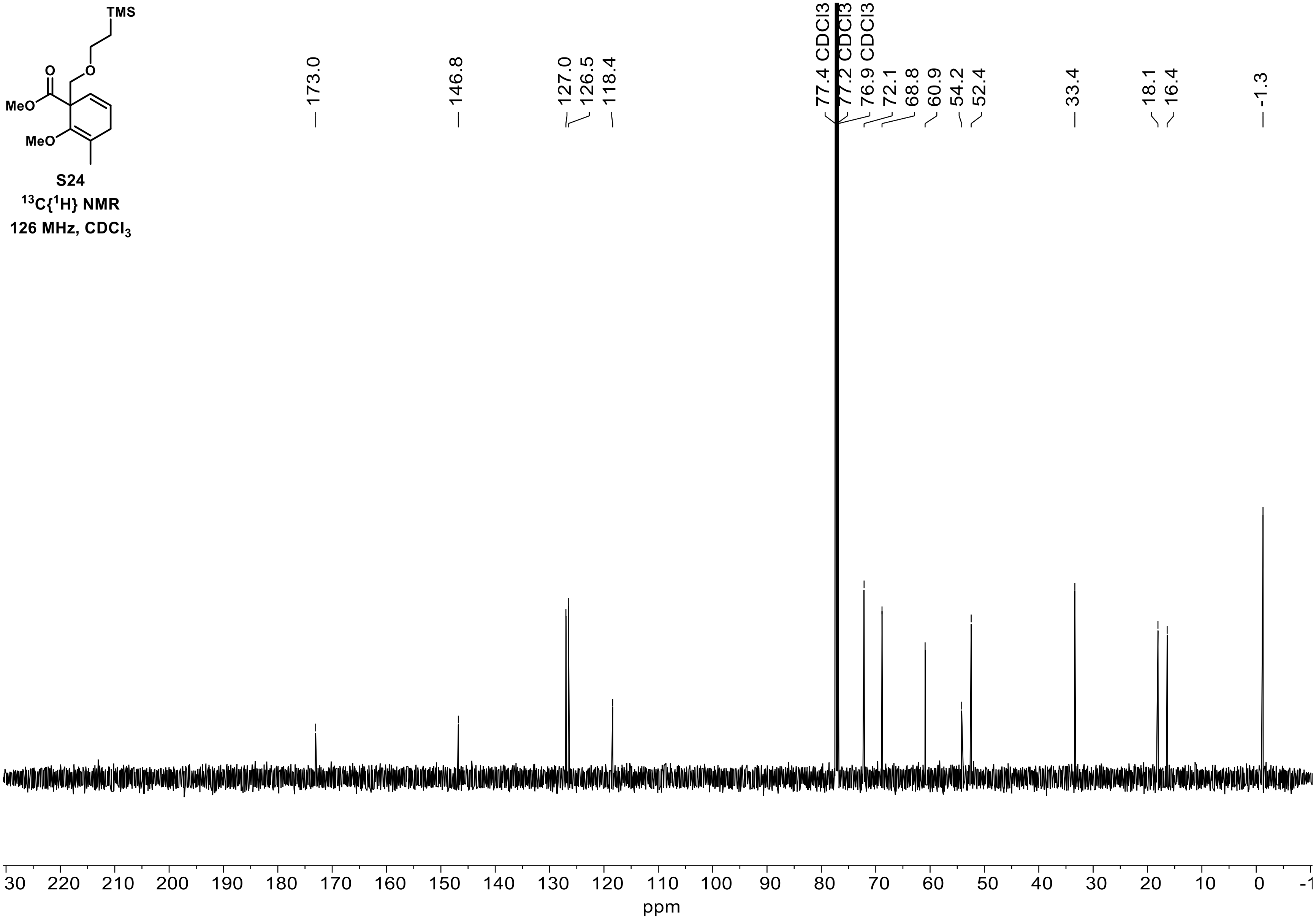
TMS
O
O
MeO
MeO
S24
13C{1H} NMR
126 MHz, CDCl3
173.0
146.8
127.0
126.5
118.4
77.4 CDCl3
77.2 CDCl3
76.9 CDCl3
72.1
68.8
60.9
54.2
52.4
33.4
18.1
16.4
-1.3
30 220 210 200 190 180 170 160 150 140 130 120 110 100 90 80 70 60 50 40 30 20 10 0 -1
ppm

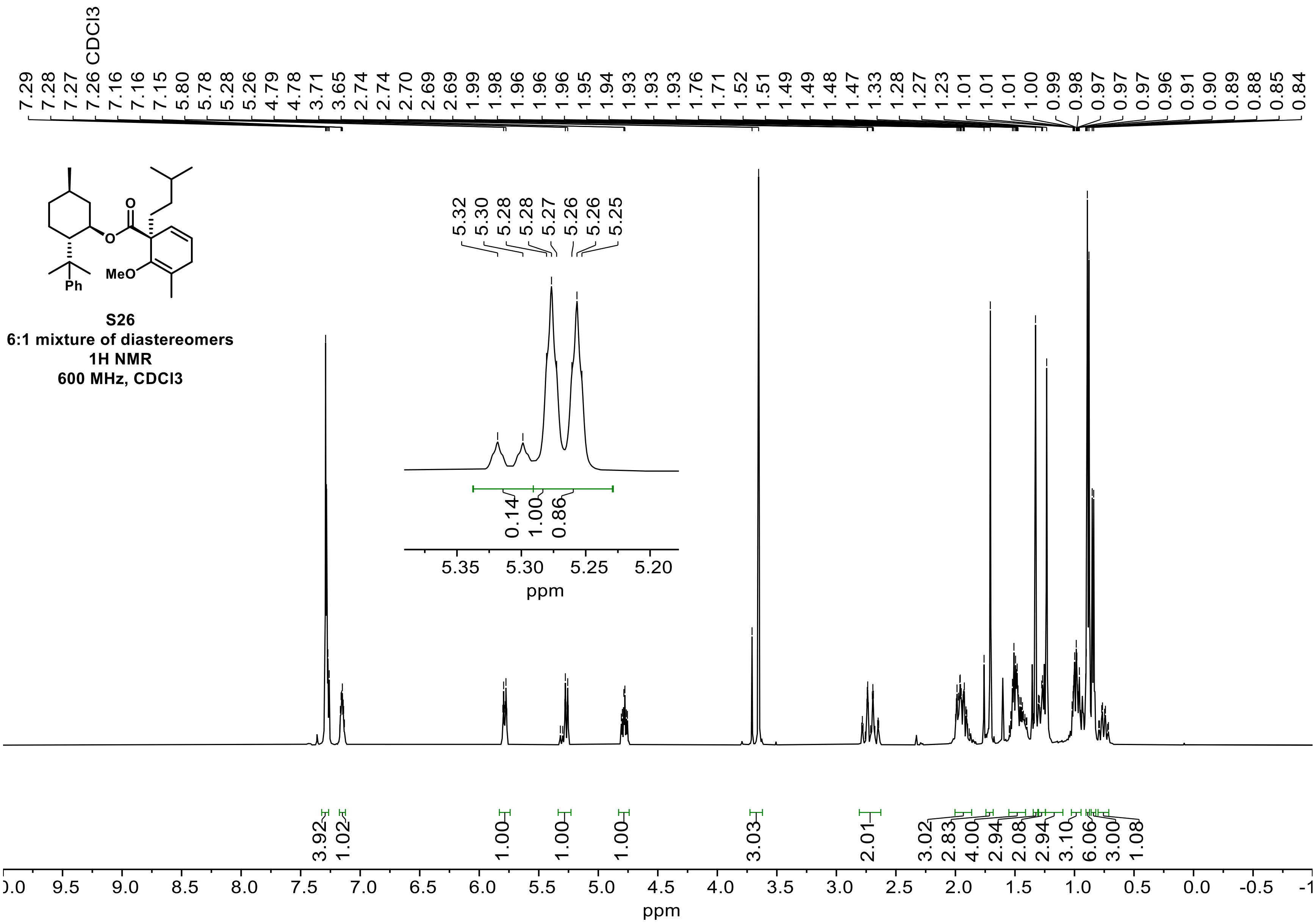
7.29 7.28 7.27 7.26 CDCl3 7.16 7.16 7.15 5.80 5.78 5.28 5.26 4.79 4.78 3.71 3.65 2.74 2.74 2.70 2.69 2.69 1.99 1.98 1.96 1.96 1.96 1.95 1.94 1.93 1.93 1.93 1.76 1.71 1.52 1.51 1.49 1.49 1.48 1.47 1.33 1.28 1.27 1.23 1.01 1.01 1.01 1.00 0.99 0.98 0.97 0.97 0.97 0.96 0.91 0.90 0.89 0.88 0.85 0.84
O
O
MeO
Ph
S26
6:1 mixture of diastereomers
1H NMR
600 MHz, CDCl3
5.32 5.30 5.28 5.28 5.27 5.26 5.26 5.25
0.14 1.00 0.86
5.35 5.30 5.25 5.20
ppm
3.92 1.02 1.00 1.00 1.00 3.03 2.01 3.02 2.83 4.00 2.94 2.08 2.94 3.10 6.06 3.00 1.08
10.0 9.5 9.0 8.5 8.0 7.5 7.0 6.5 6.0 5.5 5.0 4.5 4.0 3.5 3.0 2.5 2.0 1.5 1.0 0.5 0.0 -0.5 -1
ppm

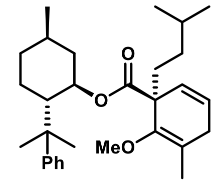

S26
6:1 mixture of diastereomers
$^{13}C\{^1H\}$ NMR
151 MHz, CDCl3

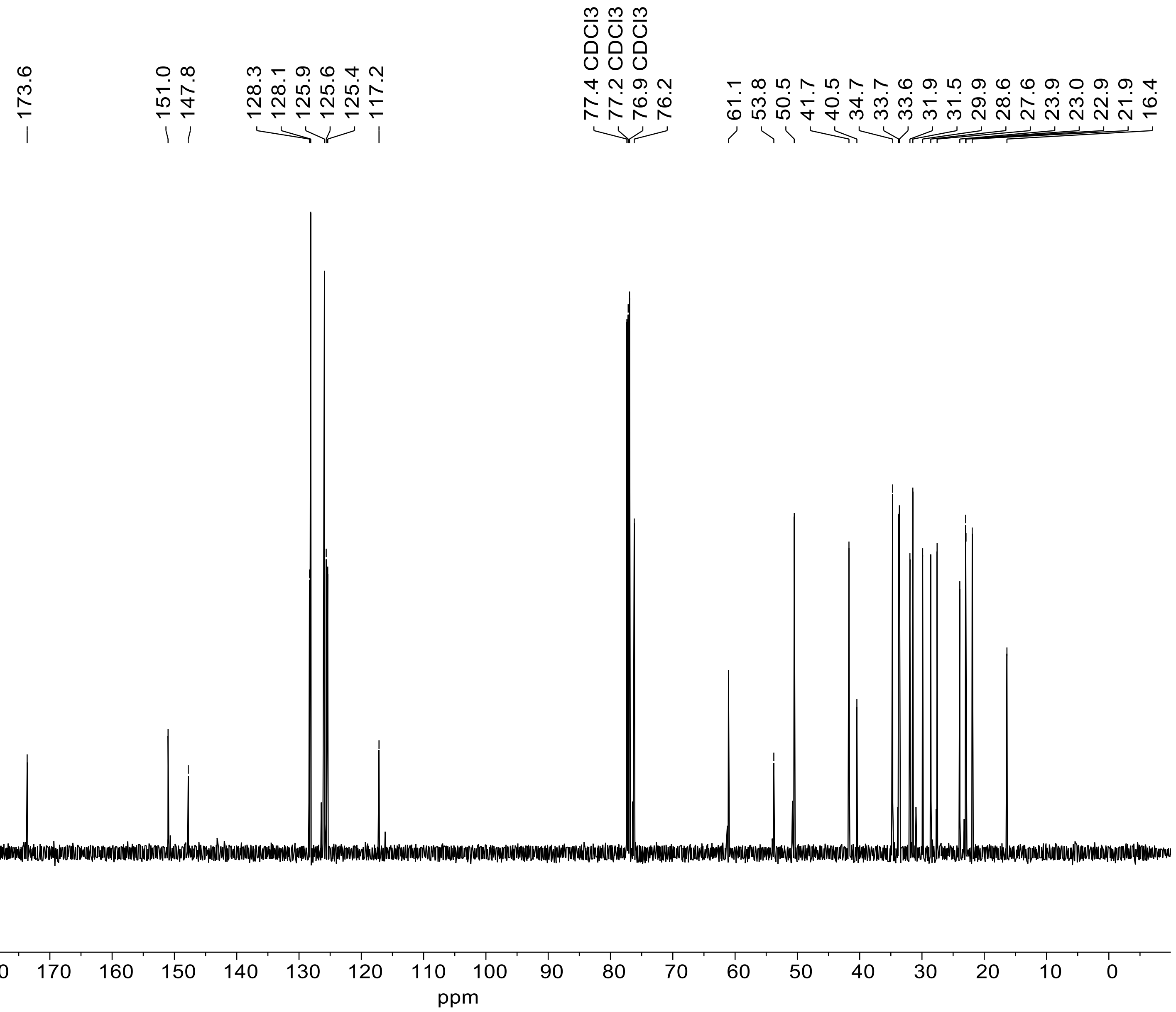

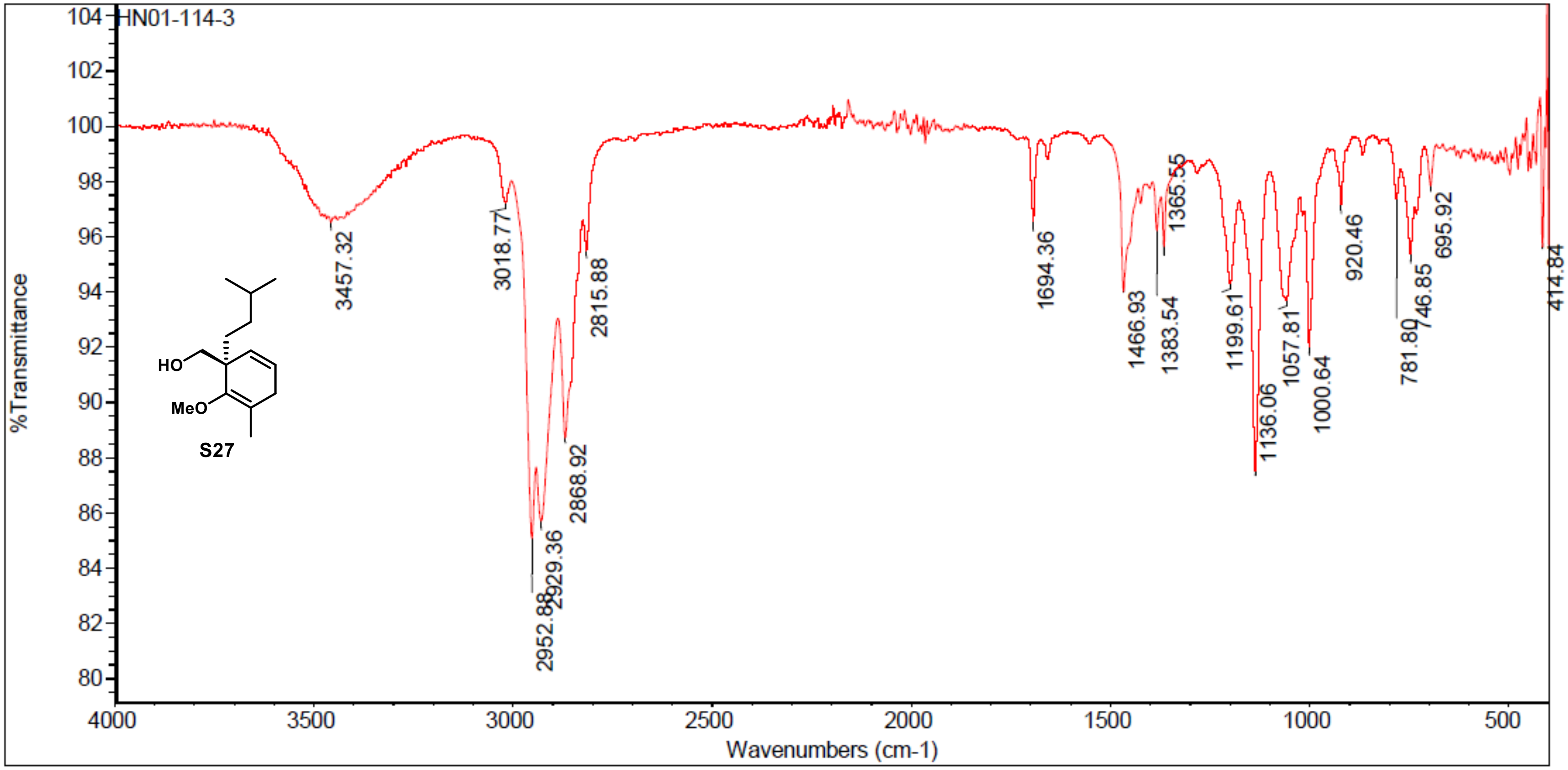

HN01-114-3
%Transmittance
Wavenumbers (cm-1)
104
102
100
98
96
94
92
90
88
86
84
82
80
4000
3500
3000
2500
2000
1500
1000
500
3457.32
3018.77
2952.88
2929.36
2868.92
2815.88
1694.36
1466.93
1383.54
1365.55
1199.61
1136.06
1057.81
1000.64
920.46
781.80
746.85
695.92
414.84
HO
MeO
S27

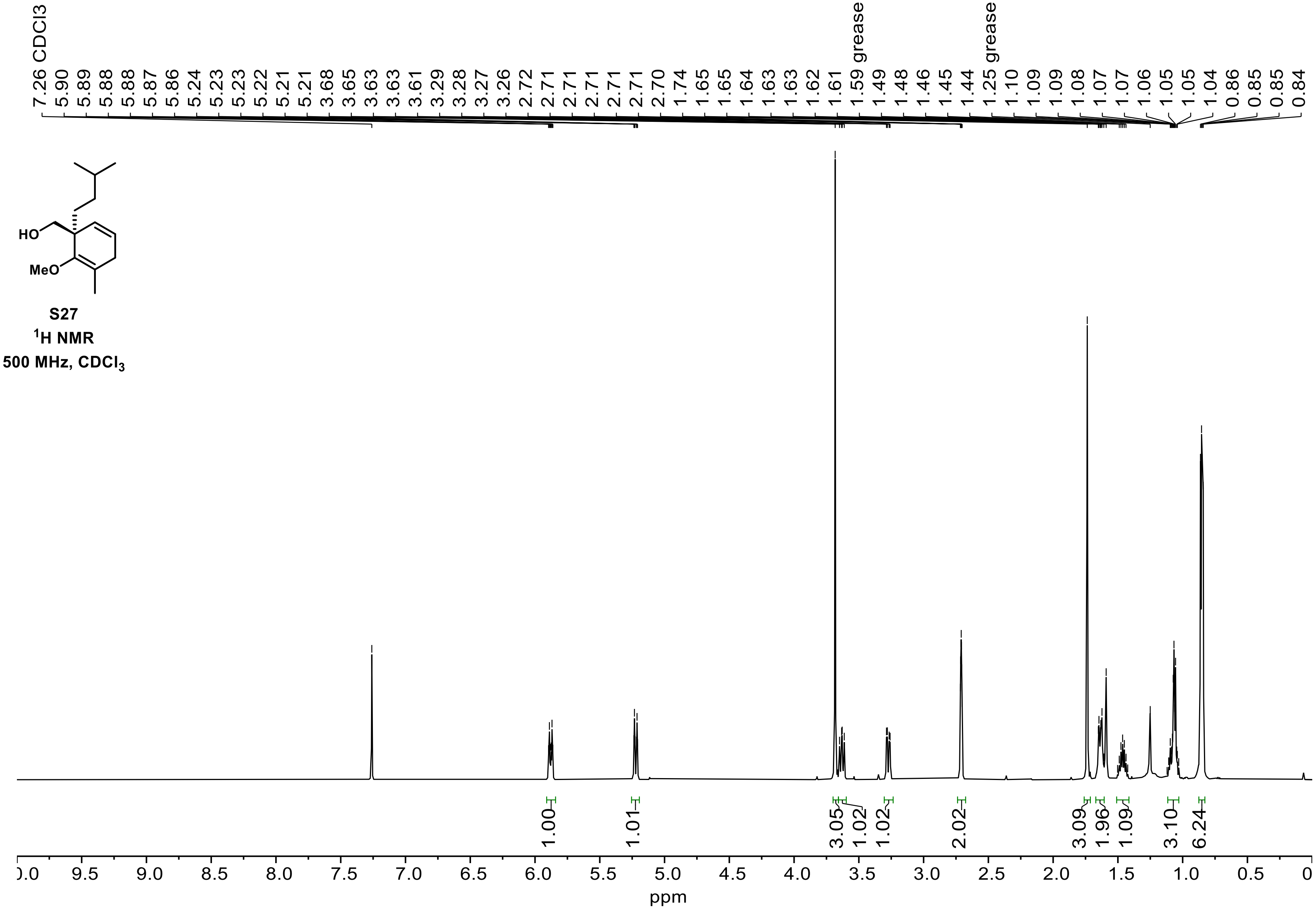

S27
^{1}H NMR
500 MHz, $CDCl_3$
7.26 CDCl3
grease
grease
ppm

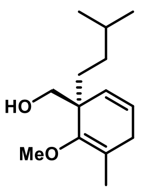

S27
$^{13}C\{^1H\}$ NMR
126 MHz, $CDCl_3$

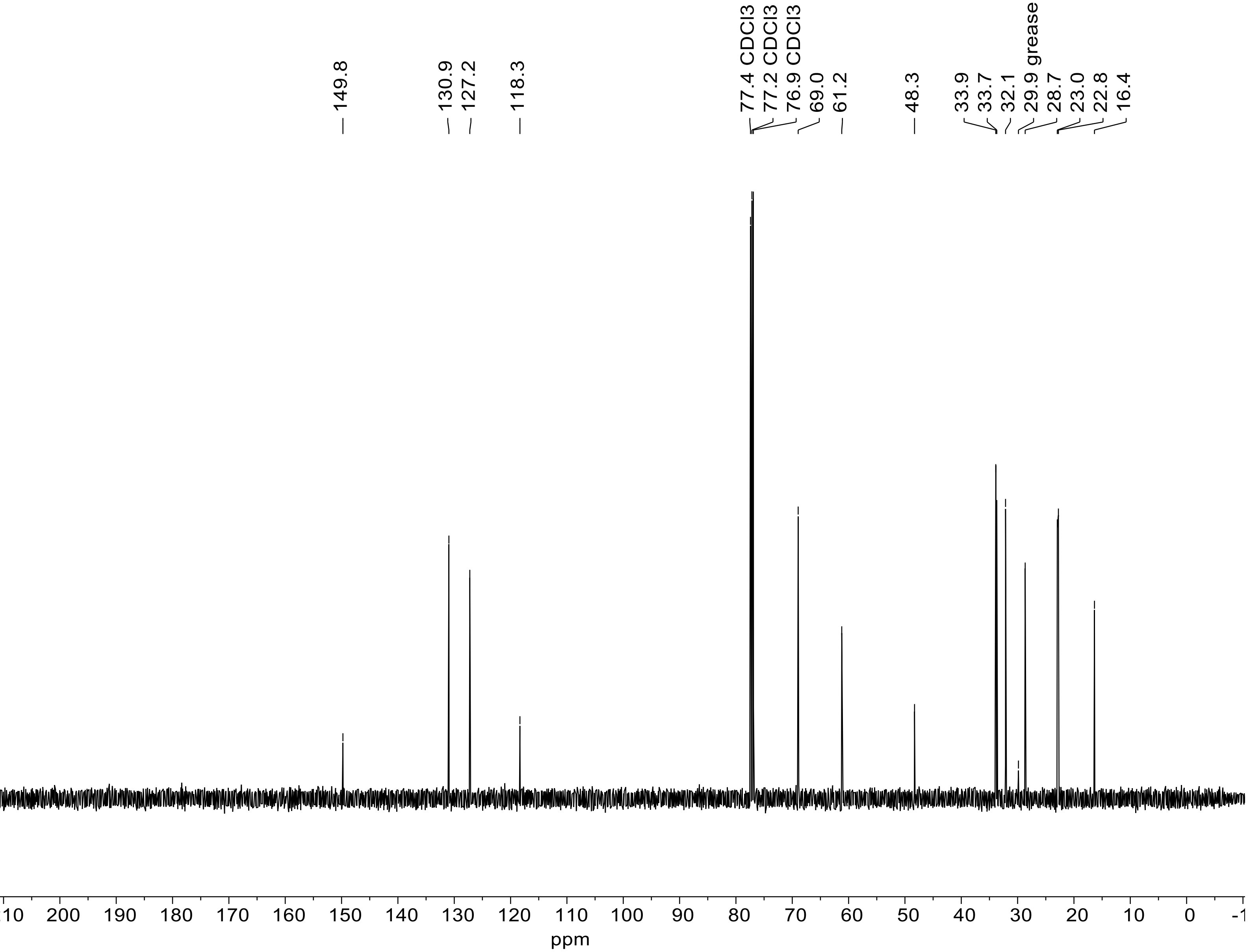

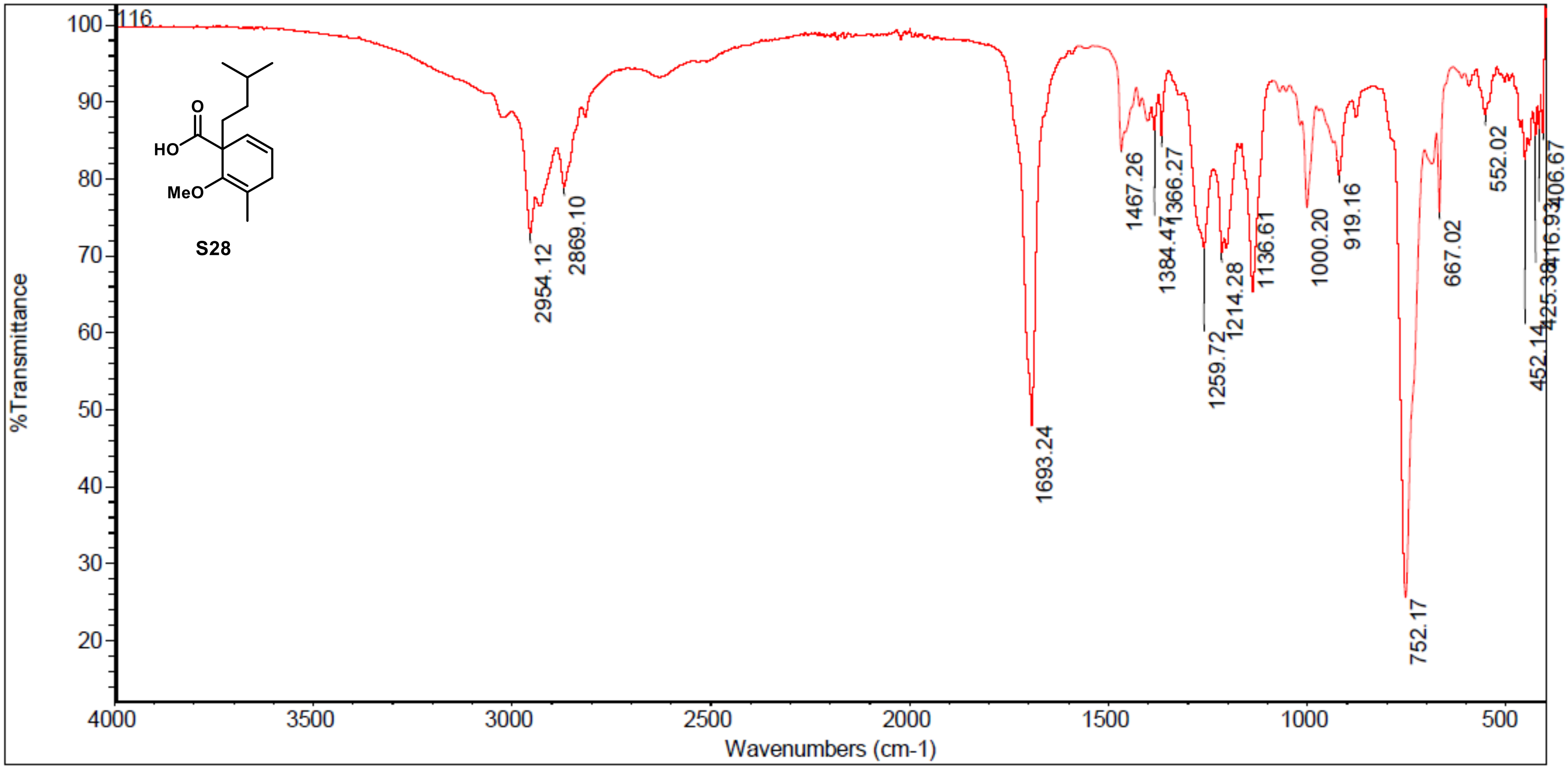

116
HO
O
MeO
S28
%Transmittance
Wavenumbers (cm-1)
2954.12
2869.10
1693.24
1467.26
1384.47
1366.27
1259.72
1214.28
1136.61
1000.20
919.16
752.17
667.02
552.02
452.14
425.38
416.93
406.67

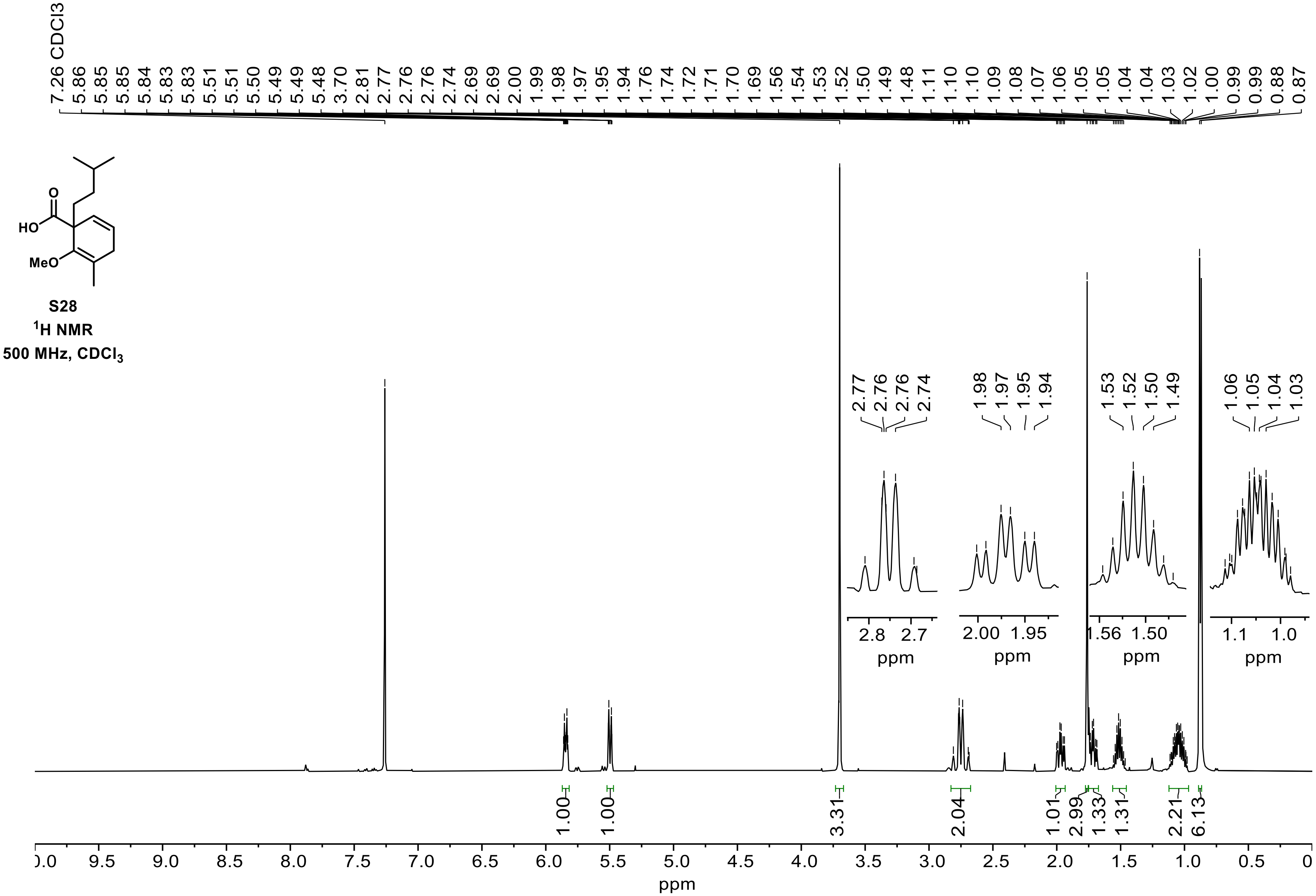
7.26 CDCl3
5.86 5.85 5.85 5.84 5.83 5.83 5.51 5.51 5.50 5.49 5.49 5.48 3.70 2.81 2.77 2.76 2.76 2.74 2.69 2.69 2.00 1.99 1.98 1.97 1.95 1.94 1.76 1.74 1.72 1.71 1.70 1.69 1.56 1.54 1.53 1.52 1.50 1.49 1.48 1.11 1.10 1.10 1.09 1.08 1.07 1.06 1.05 1.05 1.04 1.04 1.03 1.02 1.00 0.99 0.99 0.88 0.87
HO
O
MeO
S28
^{1}H NMR
500 MHz, $CDCl_3$
2.77 2.76 2.76 2.74
2.8 2.7
ppm
1.98 1.97 1.95 1.94
2.00 1.95
ppm
1.53 1.52 1.50 1.49
1.56 1.50
ppm
1.06 1.05 1.04 1.03
1.1 1.0
ppm
1.00 1.00 3.31 2.04 1.01 2.99 1.33 1.31 2.21 6.13
10.0 9.5 9.0 8.5 8.0 7.5 7.0 6.5 6.0 5.5 5.0 4.5 4.0 3.5 3.0 2.5 2.0 1.5 1.0 0.5 0
ppm

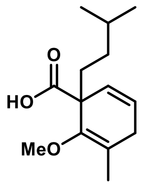

S28
$^{13}C\{^1H\}$ NMR
126 MHz, $CDCl_3$

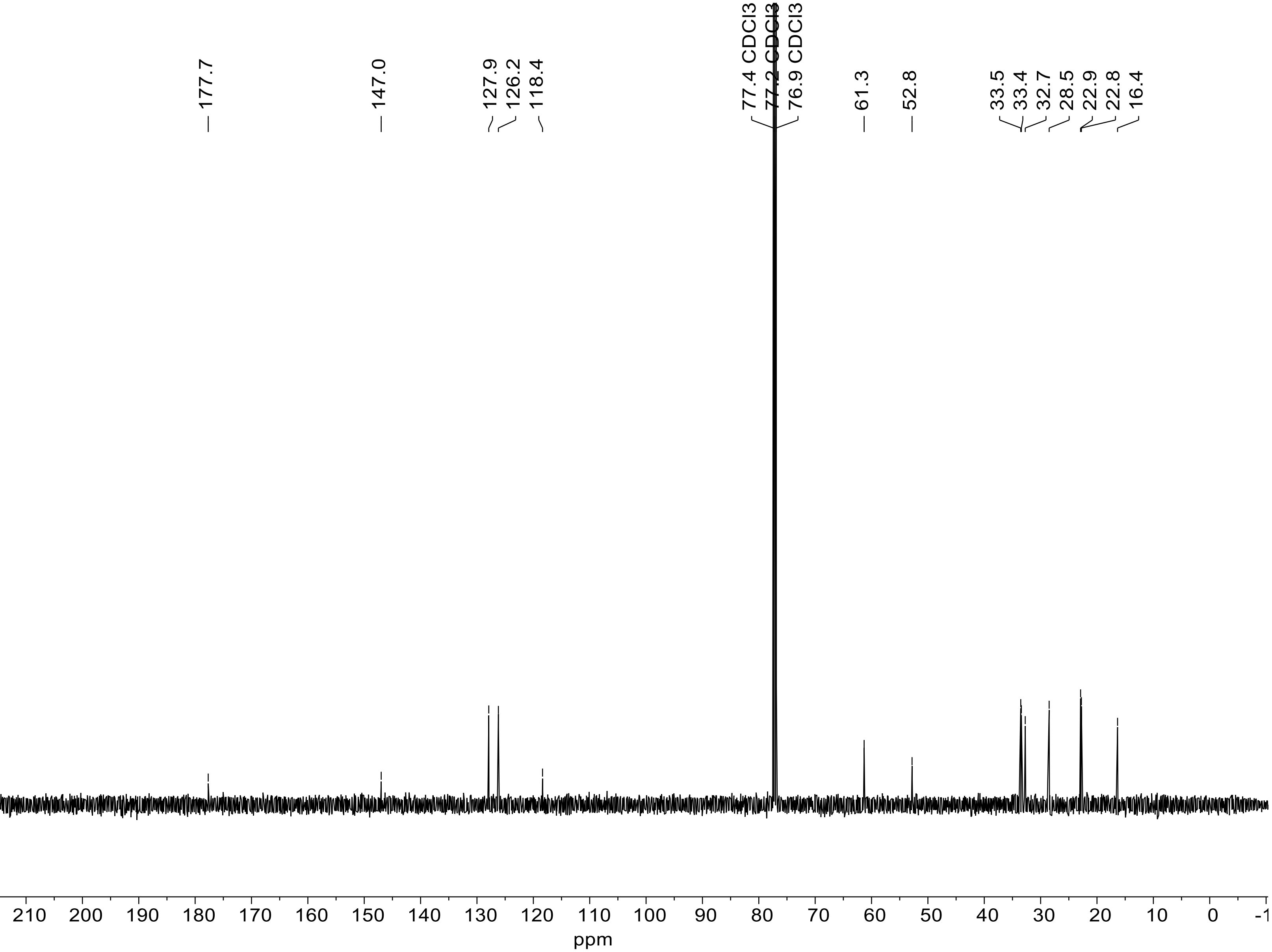

3. SFC data

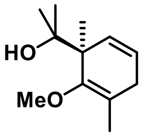

S18

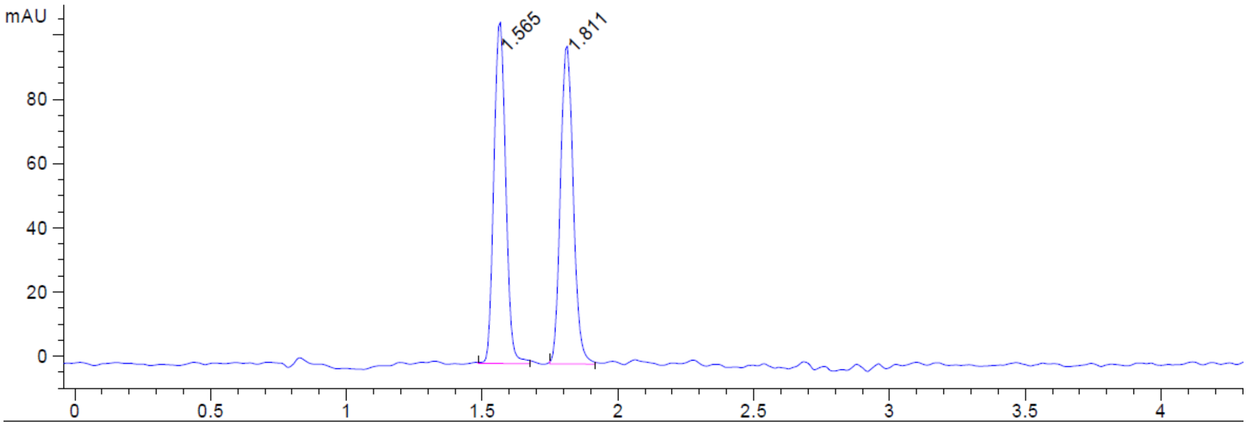

Peak #	RetTime [min]	Type	Width [min]	Area [mAU*s]	Height [mAU]	Area %
1	1.565	BB	0.0520	321.13565	103.97295	49.6797
2	1.811	BB	0.0505	325.27603	98.55428	50.3203

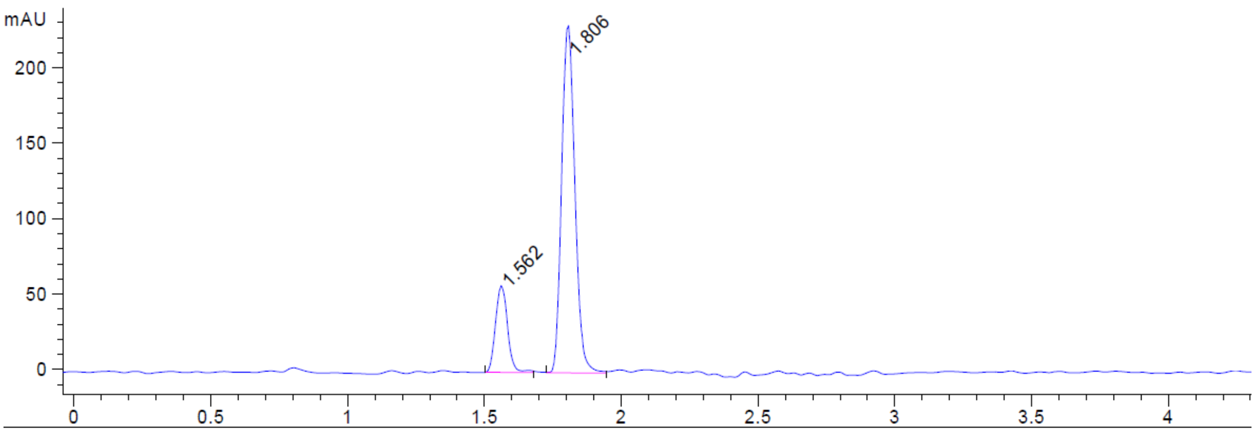

Peak #	RetTime [min]	Type	Width [min]	Area [mAU*s]	Height [mAU]	Area %
1	1.562	BB	0.0525	176.96661	56.53390	18.5992
2	1.806	BB	0.0560	774.50928	225.71388	81.4008

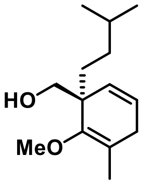

S27

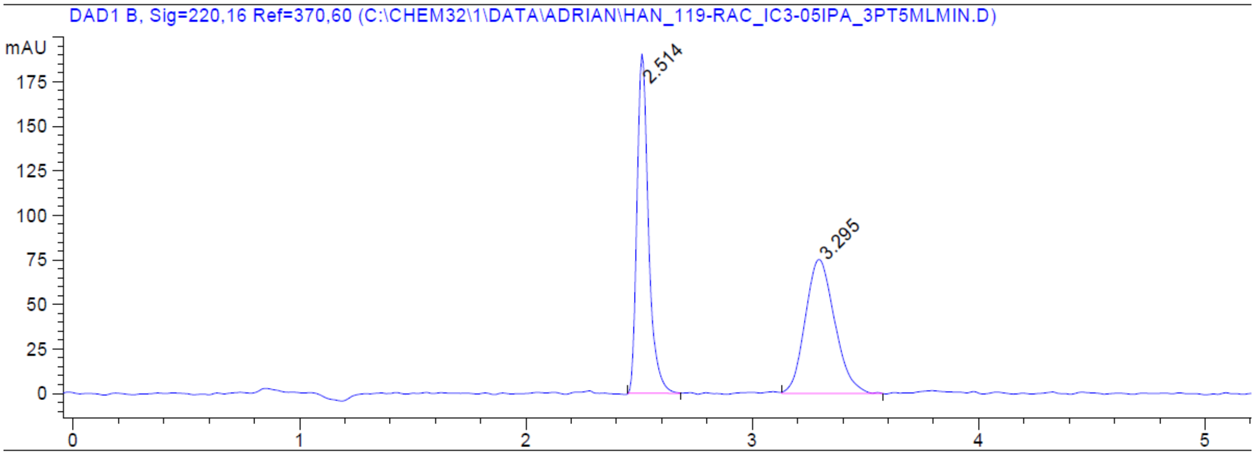

Peak #	RetTime [min]	Type	Width [min]	Area [mAU*s]	Height [mAU]	Area %
1	2.514	BB	0.0578	673.13269	188.02734	49.7281
2	3.295	BB	0.1417	680.49268	75.10517	50.2719

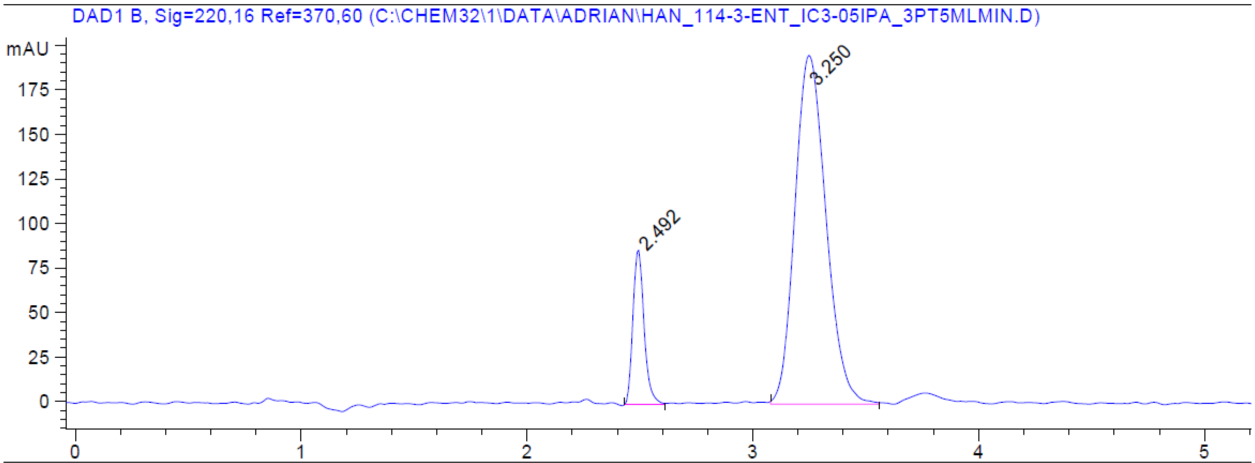

Peak #	RetTime [min]	Type	Width [min]	Area [mAU*s]	Height [mAU]	Area %
1	2.492	BB	0.0522	295.93600	85.97168	13.6200
2	3.250	BB	0.1479	1876.86646	195.61435	86.3800

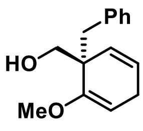

35

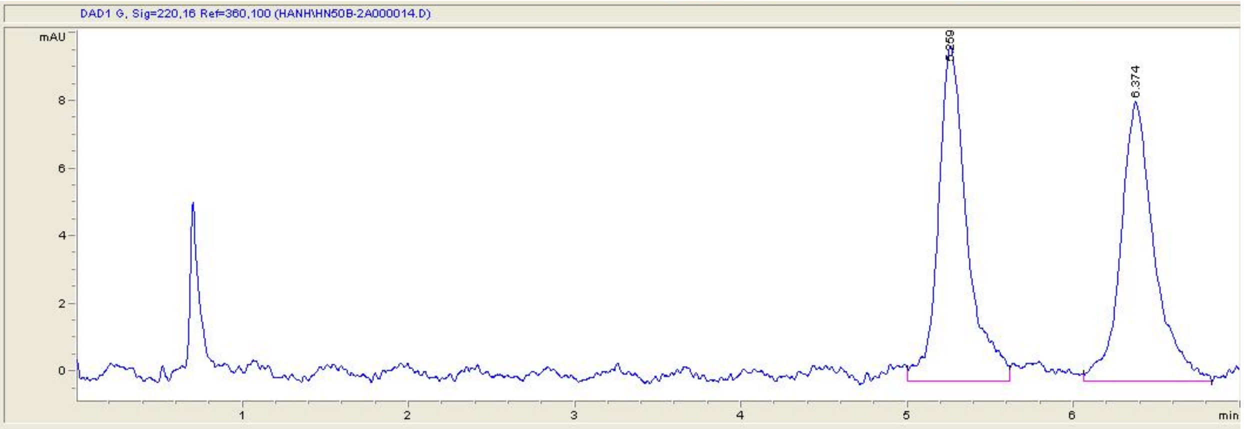

#	Time	Area	Height	Width	Area%	Symmetry
1	5.259	119.3	9.9	0.1714	50.575	0.697
2	6.374	116.6	8.3	0.1853	49.425	0.776

File Information

LC-File	HN50B-2A000014.D
File Path	C:\CHEM32\1\DATA\HANH\
Date	23-Mar-22, 15:46:51
Sample	HN50B-2a
Sample Info	OD-H, 2% IPA in CO2, 2.5 mL/min, 44C, 20 uL inj
Barcode	
Operator	HANH
Method	RUN.M
Analysis Time	6.997 min
Sampling Rate	0.0033 min (0.198 sec), 2100 datapoints

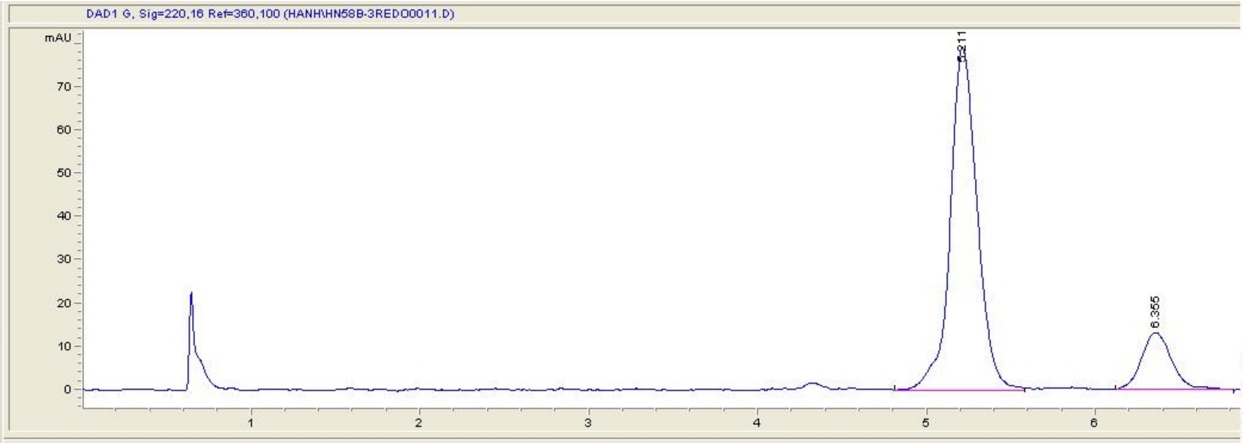

#	Time	Area	Height	Width	Area%	Symmetry
1	5.211	884.9	79.3	0.1702	83.826	0.827
2	6.355	170.7	13.3	0.1784	16.174	0.829

File Information

LC-File	HN58B-3REDO0011.D
File Path	C:\CHEM32\1\DATA\HANH\
Date	23-Mar-22, 15:16:18
Sample	HN58B-3REDO
Sample Info	OD-H, 2% IPA in CO2, 2.5 mL/min, 44C, 20 uL inj

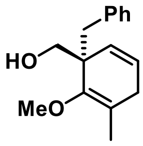

36

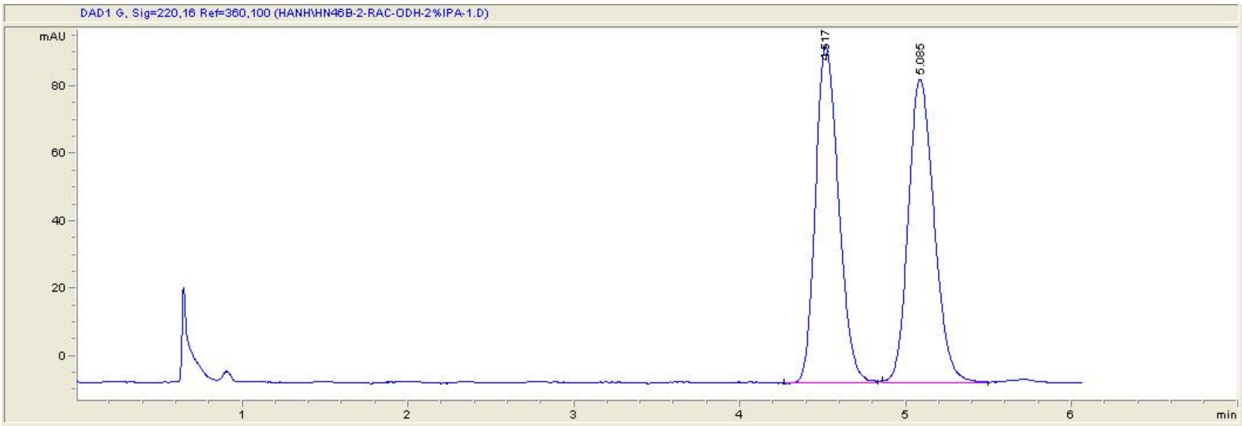

#	Time	Area	Height	Width	Area%	Symmetry
1	4.517	975.6	100.1	0.1537	49.859	0.75
2	5.085	981.2	90.3	0.1678	50.141	0.784

File Information

LC-File	HN46B-2-RAC-ODH-2%IPA-1.D
File Path	C:\CHEM32\1\DATA\HANH\
Date	20-Sep-21, 17:43:47
Sample	HN46B-2-RAC-ODH-2%IPA-1
Sample Info	OD-H, 2 % IPA in CO2, 2.5 mL/min, 44C, 30 uL inj

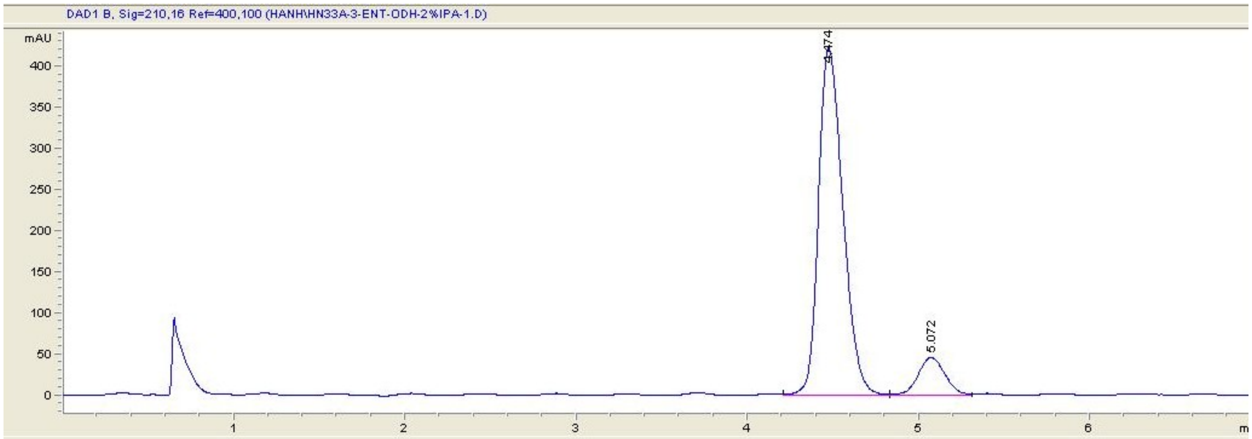

#	Time	Area	Height	Width	Area%	Symmetry
1	4.474	4206.9	422.6	0.1551	89.007	0.68
2	5.072	519.6	46.4	0.1746	10.993	0.906

File Information

LC-File	HN33A-3-ENT-ODH-2%IPA-1.D
File Path	C:\CHEM32\1\DATA\HANH\
Date	20-Sep-21, 17:52:33
Sample	HN33A-3-ENT-ODH-2%IPA-1
Sample Info	OD-H, 2 % IPA in CO2, 2.5 mL/min, 44C, 30 uL inj

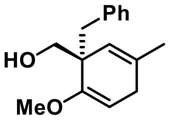

37

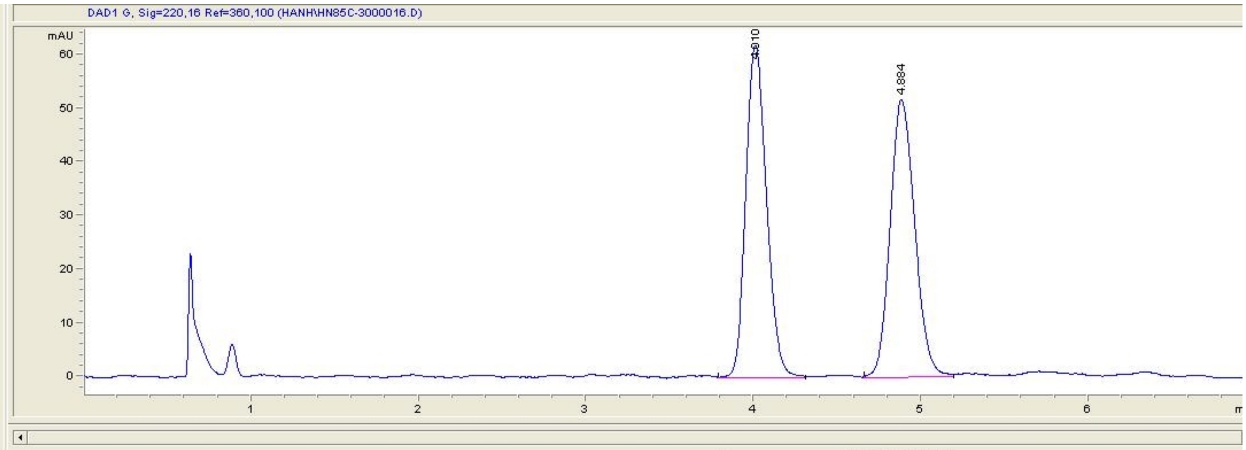

#	Time	Area	Height	Width	Area%	Symmetry
1	4.01	536.4	62	0.1339	50.118	0.787
2	4.884	533.9	51.9	0.1601	49.882	0.813

File Information

LC-File	HN85C-3000016.D
File Path	C:\CHEM32\1\DATA\HANH\
Date	23-Mar-22, 16:05:52
Sample	HN85C-3
Sample Info	OD-H, 2% IPA in CO2, 2.5 mL/min, 44C, 20 uL inj

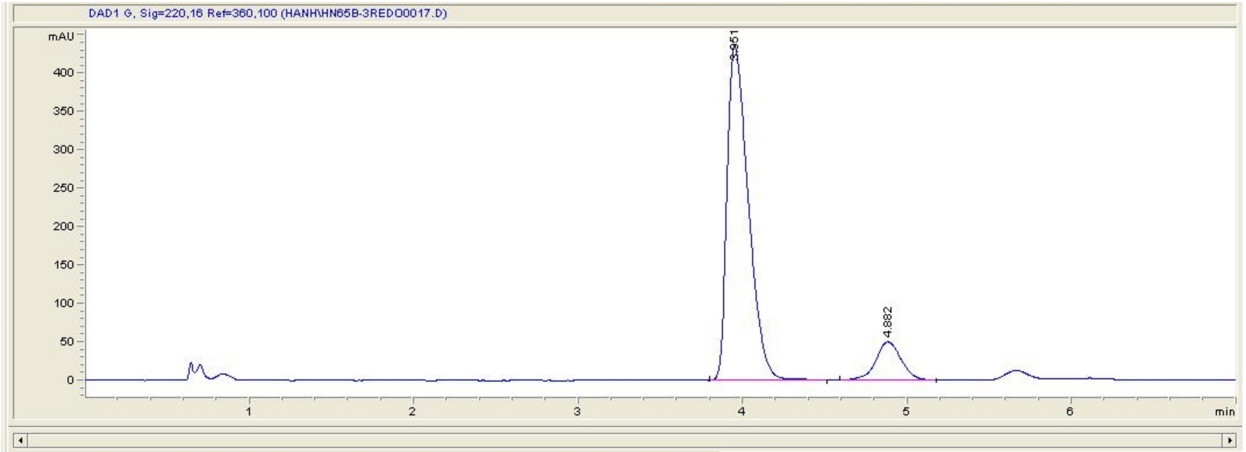

#	Time	Area	Height	Width	Area%	Symmetry
1	3.951	3927.5	435.7	0.1392	87.720	0.52
2	4.882	549.8	50.3	0.1666	12.280	0.902

File Information

LC-File	HN65B-3REDO0017.D
File Path	C:\CHEM32\1\DATA\HANH\
Date	23-Mar-22, 16:15:18
Sample	HN65B-3REDO
Sample Info	OD-H, 2% IPA in CO2, 2.5 mL/min, 44C, 20 uL inj

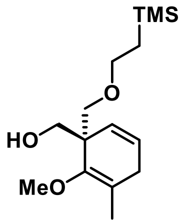

38

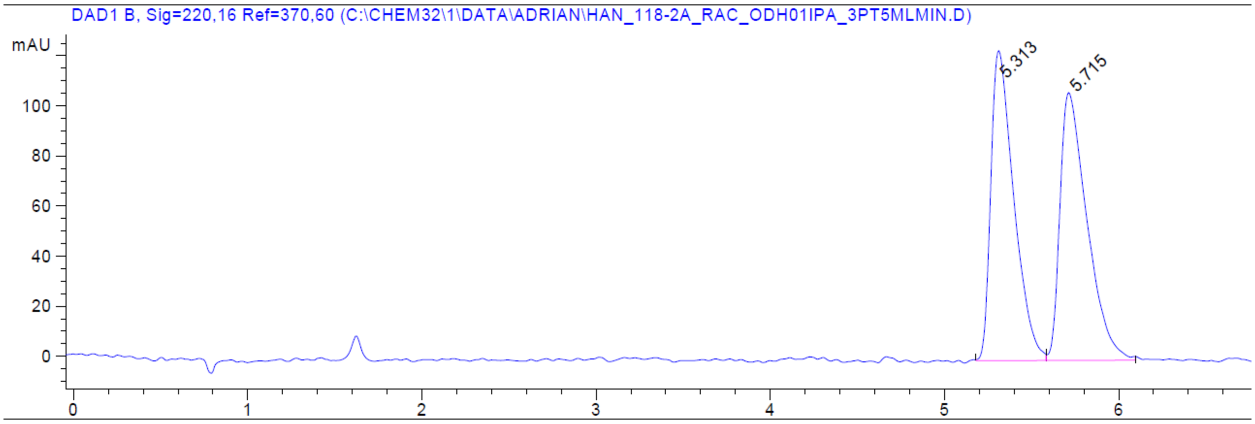

Peak #	RetTime [min]	Type	Width [min]	Area [mAU*s]	Height [mAU]	Area %
1	5.313	BV	0.1392	1137.91370	123.87967	49.5771
2	5.715	VB	0.1621	1157.32776	107.11336	50.4229

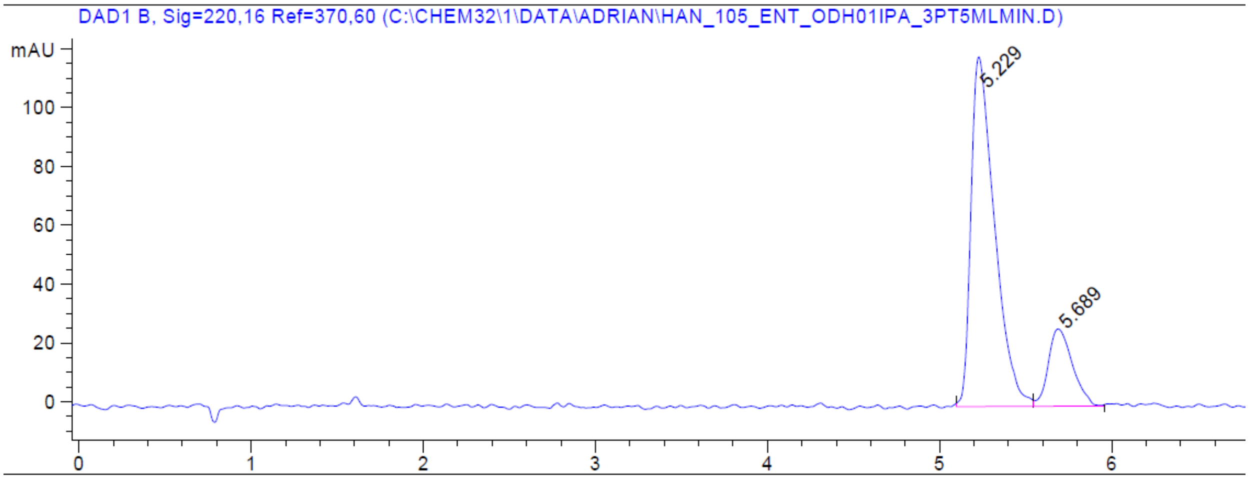

Peak #	RetTime [min]	Type	Width [min]	Area [mAU*s]	Height [mAU]	Area %
1	5.229	BB	0.1429	1131.24585	119.02321	81.4535
2	5.689	BB	0.1547	257.57877	26.19524	18.5465

4. X-Ray Crystallographic Data

X-ray Data Collection, Structure Solution and Refinement for **5**.

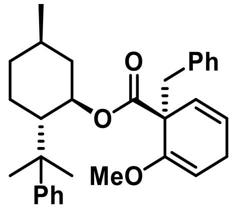

A colorless crystal of approximate dimensions 0.362 x 0.382 x 0.432 mm was mounted on a glass fiber and transferred to a Bruker SMART APEX II diffractometer system. The APEX2[1] program package was used to determine the unit-cell parameters and for data collection (10 sec/frame scan time). The raw frame data was processed using SAINT[2] and SADABS[3] to yield the reflection data file. Subsequent calculations were carried out using the SHELXTL[4] program package. The diffraction symmetry was 2/*m* and the systematic absences were consistent with the monoclinic space groups $P2_1$ and $P2_1/m$. It was later determined that space group $P2_1$ was correct.

The structure was solved by direct methods and refined on F^2 by full-matrix least-squares techniques. The analytical scattering factors5 for neutral atoms were used throughout the analysis. Hydrogen atoms were located from a difference-Fourier map and refined (x,y,z and U_{iso}).

Least-squares analysis yielded wR2 = 0.0965 and Goof = 1.039 for 459 variables refined against 7789 data (0.69 Å), R1 = 0.0375 for those 7183 data with $I > 2.0\sigma(I)$. The absolute structure could not be assigned by refinement of the Flack6 parameter. The assignment was based on the synthetic method.

Definitions:

$wR2 = [\Sigma[w(F_o^2-F_c^2)^2] / \Sigma[w(F_o^2)^2]]^{1/2}$

$R1 = \Sigma||F_o|-|F_c|| / \Sigma|F_o|$

$Goof = S = [\Sigma[w(F_o^2-F_c^2)^2] / (n-p)]^{1/2}$ where n is the number of reflections and p is the total number of parameters refined.

The thermal ellipsoid plot is shown at the 50% probability level.

References.

1. APEX2 Version 2014.11-0, Bruker AXS, Inc.; Madison, WI 2014.
2. SAINT Version 8.34a, Bruker AXS, Inc.; Madison, WI 2013.
3. Sheldrick, G. M. SADABS, Version 2014/5, Bruker AXS, Inc.; Madison, WI 2014.
4. Sheldrick, G. M. SHELXTL, Version 2014/7, Bruker AXS, Inc.; Madison, WI 2014.
5. International Tables for Crystallography 1992, Vol. C., Dordrecht: Kluwer Academic Publishers.
6. Parsons, S., Flack, H. D., Wagner, T. Acta. Cryst. B69, 249-259, 2013.

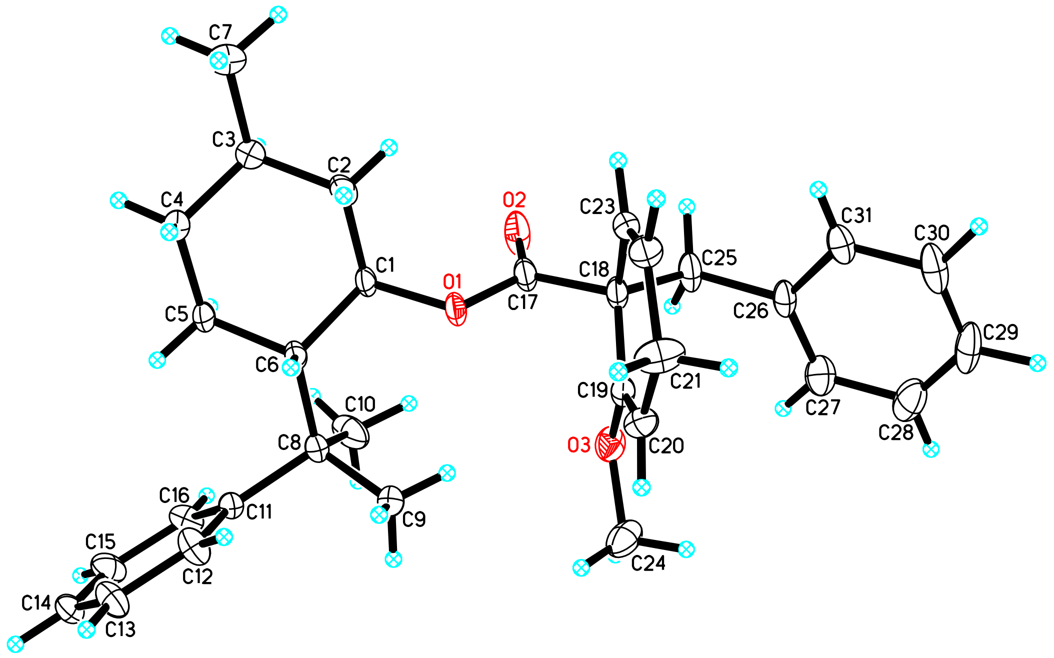
C7
C3
C2
C4
O2
C23
C31
C30
C1
O1
C25
C18
C17
C26
C29
C5
C6
C21
C19
C10
C27
C28
O3
C20
C8
C16
C11
C9
C15
C12
C24
C14
C13

Table S4. Crystal data and structure refinement for **5**.

Identification code	cdv84 (Hanh Nguyen)	
Empirical formula	$C_{31} H_{38} O_3$	
Formula weight	458.61	
Temperature	133(2) K	
Wavelength	0.71073 Å	
Crystal system	Monoclinic	
Space group	$P2_1$	
Unit cell dimensions	a = 10.8664(5) Å	α = 90°.
	b = 9.8173(4) Å	β = 100.7809(7)°.
	c = 12.4405(6) Å	γ = 90°.
Volume	1303.71(10) $Å^3$	
Z	2	
Density (calculated)	1.168 Mg/m^3	
Absorption coefficient	0.073 mm^{-1}	
F(000)	496	
Crystal color	colorless	
Crystal size	0.432 x 0.382 x 0.362 mm^3	
Theta range for data collection	1.666 to 31.055°	
Index ranges	$-15 \le h \le 15$, $-14 \le k \le 14$, $-18 \le l \le 17$	
Reflections collected	32572	
Independent reflections	7789 [R(int) = 0.0327]	
Completeness to theta = 25.242°	100.0 %	
Absorption correction	Semi-empirical from equivalents	
Max. and min. transmission	0.8622 and 0.8201	
Refinement method	Full-matrix least-squares on F^2	
Data / restraints / parameters	7789 / 1 / 459	
Goodness-of-fit on F^2	1.039	
Final R indices [I>2sigma(I) = 7183 data]	R1 = 0.0375, wR2 = 0.0931	
R indices (all data, 0.69 Å)	R1 = 0.0425, wR2 = 0.0965	
Largest diff. peak and hole	0.294 and -0.181 e.$Å^{-3}$	

Table S5. Atomic coordinates (x 10^4) and equivalent isotropic displacement parameters (Å^2 x 10^3)
for cdv84. U(eq) is defined as one third of the trace of the orthogonalized U^{ij} tensor.

	x	y	z	U(eq)
O(1)	4466(1)	366(1)	7566(1)	21(1)
O(2)	3410(1)	-1601(1)	7174(1)	33(1)
O(3)	4304(1)	484(1)	5034(1)	24(1)
C(1)	5182(1)	-231(2)	8567(1)	17(1)
C(2)	4425(1)	6(2)	9463(1)	21(1)
C(3)	5076(1)	-564(2)	10567(1)	22(1)
C(4)	6387(1)	48(2)	10858(1)	25(1)
C(5)	7127(1)	-173(2)	9943(1)	21(1)
C(6)	6485(1)	416(2)	8831(1)	16(1)
C(7)	4302(2)	-301(3)	11450(2)	36(1)
C(8)	7307(1)	229(2)	7925(1)	22(1)
C(9)	6882(2)	1222(3)	6967(2)	40(1)
C(10)	7176(2)	-1224(2)	7469(2)	36(1)
C(11)	8676(1)	560(2)	8422(1)	19(1)
C(12)	9039(1)	1899(2)	8684(1)	25(1)
C(13)	10264(1)	2225(2)	9154(2)	26(1)
C(14)	11158(1)	1207(2)	9385(1)	23(1)
C(15)	10820(1)	-122(2)	9128(2)	26(1)
C(16)	9589(1)	-444(2)	8643(1)	24(1)
C(17)	3566(1)	-418(2)	6987(1)	22(1)
C(18)	2723(1)	413(2)	6088(1)	20(1)
C(19)	3506(1)	1294(2)	5492(1)	18(1)
C(20)	3411(2)	2635(2)	5402(1)	22(1)
C(21)	2486(2)	3446(2)	5897(2)	32(1)
C(22)	1768(1)	2604(2)	6562(1)	24(1)
C(23)	1876(1)	1273(2)	6657(1)	21(1)
C(24)	5010(2)	1163(2)	4331(2)	33(1)
C(25)	1921(2)	-613(2)	5291(1)	25(1)
C(26)	1170(1)	45(2)	4280(1)	21(1)
C(27)	1583(2)	-28(2)	3288(2)	30(1)
C(28)	874(2)	521(2)	2341(2)	36(1)
C(29)	-253(2)	1160(2)	2371(2)	35(1)

C(30)	-672(2)	1255(2)	3350(2)	32(1)
C(31)	37(2)	706(2)	4302(2)	26(1)

Table S6. Bond lengths [Å] and angles [°] for cdv84.

O(1)-C(17)	1.3436(18)
O(1)-C(1)	1.4617(16)
O(2)-C(17)	1.203(2)
O(3)-C(19)	1.3765(18)
O(3)-C(24)	1.431(2)
C(1)-C(2)	1.522(2)
C(1)-C(6)	1.5308(18)
C(1)-H(1A)	0.97(2)
C(2)-C(3)	1.528(2)
C(2)-H(2A)	0.95(2)
C(2)-H(2B)	0.97(3)
C(3)-C(7)	1.526(2)
C(3)-C(4)	1.526(2)
C(3)-H(3A)	0.97(2)
C(4)-C(5)	1.527(2)
C(4)-H(4A)	0.99(3)
C(4)-H(4B)	0.99(3)
C(5)-C(6)	1.541(2)
C(5)-H(5A)	0.95(2)
C(5)-H(5B)	0.94(2)
C(6)-C(8)	1.574(2)
C(6)-H(6A)	1.02(2)
C(7)-H(7A)	0.97(3)
C(7)-H(7B)	1.01(2)
C(7)-H(7C)	1.02(3)
C(8)-C(10)	1.532(3)
C(8)-C(11)	1.5355(19)
C(8)-C(9)	1.542(3)
C(9)-H(9A)	0.91(3)
C(9)-H(9B)	1.06(4)
C(9)-H(9C)	0.93(3)
C(10)-H(10A)	0.91(3)
C(10)-H(10B)	1.01(3)

C(10)-H(10C)	0.97(3)
C(11)-C(16)	1.389(2)
C(11)-C(12)	1.393(2)
C(12)-C(13)	1.387(2)
C(12)-H(12A)	0.94(3)
C(13)-C(14)	1.386(2)
C(13)-H(13A)	0.92(2)
C(14)-C(15)	1.378(3)
C(14)-H(14A)	0.94(2)
C(15)-C(16)	1.397(2)
C(15)-H(15A)	0.95(3)
C(16)-H(16A)	0.94(3)
C(17)-C(18)	1.540(2)
C(18)-C(19)	1.502(2)
C(18)-C(23)	1.518(2)
C(18)-C(25)	1.560(2)
C(19)-C(20)	1.324(2)
C(20)-C(21)	1.502(2)
C(20)-H(20A)	0.91(2)
C(21)-C(22)	1.490(2)
C(21)-H(21A)	0.96(3)
C(21)-H(21B)	0.98(3)
C(22)-C(23)	1.315(2)
C(22)-H(22A)	0.95(2)
C(23)-H(23A)	0.98(3)
C(24)-H(24A)	0.97(3)
C(24)-H(24B)	1.02(3)
C(24)-H(24C)	0.96(2)
C(25)-C(26)	1.511(2)
C(25)-H(25A)	0.96(3)
C(25)-H(25B)	1.01(2)
C(26)-C(27)	1.390(3)
C(26)-C(31)	1.396(2)
C(27)-C(28)	1.390(3)
C(27)-H(27A)	0.95(3)
C(28)-C(29)	1.384(3)
C(28)-H(28A)	0.93(3)
C(29)-C(30)	1.381(3)
C(29)-H(29A)	1.02(3)
C(30)-C(31)	1.394(2)

C(30)-H(30A)	1.00(3)
C(31)-H(31A)	0.90(2)
C(17)-O(1)-C(1)	116.39(11)
C(19)-O(3)-C(24)	115.83(14)
O(1)-C(1)-C(2)	106.80(11)
O(1)-C(1)-C(6)	109.84(11)
C(2)-C(1)-C(6)	112.60(12)
O(1)-C(1)-H(1A)	109.1(12)
C(2)-C(1)-H(1A)	110.7(12)
C(6)-C(1)-H(1A)	107.8(11)
C(1)-C(2)-C(3)	112.30(12)
C(1)-C(2)-H(2A)	109.3(12)
C(3)-C(2)-H(2A)	108.8(12)
C(1)-C(2)-H(2B)	108.8(13)
C(3)-C(2)-H(2B)	109.4(13)
H(2A)-C(2)-H(2B)	108.2(18)
C(7)-C(3)-C(4)	112.27(14)
C(7)-C(3)-C(2)	111.11(14)
C(4)-C(3)-C(2)	108.88(13)
C(7)-C(3)-H(3A)	107.2(12)
C(4)-C(3)-H(3A)	110.9(12)
C(2)-C(3)-H(3A)	106.3(12)
C(3)-C(4)-C(5)	111.48(13)
C(3)-C(4)-H(4A)	106.7(15)
C(5)-C(4)-H(4A)	108.8(16)
C(3)-C(4)-H(4B)	106.6(14)
C(5)-C(4)-H(4B)	114.3(14)
H(4A)-C(4)-H(4B)	109(2)
C(4)-C(5)-C(6)	113.75(12)
C(4)-C(5)-H(5A)	106.6(13)
C(6)-C(5)-H(5A)	108.5(13)
C(4)-C(5)-H(5B)	109.8(12)
C(6)-C(5)-H(5B)	109.5(13)
H(5A)-C(5)-H(5B)	108.5(18)
C(1)-C(6)-C(5)	106.40(11)
C(1)-C(6)-C(8)	115.12(12)
C(5)-C(6)-C(8)	112.11(11)
C(1)-C(6)-H(6A)	109.6(12)
C(5)-C(6)-H(6A)	107.4(12)
C(8)-C(6)-H(6A)	106.0(12)

C(3)-C(7)-H(7A)	110.8(15)
C(3)-C(7)-H(7B)	111.0(14)
H(7A)-C(7)-H(7B)	108(2)
C(3)-C(7)-H(7C)	111.4(15)
H(7A)-C(7)-H(7C)	109(2)
H(7B)-C(7)-H(7C)	106(2)
C(10)-C(8)-C(11)	111.24(14)
C(10)-C(8)-C(9)	107.98(16)
C(11)-C(8)-C(9)	107.77(13)
C(10)-C(8)-C(6)	110.33(13)
C(11)-C(8)-C(6)	108.91(11)
C(9)-C(8)-C(6)	110.58(14)
C(8)-C(9)-H(9A)	111.0(18)
C(8)-C(9)-H(9B)	116.0(18)
H(9A)-C(9)-H(9B)	99(2)
C(8)-C(9)-H(9C)	112.2(18)
H(9A)-C(9)-H(9C)	111(2)
H(9B)-C(9)-H(9C)	107(3)
C(8)-C(10)-H(10A)	112.4(19)
C(8)-C(10)-H(10B)	111.2(16)
H(10A)-C(10)-H(10B)	105(2)
C(8)-C(10)-H(10C)	112.2(16)
H(10A)-C(10)-H(10C)	108(2)
H(10B)-C(10)-H(10C)	107(2)
C(16)-C(11)-C(12)	117.58(13)
C(16)-C(11)-C(8)	122.14(14)
C(12)-C(11)-C(8)	120.28(14)
C(13)-C(12)-C(11)	121.57(15)
C(13)-C(12)-H(12A)	119.3(16)
C(11)-C(12)-H(12A)	119.1(16)
C(14)-C(13)-C(12)	120.08(16)
C(14)-C(13)-H(13A)	120.0(15)
C(12)-C(13)-H(13A)	119.9(15)
C(15)-C(14)-C(13)	119.24(14)
C(15)-C(14)-H(14A)	120.6(15)
C(13)-C(14)-H(14A)	120.1(16)
C(14)-C(15)-C(16)	120.44(15)
C(14)-C(15)-H(15A)	118.5(16)
C(16)-C(15)-H(15A)	121.0(16)
C(11)-C(16)-C(15)	121.07(16)

C(11)-C(16)-H(16A)	122.4(14)
C(15)-C(16)-H(16A)	116.5(14)
O(2)-C(17)-O(1)	124.38(14)
O(2)-C(17)-C(18)	124.45(13)
O(1)-C(17)-C(18)	111.11(13)
C(19)-C(18)-C(23)	111.01(14)
C(19)-C(18)-C(17)	110.35(11)
C(23)-C(18)-C(17)	106.67(12)
C(19)-C(18)-C(25)	111.00(13)
C(23)-C(18)-C(25)	109.90(12)
C(17)-C(18)-C(25)	107.77(13)
C(20)-C(19)-O(3)	125.74(14)
C(20)-C(19)-C(18)	124.90(14)
O(3)-C(19)-C(18)	109.34(13)
C(19)-C(20)-C(21)	122.67(15)
C(19)-C(20)-H(20A)	117.5(14)
C(21)-C(20)-H(20A)	119.9(14)
C(22)-C(21)-C(20)	113.21(15)
C(22)-C(21)-H(21A)	109.5(15)
C(20)-C(21)-H(21A)	111.8(14)
C(22)-C(21)-H(21B)	110.5(18)
C(20)-C(21)-H(21B)	107.2(18)
H(21A)-C(21)-H(21B)	104(2)
C(23)-C(22)-C(21)	123.62(15)
C(23)-C(22)-H(22A)	116.0(14)
C(21)-C(22)-H(22A)	120.2(14)
C(22)-C(23)-C(18)	124.24(14)
C(22)-C(23)-H(23A)	120.9(15)
C(18)-C(23)-H(23A)	114.9(15)
O(3)-C(24)-H(24A)	99.3(18)
O(3)-C(24)-H(24B)	111.7(15)
H(24A)-C(24)-H(24B)	117(2)
O(3)-C(24)-H(24C)	108.3(13)
H(24A)-C(24)-H(24C)	110(2)
H(24B)-C(24)-H(24C)	110(2)
C(26)-C(25)-C(18)	113.83(13)
C(26)-C(25)-H(25A)	108.7(15)
C(18)-C(25)-H(25A)	104.5(15)
C(26)-C(25)-H(25B)	110.7(13)
C(18)-C(25)-H(25B)	106.8(13)

H(25A)-C(25)-H(25B)	112(2)
C(27)-C(26)-C(31)	118.24(15)
C(27)-C(26)-C(25)	120.23(15)
C(31)-C(26)-C(25)	121.51(15)
C(26)-C(27)-C(28)	120.87(17)
C(26)-C(27)-H(27A)	117.4(16)
C(28)-C(27)-H(27A)	121.7(16)
C(29)-C(28)-C(27)	120.33(18)
C(29)-C(28)-H(28A)	121.1(18)
C(27)-C(28)-H(28A)	118.5(19)
C(30)-C(29)-C(28)	119.64(16)
C(30)-C(29)-H(29A)	118.5(15)
C(28)-C(29)-H(29A)	121.8(15)
C(29)-C(30)-C(31)	120.08(17)
C(29)-C(30)-H(30A)	119.5(15)
C(31)-C(30)-H(30A)	120.5(15)
C(30)-C(31)-C(26)	120.82(17)
C(30)-C(31)-H(31A)	121.1(14)
C(26)-C(31)-H(31A)	117.8(14)

Table S7. Anisotropic displacement parameters ($Å^2 \times 10^3$) for cdv84. The anisotropic displacement factor exponent takes the form: -2□^{2}[$h^2 a^{*2}U^{11}$ + ... + 2 h k a* b* U^{12}]

	U^{11}	U^{22}	U^{33}	U^{23}	U^{13}	U^{12}
O(1)	16(1)	21(1)	21(1)	7(1)	-7(1)	-4(1)
O(2)	32(1)	23(1)	35(1)	10(1)	-16(1)	-10(1)
O(3)	24(1)	26(1)	23(1)	2(1)	3(1)	8(1)
C(1)	13(1)	18(1)	17(1)	4(1)	-4(1)	0(1)
C(2)	14(1)	23(1)	24(1)	3(1)	1(1)	0(1)
C(3)	19(1)	28(1)	21(1)	2(1)	4(1)	-1(1)
C(4)	19(1)	39(1)	17(1)	-1(1)	1(1)	-2(1)
C(5)	14(1)	30(1)	16(1)	1(1)	-2(1)	1(1)
C(6)	13(1)	19(1)	16(1)	1(1)	-2(1)	-1(1)
C(7)	28(1)	56(1)	28(1)	-2(1)	12(1)	-5(1)
C(8)	16(1)	33(1)	16(1)	-2(1)	0(1)	-6(1)
C(9)	22(1)	71(2)	24(1)	20(1)	-5(1)	-15(1)
C(10)	27(1)	50(1)	33(1)	-23(1)	11(1)	-15(1)
C(11)	16(1)	26(1)	15(1)	-2(1)	2(1)	-4(1)

C(12)	18(1)	24(1)	32(1)	-4(1)	0(1)	1(1)
C(13)	18(1)	26(1)	32(1)	-9(1)	3(1)	-4(1)
C(14)	14(1)	33(1)	23(1)	-2(1)	3(1)	-3(1)
C(15)	17(1)	28(1)	35(1)	4(1)	7(1)	2(1)
C(16)	20(1)	22(1)	30(1)	-2(1)	9(1)	-3(1)
C(17)	17(1)	23(1)	22(1)	5(1)	-6(1)	-4(1)
C(18)	17(1)	19(1)	19(1)	4(1)	-6(1)	-3(1)
C(19)	17(1)	22(1)	15(1)	1(1)	-1(1)	1(1)
C(20)	25(1)	21(1)	21(1)	1(1)	6(1)	-2(1)
C(21)	44(1)	21(1)	36(1)	1(1)	21(1)	2(1)
C(22)	22(1)	30(1)	21(1)	-1(1)	5(1)	1(1)
C(23)	16(1)	28(1)	18(1)	4(1)	0(1)	-4(1)
C(24)	34(1)	44(1)	23(1)	7(1)	11(1)	16(1)
C(25)	24(1)	20(1)	26(1)	3(1)	-11(1)	-4(1)
C(26)	21(1)	17(1)	22(1)	1(1)	-7(1)	-2(1)
C(27)	30(1)	31(1)	26(1)	-4(1)	-2(1)	1(1)
C(28)	46(1)	36(1)	21(1)	-1(1)	-4(1)	-8(1)
C(29)	43(1)	22(1)	30(1)	4(1)	-20(1)	-7(1)
C(30)	26(1)	23(1)	40(1)	-3(1)	-15(1)	1(1)
C(31)	20(1)	26(1)	28(1)	-3(1)	-5(1)	-2(1)

Table S8. Hydrogen coordinates (x 10^4) and isotropic displacement parameters (Å^2 x 10^3) for cdv84.

	x	y	z	U(eq)
H(1A)	5289(18)	-1190(20)	8454(16)	17(4)
H(2A)	3630(18)	-420(20)	9263(16)	16(4)
H(2B)	4300(20)	980(30)	9531(18)	27(5)
H(3A)	5117(19)	-1540(20)	10475(17)	22(5)
H(4A)	6280(20)	1040(30)	10950(20)	42(7)
H(4B)	6780(20)	-350(30)	11570(20)	35(6)
H(5A)	7208(19)	-1120(30)	9868(17)	24(5)
H(5B)	7936(19)	210(20)	10145(16)	20(5)
H(6A)	6400(20)	1450(20)	8928(18)	24(5)
H(7A)	4690(20)	-720(30)	12140(20)	40(6)

H(7B)	3430(20)	-680(30)	11230(20)	34(6)
H(7C)	4200(20)	710(30)	11570(20)	43(7)
H(9A)	7460(30)	1260(30)	6520(20)	45(7)
H(9B)	6860(30)	2260(40)	7180(30)	61(9)
H(9C)	6100(30)	1000(30)	6570(20)	49(7)
H(10A)	7690(30)	-1390(30)	6990(20)	49(7)
H(10B)	6300(20)	-1400(30)	7050(20)	40(6)
H(10C)	7340(20)	-1900(30)	8040(20)	38(7)
H(12A)	8430(20)	2590(30)	8560(20)	40(6)
H(13A)	10480(20)	3110(20)	9309(19)	29(5)
H(14A)	11990(20)	1430(30)	9700(20)	35(6)
H(15A)	11440(20)	-810(30)	9280(20)	40(6)
H(16A)	9420(20)	-1370(30)	8484(18)	25(5)
H(20A)	3950(20)	3070(20)	5041(19)	23(5)
H(21A)	2880(20)	4190(30)	6330(20)	34(6)
H(21B)	1910(30)	3890(30)	5290(30)	56(8)
H(22A)	1150(20)	3010(20)	6910(19)	30(6)
H(23A)	1390(20)	760(30)	7110(20)	38(6)
H(24A)	5430(30)	380(30)	4090(20)	51(7)
H(24B)	5560(20)	1910(30)	4730(20)	39(6)
H(24C)	4430(20)	1550(20)	3728(19)	27(5)
H(25A)	2530(20)	-1220(30)	5070(20)	35(6)
H(25B)	1350(20)	-1090(30)	5722(18)	30(5)
H(27A)	2350(20)	-480(30)	3280(20)	43(7)
H(28A)	1160(30)	410(30)	1690(20)	51(7)
H(29A)	-810(20)	1540(30)	1680(20)	45(7)
H(30A)	-1500(20)	1700(30)	3370(20)	41(7)
H(31A)	-250(20)	690(20)	4930(20)	28(6)

Table S9. Torsion angles [°] for cdv84.

C(17)-O(1)-C(1)-C(2)	-84.20(15)
C(17)-O(1)-C(1)-C(6)	153.41(13)
O(1)-C(1)-C(2)-C(3)	-179.57(12)
C(6)-C(1)-C(2)-C(3)	-58.94(17)
C(1)-C(2)-C(3)-C(7)	179.54(15)
C(1)-C(2)-C(3)-C(4)	55.39(18)
C(7)-C(3)-C(4)-C(5)	-177.56(16)

C(2)-C(3)-C(4)-C(5)	-54.10(19)
C(3)-C(4)-C(5)-C(6)	57.42(19)
O(1)-C(1)-C(6)-C(5)	175.06(12)
C(2)-C(1)-C(6)-C(5)	56.19(16)
O(1)-C(1)-C(6)-C(8)	-60.10(16)
C(2)-C(1)-C(6)-C(8)	-178.98(13)
C(4)-C(5)-C(6)-C(1)	-56.06(17)
C(4)-C(5)-C(6)-C(8)	177.28(14)
C(1)-C(6)-C(8)-C(10)	-41.93(18)
C(5)-C(6)-C(8)-C(10)	79.87(16)
C(1)-C(6)-C(8)-C(11)	-164.30(13)
C(5)-C(6)-C(8)-C(11)	-42.50(18)
C(1)-C(6)-C(8)-C(9)	77.45(17)
C(5)-C(6)-C(8)-C(9)	-160.75(14)
C(10)-C(8)-C(11)-C(16)	-13.7(2)
C(9)-C(8)-C(11)-C(16)	-131.88(18)
C(6)-C(8)-C(11)-C(16)	108.11(16)
C(10)-C(8)-C(11)-C(12)	167.30(15)
C(9)-C(8)-C(11)-C(12)	49.1(2)
C(6)-C(8)-C(11)-C(12)	-70.89(18)
C(16)-C(11)-C(12)-C(13)	-0.3(2)
C(8)-C(11)-C(12)-C(13)	178.75(15)
C(11)-C(12)-C(13)-C(14)	-0.8(3)
C(12)-C(13)-C(14)-C(15)	1.1(3)
C(13)-C(14)-C(15)-C(16)	-0.3(3)
C(12)-C(11)-C(16)-C(15)	1.1(2)
C(8)-C(11)-C(16)-C(15)	-177.94(14)
C(14)-C(15)-C(16)-C(11)	-0.8(3)
C(1)-O(1)-C(17)-O(2)	-8.3(2)
C(1)-O(1)-C(17)-C(18)	168.94(12)
O(2)-C(17)-C(18)-C(19)	-137.26(18)
O(1)-C(17)-C(18)-C(19)	45.48(18)
O(2)-C(17)-C(18)-C(23)	102.1(2)
O(1)-C(17)-C(18)-C(23)	-75.20(15)
O(2)-C(17)-C(18)-C(25)	-15.9(2)
O(1)-C(17)-C(18)-C(25)	166.82(13)
C(24)-O(3)-C(19)-C(20)	-5.3(2)
C(24)-O(3)-C(19)-C(18)	173.00(13)
C(23)-C(18)-C(19)-C(20)	-4.7(2)
C(17)-C(18)-C(19)-C(20)	-122.70(16)

C(25)-C(18)-C(19)-C(20)	117.89(17)
C(23)-C(18)-C(19)-O(3)	177.03(11)
C(17)-C(18)-C(19)-O(3)	58.98(16)
C(25)-C(18)-C(19)-O(3)	-60.43(15)
O(3)-C(19)-C(20)-C(21)	177.92(15)
C(18)-C(19)-C(20)-C(21)	-0.1(3)
C(19)-C(20)-C(21)-C(22)	4.5(3)
C(20)-C(21)-C(22)-C(23)	-3.8(3)
C(21)-C(22)-C(23)-C(18)	-1.2(3)
C(19)-C(18)-C(23)-C(22)	5.3(2)
C(17)-C(18)-C(23)-C(22)	125.58(16)
C(25)-C(18)-C(23)-C(22)	-117.85(16)
C(19)-C(18)-C(25)-C(26)	-51.25(19)
C(23)-C(18)-C(25)-C(26)	71.93(17)
C(17)-C(18)-C(25)-C(26)	-172.20(14)
C(18)-C(25)-C(26)-C(27)	101.61(18)
C(18)-C(25)-C(26)-C(31)	-80.11(19)
C(31)-C(26)-C(27)-C(28)	-1.2(3)
C(25)-C(26)-C(27)-C(28)	177.14(17)
C(26)-C(27)-C(28)-C(29)	0.5(3)
C(27)-C(28)-C(29)-C(30)	0.1(3)
C(28)-C(29)-C(30)-C(31)	-0.1(3)
C(29)-C(30)-C(31)-C(26)	-0.6(3)
C(27)-C(26)-C(31)-C(30)	1.2(2)
C(25)-C(26)-C(31)-C(30)	-177.09(15)

X-ray Data Collection, Structure Solution and Refinement for **10**.

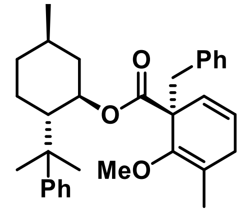

A colorless crystal of approximate dimensions 0.062 x 0.142 x 0.181 mm was mounted in a cryoloop and transferred to a Bruker X8 Prospector diffractometer system. The APEX31 program package was used to determine the unit-cell parameters and for data collection (10 sec/frame scan time). The raw frame data was processed using SAINT2 and SADABS3 to yield the reflection data file. Subsequent calculations were carried out using the SHELXTL4 program package. The systematic absences were consistent with the hexagonal space groups P61 and P65. Space group P61 was assigned and later determined to be correct.

The structure was solved by direct methods and refined on F2 by full-matrix least-squares techniques. The analytical scattering factors5 for neutral atoms were used throughout the analysis. Hydrogen atoms were included using a riding model.

Least-squares analysis yielded wR2 = 0.0696 and Goof = 1.031 for 322 variables refined against 4994 data (0.83 Å), R1 = 0.0279 for those 4721 data with I > 2.0σ(I). The absolute structure was assigned by refinement of the Flack6 parameter.

References.

7. APEX3 Version 2018.1-0, Bruker AXS, Inc.; Madison, WI 2018.
8. SAINT Version 8.38a, Bruker AXS, Inc.; Madison, WI 2013.
9. Sheldrick, G. M. SADABS, Version 2014/5, Bruker AXS, Inc.; Madison, WI 2014.
10. Sheldrick, G. M. SHELXTL, Version 2014/7, Bruker AXS, Inc.; Madison, WI 2014.
11. International Tables for Crystallography 1992, Vol. C., Dordrecht: Kluwer Academic Publishers.
12. Parsons, S., Flack, H. D., Wagner, T. Acta Cryst. B69, 249-259, 2013.

Definitions:

$wR2 = [\Sigma[w(F_o^2-F_c^2)^2] / \Sigma[w(F_o^2)^2]]^{1/2}$

$R1 = \Sigma||F_o|-|F_c|| / \Sigma|F_o|$

$Goof = S = [\Sigma[w(F_o^2-F_c^2)^2] / (n-p)]^{1/2}$ where n is the number of reflections and p is the total number of parameters refined.

The thermal ellipsoid plot is shown at the 50% probability level.

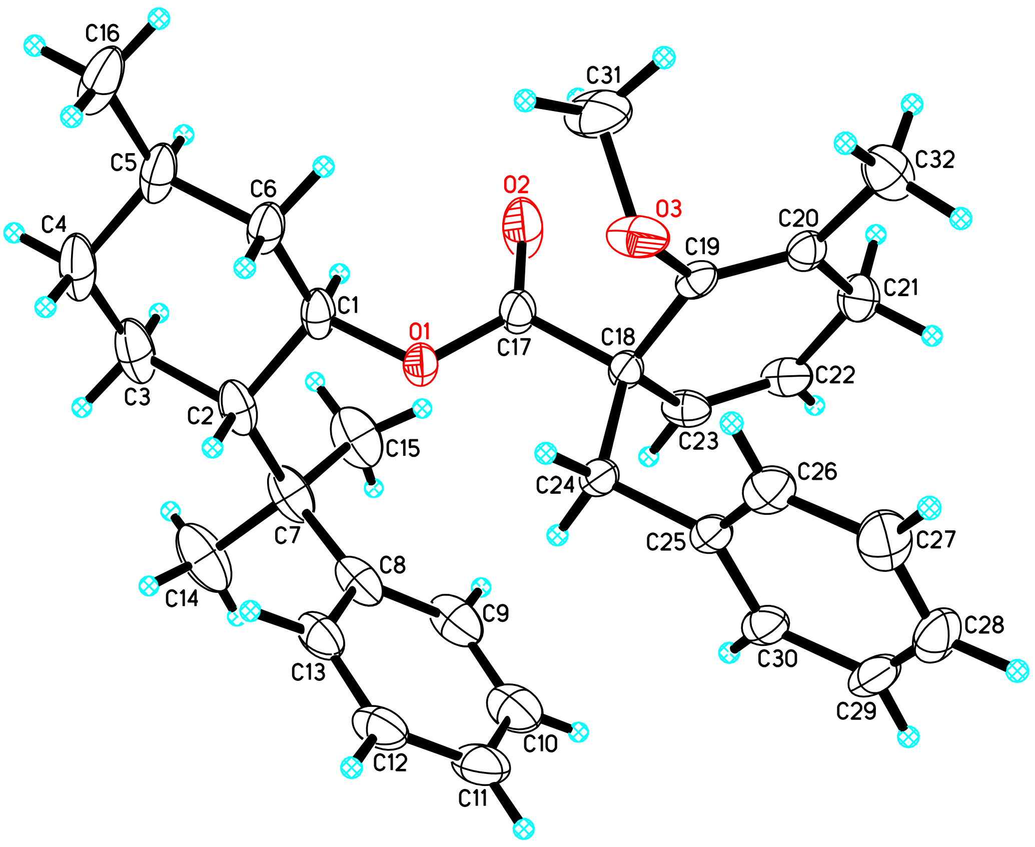
C16
C5
C6
C4
C1
C3
C2
O1
C17
O2
C31
O3
C19
C20
C32
C21
C18
C22
C23
C15
C7
C8
C14
C9
C13
C12
C11
C10
C24
C25
C26
C27
C28
C30
C29

Table S10. Crystal data and structure refinement for **10**.

Identification code	cdv99 (Hanh Ngyuen)	
Empirical formula	C_{32} H_{40} O_3	
Formula weight	472.64	
Temperature	93(2) K	
Wavelength	1.54178 Å	
Crystal system	Hexagonal	
Space group	$P6_1$	
Unit cell dimensions	a = 10.6086(4) Å	a = 90°.
	b = 10.6086(4) Å	b = 90°.
	c = 41.8143(17) Å	g = 120°.
Volume	4075.4(3) Å^3	
Z	6	
Density (calculated)	1.155 Mg/m^3	
Absorption coefficient	0.562 mm^{-1}	
F(000)	1536	
Crystal color	colorless	
Crystal size	0.181 x 0.142 x 0.062 mm^3	
Theta range for data collection	4.813 to 68.616°	
Index ranges	$-12 \leq h \leq 12$, $-12 \leq k \leq 12$, $-49 \leq l \leq 50$	
Reflections collected	54716	
Independent reflections	4994 [R(int) = 0.0569]	
Completeness to theta = 67.679°	100.0 %	
Absorption correction	Semi-empirical from equivalents	
Max. and min. transmission	0.8643 and 0.7925	
Refinement method	Full-matrix least-squares on F^2	
Data / restraints / parameters	4994 / 1 / 322	
Goodness-of-fit on F^2	1.031	
Final R indices [I>2sigma(I) = 4721 data]	R1 = 0.0279, wR2 = 0.0681	
R indices (all data, 0.83 Å)	R1 = 0.0303, wR2 = 0.0696	
Absolute structure parameter	-0.07(8)	
Extinction coefficient	0.00058(11)	
Largest diff. peak and hole	0.120 and -0.119 e.Å^{-3}	

Table S11. Atomic coordinates (x 10^4) and equivalent isotropic displacement parameters ($Å^2$ x 10^3)
for cdv99. U(eq) is defined as one third of the trace of the orthogonalized U^{ij} tensor.

	x	y	z	U(eq)
O(1)	-451(1)	1621(1)	4788(1)	23(1)
O(2)	-1659(2)	693(3)	5247(1)	60(1)
O(3)	-601(2)	-1667(2)	5031(1)	37(1)
C(1)	-1629(2)	1843(2)	4673(1)	27(1)
C(2)	-963(2)	3210(2)	4462(1)	31(1)
C(3)	-2244(3)	3381(3)	4340(1)	44(1)
C(4)	-3372(3)	2035(3)	4162(1)	45(1)
C(5)	-3993(2)	667(3)	4366(1)	37(1)
C(6)	-2730(2)	493(2)	4494(1)	30(1)
C(7)	272(3)	4589(2)	4631(1)	36(1)
C(8)	1736(2)	4628(2)	4623(1)	33(1)
C(9)	2704(3)	5135(2)	4881(1)	40(1)
C(10)	4081(3)	5290(2)	4862(1)	44(1)
C(11)	4541(3)	4933(3)	4586(1)	43(1)
C(12)	3602(2)	4424(2)	4325(1)	37(1)
C(13)	2232(2)	4281(2)	4344(1)	33(1)
C(14)	550(4)	5987(3)	4454(1)	52(1)
C(15)	-182(3)	4712(3)	4976(1)	45(1)
C(16)	-5107(3)	-698(3)	4189(1)	48(1)
C(17)	-615(2)	1033(2)	5078(1)	28(1)
C(18)	661(2)	843(2)	5189(1)	21(1)
C(19)	44(2)	-721(2)	5288(1)	22(1)
C(20)	232(2)	-1169(2)	5571(1)	23(1)
C(21)	945(2)	-111(2)	5841(1)	35(1)
C(22)	1477(2)	1431(2)	5756(1)	31(1)
C(23)	1349(2)	1865(2)	5469(1)	26(1)
C(24)	1818(2)	1225(2)	4920(1)	22(1)
C(25)	3008(2)	922(2)	5019(1)	22(1)

C(26)	2812(2)	-473(2)	4991(1)	29(1)
C(27)	3871(3)	-781(3)	5096(1)	40(1)
C(28)	5146(2)	302(3)	5229(1)	42(1)
C(29)	5365(2)	1690(3)	5254(1)	38(1)
C(30)	4303(2)	2005(2)	5151(1)	28(1)
C(31)	-2140(3)	-2568(3)	5046(1)	53(1)
C(32)	-164(3)	-2710(2)	5642(1)	37(1)

Table S12. Bond lengths [Å] and angles [°] for cdv99.

O(1)-C(17)	1.335(2)
O(1)-C(1)	1.465(2)
O(2)-C(17)	1.206(2)
O(3)-C(19)	1.395(2)
O(3)-C(31)	1.422(3)
C(1)-C(6)	1.517(3)
C(1)-C(2)	1.532(3)
C(2)-C(3)	1.546(3)
C(2)-C(7)	1.561(3)
C(3)-C(4)	1.523(4)
C(4)-C(5)	1.521(3)
C(5)-C(16)	1.526(4)
C(5)-C(6)	1.536(3)
C(7)-C(8)	1.533(3)
C(7)-C(15)	1.545(3)
C(7)-C(14)	1.548(3)
C(8)-C(9)	1.397(3)
C(8)-C(13)	1.403(3)
C(9)-C(10)	1.388(4)
C(10)-C(11)	1.380(4)
C(11)-C(12)	1.390(3)
C(12)-C(13)	1.386(3)
C(17)-C(18)	1.536(2)
C(18)-C(19)	1.505(3)

C(18)-C(23)	1.510(3)
C(18)-C(24)	1.563(2)
C(19)-C(20)	1.326(3)
C(20)-C(32)	1.500(3)
C(20)-C(21)	1.501(3)
C(21)-C(22)	1.482(3)
C(22)-C(23)	1.317(3)
C(24)-C(25)	1.508(2)
C(25)-C(26)	1.392(3)
C(25)-C(30)	1.392(3)
C(26)-C(27)	1.390(3)
C(27)-C(28)	1.379(4)
C(28)-C(29)	1.375(4)
C(29)-C(30)	1.393(3)
C(17)-O(1)-C(1)	116.96(14)
C(19)-O(3)-C(31)	115.85(16)
O(1)-C(1)-C(6)	109.12(15)
O(1)-C(1)-C(2)	108.38(15)
C(6)-C(1)-C(2)	112.53(15)
C(1)-C(2)-C(3)	106.58(18)
C(1)-C(2)-C(7)	113.66(15)
C(3)-C(2)-C(7)	114.49(18)
C(4)-C(3)-C(2)	112.12(19)
C(5)-C(4)-C(3)	112.49(17)
C(4)-C(5)-C(16)	113.22(17)
C(4)-C(5)-C(6)	108.79(18)
C(16)-C(5)-C(6)	111.28(19)
C(1)-C(6)-C(5)	111.38(17)
C(8)-C(7)-C(15)	111.84(18)
C(8)-C(7)-C(14)	105.0(2)
C(15)-C(7)-C(14)	106.44(17)
C(8)-C(7)-C(2)	112.32(16)
C(15)-C(7)-C(2)	110.44(19)
C(14)-C(7)-C(2)	110.51(18)
C(9)-C(8)-C(13)	116.3(2)
C(9)-C(8)-C(7)	122.24(18)

C(13)-C(8)-C(7)	121.22(19)
C(10)-C(9)-C(8)	122.0(2)
C(11)-C(10)-C(9)	120.7(2)
C(10)-C(11)-C(12)	118.7(2)
C(13)-C(12)-C(11)	120.5(2)
C(12)-C(13)-C(8)	121.9(2)
O(2)-C(17)-O(1)	123.76(17)
O(2)-C(17)-C(18)	121.51(16)
O(1)-C(17)-C(18)	114.72(15)
C(19)-C(18)-C(23)	111.51(14)
C(19)-C(18)-C(17)	107.51(15)
C(23)-C(18)-C(17)	106.30(15)
C(19)-C(18)-C(24)	109.70(15)
C(23)-C(18)-C(24)	109.67(15)
C(17)-C(18)-C(24)	112.12(14)
C(20)-C(19)-O(3)	122.50(17)
C(20)-C(19)-C(18)	125.42(16)
O(3)-C(19)-C(18)	111.56(14)
C(19)-C(20)-C(32)	123.84(18)
C(19)-C(20)-C(21)	120.76(18)
C(32)-C(20)-C(21)	115.34(17)
C(22)-C(21)-C(20)	114.60(16)
C(23)-C(22)-C(21)	123.78(17)
C(22)-C(23)-C(18)	123.40(18)
C(25)-C(24)-C(18)	112.22(14)
C(26)-C(25)-C(30)	118.12(18)
C(26)-C(25)-C(24)	120.54(16)
C(30)-C(25)-C(24)	121.30(18)
C(27)-C(26)-C(25)	121.0(2)
C(28)-C(27)-C(26)	120.2(2)
C(29)-C(28)-C(27)	119.6(2)
C(28)-C(29)-C(30)	120.6(2)
C(25)-C(30)-C(29)	120.5(2)

Table S13. Anisotropic displacement parameters (Å^2 x 10^3) for cdv99. The anisotropic displacement factor exponent takes the form: -2p^2[h^2 a*^{2}U^{11} + ... + 2 h k a* b* U^{12}]

	U^{11}	U^{22}	U^{33}	U^{23}	U^{13}	U^{12}
O(1)	24(1)	31(1)	18(1)	2(1)	0(1)	18(1)
O(2)	40(1)	123(2)	40(1)	42(1)	22(1)	57(1)
O(3)	31(1)	35(1)	19(1)	-8(1)	1(1)	-3(1)
C(1)	32(1)	43(1)	20(1)	2(1)	1(1)	29(1)
C(2)	48(1)	41(1)	19(1)	4(1)	4(1)	33(1)
C(3)	63(2)	64(2)	29(1)	11(1)	5(1)	51(1)
C(4)	53(1)	81(2)	26(1)	8(1)	0(1)	52(1)
C(5)	34(1)	70(2)	22(1)	-1(1)	-4(1)	37(1)
C(6)	29(1)	46(1)	25(1)	1(1)	-3(1)	25(1)
C(7)	62(1)	34(1)	23(1)	2(1)	8(1)	32(1)
C(8)	50(1)	22(1)	22(1)	2(1)	5(1)	14(1)
C(9)	56(2)	26(1)	24(1)	-4(1)	2(1)	10(1)
C(10)	44(1)	31(1)	31(1)	-3(1)	-4(1)	-1(1)
C(11)	32(1)	37(1)	36(1)	3(1)	4(1)	0(1)
C(12)	37(1)	37(1)	24(1)	1(1)	8(1)	7(1)
C(13)	42(1)	29(1)	20(1)	2(1)	3(1)	11(1)
C(14)	96(2)	40(1)	36(1)	6(1)	12(1)	45(2)
C(15)	72(2)	46(1)	27(1)	-4(1)	7(1)	38(1)
C(16)	35(1)	88(2)	33(1)	-11(1)	-11(1)	40(1)
C(17)	25(1)	45(1)	21(1)	7(1)	4(1)	21(1)
C(18)	19(1)	31(1)	15(1)	2(1)	1(1)	13(1)
C(19)	17(1)	28(1)	18(1)	-5(1)	-1(1)	9(1)
C(20)	19(1)	30(1)	22(1)	1(1)	2(1)	14(1)
C(21)	33(1)	41(1)	21(1)	2(1)	-8(1)	12(1)
C(22)	26(1)	34(1)	18(1)	-7(1)	1(1)	5(1)
C(23)	25(1)	26(1)	24(1)	-3(1)	6(1)	9(1)
C(24)	18(1)	30(1)	15(1)	-2(1)	0(1)	10(1)
C(25)	18(1)	34(1)	14(1)	-2(1)	2(1)	12(1)
C(26)	26(1)	37(1)	26(1)	-3(1)	4(1)	17(1)
C(27)	43(1)	54(1)	35(1)	6(1)	9(1)	34(1)
C(28)	31(1)	74(2)	32(1)	11(1)	7(1)	36(1)

C(29)	17(1)	66(2)	24(1)	1(1)	-1(1)	14(1)
C(30)	21(1)	39(1)	19(1)	-1(1)	1(1)	10(1)
C(31)	37(1)	51(2)	33(1)	-6(1)	-9(1)	-6(1)
C(32)	43(1)	37(1)	38(1)	7(1)	11(1)	26(1)

Table S14. Hydrogen coordinates (x 10^4) and isotropic displacement parameters (Å^2 x 10^3) for cdv99.

	x	y	z	U(eq)
H(1A)	-2119	2000	4860	32
H(2A)	-521	3007	4272	38
H(3A)	-2716	3568	4524	53
H(3B)	-1863	4234	4196	53
H(4A)	-4175	2191	4092	54
H(4B)	-2918	1896	3968	54
H(5A)	-4493	809	4554	44
H(6A)	-3119	-357	4639	36
H(6B)	-2242	309	4313	36
H(9A)	2411	5381	5075	48
H(10A)	4714	5647	5042	53
H(11A)	5480	5032	4573	51
H(12A)	3901	4174	4133	45
H(13A)	1610	3938	4163	40
H(14A)	1442	6822	4536	79
H(14B)	-274	6145	4489	79
H(14C)	657	5878	4225	79
H(15A)	500	5675	5062	67
H(15B)	-169	3961	5110	67
H(15C)	-1166	4576	4974	67
H(16A)	-5476	-1538	4333	72
H(16B)	-4645	-862	4003	72

H(16C)	-5916	-571	4118	72
H(21A)	239	-386	6018	42
H(21B)	1777	-199	5920	42
H(22A)	1936	2147	5918	37
H(23A)	1709	2875	5435	32
H(24A)	1329	649	4726	26
H(24B)	2256	2268	4864	26
H(26A)	1940	-1226	4899	35
H(27A)	3718	-1740	5077	48
H(28A)	5868	91	5302	50
H(29A)	6247	2440	5343	46
H(30A)	4465	2966	5172	34
H(31A)	-2533	-2822	4828	79
H(31B)	-2560	-2044	5154	79
H(31C)	-2387	-3458	5164	79
H(32A)	-861	-3083	5819	55
H(32B)	714	-2741	5702	55
H(32C)	-603	-3313	5452	55

Table S15. Torsion angles [°] for cdv99.

C(17)-O(1)-C(1)-C(6)	-89.96(19)
C(17)-O(1)-C(1)-C(2)	147.18(16)
O(1)-C(1)-C(2)-C(3)	178.77(15)
C(6)-C(1)-C(2)-C(3)	58.0(2)
O(1)-C(1)-C(2)-C(7)	-54.2(2)
C(6)-C(1)-C(2)-C(7)	-174.93(16)
C(1)-C(2)-C(3)-C(4)	-56.6(2)
C(7)-C(2)-C(3)-C(4)	176.81(17)
C(2)-C(3)-C(4)-C(5)	57.8(2)
C(3)-C(4)-C(5)-C(16)	-179.04(18)
C(3)-C(4)-C(5)-C(6)	-54.8(2)
O(1)-C(1)-C(6)-C(5)	-179.92(15)
C(2)-C(1)-C(6)-C(5)	-59.6(2)

C(4)-C(5)-C(6)-C(1)	55.2(2)
C(16)-C(5)-C(6)-C(1)	-179.38(17)
C(1)-C(2)-C(7)-C(8)	80.9(2)
C(3)-C(2)-C(7)-C(8)	-156.24(17)
C(1)-C(2)-C(7)-C(15)	-44.7(2)
C(3)-C(2)-C(7)-C(15)	78.1(2)
C(1)-C(2)-C(7)-C(14)	-162.18(18)
C(3)-C(2)-C(7)-C(14)	-39.4(2)
C(15)-C(7)-C(8)-C(9)	-17.3(3)
C(14)-C(7)-C(8)-C(9)	97.8(2)
C(2)-C(7)-C(8)-C(9)	-142.09(19)
C(15)-C(7)-C(8)-C(13)	168.81(19)
C(14)-C(7)-C(8)-C(13)	-76.2(2)
C(2)-C(7)-C(8)-C(13)	44.0(3)
C(13)-C(8)-C(9)-C(10)	0.1(3)
C(7)-C(8)-C(9)-C(10)	-174.1(2)
C(8)-C(9)-C(10)-C(11)	-0.6(3)
C(9)-C(10)-C(11)-C(12)	0.5(3)
C(10)-C(11)-C(12)-C(13)	0.0(3)
C(11)-C(12)-C(13)-C(8)	-0.4(3)
C(9)-C(8)-C(13)-C(12)	0.4(3)
C(7)-C(8)-C(13)-C(12)	174.7(2)
C(1)-O(1)-C(17)-O(2)	-0.6(3)
C(1)-O(1)-C(17)-C(18)	-179.24(16)
O(2)-C(17)-C(18)-C(19)	54.2(3)
O(1)-C(17)-C(18)-C(19)	-127.09(17)
O(2)-C(17)-C(18)-C(23)	-65.3(3)
O(1)-C(17)-C(18)-C(23)	113.38(18)
O(2)-C(17)-C(18)-C(24)	174.8(2)
O(1)-C(17)-C(18)-C(24)	-6.4(2)
C(31)-O(3)-C(19)-C(20)	75.9(3)
C(31)-O(3)-C(19)-C(18)	-112.0(2)
C(23)-C(18)-C(19)-C(20)	-8.6(2)
C(17)-C(18)-C(19)-C(20)	-124.72(19)
C(24)-C(18)-C(19)-C(20)	113.12(19)
C(23)-C(18)-C(19)-O(3)	179.59(15)

C(17)-C(18)-C(19)-O(3)	63.45(18)
C(24)-C(18)-C(19)-O(3)	-58.71(19)
O(3)-C(19)-C(20)-C(32)	2.6(3)
C(18)-C(19)-C(20)-C(32)	-168.44(17)
O(3)-C(19)-C(20)-C(21)	179.39(17)
C(18)-C(19)-C(20)-C(21)	8.4(3)
C(19)-C(20)-C(21)-C(22)	-3.6(3)
C(32)-C(20)-C(21)-C(22)	173.52(18)
C(20)-C(21)-C(22)-C(23)	-0.1(3)
C(21)-C(22)-C(23)-C(18)	-0.9(3)
C(19)-C(18)-C(23)-C(22)	4.6(3)
C(17)-C(18)-C(23)-C(22)	121.5(2)
C(24)-C(18)-C(23)-C(22)	-117.1(2)
C(19)-C(18)-C(24)-C(25)	-55.88(19)
C(23)-C(18)-C(24)-C(25)	66.9(2)
C(17)-C(18)-C(24)-C(25)	-175.25(16)
C(18)-C(24)-C(25)-C(26)	83.6(2)
C(18)-C(24)-C(25)-C(30)	-93.8(2)
C(30)-C(25)-C(26)-C(27)	0.7(3)
C(24)-C(25)-C(26)-C(27)	-176.81(17)
C(25)-C(26)-C(27)-C(28)	-0.4(3)
C(26)-C(27)-C(28)-C(29)	-0.4(3)
C(27)-C(28)-C(29)-C(30)	0.8(3)
C(26)-C(25)-C(30)-C(29)	-0.3(3)
C(24)-C(25)-C(30)-C(29)	177.19(16)
C(28)-C(29)-C(30)-C(25)	-0.4(3)

X-ray Data Collection, Structure Solution and Refinement for **20**.

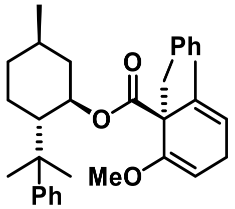

A colorless crystal of approximate dimensions 0.122 x 0.214 x 0.221 mm was mounted in a cryoloop and transferred to a Bruker Prospector APEX II system. The APEX3[1] program package was used to determine the unit-cell parameters and for data collection (4-10 sec/frame scan time). The raw frame data was processed using SAINT[2] and SADABS[3] to yield the reflection data file. Subsequent calculations were carried out using the SHELXTL[4] program package. There were no systematic absences. The noncentrosymmetric triclinic space group *P*1 was assigned and later determined to be correct.

The structure was solved by direct methods and refined on F^2 by full-matrix least-squares techniques[4]. The analytical scattering factors[5] for neutral atoms were used throughout the analysis. Hydrogen atoms were located from a difference-Fourier map and refined (x,y,z and U_{iso}). There were two molecules of the formula-unit present.

Least-squares analysis yielded wR2 = 0.0746 and Goof = 1.041 for 952 variables refined against 9320 data (0.83 Å), R1 = 0.0287 for those 9116 data with I > 2.0σ(I). The absolute structure was assigned based on the synthetic method

References.

13. APEX3 Version 2017.3-0, Bruker AXS, Inc.; Madison, WI 2017.
14. SAINT Version 8.38a, Bruker AXS, Inc.; Madison, WI 2013.
15. Sheldrick, G. M. SADABS, Version 2014/5, Bruker AXS, Inc.; Madison, WI 2014.
16. Sheldrick, G. M. SHELXTL, Version 2014/7, Bruker AXS, Inc.; Madison, WI 2014.
17. International Tables for Crystallography 1992, Vol. C., Dordrecht: Kluwer Academic Publishers

---

Definitions:

$wR2 = [\Sigma[w(F_o^2-F_c^2)^2] / \Sigma[w(F_o^2)^2]]^{1/2}$

$R1 = \Sigma||F_o|-|F_c|| / \Sigma|F_o|$

$Goof = S = [\Sigma[w(F_o^2-F_c^2)^2] / (n-p)]^{1/2}$ where n is the number of reflections and p is the total number of parameters refined.

The thermal ellipsoid plot is shown at the 50% probability level.

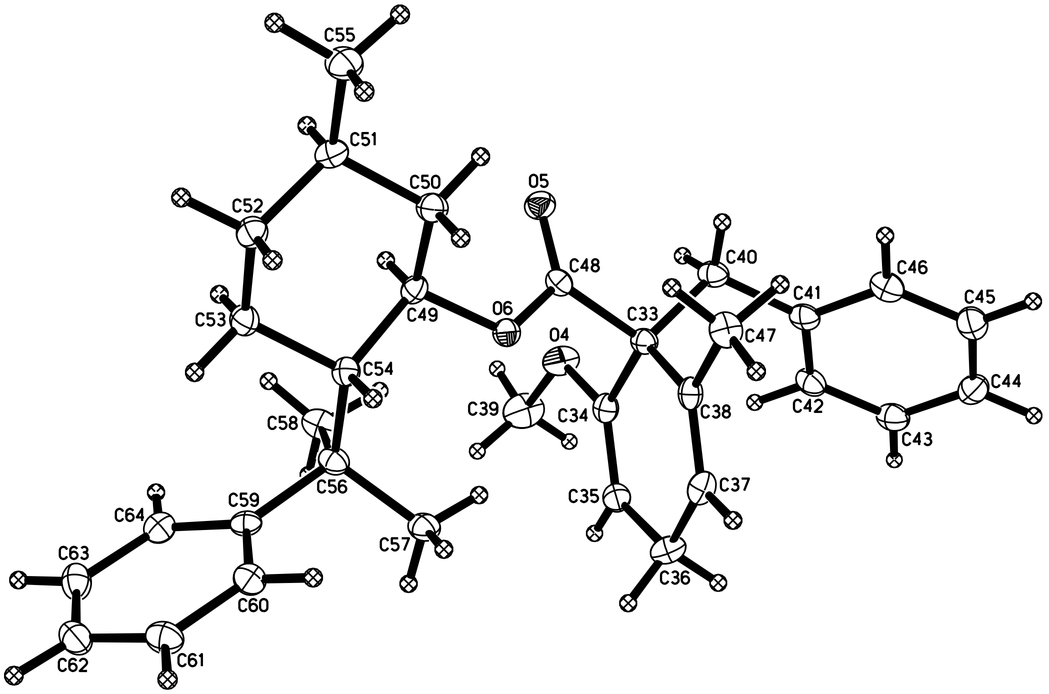
C55
C51
C50
O5
C52
C48
C40
C46
C41
C45
C53
C49
O6
C33
O4
C47
C54
C44
C39
C34
C38
C42
C58
C43
C56
C59
C35
C37
C64
C57
C63
C36
C60
C62
C61

Table S16. Crystal data and structure refinement for **20**.

Identification code	cdv90 (Hanh Nguyen)	
Empirical formula	$C_{32} H_{40} O_3$	
Formula weight	472.64	
Temperature	93(2) K	
Wavelength	1.54178 Å	
Crystal system	Triclinic	
Space group	*P*1	
Unit cell dimensions	a = 9.7653(5) Å	α = 92.583(3)°.
	b = 11.7721(5) Å	β = 112.528(2)°.
	c = 12.6007(6) Å	γ = 99.743(3)°.
Volume	1309.08(11) $Å^3$	
Z	2	
Density (calculated)	1.199 Mg/m^3	
Absorption coefficient	0.583 mm^{-1}	
F(000)	512	
Crystal color	colorless	
Crystal size	0.221 x 0.214 x 0.122 mm^3	
Theta range for data collection	3.826 to 68.875°	
Index ranges	$-11 \leq h \leq 11$, $-14 \leq k \leq 14$, $-15 \leq l \leq 15$	
Reflections collected	32324	
Independent reflections	9320 [R(int) = 0.0296]	
Completeness to theta = 67.679°	99.9 %	
Absorption correction	Semi-empirical from equivalents	
Max. and min. transmission	0.8643 and 0.8306	
Refinement method	Full-matrix least-squares on F^2	
Data / restraints / parameters	9320 / 3 / 952	
Goodness-of-fit on F^2	1.041	
Final R indices [I>2sigma(I) = 9116 data]	R1 = 0.0287, wR2 = 0.0737	
R indices (all data, 0.83 Å)	R1 = 0.0295, wR2 = 0.0746	
Extinction coefficient	0.0026(4)	
Largest diff. peak and hole	0.174 and -0.143 e.$Å^{-3}$	

Table S17. Atomic coordinates (x 10^4) and equivalent isotropic displacement parameters (Å^2 x 10^3)
for cdv90. U(eq) is defined as one third of the trace of the orthogonalized U^{ij} tensor.

	x	y	z	U(eq)
O(1)	9103(2)	1452(1)	8832(1)	22(1)
O(2)	6980(2)	860(1)	6241(1)	22(1)
O(3)	7580(2)	2822(1)	6481(1)	17(1)
C(1)	9633(2)	1862(2)	7193(2)	16(1)
C(2)	10020(2)	2210(2)	8468(2)	16(1)
C(3)	11081(2)	3106(2)	9119(2)	18(1)
C(4)	11985(3)	3917(2)	8639(2)	22(1)
C(5)	11478(2)	3670(2)	7355(2)	19(1)
C(6)	10428(2)	2777(2)	6696(2)	17(1)
C(7)	9286(3)	1676(2)	10007(2)	28(1)
C(8)	10011(3)	645(2)	7022(2)	20(1)
C(9)	11677(2)	672(2)	7330(2)	19(1)
C(10)	12744(3)	896(2)	8472(2)	21(1)
C(11)	14265(3)	924(2)	8724(2)	23(1)
C(12)	14749(3)	713(2)	7842(2)	22(1)
C(13)	13699(3)	465(2)	6709(2)	22(1)
C(14)	12179(3)	451(2)	6460(2)	20(1)
C(15)	9962(3)	2612(2)	5403(2)	23(1)
C(16)	7900(2)	1756(2)	6576(2)	16(1)
C(17)	5992(2)	2927(2)	6038(2)	16(1)
C(18)	5234(3)	2565(2)	4733(2)	20(1)
C(19)	3560(2)	2633(2)	4280(2)	19(1)
C(20)	3446(3)	3872(2)	4574(2)	20(1)
C(21)	4261(2)	4275(2)	5868(2)	19(1)
C(22)	5947(2)	4195(2)	6356(2)	16(1)
C(23)	2720(3)	2218(2)	2990(2)	28(1)
C(24)	6771(2)	4672(2)	7684(2)	17(1)
C(25)	8506(2)	4977(2)	8075(2)	19(1)
C(26)	6416(3)	3754(2)	8421(2)	20(1)
C(27)	6268(2)	5799(2)	7887(2)	16(1)
C(28)	6704(3)	6798(2)	7438(2)	21(1)
C(29)	6233(3)	7823(2)	7578(2)	25(1)

C(30)	5316(3)	7877(2)	8181(2)	27(1)
C(31)	4886(3)	6905(2)	8643(2)	25(1)
C(32)	5358(2)	5880(2)	8499(2)	19(1)
O(4)	1796(2)	8728(1)	1193(1)	23(1)
O(5)	2378(2)	9091(1)	3783(1)	20(1)
O(6)	1931(2)	7132(1)	3471(1)	16(1)
C(33)	3674(2)	8141(2)	2785(2)	15(1)
C(34)	2663(2)	7900(2)	1497(2)	16(1)
C(35)	2644(2)	7025(2)	789(2)	17(1)
C(36)	3608(3)	6144(2)	1195(2)	22(1)
C(37)	4503(2)	6310(2)	2476(2)	19(1)
C(38)	4533(2)	7173(2)	3195(2)	16(1)
C(39)	767(3)	8620(2)	7(2)	31(1)
C(40)	4773(2)	9344(2)	3062(2)	18(1)
C(41)	6074(2)	9348(2)	2689(2)	17(1)
C(42)	5862(3)	9242(2)	1525(2)	20(1)
C(43)	7085(3)	9275(2)	1211(2)	22(1)
C(44)	8544(3)	9429(2)	2048(2)	23(1)
C(45)	8775(3)	9550(2)	3208(2)	22(1)
C(46)	7550(3)	9510(2)	3520(2)	20(1)
C(47)	5433(3)	7258(2)	4483(2)	21(1)
C(48)	2601(2)	8209(2)	3414(2)	15(1)
C(49)	788(2)	7025(2)	3959(2)	15(1)
C(50)	1554(2)	7260(2)	5277(2)	18(1)
C(51)	366(2)	7230(2)	5793(2)	17(1)
C(52)	-743(2)	6065(2)	5379(2)	18(1)
C(53)	-1425(2)	5782(2)	4063(2)	18(1)
C(54)	-208(2)	5805(2)	3560(2)	15(1)
C(55)	1087(3)	7455(2)	7117(2)	23(1)
C(56)	-916(2)	5402(2)	2217(2)	17(1)
C(57)	275(3)	5079(2)	1795(2)	20(1)
C(58)	-1566(3)	6373(2)	1544(2)	21(1)
C(59)	-2128(2)	4300(2)	1993(2)	15(1)
C(60)	-1700(3)	3282(2)	2411(2)	18(1)
C(61)	-2760(3)	2279(2)	2281(2)	21(1)
C(62)	-4291(3)	2272(2)	1720(2)	21(1)
C(63)	-4741(3)	3272(2)	1284(2)	21(1)
C(64)	-3669(2)	4268(2)	1416(2)	18(1)

Table S18. Bond lengths [Å] and angles [°] for cdv90.

O(1)-C(2)	1.375(3)
O(1)-C(7)	1.429(3)
O(2)-C(16)	1.203(3)
O(3)-C(16)	1.344(3)
O(3)-C(17)	1.462(2)
C(1)-C(6)	1.519(3)
C(1)-C(2)	1.522(3)
C(1)-C(16)	1.547(3)
C(1)-C(8)	1.564(3)
C(2)-C(3)	1.326(3)
C(3)-C(4)	1.494(3)
C(3)-H(3A)	0.95(3)
C(4)-C(5)	1.498(3)
C(4)-H(4A)	1.02(3)
C(4)-H(4B)	1.02(3)
C(5)-C(6)	1.323(3)
C(5)-H(5A)	0.95(3)
C(6)-C(15)	1.507(3)
C(7)-H(7A)	0.98(3)
C(7)-H(7B)	1.01(3)
C(7)-H(7C)	1.01(3)
C(8)-C(9)	1.515(3)
C(8)-H(8A)	0.98(3)
C(8)-H(8B)	0.96(3)
C(9)-C(14)	1.393(3)
C(9)-C(10)	1.396(3)
C(10)-C(11)	1.389(3)
C(10)-H(10A)	1.01(3)
C(11)-C(12)	1.391(3)
C(11)-H(11A)	1.00(3)
C(12)-C(13)	1.384(3)
C(12)-H(12A)	0.96(3)
C(13)-C(14)	1.391(3)
C(13)-H(13A)	0.97(3)
C(14)-H(14A)	0.95(3)
C(15)-H(15A)	1.00(3)
C(15)-H(15B)	0.99(4)
C(15)-H(15C)	0.98(4)

C(17)-C(18)	1.527(3)
C(17)-C(22)	1.541(3)
C(17)-H(17A)	0.97(3)
C(18)-C(19)	1.529(3)
C(18)-H(18A)	0.99(3)
C(18)-H(18B)	0.98(3)
C(19)-C(20)	1.522(3)
C(19)-C(23)	1.525(3)
C(19)-H(19A)	1.00(3)
C(20)-C(21)	1.525(3)
C(20)-H(20A)	0.98(3)
C(20)-H(20B)	0.99(3)
C(21)-C(22)	1.543(3)
C(21)-H(21A)	1.01(3)
C(21)-H(21B)	0.98(3)
C(22)-C(24)	1.579(3)
C(22)-H(22A)	1.01(3)
C(23)-H(23A)	1.02(3)
C(23)-H(23B)	0.99(4)
C(23)-H(23C)	1.02(3)
C(24)-C(27)	1.534(3)
C(24)-C(26)	1.536(3)
C(24)-C(25)	1.544(3)
C(25)-H(25A)	0.99(3)
C(25)-H(25B)	1.01(3)
C(25)-H(25C)	0.95(3)
C(26)-H(26A)	0.94(3)
C(26)-H(26B)	0.97(3)
C(26)-H(26C)	1.00(3)
C(27)-C(32)	1.392(3)
C(27)-C(28)	1.400(3)
C(28)-C(29)	1.389(3)
C(28)-H(28A)	0.94(3)
C(29)-C(30)	1.385(4)
C(29)-H(29A)	0.95(3)
C(30)-C(31)	1.383(4)
C(30)-H(30A)	1.01(3)
C(31)-C(32)	1.391(3)
C(31)-H(31A)	0.99(3)
C(32)-H(32A)	0.97(3)

O(4)-C(34)	1.370(3)
O(4)-C(39)	1.431(3)
O(5)-C(48)	1.205(3)
O(6)-C(48)	1.343(2)
O(6)-C(49)	1.458(2)
C(33)-C(38)	1.522(3)
C(33)-C(34)	1.525(3)
C(33)-C(48)	1.546(3)
C(33)-C(40)	1.560(3)
C(34)-C(35)	1.325(3)
C(35)-C(36)	1.495(3)
C(35)-H(35A)	0.94(3)
C(36)-C(37)	1.497(3)
C(36)-H(36A)	0.96(3)
C(36)-H(36B)	1.00(3)
C(37)-C(38)	1.319(3)
C(37)-H(37A)	0.96(3)
C(38)-C(47)	1.510(3)
C(39)-H(39A)	0.99(3)
C(39)-H(39B)	1.01(3)
C(39)-H(39C)	0.99(3)
C(40)-C(41)	1.513(3)
C(40)-H(40A)	0.98(3)
C(40)-H(40B)	0.95(3)
C(41)-C(46)	1.394(3)
C(41)-C(42)	1.397(3)
C(42)-C(43)	1.390(3)
C(42)-H(42A)	1.01(3)
C(43)-C(44)	1.385(3)
C(43)-H(43A)	0.96(3)
C(44)-C(45)	1.388(3)
C(44)-H(44A)	1.02(3)
C(45)-C(46)	1.390(3)
C(45)-H(45A)	0.98(3)
C(46)-H(46A)	0.99(3)
C(47)-H(47A)	0.98(3)
C(47)-H(47B)	0.98(3)
C(47)-H(47C)	1.00(4)
C(49)-C(50)	1.527(3)
C(49)-C(54)	1.533(3)

C(49)-H(49A)	0.98(3)
C(50)-C(51)	1.529(3)
C(50)-H(50A)	1.01(3)
C(50)-H(50B)	0.98(3)
C(51)-C(52)	1.524(3)
C(51)-C(55)	1.532(3)
C(51)-H(51A)	0.99(2)
C(52)-C(53)	1.528(3)
C(52)-H(52A)	1.01(3)
C(52)-H(52B)	0.99(3)
C(53)-C(54)	1.544(3)
C(53)-H(53A)	0.97(3)
C(53)-H(53B)	0.99(3)
C(54)-C(56)	1.577(3)
C(54)-H(54A)	0.99(3)
C(55)-H(55A)	1.00(3)
C(55)-H(55B)	1.00(3)
C(55)-H(55C)	1.01(3)
C(56)-C(58)	1.533(3)
C(56)-C(59)	1.534(3)
C(56)-C(57)	1.544(3)
C(57)-H(57A)	0.99(3)
C(57)-H(57B)	0.97(3)
C(57)-H(57C)	0.99(3)
C(58)-H(58A)	0.98(3)
C(58)-H(58B)	1.03(3)
C(58)-H(58C)	0.96(3)
C(59)-C(64)	1.391(3)
C(59)-C(60)	1.395(3)
C(60)-C(61)	1.390(3)
C(60)-H(60A)	0.98(3)
C(61)-C(62)	1.386(3)
C(61)-H(61A)	0.97(3)
C(62)-C(63)	1.389(3)
C(62)-H(62A)	0.95(3)
C(63)-C(64)	1.390(3)
C(63)-H(63A)	0.96(3)
C(64)-H(64A)	0.97(3)
C(2)-O(1)-C(7)	116.67(18)
C(16)-O(3)-C(17)	118.46(16)

C(6)-C(1)-C(2)	111.50(17)
C(6)-C(1)-C(16)	109.28(16)
C(2)-C(1)-C(16)	105.72(16)
C(6)-C(1)-C(8)	111.69(17)
C(2)-C(1)-C(8)	110.04(17)
C(16)-C(1)-C(8)	108.38(17)
C(3)-C(2)-O(1)	125.76(19)
C(3)-C(2)-C(1)	124.93(19)
O(1)-C(2)-C(1)	109.32(17)
C(2)-C(3)-C(4)	122.19(19)
C(2)-C(3)-H(3A)	119.8(18)
C(4)-C(3)-H(3A)	118.0(18)
C(3)-C(4)-C(5)	113.23(18)
C(3)-C(4)-H(4A)	110.7(16)
C(5)-C(4)-H(4A)	111.4(16)
C(3)-C(4)-H(4B)	109.9(17)
C(5)-C(4)-H(4B)	109.9(16)
H(4A)-C(4)-H(4B)	101(2)
C(6)-C(5)-C(4)	125.1(2)
C(6)-C(5)-H(5A)	118.6(16)
C(4)-C(5)-H(5A)	116.3(17)
C(5)-C(6)-C(15)	121.5(2)
C(5)-C(6)-C(1)	122.22(19)
C(15)-C(6)-C(1)	116.31(19)
O(1)-C(7)-H(7A)	106.7(18)
O(1)-C(7)-H(7B)	112.3(16)
H(7A)-C(7)-H(7B)	107(2)
O(1)-C(7)-H(7C)	108.7(18)
H(7A)-C(7)-H(7C)	113(3)
H(7B)-C(7)-H(7C)	109(2)
C(9)-C(8)-C(1)	114.13(18)
C(9)-C(8)-H(8A)	111.0(16)
C(1)-C(8)-H(8A)	107.3(16)
C(9)-C(8)-H(8B)	109.5(15)
C(1)-C(8)-H(8B)	106.1(15)
H(8A)-C(8)-H(8B)	109(2)
C(14)-C(9)-C(10)	118.0(2)
C(14)-C(9)-C(8)	119.8(2)
C(10)-C(9)-C(8)	122.2(2)
C(11)-C(10)-C(9)	120.7(2)

C(11)-C(10)-H(10A)	121.4(17)
C(9)-C(10)-H(10A)	117.9(17)
C(10)-C(11)-C(12)	120.5(2)
C(10)-C(11)-H(11A)	119.0(18)
C(12)-C(11)-H(11A)	120.5(18)
C(13)-C(12)-C(11)	119.5(2)
C(13)-C(12)-H(12A)	119.7(17)
C(11)-C(12)-H(12A)	120.8(17)
C(12)-C(13)-C(14)	119.8(2)
C(12)-C(13)-H(13A)	121.3(18)
C(14)-C(13)-H(13A)	118.8(18)
C(13)-C(14)-C(9)	121.5(2)
C(13)-C(14)-H(14A)	119.3(17)
C(9)-C(14)-H(14A)	119.1(17)
C(6)-C(15)-H(15A)	109.3(19)
C(6)-C(15)-H(15B)	111(2)
H(15A)-C(15)-H(15B)	107(3)
C(6)-C(15)-H(15C)	111(2)
H(15A)-C(15)-H(15C)	107(3)
H(15B)-C(15)-H(15C)	112(3)
O(2)-C(16)-O(3)	125.17(19)
O(2)-C(16)-C(1)	125.39(19)
O(3)-C(16)-C(1)	109.43(17)
O(3)-C(17)-C(18)	110.45(16)
O(3)-C(17)-C(22)	107.69(16)
C(18)-C(17)-C(22)	111.85(17)
O(3)-C(17)-H(17A)	106.2(16)
C(18)-C(17)-H(17A)	111.2(16)
C(22)-C(17)-H(17A)	109.3(16)
C(17)-C(18)-C(19)	110.28(17)
C(17)-C(18)-H(18A)	106.8(15)
C(19)-C(18)-H(18A)	112.5(16)
C(17)-C(18)-H(18B)	110.3(16)
C(19)-C(18)-H(18B)	109.4(16)
H(18A)-C(18)-H(18B)	108(2)
C(20)-C(19)-C(23)	111.95(18)
C(20)-C(19)-C(18)	108.47(18)
C(23)-C(19)-C(18)	112.72(18)
C(20)-C(19)-H(19A)	109.9(16)
C(23)-C(19)-H(19A)	107.4(16)

C(18)-C(19)-H(19A)	106.3(15)
C(19)-C(20)-C(21)	111.80(18)
C(19)-C(20)-H(20A)	109.5(15)
C(21)-C(20)-H(20A)	109.5(15)
C(19)-C(20)-H(20B)	108.3(17)
C(21)-C(20)-H(20B)	110.7(17)
H(20A)-C(20)-H(20B)	107(2)
C(20)-C(21)-C(22)	113.19(17)
C(20)-C(21)-H(21A)	110.1(15)
C(22)-C(21)-H(21A)	106.7(15)
C(20)-C(21)-H(21B)	108.2(18)
C(22)-C(21)-H(21B)	110.0(18)
H(21A)-C(21)-H(21B)	109(2)
C(17)-C(22)-C(21)	106.29(17)
C(17)-C(22)-C(24)	115.79(16)
C(21)-C(22)-C(24)	111.74(17)
C(17)-C(22)-H(22A)	106.0(15)
C(21)-C(22)-H(22A)	110.2(14)
C(24)-C(22)-H(22A)	106.6(15)
C(19)-C(23)-H(23A)	113.0(17)
C(19)-C(23)-H(23B)	110(2)
H(23A)-C(23)-H(23B)	109(3)
C(19)-C(23)-H(23C)	109.4(17)
H(23A)-C(23)-H(23C)	105(2)
H(23B)-C(23)-H(23C)	109(3)
C(27)-C(24)-C(26)	111.74(17)
C(27)-C(24)-C(25)	106.92(17)
C(26)-C(24)-C(25)	107.53(17)
C(27)-C(24)-C(22)	108.65(16)
C(26)-C(24)-C(22)	110.27(17)
C(25)-C(24)-C(22)	111.70(17)
C(24)-C(25)-H(25A)	110.8(18)
C(24)-C(25)-H(25B)	111.0(17)
H(25A)-C(25)-H(25B)	108(2)
C(24)-C(25)-H(25C)	112.4(17)
H(25A)-C(25)-H(25C)	106(2)
H(25B)-C(25)-H(25C)	108(2)
C(24)-C(26)-H(26A)	110.5(17)
C(24)-C(26)-H(26B)	108.7(16)
H(26A)-C(26)-H(26B)	110(2)

C(24)-C(26)-H(26C)	113.3(16)
H(26A)-C(26)-H(26C)	105(2)
H(26B)-C(26)-H(26C)	110(2)
C(32)-C(27)-C(28)	117.2(2)
C(32)-C(27)-C(24)	122.66(18)
C(28)-C(27)-C(24)	120.18(19)
C(29)-C(28)-C(27)	121.6(2)
C(29)-C(28)-H(28A)	120.1(16)
C(27)-C(28)-H(28A)	118.2(16)
C(30)-C(29)-C(28)	120.1(2)
C(30)-C(29)-H(29A)	121.1(18)
C(28)-C(29)-H(29A)	118.8(18)
C(31)-C(30)-C(29)	119.3(2)
C(31)-C(30)-H(30A)	118.1(17)
C(29)-C(30)-H(30A)	122.5(17)
C(30)-C(31)-C(32)	120.4(2)
C(30)-C(31)-H(31A)	121.5(16)
C(32)-C(31)-H(31A)	118.0(16)
C(31)-C(32)-C(27)	121.4(2)
C(31)-C(32)-H(32A)	121.2(16)
C(27)-C(32)-H(32A)	117.3(15)
C(34)-O(4)-C(39)	116.64(17)
C(48)-O(6)-C(49)	117.41(15)
C(38)-C(33)-C(34)	111.55(17)
C(38)-C(33)-C(48)	109.46(16)
C(34)-C(33)-C(48)	105.93(16)
C(38)-C(33)-C(40)	111.44(17)
C(34)-C(33)-C(40)	110.48(16)
C(48)-C(33)-C(40)	107.76(16)
C(35)-C(34)-O(4)	125.79(19)
C(35)-C(34)-C(33)	124.69(19)
O(4)-C(34)-C(33)	109.53(17)
C(34)-C(35)-C(36)	122.49(19)
C(34)-C(35)-H(35A)	122.2(16)
C(36)-C(35)-H(35A)	115.3(16)
C(35)-C(36)-C(37)	113.30(18)
C(35)-C(36)-H(36A)	108.7(18)
C(37)-C(36)-H(36A)	112.5(18)
C(35)-C(36)-H(36B)	109.4(16)
C(37)-C(36)-H(36B)	110.7(15)

H(36A)-C(36)-H(36B)	102(2)
C(38)-C(37)-C(36)	125.0(2)
C(38)-C(37)-H(37A)	118.5(15)
C(36)-C(37)-H(37A)	116.4(15)
C(37)-C(38)-C(47)	121.6(2)
C(37)-C(38)-C(33)	122.51(19)
C(47)-C(38)-C(33)	115.86(18)
O(4)-C(39)-H(39A)	106.1(18)
O(4)-C(39)-H(39B)	109.4(17)
H(39A)-C(39)-H(39B)	109(2)
O(4)-C(39)-H(39C)	110.3(17)
H(39A)-C(39)-H(39C)	109(2)
H(39B)-C(39)-H(39C)	113(2)
C(41)-C(40)-C(33)	113.85(17)
C(41)-C(40)-H(40A)	110.1(17)
C(33)-C(40)-H(40A)	105.6(17)
C(41)-C(40)-H(40B)	110.2(16)
C(33)-C(40)-H(40B)	106.3(16)
H(40A)-C(40)-H(40B)	111(2)
C(46)-C(41)-C(42)	117.8(2)
C(46)-C(41)-C(40)	119.93(19)
C(42)-C(41)-C(40)	122.2(2)
C(43)-C(42)-C(41)	120.9(2)
C(43)-C(42)-H(42A)	120.7(16)
C(41)-C(42)-H(42A)	118.4(16)
C(44)-C(43)-C(42)	120.6(2)
C(44)-C(43)-H(43A)	119.9(18)
C(42)-C(43)-H(43A)	119.5(18)
C(43)-C(44)-C(45)	119.3(2)
C(43)-C(44)-H(44A)	121.0(16)
C(45)-C(44)-H(44A)	119.7(16)
C(44)-C(45)-C(46)	120.0(2)
C(44)-C(45)-H(45A)	118(2)
C(46)-C(45)-H(45A)	122(2)
C(45)-C(46)-C(41)	121.4(2)
C(45)-C(46)-H(46A)	119.8(16)
C(41)-C(46)-H(46A)	118.7(16)
C(38)-C(47)-H(47A)	110.6(16)
C(38)-C(47)-H(47B)	111.5(19)
H(47A)-C(47)-H(47B)	111(2)

C(38)-C(47)-H(47C)	112(2)
H(47A)-C(47)-H(47C)	105(3)
H(47B)-C(47)-H(47C)	107(3)
O(5)-C(48)-O(6)	124.88(18)
O(5)-C(48)-C(33)	125.48(18)
O(6)-C(48)-C(33)	109.63(16)
O(6)-C(49)-C(50)	109.80(16)
O(6)-C(49)-C(54)	108.71(15)
C(50)-C(49)-C(54)	111.40(16)
O(6)-C(49)-H(49A)	107.6(14)
C(50)-C(49)-H(49A)	109.4(14)
C(54)-C(49)-H(49A)	109.9(14)
C(49)-C(50)-C(51)	110.22(17)
C(49)-C(50)-H(50A)	110.8(14)
C(51)-C(50)-H(50A)	108.5(14)
C(49)-C(50)-H(50B)	109.1(15)
C(51)-C(50)-H(50B)	108.8(15)
H(50A)-C(50)-H(50B)	109(2)
C(52)-C(51)-C(50)	109.23(17)
C(52)-C(51)-C(55)	111.14(17)
C(50)-C(51)-C(55)	111.95(18)
C(52)-C(51)-H(51A)	107.6(14)
C(50)-C(51)-H(51A)	108.0(13)
C(55)-C(51)-H(51A)	108.8(13)
C(51)-C(52)-C(53)	112.62(17)
C(51)-C(52)-H(52A)	109.4(15)
C(53)-C(52)-H(52A)	109.3(15)
C(51)-C(52)-H(52B)	108.2(15)
C(53)-C(52)-H(52B)	109.3(15)
H(52A)-C(52)-H(52B)	108(2)
C(52)-C(53)-C(54)	112.59(17)
C(52)-C(53)-H(53A)	109.4(15)
C(54)-C(53)-H(53A)	109.2(15)
C(52)-C(53)-H(53B)	110.5(16)
C(54)-C(53)-H(53B)	108.4(16)
H(53A)-C(53)-H(53B)	107(2)
C(49)-C(54)-C(53)	105.44(16)
C(49)-C(54)-C(56)	116.38(16)
C(53)-C(54)-C(56)	112.39(16)
C(49)-C(54)-H(54A)	108.3(14)

C(53)-C(54)-H(54A)	106.8(14)
C(56)-C(54)-H(54A)	107.1(14)
C(51)-C(55)-H(55A)	111.4(16)
C(51)-C(55)-H(55B)	109.6(16)
H(55A)-C(55)-H(55B)	108(2)
C(51)-C(55)-H(55C)	110.1(15)
H(55A)-C(55)-H(55C)	109(2)
H(55B)-C(55)-H(55C)	108(2)
C(58)-C(56)-C(59)	112.23(17)
C(58)-C(56)-C(57)	107.88(17)
C(59)-C(56)-C(57)	107.29(17)
C(58)-C(56)-C(54)	110.52(17)
C(59)-C(56)-C(54)	107.28(15)
C(57)-C(56)-C(54)	111.62(17)
C(56)-C(57)-H(57A)	108.7(18)
C(56)-C(57)-H(57B)	111.3(16)
H(57A)-C(57)-H(57B)	109(2)
C(56)-C(57)-H(57C)	112.2(17)
H(57A)-C(57)-H(57C)	107(2)
H(57B)-C(57)-H(57C)	109(2)
C(56)-C(58)-H(58A)	111.7(17)
C(56)-C(58)-H(58B)	108.7(16)
H(58A)-C(58)-H(58B)	108(2)
C(56)-C(58)-H(58C)	111.4(17)
H(58A)-C(58)-H(58C)	108(2)
H(58B)-C(58)-H(58C)	108(2)
C(64)-C(59)-C(60)	117.24(19)
C(64)-C(59)-C(56)	122.97(18)
C(60)-C(59)-C(56)	119.75(19)
C(61)-C(60)-C(59)	121.9(2)
C(61)-C(60)-H(60A)	118.9(18)
C(59)-C(60)-H(60A)	119.1(18)
C(62)-C(61)-C(60)	119.9(2)
C(62)-C(61)-H(61A)	120.3(17)
C(60)-C(61)-H(61A)	119.8(17)
C(61)-C(62)-C(63)	119.1(2)
C(61)-C(62)-H(62A)	121.3(18)
C(63)-C(62)-H(62A)	119.6(18)
C(62)-C(63)-C(64)	120.4(2)
C(62)-C(63)-H(63A)	120.4(16)

C(64)-C(63)-H(63A)	119.1(16)
C(63)-C(64)-C(59)	121.4(2)
C(63)-C(64)-H(64A)	118.6(18)
C(59)-C(64)-H(64A)	119.9(18)

Table S19. Anisotropic displacement parameters ($Å^2 \times 10^3$) for cdv90. The anisotropic displacement factor exponent takes the form: $-2\pi^2[h^2 a^{*2}U^{11} + ... + 2 h k a^* b^* U^{12}]$

	U^{11}	U^{22}	U^{33}	U^{23}	U^{13}	U^{12}
O(1)	25(1)	22(1)	18(1)	3(1)	10(1)	-1(1)
O(2)	20(1)	18(1)	25(1)	-1(1)	7(1)	4(1)
O(3)	15(1)	18(1)	18(1)	0(1)	5(1)	5(1)
C(1)	15(1)	18(1)	16(1)	1(1)	6(1)	5(1)
C(2)	16(1)	18(1)	18(1)	6(1)	7(1)	6(1)
C(3)	19(1)	21(1)	14(1)	2(1)	5(1)	5(1)
C(4)	19(1)	23(1)	20(1)	0(1)	6(1)	-1(1)
C(5)	18(1)	22(1)	21(1)	6(1)	10(1)	7(1)
C(6)	17(1)	20(1)	17(1)	5(1)	7(1)	8(1)
C(7)	36(1)	30(1)	19(1)	4(1)	13(1)	0(1)
C(8)	20(1)	18(1)	20(1)	0(1)	6(1)	5(1)
C(9)	21(1)	13(1)	22(1)	2(1)	7(1)	6(1)
C(10)	26(1)	20(1)	20(1)	4(1)	10(1)	9(1)
C(11)	25(1)	18(1)	23(1)	2(1)	5(1)	8(1)
C(12)	20(1)	15(1)	30(1)	5(1)	9(1)	6(1)
C(13)	29(1)	15(1)	25(1)	4(1)	15(1)	6(1)
C(14)	25(1)	16(1)	19(1)	1(1)	7(1)	6(1)
C(15)	26(1)	27(1)	20(1)	4(1)	11(1)	7(1)
C(16)	19(1)	18(1)	13(1)	1(1)	6(1)	6(1)
C(17)	14(1)	18(1)	17(1)	3(1)	6(1)	5(1)
C(18)	18(1)	23(1)	17(1)	-1(1)	6(1)	7(1)
C(19)	18(1)	23(1)	16(1)	1(1)	4(1)	6(1)
C(20)	17(1)	24(1)	16(1)	4(1)	5(1)	6(1)
C(21)	19(1)	22(1)	17(1)	2(1)	7(1)	8(1)
C(22)	18(1)	17(1)	14(1)	3(1)	7(1)	4(1)
C(23)	25(1)	36(1)	19(1)	-4(1)	3(1)	12(1)
C(24)	19(1)	17(1)	15(1)	2(1)	7(1)	5(1)
C(25)	18(1)	21(1)	17(1)	1(1)	5(1)	4(1)
C(26)	28(1)	16(1)	16(1)	2(1)	9(1)	6(1)
C(27)	17(1)	17(1)	12(1)	1(1)	2(1)	3(1)

C(28)	25(1)	21(1)	17(1)	3(1)	8(1)	4(1)
C(29)	34(1)	18(1)	20(1)	4(1)	6(1)	4(1)
C(30)	37(1)	21(1)	19(1)	1(1)	4(1)	14(1)
C(31)	29(1)	31(1)	18(1)	2(1)	10(1)	13(1)
C(32)	20(1)	20(1)	16(1)	2(1)	5(1)	4(1)
O(4)	29(1)	23(1)	16(1)	5(1)	6(1)	13(1)
O(5)	23(1)	15(1)	24(1)	1(1)	13(1)	2(1)
O(6)	17(1)	15(1)	18(1)	1(1)	10(1)	1(1)
C(33)	17(1)	14(1)	15(1)	1(1)	7(1)	1(1)
C(34)	16(1)	16(1)	18(1)	5(1)	8(1)	3(1)
C(35)	17(1)	21(1)	13(1)	2(1)	6(1)	2(1)
C(36)	28(1)	21(1)	21(1)	1(1)	13(1)	8(1)
C(37)	18(1)	21(1)	22(1)	6(1)	10(1)	6(1)
C(38)	13(1)	17(1)	19(1)	6(1)	8(1)	2(1)
C(39)	37(1)	36(1)	20(1)	7(1)	6(1)	17(1)
C(40)	22(1)	14(1)	18(1)	1(1)	10(1)	1(1)
C(41)	21(1)	10(1)	20(1)	2(1)	9(1)	0(1)
C(42)	21(1)	17(1)	19(1)	0(1)	8(1)	-2(1)
C(43)	28(1)	19(1)	22(1)	-1(1)	14(1)	-2(1)
C(44)	24(1)	16(1)	33(1)	2(1)	17(1)	3(1)
C(45)	20(1)	18(1)	27(1)	4(1)	7(1)	4(1)
C(46)	24(1)	16(1)	18(1)	4(1)	8(1)	1(1)
C(47)	21(1)	22(1)	19(1)	6(1)	7(1)	4(1)
C(48)	14(1)	15(1)	14(1)	1(1)	4(1)	2(1)
C(49)	16(1)	16(1)	17(1)	3(1)	9(1)	4(1)
C(50)	19(1)	17(1)	17(1)	0(1)	8(1)	2(1)
C(51)	21(1)	17(1)	16(1)	2(1)	9(1)	5(1)
C(52)	18(1)	21(1)	16(1)	2(1)	8(1)	3(1)
C(53)	18(1)	19(1)	17(1)	1(1)	8(1)	1(1)
C(54)	16(1)	16(1)	14(1)	3(1)	7(1)	3(1)
C(55)	27(1)	26(1)	17(1)	0(1)	11(1)	-1(1)
C(56)	19(1)	16(1)	14(1)	1(1)	6(1)	1(1)
C(57)	23(1)	19(1)	17(1)	-2(1)	10(1)	-1(1)
C(58)	27(1)	17(1)	17(1)	4(1)	6(1)	3(1)
C(59)	19(1)	16(1)	10(1)	0(1)	6(1)	4(1)
C(60)	18(1)	18(1)	17(1)	2(1)	6(1)	3(1)
C(61)	26(1)	16(1)	21(1)	3(1)	9(1)	3(1)
C(62)	22(1)	21(1)	19(1)	0(1)	9(1)	-2(1)
C(63)	16(1)	28(1)	19(1)	3(1)	5(1)	2(1)
C(64)	20(1)	20(1)	15(1)	4(1)	7(1)	6(1)

Table S20. Hydrogen coordinates (x 10^4) and isotropic displacement parameters (Å^2 x 10^3) for cdv90.

	x	y	z	U(eq)
H(3A)	11260(30)	3270(20)	9910(30)	26(7)
H(4A)	13110(30)	3930(20)	9060(20)	24(7)
H(4B)	11950(30)	4750(30)	8850(20)	29(7)
H(5A)	11940(30)	4210(20)	7000(20)	24(7)
H(7A)	8520(40)	1100(30)	10120(30)	35(8)
H(7B)	9100(30)	2470(30)	10180(20)	27(7)
H(7C)	10350(40)	1630(30)	10530(30)	34(8)
H(8A)	9630(30)	140(20)	7480(20)	22(6)
H(8B)	9440(30)	360(20)	6220(20)	16(6)
H(10A)	12380(30)	1050(30)	9100(30)	30(7)
H(11A)	15010(40)	1110(30)	9550(30)	36(8)
H(12A)	15800(30)	710(20)	8010(20)	23(7)
H(13A)	14000(30)	310(30)	6080(30)	29(7)
H(14A)	11460(30)	280(20)	5680(30)	23(7)
H(15A)	10500(40)	3290(30)	5170(30)	39(8)
H(15B)	8870(40)	2590(30)	5000(30)	42(9)
H(15C)	10250(40)	1920(30)	5170(30)	43(9)
H(17A)	5520(30)	2420(20)	6440(20)	19(6)
H(18A)	5390(30)	1770(20)	4600(20)	20(6)
H(18B)	5730(30)	3070(20)	4340(20)	22(6)
H(19A)	3100(30)	2090(20)	4700(20)	23(7)
H(20A)	2370(30)	3920(20)	4320(20)	18(6)
H(20B)	3880(30)	4390(30)	4130(20)	29(7)
H(21A)	3780(30)	3780(20)	6310(20)	21(6)
H(21B)	4160(30)	5080(30)	6000(30)	34(8)
H(22A)	6490(30)	4680(20)	5940(20)	18(6)
H(23A)	1610(40)	2270(30)	2680(30)	31(8)
H(23B)	2810(40)	1410(30)	2820(30)	42(9)
H(23C)	3170(30)	2740(30)	2540(30)	29(7)
H(25A)	9000(30)	5370(30)	8880(30)	32(7)
H(25B)	8800(30)	5510(30)	7550(30)	30(7)
H(25C)	8930(30)	4310(30)	8070(20)	25(7)

H(26A)	6780(30)	4070(20)	9200(30)	22(6)
H(26B)	6890(30)	3110(20)	8360(20)	21(6)
H(26C)	5310(30)	3470(20)	8200(20)	22(6)
H(28A)	7340(30)	6760(20)	7040(20)	19(6)
H(29A)	6580(30)	8490(30)	7280(30)	27(7)
H(30A)	4890(40)	8590(30)	8250(30)	32(7)
H(31A)	4200(30)	6900(20)	9050(20)	23(7)
H(32A)	5030(30)	5180(20)	8790(20)	19(6)
H(35A)	2030(30)	6920(20)	-10(20)	16(6)
H(36A)	4230(30)	6140(30)	770(30)	29(7)
H(36B)	2960(30)	5350(20)	940(20)	22(7)
H(37A)	5060(30)	5720(20)	2790(20)	17(6)
H(39A)	240(40)	9280(30)	-60(30)	34(8)
H(39B)	10(30)	7870(30)	-190(30)	29(7)
H(39C)	1330(30)	8690(30)	-500(30)	29(7)
H(40A)	4150(30)	9880(30)	2650(30)	27(7)
H(40B)	5150(30)	9550(20)	3870(20)	18(6)
H(42A)	4790(30)	9130(20)	920(20)	24(7)
H(43A)	6910(30)	9190(20)	410(30)	29(7)
H(44A)	9450(30)	9450(20)	1820(30)	28(7)
H(45A)	9820(40)	9690(30)	3780(30)	41(9)
H(46A)	7720(30)	9610(20)	4350(20)	21(6)
H(47A)	5930(30)	6590(20)	4680(20)	24(7)
H(47B)	4800(40)	7320(30)	4920(30)	38(8)
H(47C)	6280(40)	7950(30)	4770(30)	47(9)
H(49A)	170(30)	7610(20)	3660(20)	10(5)
H(50A)	2260(30)	8050(20)	5530(20)	12(6)
H(50B)	2140(30)	6660(20)	5570(20)	20(6)
H(51A)	-210(30)	7840(20)	5490(20)	9(5)
H(52A)	-1590(30)	6070(20)	5660(20)	20(6)
H(52B)	-200(30)	5450(20)	5730(20)	17(6)
H(53A)	-2050(30)	6330(20)	3720(20)	17(6)
H(53B)	-2100(30)	5000(20)	3830(20)	22(6)
H(54A)	410(30)	5240(20)	3940(20)	12(6)
H(55A)	1870(30)	8190(30)	7390(20)	26(7)
H(55B)	1580(30)	6800(30)	7440(20)	23(7)
H(55C)	280(30)	7500(20)	7430(20)	20(6)
H(57A)	-250(30)	4720(30)	980(30)	31(7)
H(57B)	810(30)	4530(20)	2260(20)	25(7)
H(57C)	1020(30)	5770(30)	1810(20)	27(7)

H(58A)	-2080(30)	6120(30)	720(30)	29(7)
H(58B)	-690(30)	7060(30)	1670(20)	28(7)
H(58C)	-2280(30)	6640(30)	1810(20)	26(7)
H(60A)	-620(40)	3280(30)	2830(30)	31(7)
H(61A)	-2420(30)	1590(30)	2590(30)	27(7)
H(62A)	-5030(30)	1610(30)	1650(30)	28(7)
H(63A)	-5800(30)	3290(20)	910(20)	21(6)
H(64A)	-4020(30)	4960(30)	1110(30)	30(7)

Table S21. Torsion angles [°] for cdv90.

C(7)-O(1)-C(2)-C(3)	2.0(3)
C(7)-O(1)-C(2)-C(1)	-178.07(19)
C(6)-C(1)-C(2)-C(3)	-9.3(3)
C(16)-C(1)-C(2)-C(3)	-128.0(2)
C(8)-C(1)-C(2)-C(3)	115.2(2)
C(6)-C(1)-C(2)-O(1)	170.73(17)
C(16)-C(1)-C(2)-O(1)	52.1(2)
C(8)-C(1)-C(2)-O(1)	-64.8(2)
O(1)-C(2)-C(3)-C(4)	-177.4(2)
C(1)-C(2)-C(3)-C(4)	2.6(3)
C(2)-C(3)-C(4)-C(5)	4.9(3)
C(3)-C(4)-C(5)-C(6)	-5.4(3)
C(4)-C(5)-C(6)-C(15)	178.6(2)
C(4)-C(5)-C(6)-C(1)	-1.8(3)
C(2)-C(1)-C(6)-C(5)	8.7(3)
C(16)-C(1)-C(6)-C(5)	125.2(2)
C(8)-C(1)-C(6)-C(5)	-114.8(2)
C(2)-C(1)-C(6)-C(15)	-171.64(18)
C(16)-C(1)-C(6)-C(15)	-55.1(2)
C(8)-C(1)-C(6)-C(15)	64.8(2)
C(6)-C(1)-C(8)-C(9)	45.9(2)
C(2)-C(1)-C(8)-C(9)	-78.5(2)
C(16)-C(1)-C(8)-C(9)	166.35(18)
C(1)-C(8)-C(9)-C(14)	-110.5(2)
C(1)-C(8)-C(9)-C(10)	70.5(3)
C(14)-C(9)-C(10)-C(11)	1.6(3)
C(8)-C(9)-C(10)-C(11)	-179.5(2)

C(9)-C(10)-C(11)-C(12)	-0.9(3)
C(10)-C(11)-C(12)-C(13)	-0.5(3)
C(11)-C(12)-C(13)-C(14)	1.2(3)
C(12)-C(13)-C(14)-C(9)	-0.5(3)
C(10)-C(9)-C(14)-C(13)	-0.9(3)
C(8)-C(9)-C(14)-C(13)	-179.9(2)
C(17)-O(3)-C(16)-O(2)	6.1(3)
C(17)-O(3)-C(16)-C(1)	-173.31(16)
C(6)-C(1)-C(16)-O(2)	136.4(2)
C(2)-C(1)-C(16)-O(2)	-103.5(2)
C(8)-C(1)-C(16)-O(2)	14.5(3)
C(6)-C(1)-C(16)-O(3)	-44.2(2)
C(2)-C(1)-C(16)-O(3)	76.0(2)
C(8)-C(1)-C(16)-O(3)	-166.08(17)
C(16)-O(3)-C(17)-C(18)	-74.5(2)
C(16)-O(3)-C(17)-C(22)	163.15(17)
O(3)-C(17)-C(18)-C(19)	177.94(17)
C(22)-C(17)-C(18)-C(19)	-62.1(2)
C(17)-C(18)-C(19)-C(20)	58.6(2)
C(17)-C(18)-C(19)-C(23)	-176.89(19)
C(23)-C(19)-C(20)-C(21)	178.90(19)
C(18)-C(19)-C(20)-C(21)	-56.1(2)
C(19)-C(20)-C(21)-C(22)	57.0(2)
O(3)-C(17)-C(22)-C(21)	179.54(15)
C(18)-C(17)-C(22)-C(21)	58.0(2)
O(3)-C(17)-C(22)-C(24)	-55.7(2)
C(18)-C(17)-C(22)-C(24)	-177.26(17)
C(20)-C(21)-C(22)-C(17)	-55.4(2)
C(20)-C(21)-C(22)-C(24)	177.41(18)
C(17)-C(22)-C(24)-C(27)	-165.14(17)
C(21)-C(22)-C(24)-C(27)	-43.3(2)
C(17)-C(22)-C(24)-C(26)	-42.4(2)
C(21)-C(22)-C(24)-C(26)	79.5(2)
C(17)-C(22)-C(24)-C(25)	77.2(2)
C(21)-C(22)-C(24)-C(25)	-160.97(17)
C(26)-C(24)-C(27)-C(32)	-11.0(3)
C(25)-C(24)-C(27)-C(32)	-128.4(2)
C(22)-C(24)-C(27)-C(32)	110.9(2)
C(26)-C(24)-C(27)-C(28)	169.9(2)
C(25)-C(24)-C(27)-C(28)	52.5(2)

C(22)-C(24)-C(27)-C(28)	-68.2(2)
C(32)-C(27)-C(28)-C(29)	-1.0(3)
C(24)-C(27)-C(28)-C(29)	178.2(2)
C(27)-C(28)-C(29)-C(30)	0.4(4)
C(28)-C(29)-C(30)-C(31)	0.4(4)
C(29)-C(30)-C(31)-C(32)	-0.5(4)
C(30)-C(31)-C(32)-C(27)	-0.2(3)
C(28)-C(27)-C(32)-C(31)	0.9(3)
C(24)-C(27)-C(32)-C(31)	-178.2(2)
C(39)-O(4)-C(34)-C(35)	0.9(3)
C(39)-O(4)-C(34)-C(33)	-179.42(19)
C(38)-C(33)-C(34)-C(35)	-6.7(3)
C(48)-C(33)-C(34)-C(35)	-125.7(2)
C(40)-C(33)-C(34)-C(35)	117.9(2)
C(38)-C(33)-C(34)-O(4)	173.69(16)
C(48)-C(33)-C(34)-O(4)	54.7(2)
C(40)-C(33)-C(34)-O(4)	-61.8(2)
O(4)-C(34)-C(35)-C(36)	-178.8(2)
C(33)-C(34)-C(35)-C(36)	1.6(3)
C(34)-C(35)-C(36)-C(37)	3.7(3)
C(35)-C(36)-C(37)-C(38)	-3.6(3)
C(36)-C(37)-C(38)-C(47)	178.2(2)
C(36)-C(37)-C(38)-C(33)	-1.9(3)
C(34)-C(33)-C(38)-C(37)	6.7(3)
C(48)-C(33)-C(38)-C(37)	123.6(2)
C(40)-C(33)-C(38)-C(37)	-117.3(2)
C(34)-C(33)-C(38)-C(47)	-173.43(17)
C(48)-C(33)-C(38)-C(47)	-56.5(2)
C(40)-C(33)-C(38)-C(47)	62.6(2)
C(38)-C(33)-C(40)-C(41)	49.3(2)
C(34)-C(33)-C(40)-C(41)	-75.3(2)
C(48)-C(33)-C(40)-C(41)	169.41(17)
C(33)-C(40)-C(41)-C(46)	-112.9(2)
C(33)-C(40)-C(41)-C(42)	69.8(3)
C(46)-C(41)-C(42)-C(43)	1.1(3)
C(40)-C(41)-C(42)-C(43)	178.53(19)
C(41)-C(42)-C(43)-C(44)	-0.8(3)
C(42)-C(43)-C(44)-C(45)	0.0(3)
C(43)-C(44)-C(45)-C(46)	0.4(3)
C(44)-C(45)-C(46)-C(41)	0.0(3)

C(42)-C(41)-C(46)-C(45)	-0.7(3)
C(40)-C(41)-C(46)-C(45)	-178.21(19)
C(49)-O(6)-C(48)-O(5)	3.6(3)
C(49)-O(6)-C(48)-C(33)	-175.41(16)
C(38)-C(33)-C(48)-O(5)	139.3(2)
C(34)-C(33)-C(48)-O(5)	-100.3(2)
C(40)-C(33)-C(48)-O(5)	18.0(3)
C(38)-C(33)-C(48)-O(6)	-41.6(2)
C(34)-C(33)-C(48)-O(6)	78.75(19)
C(40)-C(33)-C(48)-O(6)	-162.99(16)
C(48)-O(6)-C(49)-C(50)	-77.9(2)
C(48)-O(6)-C(49)-C(54)	159.95(16)
O(6)-C(49)-C(50)-C(51)	176.23(16)
C(54)-C(49)-C(50)-C(51)	-63.3(2)
C(49)-C(50)-C(51)-C(52)	56.2(2)
C(49)-C(50)-C(51)-C(55)	179.72(18)
C(50)-C(51)-C(52)-C(53)	-53.1(2)
C(55)-C(51)-C(52)-C(53)	-177.08(18)
C(51)-C(52)-C(53)-C(54)	55.9(2)
O(6)-C(49)-C(54)-C(53)	-177.47(16)
C(50)-C(49)-C(54)-C(53)	61.4(2)
O(6)-C(49)-C(54)-C(56)	-52.2(2)
C(50)-C(49)-C(54)-C(56)	-173.28(17)
C(52)-C(53)-C(54)-C(49)	-57.4(2)
C(52)-C(53)-C(54)-C(56)	174.80(17)
C(49)-C(54)-C(56)-C(58)	-44.8(2)
C(53)-C(54)-C(56)-C(58)	76.9(2)
C(49)-C(54)-C(56)-C(59)	-167.48(17)
C(53)-C(54)-C(56)-C(59)	-45.8(2)
C(49)-C(54)-C(56)-C(57)	75.3(2)
C(53)-C(54)-C(56)-C(57)	-163.03(17)
C(58)-C(56)-C(59)-C(64)	-10.9(3)
C(57)-C(56)-C(59)-C(64)	-129.2(2)
C(54)-C(56)-C(59)-C(64)	110.7(2)
C(58)-C(56)-C(59)-C(60)	171.42(19)
C(57)-C(56)-C(59)-C(60)	53.1(2)
C(54)-C(56)-C(59)-C(60)	-67.0(2)
C(64)-C(59)-C(60)-C(61)	-1.1(3)
C(56)-C(59)-C(60)-C(61)	176.69(19)
C(59)-C(60)-C(61)-C(62)	0.1(3)

C(60)-C(61)-C(62)-C(63)	0.7(3)
C(61)-C(62)-C(63)-C(64)	-0.4(3)
C(62)-C(63)-C(64)-C(59)	-0.6(3)
C(60)-C(59)-C(64)-C(63)	1.4(3)
C(56)-C(59)-C(64)-C(63)	-176.37(19)

5. References

(1) Egan, B. A.; Paradowski, M.; Thomas, L. H.; Marquez, R. Regiocontrolled Rearrangement of Isobenzofurans. *Org. Lett.* **2011**, *13* (8), 2086–2089. https://doi.org/10.1021/ol200498k.

(2) Crossley, S. W. M.; Martinez, R. M.; Guevara-Zuluaga, S.; Shenvi, R. A. Synthesis of the Privileged 8-Arylmenthol Class by Radical Arylation of Isopulegol. *Org. Lett.* **2016**, *18* (11), 2620–2623. https://doi.org/10.1021/acs.orglett.6b01047.

(3) Bandgar, B. P.; Sarangdhar, R. J.; Viswakarma, S.; Ahamed, F. A. Synthesis and Biological Evaluation of Orally Active Prodrugs of Indomethacin. *J. Med. Chem.* **2011**, *54* (5), 1191–1201. https://doi.org/10.1021/jm101085j.
